# Rhodium‐Catalyzed Cyclization of Terminal and Internal Allenols: An Atom Economic and Highly Stereoselective Access Towards Tetrahydropyrans

**DOI:** 10.1002/anie.202009166

**Published:** 2020-10-26

**Authors:** Johannes P. Schmidt, Bernhard Breit

**Affiliations:** ^1^ Institute for Organic Chemistry Albert-Ludwigs-Universität Freiburg Albertstraße 21 79104 Freiburg im Breisgau Germany

**Keywords:** allenes, cyclizations, diastereoselectivity, heterocycles, rhodium

## Abstract

A comprehensive study of a diastereoselective Rh‐catalyzed cyclization of terminal and internal allenols is reported. The methodology allows the atom economic and highly *syn*‐selective access to synthetically important 2,4‐disubstituted and 2,4,6‐trisubstituted tetrahydropyrans (THP). Furthermore, its utility and versatility are demonstrated by a great functional‐group compatibility and the enantioselective total synthesis of (−)‐centrolobine.

## Graphical abstract

A comprehensive study of a diastereoselective Rh‐catalyzed cyclization of terminal and internal allenols is reported. The methodology allows the atom economic and highly *syn*‐selective access to synthetically important 2,4‐disubstituted, and 2,4,6‐trisubstituted tetrahydropyrans. Furthermore, its utility and versatility are demonstrated by a great functional‐group compatibility and the enantioselective total synthesis of (−)‐centrolobine.

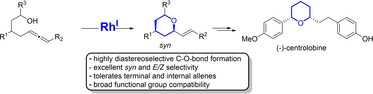

## Introduction

**Figure 1:**
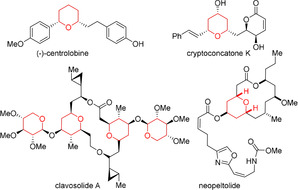
Natural products containing THP moieties as structural key elements.

Oxygen containing heterocycles, in particular substituted THP scaffolds, are important structural elements and building blocks in natural and bioactive products like centrolobine,^[1]^ cryptoconcatone K,^[2]^ clavosolide A^[3]^ and neopeltolide (Figure 1).^[4]^

**Scheme 1:**
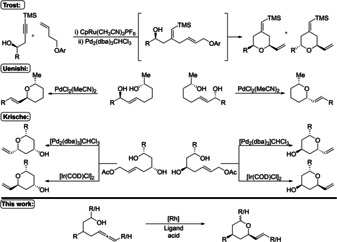
Representative examples of transition‐metal‐mediated methodologies for THP synthesis.[^13^, ^15^] Cp=cyclopentadiene, dba=dibenzylidenacetone, COD=1,5‐cyclooctadiene.

Due to their high variety, biological properties and potential use in pharmaceuticals, the synthesis of THP was tackled repeatedly, resulting in various methods allowing their stereoselective access.^[5]^ Some of the more recent approaches focused mainly on the cyclization onto oxocarbenium ions,^[6]^ oxa‐Michael reactions,^[7]^ hetero‐Diels–Alder cycloaddition,^[8]^ reduction of cyclic hemi ketals,^[9]^ gold catalyzed [2+2+2] cycloadditions^[10]^ and gold catalyzed cyclisation.^[11]^ Besides these concepts, the transition metal catalyzed substitution reaction of allylic alcohols and esters started to emerge. Trost, displayed his approach in 2002.^[13]^ He focused on a combined Ru‐catalyzed ene‐yne reaction followed by a Pd‐catalyzed substitution reaction to access the THP scaffold (Scheme 1).^[13]^ Uenishi et al. described a methodology based on a 1,3‐chirality transfer starting from 2,8‐diols.^[13]^ Krische demonstrated a catalyst‐directed diastereoselective formation of different tetrahydropyrans starting from 1,3‐diols using a chiral palladium‐ or an iridium‐based catalyst system.^[15]^

Despite their respective benefits and their high selectivity, all these methods represent versions of transition‐metal‐catalyzed substitution reactions^[16]^ and therefore undermine the principle of atom economy^[17]^ by generating stoichiometric amounts of waste (Scheme 1). An atom economic alternative to this method, is the transition‐metal‐catalyzed addition of nucleophiles to allenes and alkynes. This reaction was studied by our group intensively and represents a powerful tool to construct C−O, C−S, C−N and C−C bonds in a highly regio‐, enantio‐, and diastereoselctive fashion.[^18^, ^19^, ^20^] In addition to the broad range of different nucleophiles, the utility of this methodology has been demonstrated in various natural and bioactive product syntheses.^[19]^

Herein, we present our atom economic and stereoselective strategy to access the THP moiety by employing terminal and internal allenols as key units in an intramolecular rhodium catalyzed cyclization.

## Results and Discussion

Our study commenced by investigating terminal allenes, drafting the phenyl substituted allenol **1** as model substrate (Table 1). Primary reactivity examinations employing a combination of [Rh(COD)Cl]_2_ and bis[(2‐diphenylphosphino)phenyl] ether (DPEphos) were not successful (entry 1). Switching from DPEphos to combination of Bis(diphenylphosphino)ferrocene (dppf) and chloroacetic acid as additive, delivered the *syn*‐configurated adduct in excellent 92 % and a satisfactory diastereoselectivity of 83/17 (entry 3).^[21]^

**Table 1:** Optimization of reaction conditions for terminal allenes.^[22]^ 
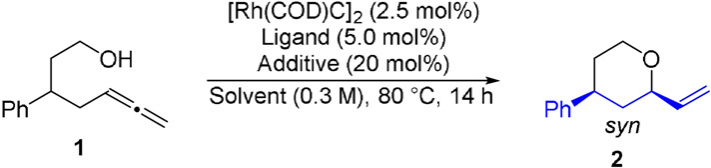

Entry	Ligand	Additive	Solvent	Yield [%]^[a]^	*syn*/*anti* ^[b]^
1	DPEphos	–	DCE	–	–
2	DPEphos	ClCH_2_CO_2_H	DCE	68	86/14
3	dppf	ClCH_2_CO_2_H	DCE	92	83/17
4	dppf	Diphenyl phosphate	DCE	87	93/7
5	dppf	Diphenyl phosphate	DCM	92	95/5

All reactions were performed on a 0.3 mmol scale. [a] Yield of isolated diastereomeric mixture. [b] Selectivity determined by ^1^H NMR analysis of the crude reaction mixture. dppf=1,1‐bis(diphenylphosphino)ferrocene, DCE=1,2‐dichloroethane, DCM=dichloromethane.

Further optimizations resulted in the finalized conditions, employing a combination of [Rh(COD)Cl]_2,_ dppf and diphenyl phosphate in DCM. These modifications provided **2** in an excellent yield and d.r. (Table 1, entry 5).^[23]^

**Figure 2:**
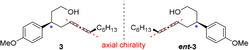
Unsymmetric internal allenols: Point and axial chirality.

After the successful optimization for terminal allenes, we focused on the ring closure of unsymmetrical internal allenols (Table 2). These compounds display point and axial chirality (Figure 2) and are therefore employed as a diastereomeric mixture (1/1) in the catalysis.^[22]^

**Table 2:** Optimization of reaction conditions for internal allenes.^[22]^ 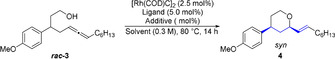

Entry	Ligand	Additive (mol %)	Solvent	Yield [%]^[a]^	*E*/*Z* ^[b]^	*syn/anti* ^[b]^
1	dppf	Diphenyl phosphate (20)	DCE	98	79/21	70/30
2	dppf	PTSA (20)	DCE	98	70/30	92/8
3	**L1**	PTSA (20)	DCE	89	65/35	94/6
4	**L2**	PTSA (20)	DCE	96	84/16	91/9
5	**L3**	PTSA (20)	DCE	65	80/20	92/8
6	**L2**	PTSA (20)	PhF	94	83/17	90/10
7	**L2**	PTSA (20)	PhF	96	84/16	95/5


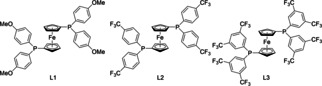

All reactions were performed on a 0.3 mmol scale[a] Yield of isolated diastereomeric mixture. [b] Selectivity determined by ^1^H NMR analysis of the crude reaction mixture. PhF=fluorobenzene, PTSA=p‐toluenesulfonic acid monohydrate.

In order to obtain a divergent reaction starting from the diastereomeric mixture ***rac*** **‐3**, an initial isomerization of the allene axis is required prior to nucleophilic addition. In earlier studies, our group found that in the presence of a transition metal, the racemization of enantiomerically enriched internal allenes takes place. Based on this knowledge, we propose that the epimerization of ***rac*** **‐3** (Table 2) follows a similar mechanism.[^18a^, ^18k^, ^18m^]

An initial experiment was conducted using the diastereomeric mixture of 3‐(4‐methoxyphenyl)trideca‐5,6‐dien‐1‐ol (**3**) as screening substrate. Employing the conditions previously developed for terminal allenes (Table 2, entry 5) delivered the corresponding THP compound **4** in excellent yield, albeit low d.r. and *E*/*Z* ratio. In this respect, we examined a variety of different additives and were delighted to find that PTSA provided a promising result.^[21]^ To increase the selectivity, derivatives of dppf with different steric and electronic properties were evaluated.^[24]^ The derivative bearing an electron donating methoxy group (**L1**) delivered an excellent d.r. but poor *E*/*Z* selectivity. Employing the dppf derivative **L2**, a ligand substituted with an electron withdrawing CF_3_‐group, increased the *E*/*Z* ratio to 84/16 while maintaining a good yield and d.r. Final optimizations were carried out by replacing the solvent to PhF and increasing the amount of additive to 30 mol % (entry 7).^[22]^ These adjustments allowed access to compound **4** in 96 % yield, an *E*/*Z* ratio of 84/16 and a perfect d.r. of 95/5 (entry 7).

Furthermore, the highly diastereo convergent cyclization of ***rac***‐**3** (Table 2) indicates, that an epimerization of the allene moiety must take place. At this point the determination of relative configuration of compound **2** and **4** was accomplished by NOE experiments.^[22]^

With the optimized conditions in hand, the scope and limitations of this reaction were explored (Table 3 and 4). First, a variety of 3‐substituted terminal allenols were subjected to the catalysis conditions. Straight‐chain and cyclic alkyl functionalized alcohols behaved well and furnished the corresponding *syn*‐tetrahydropyrans **5** to **9** in excellent yield and d.r. Next, substrates bearing phenyl, naphthyl and biaryl groups were investigated and provided the corresponding THP in yields up to 97 % and a d.r. up to 96/4. Notably, even sterically high congested substrates reacted well (**7** and **16**). Functional groups like CF_3_, halides, ether and thioether attached to the aromatic ring were compatible and afforded the desired adducts **18** to **21** in good to excellent yields.

**Table 3:** Scope of the catalytic diastereoselective cyclization of terminal allenols towards *syn*‐tetrahydropyrans.

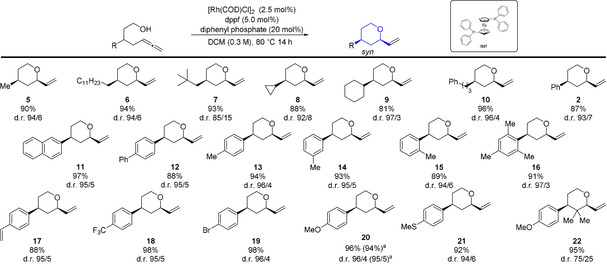

All reactions were performed on a 0.3 mmol scale. Yield of isolated diastereomeric mixture. Selectivity determined by ^1^H NMR analysis of the crude reaction mixture. [a] Yield and d.r. of large‐scale reaction (1.4 mmol). DCM=dichloromethane.

Next, different functionalized internal allenols were investigated (Table 4). Reactants substituted with linear and branched aliphatic chains (**23**–**25**) as well as cycloaliphatic (**26**, **27**) and PhC_3_ residues (**28**) transformed smoothly in good to excellent yields. Aromatic compounds, displaying higher steric hinderance and different electronic properties, provided the corresponding adducts in comparable yield and diastereoselectivities to terminal allenes. The *E*/*Z* ratio showed to be consistent across the different functionalization pattern (up to 89/11), albeit a slight deterioration was observed for more bulky (**25**) and halide substituted (**37**) compounds. The alteration of the allene moiety provided excellent results as well (**41** to **44**). Compounds **4** and **20** (Tables 3 and 4) were synthesized by large‐scale catalysis without any decline in yield and d.r.

**Table 4:** Scope of the catalytic diastereoselective cyclization of internal allenols towards *syn*‐tetrahydropyrans.

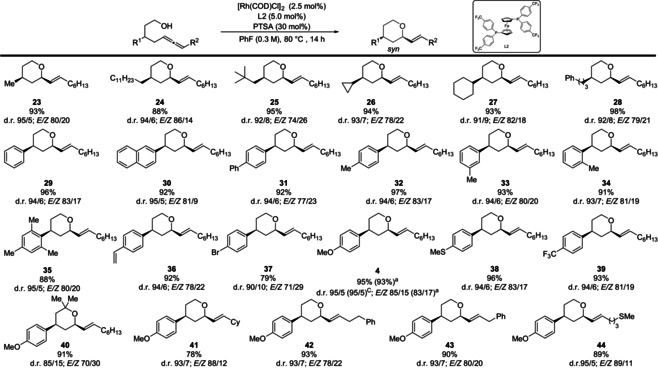

All reactions were performed on a 0.3 mmol scale. Yield of isolated diastereomeric mixture. Selectivity determined by ^1^H NMR analysis of the crude reaction mixture. [a] Yield and d.r. of large‐scale reaction (1.4 mmol).

Tetrahydropyran as part of natural products are commonly substituted in 2,4,6‐position (Figure 1). Therefore, we attempted the access to these scaffolds, by employing the more complex *syn*‐ and *anti*‐1,3‐disubstitued allenols (Tables 5 and 6). First, the *syn*‐1,3‐disubstituted terminal and internal hydroxy allenes **45**–**48** were subjected to the catalysis conditions. The terminal as well as the internal allenes provided the thermodynamically favoured all *equatorial*‐THP compounds **45A**–**48A** in superb yields and diastereoselectivity (Table 5). Further the *anti*‐1,3‐substituted allenols were examined. The terminal allenes **49** and **50** proceeded smoothly and provided the 4,6‐*syn* configurated compound **49A** and **50A** in up to 94 % and a diastereoselectivity up to 95/5. Similar results were obtained for the *anti*‐4,6‐substitued internal allene **51** and **52** (Table 6, entries 3 and 4). However, a deterioration in diastereoselectivity occurs when the substituent in 1‐position is changed from methyl (**49**, **51**) to phenyl (**50**, **52**).

**Table 5:** Scope of *syn‐1*,*3*‐substituted allenols.^[25]^ 
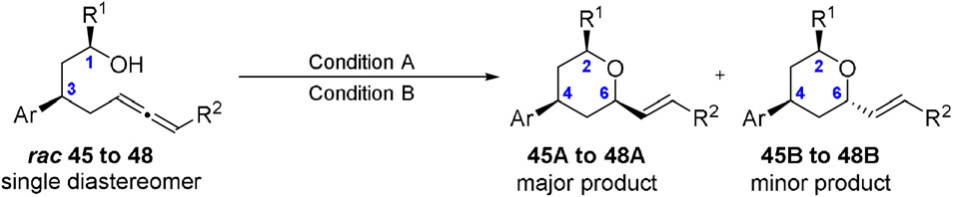

Entry	Substrate	R^1^	R^2^	Cond.	Yield [%]^[a]^	A/B^[b]^
1	45	Me	H	A	93	95/5
2	46	Ph	H	A	96	93/7
3	47	Me	C_6_H_13_	B	91	95/5
4	48	Ph	C_6_H_13_	B	90	95/5

Conditions A: [Rh(COD)Cl]_2_ (2.5 mol %); dppf (5.0 mol %); diphenyl phosphate (20 mol %); DCM (0.3 M), 80 °C, 14 h; Conditions B: [Rh(COD)Cl]_2_ (2.5 mol %); **L2** (5.0 mol); PTSA (30 mol %); PhF (0.3 M), 80 °C, 14 h. [a] Combined yield. [b] A/B ratio determined by ^1^H NMR analysis of the crude reaction mixture. Ar=4‐methoxyphenyl.

**Table 6:** Scope of *anti‐1*,*3*‐substituted allenols.^[25]^ 
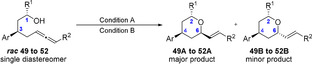

Entry	Substrate	R^1^	R^2^	Cond.	Yield [%]^[a]^	A/B^[b]^
1	49	Me	H	A	92	95/5
2	50	Ph	H	A	94	85/15
3	51	Me	C_6_H_13_	B	92	80/20
4	52	Ph	C_6_H_13_	B	88	73/27

Conditions A: [Rh(COD)Cl]_2_ (2.5 mol %); dppf (5.0 mol %); diphenyl phosphate (20 mol %); DCM (0.3 M), 80 °C, 14 h; Conditions B: [Rh(COD)Cl]_2_ (2.5 mol %); L2 (5.0 mol %); PTSA (30 mol %); PhF (0.3 M), 80 °C, 14 h. [a] Combined yield. [b] A/B ratio determined by ^1^H NMR analysis of the crude reaction mixture.

**Scheme 2:**
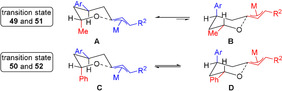
Presumed transition state. Ar=4‐methoxyphenyl, R=H or C_6_H_13_).

It is conceivable that, due to the Ph group, a decrease of energetic difference between transition states **C** and **D** (Scheme 2) compared to the more favored transition state **A**, for the methyl‐substituted compound **49** and **51** occurs. This lower energetic difference can account for the decrease in selectivity between **49**,**51** and **50**,**52** (Scheme 2).

**Scheme 3:**
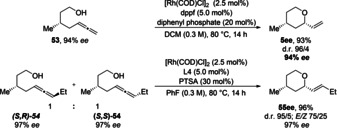
Enabling the stereoselective synthesis of **5** ***ee*** and **55** ***ee***, by employing enantiomerically enriched starting material **53** and **54**.

To enable the synthesis of enantioenriched tetrahydropyrans, the enantiomerically enriched starting materials **53** and **54** were subjected to the catalysis conditions.^[26]^ We were satisfied to observe that the enantiomeric purity was maintained for both, the terminal and internal allene (Scheme 3).

An alternative to the method shown in Table 4, can be the application of the commercially available dppf ligand, in combination with PTSA, followed by an in situ hydrogenation. This procedure allows the straightforward selective access of saturated THPs in high yields and diastereoselectivites (Table 7).

**Table 7:** Catalysis reaction followed by in situ hydrogenation.

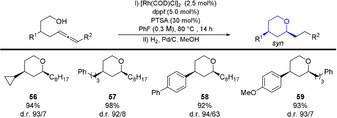

All reactions were performed on a 0.3 mmol scale. Yield of isolated diastereomeric mixture. Selectivity determined by ^1^H NMR analysis of the crude reaction mixture.

**Scheme 4:**
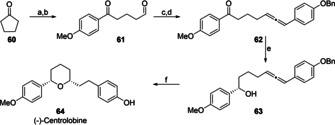
Total synthesis of (−)‐centrolobine. a] I) Methoxyphenylmagnesium bromide (1.0 equiv), THF,; II) PTSA (30 mol %), Tol, reflux, 2 h, 95 %; b] I) O_3_, MeOH, −78 °C; II) SMe_2_ (5.0 equiv), −78 °C to rt., 2 h, 83 %; c] I) Ethynylmagnesium bromide (1.0 equiv), 0 °C to rt., 2 h; II) AcCl (1.0 equiv), 0 °C to rt., 2 h, 85 %; d) (4‐(Benzyloxy)phenyl)magnesium bromide (1.0 equiv), CuBr (10 mol %) THF, 0 °C to rt., 2 h 48 %; e) (+)‐CBS (1.0 equiv), BH_3_⋅THF (2.0 equiv), THF/MeOH, −20 °C, 5 h, 85 %; f] I) [Rh(COD)Cl]_2_ (2.5 mol %), dppf (5.0 mol %), PTSA (30 mol %), PhF (0.3 M); 80 °C, 14 h 83 %, d.r. 90/10; II) Pd/C (5.0 mol %), H_2_ (1 atm), MeOH/DCM (1/1), rt. 72 h, 84 %, d.r. 90/10, 90 % *ee*.

To highlight the synthetic utility of this catalytic reaction, the enantioselective total synthesis of (−)‐centrolobine (**64**), starting from cyclopentanone (**60**), was pursued (Scheme 4).

First a Grignard addition followed by elimination and ozonolysis furnished the oxopentanal **61**. Next, the addition of ethenylmagnesium bromide in combination with an S_N_2′ reaction enabled the synthesis of allene **62** which was further reduced to allenol **63**, by CBS reduction. Employing the conditions developed in Table 7, the enantiomerically enriched allenol **63** was successfully converted into (−)‐centrolobine in high yield and enantioselectivity.^[27]^ Hence, we have realized the highly efficient stereoselective total synthesis of (−)‐centrolobine in 6 steps and an overall yield of 20 % starting from cyclopentanone **60**.

## Conclusion

In conclusion, we have established a general and efficient highly diastereoselective intramolecular *O*‐allylation of terminal and internal allenols. The presented procedure enables the construction of synthetically important *syn*‐2,4‐disubstituted and 2,4,6‐trisubstituted tetrahydropyrans. The methodology tolerates a wide range of functional groups as well as sterically high congested substrates. Furthermore, we successfully utilized the newly developed methodology in the highly stereoselective and atom economic total synthesis of (−)‐centrolobine.

## Conflict of interest

The authors declare no conflict of interest.

## Supporting information

As a service to our authors and readers, this journal provides supporting information supplied by the authors. Such materials are peer reviewed and may be re‐organized for online delivery, but are not copy‐edited or typeset. Technical support issues arising from supporting information (other than missing files) should be addressed to the authors.

Supplementary
