# Supplementary material for: Rhodium‐Catalyzed Cyclization of Terminal and Internal Allenols: An Atom Economic and Highly Stereoselective Access Towards Tetrahydropyrans
Source: Angew Chem Int Ed Engl. 2020 Oct 26;59(52):23485–90. doi: 10.1002/anie.202009166 (PMC7756761; doi:10.1002/anie.202009166)
Supplement: Supplementary file 1 — Supplementary [file ANIE-59-23485-s001.pdf]

Angewandte Chemie
Eine Zeitschrift der Gesellschaft Deutscher Chemiker

Supporting Information

Rhodium-Catalyzed Cyclization of Terminal and Internal Allenols: An Atom Economic and Highly Stereoselective Access Towards Tetrahydropyrans

*Johannes P. Schmidt and Bernhard Breit**

anie_202009166_sm_miscellaneous_information.pdf

Supporting Information

©Wiley-VCH 2016

69451 Weinheim, Germany

Abstract: A comprehensive study of a diastereoselective Rh-catalyzed cyclization of terminal and internal allenols is reported. The methodology allows an atom economic and highly *syn*-selective access to synthetically important 4,6-disubstituted, respectively 2,4,6-trisubstituted tetrahydropyrans (THP). Furthermore, it demonstrates its utility and versatility through a great functional group compatibility and the possibility to enable the stereoselective access of THP compounds.

DOI: 10.1002/anie.2016XXXXX

Table of Contents

1 General and Materials 1
2 Experimental Procedures Substart Synthesis 3
2.1 Preparation terminal allenes 3
2.1.1 General Procedure terminal allene 3
2.1.2 Synthesis and characterization of terminal allenes 5
2.2 Prepartion internal allenes 26
2.2.1 General procedure internal allenes 26
2.2.2 Synthesis and characterization of internal allenes 28
2.3 Synthesis and characterization of 1,3-substituted allenols 55
3 Experimental Procedures Catalysis 61
3.1 General Procedure catalysis 61
4 Condition screening 65
4.1 Condition screening terminal allenes 65
4.2 Condition screening internal allenes 68
5 Proposed Mechanism 72
6 Synthesis and characterization of *syn*-tetrahydropyrans 73
6.1 Large scale catalysis 99
6.2 Catalysis of 1, 3-substituded δ-allenols 100
7 Stereoselective synthesis 107
8 Catalysis followed by in situ hydration 113
9 Total synthesis of (-)-centrolobine 116
10 Determination of relative configuration 121
11 Ligand synthesis 125
12 NMR 130
13 GC-analysis 283
14 References 288

1 General and Materials

FCC (Flash Column Chromatography) was accomplished using MACHEREY-NAGEL silica gel 60 ® (230-400 mesh).

TLC (Thin Layer Chromatography) was performed on aluminum plates pre-coated with silica gel (MERCK, $60F_{254}$), which were visualized by UV fluorescence (λ_{max} = 254 nm) and/or by staining with 1% w/v $KMnO_4$ in 0.5 M aqueous K_2CO_3.

NMR (Nuclear Magnetic Resonance) spectra were acquired on a BRUKER Avance 400 spectrometer (400 MHz and 100.6 MHz for ^{1}H and ^{13}C respectively) and/or on a VARIAN Mercury (300 MHz and 75.5 MHz for ^{1}H and ^{13}C respectively). All ^{1}H NMR spectra are reported in parts per million (ppm) downfield of TMS and were measured relative to the signals at 7.26 ppm ($CHCl_3$). All ^{13}C-NMR spectra were reported in ppm relative to residual $CHCl_3$ (77.16 ppm) and were obtained with ^{1}H-decoupling. Data for ^{1}H-NMR are described as following: chemical shift (δ in ppm), multiplicity (s, singlet; d, doublet; t, triplet; q, quartet; quin, quintet; sx, sextet; m, multiplet; app, apparent; br, broad signal), coupling constant (Hz), integration. Data for ^{13}C-NMR spectra are described in terms of chemical shift (δ in ppm).

High resolution mass spectra (**HR-MS**) were obtained on a THERMO SCIENTIFIC Advantage and a THERMO SCIENTIFIC Exactive instrument (APCI/MeOH: spray voltage 4-5 kV, ion transfer tube: 250-300 °C, vaporizer: 300-400 °C).

Chiral HPLC was performed on a MERCK HITACHI HPLC apparatus (pump: L-7100, UV detector: D-7400, oven: L-7360; columns: Chiralpak AD-3, AD-H, Chiralcel OD-3, 25 cm, 4.6 mm, DAICEL; Lux A-2, C-1, C-2, C-4, 50 cm, 4.6 mm, PHENOMENEX; carrier gas: He).

GC

Chiral GC was performed on a Agilent Technologies 6890N network GC-System [Inlet: 200 °C, 1.13 bar; Column: Hydrodex-B-TBDAc 25m x 0.25mm , carrier gas He (constant flow 1 mL/min).

Optical Rotation

The optical rotation of chiral compounds was determined on an A. KRÜSS OPTRONIC P8000 T apparatus and transformed for a given temperature according to the following formula

$$[\boldsymbol{\alpha}]_{\boldsymbol{D}}^{\boldsymbol{T}} = \frac{\boldsymbol{\alpha} \cdot 100}{c \cdot d}$$

α: measured value for optical rotation; *c*: concentration in g/100 ml; *d*: length of the cuvette in dm; T: temperature in °C.

Solvents: 1,2-Dichloroethane (DCE) was freshly distilled over CaH_2 and degassed with argon prior to use. Toluene was freshly distilled over Sodium/Benzophenone and degassed with argon prior to use. Terahydrofuran (THF) was purchased in HPLC grade quality and was purified by continuous distillation over potassium under argon. Solvents employed for work-up and column chromatography were purchased in technical grade quality and distilled by rotary evaporator before use.

Ligand and Metal catalyst: The ligands were purchased from Sigma-Aldrich, ABCR, Alfa Aesar, TCI, ChemPur and used without further purification. Josiphos was either purchased from Sigma-Aldrich or received as a gift from Solvias. $[Rh(COD)Cl]_2$ and $Pd(dba)_2$ were purchased from Sigma-Aldrich

2 Experimental Procedures Substart Synthesis

2.1 Preparation terminal allenes.

2.1.1 General Procedure terminal allene

General procedure GRIGNARD synthesis (GP1):

To a suspension of magnesium (1.2 equiv.) and iodide (catalytic amount) in THF (1.0 M) corresponding bromide (15 mmol) was added dropwise. After the reaction stopped refluxing, the mixture was heated to 80 °C for 1 h. Afterwards the solution was cooled to room temperature and directly used in the substrate synthesis or was stored in the freezer.

General procedure 2: 1,4-Addition:

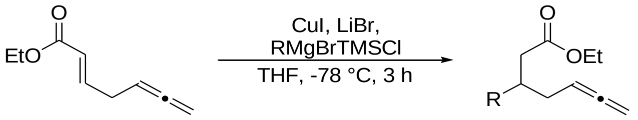

A mixture of CuI (10 mol%) and LiBr (20 mol%) was carefully dried and cooled to room temperature under vacuum and backfilled with argon using a standard SCHLENK line apparatus, before extra dry or freshly distilled THF (1.0 M) was added. The mixture was stirred at room temperature for 15 min and then cooled to −78 °C to -30 °C. A solution of ethyl (E)-hepta-2,5,6-trienoate (1.0 equiv.) in extra dry or freshly distilled THF (0.3 M) and TMSCl (1.1 equiv.) was added at this temperature. The mixture was stirred for 15 min before the GRIGNARD reagent in THF (1.5 equiv.) was added dropwise, stirred for 30 minutes at −78 °C and then slowly warmed to −30 °C. The reaction was quenched by the addition of aqueous saturated NH_4Cl-solution. The layers were separated, the aqueous layer was extracted with Et_2O (4 × 30 mL), the combined organic layer were washed with brine (40 mL) and dried over Na_2SO_4. The solvent was removed and the residue was purified by flash chromatography on silica gel using a mixture of pentane and ether.

General procedure 3: LAH-reduction:

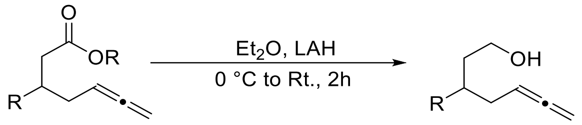

A suspension of LAH (1.5 equiv.) in dry Et_2O was cooled to 0 °C then a solution of 3-ethylhepta-5,6-dienoate in Et_2O (0.5 M) was added dropwise. The reaction was stirred at this temperature for 1 h and then warmed to room temperature and stirred for another hour. The mixture was quenched through the addition of H_2O and aqueous HCl (2.0 M). The layers were separated, the organic layer was washed with H20 (20 mL) and brine (20 mL). The aqueous layer was extracted with Et_2O (4 × 20 mL). The combined organic layer were dried over Na_2SO_4, the solvent was removed. The crude product was used without further purification.

General procedure 4:

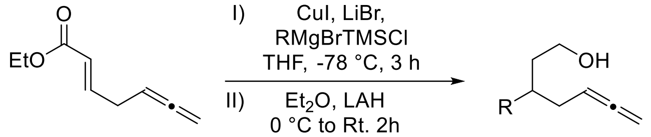

I) A mixture of CuI (10 mol%) and LiBr (20 mol%) was carefully dried and cooled to room temperature under vacuum and backfilled with argon using a standard SCHLENK line apparatus, before extra dry or freshly distilled THF (1.0 M) was added. The mixture was stirred at room temperature for 15 min and then cooled to −78 °C to -30 °C. A solution of ethyl (E)-hepta-2,5,6-trienoate (1.0 equiv.) in extra dry or freshly distilled THF (0.3 M) and TMSCl (1.1 equiv.) was added at this temperature. The mixture was stirred for 15 min before the GRIGNARD reagent in THF (1.5 equiv.) was added dropwise, stirred for 30 minutes at −78 °C and then slowly warmed to −30 °C. The reaction was quenched by the addition of aqueous saturated NH_4Cl-solution. The layers were separated, the aqueous layer was extracted with Et_2O (4 × 30 mL), the combined organic layer were washed with brine (40 mL) and dried over Na_2SO_4. The solvent was removed and the residue was filtered over a shirt silica pad

II) The crude product was dissolved in Et_2O (0.5 M) and added dropwise to a suspension of LAH (1.5 equiv.) in dry Et_2O at 0 °C. The reaction was stirred at this temperature for 1 h and then warmed to room temperature and stirred for another hour. The mixture was quenched through the addition of H_2O and aqueous HCl (2.0 M). The layers were separated, the organic layer was washed with H_20 (20 mL) and brine (20 mL). The aqueous layer was extracted with Et_2O (4 × 20 mL). The combined organic layer were dried over Na_2SO_4, the solvent was removed. The solvent was removed and the residue was purified by flash chromatography on silica gel using a mixture of pentane and ether.

2.1.2 Synthesis and characterization of terminal allenes

Synthesis of ethyl (*E*)-hepta-2,5,6-trienoate

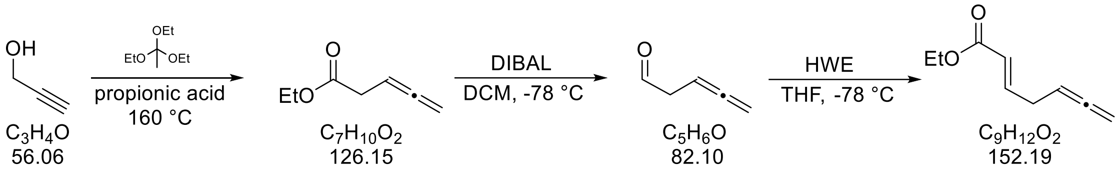

Synthesis of ethyl penta-3,4-dienoate 60

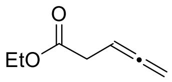

$C_7H_{10}O_2$
126.16

Prepared in analogy to a literature procedure.[1]

To a solution of propargyl alcohol (9.0 mL, 8.7 g, 0.16 mol, 0.5 equiv.) in triethyl orthoacetate (60 mL, 53 g, 0.32 mol, 1.0 equiv.) was added dropwise propionic acid (0.80 mL, 0.80 g, 11 mmol, 4.0 mol%) at 100 °C. The mixture was heated to 160 °C and resulting EtOH was continuously distilled off under atmospheric pressure for 1 h. Then another aliquot of propargyl alcohol (9.0 mL, 8.7 g, 0.16 mol, 0.5 equiv.) and propionic acid (0.80 mL, 0.80 g, 11 mmol, 4.0 mol%) were added dropwise and the mixture was stirred for another 1 h before a third portion of propionic acid (0.80 mL, 0.80 g, 11 mmol, 4.0 mol%) was added. The reaction was stirred for an additional hour and then cooled to room temperature and quenched by addition of aqueous HCl-solution (2 M, 100 mL). The organic layer was separated and the aqueous layer was extracted with Et_2O (3 × 100 mL). The combined organic layers were washed with brine (20 mL), dried over $MgSO_4$ and the solvent was removed under reduced pressure. The residue was purified by fractional distillation under reduced pressure ($bp_{46\ mbar}$: 75 °C). The title compound was obtained as colorless liquid (23 g, 0.24 mol, 76 %).

Analytical Data

^{1}H-NMR (400.1 MHz, $CDCl_3$): δ = 1.27 (t, *J* = 7.1 Hz, 3H), 3.07 (dt, *J* = 7.4 Hz, *J* = 3.0 Hz, 2H), 4.16 (q, *J* = 7.1 Hz, 2H), 4.76 (dt, *J* = 2.9 Hz, *J* = 6.7 Hz, 2H), 5.27 (m_c, 1H) ppm.

^{13}C-NMR (100.6 MHz, $CDCl_3$): δ = 14.3, 34.7, 60.9, 75.8, 83.6, 171.4, 209.5 ppm.

APCI-HRMS: *m/z* calcd for $C_7H_{10}O_2$ $[M+H]^+$ 127.0759, found 127.0759.

Synthesis of penta-3,4-dienal 61

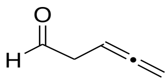

C_5H_6O
82.10

Prepared in analogy to a literature procedure.[1]

At −80°C a solution of DIBAL-H (189 mL 189 mmol, 1.4 equiv., 1.0 M in CH_2Cl_2) was added dropwise over 90 min to a solution of ethylpenta-3,4-dienoate (17.1 g, 136 mmol, 1.0 equiv.) in CH_2Cl_2 (85 mL). The reaction mixture was stirred for 1 h and then transferred to an ice cold aqueous solution of HCl (2.0 M, 500 mL) at 0 °C. The layers were separated, the organic layer was washed with HCl (2.0 M, 2 × 500 mL) and the aqueous layer was extracted with CH_2Cl_2 (2 × 300 mL). The combined organic layers were washed with brine (50 mL), dried over Na_2SO_4, filtered over a silica pad and concentrated under reduced pressure. The crude product was purified by fractional distillation ($bp_{.250\ mbar}$: 65-78 °C). The title compound **123** was obtained as colorless liquid (5.47 g, 63.5 mmol, 47 %).

Analytical Data:

^{1}H-NMR (400.1 MHz, $CDCl_3$): δ = 3.11 (dtd, *J* = 7.7, 3.0, 1.8 Hz, 2H), 4.79 (dt, *J* = 6.8, 3.0 Hz, 2H), 5.25 (m_c, 1H), 9.71 (t, *J* = 1.9 Hz, 1H) ppm.

^{13}C-NMR (100.6 MHz, $CDCl_3$): δ = 42.8, 76.0, 81.1, 199.2, 210.1 ppm.

APCI-HRMS: *m/z* calcd for C_5H_6O $[M+NH_4]^+$ 100.0762, found 100.0757.

Synthesis of ethyl (*E*)-hepta-2,5,6-trienoate 62

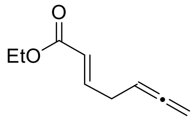

$C_9H_{12}O_2$
152.19

Prepared in analogy to a literature procedure.[2]

A suspension of NaH (60% in mineral oil, 1.1 g, 27 mmol, 1.1 equiv.) in dry THF (100 mL) was cooled to 0 °C and triethyl phosphonoacetate (6.9 g, 6.2 mL 31 mmol, 1.3 equiv.) was added dropwise to the reaction mixture. After complete addition, the suspension was stirred for 1 h at 0 °C and was then cooled to −78 °C. A solution of penta-4,5-dienal (2.0 g, 24 mmol, 1.0 equiv.) in dry THF (20 mL) was added dropwise to the reaction mixture at this temperature. The mixture was slowly warmed to −20 °C over 3 h and then quenched by the addition of aqueous saturated NH_4Cl-solution (50 mL). The layers were separated, the aqueous layer was extracted with Et_2O (3 × 100 mL) and the combined organic layers were dried over Na_2SO_4 and concentrated under reduced pressure. The residue was purified by flash chromatography on silica gel (60:1 Pentane:Et_2O). The title compound was obtained as colorless liquid (3.2 g, 21 mmol, 85 %).

Analytical Data:

^{1}H-NMR (400.1 MHz, $CDCl_3$): δ = 1.29 (t, *J* = 7.0 Hz, 3H), 2.87 – 2.93 (m, 2H), 4.19 (q, *J* = 7.0 Hz, 2H), 4.74 (dt, *J* = 6.8, 3.1 Hz, 2H), 5.12 (tt, *J* = 6.9 Hz, 1H), 5.89 (dt, *J* = 15.7, 1.7 Hz, 1H), 6.97 (dt, *J* = 15.6, 6.4 Hz, 1H) ppm.

^{13}C-NMR (100.6 MHz, $CDCl_3$): δ = 14.3, 31.2, 60.3, 75.8, 86.4, 122.2, 146.3, 166.5, 209.3 ppm.

APCI-HRMS: *m/z* calcd for $C_9H_{13}O_2$ $[M+H]^+$ 153.0910, found 153.0909.

Synthesis of ethyl 3-methylhepta-5,6-dienoate 63

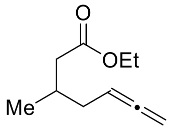

$C_{10}H_{16}O_2$
168.24

The reaction was performed according to **general procedure 3** with (*E*)-hepta-2,5,6-trienoate (1.0 g, 6.6 mmol, 1.0 equiv.) and methyl magnesium bromide in THF (3.3 mL, 9.9 mmol, 3.0 M). The desired product was obtained a colourless liquid (0.98 g, 5.8 mmol, 88 %).

Analytical Data

^{1}H-NMR (400.1 MHz, CDCl$_3$): δ = 0.98 (d, *J* = 6.4, 3H), 1.25 (t, *J* = 7.1, 3H), 1.95 – 2.16 (m, 4H), 2.30 – 2.41 (m, 1H), 4.09 – 4.17 (m, 2H), 4.65 (dt, *J* = 6.7, 2.7, 2.7, 2H), 5.00 – 5.11 (m, 1H) ppm.

^{13}C-NMR (100.6 MHz, CDCl$_3$): δ = δ = 14.4, 19.6, 30.7, 35.7, 41.2, 60.2, 74.4, 87.7, 173.1, 209.4 ppm.

APCI-HRMS: *m/z* calcd for $C_{10}H_{20}O_2N$ $[M+NH_4]^+$ 186.1489 found 186.1489

GC: Hydrodex-B-TBDAc 25m x 0.25mm, 80 °C, isothermal [50% *ee*. t_R = 29.0 min (minor), 30.0 min (major)]

Synthesis of 3-methylhepta-5,6-dien-1-ol 53rac

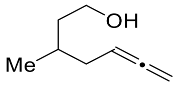

$C_8H_{14}O$
126.20

The reaction was performed according to **general procedure 4** with (E)-hepta-2,5,6-trienoate (0.8 g, 5.2 mmol, 1.0 equiv.) and methyl magnesium bromide (2.6 mL, 7.8 mmol, 3.0 M). The crude product from step I was treated with LAH (0.30 g, 7.8 mmol, 1.5 equiv.) The crude product from step II was purified by flash chromatography on silica gel (10:1 to 5:1 pentane/Et_2O). The desired product was obtained as a colorless liquid (0.63 g, 3.3 mmol, 63%).

Analytical Data

^{1}H-NMR (400.1 MHz, CDCl$_3$): δ = 0.95 (d, *J* = 6.7 Hz, 3H), 1.27 (s, 1H), 1.36 – 1.47 (m, 1H), 1.63 – 1.77 (m, 2H), 1.85 – 1.97 (m, 1H), 2.04 (ddddd, *J* = 14.1, 7.3, 5.8, 3.0, 3.0 Hz, 1H), 3.64 – 3.76 (m, 2H), 4.64 (ddd, *J* = 6.7, 2.9, 2.9 Hz, 2H), 5.06 (ddd, *J* = 7.4, 7.4, 6.6, 6.6 Hz 1H).ppm.

^{13}C-NMR (100.6 MHz, CDCl$_3$): δ = 19.6, 30.0, 36.1, 39.4, 61.2, 74.2, 88.1, 209.3 ppm.

APCI-HRMS: *m/z* calcd for C_8H15O $[M+H]^+$ 127.1117 found 127.1119.

Synthesis of 3-(buta-2,3-dien-1-yl)tetradecanol 64

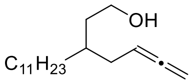

$C_{18}H_{34}O$
266.47

The reaction was performed according to **general procedure 4** with (E)-hepta-2,5,6-trienoate (0.8 g, 5.2 mmol, 1.0 equiv.) and undecyl magnesium bromide (13 mL, 13 mmol, 1.0 M). The crude product from step I was treated with LAH (0.30 g, 7.8 mmol, 1.5 equiv.) The crude product from step II was purified by flash chromatography on silica gel (10:1 to 5:1 pentane/Et_2O). The desired product was obtained as a colorless liquid (0.9 g, 3.4 mmol, 65 %).

Analytical Data

^{1}H-NMR (400.1 MHz, $CDCl_3$): δ = 0.84 – 0.92 (m, 3H), 1.26 (m, 21H), 1.50 – 1.64 (m, 3H), 1.96 – 2.11 (m, 2H), 3.68 (t, *J* = 6.8, 2H), 4.64 (dd, *J* = 6.7, 2.9, 2H), 4.94 – 5.09 (m, 1H) ppm.

^{13}C-NMR (100.6 MHz, $CDCl_3$): δ = 14.2, 22.8, 26.7, 29.4, 29.7, 29.8, 30.1, 32.0, 32.8, 33.7, 34.7, 36.8, 61.2, 74.2, 87.9, 209.3 ppm.

APCI-HRMS: *m/z* calcd for $C_{18}H_{35}O$ $[M+H]^+$ 267.2682 found 267.2682.

Synthesis of 3-neopentylhepta-5,6-dien-1-ol 65

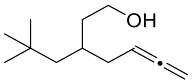

$C_{12}H_{22}O$
182.31

The reaction was performed according to **general procedure 4** with (E)-hepta-2,5,6-trienoate (0.8 g, 5.2 mmol, 1.0 equiv.) and (3,3-dimethylbutyl) magnesium bromide (7.2 mL, 7.2 mmol, 1.0 M). The crude product from step I was treated with LAH (0.30 g, 7.8 mmol, 1.5 equiv.) The crude product from step II was purified by flash chromatography on silica gel (10:1 to 5:1 pentane/Et_2O). The desired product was obtained as a colorless liquid (0.52 g, 2.9 mmol, 55 %).

Analytical Data

^{1}H-NMR (400.1 MHz, $CDCl_3$): δ = 0.90 (s, 9H), 1.13 – 1.18 (m, 1H), 1.24 – 1.29 (m, 2H), 1.50 – 1.68 (m, 3H), 1.95 – 2.13 (m, 2H), 3.62 – 3.72 (m, 2H), 4.65 (ddd, *J* = 6.7, 2.9, 2.9 Hz 2H), 5.04 (ddd, *J* = 7.5, 6.7, 6.7 Hz 1H) ppm.

^{13}C-NMR (100.6 MHz, $CDCl_3$): δ = 30.0, 31.0, 31.3 , 35.2, 39.0, 47.9, 61.2, 74.2, 88.0, 209.5 ppm.

APCI-HRMS: *m/z* calcd for $C_{12}H_{23}O$ $[M+H]^+$ 183.1743 found 183.1743.

Synthesis of ethyl 3-cyclopropylhepta-5,6-dienoate 66

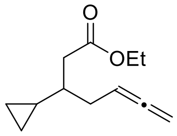

$C_{12}H_{18}O_2$
194.27

The reaction was performed according to **general procedure 2** with (E)-hepta-2,5,6-trienoate (0.8 g, 5.2 mmol, 1.0 equiv.) and cyclopropylmagnesium bromide (13 mL, 13 mmol, 1.0 M). The crude product was purified by flash chromatography on silica gel (40:1 pentane/Et_2O). The desired product was obtained as a colorless liquid (0.58 g, 3.0 mmol, 58 %).

Analytical Data

^{1}H-NMR (400.1 MHz, $CDCl_3$): δ = 0.13 – 0.16 (m, 2H), 0.39 – 0.51 (m, 2H), 0.59 – 0.68 (m, 1H), 1.18 – 1.27 (m, 4H), 2.11 (ddddd, *J* = 14.1, 8.1, 7.2, 2.5, 2.5 Hz, 1H), 2.22 (ddddd, *J*=14.2, 7.3, 5.8, 3.0, 3.0 Hz, 1H), 2.34 (dd, *J* = 14.6, 7.2 Hz, 1H), 2.44 (dd, *J* = 14.6, 6.8 Hz, 1H), 4.12 (dddd, *J* = 7.1, 7.1, 7.1, 1.5 Hz, 2H), 4.60 – 4.67 (m, 2H), 5.09 (dddd, *J* = 8.1, 6.7, 6.7, 6.7 Hz, 1H) ppm.

^{13}C-NMR (100.6 MHz, $CDCl_3$): δ = 4.0, 4.2, 14.3, 15.9, 34.2, 39.6, 41.5, 60.2, 74.2, 87.5, 173.2, 209.4 ppm.

APCI-HRMS: *m/z* calcd for $C_{12}H_{19}O_2$ $[M+H]^+$ 195.1380 found 195.1379.

Synthesis of 3-cyclopropylhepta-5,6-dienol 67

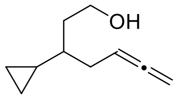

$C_{10}H_{16}O$
152.24

The reaction was performed according to **general procedure 3** with ethyl 3-(3-phenylpropyl)hepta-5,6-dienoate (0.58 g, 3.0 mmol.) and LAH (0.17 g, 4.5 mmol, 1.5 equiv.). The desired product was obtained as a colorless liquid (0.45 g, 0.29 mmol, 99%).

Analytical Data

^{1}H-NMR (400.1 MHz, $CDCl_3$): δ = 0.05 – 0.19 (m, 2H), 0.41 – 0.52 (m, 2H), 0.52 – 0.65 (m, 1H), 0.79 – 0.87 (m, 1H), 1.40 (s, 1H), 1.66 (dddd, *J* = 13.7, 7.6, 6.9, 6,9 Hz, 1H), 1.77 (dddd, *J* = 13.9, 6.9, 6.9, 6.0 Hz, 1H), 2.09 (ddddd, *J* = 14.1, 7.9, 7.0, 2.6, 2.6 Hz, 1H), 2.19 (ddddd, *J* = 14.2, 7.3, 5.4, 3.0, 3.0 Hz, 1H), 3.75 – 3.80 (m, 2H), 4.61 – 4.66 (m, 2H), 5.08 – 5.16 (m, 1H) ppm.

^{13}C-NMR (100.6 MHz, $CDCl_3$): δ = 3.8, 4.6, 16.0, 34.4, 37.7, 41.0, 61.3, 74.1, 88.0, 209.2 ppm.

APCI-HRMS: *m/z* calcd for $C_{12}H_{23}O$ $[M+H]^+$ 153.1274 found 153.1274.

Synthesis of ethyl 3-cyclohexylhepta-5,6-dienoate 68

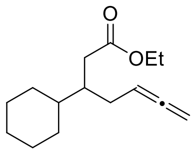

$C_{15}H_{24}O_2$
236.36

The reaction was performed according to **general procedure 2** with (E)-hepta-2,5,6-trienoate (0.8 g, 5.2 mmol, 1.0 equiv.) and cyclopropylmagnesium bromide (13 mL, 13 mmol, 1.0 M). The crude product was purified by flash chromatography on silica gel (40:1 pentane/Et_2O). The desired product was obtained as a colorless liquid (0.58 g, 3.0 mmol, 58 %).

^{1}H-NMR (400.1 MHz, CDCl3): δ = 5.05 – 4.97 (m, 1H), 4.63 (ddd, J = 6.7, 3.1, 2.4 Hz, 2H), 4.12 (q, J = 7.2 Hz, 2H), 2.32 (dd, J = 15.3, 6.2 Hz, 1H), 2.22 (dd, J = 15.4, 7.2 Hz, 1H), 2.00 – 1.92 (m, 1H), 1.92 – 1.83 (m, 1H), 1.77 – 1.58 (m, 7H), 1.25 (t, J = 7.1 Hz, 4H), 1.21 – 1.05 (m, 3H), 1.05 – 0.92 (m, 2H) ppm.
^{13}C-NMR (100.6 MHz, CDCl3): δ = 209.3, 173.9, 88.3, 74.3, 60.2, 40.6, 40.5, 36.2, 30.7, 30.0, 29.6, 26.8, 26.8, 26.8, 14.3 ppm.
HRMS (pos. APCI): [M+H+] calculated for 237.1849, found 237.1846.

Synthesis of 3-cyclohexylhepta-5,6-dien-1-ol 69

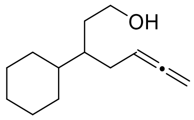

$C_{13}H_{22}O$
194.32

The reaction was performed according to **general procedure 3** with ethyl 3-(3-phenylpropyl)hepta-5,6-dienoate (0.58 g, 3.0 mmol.) and LAH (0.17 g, 4.5 mmol, 1.5 equiv.). The desired product was obtained as a colorless liquid (0.45 g, 0.29 mmol, 99%).

^{1}H-NMR (400.1 MHz, CDCl3): δ = 5.11 – 4.99 (m, 1H), 4.64 (dt, J = 6.7, 2.9 Hz, 2H), 3.74 – 3.60 (m, 2H), 2.09 (dddt, J = 14.5, 7.2, 5.5, 3.1 Hz, 1H), 1.94 (dddt, J = 14.5, 7.6, 6.8, 2.8 Hz, 1H), 1.79 – 1.70 (m, 2H), 1.69 – 1.57 (m, 4H), 1.50 (dtd, J = 13.5, 7.3, 6.1 Hz, 1H), 1.43 – 1.32 (m, 4H), 1.28 – 1.14 (m, 3H), 1.11 (dt, J = 12.6, 3.4 Hz, 1H), 1.08 – 0.96 (m, 2H) ppm.
^{13}C-NMR (100.6 MHz, CDCl3): δ = 209.1, 88.9, 74.3, 61.9, 40.5, 40.1, 34.0, 30.3, 29.9, 29.8, 26.9, 26.9, 26.9 ppm.
APCI-HRMS: *m/z* calcd for $C_{13}H_{23}O$ $[M+H]^+$ 195.1743, found 195.1743

Synthesis of ethyl 3-(3-phenylpropyl)hepta-5,6-dienoate 70

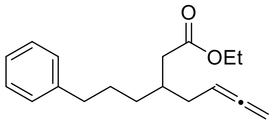

$C_{18}H_{24}O_2$
272.39

The reaction was performed according to **general procedure 2** with (E)-hepta-2,5,6-trienoate (0.8 g, 5.2 mmol, 1.0 equiv.) and (3-phenylpropyl)magnesium bromide (13 mL, 13 mmol, 1.0 M). The crude product was purified by flash chromatography on silica gel (40:1 pentane/Et_2O). The desired product was obtained as a colorless liquid (0.92 g, 3.4 mmol, 65%).

Analytical Data

^{1}H-NMR (400.1 MHz, $CDCl_3$): δ = 1.24 (t, *J* = 7.1 Hz, 3H), 1.35 – 1.47 (m, 2H), 1.60 – 1.69 (m, 2H), 1.97 – 2.12 (m, 3H), 2.21 – 2.36 (m, 2H), 2.60 (dd, *J* = 7.7 Hz, 2H), 4.12 (dddd, *J* = 7.1 Hz, 2H), 4.59 – 4.66 (m, 2H), 5.01 (ddd, *J* = 7.2, 6.6 Hz, 1H), 7.15 – 7.20 (m, 3H), 7.25 – 7.29 (m, 2H).ppm.

^{13}C-NMR (100.6 MHz, $CDCl_3$): δ = 14.3, 28.5, 32.9, 33.3, 35.3, 36.1, 38.8, 60.2, 74.4, 87.3, 125.8, 128.3, 128.4, 142.5, 173.2, 209.4 ppm.

APCI-HRMS: *m/z* calcd for $C_{18}H_{25}O_2$ $[M+H]^+$ 273.1849 found 273.1852.

Synthesis of 3-(3-phenylpropyl)hepta-5,6-dienol 71

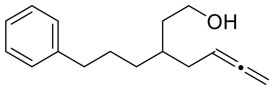

$C_{16}H_{22}O$
230.35

The reaction was performed according to **general procedure 3** with ethyl 3-(3-phenylpropyl)hepta-5,6-dienoate (0.92 g, 3.4 mmol, 1.0 equiv.) and LAH (0.19 g, 5.1 mmol, 1.5 equiv.). The desired product was obtained as a colorless liquid (0.78 g, 3.3 mmol, 98%).

Analytical Data

^{1}H-NMR (400.1 MHz, $CDCl_3$): δ =1.29 (s, 1H), 1.34 – 1.42 (m, 2H), 1.54 – 1.69 (m, 5H), 2.03 (dddd, *J* = 7.5, 5.7, 2.9, 1.0 Hz, 2H), 2.57 – 2.64 (m, 2H), 3.67 (dd, *J* = 6.8, 6.8 Hz, 2H), 4.63 (ddd, *J* = 6.7, 2.9, 2.9 Hz, 2H), 4.93 – 5.07 (m, 1H), 7.15 – 7.20 (m, 3H), 7.26 – 7.30 (m, 2H) ppm.

^{13}C-NMR (100.6 MHz, $CDCl_3$): δ = 28.6, 32.7, 33.2, 34.5, 36.3, 36.7, 61.1, 74.2, 87.7, 125.8, 128.3, 128.4, 142.6, 209.3 ppm.

APCI-HRMS: *m/z* calcd for $C_{16}H_{26}ON$ $[M+NH_4]^+$ 248.2009 found 248.2007.

Synthesis of ethyl 3-phenylhepta-5,6-dienoate 72

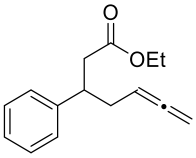

$C_{15}H_{18}O_2$
230.31

The reaction was performed according to **general procedure 2** with (E)-hepta-2,5,6-trienoate (0.8 g, 5.2 mmol, 1.0 equiv.) and phenylmagnesium bromide (13 mL, 13 mmol, 1.0 M). The crude product was purified by flash chromatography on silica gel (40:1 pentane/Et_2O). The desired product was obtained as a colorless liquid (0.66 g, 2.9 mmol, 55%).

Analytical Data

^{1}H-NMR (400.1 MHz, $CDCl_3$): δ = 1.14 (t, *J* = 7.1 Hz, 3H), 2.36 (ddt, *J* = 7.3, 7.1, 2.8 Hz, 2H), 2.58 (dd, *J* = 15.3, 8.4 Hz, 1H), 2.73 (dd, *J* = 15.3, 6.7 Hz, 1H), 3.19 – 3.31 (m, 1H), 4.03 (q, *J* = 7.2 Hz, 2H), 4.60 (dt, *J*=6.7, 4.0 Hz 2H), 4.95 (ddt, *J* = 7.1, 4.0, 2.8 Hz, 1H), 7.16 – 7.24 (m, 3H), 7.26 – 7.33 (m, 2H) ppm.

^{13}C-NMR (100.6 MHz, $CDCl_3$): δ = 14.2, 35.5, 40.7, 42.2, 60.3, 74.7, 87.5, 126.7, 127.6, 128.5, 143.5 172.3, 209.4 ppm.

APCI-HRMS: *m/z* calcd for $C_{15}H_{22}$O2N $[M+NH_4]^+$ 248.1645 found 248,1647

Synthesis of 3-phenylhepta-5,6-dien-1-ol 73

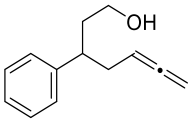

$C_{13}H_{16}O$
188.27

The reaction was performed according to **general procedure 3** with ethyl 3-phenylhepta-5,6-dienoate (0.66 g, 2.9 mmol, 1.0 equiv.) and LAH (0.17 g, 4.4 mmol, 1.5 equiv.,). The desired product was obtained as a colorless liquid (0.54 g, 2.9 mmol, 99%).

Analytical Data

^{1}H-NMR (400.1 MHz, $CDCl_3$): δ = 1.37 (s, 1H), 1.83 (dddd, *J* = 13.7, 10.1, 6.4, 5.4 Hz, 1H), 2.04 (dddd, *J* = 13.7, 7.6, 7.0, 4.8 Hz, 1H), 2.30 – 2.40 (m, 2H), 2.84 (dtd, *J* = 10.0, 7.3, 4.8 Hz, 1H), 3.42 – 3.59 (m, 2H), 4.54 – 4.64 (m, 2H), 4.95 (ddd, *J* = 14.0, 7.3, 6.7 Hz, 1H), 7.16 – 7.24 (m, 3H), 7.27 – 7.33 (m, 2H) ppm.

^{13}C-NMR (100.6 MHz, $CDCl_3$): δ = 29.8, 36.0, 38.7, 42.6, 61.1, 74.5, 88.0, 126.5, 127.8, 128.5, 144.4, 209.2 ppm.

APCI-HRMS: *m/z* calcd for $C_{13}H_{20}$ON $[M+NH_4]^+$ 206.1541 found 206.1541

Synthesis of ethyl 3-(naphthalene-2-yl)hepta-5,6-dienoate 74

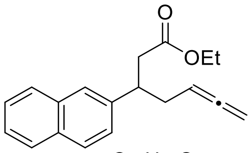

$C_{19}H_{20}O_2$
280.37

The reaction was performed according to **general procedure 2** with (E)-hepta-2,5,6-trienoate (0.8 g, 5.2 mmol, 1.0 equiv.) and naphthalene-2-ylmagnesium bromide (13 mL, 13 mmol, 1.0 M). The crude product was purified by flash chromatography on silica gel (40:1 pentane/Et_2O). The desired product was obtained as a colorless liquid (0.76 g, 2.7 mmol, 52%).

Analytical Data

^{1}H-NMR (400.1 MHz, $CDCl_3$): δ = 1.12 (t, *J* = 7.1 Hz, 3H), 2.40 – 2.54 (m, 2H), 2.70 (dd, *J* = 15.4, 8.5 Hz, 1H), 2.82 (dd, *J* = 15.4, 6.6 Hz, 1H), 3.43 (ddd, *J* = 8.5, 7.1,7.1 Hz , 1H), 4.02 (ddd, *J* = 7.1, 7.1, 3.0 Hz, 2H), 4.54 – 4.66 (m, 2H), 4.97 (ddd, *J* = 7.5, 6.8, 6.8 Hz 1H), 7.36 (m, 1H), 7.42 – 7.47 (m, 2H), 7.65 (m, 1H), 7.78 – 7.82 (m, 3H) ppm.

^{13}C-NMR (100.6 MHz, $CDCl_3$): δ = 14.2, 35.4, 40.7, 42.3, 60.4, 74.8, 87.5, 125.5, 125.9, 126.0, 126.2, 127.7, 127.8, 128.2, 132.5, 133.6, 141.0, 172.2, 209.4 ppm.

APCI-HRMS: *m/z* calcd for $C_{19}H_{24}O_2N$ $[M+NH_4]^+$ 298.1802 found 298.1798.

Synthesis of ethyl 3-([1,1'-biphenyl]-4-yl)hepta-5,6-dienoate 75

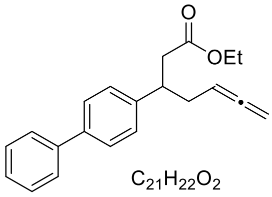

The reaction was performed according to **general procedure 2** with (E)-hepta-2,5,6-trienoate (0.8 g, 5.2 mmol, 1.0 equiv.) and [1,1'-biphenyl]-4-ylmagnesium bromide (13 mL, 13 mmol, 1.0 M). The crude product was purified by flash chromatography on silica gel (40:1 pentane/Et_2O). The desired product was obtained as a colorless liquid (0.75 g, 2.4 mmol, 47%).

^{1}H-NMR (400.1 MHz, CDCl3): 2.44 – 2.35 (m, 2H), 1.16 (t, *J* = 7.1 Hz, 3H), 2.63 (dd, *J* = 15.3, 8.5 Hz, 1H), 2.77 (dd, = 15.3, 6.6 Hz, 1H), 3.30 (dq, *J* = 8.5, 7.1 Hz, 1H) 4.06 (qd, *J* = 7.2, 0.7Hz, 2H), 4.63 (ddt, J = 6.3, 3.6, 2.7 Hz, 2H), 5.03 – 4.95 (m, 1H), 7.30 – 7.28 (m, 1H), 7.28– 7.26 (m, 1H), 7.36 – 7.30 (m, 1H), 7.46 – 7.41 (m, 2H), 7.53 – 7.52 (m, 1H), 7.55 (d, J = 4.1 Hz,1H) 7.59 – 7.57 (m, 1H), 7.61 – 7.59 (m, 1H)ppm.
^{13}C-NMR (100.6 MHz, CDCl3): δ = 14.2, 35.5, 40.7, 41.8, 60.4, 74.8, 87.5, 127.1, 127.2, 127.2, 128.0, 128.8, 142.6, 172.3, 209.4 ppm.
ESI-HRMS: *m/z* calcd for $C_{21}H_{22}O_2Na$ $[M+Na]^+$ 329.1512 found 329.1508.

Synthesis of 3-([1,1'-biphenyl]-4-yl)hepta-5,6-dien-1-ol 76

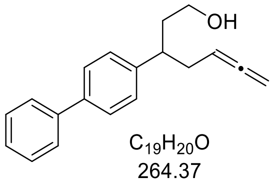

The reaction was performed according to **general procedure 3** with ethyl 3-([1,1'-biphenyl]-4-yl)hepta-5,6-dienoate (0,75 g, 2.4 mmol, 1.0 equiv.) and LAH (0.14 g, 3.6 mmol, 1.5 equiv.). The desired product was obtained as a colorless liquid (0.62 g, 2,4 mmol, 98%).

^{1}H-NMR (400.1 MHz, CDCl3): 1.36 (s, 1H), 1.87 (dddd, *J* = 13.7, 10.0, 6.4, 5.4 Hz, 1H), 2.07 (dddd, *J* = 13.8, 7.6, 7.0, 4.8 Hz, 1H), 2.38 (tt, *J* = 7.3, 2.8 Hz, 2H), 2.90 (dtd, *J* = 10.0, 7.3, 4.8 Hz, 1H), 3.48 – 3.70 (m, 2H), 4.52 – 4.67 (m, 2H), 4.91 – 5.07 (m, 1H), 7.23 – 7.26 (m, 1H), 7.26 – 7.29 (m, 1H), 7.30 – 7.38 (m, 1H), 7.40 – 7.51 (m, 2H), 7.51 – 7.66 (m, 4H) ppm.
^{13}C-NMR (100.6 MHz, CDCl3): δ = 36.0, 38.7, 42.2, 61.1, 74.6, 88.0, 127.0, 127.2, 127.2, 128.2, 128.8, 139.4, 141.0, 143.5, 209.2 ppm.
APCI-HRMS: *m/z* calcd for $C_{19}H_{24}ON$ $[M+NH_4]^+$ 282.1852 found 282.1855.

Synthesis of ethyl 3-(*p*-tolyl)hepta-5,6-dienoate 77

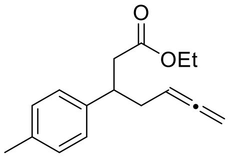

$C_{16}H_{20}O_2$
244.33

The reaction was performed according to **general procedure 2** with (E)-hepta-2,5,6-trienoate (0.8 g, 5.2 mmol, 1.0 equiv.) and *p*-tolylmagnesium bromide (13 mL, 13 mmol, 1.0 M). The crude product was purified by flash chromatography on silica gel (40:1 pentane/Et_2O). The desired product was obtained as a colorless liquid (0,76 g, 3.1 mmol, 60%).

Analytical Data

^{1}H-NMR (400.1 MHz, $CDCl_3$): δ = 1.15 (t, *J* = 7.1 Hz, 3H), 2.31 (s, 3H), 2.32 – 2.37 (m, 2H), 2.56 (dd, *J* = 15.3, 8.4 Hz, 1H), 2.71 (dd, *J* = 15.3, 6.7 Hz, 1H), 3.20 (dd, *J* = 8.4, 6.9 Hz, 1H), 4.04 (ddd, *J* = 7.2, 1.4, 1,4 Hz, 2H), 4.61 (m, 2H), 4.85 – 5.02 (m, 1H), 7.09 (d, *J* = 0.7 Hz, 4H) ppm.

^{13}C-NMR (100.6 MHz, $CDCl_3$): δ = = 14.2, 21.1, 35.5, 40.8, 41.8, 60.3, 74.6, 87.6, 127.4, 129.2, 136.1, 140.5, 172.4, 209.3 ppm.

APCI-HRMS: *m/z* calcd for $C_{16}H_{24}O_2N$ $[M+NH_4]^+$ 262.1802 found 262.1801.

Synthesis of 3-(*p*-tolyl)hepta-5,6-dien-1-ol 78

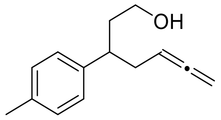

$C_{14}H_{18}O$
202.30

The reaction was performed according to **general procedure 3** with ethyl 3-(*p*-tolyl)hepta-5,6-dienoate (0,76 g, 3.1 mmol, 1.0 equiv.) and LAH (0.17 g, 4.7 mmol, 1.5 equiv.). The desired product was obtained as a colorless liquid (0.61 g, 3.0 mmol, 98%).

Analytical Data

^{1}H-NMR (400.1 MHz, $CDCl_3$): δ = 1.32 (s, 1H), 1.80 (dddd, *J* = 13.7, 10.1, 6.4, 5.4 Hz, 1H), 2.02 (dddd, *J* = 14.0, 7.5, 7.0, 4.8 Hz, 1H), 2.30 – 2.35 (m, 5H), 2.79 (dtd, *J* = 10.0, 7.3, 4.7 Hz, 1H), 3.45 – 3.61 (m, 2H), 4.50 – 4.74 (m, 2H), 4.95 (ddd, *J* = 14.0, 7.3, 6.7, 1H), 7.05 – 7.14 (m, 4H). ppm.

^{13}C-NMR (100.6 MHz, $CDCl_3$): δ = 21.1, 36.1, 38.7, 42.2, 61.2, 74.5, 88.1, 127.6, 129.2, 135.9, 141.3, 209.2 ppm.

APCI-HRMS: *m/z* calcd for $C_{14}H_{21}O_2$ $[M+HO_3]^+$ 221.1536 found 221.1531.

Synthesis of ethyl 3-(*m*-tolyl)hepta-5,6-dienoate 79

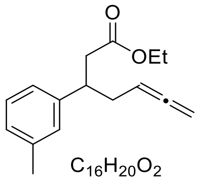

$C_{16}H_{20}O_2$
244.33

The reaction was performed according to **general procedure 2** with (E)-hepta-2,5,6-trienoate (0.8 g, 5.2 mmol, 1.0 equiv.) and *m*-tolylmagnesium bromide (13 mL, 13 mmol, 1.0 M). The crude product was purified by flash chromatography on silica gel (40:1 pentane/Et_2O). The desired product was obtained as a colorless liquid (0.67 g, 2.8 mmol, 54%).

Analytical Data

^{1}H-NMR (400.1 MHz, $CDCl_3$): δ = 1.15 (t, *J* = 7.1 Hz, 3H), 2.32 – 2.37 (m, 5H), 2.57 (dd, *J* = 15.3, 8.3 Hz, 1H), 2.71 (dd, *J* = 15.3, 6.7 Hz, 1H), 3.20 (dq, *J* = 8.4, 7.1 Hz, 1H), 4.04 (m, 2H), 4.53 – 4.68 (m, 2H), 4.95 (m, 1H), 6.96 – 7.06 (m, 3H), 7.13 – 7.25 (m, 1H) ppm.

^{13}C-NMR (100.6 MHz, $CDCl_3$): δ = 14.2, 21.5, 35.5, 40.7, 42.1, 60.3, 74.6, 87.6, 124.5, 127.4, 128.3, 128.4, 137.9, 143.5, 172.4, 209.3 ppm.

APCI-HRMS: *m/z* calcd for $C_{16}H_{24}O_2N$ $[M+NH_4]^+$ 262. 1802found 262.1799.

Synthesis of 3-(*m*-tolyl)hepta-5,6-dien-1-ol 80

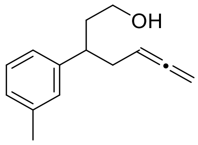

$C_{14}H_{18}O$
202.30

The reaction was performed according to **general procedure 3** with ethyl 3-(*m*-tolyl)hepta-5,6-dienoate (0.67 g, 2.8 mmol, 1.0 equiv.) and LAH (0.16 g,4.2 mmol, 1.5 equiv.). The desired product was obtained as a colorless liquid (0.55 g, 2.7 mmol, 98%).

Analytical Data

^{1}H-NMR (400.1 MHz, $CDCl_3$): δ = 1.31 (s, 1H), 1.82 (dddd, *J* = 13.7, 10.1, 6.4, 5.4 Hz, 1H), 2.02 (dddd, *J* = 13.7, 7.4, 6.9, 4.8 Hz, 1H), 2.28 – 2.38 (m, 5H), 2.79 (ddd, *J* = 10.0, 7.3, 4.8 Hz, 1H), 3.41 – 3.60 (m, 2H), 4.55 – 4.72 (m, 2H), 4.91 – 5.03 (m, 1H), 6.93 – 7.07 (m, 3H), 7.19 (m, 1H) ppm.

^{13}C-NMR (100.6 MHz, $CDCl_3$): δ = 21.6, 36.0, 38.7, 42.5, 61.2, 74.5, 88.1, 124.7, 127.2, 128.4, 128.5, 138.1, 144.3, 209.2 ppm.

APCI-HRMS: *m/z* calcd for $C_{14}H_{22}ON$ $[M+NH_4]^+$ 220.1696 found 220.1691

Synthesis of 3-(*o*-tolyl)hepta-5,6-dienoate 81

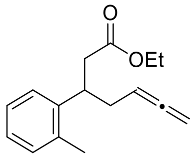

$C_{16}H_{20}O_2$
244.33

The reaction was performed according to **general procedure 2** with (E)-hepta-2,5,6-trienoate (0.8 g, 5.2 mmol, 1.0 equiv.) and *o*-tolylmagnesium bromide (13 mL, 13 mmol, 1.0 M). The crude product was purified by flash chromatography on silica gel (40:1 pentane/Et_2O). The desired product was obtained as a colorless liquid (0.74 g, 3.0 mmol, 58%).

Analytical Data

^{1}H-NMR (400.1 MHz, $CDCl_3$): δ = 1.13 (t, *J* = 7.1 Hz, 3H), 2.32 (ddd, *J* = 9.7, 7.7, 2.8 Hz, 2H), 2.38 (s, 3H), 2.60 (dd, *J* = 15.4, 8.2 Hz, 1H), 2.72 (dd, *J* = 15.4, 6.9 Hz, 1H), 3.55 (ddd, *J* = 8.2, 7.1, 7.1 Hz 1H), 4.03 (m, 2H), 4.60 (ddd, *J* = 6.6, 2.7, 1.3 Hz, 2H), 4.94 (m, 1H), 7.06 – 7.17 (m, 4H) ppm.

^{13}C-NMR (100.6 MHz, $CDCl_3$): δ = 14.2, 19.8, 35.2, 36.9, 40.2, 60.3, 74.6, 87.5, 125.7, 126.2, 126.3, 130.5, 136.1, 141.7, 172.4, 209.3 ppm.

APCI-HRMS: *m/z* calcd for $C_{16}H_{24}O_2N$ $[M+NH_4]^+$ 262.1802 found 262.1801

Synthesis of 3-(*o*-tolyl)hepta-5,6-dien-1-ol 82

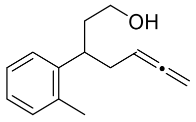

$C_{14}H_{18}O$
202.30

The reaction was performed according to **general procedure 3** with ethyl 3-(*o*-tolyl)hepta-5,6-dienoate (0.74 g, 3.0 mmol, 1.0 equiv.) and LAH (0.17 g, 4.5 mmol, 1.5 equiv.). The desired product was obtained as a colorless liquid (0.59 g, 2.9 mmol, 97%).

Analytical Data

^{1}H-NMR (400.1 MHz, $CDCl_3$): δ = 1.22 – 1.30 (m, 1H), 1.87 (dddd, *J* = 13.7, 9.6, 6.5, 5.5 Hz, 1H), 2.04 (dddd, *J* = 13.8, 7.4, 6.9, 5.1 Hz, 1H), 2.28 – 2.34 (m, 2H), 2.34 (s, 3H), 3.19 (ddd, *J* = 9.6, 7.2, 5.1 Hz, 1H), 3.40 – 3.64 (m, 2H), 4.52 – 4.65 (m, 2H), 4.95 (ddd, *J* = 14.0, 7.4, 6.7 Hz, 1H), 7.04 – 7.19 (m, 4H) ppm.

^{13}C-NMR (100.6 MHz, $CDCl_3$): δ = 20.0, 35.7, 36.9, 38.5, 61.1, 74.4, 88.0, 125.9, 126.0, 126.3, 130.4, 136.3, 142.6, 209.1 ppm.

APCI-HRMS: *m/z* calcd for $C_{14}H_{22}ON$ $[M+NH_4]^+$ 220.1696 found 220.1691.

Synthesis of ethyl 3-mesitylhepta-5,6-dienoate 83

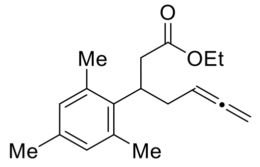

$C_{18}H_{24}O_2$
272.39

The reaction was performed according to **general procedure 2** with (E)-hepta-2,5,6-trienoate (0.8 g, 5.2 mmol, 1.0 equiv.) and mesitylmagnesium bromide (13 mL, 13 mmol, 1.0 M). The crude product was purified by flash chromatography on silica gel (40:1 pentane/Et_2O). The desired product was obtained as a colorless liquid (0.72 g, 2.7 mmol, 51%).

Analytical Data

^{1}H-NMR (400.1 MHz, $CDCl_3$): δ = 1.14 (t, *J* = 7.1 Hz, 3H), 2.22 (s, 3H), 2.36 (s, 6H), 2.42 – 2.49 (m, 2H), 2.67 – 2.87 (m, 2H), 3.79 (m, 1H), 4.03 (m, 2H), 4.53 – 4.67 (m, 2H), 4.80 – 5.07 (m, 1H), 6.79 (m, 2H) ppm.

^{13}C-NMR (100.6 MHz, $CDCl_3$): δ = 14.2, 20.7, 21.5, 21.7, 33.1, 37.1, 38.8, 60.3, 74.6, 88.2, 129.3, 131.1, 135.6, 136.4, 172.9, 209.0 ppm.

APCI-HRMS: *m/z* calcd for $C_{18}H_{28}O_2N$ $[M+NH_4]^+$ 290.2115 found 290.2118.

Synthesis of 3-mesitylhepta-5,6-dien-1-ol 84

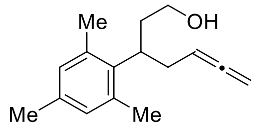

$C_{16}H_{22}O$
230.35

The reaction was performed according to **general procedure 3** with ethyl 3-mesitylhepta-5,6-dienoate (0.72 g, 2.7 mmol, 1.0 equiv.) and LAH (0.15 g, 4.1 mmol, 1.5 equiv.). The desired product was obtained as a colorless liquid (0.60 g, 2.6 mmol, 97%).

Analytical Data

^{1}H-NMR (400.1 MHz, $CDCl_3$): δ = 1.19 – 1.32 (m, 1H), 1.98 – 2.12 (m, 2H), 2.24 (s, 3H), 2.32 (s, 3H), 2.36 (s, 3H), 2.42 – 2.50 (m, 2H), 3.38 (ddd, *J* = 9.0, 7.9, 6.1 Hz, 1H), 3.47 – 3.63 (m, 2H), 4.53 – 4.70 (m, 2H), 4.97 (ddd, *J* = 7.8, 6.8, 6.8 Hz 1H), 6.69 – 6.88 (m, 2H) ppm.

^{13}C-NMR (100.6 MHz, $CDCl_3$): δ = 20.7, 21.5, 21.9, 33.5, 36.7, 37.4, 62.0, 74.6, 88.8, 129.3, 131.3, 135.4, 136.2, 137.1, 137.2, 208.8 ppm.

APCI-HRMS: *m/z* calcd for $C_{16}H_{26}ON$ $[M+NH_4]^+$ 248.2009 found 248.2009.

Synthesis of ethyl 3-(4-vinylphenyl)hepta-5,6-dienoate 85.

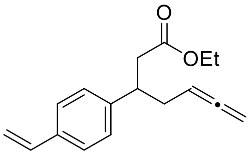

$C_{17}H_{20}O_2$
256.35

The reaction was performed according to **general procedure 2** with (E)-hepta-2,5,6-trienoate (0.8 g, 5.2 mmol, 1.0 equiv.) and (4-vinylphenyl)magnesium bromide (13 mL, 13 mmol, 1.0 M). The crude product was purified by flash chromatography on silica gel (40:1 pentane/Et_2O). The desired product was obtained as a colorless liquid (0.83 g, 3.2 mmol, 62%).

Analytical Data

^{1}H-NMR (400.1 MHz, $CDCl_3$): δ = 1.15 (t, *J* = 7.1 Hz, 3H), 2.35 (dd, *J* = 7.3, 2.8 Hz, 2H), 2.57 (dd, *J* = 15.3, 8.5 Hz, 1H), 2.72 (dd, *J* = 15.4, 6.6 Hz, 1H), 3.24 (m, 1H), 4.04 (ddd, *J* = 7.1, 1.5, 1.5 Hz, 2H), 4.54 – 4.66 (m, 2H), 4.94 (ddd, *J* = 7.4, 6.6, 6.6 Hz , 1H), 5.20 (dd, *J* = 10.9, 1.0 Hz, 1H), 5.71 (dd, *J* = 17.6, 1.0 Hz, 1H), 6.69 (ddd, *J* = 17.6, 10.9, 0.4, 1H), 7.12 – 7.21 (m, 2H), 7.32 – 7.37 (m, 2H) ppm.

^{13}C-NMR (100.6 MHz, $CDCl_3$): δ = 14.2, 35.4, 40.6, 41.9, 60.4, 74.8, 87.5, 113.4, 126.3, 127.8, 136.1, 136.7, 143.2, 172.2, 209.4 ppm.

APCI-HRMS: *m/z* calcd for $C_{17}H_{20}O_2Na$ $[M+Na]^+$ 279.1356 found 279.1359.

Synthesis of 3-(-4-vinylphenyl)hepta-5.6-dien-1-ol 86.

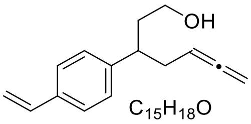

$C_{15}H_{18}O$
214.31

The reaction was performed according to **general procedure 3** with ethyl 3-(4-vinylphenyl)hepta-5,6-dienoate (0.83 g, 3.2 mmol, 1.0 equiv.) and LAH (0.18 g, 4.8 mmol, 1.5 equiv.). The desired product was obtained as a colorless liquid (0.67 g, 3.1 mmol, 97%).

Analytical Data

^{1}H-NMR (400.1 MHz, $CDCl_3$): δ = 1.34 (s, 1H), 1.82 (dddd, *J* = 13.7, 10.0, 6.3, 5.3 Hz, 1H), 2.03 (dddd, *J* = 13.7, 7.6, 6.9, 4.8 Hz, 1H), 2.25 – 2.39 (m, 2H), 2.84 (ddd, *J* = 9.9, 7.3, 4.7 Hz, 1H), 3.42 – 3.62 (m, 2H), 4.53 – 4.66 (m, 2H), 4.95 (ddd, *J* = 14.0, 7.3, 6.7 Hz, 1H), 5.21 (dd, *J* = 10.9, 1.0 Hz, 1H), 5.72 (dd, *J* = 17.6, 1.0 Hz, 1H), 6.64 – 6.76 (m, 1H), 7.11 – 7.17 (m, 2H), 7.30 – 7.39 (m, 2H) ppm.

^{13}C-NMR (100.6 MHz, $CDCl_3$): δ = 35.9, 38.6, 42.3, 61.1, 74.6, 88.0, 113.3, 126.4, 127.9, 135.9, 136.7, 144.1, 209.2 ppm.

ESI-HRMS: *m/z* calcd for $C_{17}H_{18}ONa$ $[M+Na]^+$ 237.2972 found 237.2968.

Synthesis of ethyl 3-(4-trifluoromethyl)phenyl)hepta-5,6-dienoate 87.

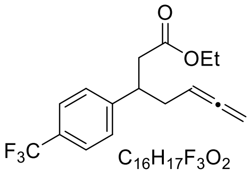

$C_{16}H_{17}F_3O_2$
298.31

The reaction was performed according to **general procedure 2** with (E)-hepta-2,5,6-trienoate (0.8 g, 5.2 mmol, 1.0 equiv.) and (4-trifluoromethyl)phenyl)magnesium bromide (13 mL, 13 mmol, 1.0 M). The crude product was purified by flash chromatography on silica gel (40:1 pentane/Et_2O). The desired product was obtained as a colorless liquid (0.70 g, 2.3 mmol, 45%).

Analytical Data

^{1}H-NMR (400.1 MHz, CDCl$_3$ δ = 1.14 (t, *J* = 7.1 Hz, 3H), 2.29 – 2.42 (m, 2H), 2.59 (dd, *J* = 15.5, 8.7 Hz, 1H), 2.75 (dd, *J* = 15.5, 6.3 Hz, 1H), 3.31 (ddd, *J* = 8.9, 7.0, 7.0 Hz, 1H), 3.99 – 4.06 (m, 2H), 4.52 – 4.66 (m, 2H), 4.85 – 4.98 (m, 1H), 7.32 (dddd, *J* = 8.0, 1.4, 1.4, 0.8 Hz, 2H), 7.49 – 7.60 (m, 2H) ppm.

^{13}C-NMR (100.6 MHz, CDCl$_3$): δ = 14.2, 35.2, 40.4, 42.0, 60.5, 75.1, 87.1, 125.4, 125.4, 125.7, 128.0, 147.6, 171.8, 209.4 ppm.

APCI-HRMS: *m/z* calcd for $C_{16}H_{21}O_2NF_3$ $[M+NH_4]^+$ 316.1519 found 316.1518.

Synthesis of 3-(4-trifluoromethyl)phenyl)hepta-5,6-dienol 88.

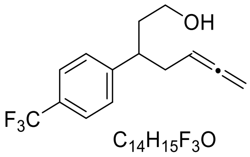

$C_{14}H_{15}F_3O$
256.27

The reaction was performed according to **general procedure 3** with Ethyl 3-(4-trifluoromethyl)phenyl)hepta-5,6-dienoate (0.70 g, 2.3 mmol, 1.0 equiv.) and LAH (0.13 mg, 3.5 mmol, 1.5 equiv.). The desired product was obtained as a colorless liquid (0.55 g, 2.2 mmol, 95%).

Analytical Data

^{1}H-NMR (400.1 MHz, CDCl$_3$): δ = 1.37 (s, 1H), 1.83 (dddd, *J* = 13.8, 9.9, 6.3, 5.2 Hz, 1H), 2.06 (dddd, *J* = 13.9, 7.8, 6.9, 5.0 Hz, 1H), 2.27 – 2.45 (m, 2H), 2.95 (dddd, *J* = 9.8, 8.0, 6.4, 4.9 Hz, 1H), 3.46 (ddd, *J* = 10.6, 7.8, 6.3 Hz, 1H), 3.57 (dddd, *J* = 10.6, 6.9, 5.3, 0.3 Hz, 1H), 4.49 – 4.65 (m, 2H), 4.86 – 4.99 (m, 1H), 7.30 (m, 2H), 7.52 – 7.61 (m, 2H).ppm.

^{13}C-NMR (100.6 MHz, CDCl$_3$): δ = 35.6, 38.5, 42.2, 60.7, 74.9, 87.6, 125.4, 125.5, 128.2, 129.0, 148.6, 209.3 ppm.

APCI-HRMS: *m/z* calcd for $C_{14}H_{19}ONF_3$ $[M+NH_4]^+$ 274.1413 found 274.1412

Synthesis of ethyl 3-(4-bromophenyl)hepta-5,6-dienoate 89.

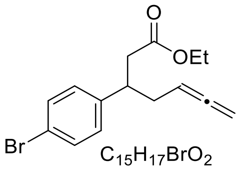

$C_{15}H_{17}BrO_2$
309.20

The reaction was performed according to **general procedure 2** with (E)-hepta-2,5,6-trienoate (0.8 g, 5.2 mmol, 1.0 equiv.) and (4-bromophenyl)magnesium bromide (13 mL, 13 mmol, 1.0 M). The crude product was purified by flash chromatography on silica gel (40:1 pentane/Et_2O). The desired product was obtained as a colorless liquid (0.69 g, 2.2 mmol, 43%).

Analytical Data

^{1}H-NMR (400.1 MHz, $CDCl_3$ δ = 1.19 (t, *J* = 7.0 Hz 3H), 2.30 – 2.45 (m, 2H), 2.56 (ddd, *J* = 17.4, 15.3, 8.5 Hz, 1H), 2.72 (ddd, *J* = 15.4, 7.3, 6.5 Hz, 1H), 3.16 – 3.25 (m, 1H), 4.03 (ddd, *J* = 7.1, 7.1 1.4 Hz, 2H), 4.52 – 4.65 (m, 2H), 4.93 (dddd, *J* = 14.0, 8.7, 7.3, 6.6 Hz, 1H), 7.03 – 7.11 (m, 1H), 7.38 – 7.46 (m, 1H)ppm.

^{13}C-NMR (100.6 MHz, $CDCl_3$): δ = 14.2, 35.3, 40.5, 41.6, 60.5, 74.9, 87.2, 127.6, 128.5, 129.4, 131.6, 142.5, 172.0, 209.4 ppm.

APCI-HRMS: *m/z* calcd for $C_{15}H_{18}O_2Br$ $[M+H]^+$ 309.0485 found 309.0486.

Synthesis of 3-(4-bromophenyl)hepta-5,6-dienol 90.

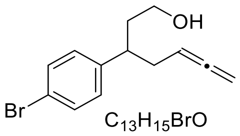

$C_{13}H_{15}BrO$
267.17

The reaction was performed according to **general procedure 3** with Ethyl 3-(4-bromophenyl)hepta-5,6-dienoate (0.69 g, 2.2 mmol, 1.0 equiv.) and LAH (0.13 mg, 3.3 mmol, 1.5 equiv.). The desired product was obtained as a colorless liquid (0.58 g, 2.2 mmol, 98%).

Analytical Data

^{1}H-NMR (400.1 MHz, $CDCl_3$): δ = 1.30 (s, 1H), 1.72 – 1.91 (m, 1H), 1.95 – 2.09 (m, 1H), 2.24 – 2.38 (m, 2H), 2.70 – 2.89 (m, 1H), 3.41 – 3.50 (m, 1H), 3.50 – 3.61 (m, 1H), 4.49 – 4.63 (m, 2H), 4.80 – 5.08 (m, 1H), 7.05 – 7.07 (m, 1H), 7.17 – 7.32 (m, 2H), 7.41 – 7.44 (m, 1H).ppm.

^{13}C-NMR (100.6 MHz, $CDCl_3$): δ = 35.8, 38.6, 41.9, 60.8, 74.7, 87.7, 120.1, 127.8, 128.5, 129.6, 131.6, 143.4, 209.2 ppm.

APCI-HRMS: *m/z* calcd for $C_{13}H_{15}O^{81}BrCl$ $[M+Cl]^-$ 302.9980 found 302.9980.

Synthesis of ethyl 3-(methoxyphenyl)hepta-5,6-dienoate 91.

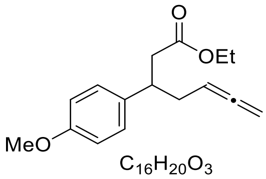

$C_{16}H_{20}O_3$
260.33

The reaction was performed according to **general procedure 2** with (E)-hepta-2,5,6-trienoate (0.8 g, 5.2 mmol, 1.0 equiv.) and 4-bromoanisole (13 mL, 13 mmol, 1.0 M). The crude product was purified by flash chromatography on silica gel (40:1 pentane/Et_2O). The desired product was obtained as a colorless liquid (0.88 g, 3.4 mmol, 65%).

Analytical Data

^{1}H-NMR (400.1 MHz, $CDCl_3$): δ = 1.15 (t, *J* = 7.1, 3H), 2.32 (dd, *J* = 7.3, 2.8 Hz 2H), 2.54 (dd, *J* = 15.2, 8.5 Hz, 1H), 2.70 (dd, *J* = 15.2, 6.6 Hz, 1H), 3.19 (ddd, *J*=8.6, 7.1, 7.1 Hz, 1H), 3.78 (s, 3H), 4.53 – 4.68 (m, 2H), 4.94 (ddd, *J*=7.2, 6.6, 6.6 Hz 1H), 6.81 – 6.86 (m, 2H), 7.08 – 7.14 (m, 2H) ppm.

^{13}C-NMR (100.6 MHz, $CDCl_3$): δ = 14.2, 35.6, 40.9, 41.4, 55.3, 60.3, 74.6, 87.6, 113.9, 128.5, 135.6, 158.3, 172.4, 209.3 ppm.

ESI-HRMS: *m/z* calcd for $C_{16}H_{20}O_3Na$ $[M+Na]^+$ 283.1305 found 283.1302.

Synthesis of 3-(4-methoxyphenyl)hepta-5,6-dienol 92.

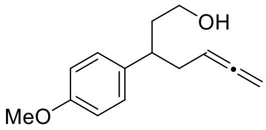

$C_{14}H_{18}O_2$
218.30

The reaction was performed according to **general procedure 3** with ethyl 3-(methoxyphenyl)hepta-5,6-dienoate (0.88 g, 3.4 mmol, 1.0 equiv.) and LAH (0.19 mg, 5.1 mmol, 1.5 equiv.). The desired product was obtained as a colorless liquid (0.73 g, 3.3 mmol, 98%).

Analytical Data

^{1}H-NMR (400.1 MHz, $CDCl_3$): δ = 1.30 (s, 1H), 1.78 (dddd, *J* = 13.7, 10.1, 6.3, 5.4 Hz, 1H), 2.01 (dddd, *J* = 13.8, 7.5, 6.9, 4.8 Hz, 1H), 2.25 – 2.36 (m, 2H), 2.78 (ddd, *J* = 10.1, 7.3, 4.8 Hz, 1H), 3.42 – 3.63 (m, 2H), 3.79 (s, 3H), 4.52 – 4.68 (m, 2H), 4.87 – 5.05 (m, 1H), 6.82 – 6.88 (m, 2H), 7.07 – 7.13 (m, 2H) ppm.

^{13}C-NMR (100.6 MHz, $CDCl_3$): δ = 36.2, 38.8, 41.8, 55.3, 61.2, 74.5, 88.1, 114.0, 128.6, 136.3, 158.2, 209.2 ppm.

ESI-HRMS: *m/z* calcd for $C_{14}H_{18}ONa$ $[M+Na]^+$ 241.1199 found 241.1197.

Synthesis of ethyl 3-(4-methylthio)phenyl)hepta-5,6-dienoate 93.

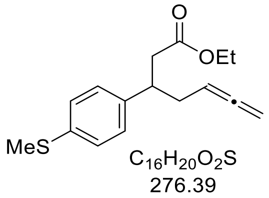

The reaction was performed according to **general procedure 2** with (E)-hepta-2,5,6-trienoate (0.8 g, 5.2 mmol, 1.0 equiv.) and (4-methylthio)phenyl)magnesium bromide (13 mL, 13 mmol, 1.0 M). The crude product was purified by flash chromatography on silica gel (40:1 pentane/Et_2O). The desired product was obtained as a colorless liquid (0.88 g, 3.2 mmol, 61%).

Analytical Data

^{1}H-NMR (400.1 MHz, $CDCl_3$ δ = 1.15 (t, *J*=7.1, 3H), 2.33 (dd, *J* = 7.3, 2.8, 2H), 2.46 (s, 3H), 2.55 (dd, *J* = 15.4, 8.7 Hz, 1H), 2.71 (dd, *J* = 15.3, 6.5 Hz, 1H), 3.20 (ddd, *J* = 8.6, 7.1, 7.1 Hz, 1H), 4.04 (ddd, *J* = 7.1, 1.4, 1.4 Hz, 2H), 4.61 (ddd, *J*=6.5, 2.8, 2.8 Hz 2H), 4.93 (dddd, *J*=7.3, 7.3, 6.7, 6.7 Hz, 1H), 7.10 – 7.14 (m, 2H), 7.18 – 7.22 (m, 2H) ppm.

^{13}C-NMR (100.6 MHz, $CDCl_3$): δ = 14.2, 16.1, 35.4, 40.6, 41.7 , 60.4, 74.8, 87.4, 127.0, 128.1, 136.3, 140.6, 172.2, 209.4 ppm.

APCI-HRMS: *m/z* calcd for $C_{16}H_{20}O_2NaS$ $[M+Na]^+$ 299.1076 found 299.1075.

Synthesis of 3-(4-methylthio)phenyl)hepta-5,6-dienol 94.

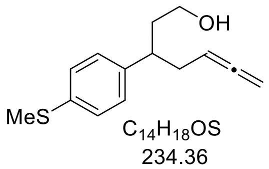

The reaction was performed according to **general procedure 3** with Ethyl 3-(4-methylthio)phenyl)hepta-5,6-dienoate (0.88 g, 3.2 mmol, 1.0 equiv.) and LAH (0.18 mg, 4.8 mmol, 1.5 equiv). The desired product was obtained as a colorless liquid (0.74 g, 3.2 mmol, 99%).

Analytical Data

^{1}H-NMR (400.1 MHz, $CDCl_3$): δ = 1.32 (s, 1H), 1.79 (dddd, *J* = 13.7, 10.0, 6.4, 5.3 Hz, 1H), 2.01 (dddd, *J* = 13.8, 7.7, 6.9, 4.9 Hz, 1H), 2.28 – 2.35 (m, 2H), 2.47 (s, 3H), 2.75 – 2.88 (m, 1H), 3.47 (ddd, *J* = 10.6, 7.7, 6.4 Hz, 1H), 3.56 (dddd, *J* = 10.6, 7.0, 5.3, 0.3 Hz, 1H), 4.53 – 4.65 (m, 2H), 4.88 – 4.98 (m, 1H), 7.09 – 7.13 (m, 2H), 7.19 – 7.23 (m, 2H) ppm.

^{13}C-NMR (100.6 MHz, $CDCl_3$): δ = 16.2, 35.9, 38.6, 42.0, 61.0, 74.6, 87.9, 127.2, 128.3, 136.0, 141.4, 209.2 ppm.

APCI-HRMS: *m/z* calcd for $C_{16}H_{21}O_2S$ $[M+H_3O]^+$ 277.1257 found 277.1256.

Synthesis of ethyl 3-(4-methoxyhenyl)-4-dimethylhepta-5,6-dienoate 95.

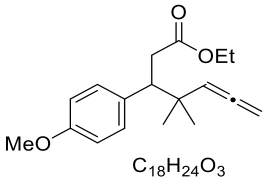

$C_{18}H_{24}O_3$
288.39

The reaction was performed according to **general procedure 2** with (E)-hepta-2,5,6-trienoate (0.8 g, 5.2 mmol, 1.0 equiv.) and (4-methoxyphenyl)magnesium bromide (13 mL, 13 mmol, 1.0 M). The crude product was purified by flash chromatography on silica gel (40:1 pentane/Et_2O). The desired product was obtained as a colorless liquid (0.97 g, 3.4 mmol, 65%).

Analytical Data

^{1}H-NMR (400.1 MHz, $CDCl_3$ δ = 0.92 (s, 3H), 0.99 (s, 3H), 1.04 (t, *J* = 7.1 Hz, 3H), 2.66 (dd, *J* = 15.3, 11.2 Hz, 1H), 2.80 (dd, *J* = 15.4, 4.4 Hz, 1H), 3.02 (dd, *J* = 11.2, 4.5 Hz, 1H), 3.78 (s, 3H), 3.86 – 3.99 (m, 2H), 4.69 – 4.77 (m, 2H), 5.08 (dd, *J* = 6.6, 6.6 Hz, 1H), 6.76 – 6.83 (m, 2H), 7.06 – 7.14 (m, 2H) ppm.

^{13}C-NMR (100.6 MHz, $CDCl_3$): δ = 14.1, 24.7, 27.7, 36.5, 37.6, 51.4, 55.2, 60.2, 98.9, 113.1, 130.4, 132.8, 158.3, 173.0, 207.3 ppm.

APCI-HRMS: *m/z* calcd for $C_{18}H_{28}O_3N$ $[M+NH_4]^+$ 306.2064 found 306.2065.

Synthesis of 3-(4-methoxyhenyl)-4-dimethylhepta-5,6-dienol 96.

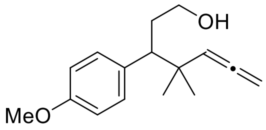

$C_{16}H_{22}O_2$
246.35

The reaction was performed according to **general procedure 3** with 3-(4-methoxyhenyl)-4-dimethylhepta-5,6-dienoate (0.97 g, 3.4 mmol, 1.0 equiv.) and LAH (0.19 g, 5.1 mmol, 1.5 equiv.). The desired product was obtained as a colorless liquid (0.83 g, 3.4 mmol, 99%).

Analytical Data

^{1}H-NMR (400.1 MHz, $CDCl_3$): δ = 0.91 (s, 3H), 0.98 (s, 3H), 1.28 (s, 1H), 1.89 (dddd, *J* = 13.6, 12.2, 6.6, 4.6 Hz, 1H), 2.03 – 2.13 (m, 1H), 2.53 (dd, *J* = 12.2, 2.9 Hz, 1H), 3.35 (ddd, *J* = 10.5, 8.1, 6.6 Hz, 1H), 3.47 (ddd, *J* = 10.5, 7.5, 4.6 Hz, 1H), 3.79 (s, 3H), 4.71 (d, *J* = 6.7 Hz, 2H), 5.10 (dd, *J* = 6.7, 6.7 Hz, 1H), 6.76 – 6.85 (m, 2H), 7.05 – 7.12 (m, 2H) ppm.

^{13}C-NMR (100.6 MHz, $CDCl_3$): δ = 25.0, 27.9, 33.2, 37.7, 52.0, 55.3, 62.0, 99.4, 113.3, 130.4, 133.4, 158.2, 207.3 ppm.

APCI-HRMS: *m/z* calcd for $C_{16}H_{26}O_2N$ $[M+NH_4]^+$ 264.3885 found 264.3886.

2.2 Prepartion internal allenes

2.2.1 General procedure internal allenes

General procedure 5: Synthesis ethyl 3,4-dienoate derivate:

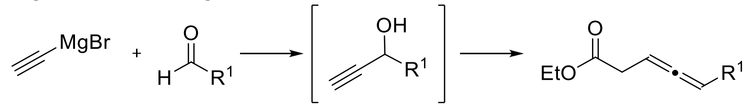

I) A solution of the Aldehyde (1.0 equiv.) in THF (0.5 M) was added to ethynylmagnesium bromide (0.5 M in THF, 1.1 equiv.) within 1 h at 0 °C. The reaction mixture was then stirred for 1 h at room temperature and quenched via addition of saturated aqueous NH_4Cl solution. The phases were separated and the aqueous phase was extracted with Et_2O. The combined organic layers were dried over Na_2SO_4, filtered over a short silica pad and concentrated.

II) To a solution of the crude alcohol (**step I**) (0.5 equiv.) in triethyl orthoacetate (1.05 equiv.) was added dropwise propionic acid (4.0 mol%) at 100 °C. The mixture was heated to 160 °C and resulting EtOH was continuously distilled off under atmospheric pressure for 1 h. Then another aliquot of crude alcohol (**step I**) (0.5 equiv.) and propionic acid (0.80 mL, 0.80 g, 11 mmol, 4.0 mol%) were added dropwise and the mixture was stirred for another 1 h before a third portion of propionic acid (4.0 mol%) was added. The reaction was stirred for an additional hour and then cooled to room temperature and quenched by addition of aqueous HCl-solution (2 M). The organic layer was separated and the aqueous layer was extracted with Et_2O (× 3). The combined organic layers were washed with brine, dried over $MgSO_4$ and the solvent was removed under reduced pressure. The residue was either purified by fractional distillation under reduced pressure or by flash column chromatography eluting with pentane:ether.

General Procedure 6: Synthesis ethyl (E)-2,5,6-trienoate derivate:.

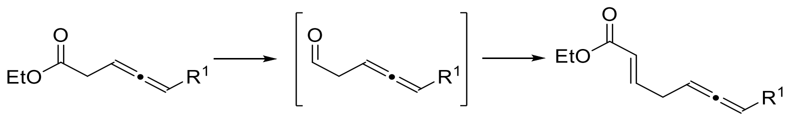

I) At −80°C a solution of DIBAL-H (1.4 equiv., 1.0 M in CH_2Cl_2) was added dropwise over 90 min to a solution of ethylpenta-3,4-dienoate (1.0 equiv.) in CH_2Cl_2 (1.6 M). The reaction mixture was stirred for 1 h and then transferred to an ice cold aqueous solution of HCl (2.0 M) at 0 °C. The layers were separated, the organic layer was washed with HCl (2.0 M, × 2) and the aqueous layer was extracted with CH_2Cl_2 (× 2). The combined organic layers were washed with brine, dried over Na_2SO_4, filtered over a silica pad and concentrated under reduced pressure.

II) A suspension of NaH (60% in mineral oil, 1.1 equiv.) in dry THF (0.25 M) was cooled to 0 °C and triethyl phosphonoacetate (1.3 equiv.) was added dropwise to the reaction mixture. After complete

addition, the suspension was stirred for 1 h at 0 °C and was then cooled to −78 °C. A solution of penta-4,5-dienal (1.0 equiv.) was added to the reaction mixture at this temperature. The mixture was slowly warmed to −20 °C over 3 h and then quenched by the addition of aqueous saturated NH_4Cl-solution. The layers were separated, the aqueous layer was extracted with Et_2O, the combined organic layers were dried over Na_2SO_4 and concentrated under reduced pressure. The residue was purified by flash chromatography on silica gel (Pentane:Et_2O).

General procedure 7: 1,4-Addition and reduction:

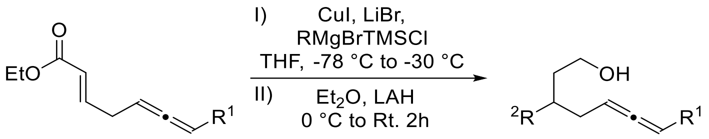

I) A mixture of CuI (10 mol%) and LiBr (20 mol%) was carefully dried and cooled to room temperature under vacuum and backfilled with argon using a standard SCHLENK line apparatus, before extra dry or freshly distilled THF (1.0 M) was added. The mixture was stirred at room temperature for 15 min and then cooled to −78 °C. A solution of ethyl (E)-hepta-2,5,6-trienoate derivative (1.0 equiv) in extra dry or freshly distilled THF (0.3 M) and TMSCl (1.1 equiv.) was added at this temperature. The mixture was stirred for 15 min before the GRIGNARD reagent in THF (1.5 equiv.) was added dropwise, stirred for 30 minutes at −78 °C and then slowly warmed to −30 °C over 3 h. The reaction was quenched by the addition of aqueous saturated NH_4Cl-solution. The layers were separated, the aqueous layer was extracted with Et_2O (× 4), the combined organic layer were washed with brine and dried over Na_2SO_4. The solvent was removed and the residue was filtered over a shirt silica pad

II) The crude product was dissolved in Et_2O () and added dropwise to a suspension of LAH () in dry Et_2O at 0 °C. The reaction was stirred at this temperature for 1 h and then warmed to room temperature and stirred for another hour. The mixture was quenched through the addition of H_2O and aqueous HCl (2.0 M). The layers were separated, the organic layer was washed with H_20 and brine. The aqueous layer was extracted with Et_2O (× 4). The combined organic layer were dried over Na_2SO_4, the solvent was removed. The solvent was removed and the residue was purified by flash chromatography on silica gel using a mixture of pentane and ether.

2.2.2 Synthesis and characterization of internal allenes

Synthesis of ethyl undeca-3,4-dienoate 97.

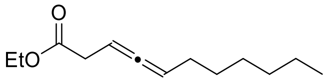

$C_{13}H_{22}O_2$
210.32

The reaction was performed according to **general procedure 5.**

I) Heptanal (21.0 g, 26.0 mL 184 mmol, 1.0 equiv.) and ethinyl magnesium bromide (400 mL, 200 mmol, 0.5 M in THF). The crude product was filtered over a short silica pad (2:1 pentane/Et_2O). Non-1-yn-3-ol was obtained as a yellow liquid (24.9 g, 178 mmol, 97%)

II) Crude non-1-yn-3-ol (step **I**) (24.9 g, 178 mmol), triethyl orthoacetate (30.4 g, 34.3 mL, 187 mmol), propionic acid (3 × 4 mol%). The crude product was purified by fractional distillation under reduced pressure (110 °C, 4 mbar) to obtain the desired product (22.9 g, 109 mmol, 61%) as a colorless liquid.

Analytical Data

^{1}H-NMR (400.1 MHz, $CDCl_3$): δ = 0.89 (t, *J* = 6.8 Hz, 3H), 1.27 (m, 10H), 1.35 – 1.45 (m, 3H), 1.99 (ddd, *J* = 7.6, 6.6, 3.1 Hz, 2H), 3.01 (dd, *J* = 7.1, 2.9 Hz, 2H), 4.16 (ddd, *J* = 7.2 Hz, 2H), 5.12 – 5.28 (m, 2H) ppm.

^{13}C-NMR (100.6 MHz, $CDCl_3$): δ = 14.1, 14.3, 22.7, 28.6, 28.8, 29.1, 31.8, 35.2, 60.7, 84.2, 92.3, 171.7, 205.1 ppm.

ESI-HRMS: *m/z* calcd for $C_{13}H_{22}O_2Na$ $[M+Na]^+$ 233.1512 found 233.1513.

Synthesis of ethyl (E)-trideca-2,5,6-trienoate 98.

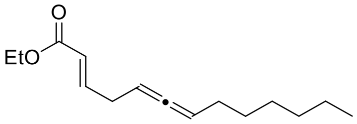

$C_{15}H_{24}O_2$
236.36

The reaction was performed according to **general procedure 6**.

I) ethyl undeca-3,4-dienoate (22.9 g, 109 mmol, 1.0 equiv.) and DIBAL-H (158 mL, 158 mmol, 1.45 equiv., 1.1 M in DCM). The crude product was filtered over a short silica pad (DCM). Undeca-3,4-dienal was obtained as a yellow liquid (16.3 g, 98.1 mmol, 90%).

II) Crude undeca-3,4-dienal (step **I**) (7.0 g, 42 mmol), NaH (60% in mineral oil, 1.8 g, 46 mmol, 1.1 equiv.) in dry THF (168 mL) and triethyl phosphonoacetate (12 g, 11 mL, 55 mmol,1.3 equiv.). The crude product was purified by flash chromatography on silica gel (40:1 Pentane:Et_2O). The title compound was obtained as colorless liquid (7.1 g, 30 mmol, 72 %).

Analytical Data

^{1}H-NMR (400.1 MHz, $CDCl_3$): δ = 0.87 – 0.90 (m, 3H), 1.27 – 1.31 (m, 10H), 1.95 – 2.04 (m, 2H), 2.88 (ddd, *J* = 6.6, 2.8, 1.7 Hz, 2H), 4.19 (ddd, *J* = 7.2 Hz, 2H), 5.08 (ddd, *J* = 6.6 Hz, 3.4, 1H), 5.12 – 5.21 (m, 1H), 5.88 (ddd, *J* = 15.6 Hz, 1.7, 1H), 6.97 (ddd, *J* = 15.7, 6.5 Hz, 1H) ppm.

^{13}C-NMR (100.6 MHz, $CDCl_3$): δ = 14.2, 14.4, 28.8, 28.8, 29.1, 29.8, 31.8, 32.1, 60.3, 87.1, 92.3, 122.0, 146.9, 166.6, 204.9bppm.

ESI-HRMS: *m/z* calcd for $C_{15}H_{25}O_2$ $[M+H]^+$ 237.1849 found 237.1849.

Synthesis of 3-methyltrideca-5,6-dien-1-ol 99.

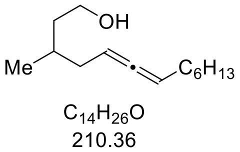

The reaction was performed according to **general procedure 7** with ethyl (E)-trideca-2,5,6-trienoate (0.8 g, 3.4 mmol, 1.0 equiv.) and methylmagnesium bromide (1.7 mL, 5.1 mmol, 3.0 M in Et_2O). The crude product from step I was treated with LAH (0.19 mg, 5.1 mmol, 1.5 equiv.). The crude product from step II was purified by flash chromatography on silica gel (10:1 to 5:1 pentane/Et_2O). The desired product was obtained as a colorless liquid (0.74 g, 3.54 mmol, 68%).

Analytical Data[3]

^{1}H-NMR (400.1 MHz, $CDCl_3$): δ = 0.88 (d, *J* = 7.1 Hz, 3H), 0.95 (m, 3H), 1.25 – 1.33 (m, 7H), 1.37 – 1.45 (m, 3H), 1.61 – 1.74 (m, 2H), 1.83 – 1.95 (m, 1H), 1.95 – 2.06 (m, 3H), 3.63 – 3.74 (m, 2H), 4.94 – 5.11 (m, 2H) ppm.

^{13}C-NMR (125.6 MHz, $CDCl_3$): δ = 28.9, 29.1, 29.3, 29.3, 30.0, 31.8, 36.8, 36.9, 39.4, 39.5, 61.3, 77.4, 88.8, 90.6, 204.7 ppm.

ESI-HRMS: *m/z* calcd for $C_{14}H_{27}O$ $[M+H]^+$ found.

Synthesis of 3-(deca-2,3-dien-1-yl)pentadecan-1-ol 100.

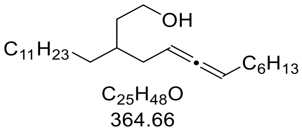

The reaction was performed according to **general procedure 7** with ethyl (E)-trideca-2,5,6-trienoate (0.8 g, 3.4 mmol, 1.0 equiv.) and dodecylmagnesium bromide (5.1 mL, 5.1 mmol, 1.0 M). The crude product from step I was treated with LAH (0.19 mg, 5.1 mmol, 1.5 equiv.). The crude product from step II was purified by flash chromatography on silica gel (10:1 to 5:1 pentane/Et_2O). The desired product was obtained as a colorless liquid (0.68 g, 1.9 mmol, 55%).

Analytical Data[3]

^{1}H-NMR (400.1 MHz, $CDCl_3$): δ = 0.86 – 0.91 (m, 6H), 1.22 – 1.36 (m, 31H), 1.57 (ddd, *J* = 4.5, 1.2 Hz, 3H), 1.98 (dddd, *J* = 10.8, 9.7, 5.4, 2.9 Hz, 4H), 3.65 – 3.73 (m, 2H), 4.94 – 5.11 (m, 2H) ppm.

^{13}C-NMR (100.6 MHz, $CDCl_3$): δ = 14.2, 14.2, 22.7, 22.8, 26.7, 26.8, 28.9, 29.1, 29.4, 29.5, 29.8, 29.8, 30.1, 31.8, 32.0, 33.4, 33.6, 33.7, 33.7, 34.6, 36.9, 61.3, 61.3, 88.5, 88.6, 90.5, 90.6, 204.7, 204.8 ppm.

ESI-HRMS: *m/z* calcd for $C_{25}H_{48}OCl$ $[M+Cl]^+$ 399.3399 found 399.3394.

Synthesis of 3-neopentyltrideca-5,6-dien-1-ol 101.

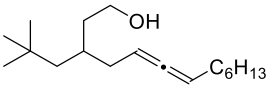

$C_{18}H_{34}O$
266.47

The reaction was performed according to **general procedure 7** with ethyl (E)-trideca-2,5,6-trienoate (0.8 g, 3.4 mmol, 1.0 equiv.) and neopentylmagnesium bromide (5.1 mL, 5.1 mmol, 1.0 M). The crude product from step I was treated with LAH (0.19 mg, 5.1 mmol, 1.5 equiv.). The crude product from step II was purified by flash chromatography on silica gel (10:1 to 5:1 pentane/Et_2O). The desired product was obtained as a colorless liquid (0.44 g, 1.7 mmol, 49%).

Analytical Data[3]

^{1}H-NMR (400.1 MHz, $CDCl_3$): δ = 0.84 – 0.92 (m, 11H), 1.13 (ddd, *J* = 14.1, 4.0 Hz, 1.5, 1H), 1.22 – 1.36 (m, 9H), 1.36 – 1.44 (m, 2H), 1.50 – 1.68 (m, 3H), 1.91 – 2.10 (m, 4H), 3.63 – 3.71 (m, 2H), 5.00 (dddd, *J* = 10.5, 5.3, 4.2, 2.5 Hz, 1H), 5.02 – 5.10 (m, 1H) ppm.

^{13}C-NMR (100.6 MHz, $CDCl_3$): δ = 14.2, 22.7, 28.9, 29.1, 29.4, 30.0, 30.0, 30.9, 31.1, 31.3, 31.8, 35.9, 36.0, 39.0, 39.1, 47.8, 47.8, 61.2, 88.7, 88.8, 90.5, 90.6, 204.9, 204.9 ppm.

ESI-HRMS: *m/z* calcd for $C_{18}H_{34}OCl$ $[M+Cl]^+$ 252.2443 found 252.2451.

Synthesis of 3-cyclopropyltrideca-5,6-dien-1-ol 102.

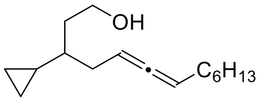

$C_{16}H_{28}O$
236.40

The reaction was performed according to **general procedure 7** with ethyl (E)-trideca-2,5,6-trienoate (0.8 g, 3.4 mmol, 1.0 equiv.) and cyclopropylmagnesium bromide (5.1 mL, 5.1 mmol, 1.0 M). The crude product from step I was treated with LAH (0.19 mg, 5.1 mmol, 1.5 equiv.). The crude product from step II was purified by flash chromatography on silica gel (10:1 to 5:1 pentane/Et_2O). The desired product was obtained as a colorless liquid (0.50 g, 2.1 mmol, 63%).

Analytical Data[3]

^{1}H-NMR (400.1 MHz, $CDCl_3$): δ = 0.07 – 0.17 (m, 2H), 0.42 – 0.53 (m, 2H), 0.58 (dddd, *J* = 15.7, 9.7, 5.0, 2.5 Hz, 1H), 0.73 – 0.85 (m, 1H), 0.86 – 0.92 (m, 3H), 1.23 – 1.43 (m, 9H), 1.60 – 1.71 (m, 1H), 1.72 – 1.83 (m, 1H), 1.93 – 2.01 (m, 2H), 2.02 – 2.13 (m, 1H), 2.13 – 2.22 (m, 1H), 3.72 – 3.82 (m, 2H), 5.01 – 5.11 (m, 2H) ppm.

^{13}C-NMR (100.6 MHz, $CDCl_3$): δ = 3.8, 3.9 , 4.6, 4.7, 14.2, 16.0, 16.1, 22.7, 28.9, 29.1, 29.3, 29.4, 31.8, 35.1, 35.2, 37.7, 37.7, 41.1, 41.1, 61.4, 61.5, 88.8, 88.8, 90.5, 204.6, 204.7 ppm.

APCI-HRMS: *m/z* calcd for $C_{16}H_{29}O$ $[M+HI]^+$ 237.2213 found 237.2212.

Synthesis of 3-cyclohexyltrideca-5,6-dien-1-ol 103.

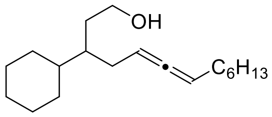

$C_{19}H_{34}O$
278.48

The reaction was performed according to **general procedure 7** with ethyl (E)-trideca-2,5,6-trienoate (0.7 g, 2.9 mmol, 1.0 equiv.) and cyclohexylmagnesium bromide (4.4 mL, 4.4 mmol, 1.5 equiv. 1.0 M). The crude product from **step I** was treated with LAH (0.19 mg, 5.1 mmol, 1.5 equiv.). The crude product from **step II** was purified by flash chromatography on silica gel (10:1 to 3:1 pentane/Et_2O). The desired product was obtained as a colorless liquid (0.5 g, 1.8 mmol, 62%).

Analytical Data[3]

^{1}H-NMR (400.1 MHz, $CDCl_3$): δ = 0.86 – 0.91 (m, 3H), 1.00 – 1.24 (m, 5H), 1.24 – 1.35 (m, 8H), 1.35 – 1.43 (m, 4H), 1.44 – 1.55 (m, 1H), 1.56 – 1.70 (m, 4H), 1.70 – 1.78 (m, 2H), 1.87 – 2.12 (m, 4H), 3.56 – 3.76 (m, 2H), 4.90 – 5.13 (m, 2H) ppm.

^{13}C-NMR (100.6 MHz, $CDCl_3$): δ = 14.2, 22.7, 26.9, 26.9, 27.0, 27.0, 28.9, 29.1, 29.1, 29.4, 29.4, 29.5, 29.8, 30.1, 30.1, 31.0, 31.2, 31.8, 34.0, 34.1, 40.1, 40.3, 40.4, 40.5, 62.0, 89.6, 89.7, 90.7, 90.8, 204.5, 204.6 ppm.

APCI-HRMS: *m/z* calcd for $C_{19}H_{35}O$ [M+HI]$^+$ 279.2672 found 279.2674.

Synthesis of 3-(3-phenylpropyl)trideca-5,6-dien-1-ol 104.

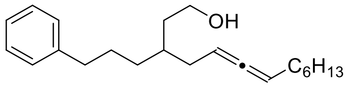

$C_{22}H_{34}O$
314.51

The reaction was performed according to **general procedure 7** with ethyl (E)-trideca-2,5,6-trienoate (0.7 g, 2.9 mmol, 1.0 equiv.) and (3-phenylpropyl)magnesium bromide (4.4 mL, 4.4 mmol, 1.5 equiv. 1.0 M). The crude product from **step I** was treated with LAH (0.19 mg, 5.1 mmol, 1.5 equiv.). The crude product from **step II** was purified by flash chromatography on silica gel (10:1 to 3:1 pentane/Et_2O). The desired product was obtained as a colorless liquid (0.48 g, 1.5 mmol, 53%).

Analytical Data[3]

^{1}H-NMR (400.1 MHz, $CDCl_3$): δ = 0.86 – 0.92 (m, 3H), 1.24 – 1.45 (m, 11H), 1.53 – 1.72 (m, 5H), 1.91 – 2.04 (m, 4H), 2.60 (td, *J*=7.6, 1.8, 2H), 3.62 – 3.69 (m, 2H), 4.88 – 5.10 (m, 2H), 7.12 – 7.22 (m, 3H), 7.25 – 7.30 (m, 2H) ppm.

^{13}C-NMR (100.6 MHz, $CDCl_3$): δ = 14.2, 22.7, 28.6, 28.7, 28.9, 29.1, 29.4, 31.8, 33.3, 33.3, 33.4, 33.5, 34.5, 34.6, 36.3, 36.8, 61.2, 61.2, 88.4, 88.5, 90.6, 90.7, 125.7, 128.3, 128.4, 142.7, 204.8, 204.8 ppm.

APCI-HRMS: *m/z* calcd for $C_{22}H_{38}ON$ [M+NH$_4$I]$^+$ 332.2948 found 332.2950.

Synthesis of 3-phenyltrideca-5,6-dien-1-ol 105.

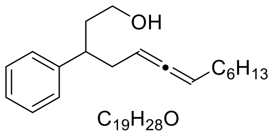

$C_{19}H_{28}O$
272.43

The reaction was performed according to **general procedure 7** with ethyl (E)-trideca-2,5,6-trienoate (0.7 g, 2.9 mmol, 1.0 equiv.) and phenylmagnesium bromide (4.4 mL, 4.4 mmol, 1.5 equiv. 1.0 M). The crude product from **step I** was treated with LAH (0.19 mg, 5.1 mmol, 1.5 equiv.). The crude product from **step II** was purified by flash chromatography on silica gel (10:1 to 3:1 pentane/Et_2O). The desired product was obtained as a colorless liquid (0.51 g, 0.19 mmol, 65%).

Analytical Data[3]
^{1}H-NMR (400.1 MHz, $CDCl_3$): δ = 0.87 – 0.91 (m, 3H), 1.20 – 1.41 (m, 9H), 1.78 – 1.96 (m, 3H), 1.99 – 2.09 (m, 1H), 2.24 – 2.40 (m, 2H), 2.82 (dddd, *J* = 14.4, 9.9, 7.0, 4.8, 1H), 3.44 – 3.61 (m, 2H), 4.86 – 4.96 (m, 1H), 4.96 – 5.04 (m, 1H), 7.16 – 7.23 (m, 3H), 7.27 – 7.33 (m, 2H) ppm.
^{13}C-NMR (100.6 MHz, $CDCl_3$): δ = 14.2, 22.7, 28.9, 28.9, 28.9, 28.9, 29.2, 29.2, 31.8, 31.8, 36.6, 36.9, 38.8, 38.9, 42.6, 42.8, 61.2, 88.7, 88.8, 90.9, 91.0, 126.4, 126.4, 127.8, 128.5, 144.6, 204.7, 204.7 ppm.
APCI-HRMS: *m/z* calcd for $C_{19}H_{28}OCl$ $[M+Cl]^+$ 307.1833 found 307.1834.

Synthesis of 3-(naphthalen-2-yl)trideca-5,6-dien-1-ol 106.

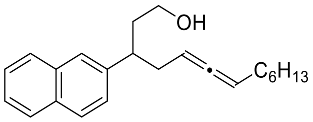

$C_{23}H_{30}O$
322.49

The reaction was performed according to **general procedure 7** with ethyl (E)-trideca-2,5,6-trienoate (0.80 g, 3.4 mmol, 1.0 equiv.) and naphthalen-2-ylmagnesium bromide (5.1 mL, 5.1 mmol, 1.0 M). The crude product from step I was treated with LAH (0.19 mg, 5.1 mmol, 1.5 equiv.). The crude product from step II was purified by flash chromatography on silica gel (10:1 to 5:1 pentane/Et_2O). The desired product was obtained as a colorless liquid (0.74 g, 2.3 mmol, 68%).

Analytical Data[3]

^{1}H-NMR (400.1 MHz, $CDCl_3$): δ = 0.88 (ddd, *J* = 7.0, 4.8 Hz, 3H), 1.15 – 1.32 (m, 9H), 1.75 (dddd, *J* = 8.1, 6.7, 6.0, 3.0 Hz, 1H), 1.81 – 1.89 (m, 1H), 1.89 – 1.99 (m, 1H), 2.06 – 2.18 (m, 1H), 2.42 (dddd, *J* = 10.1, 4.5, 3.6, 2.6 Hz, 2H), 3.01 (dddd, *J* = 9.8, 7.1, 4.8 Hz, 1H), 3.47 – 3.63 (m, 2H), 4.88 – 5.04 (m, 2H), 7.35 (ddd, *J* = 8.5, 1.6 Hz, 1H), 7.40 – 7.48 (m, 2H), 7.62 (d, *J* = 1.7 Hz, 1H), 7.77 – 7.82 (m, 3H).ppm.

^{13}C-NMR (100.6 MHz, $CDCl_3$): δ = 14.2, 14.2, 22.7, 22.7, 28.8, 28.9, 28.9, 29.2, 29.2, 31.7, 31.8, 36.5, 36.9, 38.7, 38.8, 42.6, 42.9, 61.2, 61. , 88.7, 88.8, 90.9, 91.1, 125.4, 126.0, 126.0, 126.0, 126.5, 126.6, 127.6, 127.7, 128.2, 132.5, 132.5, 133.6, 142.0, 142.0, 204.7, 204.7 ppm.

ESI-HRMS: *m/z* calcd for $C_{23}H_{34}ON$ $[M+NH_4]^+$ 340.2635 found 340.2638.

Synthesis of 3-([1,1'-biphenyl]-4-yl)trideca-5,6-dien-1-ol 107

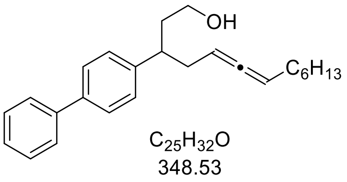

The reaction was performed according to **general procedure 7** with ethyl (E)-trideca-2,5,6-trienoate (0.7 g, 2.9 mmol, 1.0 equiv.) and [1,1'-biphenyl]-4ylmagnesium bromide (4.4 mL, 4.4 mmol, 1.5 equiv. 1.0 M). The crude product from **step I** was treated with LAH (0.19 mg, 5.1 mmol, 1.5 equiv.). The crude product from **step II** was purified by flash chromatography on silica gel (10:1 to 3:1 pentane/Et_2O). The desired product was obtained as a colorless liquid (0.59 g, 1.7 mmol, 59%).

Analytical Data[3]

^{1}H-NMR (400.1 MHz, $CDCl_3$): δ = = 0.80 – 0.92 (m, 3H), 1.15 – 1.34 (m, 9H), 1.77 – 1.99 (m, 3H), 1.99 – 2.15 (m, 1H), 2.27 – 2.46 (m, 2H), 2.82 – 2.95 (m, 1H), 3.47 – 3.67 (m, 2H), 4.91 – 5.05 (m, 2H), 7.23 – 7.26 (m, 1H), 7.27 (d, *J*=0.5, 1H), 7.29 – 7.36 (m, 1H), 7.40 – 7.45 (m, 2H), 7.51 – 7.56 (m, 2H), 7.56 – 7.61 (m, 2H) ppm.

^{13}C-NMR (100.6 MHz, $CDCl_3$): δ = 14.1, 14.2, 22.7, 22.7, 28.9, 28.9, 28.9, 28.9, 29.2, 29.3, 31.8, 31.8, 36.6, 36.9, 38.8, 38.9, 42.2, 42.4, 61.2, 88.7, 88.8, 90.9, 91.1, 127.0, 127.1, 127.2, 128.2, 128.8, 139.3, 139.3, 141.1, 143.7, 143.7, 204.7, 204.8 ppm.

ESI-HRMS: *m/z* calcd for $C_{25}H_{36}ON$ $[M+NH_4]^+$ 366.2791 found 366.2795.

Synthesis of 3-(p-tolyl)trideca-5,6-dien-1-ol 108.

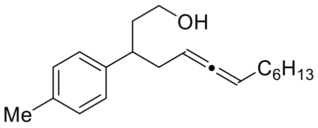

$C_{20}H_{30}O$
286.46

The reaction was performed according to **general procedure 7** with ethyl (E)-trideca-2,5,6-trienoate (0.7 g, 2.9 mmol, 1.0 equiv.) and *p*-tolylmagnesium bromide (4.4 mL, 4.4 mmol, 1.5 equiv. 1.0 M). The crude product from **step I** was treated with LAH (0.19 mg, 5.1 mmol, 1.5 equiv.). The crude product from **step II** was purified by flash chromatography on silica gel (10:1 to 3:1 pentane/Et_2O). The desired product was obtained as a colorless liquid (0.48 g, 1.7 mmol, 58%).

Analytical Data[3]

^{1}H-NMR (400.1 MHz, $CDCl_3$): δ = 0.85 – 0.94 (m, 3H), 1.23 – 1.36 (m, 9H), 1.74 – 1.86 (m, 2H), 1.90 (ddd, *J* = 9.6, 7.4, 4.8, 1H), 1.96 – 2.07 (m, 1H), 2.26 – 2.31 (m, 2H), 2.32 (m, 3H), 2.78 (dddd, *J* = 14.5, 10.0, 7.0, 4.7, 1H), 3.45 – 3.62 (m, 2H), 4.86 – 4.96 (m, 1H), 4.96 – 5.05 (m, 1H), 7.05 – 7.13 (m, 4H) ppm.

^{13}C-NMR (100.6 MHz, $CDCl_3$): δ = 14.2, 21.1, 22.7, 28.9, 28.9, 29.0, 29.2, 29.3, 31.8, 31.8, 36.7, 37.0, 38.8, 38.9, 42.2, 42.4, 61.3, 61.3, 88.8, 88.9, 90.8, 90.9, 127.6, 127.7, 129.2, 135.8, 141.5, 141.5, 204.6, 204.7 ppm.

APCI-HRMS: *m/z* calcd for $C_{20}H_{34}ON$ $[M+NH_4]^+$ 304.2635 found 304.2634.

Synthesis of 3-(m-tolyl)trideca-5,6-dien-1-ol 109.

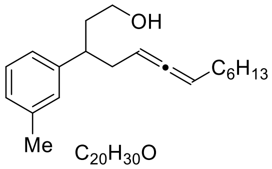

The reaction was performed according to **general procedure 7** with ethyl (E)-trideca-2,5,6-trienoate (0.7 g, 2.9 mmol, 1.0 equiv.) and *m*-tolylmagnesium bromide (4.4 mL, 4.4 mmol, 1.5 equiv. 1.0 M). The crude product from **step I** was treated with LAH (0.19 mg, 5.1 mmol, 1.5 equiv.). The crude product from **step II** was purified by flash chromatography on silica gel (10:1 to 3:1 pentane/Et_2O). The desired product was obtained as a colorless liquid (0.50 g, 1.8 mmol, 61%).

Analytical Data[3]

^{1}H-NMR (400.1 MHz, $CDCl_3$): δ = 0.85 – 0.92 (m, 3H), 1.23 – 1.35 (m, 9H), 1.77 – 1.95 (m, 3H), 1.98 – 2.07 (m, 1H), 2.27 – 2.33 (m, 2H), 2.33 (s, 3H), 2.78 (dddd, *J* = 10.1, 7.3, 4.8, 1H), 3.45 – 3.59 (m, 2H), 4.87 – 4.97 (m, 1H), 4.97 – 5.04 (m, 1H), 6.93 – 7.03 (m, 3H), 7.16 – 7.20 (m, 1H) ppm.

^{13}C-NMR (100.6 MHz, $CDCl_3$): δ = 14.2, 21.6, 22.7, 28.9, 28.9, 28.9, 29.0, 29.2, 31.8, 31.8, 36.7, 37.0, 38.8, 38.9, 42.5, 42.8, 61.3, 88.9, 90.8, 91.0, 124.8, 124.8, 127.1, 128.4, 128.6, 128.6, 138.0, 144.6, 204.7 ppm.

APCI-HRMS: *m/z* calcd for $C_{20}H_{34}ON$ $[M+NH_4]^+$ 304.2635 found 304.2635.

Synthesis of 3-(o-tolyl)trideca-5,6-dien-1-ol 110.

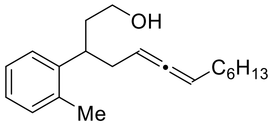

$C_{20}H_{30}O$
286.46

The reaction was performed according to **general procedure 7** with ethyl (E)-trideca-2,5,6-trienoate (0.8 g, 3.4 mmol, 1.0 equiv.) and *o*-tolylmagnesium bromide (5.1 mL, 5.1 mmol, 1.0 M). The crude product from step I was treated with LAH (0.19 mg, 5.1 mmol, 1.5 equiv.). The crude product from step II was purified by flash chromatography on silica gel (10:1 to 5:1 pentane/Et_2O). The desired product was obtained as a colorless liquid (0.58 g, 2.0 mmol, 60%).

Analytical Data[3]

^{1}H-NMR (400.1 MHz, $CDCl_3$): δ = 0.87 – 0.90 (m, 3H), 1.23 – 1.32 (m, 9H), 1.78 – 1.93 (m, 3H), 1.99 – 2.09 (m, 1H), 2.27 – 2.33 (m, 2H), 2.34 (dd, *J* = 1.2, 0.7 Hz, 3H), 3.17 (dddd, *J* = 12.9, 9.4, 5.1, 2.0 Hz, 1H), 3.49 (dddd, *J* = 10.6, 7.4, 6.5, 3.5 Hz, 1H), 3.58 (dddd, *J* = 10.6, 6.9, 5.5, 2.8 Hz, 1H), 4.87 – 4.96 (m, 1H), 4.96 – 5.04 (m, 1H), 7.05 – 7.10 (m, 1H), 7.11 – 7.17 (m, 2H), 7.18 (t, *J* = 1.1 Hz, 1H).ppm.

^{13}C-NMR (100.6 MHz, $CDCl_3$): δ = 14.2, 20.0, 20.0, 22.7, 28.9, 28.9, 29.2, 29.2, 31.8, 31.8, 36.3, 36.8, 36.8, 37.1, 38.7, 38.7, 61.2, 61.3, 88.7, 88.8, 90.8, 91.0, 125.9, 125.9, 126.0, 126.3, 126.3, 130.3, 130.4, 136.2, 136.3, 142.8, 142.9, 204.6, 204.7 ppm.

APCI-HRMS: *m/z* calcd for $C_{20}H_{34}ON$ $[M+NH_4]^+$ 304.2635 found 304.2635.

Synthesis of 3-mesityltrideca-5,6-dien-1-ol 111.

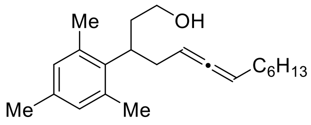

$C_{22}H_{34}O$
314.51

The reaction was performed according to **general procedure 7** with ethyl (E)-trideca-2,5,6-trienoate (0.8 g, 3.4 mmol, 1.0 equiv.) and mesitylmagnesium bromide (5.1 mL, 5.1 mmol, 1.0 M). The crude product from step I was treated with LAH (0.19 mg, 5.1 mmol, 1.5 equiv.). The crude product from step II was purified by flash chromatography on silica gel (10:1 to 4:1 pentane/Et_2O). The desired product was obtained as a colorless liquid (0.55 g, 1.7 mmol, 51%).

Analytical Data[3]

^{1}H-NMR (400.1 MHz, $CDCl_3$): δ = 0.87 – 0.92 (m, 3H), 1.24 – 1.37 (m, 9H), 1.78 – 1.86 (m, 1H), 1.92 – 1.97 (m, 1H), 2.01 – 2.08 (m, 2H), 2.23 (s, 3H), 2.33 (s, 3H), 2.36 (s, 3H), 2.40 – 2.49 (m, 2H), 3.32 – 3.42 (m, 1H), 3.48 – 3.61 (m, 2H), 4.92 – 4.99 (m, 1H), 4.99 – 5.06 (m, 1H), 6.76 – 6.85 (m, 2H) ppm.

^{13}C-NMR (100.6 MHz, $CDCl_3$): δ = 14.2, 20.7, 21.5, 21.9, 22.7, 28.9, 29.0, 29.1, 29.3, 29.3, 31.8, 31.8, 34.2, 34.4, 36.7, 36.8, 37.3, 37.6, 62.0, 89.5, 89.7, 90.9, 91.1, 129.3, 129.3, 131.2, 135.3, 137.0, 137.4, 204.2 ppm.

APCI-HRMS: *m/z* calcd for $C_{22}H_{38}ON$ $[M+NH_4]^+$ 332.2948 found 332.2944.

Synthesis of 3-(4-vinylphenyl)trideca-5,6-dien-1-ol 112.

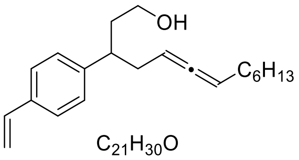

$C_{21}H_{30}O$
298.47

The reaction was performed according to **general procedure 7** with ethyl (E)-trideca-2,5,6-trienoate (0.8 g, 3.4 mmol, 1.0 equiv.) and (4-vinylphenyl)magnesium bromide (5.1 mL, 5.1 mmol, 1.0 M). The crude product from step I was treated with LAH (0.19 mg, 5.1 mmol, 1.5 equiv.). The crude product from step II was purified by flash chromatography on silica gel (10:1 to 5:1 pentane/Et_2O). The desired product was obtained as a colorless liquid (0.64g, 2.1 mmol, 63%).

Analytical Data[3]

^{1}H-NMR (400.1 MHz, $CDCl_3$): δ = 0.86 – 0.94 (m, 3H), 1.21 – 1.35 (m, 9H), 1.81 (dddd, *J* = 13.7, 10.0, 6.4, 5.5 Hz, 2H), 1.90 (dddd, *J* = 7.0, 7.0, 7.0, 3.0 Hz, 1H), 1.95 – 2.09 (m, 1H), 2.21 – 2.41 (m, 2H), 2.82 (dddd, *J* = 10.5, 7.7, 7.7, 7.7, 4.9 Hz, 1H), 3.39 – 3.64 (m, 2H), 4.86 – 4.97 (m, 1H), 4.97 – 5.08 (m, 1H), 5.20 (ddd, *J* = 10.9, 1.0, 1.0 Hz, 1H), 5.71 (ddd, *J* = 17.6, 1.2, 1.2 Hz, 1H), 6.69 (ddd, *J* = 17.6, 10.9, 1.2 Hz, 1H), 7.05 – 7.20 (m, 2H), 7.32 – 7.42 (m, 2H) ppm.

^{13}C-NMR (100.6 MHz, $CDCl_3$): δ = 14.2, 22.7, 28.9, 28.9, 29.2, 29.2, 31.8, 31.8, 36.5, 36.9, 38.8, 38.8, 42.3, 42.5, 61.2, 61.2, 88.7, 88.7, 90.9, 91.1, 113.2, 113.2, 126.4, 128.0, 135.8, 135.8, 136.7, 144.3, 144.4, 204.7, 204.7 ppm.

APCI-HRMS: *m/z* calcd for $C_{21}H_{31}O$ $[M+H]^+$ 299.2369 found 299.2371.

Synthesis of 3-(4-bromophenyl)trideca-5,6-dien-1-ol 113

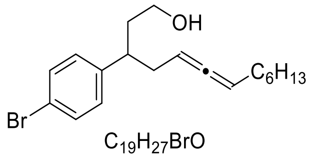

$C_{19}H_{27}BrO$
351.33

The reaction was performed according to **general procedure 7** with ethyl (E)-trideca-2,5,6-trienoate (0.7 g, 2.9 mmol, 1.0 equiv.) and (4-bromophenyl)magnesium bromide (4.4 mL, 4.4 mmol, 1.5 equiv. 1.0 M). The crude product from **step I** was treated with LAH (0.19 mg, 5.1 mmol, 1.5 equiv.). The crude product from **step II** was purified by flash chromatography on silica gel (10:1 to 3:1 pentane/Et_2O). The desired product was obtained as a colorless liquid (0.43 g, 1.2 mmol, 43%).

Analytical Data[3]

^{1}H-NMR (400.1 MHz, $CDCl_3$): δ = 0.87 – 0.91 (m, 3H), 1.22 – 1.34 (m, 9H), 1.75 – 1.96 (m, 3H), 1.96 – 2.12 (m, 1H), 2.23 – 2.39 (m, 2H), 2.72 – 2.88 (m, 1H), 3.44 – 3.62 (m, 2H), 4.87 – 4.96 (m, 1H), 4.96 – 5.04 (m, 1H), 7.17 – 7.21 (m, 2H), 7.27 – 7.33 (m, 2H) ppm.

^{13}C-NMR (100.6 MHz, $CDCl_3$): δ = 14.2, 22.7, 28.9, 28.9, 28.9, 28.9, 29.2, 29.2, 31.8, 31.8, 36.6, 37.0, 38.8, 38.9, 42.6, 42.8, 61.3, 88.7, 88.8, 90.9, 91.0, 126.4, 126.4, 127.8, 128.5, 129.6, 144.6, 144.6, 204.7, 204.7 ppm.

APCI-HRMS: *m/z* calcd for $C_{19}H_{28}O_2Br$ $[M+H]^+$ 352.1318 found 352.1318.

Synthesis of 3-(4-methoxyphenyl)trideca-5,6-dien-1-ol 114.

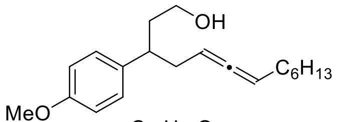

$C_{20}H_{30}O_2$
302.46

The reaction was performed according to **general procedure 7** with ethyl (E)-trideca-2,5,6-trienoate (0.8 g, 3.4 mmol, 1.0 equiv.) and (4-methoxyphenyl)magnesium bromide (5.1 mL, 5.1 mmol, 1.0 M). The crude product from step I was treated with LAH (0.19 mg, 5.1 mmol, 1.5 equiv.). The crude product from step II was purified by flash chromatography on silica gel (10:1 to 5:1 pentane/Et_2O). The desired product was obtained as a colorless liquid (0.68 g, 2.2 mmol, 66%).

Analytical Data[3]
^{1}H-NMR (400.1 MHz, $CDCl_3$): δ = 0.86 – 0.91 (m, 3H), 1.23 – 1.34 (m, 9H), 1.72 – 1.87 (m, 2H), 1.87 – 1.96 (m, 1H), 1.96 – 2.09 (m, 1H), 2.19 – 2.38 (m, 2H), 2.71 – 2.85 (m, 1H), 3.44 – 3.52 (m, 1H), 3.56 (dddd, *J* = 10.6, 6.8, 5.5, 1.3 Hz, 1H), 3.79 (s*, 1.4H), 3.79 (s*, 1.6), 4.86 – 4.97 (m, 1H), 4.97 – 5.04 (m, 1H), 6.81 – 6.87 (m, 2H), 7.07 – 7.13 (m, 2H).ppm.
^{13}C-NMR (100.6 MHz, $CDCl_3$): δ = 14.2, 22.7, 28.9, 28.9, 29.0, 29.2, 29.3, 31.8, 31.8, 36.8, 37.1, 38.9, 39.0, 41.8, 42.0, 55.3, 61.3, 88.8, 88.8, 90.8 90.9, 113.9, 128.7, 136.6, 136.6, 158.1, 158.2, 204.6, 204.7 ppm.
ESI-HRMS: *m/z* calcd for $C_{20}H_{29}O_2$ $[M+H]^-$ 301.2162 found 301.2162.

Synthesis of 3-(4-(methylthio)phenyl)trideca-5,6-dien-1-ol 115.

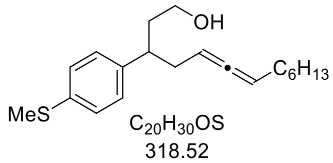

The reaction was performed according to **general procedure 7** with ethyl (E)-trideca-2,5,6-trienoate (0.70 g, 3.0 mmol, 1.0 equiv.) and (4-(methylthio)phenyl)magnesium bromide (4.5 mL, 4.5 mmol, 1.5 equiv. 1.0 M). The crude product from **step I** was treated with LAH (0.17 mg, 4.5 mmol, 1.5 equiv.). The crude product from **step II** was purified by flash chromatography on silica gel (10:1 to 3:1 pentane/Et_2O). The desired product was obtained as a colorless liquid (0.60 g, 1.9 mmol, 63%).

Analytical Data[3]

^{1}H-NMR (400.1 MHz, $CDCl_3$): δ = 0.89 (m_c, 3H), 1.24 – 1.33 (m, 9H), 1.73 – 1.86 (m, 2H), 1.86 – 1.96 (m, 1H), 1.96 – 2.08 (m, 1H), 2.23 – 2.36 (m, 2H), 2.47 (d, *J*=1.5, 3H), 2.79 (dddd, *J* = 13.2, 8.4, 6.8, 4.8 Hz, 1H), 3.42 – 3.51 (m, 1H), 3.56 (dddd, *J* = 10.6, 6.9, 5.3, 1.6 Hz, 1H), 4.90 (ddddd, *J* = 12.3, 9.2, 7.3, 6.1, 3.0 Hz, 1H), 4.99 (ddddd, *J* = 6.5, 6.5 4.0, 2.6, 2.5 Hz, 1H), 7.08 – 7.13 (m, 2H), 7.19 – 7.22 (m, 2H) ppm.

^{13}C-NMR (100.6 MHz, $CDCl_3$): δ = 14.2, 16.3, 22.8, 28.9, 29.2, 29.3, 31.8, 31.8, 36.6, 36.9, 38.7, 38.8, 42.0, 61.1, 88.7, 91.1, 127.1, 128.4, 135.9, 141.6, 204.6 ppm.

APCI-HRMS: *m/z* calcd for $C_{20}H_{31}OS$ $[M+H]^+$ 319.2096 found 319.2087.

Synthesis of 3-(4-(trifluoromethyl)phenyl)trideca-5,6-dien-1-ol 116.

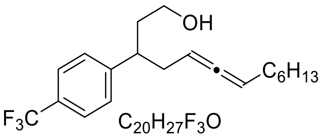

$C_{20}H_{27}F_3O$
340.20

The reaction was performed according to **general procedure 7** with ethyl (E)-trideca-2,5,6-trienoate (0.70 g, 3.0 mmol, 1.0 equiv.) and (4-(trifluoromethyl)phenyl)magnesium bromide (4.5 mL, 4.5 mmol, 1.5 equiv. 1.0 M). The crude product from **step I** was treated with LAH (0.17 mg, 4.5 mmol, 1.5 equiv.). The crude product from **step II** was purified by flash chromatography on silica gel (10:1 to 3:1 pentane/Et_2O). The desired product was obtained as a colorless liquid (0.46 g, 1.4 mmol, 45%).

Analytical Data[3]

^{1}H-NMR (400.1 MHz, $CDCl_3$): δ = 0.86 – 0.93 (m, 3H), 1.14 (s, 1H), 1.23 – 1.31 (m, 8H), 1.75 – 1.90 (m, 3H), 1.98 – 2.13 (m, 1H), 2.26 – 2.42 (m, 2H), 2.94 (ddd, *J* = 14.5, 9.6, 6.4 Hz, 1H), 3.46 (dddd, *J* = 10.6, 7.5, 6.3, 0.9 Hz, 1H), 3.57 (dddd, *J* = 10.6, 6.8, 5.3, 1.5 Hz, 1H), 4.84 – 4.94 (m, 1H), 4.94 – 5.03 (m, 1H), 7.27 – 7.32 (m, 2H), 7.53 – 7.57 (m, 2H) ppm.

^{13}C-NMR (100.6 MHz, $CDCl_3$): δ = 14.1, 14.1, 22.7, 28.8, 28.9, 29.2, 29.2, 31.7, 31.8, 36.2, 36.6, 38.7, 38.7, 42.2, 42.5, 60.8, 60.8, 88.2, 88.3, 91.2, 91.3, 125.4, 125.4, 125.4, 128.2, 204.8 ppm.

APCI-HRMS: *m/z* calcd for $C_{20}H_{28}OF_3$ $[M+H]^+$ 341.2092 found 341.2089.

Synthesis of 4-(4-methoxyphenyl)-2-methyltetradeca-6,7-dien-2-ol 117.

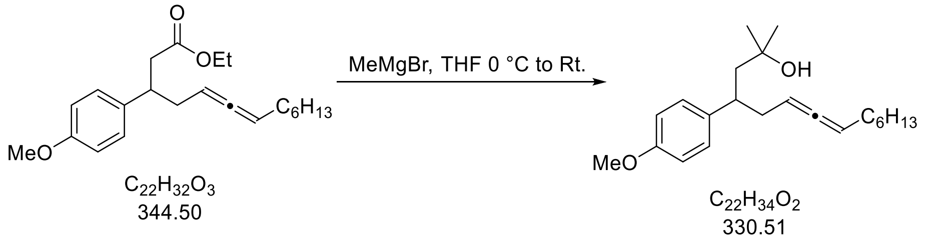

A solution of MeMgBr (2.5 mL, 7.3 mmol, 2.5 equiv, 3 M in Et_2O) in THF (10 mL) was cooled to 0 °C and the crude ethyl 3-(4-methoxyphenyl)trideca-5,6-dienoate (1.0 g, 2.9 mmol, 1.0 equiv.) in THF (5 mL) was added dropwise. The Mixture was stirred for 30 minutes and then warmed to room temperature and stirred for 2 hours. Saturated ammonium chloride (20 mL) was added and the aqueous layer was separated and extracted with Et_2O (2 × 30 mL). The combined organic layers were dried over Na_2SO_4, the solvent was removed under reduced pressure and the two Diastereomers were separated by flash Chromatography (SiO2, pentane/Et2O = 2/1). The title compound was obtained (0.85 g, 2.6 mmol, 89%) as a colorless oil.

Analytical Data (*syn*-product)[3]

^{1}H-NMR (400.1 MHz, $CDCl_3$): δ = 0.86 – 0.92 (m, 3H), 1.11 (m_c, 3H), 1.14 (m_c, 3H), 1.23 – 1.39 (m, 9H), 1.79 – 1.86 (m, 1H), 1.89 – 1.97 (m, 3H), 2.15 – 2.32 (m, 2H), 2.83 (m_c, 1H), 3.78 (m_c, 3H), 4.82 – 4.93 (m, 1H), 4.96 – 5.05 (m, 1H), 6.81 – 6.88 (m, 2H), 7.11 – 7.17 (m, 2H) ppm.

^{13}C-NMR (100.6 MHz, $CDCl_3$): δ = 14.2, 22.7, 28.9, 28.9, 28.9, 29.0, 29.2, 29.9, 30.3, 30.3, 31.8, 31.8, 38.8, 39.1, 41.5, 41.7, 48.8, 49.0, 55.3, 55.3, 71.5, 88.9, 88.9, 90.8, 90.9, 114.1, 128.8, 128.9, 158.2 ppm.

ESI-HRMS: *m/z* calcd for $C_{22}H_{38}O_2$ $[M+NH_4]^+$ 348.2897 found 348.2893.

Synthesis of 7-cyclohexyl-3-(4-methoxyphenyl)hepta-5,6-dien-1-ol

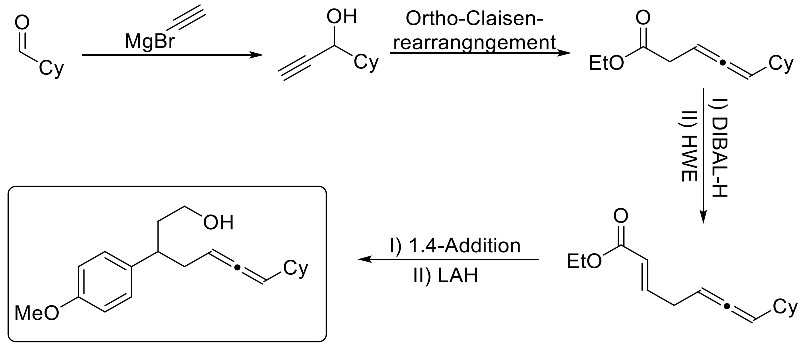

Synthesis of ethyl 5-cyclohexylpenta-3,4-dienoate 118.

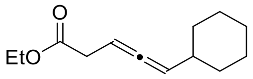

$C_{13}H_{20}O_2$
208.30

The reaction was performed according to **general procedure 5.**

I) Cyclohexanecarbaldehyde (11 g, 93 mmol, 1.0 equiv.) and ethinyl magnesium bromide (200 mL, 100 mmol, 0.5 M in THF, 1.08 equiv.). The crude product was filtered over a short silica pad (2:1 pentane/Et_2O). 1-cyclohexylprop-2-yn-1-ol was obtained as a yellow liquid (12 g, 88 mmol, 95%)

II) Crude 1-cyclohexylprop-2-yn-1-ol (step **I**) (14 g, 86 mmol, 1.0 equiv.), triethyl orthoacetate (15 g, 17 mL, 90 mmol, 1.05 equiv.), propionic acid (3 × 5 mol%). The crude product was purified by flash chromatography on silica gel (40:1pentane/Et_2O) to obtain the title compound (12 g, 54 mmol, 63%) as a colorless liquid.

Analytical Data

^{1}H-NMR (400.1 MHz, $CDCl_3$): δ = 1.02 – 1.23 (m, 4H), 1.24 – 1.27 (m, 4H), 1.61 (dddd, *J* = 10.9, 4.0, 3.0, 1.6 Hz, 1H), 1.67 – 1.75 (m, 4H), 1.97 (ddd, *J* = 11.0, 6.0, 3.1 Hz, 1H), 2.99 (dd, *J* = 7.2, 2.8 Hz, 2H), 4.14 (q, *J* = 7.1, 2H), 5.16 (ddd, *J* = 8.9, 5.9, 2.7 Hz, 1H), 5.24 (ddd, *J* = 7.2, 6.3, 3.0 Hz, 1H) ppm.

^{13}C-NMR (100.6 MHz, $CDCl_3$): δ = 14.3, 26.0, 26.0 , 26.2, 33.0, 33.0, 37.0, 60.7, 85.0, 98.2, 171.7, 204.0 ppm.

APCI-HRMS: *m/z* calcd for $C_{13}H_{12}O_2$ $[M+H]^+$ 200.0837 found 200.0838.

Synthesis of ethyl (E)-7-cyclohexylhepta-2,5,6-trienoat 119.

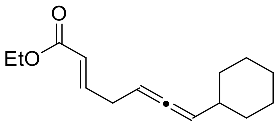

$C_{15}H_{22}O_2$
234.34

The reaction was performed according to **general procedure 6**.

I) ethyl 5-cyclohexylpenta-3,4-dienoate (12 g, 54 mmol, 1.0 equiv.) and DIBAL-H (78 mL, 78 mmol, 1.45 equiv., 1.0 M in DCM). The crude product was filtered over a short silica pad (DCM). 5-cyclohexylpenta-3,4-dienal was obtained as a yellow liquid (7.9 g, 49 mmol, 90%).

II) Crude 7-phenylhepta-3,4-dienal (step **I**) (7.9 g, 49 mmol), NaH (60% in mineral oil, 2.2 g, 54 mmol, 1.1 equiv.) in dry THF (200 mL) and triethyl phosphonoacetate (14.4 g, 12.7 mL, 64.0 mmol,1.3 equiv.). The crude product was purified by flash chromatography on silica gel (40:1 Pentane:Et_2O). The title compound was obtained as colorless liquid (6.8 g, 29 mmol, 59 %).

Analytical Data

^{1}H-NMR (400.1 MHz, CDCl$_3$): δ =1.03 – 1.24 (m, 4H), 1.29 (t, *J* = 7.1 Hz, 3H), 1.62 (ddtd, *J* = 10.8, 5.0, 2.4, 1.2 Hz, 1H), 1.67 – 1.78 (m, 5H), 1.92 – 2.03 (m, 1H), 2.88 (tdd, *J* = 6.5, 3.0, 1.7 Hz, 2H), 4.19 (q, *J* = 7.1 Hz, 2H), 5.08 – 5.20 (m, 2H), 5.87 (dt, *J*=15.6, 1.6, 1H), 6.97 (dtd, *J* = 15.7, 6.5, 0.3 Hz, 1H). ppm.

^{13}C-NMR (100.6 MHz, CDCl$_3$): δ = 14.4, 26.1, 26.1, 26.2, 32.1, 33.1, 33.1, 37.2, 60.3, 88.0, 98.3, 122.0, 146.9, 166.6, 203.8 ppm.

APCI-HRMS: *m/z* calcd for $C_{15}H_{23}O_2$ [M+H]$^+$ 235.1693 found 235.1692.

Synthesis of 7-cyclohexyl-3-(4-methoxyphenyl)hepta-5,6-dien-1-ol 120.

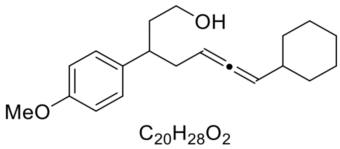

$C_{20}H_{28}O_2$
300.44

The reaction was performed according to **general procedure 7** with ethyl (E)-7-cyclohexylhepta-2,5,6-trienoat (0.8 g, 3.4 mmol, 1.0 equiv.) and (4-methoxyphenyl)magnesium bromide (5.1 mL, 5.1 mmol, 1.0 M). The crude product from step I was treated with LAH (0.19 mg, 5.1 mmol, 1.5 equiv.). The crude product from step II was purified by flash chromatography on silica gel (10:1 to 5:1 pentane/Et$_2$O). The desired product was obtained as a colorless liquid (0.62 g, 2.1 mmol, 61 %).

Analytical Data[3]

^{1}H-NMR (400.1 MHz, CDCl$_3$): δ = 0.83 – 1.08 (m, 2H), 1.13 – 1.32 (m, 4H), 1.54 – 1.72 (m, 5H), 1.74 – 1.93 (m, 2H), 1.93 – 2.11 (m, 1H), 2.21 – 2.39 (m, 2H), 2.70 – 2.85 (m, 1H), 3.43 – 3.60 (m, 2H), 3.79 (m$_c$, 3H), 4.90 – 5.03 (m, 2H), 6.81 – 6.87 (m, 2H), 7.07 – 7.12 (m, 2H) ppm.

^{13}C-NMR (100.6 MHz, CDCl$_3$): δ = 26.1, 26.1, 26.2, 26.3, 33.1, 33.1, 33.2, 37.0, 37.2, 37.3, 37.5, 38.9, 39.1, 41.8, 42.1, 55.3, 55.3, 61.3, 61.3, 89.7, 89.8, 96.8, 97.0, 113.9, 113.9, 128.7, 128.7, 158.2, 158.2, 203.4, 203.5 ppm.

APCI-HRMS: *m/z* calcd for $C_{20}H_{32}O_2N$ [M+NH$_4$]$^+$ 318.2433 found 318.2433.

Synthesis of 3-(4-methoxyphenyl)-9-phenylnona-5,6-dien-1-ol.

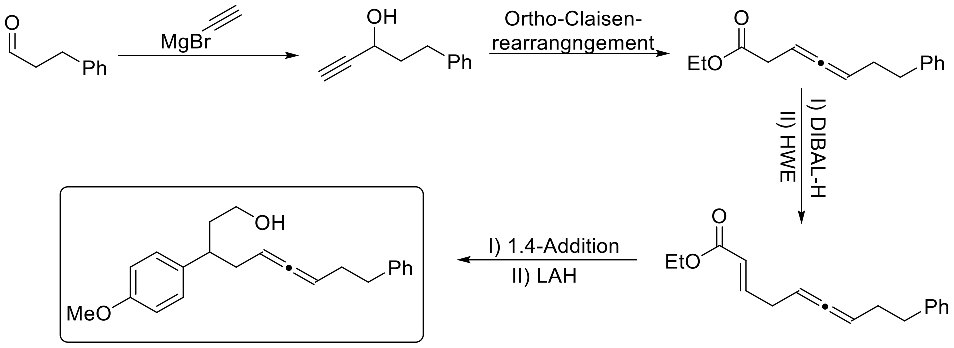

Synthesis of ethyl 7-phenylhepta-3,4-dienoate 121.

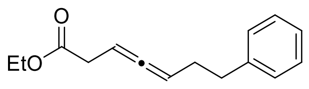

$C_{15}H_{18}O_2$
230.31

The reaction was performed according to **general procedure 5.**

I) 3-Phenylpropanal (12 g, 12 mL 93 mmol, 1.0 equiv.) and ethinyl magnesium bromide (200 mL, 100 mmol, 0.5 M in THF, 1.08 equiv.). The crude product was filtered over a short silica pad (2:1 pentane/Et_2O). 5-phenylpent-1-yn-3-ol was obtained as a yellow liquid (14 g, 86 mmol, 93%)

II) Crude phenylpent-1-yn-3-ol (step **I**) (14 g, 86 mmol, 1.0 equiv.), triethyl orthoacetate (15 g, 17 mL, 90 mmol, 1.05 equiv.), propionic acid (3 × 5 mol%). The crude product was purified by fractional distillation under reduced pressure (110 °C, 2 mbar) to obtain the title product (12 g, 54 mmol, 63%) as a colorless liquid.

Analytical Data

^{1}H-NMR (400.1 MHz, $CDCl_3$): δ = 1.27 (t, *J* = 7.1 Hz, 3H), 2.28 – 2.38 (m, 2H), 2.70 – 2.77 (m, 2H), 2.91 – 2.98 (m, 2H), 4.15 (q, *J* = 7.2 Hz, 2H), 5.18 – 5.28 (m, 2H), 7.17 – 7.20 (m, 3H), 7.26 – 7.31 (m, 2H) ppm.

^{13}C-NMR (100.6 MHz, $CDCl_3$): δ = 14.3, 30.2, 35.0, 35.3, 60.8, 84.8, 91.5, 125.9, 128.4, 128.6, 141.7, 1071.6, 205.3 ppm.

ESI-HRMS: *m/z* calcd for $C_{15}H_{19}O_2$ $[M+H]^+$ 231.1380 found 231.1383.

Synthesis of ethyl (E)-9-phenylnona-2,5,6-trienoate 122.

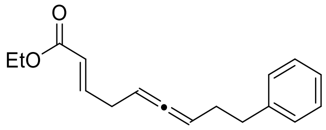

$C_{17}H_{20}O_2$
256.35

The reaction was performed according to **general procedure 6**.

I) Ethyl 7-phenylhepta-3,4-dienoate (12 g, 54 mmol, 1.0 equiv.) and DIBAL-H (78 mL, 78 mmol, 1.45 equiv., 1.0 M in DCM). The crude product was filtered over a short silica pad (DCM). Undeca-3,4-dienal was obtained as a yellow liquid (8.4 g, 50 mmol, 93%).

II) Crude 7-phenylhepta-3,4-dienal (step **I**) (8.4 g, 50 mmol), NaH (60% in mineral oil, 2.2 g, 55 mmol, 1.1 equiv.) in dry THF (200 mL) and triethyl phosphonoacetate (16 g, 14 mL, 72 mmol,1.3 equiv.). The crude product was purified by flash chromatography on silica gel (40:1 Pentane:Et_2O). The title compound was obtained as colorless liquid (11 g, 43 mmol, 79 %).

Analytical Data

^{1}H-NMR (400.1 MHz, $CDCl_3$): δ = 1.29 (t, *J* = 7.1 Hz, 3H), 2.29 – 2.36 (m, 2H), 2.72 (t, *J* = 7.7 Hz, 2H), 2.82 (ddd, *J* = 6.7, 2.8, 1.7 Hz, 2H), 4.19 (q, *J*=7.1, 2H), 5.04 – 5.14 (m, 1H), 5.17 – 5.28 (m, 1H), 5.86 (dd, *J* = 15.6, 1.7 Hz, 1H), 6.89 – 7.01 (m, 1H), 7.17 – 7.21 (m, 3H), 7.27 – 7.34 (m, 2H) ppm.

^{13}C-NMR (100.6 MHz, $CDCl_3$): δ = 14.4, 30.4, 31.9, 35.4, 60.3, 87.7, 91.5, 122.0, 125.9, 128.4, 128.6, 141.7, 146.7, 166.6, 205.1 ppm.

APCI-HRMS: *m/z* calcd for $C_{17}H_{24}O_2N$ $[M+NH_4]^+$ 274.1802 found 274.1801.

Synthesis of 3-(4-methoxyphenyl)-9-phenylnona-5,6-dien-1-ol 123.

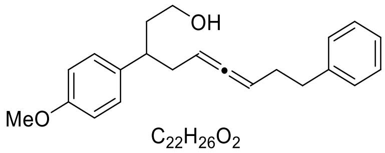

$C_{22}H_{26}O_2$
322.45

The reaction was performed according to **general procedure 7** with ethyl ethyl (E)-9-phenylnona-2,5,6-trienoate (0.8 g, 3.4 mmol, 1.0 equiv.) and (4-methoxyphenyl)magnesium bromide (5.1 mL, 5.1 mmol, 1.0 M). The crude product from step I was treated with LAH (0.19 mg, 5.1 mmol, 1.5 equiv.). The crude product from step II was purified by flash chromatography on silica gel (10:1 to 5:1 pentane/Et_2O). The desired product was obtained as a colorless liquid (0.64 g, 1.9 mmol, 58 %).

Analytical Data[3]

^{1}H-NMR (400.1 MHz, $CDCl_3$): δ = 1.76 (dddd, *J* = 13.9, 10.1, 6.3, 5.4 Hz, 1H), 1.98 (dddd, *J* = 13.8, 7.5, 6.9, 4.8 Hz, 1H), 2.12 – 2.20 (m, 1H), 2.21 – 2.30 (m, 3H), 2.56 (ddd, *J* = 7.9, 7.9, 2.4 Hz, 1H), 2.64 (dd,

J = 7.5, 7.5 Hz, 1H), 2.74 (dddd, *J* = 14.5, 9.8, 4.8, 2.2 Hz, 1H), 3.44 – 3.59 (m, 2H), 3.72 (s, 1H), 3.78 (s, 2H), 4.88 – 5.00 (m, 1H), 5.00 – 5.10 (m, 1H), 6.81 – 6.86 (m, 2H), 7.05 – 7.11 (m, 2H), 7.13 – 7.22 (m, 3H), 7.26 – 7.31 (m, 1H) ppm.

^{13}C-NMR (100.6 MHz, $CDCl_3$): δ = 30.5, 30.5, 35.4, 35.4, 36.6, 37.0, 38.9, 39.1, 41.7, 41.9, 55.2, 55.3, 61.2, 61.2, 89.4, 89.5, 90.1, 90.2, 113.9, 125.8 125.9, 128.3, 128.3, 128.6, 128.6, 128.7, 128.7, 136.4, 136.5, 142.0, 142.0, 158.1, 158.2, 204.8 ppm.

APCI-HRMS: *m/z* calcd for $C_{22}H_{30}O_2N$ $[M+NH_4]^+$ 340.2271 found 340.2272.

Synthesis of3-(4-methoxyphenyl)-8-phenylocta-5,6-dien-1-ol.

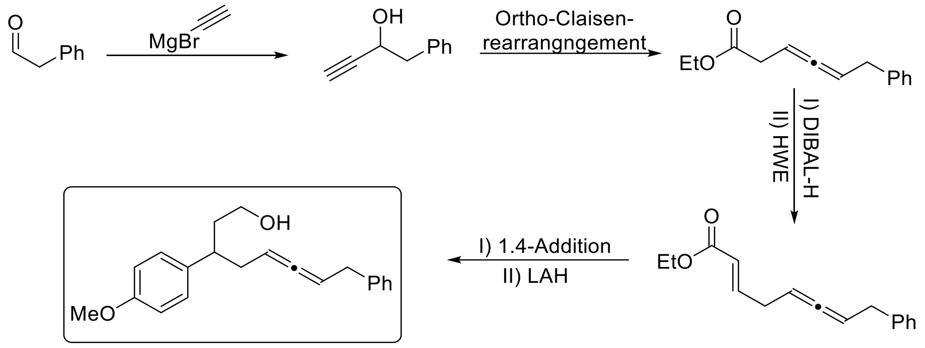

Synthesis of ethyl 6-phenylhexa-3,4-dienoate 124.

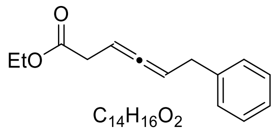

The reaction was performed according to **general procedure 5.**

I) 2-phenylacetaldehyde (g, 11.2 mL 93 mmol, 1.0 equiv.) and ethinyl magnesium bromide (200 mL, 100 mmol, 0.5 M in THF, 1.08 equiv.). The crude product was filtered over a short silica pad (2:1 pentane/Et_2O). 1-phenylbut-3-yn-2-ol was obtained as a yellow liquid (13 g, 89 mmol, 96%)

II) Crude 1-phenylbut-3-yn-2-ol (step **I**) (13 g, 89 mmol, 1.0 equiv.), triethyl orthoacetate (15 g, 17 mL, 90 mmol, 1.05 equiv.), propionic acid (3 × 5 mol%). The crude product was purified by fractional distillation under reduced pressure (110 °C, 2 mbar) to obtain the title compound (11 g, 61 mmol, 55%) as a colorless liquid.

Analytical Data

^{1}H-NMR (400.1 MHz, $CDCl_3$): δ = 1.27 (t, *J* = 7.1 Hz, 3H), 3.03 (dd, *J* = 7.2, 2.7 Hz, 2H), 3.36 (dd, *J* = 7.2, 2.8 Hz, 2H), 4.15 (q, *J* = 7.1 Hz, 2H), 5.25 – 5.33 (m, 1H), 5.33 – 5.40 (m, 1H), 7.20 – 7.24 (m, 3H), 7.27 – 7.32 (m, 2H). ppm.

^{13}C-NMR (100.6 MHz, $CDCl_3$): δ = 14.3, 35.0, 35.4, 60.8, 84.8, 91.7, 126.3, 128.5, 128.5, 140.2, 171.5, 205.6 ppm.

APCI-HRMS: *m/z* calcd for $C_{14}H_{20}O_2N$ $[M+NH_4]^+$ 234.1489 found 234.1488.

Synthesis of ethyl (E)-8-phenylocta-2,5,6-trienoate 125.

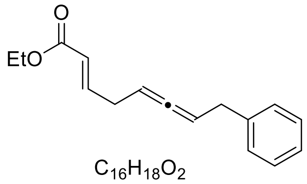

$C_{16}H_{18}O_2$
242.32

The reaction was performed according to **general procedure 6**.

I) Ethyl 6-phenylhexa-3,4-dienoate (11 g, 61 mmol, 1.0 equiv.) and DIBAL-H (89 mL, 89 mmol, 1.45 equiv., 1.0 M in DCM). The crude product was filtered over a short silica pad (DCM). 6-phenylhexa-3,4-dienal was obtained as a yellow liquid (9.5 g, 55 mmol, 90%).

II) Crude 6 phenylhexa-3,4-dienal (step **I**) (9.5 g, 55 mmol), NaH (60% in mineral oil, 2.4 g, 61 mmol, 1.1 equiv.) in dry THF (150 mL) and triethyl phosphonoacetate (16 g, 14 mL, 72 mmol,1.3 equiv.). The crude product was purified by flash chromatography on silica gel (40:1 Pentane:Et_2O). The title compound was obtained as colorless liquid (5.9 g, 28 mmol, 50 %).

Analytical Data10

^{1}H-NMR (400.1 MHz, $CDCl_3$): δ = 1.29 (t, *J* = 7.1 Hz, 3H), 2.87 (ddd, *J* = 6.7, 2.8, 1.7 Hz, 2H), 3.03 (dd, *J* = 7.2, 2.7 Hr, 1H), 3.35 (td, *J* = 6.6, 2.8 Hz, 2H), 4.18 – 4.22 (m, 2H), 5.10 – 5.17 (m, 1H), 5.33 – 5.36 (m, 1H), 5.84 (dt, *J* = 15.6, 1.7 Hz, 1H), 6.94 (dtd, *J* = 15.6, 6.5 Hz, 0.3, 1H), 7.22 – 7.24 (m, 2H), 7.27 – 7.29 (m, 2H) ppm.

^{13}C-NMR (100.6 MHz, $CDCl_3$): δ = 14.4, 31.8, 35.0, 35.6, 60.3, 60.8, 84.8, 87.9, 91.8, 122.2, 126.3, 128.6, 146.4, 205.4 ppm.

APCI-HRMS: *m/z* calcd for $C_{16}H_{22}O_2N$ $[M+NH_4]^+$ 260.1651 found 260.1652.

Synthesis of 3-(4-methoxyphenyl)-8-phenylocta-5,6-dien-1-ol 126.

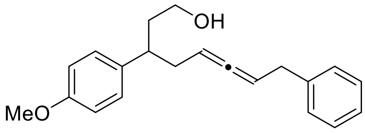

$C_{21}H_{24}O_2$
308.42

The reaction was performed according to **general procedure 7** with ethyl ethyl (E)-8-phenylocta-2,5,6-trienoate (0.82 g, 3.4 mmol, 1.0 equiv.) and (4-methoxyphenyl)magnesium bromide (5.1 mL, 5.1 mmol, 1.0 M). The crude product from step I was treated with LAH (0.19 mg, 5.1 mmol, 1.5 equiv.). The crude product from step II was purified by flash chromatography on silica gel (10:1 to 5:1 pentane/Et_2O). The desired product was obtained as a colorless liquid (0.55 g, 1.8 mmol, 52 %).

Analytical Data[3]

^{1}H-NMR (400.1 MHz, $CDCl_3$): δ = 1.22 (s, 1H), 1.67 – 1.82 (m, 1H), 1.88 – 2.02 (m, 1H), 2.25 – 2.34 (m, 2H), 2.73 (dddd, *J* = 22.1, 10.0, 7.3, 6.8, 6.8, 4.8 Hz, 1H), 3.19 (ddd, *J* = 7.5, 2.0, 2.0 Hz, 1H), 3.27 (dd, *J* = 7.0, 2.9 Hz, 1H), 3.41 – 3.57 (m, 2H), 3.77 (m_c, 3H), 4.98 (dddd, *J* = 8.4, 7.4, 5.6, 3.9 Hz, 1H), 5.14 – 5.25 (m, 1H), 6.82 – 6.84 (m, 2H), 7.05 – 7.09 (m, 2H), 7.16 – 7.22 (m, 3H), 7.20 -7.30 (m, 2H) ppm.
^{13}C-NMR (100.6 MHz, $CDCl_3$): δ = 35.7, 35.7, 36.8, 36.9, 38.8, 38.8, 41.7, 41.8, 55.3, 61.2, 61.2, 89.4, 89.6, 90.2, 90.3, 113.9, 114.0, 126.1, 128.4, 128.5, 128.6, 128.6, 136.4, 136.5, 158.2, 205.3, 205.3 ppm.
APCI-HRMS: *m/z* calcd for $C_{21}H_{28}O_2N$ $[M+NH_4]^+$ 326.2115 found 326.2116.

Synthesis of 3-(4-methoxyphenyl)-10-(methylthio)deca-5,6-dien-1-ol

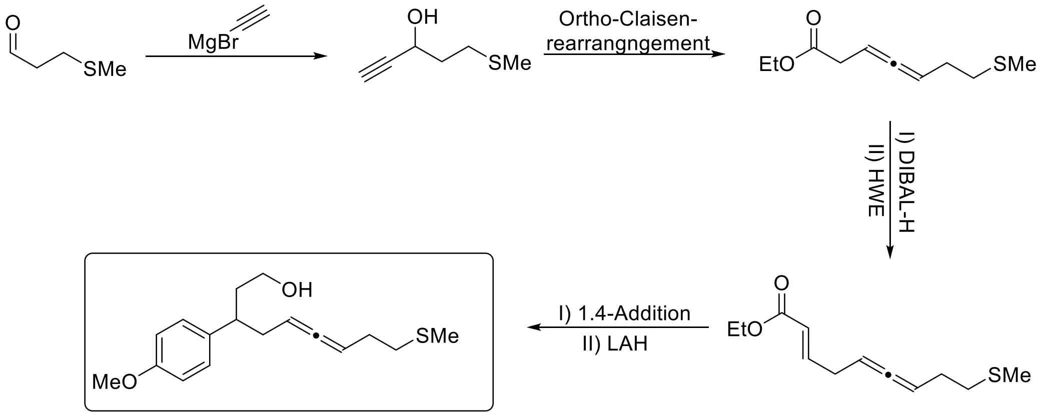

Synthesis of ethyl 7-(methylthio)hepta-3,4-dienoate 127.

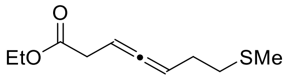

$C_{10}H_{16}O_2S$
200.30

The reaction was performed according to **general procedure 5.**

I) 3-(methylthio)propanal (10 g, 85 mmol, 1.0 equiv.) and ethinyl magnesium bromide (180 mL, 90 mmol, 0.5 M in THF, 1.08 equiv.). The crude product was filtered over a short silica pad (2:1 pentane/Et_2O). 5-(methylthio)pent-1-yn-3-ol was obtained as a yellow liquid (12 g, 79 mmol, 93%)

II) Crude 5-(methylthio)pent-1-yn-3-ol (step **I**) (11 g, 87 mmol 1.0 equiv.), triethyl orthoacetate (15 g, 17 mL, 91 mmol, 1.05 equiv.), propionic acid (3 × 5 mol%). The crude product was purified by flash chromatography on silica gel (40:1pentane/Et_2O) to obtain the title compound (11 g, 52 mmol, 60%) as a colorless liquid.

Analytical Data

^{1}H-NMR (400.1 MHz, CDCl$_3$): δ = 1.27 (t, *J* = 7.1 Hz, 3H), 2.11 (s, 3H), 2.26 – 2.33 (m, 2H), 2.55 – 2.59 (m, 2H), 3.03 (dd, *J* = 7.1, 2.9 Hz, 2H), 4.16 (q, *J* = 7.2 Hz, 2H), 5.21 – 5.33 (m, 2H) ppm.

^{13}C-NMR (100.6 MHz, CDCl$_3$): δ = 14.3, 15.6, 28.4, 33.6, 34.9, 60.8, 85.2, 90.6, 171.5, 205.3 ppm.

APCI-HRMS: *m/z* calcd for $C_{10}H_{17}O_2S$ $[M+H]^+$ 201.0944 found 201.0945.

Synthesis of ethyl (E)-9-(methylthio)nona-2,5,6-trienoate 128.

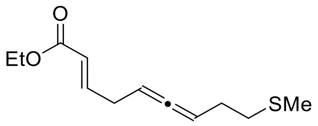

$C_{12}H_{18}O_2S$
226.33

The reaction was performed according to **general procedure 6**.

I) ethyl 7-(methylthio)hepta-3,4-dienoate (11 g, 52 mmol, 1.0 equiv.) and DIBAL-H (75 mL, 75 mmol, 1.45 equiv., 1.0 M in DCM). The crude product was filtered over a short silica pad (DCM). 7-(methylthio)hepta-3,4-dienal was obtained as a yellow liquid (7.3 g, 43 mmol, 83%).

II) Crude 7-(methylthio)hepta-3,4-dienal (step **I**) (7.3 g, 48 mmol), NaH (60% in mineral oil, 2.1 g, 53 mmol, 1.1 equiv.) in dry THF (200 mL) and triethyl phosphonoacetate (15 g, 13 mL, 69 mmol,1.3 equiv.). The crude product was purified by flash chromatography on silica gel (40:1 Pentane:Et_2O). The title compound was obtained as colorless liquid (7.9 g, 33 mmol, 62 %).

Analytical Data10

^{1}H-NMR (400.1 MHz, CDCl$_3$): δ = 1.28 (t, *J* = 7.1 Hz, 3H), 2.11 (s, 3H), 2.25 – 2.34 (m, 2H), 2.53 – 2.59 (m, 2iH), 2.90 (tdd, *J* = 6.6, 2.8, 1.7 Hz, 2H), 4.16 – 4.22 (m, 2H), 5.11 – 5.18 (m, 1H), 5.22 (qt, *J* = 6.5, 2.8 Hz, 1H), 5.88 (dt, *J* = 15.6, 1.7 Hz, 1H), 6.96 (dtd, *J* = 15.7, 6.5, 0.3 Hz, 1H).ppm.

^{13}C-NMR (100.6 MHz, CDCl$_3$): δ = 14.3, 15.6, 28.6, 31.8, 33.7, 60.3, 88.1, 90.7, 122.1, 146.5, 166.5, 205.1 ppm.

APCI-HRMS: *m/z* calcd for $C_{12}H_{19}O_3S$ $[M+OH]^-$ 243.1049 found 243.1047.

Synthesis of 3-(4-methoxyphenyl)-9-(methylthio)nona-5,6-dien-1-ol 129.

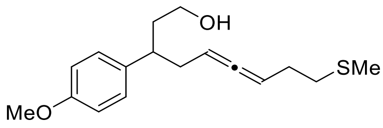

$C_{17}H_{24}O_2S$
292.44

The reaction was performed according to **general procedure 7** with ethyl (E)-10-(methylthio)deca-2,5,6-trienoate. (0.82 g, 3.4 mmol, 1.0 equiv.) and (4-methoxyphenyl)magnesium bromide (5.1 mL, 5.1 mmol, 1.0 M). The crude product from step I was treated with LAH (0.19 mg, 5.1 mmol, 1.5 equiv.). The crude product from step II was purified by flash chromatography on silica gel (10:1 to 5:1 pentane/Et_2O). The desired product was obtained as a colorless liquid (0.68 g, 2.2 mmol, 65%).

Analytical Data[3]

^{1}H-NMR (400.1 MHz, $CDCl_3$): δ = 1.27 (s, 1H), 1.78 (dddt, *J* = 13.7, 10.0, 6.3, 5.5 Hz, 1H), 1.94 – 2.05 (m, 1H), 2.08 (m_c, 3H), 2.11 – 2.17 (m, 1H), 2.17 – 2.23 (m, 1H), 2.28 – 2.34 (m, 2H), 2.36 – 2.42 (m, 1H), 2.43 – 2.49 (m, 1H), 2.73 – 2.86 (m, 1H), 3.48 (dddd, *J* = 10.6, 7.6, 6.3, 2.2 Hz, 1H), 3.56 (dddd, *J* = 10.6, 6.8, 5.4, 2.3 Hz, 1H), 3.79 (m_c,, 3H), 4.92 – 5.11 (m, 2H), 6.81 – 6.87 (m, 2H), 7.07 – 7.12 (m, 2H) ppm.

^{13}C-NMR (100.6 MHz, $CDCl_3$): δ = 15.5, 15.6, 28.7, 28.7, 33.6, 33.7, 36.4, 37.0, 39.0, 39.1, 41.5, 41.9, 55.3, 61.2, 61.2, 89.2, 89.4, 89.8, 89.9, 113.9, 128.6, 128.7, 136.4, 204.8 ppm.

APCI-HRMS: *m/z* calcd for $C_{17}H_{25}O_2S$ $[M+H]^+$ 293.1575 found 293.1576.

2.3 Synthesis and characterization of 1,3-substituted allenols

Synthesis of syn and anti - 4-(4-methoxyphenyl)octa-6,7-dien-2-ol 45, 49

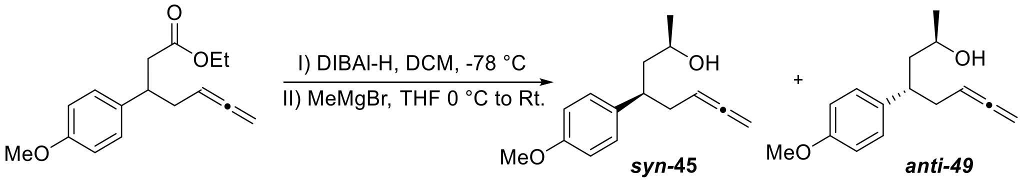

$C_{16}H_{20}O_3$
260.33

$C_{15}H_{20}O_2$
232.32

I) At −80°C a solution of DIBAL-H (5.3 mL, 5.3 mmol, 1.4 equiv., 1.0 M in CH_2Cl_2) was added dropwise over 90 min to a solution of ethyl 3-(4-methoxyphenyl)hepta-5,6-dienoate (1.0 g, 3.8 mmol, 1.0 equiv.) in CH_2Cl_2 (5 mL). The reaction mixture was stirred for 1 h and then transferred to an ice-cold aqueous solution of HCl (2.0 M, 50 mL). The layers were separated, the organic layer was washed with HCl (2.0 M, 2 × 10 mL) and the aqueous layer was extracted with CH_2Cl_2 (2 × 20 mL). The combined organic layers were washed with brine, dried over Na_2SO_4, filtered over a silica pad (DCM) and concentrated under

reduced pressure. The crude 7 3-(4-methoxyphenyl)hepta-5,6-dienal was obtained as a yellow liquid (0.78 g, 3.6 mmol, 95%).

II) A solution of MeMgBr (1.8 mL, 5.4 mmol, 1.5 equiv, 3 M in Et_2O) in THF (5 mL) was cooled to 0 °C and the crude 7 3-(4-methoxyphenyl)hepta-5,6-dienal (0.78 g, 3.6 mmol) in THF (2 mL) was added dropwise. The Mixture was stirred for 30 minutes and then warmed to room temperature. Saturated ammonium chloride (10 mL) was added and the aqueous layer was separated and extracted with Et_2O (2 × 20 mL). The combined organic layers were dried over Na_2SO_4, the solvent was removed under reduced pressure and the two Diastereomers were separated by flash Chromatography (SiO2, pentane/Et2O = 4/1). The *syn*-diastereomer was obtained as a single diastereomer (0.40 g, 1.7 mmol, 48 %). The *anti* diastereomer was obtained (syn/anti = 25/75, 0.20 g, 0,86 mmol, 24 %) as colorless liquid.

Analytical Data (*syn*-product) syn-45[4]

^{1}H-NMR (400.1 MHz, $CDCl_3$): δ = 1.12 (d, *J* = 6.2 Hz, 3H), 1.31 (s, 1H), 1.64 (ddd, *J* = 14.0, 11.1, 3.1 Hz, 1H), 1.80 (ddd, *J* = 13.8, 9.6, 4.1 Hz, 1H), 2.28 (dddd, *J* = 7.7, 7.3, 3.0, 2.8 Hz, 2H), 2.89 (dddd, *J* = 11.4, 7.3, 7.2, 3.7 Hz, 1H), 3.53 (m_c, 1H), 3.79 (s, 3H), 4.53 – 4.64 (m, 2H), 4.90 – 4.98 (m, 1H), 6.82 – 6.87 (m, 2H), 7.08 – 7.13 (m, 2H) ppm.

^{13}C-NMR (100.6 MHz, $CDCl_3$): δ = 24.5, 36.6, 41.7, 45.5, 55.3, 65.7, 74.4, 88.2, 113.9, 128.8, 136.5, 158.2, 209.2 ppm.

APCI-HRMS: *m/z* calcd for $C_{16}H_{22}O_2$ $[M+H]^+$233.1534 found 233.1536.

Analytical Data (*anti*-product) anti-49[5]

^{1}H-NMR (400.1 MHz, $CDCl_3$): δ = 1.17 (d, *J* = 6.2 Hz, 3H), 1.28 (s, 1H), 1.78 – 1.85 (m, 1H), 2.21 – 2.39 (m, 2H), 2.72 (dddd, *J* = 8.2, 8.1, 5.7, 5.5 Hz, 1H), 3.44 – 3.61 (m, 1H), 3.71 (m_c, 1H), 3.79 (s, 3H), 4.53 – 4.64 (m, 2H), 4.88 – 4.98 (m, 1H), 6.82 – 6.87 (m, 2H), 7.08 – 7.13 (m, 2H) ppm.

^{13}C-NMR (100.6 MHz, $CDCl_3$): δ = 23.4, 36.3, 42.5, 45.6, 55.3, 66.8, 74.5, 88.0, 114.1, 128.6, 136.5, 158.3, 209.1 ppm.

APCI-HRMS: *m/z* calcd for $C_{16}H_{22}O$ $[M+H]^+$265.1692 found 265.1693.

Synthesis of syn and anti - 4-(4-methoxyphenyl)octa-6,7-dien-2-ol 46, 50

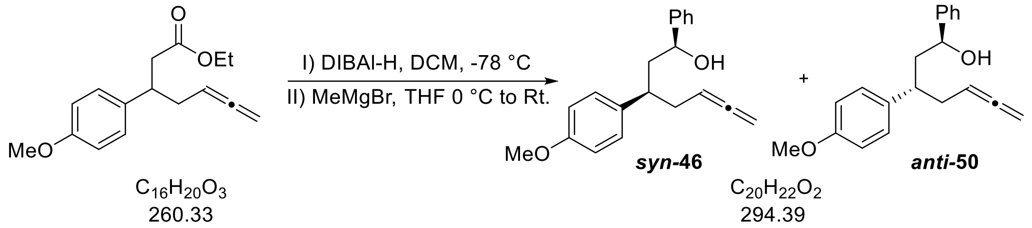

I) At −80°C a solution of DIBAL-H (5.3 mL, 5.3 mmol, 1.4 equiv., 1.0 M in CH_2Cl_2) was added dropwise over 90 min to a solution of ethyl 3-(4-methoxyphenyl)hepta-5,6-dienoate (1.0 g, 3.8 mmol, 1.0 equiv.) in CH_2Cl_2 (5 mL). The reaction mixture was stirred for 1 h and then transferred to an ice-cold aqueous solution of HCl (2.0 M, 50 mL). The layers were separated, the organic layer was washed with HCl (2.0 M, 2 × 10 mL) and the aqueous layer was extracted with CH_2Cl_2 (2 × 20 mL). The combined organic layers were washed with brine, dried over Na_2SO_4, filtered over a silica pad (DCM) and concentrated under reduced pressure. The crude 7 3-(4-methoxyphenyl)hepta-5,6-dienal was obtained as a yellow liquid (0.78 g, 3.6 mmol, 96%).

II) A solution of PhMgBr (5.4 mL, 5.4 mmol, 1.5 equiv, 1 M in THF) in THF (5 mL) was cooled to 0 °C and the crude 7 3-(4-methoxyphenyl)hepta-5,6-dienal (0.78 g, 3.6 mmol) in THF (2 mL) was added dropwise. The Mixture was stirred for 30 minutes and then warmed to room temperature. Saturated ammonium chloride (10 mL) was added and the aqueous layer was separated and extracted with Et_2O (2 × 20 mL). The combined organic layers were dried over Na_2SO_4, the solvent was removed under reduced pressure and the two Diastereomers were separated by flash Chromatography (SiO2, pentane/Et2O = 4/1). The *syn*-diastereomer was obtained as a single diastereomer (0.39 g, 1.3 mmol, 37 %). The *anti* diastereomer was obtained in a single diastereomer (0.21 g, 0,71 mmol, 20 %).

Analytical Data (*syn*-product) syn-46[4]

^{1}H-NMR (400.1 MHz, $CDCl_3$): δ = 1.68 (s, 1H), 1.87 (ddd, *J* = 14.1, 11.1, 2.9 Hz, 1H), 2.14 (ddd, *J* = 14.2, 10.2, 4.1 Hz, 1H), 2.27 – 2.39 (m, 2H), 3.03 (dddd, *J* = 11.3, 7.4, 7.3, 4.0 Hz, 1H), 3.82 (s, 3H), 4.39 (dd, *J* = 10.2, 2.9 Hz, 1H), 4.53 – 4.63 (m, 2H), 4.91 – 5.00 (m, 1H), 6.86 – 6.91 (m, 2H), 7.14 – 7.19 (m, 2H), 7.22 – 7.26 (m, 3H), 7.28 – 7.34 (m, 2H) ppm.

^{13}C-NMR (100.6 MHz, $CDCl_3$): δ = 36.6, 41.8, 45.7, 55.3, 72.0, 74.4, 88.1, 114.0, 125.6, 127.4, 128.5, 128.9, 136.2, 145.4, 158.2, 209.2 ppm.

APCI-HRMS: *m/z* calcd for $C_{16}H_{22}O_2$ $[M+H]^+$295.1692 found 295.1693.

Analytical Data (*anti*-product) anti-50[4]

^{1}H-NMR (400.1 MHz, $CDCl_3$): δ = 1.71 (s, 1H), 2.15 (ddd, *J* = 7.2, 6.7, 1.4 Hz, 2H), 2.21 – 2.35 (m, 2H), 2.54 (dddd, *J* = 8.0, 7.9, 6.5, 6.1 Hz, 1H), 3.81 (s, 3H), 4.50 – 4.60 (m, 3H), 4.86 (m_c, 1H), 6.85 – 6.89 (m, 2H), 7.06 – 7.12 (m, 2H), 7.26 – 7.31 (m, 3H), 7.32 – 7.38 (m, 2H) ppm.

^{13}C-NMR (100.6 MHz, CDCl$_3$): δ = 36.4, 41.8, 44.8, 55.3, 73.2, 74.4, 87.8, 114.0, 126.4, 127.8, 128.6, 128.8, 136.4, 144.3, 158.2, 209.1 ppm.

APCI-HRMS: *m/z* calcd for $C_{16}H_{22}O_2$ [M+H]$^+$295.1692 found 295.1693.

Synthesis of syn and anti - 4-(4-methoxyphenyl)tetradeca-6,7-dien-2-ol 47, 51

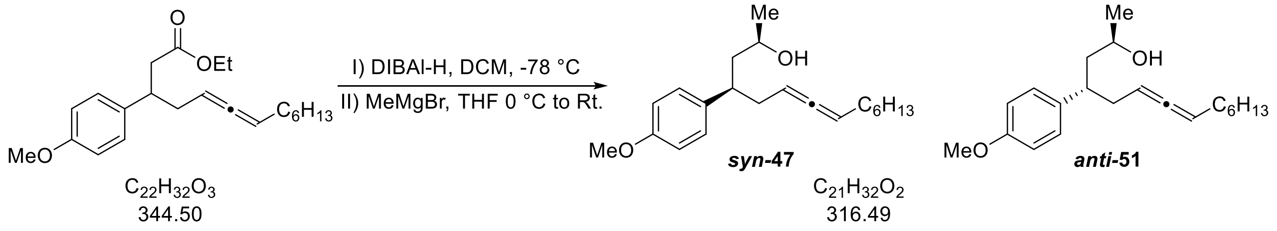

I) At −80°C a solution of DIBAL-H (4.2 mL, 4.2 mmol, 1.4 equiv., 1.0 M in CH_2Cl_2) was added dropwise over 90 min to a solution of ethyl 3-(4-methoxyphenyl)trideca-5,6-dienoate (1.0 g, 2.9 mmol, 1.0 equiv.) in CH_2Cl_2 (5 mL). The reaction mixture was stirred for 1 h and then transferred to an ice-cold aqueous solution of HCl (2.0 M, 50 mL). The layers were separated, the organic layer was washed with HCl (2.0 M, 2 × 10 mL) and the aqueous layer was extracted with CH_2Cl_2 (2 × 20 mL). The combined organic layers were washed with brine, dried over Na_2SO_4, filtered over a silica pad (DCM) and concentrated under reduced pressure. The crude 3-(4-methoxyphenyl)trideca-5,6-dienal was obtained as a yellow liquid (0.85 g, 2.8 mmol, 98%).

II) A solution of MeMgBr (1.4 mL, 4.2 mmol, 1.5 equiv, 3 M in Et_2O) in THF (5 mL) was cooled to 0 °C and the crude 3-(4-methoxyphenyl)trideca-5,6-dienal (0.85 g, 2.8 mmol) in THF (2 mL) was added dropwise. The Mixture was stirred for 30 minutes and then warmed to room temperature. Saturated ammonium chloride (10 mL) was added and the aqueous layer was separated and extracted with Et_2O (2 × 20 mL). The combined organic layers were dried over Na_2SO_4, the solvent was removed under reduced pressure and the two Diastereomers were separated by flash Chromatography (SiO2, pentane/Et2O = 4/1). The *syn*-diastereomer was obtained in (0.39 g, 1.2 mmol, 41 %) yield. The *anti* diastereomer was obtained in (0.25 g, 0.79 mmol, 33 %) yield.

Analytical Data (*syn*-product) syn-47

^{1}H-NMR (400.1 MHz, CDCl$_3$): δ = 0.88 (t, *J* = 7.1 Hz, 3H), 1.11 (dd, *J* = 6.2, 1.0 Hz, 3H), 1.22 – 1.38 (m, 9H), 1.64 (dddd, *J* = 14.0, 11.0, 7.7, 3.1 Hz, 1H), 1.81 (dddd, *J* = 13.7, 9.5, 9.4 4.2 Hz, 2H), 1.86 – 1.97 (m, 1H), 2.20 – 2.31 (m, 2H), 2.88 (ddddd, *J* = 10.8, 7.5, 7.4, 3.8, 3.5 Hz, 1H), 3.53 (ddddd, *J* = 9.4, 6.2, 6.0, 3.4, 3.1, 2.0 Hz, 1H), 3.79 (s, 3H), 4.86 – 4.95 (m, 1H), 4.95 – 5.04 (m, 1H), 6.81 – 6.87 (m, 2H), 7.07 – 7.14 (m, 2H) ppm.

^{13}C-NMR (100.6 MHz, CDCl$_3$): δ = 14.2, 22.7, 24.4, 28.9, 28.9, 29.0, 29.2, 31.8, 31.8, 37.3, 37.5, 41.7, 41.8, 45.6, 45.7, 55.3, 65.7, 65.8, 88.9, 89.0, 90.7, 113.9, 128.8, 136.7, 158.1, 204.7 ppm.

ESI-HRMS: *m/z* calcd for $C_{21}H_{33}O$ [M+H]$^+$ 317.2475 found 317.2476.

Analytical Data (*anti*-product) anti-51

^{1}H-NMR (400.1 MHz, $CDCl_3$): δ = 0.90 (t, *J* = 6.1 Hz 3H), 1.16 (d, *J* = 6.2 Hz, 2H), 1.24 – 1.34 (m, 9H), 1.75 – 1.89 (m, 3H), 1.91 (dddd, *J* = 5.5, 5.3, 4.4, 3.7, 2.4 Hz, 1H), 2.18 – 2.36 (m, 2H), 2.67 – 2.81 (m, 1H), 3.44 – 3.62 (m, 1H), 3.71 (m_c, 1H), 3.79 (m_c, 3H), 4.84 – 4.95 (m, 1H), 4.95 – 5.05 (m, 1H), 6.82 – 6.89 (m, 2H), 7.07 – 7.14 (m, 2H) ppm.

^{13}C-NMR (100.6 MHz, $CDCl_3$): δ = 14.2, 22.7, 23.4, 28.9, 28.9, 29.2, 29.3, 31.8, 31.8, 37.0, 37.3, 42.6, 42.7, 45.7, 45.7, 55.3, 61.3, 66.9, 66.9, 88.7, 88.8, 90.8, 90.9, 91.0, 113.9, 114.0, 128.6, 128.6, 128.7, 136.8, 158.2, 204.6 ppm.

ESI-HRMS: *m/z* calcd for $C_{21}H_{33}O$ $[M+H]^+$ 317.2475 found 317.2476.

Synthesis of syn and anti - 3-(4-methoxyphenyl)-1-phenyltrideca-5,6-dien-1-ol 48,52

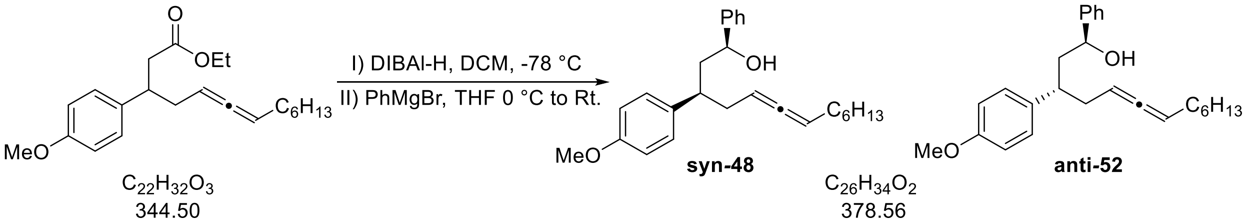

I) At −80°C a solution of DIBAL-H (4.2 mL, 4.2 mmol, 1.4 equiv., 1.0 M in CH_2Cl_2) was added dropwise over 90 min to a solution of ethyl 3-(4-methoxyphenyl)trideca-5,6-dienoate (1.0 g, 2.9 mmol, 1.0 equiv.) in CH_2Cl_2 (5 mL). The reaction mixture was stirred for 1 h and then transferred to an ice-cold aqueous solution of HCl (2.0 M, 50 mL). The layers were separated, the organic layer was washed with HCl (2.0 M, 2 × 10 mL) and the aqueous layer was extracted with CH_2Cl_2 (2 × 20 mL). The combined organic layers were washed with brine, dried over Na_2SO_4, filtered over a silica pad (DCM) and concentrated under reduced pressure. The crude 3-(4-methoxyphenyl)trideca-5,6-dienal was obtained as a yellow liquid (0.85 g, 2.8 mmol, 98%).

II) A solution of PhMgBr (4.2 mL, 4.2 mmol, 1.5 equiv, 1 M in THF) in THF (5 mL) was cooled to 0 °C and the crude 3-(4-methoxyphenyl)trideca-5,6-dienal (0.85 g, 2.8 mmol) in THF (2 mL) was added dropwise. The Mixture was stirred for 30 minutes and then warmed to room temperature. Saturated ammonium chloride (10 mL) was added and the aqueous layer was separated and extracted with Et_2O (2 × 20 mL). The combined organic layers were dried over Na_2SO_4, the solvent was removed under reduced pressure and the two Diastereomers were separated by flash Chromatography (SiO2, pentane/$E_{t2}O$ = 4/1). The *syn*-diastereomer was obtained in (0.42 g, 1.1 mmol, 40%) yiled. The *anti* diastereomer was obtained in (0.29 g, 0.77 mmol, 28%) yield.

Analytical Data (*syn*-product) syn-48

^{1}H-NMR (400.1 MHz, $CDCl_3$): δ = 0.90 (t, *J* = 6.0 Hz 3H), 1.16 – 1.38 (m, 9H), 1.56 (s, 1H), 1.80 – 1.94 (m, 3H), 2.15 (dddd, *J* = 14.3, 10.3, 10.0, 4.1 Hz, 1H), 2.29 (dddd, *J* = 9.5, 4.8, 3.4, 2.0 Hz, 2H), 2.95 – 3.08 (m, 1H), 3.81 (m, 3H), 4.40 (dt, *J* = 10.2, 2.5 Hz, 1H), 4.87 – 4.95 (m, 1H), 4.99 (m_c, 1H), 6.86 – 6.89 (m, 2H), 7.12 – 7.19 (m, 2H), 7.21 – 7.26 (m, 3H), 7.27 – 7.33 (m, 2H) ppm.

^{13}C-NMR (100.6 MHz, $CDCl_3$): δ = 14.2, 22.7, 28.9, 28.9, 28.9, 29.0, 29.2, 29.2, 31.8, 31.8, 37.3, 37.5, 41.8, 41.9, 45.7, 45.8, 55.3, 55.3, 72.0, 88.8, 88.9, 90.8, 90.8, 114.0, 115.4, 125.6, 125.6, 127.4, 128.5 128.9, 128.9, 145.4, 204.7 ppm.

APCI-HRMS: *m/z* calcd for $C_{26}H_{38}ON$ $[M+NH_{4]}]^+$ 396.2897 found 396.2898.

Analytical Data (*anti*-product) anti-52

^{1}H-NMR (400.1 MHz, $CDCl_3$): δ = 0.82 – 0.93 (m, 3H), 1.23 – 1.29 (m, 8H), 1.66 (s, 1H), 1.80 (m_c, 1H), 1.88 (m_c, 1H), 2.09 – 2.19 (m, 2H), 2.19 – 2.37 (m, 2H), 2.53 (dddd, *J* = 14.6, 12.6, 8.5, 6.4 Hz, 1H), 3.81 (s, 3H), 4.53 (m_c, 1H), 4.76 – 4.88 (m, 1H), 4.95 (dddd, *J* = 13.5, 9.2, 6.6, 3.4 Hz, 1H), 6.84 – 6.89 (m, 2H), 7.05 – 7.11 (m, 2H), 7.26 – 7.31 (m, 3H), 7.31 – 7.37 (m, 2H) ppm.

^{13}C-NMR (100.6 MHz, $CDCl_3$): δ = 14.2, 22.7, 28.9, 28.9, 29.2, 29.2, 31.7, 37.1, 37.5, 41.9, 42.2, 44.8, 45.0, 55.3, 73.3, 88.6, 90.7, 90.9, 114.0, 126.4, 126.4, 127.8, 127.8, 128.6, 128.8, 128.8, 144.4, 158.2, 204.7 ppm.

APCI-HRMS: *m/z* calcd for $C_{26}H_{38}ON$ $[M+NH_{4]}]^+$ 396.2897 found 396.2898.

3 Experimental Procedures Catalysis

3.1 General Procedure catalysis

General Procedure 8: Rh-catalyzed cyclisation of terminal allenols:

A 10 mL screw-cap flask was flame-dried, cooled to room temperature under vacuum and backfilled with argon (Argon 5.0 Sauerstoffwerk Friedrichshafen) using a standard SCHLENK line apparatus. The screw-cap flask was charged with the corresponding δ-hydroxy allene (0.3 mmol, 1.0 equiv.), evacuated for 15 and then backfilled with argon three times. Then $[Rh(COD)Cl]_2$ (3.7 mg, 0.0075 mmol, 2.5 mol%), dppf (8.3 mg, 0.015 mmol, 5.0 mol%) and diphenyl phosphate (12.1 mg, 0.06 mmol, 20 mol%) was added under a flow of argon followed by freshly distilled DCM (0.3 M). The flask was sealed and stirred at 80 °C overnight. The reaction mixture was filtered over silica and concentrated under reduced pressure. The residue was analyzed by ^{1}H-NMR spectroscopy. If desired, the crude product was purified by flash chromatography on silica gel using a mixture of pentane/ether.

General Procedure 9: Rh-catalyzed cyclisation of internal allenols:

A 10 mL screw-cap flask was flame-dried, cooled to room temperature under vacuum and backfilled with argon (Argon 5.0 Sauerstoffwerk Friedrichshafen) using a standard SCHLENK line apparatus. The screw-cap flask was charged with the corresponding internal δ-hydroxy allene (0.3 mmol, 1.0 equiv.), evacuated for 15 and then backfilled with argon three times. Then $[Rh(COD)Cl]_2$ (3.7 mg, 0.0075 mmol, 2.5 mol%), L5 (12.6 mg, 0.015 mmol, 5.0 mol%) and PTSA (18 mg, 0.09 mmol, 30 mol%) was added under a flow of argon followed by freshly distilled PhF (1.0 mL, 0.3 M). The flask was sealed and stirred at 80 °C overnight. The reaction mixture was filtered over silica and concentrated under reduced pressure. The residue was analyzed by ^{1}H-NMR spectroscopy. If desired, the crude product was purified by flash chromatography on silica gel using a mixture of pentane/ether.

General procedure for the determination d.r. – ratio: Catalysis terminal allene

To determine the syn/anti ratio the crude ^{1}H-NMR was measured in $CDCl_3$

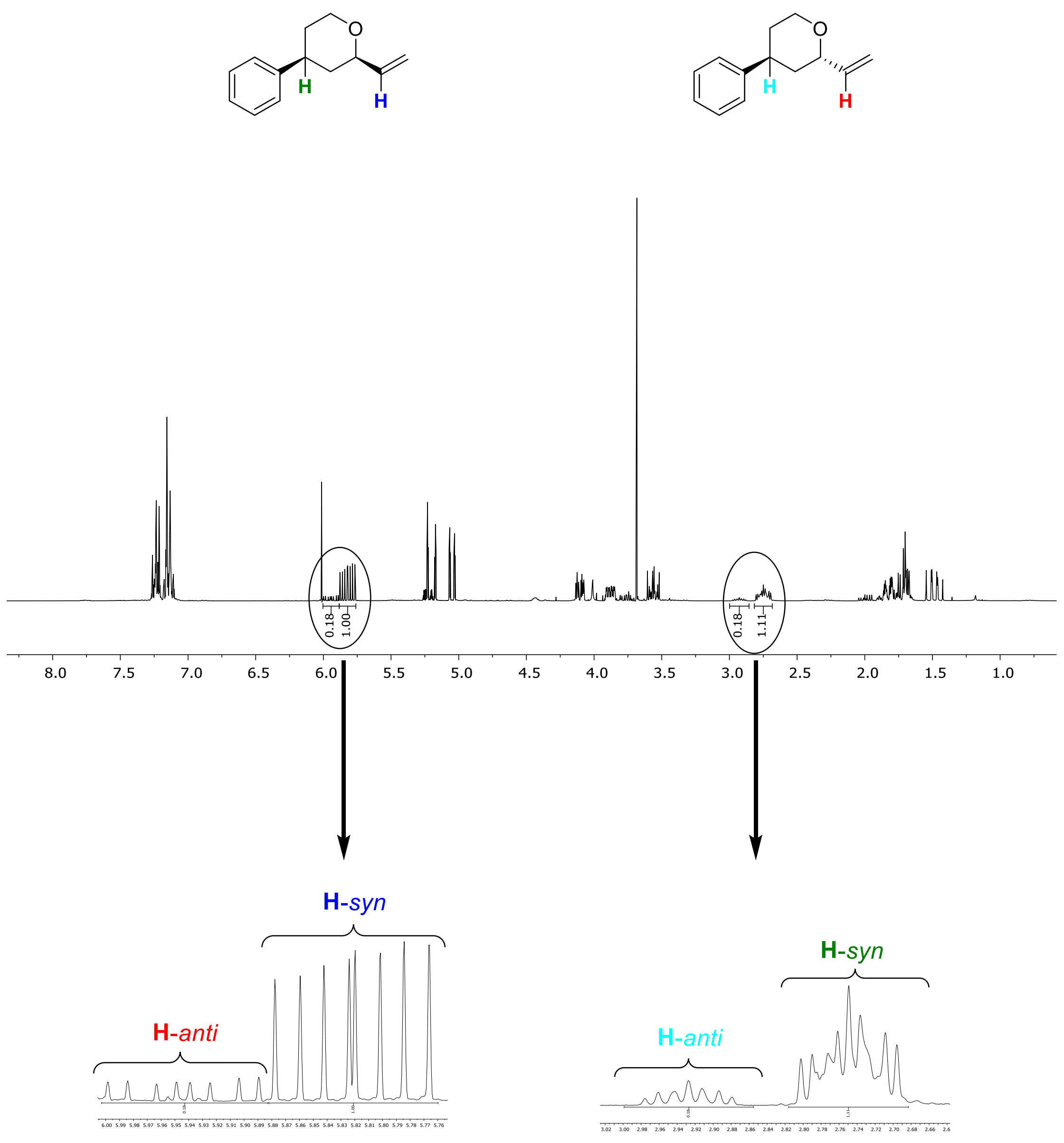

Calculation:

d.r. = H-syn/(H-anti + H-syn)

General procedure determination of d.r ratio: Catalysis Internal allene

To determine the syn/anti ratio the crude ^{1}H-NMR was measured in $CDCl_3$

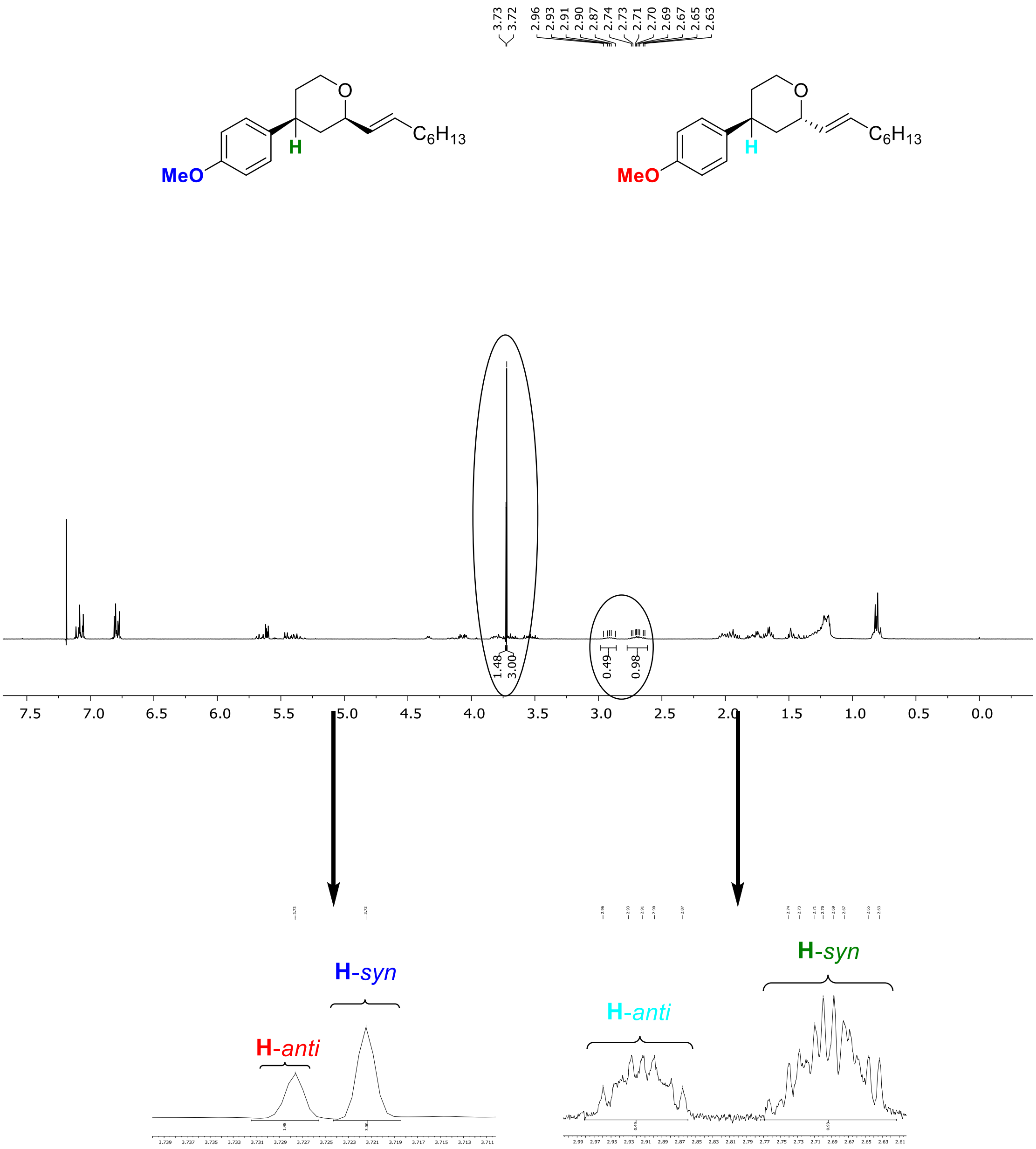

Calculation:

d.r. = H-syn/(H-anti + H-syn)

General procedure determination of E/Z-ratio: Catalysis internal allene

To determine the E/Z-ratio the crude ^{1}H-NMR was measured in toluene d-8.

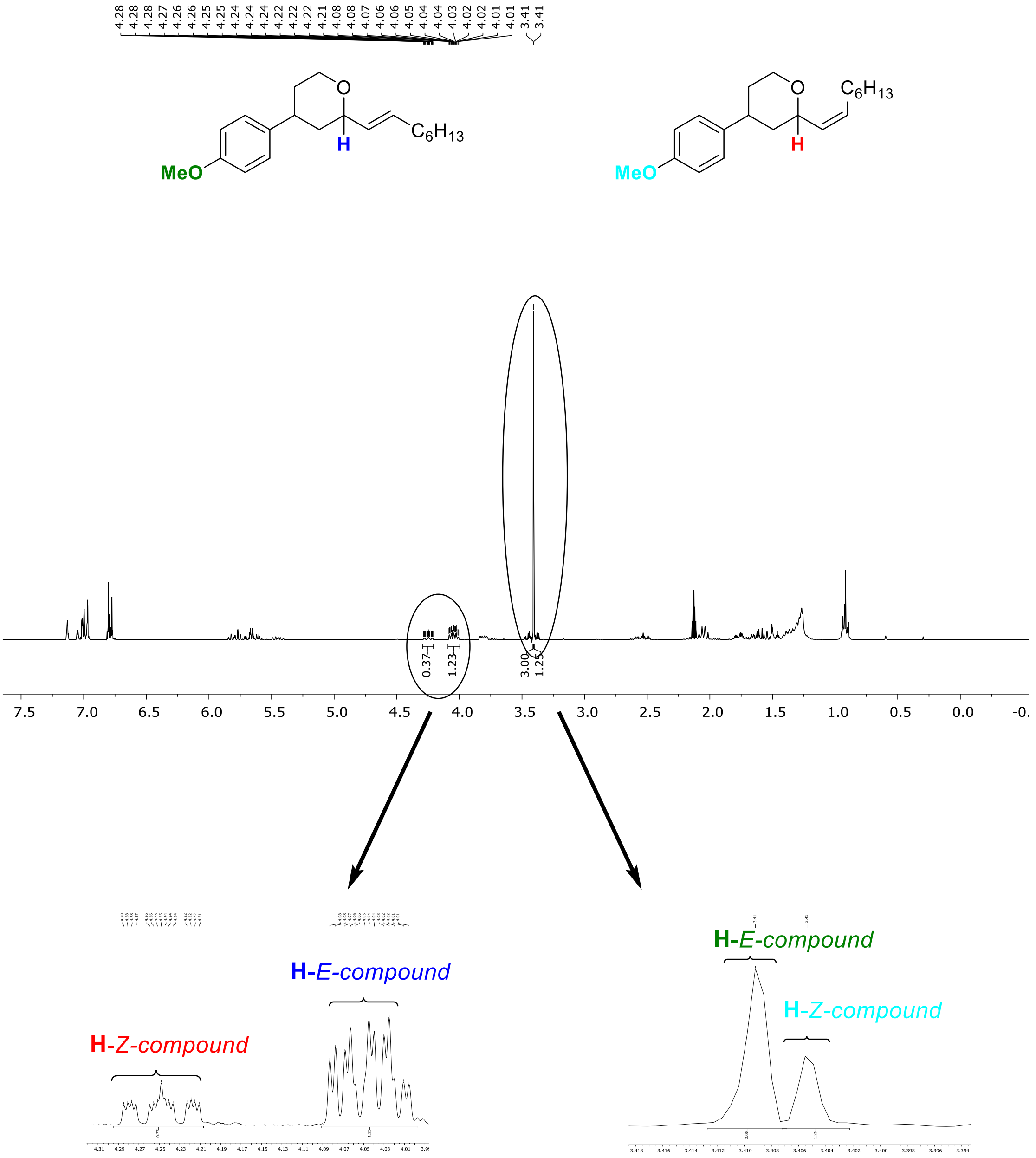

Calculation:

E/Z = H-E-compound(H-Z-compound+ H-E-compound

.

4 Condition screening

The condition screening was executed employing the general procedure 7 for terminal allenes and general procedure 8 for internal allenes. The general procedures were adapted towards the conditions shown in the corresponding tables. All reactions were performed in a 0.3 mmol scale.

4.1 Condition screening terminal allenes

Ligand-Screening:

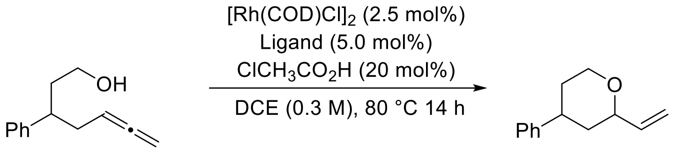

#	Ligand	Yield /%[a, b]	d.r. *(syn/anti)*[b]
1	DPEPhos	68	86/14
2	Xantphos	traces	n.d.
3	*rac*-BINAP	70	84/16
**4**	**DPPF**	**92**	**83/17**

All reactions were performed on a 0.3 mmol scale. (a) isolated yield of diastereomer mixture; (b) determined by ^{1}H-NMR analysis of the crude product

Additive-Screening:

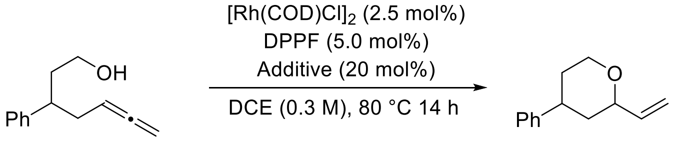

#	Additive	Yield /%[a]	d.r. *(syn/anti)* [b]
1	-		
2	Chloroacetic acid	92	87/13
3	$PhCMe_2CO_2H$	68	34/66
4	PPTS	80	92/8
5	Phenylacetic acid	31	71/29
**6**	**Diphenyl phosphate**	**87**	**93/7**

All reactions were performed on a 0.3 mmol scale. (a) isolated yield of diastereomer mixture; (b) determined by ^{1}H-NMR analysis of the crude product

Additive concentration screening:

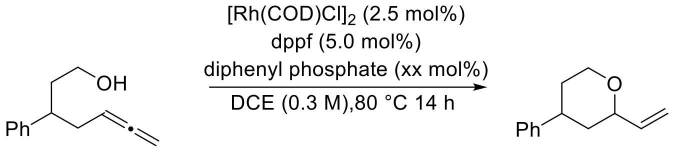

#	xx mol%	Yield /%[a]	d.r. *(syn/anti)* [b]
1	5.0	68	94/6
2	10	73	94/6
**3**	**20**	**87**	**93/7**
4	40	60	97/3

All reactions were performed on a 0.3 mmol scale. (a) isolated yield of diastereomer mixture; (b) determined by ^{1}H-NMR analysis of the crude product

Temperature screening:

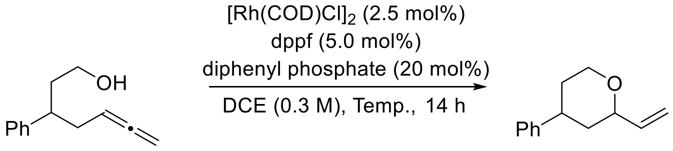

#	Temp. / °C	Yield /%[a]	d.r. *(syn/anti)* [b]
1	90	78	91/9
**2**	**80**	**87**	**93/7**
3	60	84	90/10
4	40	80	93/7
5	rt	56	87/13

All reactions were performed on a 0.3 mmol scale. (a) isolated yield of diastereomer mixture; (b) determined by ^{1}H-NMR analysis of the crude product

Reaction time screening:

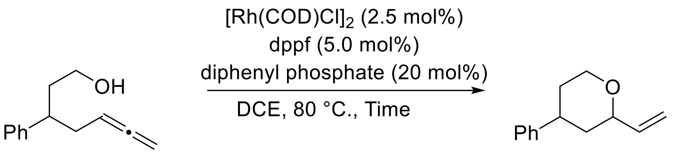

#	Time / h	**Yield /%**[(a)]	**d.r. *(synlanti)*** [(b)]
1	2	69	90/10
**2**	**4**	**70**	**93/7**
3	8	71	95/5
**4**	**14**	**87**	**93/7**

All reactions were performed on a 0.3 mmol scale. (a) isolated yield of diastereomer mixture; (b) determined by ^{1}H-NMR analysis of the crude product

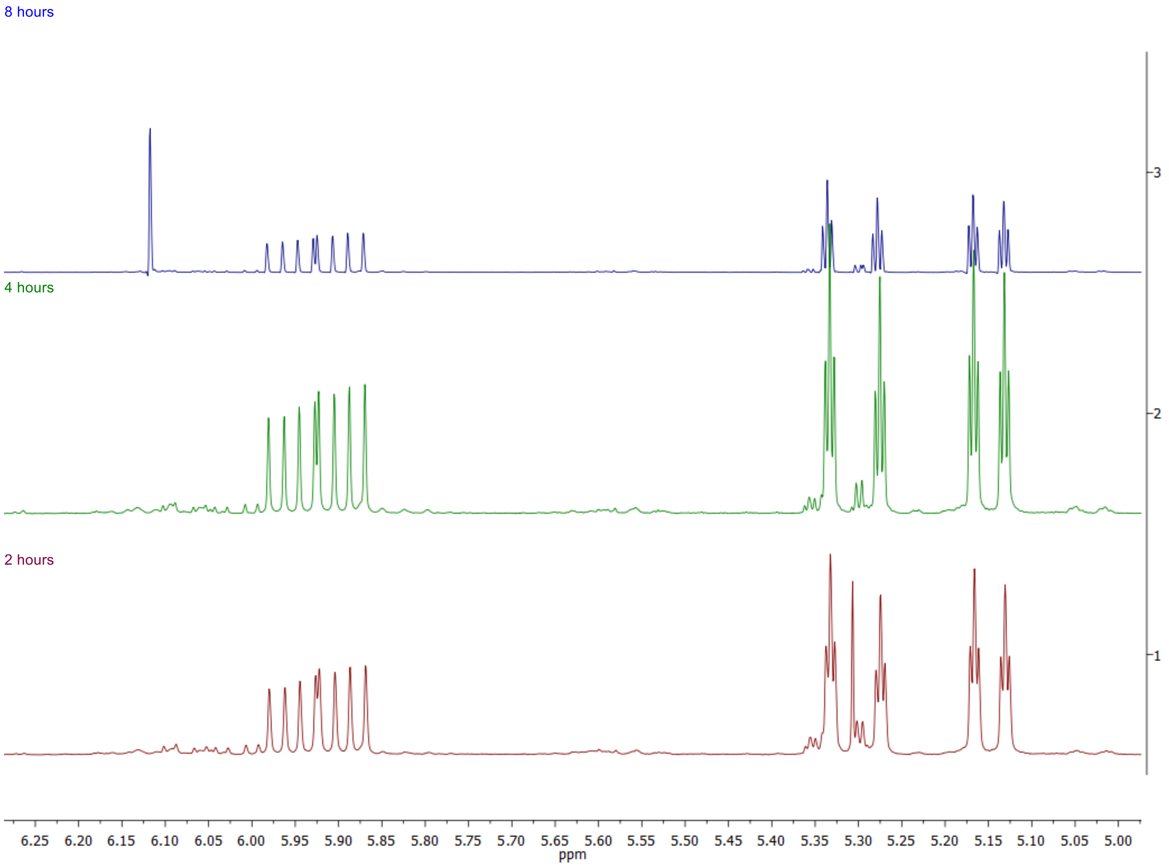

Solvent-Screening:

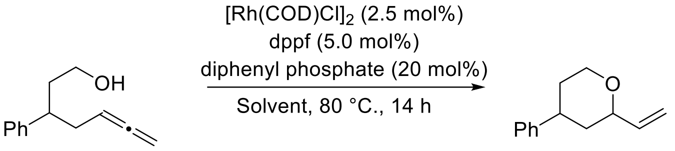

#	Solvent	Yield /%[(a)]	d.r. *(syn/anti)* [(b)]
**1**	**DCE**	**87**	**93/7**
**2**	**DCM**	**92**	**95/5**
3	Toluene	80	92/8
4	THF	80	92/8
5	PhF	77	93/7

All reactions were performed on a 0.3 mmol scale. (a) isolated yield of diastereomer mixture; (b) determined by ^{1}H-NMR analysis of the crude product

4.2 Condition screening internal allenes

Ligand-Screening:

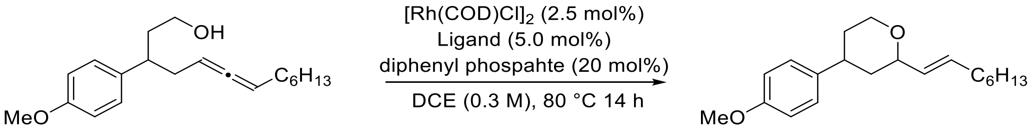

#	Ligand	Yield /%[(a)]	d.r. *(anti/syn)*[(b)]	*E/Z* [(b)]
1	**DPEPhos**	**86**	**82/18**	**75/25**
2	**dppf**	**98**	**70/30**	**79/21**
3	Xantphos	86	82/18	71/29
4	rac-Binap	55	76/24	73/27
5	Segphos	83	64/36	79/21
6	dppp	75	70/30	67/33

All reactions were performed on a 0.3 mmol scale. (a) isolated yield of diastereomer mixture; (b) determined by ^{1}H-NMR analysis of the crude product

Additive-Screening DPEPhos:

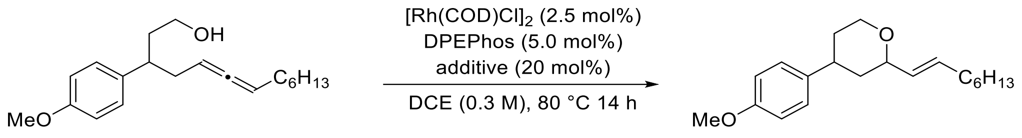

#	Additive	Yield /%[a]	d.r. (*syn/anti*)[b]	*E/Z* [b]
1	-	traces	-	-
2	Diphenyl phosphate	**86**	**82/18**	**75/25**
3	$ClCH_2CO_2H$	47	91/9	n.d.
4	PPTS	83	88/12	
5	(-)-Binol phospahte	82	80/20	
6	Phenylacetic acid	52	78/22	
**7**	TFA	78	81/19	60/40

All reactions were performed on a 0.3 mmol scale. (a) isolated yield of diastereomer mixture; (b) determined by ^{1}H-NMR analysis of the crude product

Additive-Screening dppf:

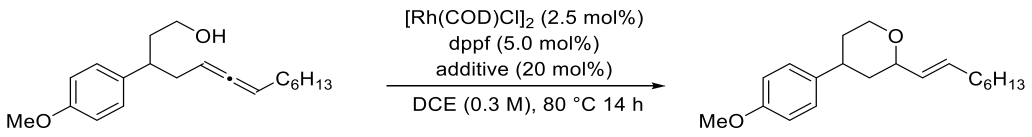

#	Additive	Yield /%[a]	d.r. (*syn/anti*)[b]	*E/Z* [b]
1	-	traces	-	-
2	Diphenyl phosphate	**98**	**70/30**	**79/21**
	(-)-Binol phosphate	**84**	**69/31**	**74/26**
3	$ClCH_2CO_2H$	49	70/30	n.d.
4	PPTS	86	82/18	80/20
5	**PTSA**	**98**	**92/8**	**70/30**
6	PPTS derivate	70	70/30	85/15
**7**	PPTS derivate	68	70/30	84/16
8	TFA	80	85/15	65/35
9	Benzosulfonsäure	88	94/6	72/28
10	(-) - Campfersulfonsäure	25	68/32	79/11
11	(+) - Campfersulfonsäure	23	70/30	80/20

12	Ethansulfonsäure	83	95/5	68/32

All reactions were performed on a 0.3 mmol scale. (a) NMR-Yield; (b) determined by ^{1}H-NMR analysis of the crude product

Extended ligand screening

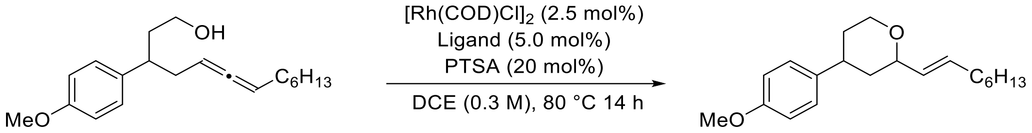

#	Ligand	Yield /%[(a)]	d.r. (*syn*/*anti*)[(b)]	*E/Z* [(b)]
1	dppf	98	92/8	70/30
2	L1	-	-	-
3	L2	40	88/12	63/27
4	L3	89	94/6	65/35
5	L4	91	96/4	68/32
**6**	**L5**	**96**	**91/9**	**86/14**
7	L6	65	92/8	80/20

All reactions were performed on a 0.3 mmol scale. (a) isolated yield of diastereomer mixture; (b) determined by ^{1}H-NMR analysis of the crude product

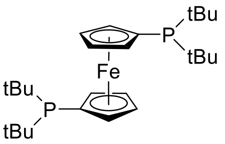

L1

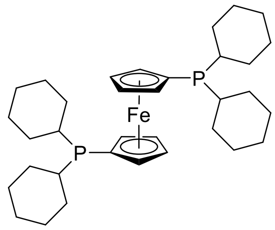

L2

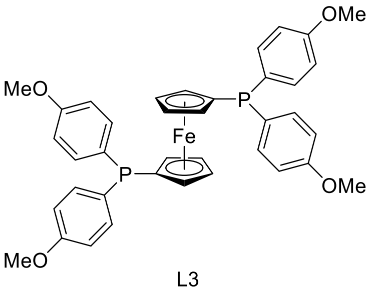

L3

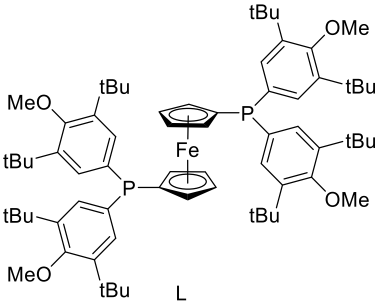

L
4

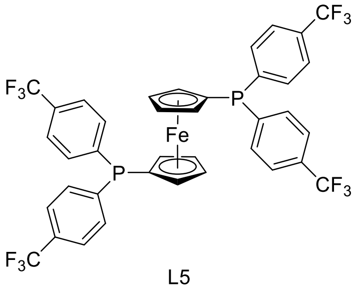

L5

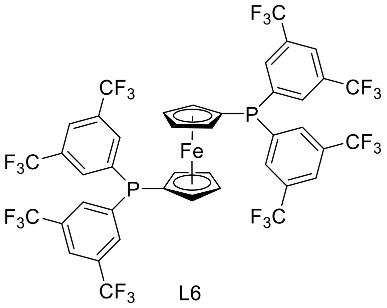

L6

Temperature-Screening:

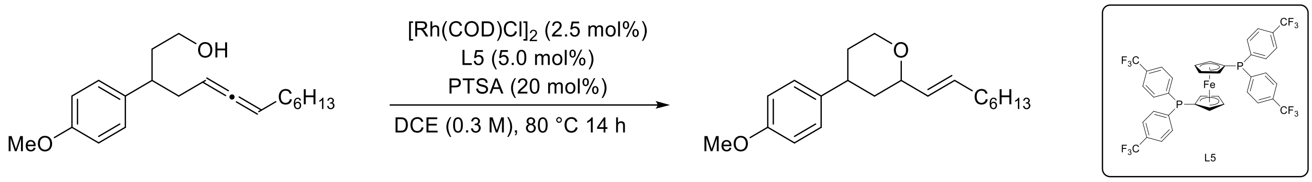

#	**Temp. / °C**	**Yield /%**[(a)]	**d.r. *(synlanti)***[(b)]	***E/Z*** [(b)]
1	Rt	95	73/27	68/32
2	50	97	72/28	67/33
3	70	94	77/23	82/18
4	80	98	92/8	70/30
5	90	97	91/9	78/22

All reactions were performed on a 0.3 mmol scale. (a) isolated yield of diastereomer mixture; (b) determined by ^{1}H-NMR analysis of the crude product

Solvent-Screening:

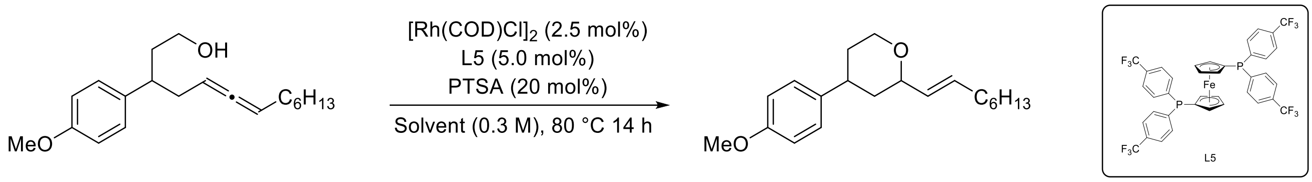

#	**Solvent**	**Yield /%**[(a)]	**d.r. *(synlanti)***[(b))]	***E/Z*** [(b)]
1	THF	81	78/22	73/27
2	Toluol	75	75/25	75/25
3	DCM	95	77/23	85/15
4	PhF	94	90/10	83/17

All reactions were performed on a 0.3 mmol scale. (a) isolated yield of diastereomer mixture; (b) determined by ^{1}H-NMR analysis of the crude product

Additive concentration-Screening

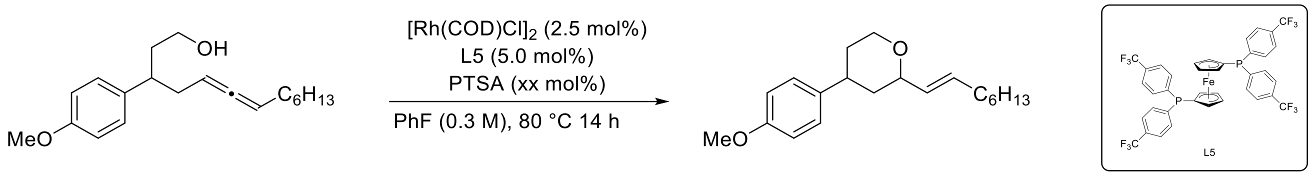

#	PTSA (xx mol%)	Yield /%[(a)]	d.r. *(syn/anti)*[(b)]	*E/Z* [(b)]
1	10	91	80/20	79/21
3	20	93	90/10	83/17
2	30	**96**	**95/5**	**84/16**
4	40	95	94/6	80/20

All reactions were performed on a 0.3 mmol scale. (a) isolated yield of diastereomer mixture; (b) determined by ^{1}H-NMR analysis of the crude product

5 Proposed Mechanism

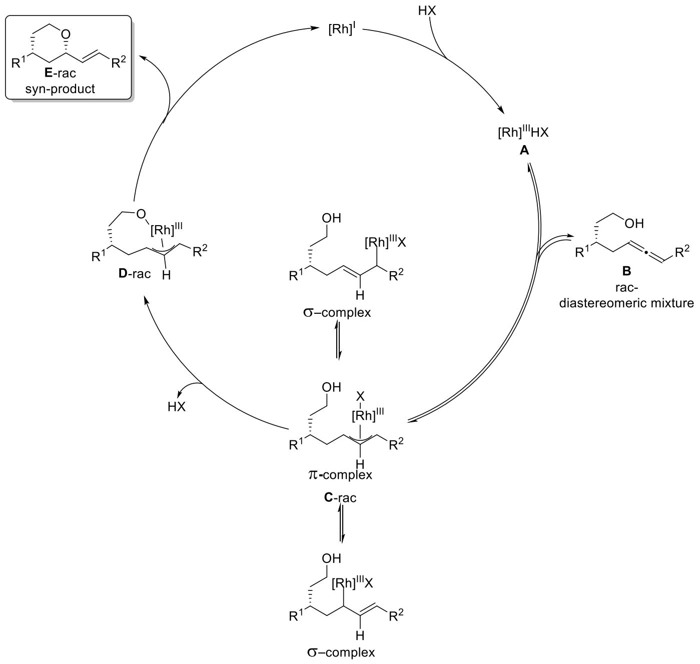

Scheme 1: Proposed mechanism for the cyclization of internal allenes

The cyclisation of allenols could only be achieved by adding an acidic additive. This observation suggests that these substrates are not able to form a Rh(III) allyl species on their own. Hence, the following mechanistic scheme can be proposed (Scheme 1). The role of the acid Additive may be the generation of a rhodium-hydride species (A) to initiate the catalytic cycle. After hydrometallation of the allene the rhodium-allyl species C is formed. The desired product is then formed via Ligand exchange and a reductive reductive elimination process.

6 Synthesis and characterization of *syn*-tetrahydropyrans

Synthesis of 4-methyl-2-vinyltetrahydro-2H-pyran 5

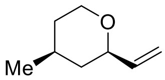

$C_8H_{14}O$
126.20

The reaction was performed according to **general procedure 8** with 3-methylhepta-5,6-dien-1-ol (38 mg, 0.3 mmol, 1.0 equiv.). The reaction mixture was filtered, concentrated and the **d.r.** ratio (**d.r. = 94/6**) was determined by ^{1}H-NMR spectroscopy of the crude product. After purification by flash chromatography on silica gel (pentane/ether 80/1 to 40/1) the product was obtained as colorless liquid (34 mg, 0.27 mmol, 90%).

Analytical Data[6]

^{1}H-NMR (400.1 MHz, $CDCl_3$): δ = 0.95 (d, *J* = 6.4, 3H), 0.99 – 1.07 (m, 1H), 1.22 (dddd, *J* = 13.2, 12.5, 11.6, 4.6 Hz, 1H), 1.50 – 1.58 (m, 1H), 1.63 – 1.72 (m, 2H), 3.47 (ddd, *J* = 12.5, 11.4, 2.3 Hz, 1H), 3.74 – 3.82 (m, 1H), 4.03 (ddd, *J* = 11.4, 4.5, 1.6 Hz, 1H), 5.08 (dt, *J* = 10.6, 1.5 Hz, 1H), 5.22 (ddd, *J* = 17.3, 1.6, 1.6 Hz, 1H), 5.85 (ddd, *J* = 17.3, 10.6, 5.5, 1H) ppm.

^{13}C-NMR (100.6 MHz, $CDCl_3$): δ = 22.4, 30.3, 34.5, 40.6, 68.1, 114.5, 139.5 ppm.

APCI-HRMS: *m/z* calcd for $C_8H_{15}O$ $[M+H]^+$ 127.1118 found 127.1118.

Synthesis of syn- 4-dodecyl-2-vinyltetrahydro-2H-pyran 6

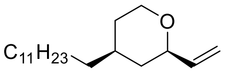

$C_{19}H_{36}O$
280.50

The reaction was performed according to **general procedure 8** with 3-(buta-2,3-dien-1-yl)pentadecan-1-ol (85 mg, 0.3 mmol, 1.0 equiv.). The reaction mixture was filtered, concentrated and the **d.r.** ratio (**d.r. = 94/6**) was determined by ^{1}H-NMR spectroscopy of the crude product. After purification by flash chromatography on silica gel (pentane/ether 80/1 to 40/1) the product was obtained as colorless liquid (79 mg, 0.28 mmol, 94%).

Analytical Data[6]

^{1}H-NMR (400.1 MHz, $CDCl_3$): δ = .0.86 – 0.91 (m, 3H), 1.00 (dd, *J* = 13.0, 11.4, 11.4 Hz, 1H), 1.26 (s, 23H), 1.45 – 1.55 (m, 1H), 1.55 – 1.61 (m, 1H), 1.67 – 1.75 (m, 1H), 3.46 (ddd, *J* = 12.6, 11.4, 2.1 Hz, 1H), 3.77 (dddd, *J* = 11.3, 5.3, 2.4, 1.3 Hz, 1H), 4.04 (ddd, *J* = 11.4, 4.6, 1.5 Hz, 1H), 5.08 (ddd, *J* = 10.6, 1.5, 1.5 Hz, 1H), 5.23 (ddd, *J* = 17.3, 1.6, 1.6 Hz, 1H), 5.85 (ddd, *J* = 17.4, 10.6, 5.5 Hz, 1H) ppm.

^{13}C-NMR (1006 MHz, $CDCl_3$): δ = 14.2, 22.8, 26.5, 29.4, 29.7, 29.8, 29.9, 32.0, 32.8, 35.3, 37.1, 38.8, 68.1, 78.3, 114.5, 139.6 ppm.

APCI-HRMS: *m/z* calcd for $C_{19}H_{36}O$ $[M+H]^+$ 281.2841 found 281.2842.

Synthesis of syn- 4-neopentyl-2-vinyltetrahydro-2H-pyran 7

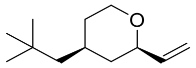

$C_{12}H_{22}O$
182.31

The reaction was performed according to **general procedure 8** with 3-neopentylhepta-5,6-dien-1-ol (55 mg, 0.3 mmol, 1.0 equiv.). The reaction mixture was filtered, concentrated and the **d.r.** ratio (**d.r. = 85/15**) was determined by ^{1}H-NMR spectroscopy of the crude product. After purification by flash chromatography on silica gel (pentane/ether 80/1 to 40/1) the product was obtained as colorless liquid (51 mg, 0.28 mmol, 93%).

Analytical Data[5]

^{1}H-NMR (400.1 MHz, $CDCl_3$): δ = 0.92 (s, 9H), 1.07 (dd, *J* = 13.0, 11.4 Hz, 1H), 1.16 (dd, *J* = 5.0, 2.1 Hz, 2H), 1.25 – 1.31 (m, 1H), 1.57 – 1.62 (m, 1H), 1.67 – 1.74 (m, 1H), 3.48 (ddd, *J* = 12.4, 11.5, 2.1, 1H), 3.79 (dddd, *J* = 11.3, 5.4, 2.4, 1.4 Hz, 1H), 4.01 (ddd, *J* = 11.6, 4.5, 1.6 Hz, 1H), 5.08 (ddd, *J* = 10.6, 1.3 Hz, 1H), 5.22 (dt, *J* = 17.4, 1.6 Hz, 1H), 5.84 (ddd, *J* = 17.4, 10.6, 5.5 Hz, 1H).ppm.
^{13}C-NMR (100.6 MHz, $CDCl_3$): δ = 29.8, 30.2, 31.2, 32.0, 35.1, 41.0, 51.4, 68.1, 78.3, 114.5, 139.5 ppm.
APCI-HRMS: *m/z* calcd for $C_{12}H_{23}O$ $[M+H]^+$ 183.1742 found 183.1742.

Synthesis of syn- 4-cyclopropyl-2-vinyltetrahydro-2H-pyran 8

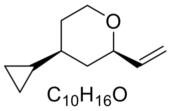

$C_{10}H_{16}O$
152.24

The reaction was performed according to **general procedure 8** with 3-cyclopropylhepta-5,6-dien-1-ol (46 mg, 0.3 mmol, 1.0 equiv.). The reaction mixture was filtered, concentrated and the **d.r.** ratio (**d.r. = 92/8**) was determined by ^{1}H-NMR spectroscopy of the crude product. After purification by flash chromatography on silica gel (pentane/ether 80/1 to 40/1) the product was obtained as colorless liquid (40 mg, 0.26 mmol, 88%).

Analytical Data[5]

^{1}H-NMR (400.1 MHz, $CDCl_3$): δ = 0.05 – 0.13 (m, 2H), 0.35 – 0.45 (m, 2H), 0.47 – 0.60 (m, 1H), 0.81 (ddd, *J* = 12.1, 8.6, 3.9 Hz, 1H), 1.18 (ddd, *J* = 13.1, 11.9, 11.3 Hz, 1H), 1.32 – 1.47 (m, 1H), 1.64 (ddd, *J* = 13.3, 3.9, 2.0 Hz, 1H), 1.73 – 1.83 (m, 1H), 3.40 (ddd, *J* = 12.5, 11.4, 2.3 Hz, 1H), 3.71 (dddd, *J* = 11.3, 5.2, 2.5, 1.4 Hz, 1H), 4.05 (ddd, *J* = 11.4, 4.6, 1.6 Hz, 1H), 5.04 – 5.13 (m, 1H), 5.23 (dd, *J* = 17.3, 1.6 Hz, 1H), 5.86 (ddd, *J* = 17.4, 10.7, 5.5 Hz, 1H) ppm.
^{13}C-NMR (100.6 MHz, $CDCl_3$): δ = 2.8, 2.9, 17.2, 32.3, 38.3, 40.6, 68.1, 78.2, 114.5, 139.5 ppm.
APCI-HRMS: *m/z* calcd for $C_{10}H_{16}ONNaS$ $[M+]^+$ 153.1273 found 153.1273.

Synthesis of syn- 4-cyclohexyl-2-vinyltetrahydro-2H-pyran 9

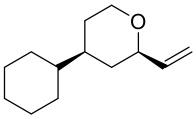

$C_{13}H_{22}O$
194.32

The reaction was performed according to **general procedure 8** with 3-cyclohexylhepta-5,6-dien-1-ol (58 mg, 0.3 mmol, 1.0 equiv.). The reaction mixture was filtered, concentrated and the **d.r.** ratio (**d.r. = 97/3**) was determined by ^{1}H-NMR spectroscopy of the crude product. After purification by flash chromatography on silica gel (pentane/ether 80/1 to 40/1) the product was obtained as colorless liquid (47 mg, 0.24 mmol, 81%).

Analytical Data[5]

^{1}H-NMR (400.1 MHz, $CDCl_3$): δ = 0.87 – 1.01 (m, 2H), 1.04 – 1.30 (m, 7H), 1.53 – 1.59 (m, 1H), 1.64 – 1.76 (m, 6H), 3.44 (ddd, *J* = 12.0, 11.4, 2.3 Hz, 1H), 3.75 (dddd, *J* = 11.2, 5.2, 2.5, 1.4 Hz, 1H), 4.06 (ddd, *J* = 11.2, 4.3, 1.7 Hz, 1H), 5.06 – 5.10 (m, 1H), 5.22 (ddd, *J* = 17.3, 1.6 Hz, 1H), 5.85 (ddd, *J* = 17.3, 10.6, 5.5 Hz, 1H) ppm.

^{13}C-NMR (100.6 MHz, $CDCl_3$): δ = 26.7, 26.8, 29.8, 30.0, 30.1, 35.9, 40.9, 42.9, 68.3, 78.5, 114.4, 139.7 ppm.

APCI-HRMS: *m/z* calcd for $C_{13}H_{23}O$ $[M+H]^+$ 195.1743 found 195.1743.

Synthesis of syn- 4-(3-phenylpropyl)-2-vinyltetrahydro-2H-pyran 10

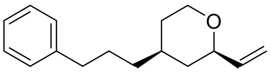

$C_{16}H_{22}O$
230.35

The reaction was performed according to **general procedure 8** with 3-(3-phenylpropyl)hepta-5,6-dien-1-ol (69 mg, 0.3 mmol, 1.0 equiv.). The reaction mixture was filtered, concentrated and the **d.r.** ratio (**d.r. = 96/4**) was determined by ^{1}H-NMR spectroscopy of the crude product. After purification by flash chromatography on silica gel (pentane/ether 80/1 to 40/1) the product was obtained as colorless liquid (66 mg, 0.29 mmol, 96%).

Analytical Data[5]

^{1}H-NMR (400.1 MHz, $CDCl_3$): δ = 1.01 (dd, *J* = 13.1, 11.4, 1H), 1.19 – 1.34 (m, 3H), 1.55 – 1.75 (m, 5H), 2.58 – 2.64 (m, 2H), 3.46 (ddd, *J* = 12.6, 11.4, 2.1 Hz, 1H), 3.77 (dddd, *J* = 11.3, 5.2, 2.4, 1.4 Hz, 1H), 4.04 (ddd, *J* = 11.5, 4.6, 1.5 Hz, 1H), 5.06 – 5.10 (m, 1H), 5.23 (dd, *J* = 17.3, 1.6 Hz, 1H), 5.85 (ddd, *J* = 17.3, 10.6, 5.5 Hz, 1H), 7.16 – 7.21 (m, 3H), 7.26 – 7.31 (m, 2H) ppm.

^{13}C-NMR (100.6 MHz, $CDCl_3$): δ = 28.4, 32.7, 35.2, 36.2, 36.7, 38.7, 68.1 , 78.2, 114.5, 125.8, 128.4, 128.4, 139.5, 142.6 ppm.

APCI-HRMS: *m/z* calcd for $C_{16}H_{26}ON$ $[M+NH_4]^+$ 248.2014 found 248.2009.

Synthesis of syn- 4-phenyl-2-vinyltetrahydro-2H-pyran 2

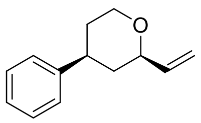

$C_{13}H_{16}O$
188.27

The reaction was performed according to **general procedure 8** with 3-phenylhepta-5,6-dien-1-ol (56 mg, 0.3 mmol, 1.0 equiv.). The reaction mixture was filtered, concentrated and the **d.r.** ratio (**d.r. = 93/7**) was determined by ^{1}H-NMR spectroscopy of the crude product. After purification by flash chromatography on silica gel (pentane/ether 80/1 to 40/1) the product was obtained as colorless liquid (49 mg, 0.26 mmol, 87%).

Analytical Data[5]

^{1}H-NMR (400.1 MHz, $CDCl_3$): δ = 1.57 (ddd, *J* = 13.2, 12.3, 11.1 Hz, 2H), 1.76 – 1.84 (m, 2H), 1.89 – 1.95 (m, 1H), 2.79 – 2.90 (m, 1H), 3.61 – 3.69 (m, 1H), 3.96 (dddd, *J* = 11.1, 5.2, 2.4 Hz, 1.4, 1H), 4.18 (ddd, *J* = 11.5, 4.0, 2.3 Hz, 1H), 5.13 (dd, *J* = 10.7, 1.5 Hz, 1H), 5.29 (dd, *J* = 17.3, 1.6 Hz, 1H), 5.91 (ddd, *J* = 17.3, 10.6, 5.4 Hz, 1H), 7.22 – 7.25 (m, 3H), 7.30 – 7.35 (m, 2H) ppm.

^{13}C-NMR (100.6 MHz, $CDCl_3$): δ =33.4, 39.4, 41.8, 68.2, 78.3, 114.9, 126.5, 126.8, 128.6, 139.1, 145.7 ppm.

APCI-HRMS: *m/z* calcd for $C_{13}H_{17}O$ $[M+H]^+$ 189.1274 found 189.1273.

Synthesis of syn- 4-(naphthalen-2-yl)-2-vinyltetrahydro-2H-pyran 11

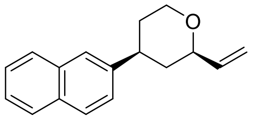

$C_{17}H_{18}O$
238.33

The reaction was performed according to **general procedure 8** with 3-(naphthalen-2-yl)hepta-5,6-dien-1-ol (72 mg, 0.3 mmol, 1.0 equiv.). The reaction mixture was filtered, concentrated and the **d.r.** ratio (**d.r. = 95/5**) was determined by ^{1}H-NMR spectroscopy of the crude product. After purification by flash chromatography on silica gel (pentane/ether 80/1 to 40/1) the product was obtained as colorless liquid (69 mg, 0.29 mmol, 97%).

Analytical Data[5]

^{1}H-NMR (400.1 MHz, $CDCl_3$): δ = 1.67 (ddd, *J* = 13.2, 12.2, 11.1 Hz, 1H), 1.82 – 1.94 (m, 2H), 1.97 – 2.05 (m, 1H), 2.96 – 3.05 (m, 1H), 3.66 – 3.74 (m, 1H), 4.01 (dddd, *J* = 11.1, 5.2, 2.4, 1.4 Hz, 1H), 4.23 (ddd, *J* = 11.6, 4.2, 2.1 Hz, 1H), 5.15 (dd, *J* = 10.6, 1.5 Hz, 1H), 5.31 (dd, *J* = 17.4, 1.5 Hz, 1H), 5.94 (ddd, *J* = 17.3, 10.7, 5.4 Hz, 1H), 7.36 – 7.51 (m, 3H), 7.64 – 7.67 (m, 1H), 7.78 – 7.84 (m, 3H).ppm.

^{13}C-NMR (100.6 MHz, $CDCl_3$): δ = 33.4, 39.3, 41.9, 68.3, 78.4, 115.0, 124.8, 125.5, 125.7, 126.1, 127.7, 127.7, 128.2, 132.4, 133.7, 139.1, 143.1 ppm.

APCI-HRMS: *m/z* calcd for $C_{17}H_{22}O$<n $[M+NH_4]^+$ 256.1697 found 256.1697.

Synthesis of syn- 4-([1,1'-biphenyl]-4-yl)-2-vinyltetrahydro-2H-pyran 12

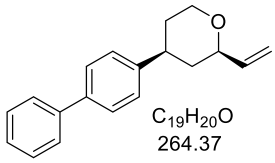

$C_{19}H_{20}O$
264.37

The reaction was performed according to **general procedure 8** with 3-([1,1'-biphenyl]-4-yl)hepta-5,6-dien-1-ol (79 mg, 0.3 mmol, 1.0 equiv.). The reaction mixture was filtered, concentrated and the **d.r.** ratio (**d.r. = 95/5**) was determined by ^{1}H-NMR spectroscopy of the crude product. After purification by flash chromatography on silica gel (pentane/ether 80/1 to 40/1) the product was obtained as colorless liquid (70 mg, 0.26 mmol, 88%).

Analytical Data[5]

^{1}H-NMR (400.1 MHz, $CDCl_3$): δ = 1.57 – 1.66 (m, 1H), 1.79 – 1.88 (m, 2H), 1.93 – 1.99 (m, 1H), 2.85 – 2.94 (m, 1H), 3.63 – 3.71 (m, 1H), 3.98 (dddd, *J* = 11.2, 5.2, 2.5 Hz, 1.4, 1H), 4.21 (ddd, *J* = 11.5, 4.1, 2.4 Hz, 1H), 5.15 (d, *J* = 10.6, 1.5 Hz, 1H), 5.31 (dd, *J* = 17.4, 1.5 Hz, 1H), 5.93 (ddd, *J* = 17.3, 10.6, 5.4 Hz, 1H), 7.30 – 7.33 (m, 2H), 7.33 – 7.37 (m, 1H), 7.42 – 7.46 (m, 2H), 7.55 – 7.61 (m, 4H) ppm.

^{13}C-NMR (100.6 MHz, $CDCl_3$): δ = 33.4, 39.4, 41.4, 68.2, 78.3, 114.9, 127.1, 127.2, 127.2, 127.4, 128.8, 139.1, 139.5, 141.0, 144.7 ppm.

APCI-HRMS: *m/z* calcd for $C_{19}H_{21}O$ $[M+H]^+$ 265.1587 found 265.1586.

Synthesis of syn- 4-(p-tolyl)-2-vinyltetrahydro-2H-pyran 13

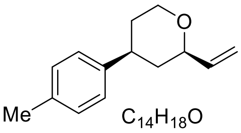

$C_{14}H_{18}O$
202.30

The reaction was performed according to **general procedure 8** with 3-(p-tolyl)hepta-5,6-dien-1-ol (61 mg, 0.3 mmol, 1.0 equiv.). The reaction mixture was filtered, concentrated and the **d.r.** ratio (**d.r. = 96/4**) was determined by ^{1}H-NMR spectroscopy of the crude product. After purification by flash chromatography on silica gel (pentane/ether 80/1 to 40/1) the product was obtained as colorless liquid (57 mg, 0.28 mmol, 94%).

Analytical Data[5]

^{1}H-NMR (400.1 MHz, $CDCl_3$): δ = 1.55 (ddd, *J* = 13.2, 12.3, 11.1 Hz, 1H), 1.74 – 1.81 (m, 2H), 1.87 – 1.93 (m, 1H), 2.34 (s, 3H), 2.80 (dddd, *J* = 12.4, 11.0, 5.7, 3.7 Hz, 1H), 3.60 – 3.68 (m, 1H), 3.95 (dddd, *J* = 11.2, 5.2, 2.5, 1.4 Hz, 1H), 4.18 (ddd, *J* = 11.5, 4.3, 2.4 Hz, 1H), 5.13 (dd, *J* = 10.6, 1.5 Hz, 1H), 5.28 (dd, *J* = 17.4, 1.6 Hz, 1H), 5.91 (ddd, *J* = 17.3, 10.6, 5.4 Hz, 1H), 7.13 (m_c, 4H).ppm.

^{13}C-NMR (100.6 MHz, $CDCl_3$): δ = 21.0, 33.5, 39.5, 41.3, 68.3, 78.3, 114.8, 126.7, 129.3, 136.0, 139.1, 142.7 ppm.

APCI-HRMS: *m/z* calcd for $C_{14}H_{19}O$ $[M+H]^+$ 203.1430 found 203.1429.

Synthesis of syn- 4-(m-tolyl)-2-vinyltetrahydro-2H-pyran 14

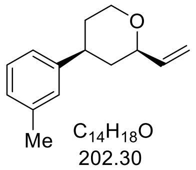

$C_{14}H_{18}O$
202.30

The reaction was performed according to **general procedure 8** with 3-(m-tolyl)hepta-5,6-dien-1-ol (61 mg, 0.3 mmol, 1.0 equiv.). The reaction mixture was filtered, concentrated and the **d.r.** ratio (**d.r. = 95/5**) was determined by ^{1}H-NMR spectroscopy of the crude product. After purification by flash chromatography on silica gel (pentane/ether 80/1 to 40/1) the product was obtained as colorless liquid (57 mg, 0.28 mmol, 93%).

Analytical Data[5]

^{1}H-NMR (400.1 MHz, $CDCl_3$): δ = 1.47 – 1.64 (m, 1H), 1.73 – 1.83 (m, 2H), 1.86 – 1.95 (m, 1H), 2.35 (s, 3H), 2.74 – 2.86 (m, 1H), 3.59 – 3.70 (m, 1H), 3.95 (dddd, *J* = 11.1 5.2, 2.5, 1.4 Hz, 1H), 4.18 (ddd, *J* = 11.5, 4.1, 2.2 Hz, 1H), 5.13 (dd, *J* = 10.6, 1.5 Hz, 1H), 5.28 (dd, *J* = 17.3, 1.6 Hz, 1H), 5.91 (ddd, *J* = 17.4, 10.7, 5.4 Hz, 1H), 7.01 – 7.06 (m, 3H), 7.19 – 7.24 (m, 1H) ppm.

^{13}C-NMR (100.6 MHz, $CDCl_3$): δ =21.6, 33.4, 39.4, 41.7, 68.3, 78.3, 114.8, 123.8, 127.2, 127.6, 128.5, 138.2, 139.1, 145.7 ppm.

APCI-HRMS: *m/z* calcd for $C_{14}H_{22}ON$ $[M+NH_4]^+$ 220.1696 found 220.1693.

Synthesis of syn- 4-(o-tolyl)-2-vinyltetrahydro-2H-pyran 15

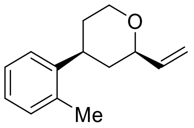

$C_{14}H_{18}O$
202.30

The reaction was performed according to **general procedure 8** with 3-(o-tolyl)hepta-5,6-dien-1-ol (61 mg, 0.3 mmol, 1.0 equiv.). The reaction mixture was filtered, concentrated and the **d.r.** ratio (**d.r. = 94/6**) was determined by ^{1}H-NMR spectroscopy of the crude product. After purification by flash chromatography on silica gel (pentane/ether 80/1 to 40/1) the product was obtained as colorless liquid (54 mg, 0.27 mmol, 89%).

Analytical Data[5]

^{1}H-NMR (400.1 MHz, $CDCl_3$): δ = 1.57 (ddd, *J* = 13.2, 12.1, 11.1 Hz, 1H), 1.70 (ddd, *J* = 13.3, 4.0, 2.1 Hz, 1H), 1.78 – 1.87 (m, 2H), 2.37 (s, 3H), 3.07 (dddd, *J* = 12.0, 3.7 Hz, 1H), 3.61 – 3.74 (m, 1H), 3.99 (dddd, *J* = 11.1, 5.4, 2.3, 1.3 Hz, 1H), 4.17 – 4.24 (m, 1H), 5.13 (dd, *J* = 10.6, 1.5 Hz, 1H), 5.29 (dd, *J* = 17.3, 1.5 Hz, 1H), 5.91 (ddd, *J* = 17.4, 10.7, 5.4 Hz, 1H), 7.11 – 7.22 (m, 4H) ppm.

^{13}C-NMR (100.6 MHz, $CDCl_3$): δ =19.4, 32.7, 37.4, 38.6, 68.5, 114.9, 125.6, 126.1, 126.5, 130.5, 135.1, 139.1, 143.4 ppm.

APCI-HRMS: *m/z* calcd for $C_{14}H_{22}ON$ $[M+NH_4]^+$ 220.1696 found 220.1693.

Synthesis of syn- 4-mesityl-2-vinyltetrahydro-2H-pyran 16

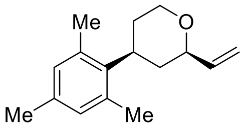

The reaction was performed according to **general procedure 8** with 3-mesitylhepta-5,6-dien-1-o (69 mg, 0.3 mmol, 1.0 equiv.). The reaction mixture was filtered, concentrated and the **d.r.** ratio (**d.r. = 97/3**) was determined by ^{1}H-NMR spectroscopy of the crude product. After purification by flash chromatography on silica gel (pentane/ether 80/1 to 40/1) the product was obtained as colorless liquid (63 mg, 0.27 mmol, 91%).

Analytical Data[5]

^{1}H-NMR (400.1 MHz, CDCl$_3$): δ = 1.5 (dd, *J* = 13.d4, 4.0, 1.8 Hz, 1H), 1.7 (dddd, *J* = 13.3, 3.9, 2.3, 1.7 Hz, 1H), 2.0 (dd, *J* = 12.9, 10.9 Hz, 1H), 2.2 (s, 3H), 2.3 – 2.3 (m, 1H), 2.4 (s, 6H), 3.3 (dddd, *J* = 12.6, 3.8 Hz, 1H), 3.6 (ddd, *J* = 12.0, 11.4, 2.4 Hz, 1H), 3.9 (dddd, *J* = 10.7, 5.3, 2.6, 1.4 Hz, 1H), 4.2 – 4.2 (m, 1H), 5.1 (dd, *J* = 10.7, 1.5 Hz, 1H), 5.3 (dd, *J* = 17.4, 1.6 Hz, 1H), 5.9 (ddd, *J* = 17.3, 10.7, 5.3 Hz, 1H), 6.8 (m_c, 2H).ppm.

^{13}C-NMR (100.6 MHz, CDCl$_3$): δ = 20.7, 21.8, 29.8, 35.8, 38.3, 69.1, 79.2, 114.8, 135.5, 136.3, 137.6, 139.2 ppm.

APCI-HRMS: *m/z* calcd for $C_{16}H_3O$ $[M+H]^+$ 231.1743 found 231.1741.

Synthesis of syn- 2-vinyl-4-(4-vinylphenyl)tetrahydro-2H-pyran 17

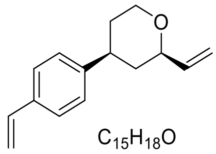

$C_{15}H_{18}O$
214.31

The reaction was performed according to **general procedure 8** with 2-vinyl-4-(4-vinylphenyl)tetrahydro-2H-pyran (64 mg, 0.3 mmol, 1.0 equiv.). The reaction mixture was filtered, concentrated and the **d.r.** ratio (**d.r. = 95/5**) was determined by ^{1}H-NMR spectroscopy of the crude product. After purification by flash chromatography on silica gel (pentane/ether 80/1 to 40/1) the product was obtained as colorless liquid (56 mg, 0.26 mmol, 88%).

Analytical Data[5]

^{1}H-NMR (400.1 MHz, $CDCl_3$): δ = 1.55 (ddd, *J* = 13.2, 12.3, 11.1 Hz, 1H), 1.74 – 1.81 (m, 2H), 1.86 – 1.94 (m, 1H), 2.78 – 2.89 (m, 1H), 3.59 – 3.68 (m, 1H), 3.95 (dddd, *J* = 11.1, 5.2, 2.5, 1.4 Hz, 1H), 4.18 (ddd, *J* = 11.5, 4.2, 2.4 Hz, 1H), 5.13 (dd, *J* = 10.6, 1.5 Hz, 1H), 5.21 (dd, *J* = 10.9, 1.0 Hz, 1H), 5.28 (dd, *J* = 17.3, 1.5 Hz, 1H), 5.72 (dd, *J* = 17.6, 1.0 Hz, 1H), 5.90 (ddd, *J* = 17.3, 10.6, 5.4 Hz, 1H), 6.66 – 6.76 (m, 1H), 7.16 – 7.23 (m, 2H), 7.34 – 7.40 (m, 2H).ppm.

^{13}C-NMR (100.6 MHz, $CDCl_3$): δ =33.3, 39.3, 41.5, 68.2, 78.3, 113.4, 114.9, 126.5, 127.0, 136.0, 136.6, 139.1, 145.3 ppm.

ESI-HRMS: *m/z* calcd for $C_{15}H_{19}O$ $[M+H]^+$ 215.1433 found 215.1430.

Synthesis of syn- 4-(4-(trifluoromethyl)phenyl)-2-vinyltetrahydro-2H-pyran 18

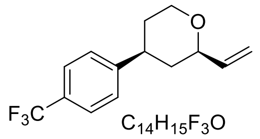

The reaction was performed according to **general procedure 8** with 3-(4-(trifluoromethyl)phenyl)hepta-5,6-dien-1-ol (77 mg, 0.3 mmol, 1.0 equiv.). The reaction mixture was filtered, concentrated and the **d.r.** ratio (**d.r. = 95/5**) was determined by ^{1}H-NMR spectroscopy of the crude product. After purification by flash chromatography on silica gel (pentane/ether 80/1 to 40/1) the product was obtained as colorless liquid (74 mg, 0.29 mmol, 98%).

Analytical Data[5]

^{1}H-NMR (400.1 MHz, $CDCl_3$): δ = 1.52 – 1.61 (m, 1H), 1.75 – 1.81 (m, 2H), 1.88 – 1.96 (m, 1H), 2.85 – 2.96 (m, 1H), 3.60 – 3.69 (m, 1H), 3.92 – 4.00 (m, 1H), 4.14 – 4.25 (m, 1H), 5.14 (dd, *J* = 10.6, 1.4 Hz, 1H), 5.29 (dd, *J* = 17.3, 1.5 Hz, 1H), 5.90 (ddd, *J* = 17.4, 10.7, 5.4 Hz, 1H), 7.34 (dd, *J*=8.1, 0.7 Hz, 2H), 7.57 (dd, *J* = 8.0, 0.8 Hz, 2H) ppm.

^{13}C-NMR (100.6 MHz, $CDCl_3$): δ =33.1, 39.1, 41.7, 68.0, 78.2, 115.2, 125.5, 125.6, 125.6, 127.2, 138.8, 149.6 ppm.

APCI-HRMS: *m/z* calcd for $C_{14}H_{16}F_3O$ $[M+H]^+$ 257.1145 found 257.1144.

Synthesis of syn- 4-(4-bromophenyl)-2-vinyltetrahydro-2H-pyran 19

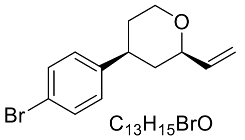

The reaction was performed according to **general procedure 8** with 3-(4-bromophenyl)hepta-5,6-dien-1-ol (80 mg, 0.3 mmol, 1.0 equiv.). The reaction mixture was filtered, concentrated and the **d.r.** ratio (**d.r. = 96/4**) was determined by ^{1}H-NMR spectroscopy of the crude product. After purification by flash chromatography on silica gel (pentane/ether 80/1 to 40/1) the product was obtained as colorless liquid (77 mg, 0.29 mmol, 98%).

Analytical Data[5]

^{1}H-NMR (400.1 MHz, $CDCl_3$): δ = 1.52 – 1.61 (m, 1H), 1.76 – 1.83 (m, 2H), 1.86 – 1.96 (m, 1H), 2.79 – 2.91 (m, 1H), 3.60 – 3.70 (m, 1H), 3.96 (dddd, *J* = 11.1, 5.2, 2.5, 1.4 Hz, 1H), 4.19 (ddd, *J* = 11.6, 4.0, 2.4 Hz, 1H), 5.12 – 5.15 (m, 1H), 5.29 (dd, *J* = 17.3, 1.5 Hz, 1H), 5.91 (ddd, *J* = 17.4, 10.7, 5.4 Hz, 1H), 7.22 – 7.24 (m, 2H), 7.30 – 7.35 (m, 2H) ppm.

^{13}C-NMR (100.6 MHz, $CDCl_3$): δ = 33.4, 39.4, 41.8, 68.2, 78.3, 114.9, 126.5, 126.8, 128.6, 139.1, 145.7 ppm.

ESI-HRMS: *m/z* calcd for $C_{13}H_{16}OBr$ $[M+H]^+$ 267.0380 found 267.0380.

Synthesis of syn- 4-(4-methoxyphenyl)-2-vinyltetrahydro-2H-pyran 20

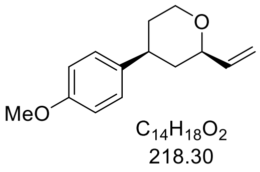

The reaction was performed according to **general procedure 8** with 3-(4-methoxyphenyl)hepta-5,6-dien-1-ol (66 mg, 0.3 mmol, 1.0 equiv.). The reaction mixture was filtered, concentrated and the **d.r.** ratio (**d.r. = 96/4**) was determined by ^{1}H-NMR spectroscopy of the crude product. After purification by flash chromatography on silica gel (pentane/ether 80/1 to 40/1) the product was obtained as colorless liquid (63 mg, 0.28 mmol, 96%).

Analytical Data[5]

^{1}H-NMR (400.1 MHz, $CDCl_3$): δ = 1.52 (ddd, *J* = 13.1, 12.3, 11.1 Hz, 1H), 1.72 – 1.79 (m, 2H), 1.89 (ddddd, *J* = 13.3, 3.8, 2.2, 1.6, 0.6 Hz, 1H), 2.74 – 2.84 (m, 1H), 3.59 – 3.67 (m, 1H), 3.80 (s, 3H), 3.91 – 3.98 (m, 1H), 4.14 – 4.20 (m, 1H), 5.12 (dd, *J* = 10.6, 1.5 Hz, 1H), 5.28 (dd, *J* = 17.3, 1.5 Hz, 1H), 5.90 (ddd, *J* = 17.4, 10.7, 5.4 Hz, 1H), 6.84 – 6.90 (m, 2H), 7.13 – 7.17 (m, 2H) ppm.

^{13}C-NMR (100.6 MHz, $CDCl_3$): δ = 33.6, 39.7, 40.9, 55.4, 68.3, 78.3, 114.0, 114.8, 127.7, 137.9, 139.1, 158.2 ppm.

APCI-HRMS: *m/z* calcd for $C_{14}H_{19}O_2$ $[M+H]^+$ 219.1385 found 219.1385.

Synthesis of syn- 4-(4-(methylthio)phenyl)-2-vinyltetrahydro-2H-pyran 21

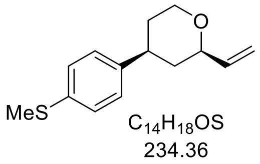

The reaction was performed according to **general procedure 8** with 3-(4-(methylthio)phenyl)hepta-5,6-dien-1-ol (70 mg, 0.3 mmol, 1.0 equiv.). The reaction mixture was filtered, concentrated and the **d.r.** ratio (**d.r. = 94/6**) was determined by ^{1}H-NMR spectroscopy of the crude product. After purification by flash chromatography on silica gel (pentane/ether 80/1 to 40/1) the product was obtained as colorless liquid (64 mg, 0.28 mmol, 92%).

Analytical Data[5]

^{1}H-NMR (400.1 MHz, $CDCl_3$): δ = .1.52 (ddd, *J* = 13.1, 12.3, 11.1 Hz, 1H), 1.72 – 1.79 (m, 2H), 1.85 – 1.92 (m, 1H), 2.47 (s, 3H), 2.74 – 2.85 (m, 1H), 3.58 – 3.67 (m, 1H), 3.94 (dddd, *J* = 11.1, 5.2, 2.5, 1.4 Hz, 1H), 4.14 – 4.20 (m, 1H), 5.13 (dd, *J* = 10.7, 1.5 Hz, 1H), 5.28 (dd, *J* = 17.3, 1.6 Hz, 1H), 5.89 (ddd, *J* = 17.4, 10.7, 5.4 Hz, 1H), 7.13 – 7.17 (m, 2H), 7.21 – 7.25 (m, 2H) ppm.

^{13}C-NMR (100.6 MHz, $CDCl_3$): δ =16.3, 33.4, 39.4, 41.3, 68.2, 78.3, 114.9, 127.4, 136.1, 139.0, 142.8 ppm.

ESI-HRMS: *m/z* calcd for $C_{14}H_{19}OS$ $[M+H]^+$ 235.1153 found 235.1151.

Synthesis of syn- 4-(4-methoxyphenyl)-3,3-dimethyl-2-vinyltetrahydro-2H-pyran 22

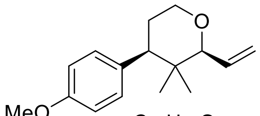

$C_{16}H_{22}O_2$
246.35

The reaction was performed according to **general procedure 8** with 3-(4-methoxyphenyl)-4,4-dimethylhepta-5,6-dien-1-ol (74 mg, 0.3 mmol, 1.0 equiv.). The reaction mixture was filtered, concentrated and the **d.r.** ratio (**d.r. = 75/25**) was determined by ^{1}H-NMR spectroscopy of the crude product. After purification by flash chromatography on silica gel (pentane/ether 80/1 to 40/1) the product was obtained as colorless liquid (71 mg, 0.28 mmol, 95%).

Analytical Data[6]

^{1}H-NMR (400.1 MHz, CDCl$_3$): δ = 0.72 (s, 3H), 0.80 (s, 3H), 1.48 (dddd, *J* = 13.5, 3.8, 2.5, 1.4 Hz, 1H), 2.29 (ddd, *J* = 13.3, 12.4, 4.9 Hz, 1H), 2.56 (dd, *J* = 13.1, 3.5 Hz, 1H), 3.59 – 3.67 (m, 2H), 3.80 (s, 3H), 4.17 (ddd, *J* = 11.3, 4.9, 1.4 Hz, 1H), 5.21 (ddd, *J* = 10.6, 2.0, 1.0 Hz, 1H), 5.27 (ddd, *J* = 17.3, 2.1, 1.3 Hz, 1H), 5.87 (ddd, *J* = 17.2, 10.6, 6.6 Hz, 1H), 6.82 – 6.87 (m, 2H), 7.07 – 7.11 (m, 2H) ppm.

^{13}C-NMR (100.6 MHz, CDCl$_3$): δ = 14.3, 24.7, 28.8, 37.5, 51.9, 55.3, 68.9, 88.1, 113.1, 117.3, 130.5, 133.8, 135.6, 158.3 ppm.

APCI-HRMS: *m/z* calcd for $C_{16}H_{26}ON$ $[M+NH_4]^+$ 264.1959 found 264.1958.

Synthesis of (2R,4S)-4-methyl-2-((E)-oct-1-en-1-yl)tetrahydro-2H-pyran 23

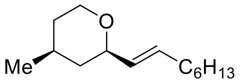

$C_{14}H_{26}O$
210.36

The reaction was performed according to **general procedure 8** with 3-methyltrideca-5,6-dien-1-ol (63 mg, 0.3 mmol, 1.0 equiv.). The reaction mixture was filtered, concentrated and the **d.r.** ratio (**d.r. = 95/5**) and the ***E/Z*** ratio (***E/Z* = 80/20**) was determined by ^{1}H-NMR spectroscopy of the crude product. After purification by flash chromatography on silica gel (pentane/ether 60/1 to 40/1) the product was obtained as colorless liquid (58 mg, 0.28 mmol, 93%).

Analytical Data[6]

^{1}H-NMR (400.1 MHz, CDCl$_3$): δ = 0.88 (d, *J* = 6.8 Hr, 3H), 0.94 (m, 3H), 0.97 – 1.09 (m, 1H), 1.25 – 1.31 (m, 6H), 1.33 – 1.43 (m, 2H), 1.50 (m$_c$, 1H), 1.52 – 1.56 (m, 1H), 1.59 – 1.67 (m, 2H), 1.98 – 2.05 (m, 2H), 3.45 (ddd, *J* = 12.4, 11.4, 2.3 Hz, 1H), 3.72 (dddt, *J* = 12.1, 7.1, 1.9, 0.8 Hz, 1H), 4.01 (ddd, *J* = 11.4, 4.6, 1.6 Hz, 1H), 5.46 (ddt, *J* = 15.5, 6.3, 1.5 Hz, 1H), 5.67 (dtd, *J* = 15.5, 6.7, 1.1 Hr, 1H) ppm.

^{13}C-NMR (100.6 MHz, CDCl$_3$): δ = 14.2, 22.4, 22.7, 29.0, 29.2, 30.4, 31.8, 32.4, 34.6, 41.0, 68.1, 78.2, 131.2, 132.0 ppm.

APCI-HRMS: *m/z* calcd for $C_{14}H_{27}O$ $[M+H]^+$ 365.3778 found 365.3774.

Synthesis of *syn*-4-dodecyl-2-(E)-oct-1-en-1-yl)tetrahydro-2H-pyran 24

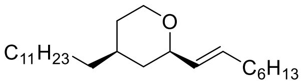

$C_{25}H_{48}O$
364.66

The reaction was performed according to **general procedure 9** with 3-(deca-2,3-dien-1-yl)pentadecan-1-ol (109 mg, 0.3 mmol, 1.0 equiv.). The reaction mixture was filtered, concentrated and the **d.r.** ratio (**d.r. = 94/6**) and the ***E/Z*** ratio (***E/Z* = 86/14**) was determined by ^{1}H-NMR spectroscopy of the crude product. After purification by flash chromatography on silica gel (pentane/ether 60/1 to 40/1) the product was obtained as colorless liquid (96 mg, 0.26 mmol, 88%).

Analytical Data[6]

^{1}H-NMR (400.1 MHz, $CDCl_3$): δ = 0.85 – 0.89 (m, 6H), 1.21 – 1.32 (m, 30H), 1.45 – 1.54 (m, 2H), 1.54 – 1.58 (m, 1H), 1.58 – 1.66 (m, 1H), 1.80 – 1.87 (m, 1H), 1.97 – 2.05 (m, 2H), 3.47 (td, *J* = 11.5, 2.6 Hz, 1H), 3.71 – 3.77 (m, 1H), 3.97 – 4.03 (m, 1H), 5.46 (dddd, *J* = 15.5, 6.2, 6.1, 1.4 Hz, 1H), 5.66 (ddd, *J* = 15.5, 6.7, 1.1 Hz, 1H).

^{13}C-NMR (100.6 MHz, $CDCl_3$): δ = 14.2, 14.2, 22.7, 22.8, 26.7, 26.8, 28.9, 29.1, 29.4, 29.5, 29.8, 29.8, 30.1, 31.8, 32.0, 33.4, 33.6, 33.7, 33.7, 34.6, 36.9, 61.3, 78.4, 131.3, 132 .ppm.

APCI-HRMS: *m/z* calcd for $C_{25}H_{49}O$ $[M+H]^+$ 365.3778 found 365.3774.

Synthesis of *syn*-4-neopentyl-2-(E)-oct-1-en-1-yl)tetrahydro-2H-pyran 25

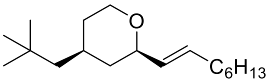

$C_{18}H_{34}O$
266.47

The reaction was performed according to **general procedure 9** with 3-neopentyltrideca-5,6-dien-1-ol (80 mg, 0.3 mmol, 1.0 equiv.). The reaction mixture was filtered, concentrated and the **d.r.** ratio (**d.r. = 92/8**) and the ***E/Z*** ratio (***E/Z* = 74/26**) was determined by ^{1}H-NMR spectroscopy of the crude product. After purification by flash chromatography on silica gel (pentane/ether 60/1 to 40/1) the product was obtained as colorless liquid (76 mg, 0.29 mmol, 95%).

Analytical Data[6]

^{1}H-NMR (400.1 MHz, $CDCl_3$): δ = 0.88 (d, *J* = 6.8 Hz, 3H), 0.91 (s, 9H), 1.07 (dd, *J* = 12.6, 11.2 Hz, 1H), 1.15 (dd, *J* = 4.9, 2.4 Hz, 2H), 1.23 – 1.31 (m, 7H), 1.33 – 1.39 (m, 2H), 1.53 – 1.61 (m, 2H), 1.66 (ddd, *J* = 12.0, 3.6, 1.6 Hz, 1H), 1.98 – 2.04 (m, 2H), 3.47 (ddd, *J* = 12.3, 11.4, 2.1 Hz, 1H), 3.73 (dddd, *J* = 11.3, 6.3, 2.1, 1.1 Hz, 1H), 3.98 (ddd, *J* = 11.5, 4.6, 1.6 Hz, 1H), 5.44 (ddd, *J* = 15.5, 6.3, 1.5 Hz, 1H), 5.67 (ddd, *J* = 15.5, 6.7, 1.1 Hz, 1H) ppm.

^{13}C-NMR (100.6 MHz, $CDCl_3$): δ = 14.2, 22.7, 29.0, 29.2, 30.3, 31.2, 31.8, 32.0, 32.4, 35.1, 41.5, 51.5, 68.1, 78.2, 131.2, 132.0 ppm.

ESI-HRMS: *m/z* calcd for $C_{19}H_{35}O$ $[M+H]^+$ 267.2668 found 267.2668.

Synthesis of *syn*-4 -cyclopropyl-2-(E)-oct-1-en-1-yl)tetrahydro-2H-pyran 26

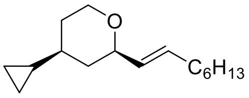

$C_{16}H_{28}O$
236.40

The reaction was performed according to **general procedure 9** with 3-cyclopropyltrideca-5,6-dien-1-ol (71 mg, 0.3 mmol, 1.0 equiv.). The reaction mixture was filtered, concentrated and the **d.r.** ratio (**d.r. = 93/7**) and the ***E/Z*** ratio (***E/Z* = 78/22**) was determined by ^{1}H-NMR spectroscopy of the crude product. After purification by flash chromatography on silica gel (pentane/ether 60/1 to 40/1) the product was obtained as colorless liquid (67 mg, 0.28 mmol, 94%).

Analytical Data[6]

^{1}H-NMR (400.1 MHz, $CDCl_3$): δ = 0.06 – 0.10 (m, 2H), 0.37 – 0.42 (m, 2H), 0.49 – 0.58 (m, 1H), 0.79 (ddd, *J* = 12.2, 8.7, 3.9 Hz, 1H), 0.86 – 0.89 (m, 3H), 1.19 (ddd, *J* = 13.2, 11.9, 11.2 Hz, 1H), 1.25 – 1.40 (m, 9H), 1.62 (ddddd, *J* = 13.3, 3.9, 1.9 Hz, 1H), 1.74 (ddd, *J* = 13.2, 3.8, 2.0 Hz, 1H), 1.98 – 2.05 (m, 2H), 3.39 (ddd, *J* = 12.5, 11.4, 2.3 Hz, 1H), 3.66 (dddd, *J* = 11.2, 6.2, 2.2, 1.1 Hz, 1H), 4.03 (ddd, *J* = 11.5, 4.6, 1.6 Hz, 1H), 5.47 (ddd, *J* = 15.5, 6.3, 1.4 Hz, 1H), 5.67 (ddd, *J* =1 5.5, 6.7, 1.1 Hz, 1H) ppm.

^{13}C-NMR (100.6 MHz, $CDCl_3$): δ = 2.8, 2.9, 14.2, 17.2, 22.7, 29.0, 29.2, 31.8, 32.4, 32.4, 38.7, 40.6, 68.1, 78.2, 131.1, 132.0 ppm.

APCI-HRMS: *m/z* calcd for $C_{16}H_{29}O$ $[M+H]^+$ 237.2210 found 237.2210.

Synthesis of *syn*-4-cyclohexyl-2-(E)-oct-1-en-1-yl)tetrahydro-2H-pyran 27

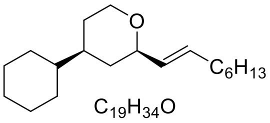

$C_{19}H_{34}O$
278.48

The reaction was performed according to **general procedure 9** with 3-cyclohexyltrideca-5,6-dien-1-ol (84 mg, 0.3 mmol, 1.0 equiv.). The reaction mixture was filtered, concentrated and the **d.r.** ratio (**d.r. = 91/9**) and the ***E/Z*** ratio (***E/Z* = 82/18**) was determined by ^{1}H-NMR spectroscopy of the crude product. After purification by flash chromatography on silica gel (pentane/ether 60/1 to 40/1) the product was obtained as colorless liquid (78 mg, 0.28 mmol, 93%).

Analytical Data[6]

^{1}H-NMR (400.1 MHz, $CDCl_3$): δ = 0.88 (t, *J* = 6.9 Hz, 3H), 0.95 (ddd, *J* = 14.2, 8.2, 4.0 Hz, 2H), 1.05 – 1.11 (m, 2H), 1.13 – 1.23 (m, 3H), 1.25 – 1.38 (m, 10H), 1.52 – 1.57 (m, 1H), 1.66 (dddd, *J* = 11.5, 5.0, 3.2, 2.0 Hz, 2H), 1.70 – 1.77 (m, 4H), 1.97 – 2.05 (m, 2H), 3.43 (ddd, *J* = 12.0, 11.4, 2.3 Hz, 1H), 3.70 (dddd, *J* = 11.1, 6.3, 2.2, 1.0 Hz, 1H), 4.04 (ddd, *J* = 11.2, 4.3, 1.7 Hz, 1H), 5.46 (ddd, *J* = 15.5, 6.3, 1.5 Hz, 1H), 5.67 (ddd, *J* = 15.5, 6.7, 1.1 Hz, 1H) ppm.

^{13}C-NMR (100.6 MHz, $CDCl_3$): δ = 14.2, 22.7, 26.7, 26.9, 29.0, 29.2, 29.8, 30.0, 30.1, 31.8, 32.4, 36.3, 40.9, 43.0, 68.3, 78.4, 131.3, 131.9 ppm.

APCI-HRMS: *m/z* calcd for $C_{19}H_{35}O$ $[M+H]^+$ 279.2687 found 279.2687.

Synthesis of *syn*-2-(E)-oct-1-en-1-yl)-4-(3-phenylpropyl)tetrahydro-2H-pyran 28

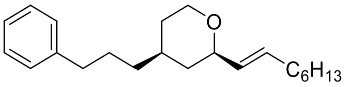

$C_{22}H_{34}O$
314.51

The reaction was performed according to **general procedure 9** with 3-(3-phenylpropyl)trideca-5,6-dien-1-ol (94 mg, 0.3 mmol, 1.0 equiv.). The reaction mixture was filtered, concentrated and the **d.r.** ratio (**d.r. = 92/8**) and the ***E/Z*** ratio (***E/Z* = 79/21**) was determined by ^{1}H-NMR spectroscopy of the crude product. After purification by flash chromatography on silica gel (pentane/ether 60/1 to 40/1) the product was obtained as colorless liquid (93 mg, 0.29 mmol, 98%).

Analytical Data[6]

^{1}H-NMR (400.1 MHz, $CDCl_3$): δ = 0.86 – 0.90 (m, 3H), 1.01 (dd, *J* = 13.1, 11.4 Hz, 1H), 1.22 – 1.39 (m, 11H), 1.48 – 1.60 (m, 3H), 1.63 – 1.69 (m, 2H), 1.97 – 2.05 (m, 2H), 2.56 – 2.64 (m, 2H), 3.45 (ddd, *J* = 12.6, 11.4, 2.1 Hz, 1H), 3.71 (dddd, *J* = 11.2, 6.3, 2.2, 1.1 Hz, 1H), 4.02 (ddd, *J* = 11.4, 4.6, 1.5 Hz, 1H), 5.45 (ddd, *J* = 15.5, 6.3, 1.5 Hz, 1H), 5.67 (ddd, *J* = 15.5, 6.7, 1.1 Hz, 1H), 7.14 – 7.20 (m, 3H), 7.26 – 7.30 (m, 2H) ppm.

^{13}C-NMR (100.6 MHz, $CDCl_3$): δ = 14.2, 22.7, 28.4, 29.0, 29.2, 31.8, 32.4, 32.7, 35.3, 36.2, 36.8, 39.1, 68.1, 78.2, 125.8, 128.3, 128.4, 131.1, 132.0, 142.7 ppm.

APCI-HRMS: *m/z* calcd for $C_{22}H_{38}ON$ $[M+NH_4]^+$ 332.2948 found 332.2947.

Synthesis of *syn*-2-(E)-oct-1-en-1-yl)-4-phenyltetrahydro-2H-pyran 29

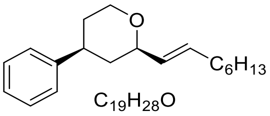

$C_{19}H_{28}O$
272.43

The reaction was performed according to **general procedure 9** with 3-phenyltrideca-5,6-dien-1-ol (82 mg, 0.3 mmol, 1.0 equiv.). The reaction mixture was filtered, concentrated and the **d.r.** ratio (**d.r. = 94/6**) and the ***E/Z*** ratio (***E/Z* = 83/17**) was determined by ^{1}H-NMR spectroscopy of the crude product. After purification by flash chromatography on silica gel (pentane/ether 60/1 to 40/1) the product was obtained as colorless liquid (78 mg, 0.29 mmol, 96%).

Analytical Data[6]

^{1}H-NMR (400.1 MHz, $CDCl_3$): δ = 0.85 – 0.90 (m, 3H), 1.28 (dddd, *J* = 10.2, 4.7, 2.7, 1.6 Hz, 6H), 1.34 – 1.40 (m, 2H), 1.52 – 1.61 (m, 1H), 1.73 – 1.78 (m, 2H), 1.84 – 1.90 (m, 1H), 1.99 – 2.08 (m, 2H), 2.77 – 2.87 (m, 1H), 3.59 – 3.67 (m, 1H), 3.90 (dddd, *J* = 10.9, 6.1, 2.1, 1.1 Hz, 1H), 4.16 (ddd, *J* = 11.5, 4.0, 2.3 Hz, 1H), 5.52 (ddd, *J* = 15.5, 6.2, 1.4 Hz, 1H), 5.72 (ddd, *J* = 15.5, 6.7, 1.1 Hz, 1H), 7.20 – 7.24 (m, 3H), 7.29 – 7.34 (m, 2H) ppm.

^{13}C-NMR (100.6 MHz, $CDCl_3$): δ = 14.2, 22.7, 29.0, 29.2, 31.8, 32.4, 33.4, 39.9, 41.8, 68.2, 78.3, 126.4, 126.8, 128.6, 130.8, 132.4, 145.8 ppm.
APCI-HRMS: *m/z* calcd for $C_{19}H_{29}O$ $[M+H]^+$ 273.2215 found 273.2216.

Synthesis of *syn*-4-(naphthalen-2-yl)-2-(E)-oct-1-en-1-yl)tetrahydro-2H-pyran 30

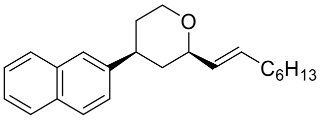

$C_{23}H_{30}O$
322.49

The reaction was performed according to **general procedure 9** with 3-(naphthalen-2-yl)trideca-5,6-dien-1-ol (97 mg, 0.3 mmol, 1.0 equiv.). The reaction mixture was filtered, concentrated and the **d.r.** ratio (**d.r. = 95/5**) and the ***E/Z*** ratio (***E/Z* = 81/19**) was determined by ^{1}H-NMR spectroscopy of the crude product. After purification by flash chromatography on silica gel (pentane/ether 60/1 to 40/1) the product was obtained as colorless liquid (89 mg, 0.28 mmol, 92%).

Analytical Data[6]
^{1}H-NMR (400.1 MHz, $CDCl_3$): δ = 0.87 – 0.90 (m, 3H), 1.26 – 1.33 (m, 6H), 1.35 – 1.44 (m, 2H), 1.68 (ddd, *J* = 13.1, 12.2, 11.0 Hz, 1H), 1.81 – 1.89 (m, 2H), 1.97 (ddd, *J* = 13.2, 3.5, 1.9 Hz, 1H), 2.01 – 2.09 (m, 2H), 2.99 (dd, *J* = 11.7, 4.1 Hz, 1H), 3.65 – 3.74 (m, 1H), 3.96 (dddd, *J* = 11.1, 6.2, 2.1, 1.1 Hz, 1H), 4.20 (ddd, *J* = 11.5, 4.2, 2.0 Hz, 1H), 5.55 (ddd, *J* = 15.5, 6.2, 1.5 Hz, 1H), 5.76 (ddd, *J* = 15.5, 6.7, 1.1 Hz, 1H), 7.37 – 7.49 (m, 3H), 7.65 (dd, *J* = 1.8, 0.8 Hz, 1H), 7.79 – 7.83 (m, 3H) ppm.
^{13}C-NMR (100.6 MHz, $CDCl_3$): δ = 14.2, 22.7, 29.0, 29.2, 31.8, 32.4, 33.4, 39.8, 41.9, 68.3, 78.4, 124.8, 125.4, 125.8, 126.1, 127.7, 127.7, 128.2, 130.8, 132.5, 143.2 ppm.
APCI-HRMS: *m/z* calcd for $C_{23}H_{31}O$ $[M+H]^+$ 323.2369 found 323.2368.

Synthesis of *syn*-4-([1,1'-biphenyl]-4-yl)-2-((E)-oct-1-en-1-yl)tetrahydro-2H-pyran 31

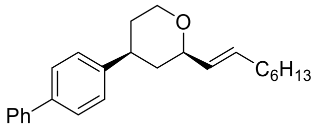

$C_{25}H_{32}O$
348.53

The reaction was performed according to **general procedure 9** with 3-([1,1'-biphenyl]-4-yl)trideca-5,6-dien-1-ol (105 mg, 0.3 mmol, 1.0 equiv.). The reaction mixture was filtered, concentrated and the **d.r.** ratio (**d.r. = 94/6**) and the ***E/Z*** ratio (***E/Z* = 77/23**) was determined by ^{1}H-NMR spectroscopy of the crude product. After purification by flash chromatography on silica gel (pentane/ether 60/1 to 40/1) the product was obtained as colorless liquid (96 mg, 0.28 mmol, 92%).

Analytical Data[6]

^{1}H-NMR (400.1 MHz, $CDCl_3$): δ = 0.89 (t, *J* = 6.8 Hz, 3H), 1.26 – 1.31 (m, 6H), 1.36 – 1.41 (m, 2H), 1.56 – 1.65 (m, 1H), 1.76 – 1.83 (m, 2H), 1.91 (ddd, *J* = 11.5, 3.8, 1.9 Hz, 1H), 2.00 – 2.08 (m, 2H), 2.87 (ddd, *J* = 16.1, 10.5, 3.9 Hz, 1H), 3.62 – 3.69 (m, 1H), 3.93 (dddd, *J* = 11.0, 6.2, 2.2 Hz, 1.1, 1H), 4.18 (ddd, *J* = 11.5, 4.1, 2.4 Hz, 1H), 5.53 (ddd, *J* = 15.5, 6.2, 1.5 Hz, 1H), 5.74 (ddd, *J* = 15.5, 6.7, 1.1 Hz, 1H), 7.29 – 7.32 (m, 2H), 7.33 – 7.36 (m, 1H), 7.41 – 7.46 (m, 2H), 7.54 – 7.60 (m, 4H) ppm.

^{13}C-NMR (100.6 MHz, $CDCl_3$): δ = 14.2, 22.7, 29.0, 29.2, 31.8, 32.4, 33.4, 39.9, 41.5, 68.2, 78.3, 127.1, 127.2, 127.2, 127.3, 128.8, 130.7, 132.5, 139.4, 141.1, 144.9 ppm.

APCI-HRMS: *m/z* calcd for $C_{23}H_{31}O$ $[M+H]^+$ 349.2526 found 349.2524.

Synthesis of *syn*-2-((E)-oct-1-en-1-yl)-4-(p-tolyl)tetrahydro-2H-pyran 32

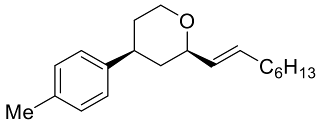

$C_{20}H_{30}O$
286.46

The reaction was performed according to **general procedure 7** with 3-(p-tolyl)trideca-5,6-dien-1-ol (86 mg, 0.3 mmol, 1.0 equiv.). The reaction mixture was filtered, concentrated and the **d.r.** ratio (**d.r. = 94/6**) and the ***E/Z*** ratio (***E/Z* = 83/17**) was determined by ^{1}H-NMR spectroscopy of the crude product. After purification by flash chromatography on silica gel (pentane/ether 60/1 to 40/1) the product was obtained as colorless liquid (83 mg, 0.29 mmol, 97%).

Analytical Data[6]

^{1}H-NMR (400.1 MHz, $CDCl_3$): δ = 0.88 (d, *J* = 6.8 Hz, 3H), 1.25 – 1.31 (m, 6H), 1.33 – 1.40 (m, 2H), 1.51 – 1.57 (m, 1H), 1.71 – 1.79 (m, 2H), 1.82 – 1.89 (m, 1H), 1.99 – 2.07 (m, 2H), 2.33 (s, 3H), 2.72 – 2.83 (m, 1H), 3.58 – 3.66 (m, 1H), 3.89 (dddd, *J* = 11.0, 6.1, 2.2, 1.1 Hz, 1H), 4.15 (ddd, *J* = 11.5, 4.0, 2.4 Hz, 1H), 5.51 (ddd, *J* = 15.5, 6.2, 1.4 Hz, 1H), 5.72 (ddd, *J* = 15.5, 6.7, 1.1 Hz, 1H), 7.12 (m_c, 4H) ppm.

^{13}C-NMR (100.6 MHz, CDCl$_3$): δ = 14.2, 21.6, 22.7, 28.9, 29.2, 31.8, 32.4, 33.4, 39.9, 41.8, 68.3, 123.8, 127.1, 127.7, 128.5, 130.8, 132.4, 145.8 ppm.

APCI-HRMS: *m/z* calcd for $C_{20}H_{31}O$ $[M+H]^+$ 287.2376 found 287.2377.

Synthesis of *syn*-2-((E)-oct-1-en-1-yl)-4-(m-tolyl)tetrahydro-2H-pyran 33

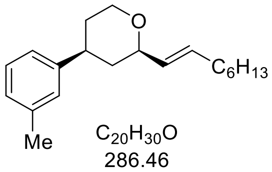

$C_{20}H_{30}O$
286.46

The reaction was performed according to **general procedure 9** with 3-(m-tolyl)trideca-5,6-dien-1-ol (86 mg, 0.3 mmol, 1.0 equiv.). The reaction mixture was filtered, concentrated and the **d.r.** ratio (**d.r. = 94/6**) and the ***E/Z*** ratio (***E/Z* = 80/20**) was determined by ^{1}H-NMR spectroscopy of the crude product. After purification by flash chromatography on silica gel (pentane/ether 60/1 to 40/1) the product was obtained as colorless liquid (80 mg, 0.28 mmol, 93%).

Analytical Data[6]

^{1}H-NMR (400.1 MHz, CDCl$_3$): δ = 0.86 – 0.90 (m, 3H), 1.26 – 1.31 (m, 6H), 1.33 – 1.40 (m, 2H), 1.52 – 1.59 (m, 1H), 1.73 – 1.77 (m, 2H), 1.83 – 1.89 (m, 1H), 2.03 (ddd, *J* = 8.7, 6.7, 0.8 Hz, 2H), 2.35 (8, 3H), 2.73 – 2.82 (m, 1H), 3.58 – 3.67 (m, 1H), 3.89 (dddd, *J* = 11.1, 6.2, 2.1, 1.0 Hr, 1H), 4.15 (ddd, *J* = 11.5, 4.1, 2.1 Hz, 1H), 5.52 (ddd, *J* = 15.5, 6.2, 1.4 Hz, 1H), 5.72 (ddd, *J* = 15.6, 6.7, 1.1 Hz, 1H), 7.03 (ddd, *J* = 8.3, 1.2, 0.6 Hz, 3H), 7.21 (dd, *J* = 7.3, 1.2 Hz, 1H).

^{13}C-NMR (100.6 MHz, CDCl$_3$): δ = δ = 14.2, 21.6, 22.7, 28.9, 29.2, 31.8, 32.4, 33.4, 39.9, 41.8, 68.3, 123.8, 127.1, 127.7, 128.5, 130.8, 132.4, 145.8 ppm

APCI-HRMS: *m/z* calcd for $C_{20}H_{31}O$ $[M+H]^+$ 287.2369 found 287.2365.

Synthesis of *syn*-2-((E)-oct-1-en-1-yl)-4-(o-tolyl)tetrahydro-2H-pyran 34

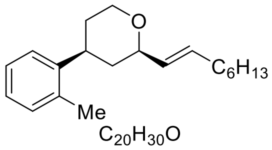

$C_{20}H_{30}O$
286.46

The reaction was performed according to **general procedure 9** with 3-(o-tolyl)trideca-5,6-dien-1-ol (86 mg, 0.3 mmol, 1.0 equiv.). The reaction mixture was filtered, concentrated and the **d.r.** ratio (**d.r. = 93/7**) and the ***E/Z*** ratio (***E/Z* = 81/19**) was determined by ^{1}H-NMR spectroscopy of the crude product. After purification by flash chromatography on silica gel (pentane/ether 60/1 to 40/1) the product was obtained as colorless liquid (78 mg, 0.27 mmol, 91%).

Analytical Data[6]

^{1}H-NMR (400.1 MHz, CDCl$_3$): δ = 0.86 – 0.90 (m, 3H), 1.27 (ddd, *J* = 5.1, 2.0, 1.3 Hz, 6H), 1.34 – 1.42 (m, 2H), 1.53 – 1.61 (m, 1H), 1.67 (dddd, *J* = 13.3, 4.1, 2.1 Hz, 1H), 1.76 – 1.82 (m, 2H), 2.00 – 2.07 (m,

2H), 2.36 (s, 3H), 3.04 (dd, *J* = 12.0, 3.6 Hz, 1H), 3.62 – 3.69 (m, 1H), 3.93 (dddd, *J* = 11.0, 6.1, 2.2, 1.0 Hz, 1H), 4.17 (ddd, *J* = 11.4, 4.4, 1.7 Hz, 1H), 5.52 (ddd, *J* = 15.5, 6.2, 1.5 Hz, 1H), 5.73 (ddd, *J* = 15.5, 6.7, 1.1 Hz, 1H), 7.08 – 7.24 (m, 4H) ppm.

^{13}C-NMR (100.6 MHz, $CDCl_3$): δ = 14.2, 19.4, 22.7, 29.0, 29.2, 31.8, 32.4, 32.7, 37.5, 39.1, 68.5, 78.5, 125.6, 126.1, 126.4, 130.5, 130.8, 132.4, 135.1, 143.6 ppm.

APCI-HRMS: *m/z* calcd for $C_{20}H_{31}O$ $[M+H]^+$ 287.2369 found 287.2367.

Synthesis of *syn*-4-mesityl-2-((E)-oct-1-en-1-yl)tetrahydro-2H-pyran 35

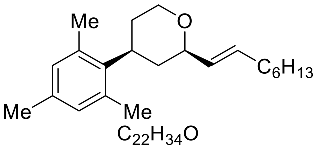

$C_{22}H_{34}O$
314.51

The reaction was performed according to **general procedure 9** with 3-mesityltrideca-5,6-dien-1-ol (94 mg, 0.3 mmol, 1.0 equiv.). The reaction mixture was filtered, concentrated and the **d.r.** ratio (**d.r. = 95/5**) and the ***E/Z*** ratio (***E/Z* = 80/20**) was determined by ^{1}H-NMR spectroscopy of the crude product. After purification by flash chromatography on silica gel (pentane/ether 60/1 to 40/1) the product was obtained as colorless liquid (83 mg, 0.26 mmol, 88%).

Analytical Data[6]

^{1}H-NMR (400.1 MHz, $CDCl_3$): δ = 0.86 – 0.90 (m, 3H), 1.26 – 1.32 (m, 6H), 1.37 (m, 2H), 1.51 – 1.66 (m, 2H), 1.97 – 2.09 (m, 3H), 2.24 (s, 3H), 2.40 (s, 6H), 3.29 (ddd, *J* = 12.7, 3.8 Hz, 1H), 3.57 – 3.65 (m, 1H), 3.83 – 3.91 (m, 1H), 4.17 (dd, *J* = 11.4, 4.5 Hz, 1H), 5.48 – 5.56 (m, 1H), 5.67 – 5.76 (m, 1H), 6.83 (m_c, 2H) ppm.

^{13}C-NMR (100.6 MHz, $CDCl_3$): δ = 14.2, 20.7, 21.8, 22.7, 28.9, 29.2, 29.8, 31.8, 32.4, 36.2, 38.4, 69.1, 79.3, 130.9, 132.3, 135.4, 136.3, 137.8 ppm.

APCI-HRMS: *m/z* calcd for $C_{22}H_{35}O$ $[M+H]^+$ 315.2682 found 315.2681.

Synthesis of *syn*-2-((E)-oct-1-en-1-yl)-4-(4-vinylphenyl)tetrahydro-2H-pyran 36

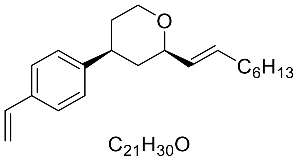

$C_{21}H_{30}O$
298.47

The reaction was performed according to **general procedure 9** with 3-(4-vinylphenyl)trideca-5,6-dien-1-ol (89 mg, 0.3 mmol, 1.0 equiv.). The reaction mixture was filtered, concentrated and the **d.r.** ratio (**d.r. = 94/6**) and the ***E/Z*** ratio (***E/Z* = 78/22**) was determined by ^{1}H-NMR spectroscopy of the crude product. After purification by flash chromatography on silica gel (pentane/ether 60/1 to 40/1) the product was obtained as colorless liquid (82 mg, 0.28 mmol, 92%).

Analytical Data[6]

^{1}H-NMR (400.1 MHz, $CDCl_3$): δ = 0.85 – 0.88 (m, 3H), 1.24 – 1.28 (m, 6H), 1.34 – 1.38 (m, 2H), 1.42 – 1.46 (m, 2H), 1.72 – 1.76 (m, 2H), 1.80 – 1.89 (m, 1H), 1.99 – 2.05 (m, 2H), 2.72 – 2.83 (m, 1H), 3.58 – 3.64 (m, 1H), 3.84 – 3.94 (m, 1H), 4.14 (ddd, *J* = 11.4, 4.1, 2.3 Hz, 1H), 5.50 (dddd, *J* = 15.5, 6.2, 1.5 Hz, 1H), 5.67 – 5.76 (m, 1H), 6.28 – 6.44 (m, 1H), 7.11 – 7.25 (m, 4H), 7.28 – 7.32 (m, 1H) ppm.

^{13}C-NMR (100.6 MHz, $CDCl_3$): δ = 14.2, 21.3, 29.2, 31.8, 33.3, 33.4, 39.7, 39.9, 41.3, 42.2, 68.2, 78.3, 126.4, 126.9, 127.0, 127.5, 128.1, 132.5, 134.9, 135.8, 143.7,144.7 ppm.

APCI-HRMS: *m/z* calcd for $C_{21}H_{34}ON$ $[M+ NH_4]^+$ 316.2635 found 316.2632.

Synthesis of *syn*-4-(4-bromophenyl)-2-((E)-oct-1-en-1-yl)tetrahydro-2H-pyran 37

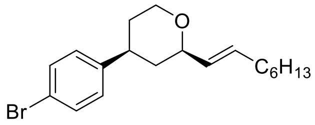

$C_{19}H_{27}BrO$
351.33

The reaction was performed according to **general procedure 9** with 3-(4-bromophenyl)trideca-5,6-dien-1-ol (105 mg, 0.3 mmol, 1.0 equiv.). The reaction mixture was filtered, concentrated and the **d.r.** ratio (**d.r. = 90/10**) and the ***E/Z*** ratio (***E/Z* = 71/29**) was determined by ^{1}H-NMR spectroscopy of the crude product. After purification by flash chromatography on silica gel (pentane/ether 60/1 to 40/1) the product was obtained as colorless liquid (83 mg, 0.24 mmol, 79%).

Analytical Data[6]

^{1}H-NMR (400.1 MHz, $CDCl_3$): δ = 0.85 – 0.90 (m, 3H), 1.25 – 1.31 (m, 6H), 1.34 – 1.40 (m, 2H), 1.53 – 1.60 (m, 1H), 1.74 – 1.78 (m, 2H), 1.83 – 1.91 (m, 1H), 1.98 – 2.07 (m, 2H), 2.77 – 2.87 (m, 1H), 3.59 – 3.67 (m, 1H), 3.90 (dddd, *J* = 10.9, 6.2, 2.2 Hz, 1.1, 1H), 4.16 (ddd, *J* = 11.5, 4.0, 2.3 Hz, 1H), 5.51 (ddd, *J* = 15.5, 6.2, 1.4 Hz, 1H), 5.72 (ddd, *J* = 15.5, 6.7, 1.1 Hz, 1H), 7.21 – 7.23 (m, 2H), 7.29 – 7.34 (m, 2H) ppm.

^{13}C-NMR (100.6 MHz, $CDCl_3$): δ = 14.2, 22.7, 29.0 29.2, 31.8, 32.4, 33.4, 39.9, 41.8, 68.2, 78.3, 126.4, 126.8, 128.6, 130.8, 132.4, 145.8 ppm.

APCI-HRMS: *m/z* calcd for $C_{19}H_{31}ON$ $[M+NH_4]^+$ 370.1569 found 370.1570.

Synthesis of *syn*-4-(4-methoxyphenyl)-2-((E)-oct-1-en-1-yl)tetrahydro-2H-pyran 4

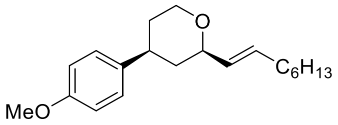

$C_{20}H_{30}O_2$
302.46

The reaction was performed according to **general procedure 9** with 3-(4-bromophenyl)trideca-5,6-dien-1-ol (91 mg, 0.3 mmol, 1.0 equiv.). The reaction mixture was filtered, concentrated and the **d.r.** ratio (**d.r. = 95/5**) and the ***E/Z*** ratio (***E/Z* = 85/15**) was determined by ^{1}H-NMR spectroscopy of the crude product. After purification by flash chromatography on silica gel (pentane/ether 60/1 to 40/1) the product was obtained as colorless liquid (86 mg, 0.29 mmol, 95%).

Analytical Data[6]

^{1}H-NMR (500 MHz, Toluene-*d*$_8$) δ = 0.88 (t, *J* = 6.9 Hz, 3H), 1.22 – 1.28 (m, 6H), 1.33 – 1.37 (m, 2H), 1.43 – 1.47 (m, 1H), 1.48 – 1.51 (m, 1H), 1.60 (ddd, *J* = 13.0, 12.2, 4.5 Hz, 1H), 1.73 (ddd, *J* = 13.0, 3.9, 2.1 Hz, 1H), 1.99 – 2.04 (m, 2H), 2.09 (dd, *J* = 4.4, 2.2 Hz, 2H), 2.50 (dd, *J* = 12.3, 3.9 Hz, 1H), 3.37 (s, 3H), 3.78 (dddd, *J* = 11.0, 5.4, 1.2 Hz, 1H), 4.02 (ddd, *J* = 11.3, 4.5, 1.6 Hz, 1H), 5.61 (ddd, *J* = 15.5, 5.4, 1.4 Hz, 1H), 5.73 – 5.80 (m, 1H), 6.75 – 6.77 (m, 2H), 6.94 – 6.96 (m, 2H) ppm.

^{13}C-NMR (126 MHz, Toluene-*d*8): δ = 14.3, 23.1, 29.4, 29.8, 32.2, 32.9, 34.2, 40.8, 41.4, 54.7, 68.1, 78.2, 114.2, 130.6, 132.1, 137.4, 138.3, 158.7 ppm.

APCI-HRMS: *m/z* calcd for $C_{20}H_{30}O_2$ $[M+H]^+$ 302.2246 found 302.2246.

Synthesis of *syn*-4-(4-(methylthio)phenyl)-2-((E)-oct-1-en-1-yl)tetrahydro-2H-pyran 38

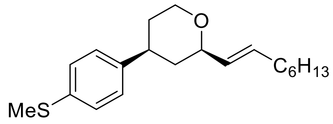

$C_{20}H_{30}OS$
318.52

The reaction was performed according to **general procedure 9** with 3-(4-(methylthio)phenyl)trideca-5,6-dien-1-ol (96 mg, 0.3 mmol, 1.0 equiv.). The reaction mixture was filtered, concentrated and the **d.r.** ratio (**d.r. = 94/6**) and the ***E/Z*** ratio (***E/Z* = 83/17**) was determined by ^{1}H-NMR spectroscopy of the crude product. After purification by flash chromatography on silica gel (pentane/ether 60/1 to 40/1) the product was obtained as colorless liquid (91 mg, 0.29 mmol, 96%).

Analytical Data[6]

^{1}H-NMR (400.1 MHz, CDCl$_3$): δ = 0.88 (t, *J* = 6.8 Hz 3H), 1.23 - 1.27 (m, *J* = 3.4, 1.6 Hz, 6H), 1.34 – 1.40 (m, 2H), 1.47 – 1.54 (m, 1H), 1.71 – 1.77 (m, 2H), 1.81 – 1.87 (m, 1H), 2.00 – 2.06 (m, 2H), 2.47 (s, 3H), 2.77 (ddtd *J* = 11.9, 8.7, 4.3 Hz, 1H), 3.58 – 3.65 (m, 1H), 3.88 (dddd, *J* = 11.0, 6.2, 2.2, 1.1 Hz, 1H), 4.11 – 4.18 (m, 1H), 5.50 (ddd, *J* = 15.5, 6.2, 1.5 Hz, 1H), 5.72 (ddd, *J* = 15.5, 6.7, 1.1 Hz, 1H), 7.13 – 7.16 (m, 2H), 7.21 – 7.24 (m, 2H) ppm.

^{13}C-NMR (100.6 MHz, CDCl$_3$): δ = 14.2, 16.4, 22.7, 28.9, 29.2, 31.8, 32.4, 33.4, 39.8, 41.3, 68.2, 78.3, 127.4, 130.7, 132.5, 136.0, 143.0bppm.

ESI-HRMS: *m/z* calcd for $C_{15}H_{22}O_3NNaS$ [M+]$^+$ found

Synthesis of *syn*-2-((E)-oct-1-en-1-yl)-4-(4-(trifluoromethyl)phenyl)tetrahydro-2H-pyran 39

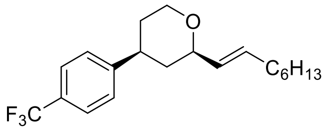

$C_{20}H_{27}F_3O$
340.43

The reaction was performed according to **general procedure 9** with 3-(4-(trifluoromethyl)phenyl)trideca-5,6-dien-1-ol (96 mg, 0.3 mmol, 1.0 equiv.). The reaction mixture was filtered, concentrated and the **d.r.** ratio (**d.r. = 94/6**) and the ***E/Z*** ratio (***E/Z* = 81/19**) was determined by ^{1}H-NMR spectroscopy of the crude product. After purification by flash chromatography on silica gel (pentane/ether 60/1 to 40/1) the product was obtained as colorless liquid (95 mg, 0.28 mmol, 93%).

Analytical Data[6]

^{1}H-NMR (400.1 MHz, CDCl$_3$): δ = 0.88 (t, *J* = 6.7 Hz, 3H), 1.26 – 1.31 (m, 6H), 1.35 – 1.41 (m, 2H), 1.54 – 1.59 (m, 1H), 1.76 (ddd, *J* = 6.1, 4.7, 1.7 Hz, 2H), 1.85 – 1.89 (m, 1H), 2.01 – 2.07 (m, 2H), 2.85 – 2.93 (m, 1H), 3.60 – 3.67 (m, 1H), 3.91 (dddd, *J* = 10.8, 6.1, 2.1, 1.1 Hz, 1H), 4.17 (ddd, *J* = 11.6, 4.1, 2.4 Hz, 1H), 5.51 (ddd, *J* = 15.4, 6.2, 1.4 Hz, 1H), 5.73 (ddd, *J* = 15.5, 6.7, 1.1 Hz, 1H), 7.32 – 7.35 (m, 2H), 7.56 – 7.58 (m, 2H) ppm.

^{13}C-NMR (100.6 MHz, CDCl$_3$): δ = 14.2, 22.7, 28.9, 29.2, 31.8, 32.4, 33.1, 39.5, 41.7, 68.0, 74.1, 78.2, 125.6, 125.6, 127.2, 130.5, 132.7, 149.7 ppm.

APCI-HRMS: *m/z* calcd for $C_{20}H_{31}OF_3N$ [M+NH$_4$]$^+$ 358.2358 found 358.2358.

Synthesis of *syn*-2,2-dimethyl-6-((E)-oct-1-en-1-yl)-4-(4-(trifluoromethyl)phenyl)tetrahydro-2H-pyran 40

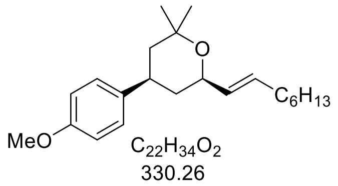

The reaction was performed according to **general procedure 9** with 4-(4-methoxyphenyl)-2-methyltetradeca-6,7-dien-2-ol (96 mg, 0.3 mmol, 1.0 equiv.). The reaction mixture was filtered, concentrated and the **d.r.** ratio (**d.r. = 85/15**) and the ***E/Z*** ratio (***E/Z* = 70/30**) was determined by ^{1}H-NMR spectroscopy of the crude product. After purification by flash chromatography on silica gel (pentane/ether 60/1 to 40/1) the product was obtained as colorless liquid (90 mg, 0,27 mmol, 91%).

Analytical Data[6]

^{1}H-NMR (400.1 MHz, CDCl$_3$): δ = 0.88 (t, *J* = 6.8 Hz, 3H), 1.24 – 1.29 (m, 5H), 1.30 (s, 3H), 1.33 (s, 3H), 1.33 – 1.41 (m, 3H), 1.41 – 1.58 (m, 2H), 1.66 (ddd, *J* = 13.1, 3.8, 1.9 Hz, 1H), 1.79 (ddd, *J* = 12.9, 3.9, 2.1 Hz, 1H), 1.95 – 2.10 (m, 2H), 2.93 (ddd, *J* = 12.6, 3.7 Hz, 1H), 3.79 (s, 3H), 4.15 (dddd, *J* = 11.4, 6.5, 2.3, 0.9 Hz, 1H), 5.49 (ddd, *J* = 15.4, 6.5, 1.4 Hz, 1H), 5.68 (ddd, *J* = 15.5, 6.6, 1.0 Hz, 1H), 6.83 – 6.88 (m, 2H), 7.11 – 7.16 (m, 2H) ppm.

^{13}C-NMR (100.6 MHz, CDCl$_3$): δ = 14.2, 22.6, 22.7, 29.0, 29.1, 31.8, 32.0, 32.4, 36.9, 39.9, 44.1, 55.4, 71.8, 72.4, 114.0, 127.7, 131.6, 132.2, 138.2, 158.1 ppm.

APCI-HRMS: *m/z* calcd for $C_{22}H_{35}O_2$ [M+H]$^+$ 331.2629 found 331.2629.

Synthesis of *syn*-2-((E)-2-cyclohexylvinyl)-4-(4-methoxyphenyl)tetrahydro-2H-pyran 41

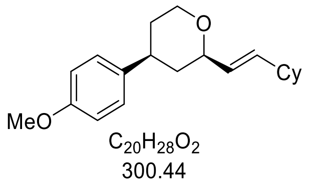

The reaction was performed according to **general procedure 9** with 7-cyclohexyl-3-(4-methoxyphenyl)hepta-5,6-dien-1-ol (90 mg, 0.3 mmol, 1.0 equiv.). The reaction mixture was filtered, concentrated and the **d.r.** ratio (**d.r. = 93/7**) and the ***E/Z*** ratio (***E/Z* = 88/12**) was determined by ^{1}H-NMR spectroscopy of the crude product. After purification by flash chromatography on silica gel (pentane/ether 60/1 to 40/1) the product was obtained as colorless liquid (70 mg, 0.23 mmol, 78%).

Analytical Data[6]

^{1}H-NMR (400.1 MHz, CDCl$_3$): δ = 1.02 – 1.32 (m, 6H), 1.51 (ddd, *J* = 13.1, 12.2, 11.0 Hz, 1H), 1.64 (dtt, *J* = 12.4, 3.3, 1.7 Hz, 1H), 1.68 – 1.74 (m, 5H), 1.82 – 1.88 (m, 1H), 1.95 (dddd, *J* = 11.5, 8.4, 5.0, 2.1 Hz, 1H), 2.76 (ddd, *J* = 16.0, 8.7, 3.8 Hz, 1H), 3.57 – 3.65 (m, 1H), 3.79 (s, 3H), 3.85 – 3.91 (m, 1H), 4.12 – 4.18 (m, 1H), 5.47 (ddd, *J* = 15.7, 6.1, 1.3 Hz, 1H), 5.67 (ddd, *J* = 15.7, 6.5, 1.1 Hz, 1H), 6.84 – 6.88 (m, 2H), 7.13 – 7.16 (m, 2H) ppm.

^{13}C-NMR (100.6 MHz, CDCl$_3$): δ = 26.1, 26.3, 32.9, 33.6, 40.2, 40.4, 41.0, 55.4, 68.3, 78.5, 114.0, 127.7, 128.4, 137.9, 138.1, 158.2 ppm.

APCI-HRMS: *m/z* calcd for $C_{20}H_{29}O_2$ [M+H]$^+$ 301.2162 found 301.2169.

Synthesis of *syn*-4-(4-methoxyphenyl)-2-((E)-4-phenylbut-1-en-1-yl)tetrahydro-2H-pyran 42

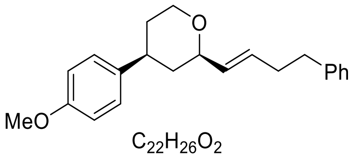

$C_{22}H_{26}O_2$
322.45

The reaction was performed according to **general procedure 9** with 3-(4-methoxyphenyl)-9-phenylnona-5,6-dien-1-ol (97 mg, 0.3 mmol, 1.0 equiv.). The reaction mixture was filtered, concentrated and the **d.r.** ratio (**d.r. = 93/7**) and the ***E/Z*** ratio (***E/Z* = 78/22**) was determined by ^{1}H-NMR spectroscopy of the crude product. After purification by flash chromatography on silica gel (pentane/ether 60/1 to 40/1) the product was obtained as colorless liquid (89 mg, 0.28 mmol, 93%).

Analytical Data[6]

^{1}H-NMR (400.1 MHz, CDCl$_3$): δ = 1.43 – 1.56 (m, 1H), 1.73 (ddd, *J* = 9.6, 5.2, 3.9 Hz, 2H), 1.83 (dddd, *J* = 13.1, 3.3, 1.4 Hz, 1H), 2.33 – 2.39 (m, 2H), 2.70 (dd, *J* = 8.6, 7.1 Hz, 2H), 2.73 – 2.80 (m, 1H), 3.58 – 3.64 (m, 1H), 3.80 (s, 3H), 3.86 – 3.92 (m, 1H), 4.12 – 4.20 (m, 1H), 5.57 (dddd, *J* = 15.5, 6.1, 1.4 Hz, 1H), 5.77 (ddd, *J* = 15.5, 6.6 Hz, 1.1, 1H), 6.84 – 6.89 (m, 2H), 7.12 – 7.20 (m, 5H), 7.26 – 7.29 (m, 2H) ppm.

^{13}C-NMR (100.6 MHz, CDCl$_3$): δ = 33.7, 34.3, 35.7, 40.0, 40.9, 55.4, 68.3, 78.2, 114.0, 125.9, 127.7, 128.4, 128.5, 131.2, 131.5, 138.0, 141.9, 158.2 ppm.

APCI-HRMS: *m/z* calcd for $C_{22}H_{27}O_2$ [M+H]$^+$ 323.2006 found 323.2008.

Synthesis of *syn*-4-(4-methoxyphenyl)-2-((E)-3-phenylprop-1-en-1-yl)tetrahydro-2H-pyran 43

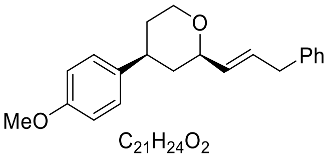

$C_{21}H_{24}O_2$
308.42

The reaction was performed according to **general procedure 9** with 3-(4-methoxyphenyl)-8-phenylocta-5,6-dien-1-ol (93 mg, 0.3 mmol, 1.0 equiv.). The reaction mixture was filtered, concentrated and the **d.r.** ratio (**d.r. = 93/7**) and the ***E/Z*** ratio (***E/Z* = 80/20**) was determined by ^{1}H-NMR spectroscopy of the crude product. After purification by flash chromatography on silica gel (pentane/ether 60/1 to 40/1) the product was obtained as colorless liquid (83 mg, 0.27 mmol, 90%).

Analytical Data[6]

^{1}H-NMR (400.1 MHz, CDCl$_3$): 1.44 – 1.57 (m, 1H), 1.72 – 1.77 (m, 2H), 1.84 – 1.89 (m, 1H), 2.76 (ddd, *J* = 12.2, 8.4, 4.4 Hz, 1H), 3.35 – 3.40 (m, 2H), 3.56 – 3.66 (m, 1H), 3.79 (s, 3H), 3.90 – 3.96 (m, 1H), 4.11 – 4.17 (m, 1H), 5.54 – 5.62 (m, 1H), 5.88 (ddd, *J* = 15.5, 6.7, 1.2 Hz, 1H), 7.12 – 7.22 (m, 6H), 7.26 – 7.32 (m, 3H) ppm.

^{13}C-NMR (100.6 MHz, CDCl$_3$): δ = 33.6, 38.8, 40.1, 40.9, 55.4, 68.3, 78.1, 114.0, 114.0, 126.1, 127.7, 127.7, 128.5, 128.7, 130.6, 132.3, 138.0, 140.1, 158.2 ppm.

APCI-HRMS: *m/z* calcd for $C_{21}H_{25}O_2$ [M+H]$^+$ 309.1849 found 309.1854.

Synthesis of *syn*-4-(4-methoxyphenyl)-2-((E)-5-(methylthio)pent-1-en-1-yl)tetrahydro-2H-pyran 44

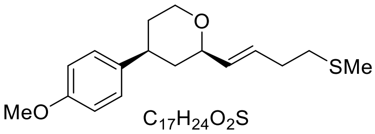

$C_{17}H_{24}O_2S$
292.44

The reaction was performed according to **general procedure 9** with 3-(4-methoxyphenyl)-8-phenylocta-5,6-dien-1-ol (91 mg, 0.3 mmol, 1.0 equiv.). The reaction mixture was filtered, concentrated and the **d.r.** ratio (**d.r. = 95/5**) and the ***E/Z*** ratio (***E/Z* = 89/11**) was determined by ^{1}H-NMR spectroscopy of the crude product. After purification by flash chromatography on silica gel (pentane/ether 60/1 to 40/1) the product was obtained as colorless liquid (81 mg, 0.27 mmol, 89%).

Analytical Data[6]

^{1}H-NMR (400.1 MHz, CDCl$_3$): δ = 1.43 – 1.56 (m, 1H), 1.71 – 1.76 (m, 2H), 1.83 – 1.89 (m, 1H), 2.10 (s, 3H), 2.30 – 2.37 (m, 2H), 2.52 – 2.57 (m, 2H), 2.71 – 2.80 (m, 1H), 3.58 – 3.65 (m, 1H), 3.79 (s, 3H), 3.90 (dddd, *J* = 11.0, 5.8, 2.1, 1.0 Hz, 1H), 4.12 – 4.17 (m, 1H), 5.56 – 5.63 (m, 1H), 5.75 (ddd, *J* = 15.5, 6.6, 1.2 Hz, 1H), 6.84 – 6.88 (m, 2H), 7.12 – 7.15 (m, 2H) ppm.

^{13}C-NMR (100.6 MHz, CDCl$_3$): δ = 15.6, 32.2, 33.6, 33.9, 40.0, 40.9, 55.4, 68.3, 78.0, 78.0, 114.0, 127.7, 129.7, 132.4, 137.9, 158.2 ppm.

APCI-HRMS: *m/z* calcd for $C_{17}H_{24}O_2S$ [M+H]$^+$ 293.1575 found 293.1575.

6.1 Large scale catalysis

Synthesis of syn- 4-(4-methoxyphenyl)-2-vinyltetrahydro-2H-pyran 20

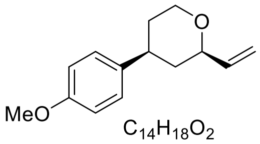

$C_{14}H_{18}O_2$
218.29

A screw-cap flask was flame-dried, cooled to room temperature under vacuum and backfilled with argon using a standard SCHLENK line apparatus. The screw-cap flask was charged with 3-(4-methoxyphenyl)hepta-5,6-dien-1-ol (306 mg, 1.40 mmol, 1.0 equiv.) the flask was evacuated for 15 min and backfilled with argon three times. Then $[Rh(COD)Cl]_2$ (17.3 mg, 0.035 mmol, 2.5 mol%), dppf (38.8 mg, 0.700 mmol, 5.0 mol%) and diphenyl phosphate (70 mg, 0.28 mmol, 20 mol%) was added under a flow of argon followed by freshly distilled DCM (5 mL). The flask was sealed and stirred at room temperature overnight. The reaction mixture was filtered, concentrated and the **d.r.** ratio (**d.r. = 95/5**) was determined by ^{1}H-NMR spectroscopy of the crude product. After purification by flash chromatography on silica gel (DCM) the product was obtained as a colorless solid (287 mg, 1.32 mmol, 94%).

Analytical Data[5]

^{1}H-NMR (400.1 MHz, $CDCl_3$): δ = 1.52 (ddd, *J* = 13.1, 12.3, 11.1 Hz, 1H), 1.72 – 1.79 (m, 2H), 1.89 (ddddd, *J* = 13.3, 3.8, 2.2, 1.6, 0.6 Hz, 1H), 2.74 – 2.84 (m, 1H), 3.59 – 3.67 (m, 1H), 3.80 (s, 3H), 3.91 – 3.98 (m, 1H), 4.14 – 4.20 (m, 1H), 5.12 (dd, *J* = 10.6, 1.5 Hz, 1H), 5.28 (dd, *J* = 17.3, 1.5 Hz, 1H), 5.90 (ddd, *J* = 17.4, 10.7, 5.4 Hz, 1H), 6.84 – 6.90 (m, 2H), 7.13 – 7.17 (m, 2H) ppm.

^{13}C-NMR (100.6 MHz, $CDCl_3$): δ = 33.6, 39.7, 40.9, 55.4, 68.3, 78.3, 114.0, 114.8, 127.7, 137.9, 139.1, 158.2 ppm.

APCI-HRMS: *m/z* calcd for $C_{14}H_{19}O_2$ $[M+H]^+$ 219.1385 found 219.1385

Synthesis of syn- 4-(4-methoxyphenyl)-2-vinyltetrahydro-2H-pyran 4

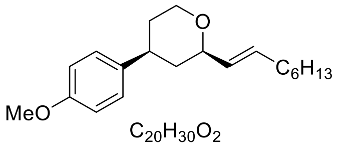

$C_{20}H_{30}O_2$
302.45

A screw-cap flask was flame-dried, cooled to room temperature under vacuum and backfilled with argon using a standard SCHLENK line apparatus. The screw-cap flask was charged with 3-(4-methoxyphenyl)trideca-5,6-dien-1-ol (423 mg, 1.40 mmol, 1.0 equiv.) the flask was evacuated for 15 min and backfilled with argon three times. Then $[Rh(COD)Cl]_2$ (17.3 mg, 0.035 mmol, 2.5 mol%), dppf (38.8 mg, 0.700 mmol, 5.0 mol%) and PTSA (80 mg, 0.42 mmol, 30 mol%) was added under a flow of argon followed by freshly distilled PhF (5 mL). The flask was sealed and stirred at room temperature overnight. The reaction mixture was filtered, concentrated and the **d.r.** ratio (**d.r. = 95/5**) and ***E/Z*** ratio (**E/Z = 83/17**) was determined by ^{1}H-NMR spectroscopy of the crude product. After purification by flash chromatography on silica gel (DCM) the product was obtained as a colorless solid (394 mg, 1.3 mmol, 93%).

Analytical Data[6]

^{1}H-NMR (500 MHz, Toluene-*d*$_8$) δ = 0.88 (t, *J* = 6.9 Hz, 3H), 1.22 – 1.28 (m, 6H), 1.33 – 1.37 (m, 2H), 1.43 – 1.47 (m, 1H), 1.48 – 1.51 (m, 1H), 1.60 (ddd, *J* = 13.0, 12.2, 4.5 Hz, 1H), 1.73 (ddd, *J* = 13.0, 3.9, 2.1 Hz, 1H), 1.99 – 2.04 (m, 2H), 2.09 (dd, *J* = 4.4, 2.2 Hz, 2H), 2.50 (dd, *J* = 12.3, 3.9 Hz, 1H), 3.37 (s, 3H), 3.78 (dddd, *J* = 11.0, 5.4, 1.2 Hz, 1H), 4.02 (ddd, *J* = 11.3, 4.5, 1.6 Hz, 1H), 5.61 (ddd, *J* = 15.5, 5.4, 1.4 Hz, 1H), 5.73 – 5.80 (m, 1H), 6.75 – 6.77 (m, 2H), 6.94 – 6.96 (m, 2H) ppm.

^{13}C-NMR (126 MHz, Toluene-*d*8): δ = 14.3, 23.1, 29.4, 29.8, 32.2, 32.9, 34.2, 40.8, 41.4, 54.7, 68.1, 78.2, 114.2, 130.6, 132.1, 137.4, 138.3, 158.7 ppm.

APCI-HRMS: *m/z* calcd for $C_{20}H_{30}O_2$ $[M+H]^+$ 302.2246 found 302.2246

6.2 Catalysis of 1, 3-substituded δ-allenols

Synthesis of 4-(4-methoxyphenyl)-3,3-dimethyl-2-vinyltetrahydro-2H-pyran 45A

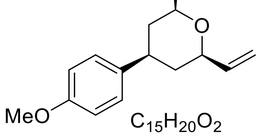

$C_{15}H_{20}O_2$
232.32

The reaction was performed according to **general procedure 6** with *syn*-4-(4-methoxyphenyl)octa-6,7-dien-2-ol (70 mg, 0.3 mmol, 1.0 equiv.). The reaction mixture was filtered, concentrated and the **d.r.** ratio (**d.r. = 95/5**) was determined by ^{1}H-NMR spectroscopy of the crude product. After purification by flash chromatography on silica gel (pentane/ether 80/1 to 40/1) the product was obtained as colorless liquid (65 mg, 0.28 mmol, 93%).

Analytical Data

^{1}H-NMR (500.1 MHz, $CDCl_3$): δ = 1.28 (d, *J* = 6.1 Hz, 3H), 1.33 – 1.50 (m, 2H), 1.83 (dddd, *J* = 6.3, 13.1, 3.9, 2.0 Hz, 2H), 2.80 (dddd, *J* = 12.3, 12.2, 3.7, 3.8 Hz, 1H), 3.62 – 3.70 (m, 1H), 3.79 (s, 3H), 3.99 (dddt, *J* = 11.0, 5.7, 2.4, 1.4 Hz, 1H), 5.13 (dd, *J* = 10.6, 1.4 Hz, 1H), 5.29 (dd, *J* = 17.3, 1.5 Hz, 1H), 5.92 (ddd, *J* = 17.3, 10.6, 5z.7 Hz, 1H), 6.84 – 6.88 (m, 2H), 7.10 – 7.16 (m, 2H) ppm.

^{13}C-NMR (125.6 MHz, $CDCl_3$): δ = 22.1, 40.9, 40.9, 55.4, 73.7, 78.2, 114.0, 115.1, 127.7, 137.9, 139.2, 158.1 ppm.

APCI-HRMS: *m/z* calcd for $C_{15}H_{21}O_2$ $[M+H]^+$ 233.1536 found 233.1534.

Synthesis of 4-(4-methoxyphenyl)-3,3-dimethyl-2-vinyltetrahydro-2H-pyran 49A, 49B

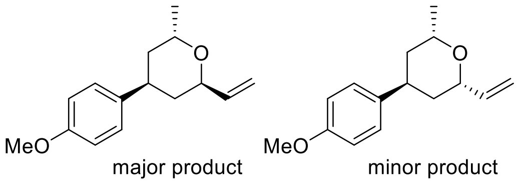

$C_{15}H_{20}O_2$
232.32

The reaction was performed according to **general procedure 8** with *anti*-4-(4-methoxyphenyl)octa-6,7-dien-2-ol (70 mg, 0.3 mmol, 1.0 equiv.). The reaction mixture was filtered, concentrated and the **d.r.** ratio (**d.r. = 95/5**) was determined by ^{1}H-NMR spectroscopy of the crude product. After purification by flash chromatography on silica gel (pentane/ether 80/1 to 40/1) the product was obtained as colorless liquid (71 mg, 0.28 mmol, 92%).

Analytical Data (major compound) **49A**

^{1}H-NMR (400.1 MHz, $CDCl_3$): 1.40 (s, 1H), 1.43 – 1.56 (m, 1H), 1.64 (dddd, *J* = 13.2, 3.5, 2.0, 1.4 Hz, 1H), 1.72 – 1.78 (m, 1H), 1.82 – 1.88 (m, 1H), 1.89 – 1.97 (m, 1H), 3.01 (dddd, *J* = 12.6, 12.5, 3.7, 3.6 Hz 1H), 3.79 (s, 3H), 4.26 ddddd, *J* = 11.1, 5.4, 2.6, 1.4, 1.4 Hz 1H), 4.41 (dddd, *J* = 7.3, 7.2, 5.8, 5.6 Hz, 1H), 5.11 (ddd, *J* = 10.5, 1.5 Hz, 1H), 5.26 (ddd, *J* = 17.3, 1.5 Hz, 1H), 5.87 (ddd, *J* = 17.3, 10.5, 5.6 Hz, 1H), 6.85 – 6.88 (m, 2H), 7.13 – 7.17 (m, 2H) ppm.

^{13}C-NMR (100.6 MHz, $CDCl_3$): δ = 17.3, 34.9, 37.3, 39.8, 55.3, 68.3, 69.4, 70.0, 78.3, 114.0, 114.8, 139.6, 158.1 ppm.

APCI-HRMS: *m/z* calcd for $C_{15}H_{21}O_2$ $[M+H]^+$ 233.1536 found 233.1534.

Analytical Data (minor compound) **49B**

^{1}H-NMR (400.1 MHz, CDCl$_3$): 1.21 (d, *J* = 6.1 Hz, 3H), 1.59 (s, 1H), 1.79 (dddd, *J* = 44.9, 13.9, 11.6, 5.5 Hz, 3H), 2.08 (dddd, *J* = 18.4, 13.9, 2.2 Hz, 2H), 3.25 – 3.30 (m, 1H), 3.72 – 3.79 (m, 1H), 3.81 (s, 3H), 4.04 – 4.12 (m, 1H), 5.11 (dd, *J* = 10.6, 1.4 Hz, 1H), 5.25 (ddd, *J* = 17.3, 2.0, 1.5 Hz, 1H), 5.87 (ddd, *J* = 17.4, 10.6, 5.8 Hz, 1H), 6.87 – 6.91 (m, 2H), 7.23 – 7.26 (m, 2H) ppm.

^{13}C-NMR (100.6 MHz, CDCl$_3$): δ = 22.4, 34.2, 35.5, 37.3, 55.3, 68.9, 73.7, 115.1, 127.7, 128.8, 135.9, 139.6, 157.6 ppm.

APCI-HRMS: *m/z* calcd for $C_{15}H_{21}O_2$ [M+H]$^+$ 233.1536 found 233.1534.

Synthesis of 4-(4-methoxyphenyl)-2-phenyl-6-vinyltetrahydro-2H-pyran 46A

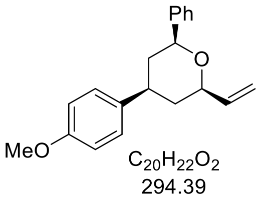

The reaction was performed according to **general procedure 8** with *syn*-3-(4-methoxyphenyl)-1-phenylhepta-5,6-dien-1-ol (88 mg, 0.3 mmol, 1.0 equiv.). The reaction mixture was filtered, concentrated and the **d.r.** ratio (**d.r. = 95/5**) was determined by ^{1}H-NMR spectroscopy of the crude product. After purification by flash chromatography on silica gel (pentane/ether 80/1 to 40/1) the product was obtained as colorless liquid (85 mg, 0.29 mmol, 96%).

Analytical Data

^{1}H-NMR (500.1 MHz, CDCl$_3$): δ = 1.58 (dd, *J* = 12.8, 11.6 Hz, 1H), 1.64 – 1.77 (m, 1H), 2.03 (dddd, *J* = 52.8, 13.2, 4.0, 2.1 Hz, 2H), 3.00 (dddd, *J* = 12,4, 12.3, 4.5, 3.7 Hz, 1H), 3.79 (s, 3H), 4.20 (ddd, *J* = 10.9, 5.3, 1.8 Hz, 1H), 4.60 (dd, *J* = 11.2, 2.2 Hz, 1H), 5.16 (ddd, *J* = 10.7, 1.5, 0.7 Hz, 1H), 5.37 (ddd, *J* = 17.4, 1.6, 0.7 Hz, 1H), 6.01 (dddd, *J* = 17.2, 10.6, 5.3, 0.6 Hz, 1H), 6.84 – 6.89 (m, 2H), 7.15 – 7.18 (m, 2H), 7.24 – 7.28 (m, 1H), 7.32 – 7.37 (m, 2H), 7.43 (ddd, *J* = 8.3, 1.4, 0.7 Hz, 2H) ppm.

^{13}C-NMR (11 0.6 MHz, CDCl$_3$): δ = 39.2, 41.2, 41.3, 55.4, 78.5, 79.5, 114.0, 114.8, 125.9, 127.4, 127.7, 128.3, 137.6, 139.1, 142.9, 158.2 ppm.

ESI-HRMS: *m/z* calcd for $C_{15}H_{22}O_3NNaS$ [M+]$^+$ found

Synthesis of 4-(4-methoxyphenyl)-2-phenyl-6-vinyltetrahydro-2H-pyran 50A, 50B

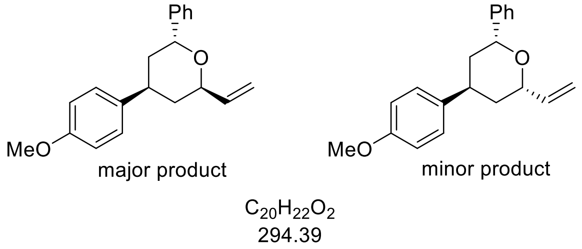

The reaction was performed according to **general procedure 8** with *anti*-3-(4-methoxyphenyl)-1-phenylhepta-5,6-dien-1-ol (88 mg, 0.3 mmol, 1.0 equiv.). The reaction mixture was filtered, concentrated and the **d.r.** ratio (**d.r. = 85/15**) was determined by ^{1}H-NMR spectroscopy of the crude product. After purification by flash chromatography on silica gel (pentane/ether 80/1 to 40/1) the product was obtained as colorless liquid (71 mg, 0.28 mmol, 94%).

Analytical Data (major compound) **50A**

^{1}H-NMR (400.1 MHz, CDCl$_3$): δ = 1.59 – 1.67 (m, 1H), 1.80 (ddd, *J* = 12.8, 4.0, 2.2 Hz, 1H), 2.19 (ddd, *J* = 13.8, 12.8, 5.5 Hz, 1H), 2.53 (dddd, *J* = 13.9, 3.9, 3.5, 1.8 Hz, 1H), 2.89 (dddd, *J*= 12.9, 12.5, 3.9, 3.5 Hz, 1H), 3.81 (s, 3H), 4.10 (dddd, *J* = 11.1, 5.4, 2.7, 1.5Hz , 1H), 5.15 (dd, *J* = 10.6, 1.5 Hz, 1H), 5.26 – 5.35 (m, 2H), 5.92 (ddd, *J* = 17.3, 10.6, 5.3 Hz, 1H), 6.85 – 6.92 (m, 2H), 7.15 – 7.20 (m, 2H), 7.30 (dddd, *J* = 8.5, 6.7, 2.2, 1.2 Hz, 1H), 7.42 (dd, *J* = 8.5, 7.0 Hz, 2H), 7.50 (m_c 2H) ppm.

^{13}C-NMR (100.6 MHz, CDCl$_3$): δ = 34.0, 35.6, 39.6, 55.4, 71.1, 73.7, 114.1, 114.9, 126.7, 126.9, 127.7, 128.8, 137.8, 139.2, 140.4, 158.2 ppm.

ESI-HRMS: *m/z* calcd for $C_{15}H_{22}O_3NNaS$ [M+]$^+$ found

Analytical Data[3] (minor product) **50B**

^{1}H-NMR (400.1 MHz, CDCl$_3$): δ = 1.96 (ddd, *J* = 14.0, 11.7, 5.4 Hz, 1H), 2.06 (ddd, *J* = 14.1, 11.8, 5.3, 1H), 2.24 (ddd, *J* = 14.0, 2.5, 2.3 Hz, 1H), 2.33 (ddd, *J*=14.1, 2.6 2.2 Hz, 1H), 3.39 (m_c, 1H), 3.84 (s, 3H), 4.26 – 4.32 (m, 1H), 4.68 (dd, *J* = 11.8, 2.2 Hz, 1H), 5.14 (dd, *J* = 10.6, 1.5 Hz, 1H), 5.32 (dd, *J* = 17.3, 1.6 Hz, 1H), 5.95 (ddd, *J* = 17.3, 10.6, 5.3 Hz, 1H), 6.92 – 6.97 (m, 2H), 7.24 – 7.26 (m, 1H), 7.31 – 7.36 (m, 4H), 7.39 (ddd, *J* = 7.8, 1.5, 0.7 Hz, 2H) ppm.

^{13}C-NMR (100.6 MHz, CDCl$_3$): δ = 34.4, 35.4, 37.9, 55.4, 74.0, 74.9, 114.0, 114.9, 125.9, 127.3, 128.3, 128.8, 135.3, 139.5, 143.3, 157.7 ppm.

ESI-HRMS: *m/z* calcd for $C_{15}H_{22}O_3NNaS$ [M+]$^+$ found

Synthesis of *syn*-4-(4-methoxyphenyl)-2-methyl-6-((E)-oct-1-en-1-yl)tetrahydro-2H-pyran 47

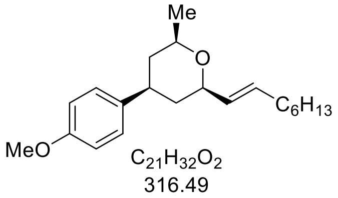

The reaction was performed according to **general procedure 9** with *syn*-4-(4-methoxyphenyl)tetradeca-6,7-dien-2-ol (95 mg, 0.3 mmol, 1.0 equiv.). The reaction mixture was filtered, concentrated and the **d.r.** ratio (**d.r. = 95/5**) and the ***E/Z*** ratio (***E/Z* =**) was determined by ^{1}H-NMR spectroscopy of the crude product. After purification by flash chromatography on silica gel (pentane/ether 60/1 to 40/1) the product was obtained as colorless liquid (86 mg, 0.27 mmol, 91%).

Analytical Data[3]

^{1}H-NMR (400.1 MHz, CDCl$_3$): δ = 0.85 – 0.90 (m, 3H), 1.24 – 1.31 (m, 9H), 1.32 – 1.41 (m, 3H), 1.41 – 1.50 (m, 1H), 1.75 – 1.85 (m, 2H), 1.98 – 2.07 (m, 2H), 2.78 (dddd, *J* = 12.5, 12.3, 4.1, 3.8 Hz, 1H), 3.64 (m$_c$, 1H), 3.79 (s, 3H), 3.91 – 3.97 (m, 1H), 5.53 (ddd, *J* = 15.5, 6.5, 1.5 Hz, 1H), 5.71 (ddd, *J* = 15.5, 6.7, 1.1 Hz, 1H), 6.83 – 6.88 (m, 2H), 7.11 – 7.15 (m, 2H) ppm.

^{13}C-NMR (100.6 MHz, CDCl$_3$): δ = 14.2, 22.2, 22.7, 29.0, 29.1, 31.8, 32.5, 39.5, 40.9, 55.4, 73.6, 78.3, 114.0, 127.7, 130.9, 132.5, 138.1, 158.1 ppm.

ESI-HRMS: *m/z* calcd for $C_{15}H_{22}O_3NNaS$ [M+]$^+$ found

Synthesis of *syn*-4-(4-methoxyphenyl)-2-methyl-6-((E)-oct-1-en-1-yl)tetrahydro-2H-pyran 51A, 51B

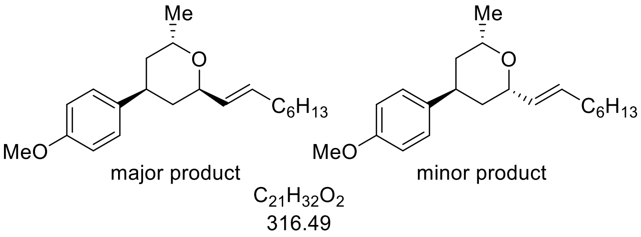

The reaction was performed according to **general procedure 9** with *trans*-4-(4-methoxyphenyl)tetradeca-6,7-dien-2-ol (95 mg, 0.3 mmol, 1.0 equiv.). The reaction mixture was filtered, concentrated and the **d.r.** ratio (**d.r. = 80/20**) was determined by ^{1}H-NMR spectroscopy of the crude product. After purification by flash chromatography on silica gel (pentane/ether 60/1 to 40/1) the product was obtained as colorless liquid (86 mg, 0.27 mmol, 92%).

Analytical Data[3] (major product) **51A**

^{1}H-NMR (400.1 MHz, CDCl$_3$): δ = 0.87 (t, *J* = 5.4 Hz, 3H), 1.25 – 1.30 (m, 10H), 1.35 – 1.38 (m, 2H), 1.51 (ddd, *J* = 13.1, 12.3, 11.0 Hz, 2H), 1.71 – 1.74 (m, 2H), 1.82 – 1.86 (m, 1H), 1.99 – 2.03 (m, 2H), 2.70 – 2.80 (m, 1H), 3.58 – 3.65 (m, 1H), 3.85 – 3.91 (m, 1H), 4.10 – 4.17 (m, 1H), 5.47 – 5.53 (m, 1H), 5.71 (ddd, *J* = 15.5, 6.7, 1.1 Hz, 1H), 6.83 – 6.88 (m, 2H), 7.13 – 7.15 (m, 2H) ppm.

^{13}C-NMR (100.6 MHz, CDCl$_3$): δ = 14.2, 22.7, 29.0, 29.2, 32.4, 33.6, 40.1, 40.9, 55.4, 68.3, 78.4, 114.0, 127.7, 128.7, 130.8, 132.4, 138.1, 158.1 ppm.

ESI-HRMS: *m/z* calcd for $C_{15}H_{22}O_3NNaS$ $[M+]^+$ found

Analytical Data[3] (minor product) **51B**

^{1}H-NMR (500.1 MHz, CDCl$_3$): δ = 0.88 (t, *J* = 5.2 Hz, 3H), 1.20 (d, *J* = 6.2, 3H), 1.25 – 1.30 (m, 7H), 1.34 – 1.39 (m, 2H), 1.73 (ddd, *J* = 14.1, 11.4, 5.6 Hz, 1H), 1.79 – 1.88 (m, 1H), 2.03 (ddd, *J* = 9.2, 4.9, 1.7 Hz, 3H), 3.25 (m$_c$, 1H), 3.73 (ddd, *J* = 12.5, 6.2, 1.9 Hz, 1H), 3.81 (s, 3H), 4.01 – 4.10 (m, 1H), 5.48 (ddd, *J* = 15.5, 6.6, 1.5 Hz, 1H), 5.67 (ddd, *J* = 15.5, 6.7, 1.1 Hz, 1H), 6.87 – 6.90 (m, 2H), 7.22 – 7.26 (m, 2H) ppm.

^{13}C-NMR (100.6 MHz, CDCl$_3$): δ = 14.2, 22.4, 22.7, 29.1, 32.5, 34.3, 35.8, 37.4, 40.9, 55.3, 68.8, 73.7, 78.3, 113.8, 114.0, 127.7, 128.8, 131.2, 132.5 , 136.1, 157.6 ppm.

ESI-HRMS: *m/z* calcd for $C_{15}H_{22}O_3NNaS$ $[M+]^+$ found

Synthesis of *4*-(4-methoxyphenyl)-2-((E)-oct-1-en-1-yl)-6-phenyltetrahydro-2H-pyran 48A

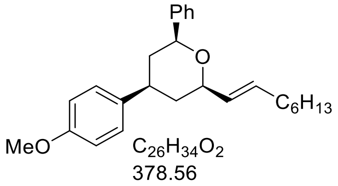

The reaction was performed according to **general procedure 9** with *syn*-3-(4-methoxyphenyl)-1-phenylocta-5,6-dien-1-o (114 mg, 0.3 mmol, 1.0 equiv.). The reaction mixture was filtered, concentrated and the **d.r.** ratio (**d.r. = 95/5**) was determined by ^{1}H-NMR spectroscopy of the crude product. After purification by flash chromatography on silica gel (pentane/ether 60/1 to 40/1) the product was obtained as colorless liquid (102 mg, 0.27 mmol, 90%).

Analytical Data[3]

^{1}H-NMR (400.1 MHz, CDCl$_3$): δ = 0.88 (t, *J* = 4.8 Hz, 3H), 1.26 – 1.31 (m, 7H), 1.35 – 1.40 (m, 2H), 1.58 – 1.72 (m, 2H), 1.92 (ddd, *J* = 13.2, 4.0, 2.0 Hz, 1H), 2.03 – 2.07 (m, 2H), 2.97 (dddd, *J* = 12.8, 12.3, 4.0, 3.7 Hz, 1H), 3.79 (s, 3H), 4.14 (dddd, *J* = 11.1, 6.0, 2.2, 1.1 Hz, 1H), 4.57 (dd, *J* = 11.2, 2.2 Hz, 1H), 5.61 (ddd, *J* = 15.5, 6.1, 1.4 Hz, 1H), 5.76 (ddd, *J* = 15.5, 6.6, 1.1 Hz, 1H), 6.84 – 6.87 (m, 2H), 7.14 – 7.18 (m, 2H), 7.22 – 7.26 (m, 1H), 7.31 – 7.34 (m, 2H), 7.39 – 7.43 (m, 2H) ppm.

^{13}C-NMR (100.6 MHz, CDCl$_3$): δ = 14.2, 22.7, 29.0, 29.2, 31.8, 32.5, 39.7, 41.3, 55.4, 78.7, 79.6, 114.0, 126.1, 127.4, 127.7, 128.3, 128.3, 130.8, 132.1, 137.7, 143.0, 158.2 ppm

ESI-HRMS: *m/z* calcd for $C_{15}H_{22}O_3NNaS$ $[M+]^+$ found

Synthesis of 4-(4-methoxyphenyl)-2-((E)-oct-1-en-1-yl)-6-phenyltetrahydro-2H-pyran 52A, 52B

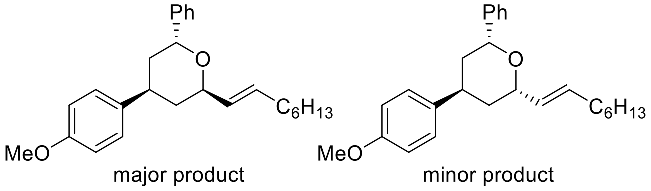

$C_{26}H_{34}O_2$
378.56

The reaction was performed according to **general procedure 9** with *trans*-3-(4-methoxyphenyl)-1-phenylocta-5,6-dien-1-o (114 mg, 0.3 mmol, 1.0 equiv.). The reaction mixture was filtered, concentrated and the **d.r.** ratio (**d.r. = 73/27**) was determined by ^{1}H-NMR spectroscopy of the crude product. After purification by flash chromatography on silica gel (pentane/ether 60/1 to 40/1) the product was obtained as colorless liquid (99 mg, 0.26 mmol, 88%).

Analytical Data[3] (major product) **52A**

^{1}H-NMR (400.1 MHz, CDCl$_3$): δ = 0.87 (t, *J* = 7.1 Hz, 3H), 1.26 – 1.29 (m, 6H), 1.36 – 1.41 (m, 2H), 1.59 – 1.68 (m, 1H), 1.74 – 1.80 (m, 1H), 2.02 – 2.07 (m, 2H), 2.14 – 2.22 (m, 1H), 2.50 (ddd, *J* = 13.9, 3.5, 1.8 Hz, 1H), 2.86 (dddd, *J* = 13.3, 12.5, 3.9, 3.5 Hz, 1H), 3.80 (s, 3H), 4.04 – 4.09 (m, 1H), 5.28 – 5.31 (m, 1H), 5.54 (ddd, *J* = 15.5, 6.1, 1.5 Hz, 1H), 5.70 (ddd, *J* = 15.5, 6.7, 1.1 Hz, 1H), 6.86 – 6.89 (m, 2H), 7.15 – 7.18 (m, 2H), 7.27 – 7.31 (m, 1H), 7.40 – 7.44 (m, 2H), 7.48 – 7.50 (m, 2H) ppm.

^{13}C-NMR (100.6 MHz, CDCl$_3$): δ = 14.2, 22.7, 29.0, 29.2, 31.8, 32.5, 34.1, 35.7, 40.0, 55.4, 71.1, 73.8, 114.0, 126.7, 126.9, 127.7, 128.7, 130.8, 132.4, 138.0, 140.6, 158.2 ppm.

ESI-HRMS: *m/z* calcd for $C_{15}H_{22}O_3NNaS$ $[M+]^+$ found

Analytical Data[3] (minor product) **52B**

^{1}H-NMR (400.1 MHz, CDCl$_3$): δ = 0.89 (t, *J* = 6.5 Hz, 3H), 1.24 – 1.33 (m, 7H), 1.35 – 1.39 (m, 2H), 1.92 – 1.99 (m, 1H), 2.02 – 2.06 (m, 3H), 2.17 – 2.23 (m, 1H), 2.28 – 2.33 (m, 1H), 3.37 (dd, *J* = 5.7, 3.1 Hz, 1H), 3.83 – 3.84 (m, 3H), 4.20 – 4.26 (m, 1H), 4.62 – 4.66 (m, 1H), 5.56 (ddd, *J* = 15.5, 6.2, 1.5 Hz, 1H), 5.72 (ddd, *J* = 15.4, 6.6, 1.1 Hz, 1H), 6.93 – 6.95 (m, 2H), 7.30 – 7.35 (m, 5H), 7.36 – 7.39 (m, 2H) ppm.

^{13}C-NMR (100.6 MHz, CDCl$_3$): δ = 14.2, 22.7, 29.0, 29.1, 31.8, 32.5, 34.5, 35.8, 37.9, 55.4, 70.2, 74.1, 75.0, 114.0, 126.1, 127.3, 128.8, 131.1, 132.1, 135.5, 143.4, 157.7 .ppm.

ESI-HRMS: *m/z* calcd for $C_{15}H_{22}O_3NNaS$ $[M+]^+$ found

7 Stereoselective synthesis

Stereoselective synthesis of (2S,4R)-4-methyl-2-vinyltetrahydro-2H-pyran

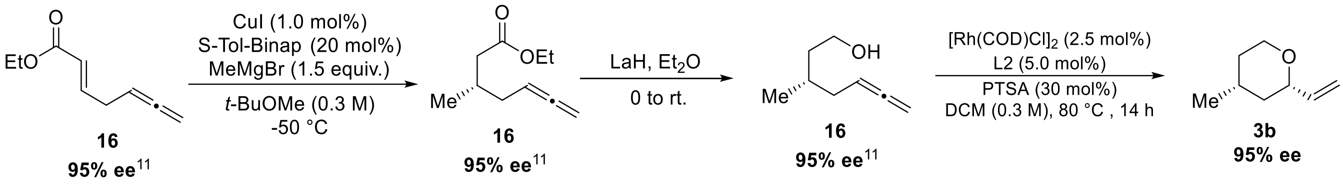

Synthesis of ethyl (S)-3-methylhepta-5,6-dienoate 130

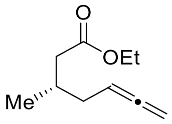

$C_{10}H_{16}O_2$
168.24

CuI (1.0 mol%) and S-Tol-Binap (20 mol%) was dissolved in *t*-BuOMe (40 mL) and stirred under argon at room Temperature for 1 h until a yellow suspension was observe. The mixture was cooled to −50 °C and ethyl (*E*)-hepta-2,5,6-trienoate (2.0 g, 13 mmol) was added and stirred for 15 min. Then MeMgBr in THF (6.5 mL, 20 mmol, 3.0 M, 1.5 equiv.) was added dropwise. The mixture was stirred at −50 °C for 2 h, then quenched by the addition of aqueous saturated NH_4Cl-solution. The layers were separated, the aqueous layer was extracted with Et_2O (4 × 30 mL), the combined organic layers were washed with brine (40 mL) and dried over Na_2SO_4. The solvent was removed and the residue was purified by flash chromatography on silica gel (Pentane:Et_2O = 60:1). The Product was obtained as a colorless oil (1.0 g, 6.2 mmol, 48 %).

Analytical Data

^{1}H-NMR (400.1 MHz, $CDCl_3$): δ = 0.98 (d, *J* = 6.4, 3H), 1.25 (t, *J* = 7.1, 3H), 1.95 – 2.16 (m, 4H), 2.30 – 2.41 (m, 1H), 4.09 – 4.17 (m, 2H), 4.65 (dt, *J* = 6.7, 2.7, 2.7, 2H), 5.00 – 5.11 (m, 1H) ppm.

^{13}C-NMR (100.6 MHz, $CDCl_3$): δ = δ = 14.4, 19.6, 30.7, 35.7, 41.2, 60.2, 74.4, 87.7, 173.1, 209.4 ppm.

APCI-HRMS: *m/z* calcd for $C_{10}H_{20}O_2N$ $[M+NH_4]^+$ 186.1489 found 186.1489

GC: Hydrodex-B-TBDAc 25m x 0.25mm, 80 °C, isothermal [94% ee. t_R = 28.9 min (minor), 29.7 min (major)], $[\alpha]_D^{25}$= −12 (c = 0.59 $CHCl_3$).

Synthesis of (S)-3-methylhepta-5,6-dien-1-ol 53

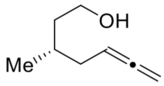

$C_8H_{14}O$
126.20

A suspension of LAH () in dry Et_2O was cooled to 0 °C then a solution of 3-ethylhepta-5,6-dienoate in Et_2O (2 mL) was added dropwise. The reaction was stirred at this temperature for 1 h and then warmed to room temperature and stirred for another hour. The mixture was quenched through the addition of H_2O and aqueous HCl (2.0 M). The layers were separated, the organic layer was washed with H_20 (20 mL) and brine (20 mL). The aqueous layer was extracted with Et_2O (4 × 20 mL). The combined organic layer were dried over Na_2SO_4, the solvent was removed. The crude product was used without further purification.

Analytical Data

^{1}H-NMR (400.1 MHz, $CDCl_3$): δ = 0.95 (d, *J* = 6.7 Hz, 3H), 1.27 (s, 1H), 1.38 – 1.48 (m, 1H), 1.61 – 1.77 (m, 2H), 1.86 – 1.98 (m, 1H), 2.04 (dddd, *J* = 14.1, 7.3, 5.8, 3.0 Hz, 1H), 3.64 – 3.77 (m, 2H), 4.64 (dd, *J* = 6.7, 2.9 Hz, 2H), 5.06 (ddt, *J*=7.4, 6.6, 2.8 Hz, 1H) ppm.

^{13}C-NMR (100.6 MHz, $CDCl_3$): δ = 19.6, 30.0, 36.1, 39.4, 61.2 74.2, 88.1, 209.3 ppm.

APCI-HRMS: *m/z* calcd for $C_8H_{18}ON$ $[M+NH_4]^+$ 144.1388 found 144.1383.

Synthesis of (2S,4R)-4-methyl-2-vinyltetrahydro-2H-pyran *5ee*

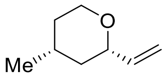

$C_8H_{14}O$
126.20

The reaction was performed according to **general procedure 8** with (S)-3-methylhepta-5,6-dien-1-ol (38 mg, 0.3 mmol, 1.0 equiv.). The reaction mixture was filtered, concentrated and the **d.r.** ratio (**d.r. = 96/4**) was determined by ^{1}H-NMR spectroscopy of the crude product. After purification by flash chromatography on silica gel (pentane/ether 80/1 to 40/1) the product was obtained as colorless liquid (63 mg, 0.28 mmol, 96%).

Analytical Data[3]

^{1}H-NMR (400.1 MHz, $CDCl_3$): δ = 0.95 (d, *J* = 6.4 Hz, 3H), 0.99 – 1.08 (m, 1H), 1.16 – 1.30 (m, 1H), 1.50 – 1.58 (m, 1H), 1.63 – 1.72 (m, 2H), 3.47 (ddd, *J* = 12.5, 11.4, 2.3 Hz, 1H), 3.73 – 3.82 (m, 1H), 4.03 (ddd, *J* = 11.4, 4.5, 1.6 Hz, 1H), 5.08 (dt, *J*=10.6, 1.5, 1H), 5.22 (ddd, *J* = 17.3, 1.6 Hz, 1H), 5.85 (ddd, *J* = 17.3, 10.6, 5.5 Hz, 1H) ppm.

^{13}C-NMR (100.6 MHz, $CDCl_3$): δ = 22.4, 30.3, 34.5, 40.6, 68.1, 78.2, 114.5, 139.5 ppm.

APCI-HRMS: *m/z* calcd for $C_8H_{15}O$ $[M+H]^+$ 127.1123 found 127.1117.

GC: Hydrodex-B-TBDAc 25m x 0.25mm, 80 °C, isothermal [94% ee. t_R = 4.34 min (major), 4.57 min (major)], $[\alpha]_D^{25}$= −12 (c = 0.59 $CHCl_3$).

Stereoselective synthesis of (2S,4R)-2-((E)-but-1-en-1-yl)-4-methyltetrahydro-2H-pyran

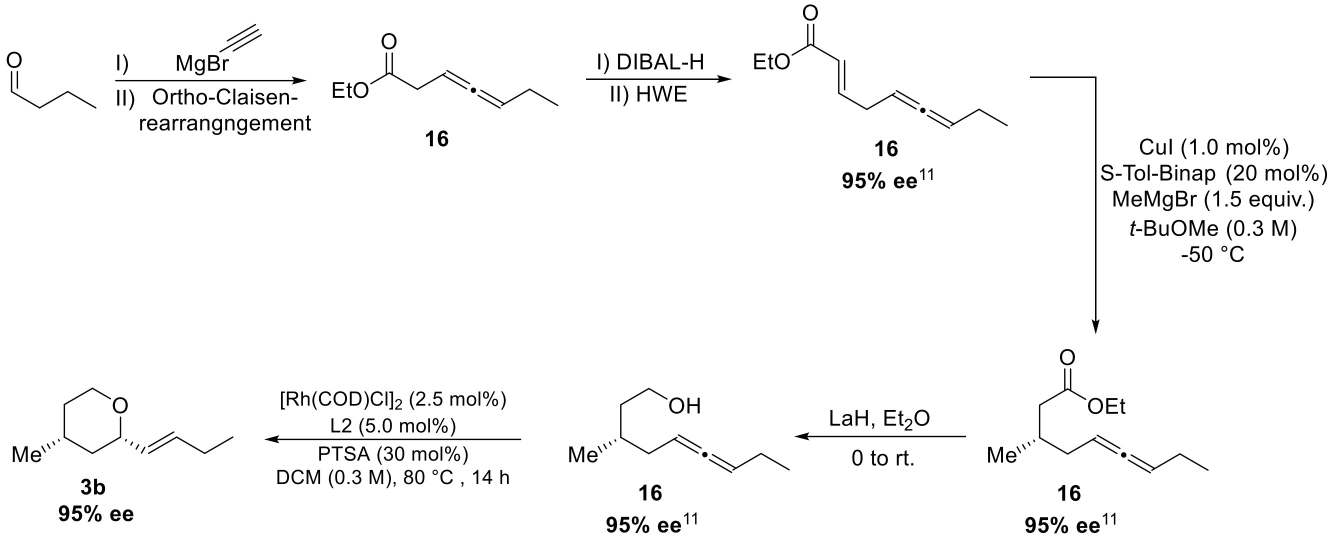

Synthesis of ethyl hepta-3,4-dienoate 131

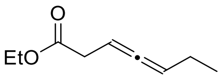

$C_9H_{14}O_2$
154.21

The reaction was performed according to **general procedure 5.**

I) Butyraldehyde (4.3 g, 5.4 mL, 60 mmol, 1.0 equiv.) and ethinyl magnesium bromide (130 mL, 65 mmol, 0.5 M in THF, 1.08 equiv.). The crude product was filtered over a short silica pad (2:1 pentane/Et_2O). hex-1-yn-3-ol was obtained as a yellow liquid (5.6 g, 57 mmol, 95%)

II) Crude hex-1-yn-3-ol (step **I**) (5.6 g, 57 mmol, 1.0 equiv.), triethyl orthoacetate (15 g, 17 mL, 90 mmol, 1.05 equiv.), propionic acid (3 × 5 mol%). The crude product was purified by fractional distillation under reduced pressure (75 - 78 °C, 10 mbar) to obtain the title product (5.6 g, 36 mmol, 63%) as a colorless liquid.

Analytical Data

^{1}H-NMR (400.1 MHz, $CDCl_3$): δ= 0.99 (t, *J* = 7.4 Hz, 3H), 1.26 (t, *J* = 7.1 Hz, 3H), 1.97 – 2.04 (m, 2H), 2.99 – 3.02 (m, 2H), 4.15 (q, *J* = 7.2 Hz, 2H), 5.21 – 5.29 (m, 2H) ppm.

^{13}C-NMR (100.6 MHz, $CDCl_3$): δ =13.3, 14.3, 21.7, 35.2, 60.7, 84.9, 94.0, 171.7 204.8 ppm.

APCI-HRMS: *m/z* calcd for $C_9H_{14}O_2$ $[M+H]^+$ 155.1067 found 155.1067.

Synthesis of ethyl (E)-nona-2,5,6-trienoate 132.

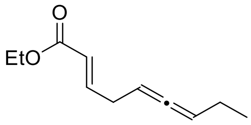

$C_{11}H_{16}O_2$
180.25

The reaction was performed according to **general procedure 6**.

I) Ethyl hepta-3,4-dienoate (5.6 g, 36 mmol, 1.0 equiv.) and DIBAL-H (52 mL, 52 mmol, 1.45 equiv., 1.0 M in DCM). The crude product was filtered over a short silica pad (DCM). Hepta-3,4-dienal was obtained as a yellow liquid (3.7g, 33 mmol, 92%).

II) Crude hepta-3,4-dienal (step **I**) (3.7 g, 33 mmol), NaH (60% in mineral oil, 1.4 g, 61 mmol, 1.1 equiv.) in dry THF (100 mL) and triethyl phosphonoacetate (9.6 g, 8.5 mL, 43 mmol,1.3 equiv.). The crude product was purified by flash chromatography on silica gel (40:1 Pentane:Et_2O). The title compound was obtained as colorless liquid (4.5 g, 25 mmol, 75%).

Analytical Data10

^{1}H-NMR (400.1 MHz, $CDCl_3$): δ = 1.0 (t, *J* = 7.4 Hz, 3H), 1.3 (t, *J* = 7.1 Hr, 3H), 2.0 (qdd, *J* = 7.4, 6.2, 3.2 Hz, 2H), 2.9 (tdd, *J* = 6.6, 2.8, 1.7 Hz, 2H), 4.2 (q, *J* = 7.1 Hz, 2H), 5.1 – 5.2 (m, 1H), 5.2 (qt, *J* = 6.3, 2.8 Hz, 1H), 5.9 (dt, *J* = 15.6, 1.7 Hz, 1H), 7.0 (dtd, *J* = 15.6, 6.5, 0.4 Hz, 1H) ppm.

^{13}C-NMR (100.6 MHz, $CDCl_3$): δ = 13.4, 14.4, 21.9, 32.1, 60.3, 87.8, 94.0, 122.0, 146.8, 166.6, 204.5 ppm.

APCI-HRMS: *m/z* calcd for $C_{11}H_{17}O_2$ $[M+H]^+$ 181.1225 found 181.1225.

Synthesis of ethyl (3S)-3-methylnona-5,6-dienoate 133.

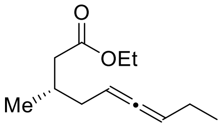

$C_{12}H_{20}O_2$
196.29

CuI (1.0 mol%) and S-Tol-Binap (20 mol%) was dissolved in *t*-BuOMe (40 mL) and stirred under argon at room Temperature for 1 h until a yellow suspension was observe. The mixture was cooled to −50 °C and ethyl (E)-nona-2,5,6-trienoate (1.8 g, 10 mmol) was added and stirred for 15 min. Then MeMgBr in THF (5.0 mL, 25 mmol, 3.0 M, 1.5 equiv.) was added dropwise. The mixture was stirred at −50 °C for 2 h, then quenched by the addition of aqueous saturated NH_4Cl-solution. The layers were separated, the aqueous layer was extracted with Et_2O (4 × 30 mL), the combined organic layers were washed with brine (40 mL) and dried over Na_2SO_4. The solvent was removed and the residue was purified by flash chromatography on silica gel (Pentane:Et_2O = 60:1). The Product was obtained as a colorless oil (0.88 g, 4.5 mmol, 45 %).

Analytical Data[3]

^{1}H-NMR (400.1 MHz, $CDCl_3$): δ = 0.93 – 0.98 (m, 2H), 1.00 (t, *J* = 7.4 Hz, 3H), 1.25 (dd, *J* = 7.1, 6.9 Hz 3H), 1.90 – 2.14 (m, 7H), 2.35 – 2.42 (m, 1H), 4.13 (qd, *J* = 7.2, 1.2 Hz, 2H), 5.04 (ddddd, *J* = 9.3, 6.2, 4.0, 3.2, 2.1 Hz, 1H), 5.10 – 5.16 (m, 1H).ppm.

^{13}C-NMR (100.6 MHz, $CDCl_3$): δ =7.9, 13.6, 14.4, 19.6, 19.7, 22.1, 27.7, 30.7, 30.8, 36.5, 36.6, 41.2, 41.2, 60.2, 89.1, 89.2, 92.5, 102.5, 173.2, 204.4 ppm.

APCI-HRMS: *m/z* calcd for $C_{12}H_{21}O_2$ $[M+H]^+$ 197.1541 found 197.1537.

GC: Hydrodex-B-TBDAc 25m x 0.25mm, 75 °C, isothermal [97% ee. t_R = 132.02 min (minor), 136.50 min (major)].

Synthesis of (S)-3-methylhepta-5,6-dien-1-ol 54.

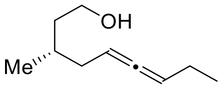

$C_{10}H_{18}O$
154.25

A suspension of LAH () in dry Et_2O was cooled to 0 °C then a solution of 3-ethylhepta-5,6-dienoate in Et_2O (2 mL) was added dropwise. The reaction was stirred at this temperature for 1 h and then warmed to room temperature and stirred for another hour. The mixture was quenched through the addition of H_2O and aqueous HCl (2.0 M). The layers were separated, the organic layer was washed with H20 (20 mL) and brine (20 mL). The aqueous layer was extracted with Et_2O (4 × 20 mL). The combined organic layer were dried over Na_2SO_4, the solvent was removed. The crude product was used without further purification.

Analytical Data[3]

^{1}H-NMR (400.1 MHz, $CDCl_3$): δ = 0.94 – 0.97 (m, 3H), 1.01 (t, *J* = 7.4 Hz, 3H), 1.33 (s, 1H), 1.39 – 1.48 (m, 1H), 1.63 – 1.75 (m, 2H), 1.83 – 1.95 (m, 1H), 1.96 – 2.08 (m, 3H), 3.64 – 3.76 (m, 2H), 5.01 – 5.15 (m, 2H) ppm.

^{13}C-NMR (100.6 MHz, $CDCl_3$): δ = 13.6, 13.6, 19.6, 22.1, 22.1, 30.0, 36.8, 36.9, 39.4, 39.5, 61.3, 89.5, 89.6, 92.2, 92.3, 204.3 ppm.

APCI-HRMS: *m/z* calcd for $C_{10}H_{19}O$ $[M+H]^+$ 155.1430 found 155.1430.

Synthesis of (2S,4R)-2-((E)-but-1-en-1-yl)-4-methyltetrahydro-2H-pyran 55*ee*.

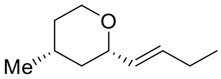

$C_{10}H_{18}O$
154.25

The reaction was performed according to **general procedure 9** with (S)-3-methylhepta-5,6-dien-1-ol (46 mg, 0.3 mmol, 1.0 equiv.). The reaction mixture was filtered, concentrated and the **d.r.** ratio (**d.r. = 75/25**) was determined by ^{1}H-NMR spectroscopy of the crude product. After purification by flash chromatography on silica gel (pentane/ether 80/1 to 40/1) the product was obtained as colorless liquid (63 mg, 0.28 mmol, 96%).

Analytical Data[6]

^{1}H-NMR (400.1 MHz, $CDCl_3$): δ = 0.94 (d, *J* = 6.4 Hz, 3H), 0.97 – 1.01 (m, 3H), 1.17 – 1.28 (m, 2H), 1.49 – 1.55 (m, 1H), 1.58 – 1.68 (m, 2H), 1.99 – 2.16 (m, 2H), 3.40 – 3.54 (m, 1H), 3.61 – 3.84 (m, 1H), 4.00 (dddd, *J* = 11.1, 6.5, 4.5, 1.6 Hz, 1H), 5.40 – 5.50 (m, 1H), 5.72 (ddd, *J*= 15.5, 6.3, 6.2, 1.1 Hz, 1H).ppm.

^{13}C-NMR (100.6 MHz, $CDCl_3$): δ = 13.4, 14.4, 21.3, 22.4, 25.3, 30.3, 30.3, 34.5, 34.6 , 40.9, 41.0, 67.9, 68.1 , 130.3, 130.5, 133.5 ppm.

ESI-HRMS: *m/z* calcd for $C_{10}H_{18}O$ [M+H]$^+$ 155. 1430 found 155.1436.

GC: CYCLOSIL-B 30m x 0.25mm, 70°C, isothermal [94% *ee* t_R = 25.9 min (minor), 26.3 min (major)].

8 Catalysis followed by in situ hydration

General Procedure 10

A 10 mL screw-cap flask was flame-dried, cooled to room temperature under vacuum and backfilled with argon (Argon 5.0 Sauerstoffwerk Friedrichshafen) using a standard SCHLENK line apparatus. The screw-cap flask was charged with the corresponding internal δ-hydroxy allene (0.3 mmol, 1.0 equiv.), evacuated for 15 and then backfilled with argon three times. Then $[Rh(COD)Cl]_2$ (3.7 mg, 0.0075 mmol, 2.5 mol%), dppf (8.3 mg, 0.015 mmol, 5.0 mol%) and PTSA (17.1 mg, 0.09 mmol, 30 mol%) was added under a flow of argon followed by freshly distilled PhF (0.3 M). The flask was sealed and stirred at 80 °C overnight. The reaction mixture was diluted with MeOH (0.3 M) and Pd/C (5%, 30 mol%) was added. The mixture was hydrogenated at roomtemperature for 6 hours. The suspension was filteredand the solvent was evaporated to obtain the crude product. The residue was analyzed by ^{1}H-NMR spectroscopy. The crude product was purified by flash chromatography on silica gel using a mixture of pentane/ether.

Synthesis of *syn*-(4-cyclopropyl-2-octyltetrahydro-2H-pyran 56

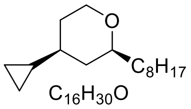

$C_{16}H_{30}O$
238.42

The reaction was performed according to **general procedure 10** with 3-cyclopropyltrideca-5,6-dien-1-ol (71 mg, 0.3 mmol, 1.0 equiv.). The reaction mixture was filtered, concentrated and the **d.r.** ratio (**d.r. = 93/7**) was determined by ^{1}H-NMR spectroscopy of the crude product. After purification by flash chromatography on silica gel (pentane/ether 80/1 to 40/1) the product was obtained as colorless liquid (67 mg, 0.28 mmol, 94%).

Analytical Data

^{1}H-NMR (400.1 MHz, $CDCl_3$): δ = 0.05 – 0.09 (m, 2H), 0.36 – 0.42 (m, 2H), 0.47 – 0.57 (m, 1H), 0.69 – 0.79 (m, 1H), 0.88 (s, 3H), 1.05 (ddd, *J* = 13.1, 11.9, 11.0 Hz, 1H), 1.25 – 1.30 (m, 11H), 1.35 – 1.43 (m, 3H), 1.47 – 1.54 (m, 1H), 1.59 – 1.64 (m, 1H), 1.71 (dddd, *J* = 13.0, 3.9, 2.0 Hz, 1H), 3.14 (dddd, *J* = 11.0, 7.2, 4.9, 2.1 Hz, 1H), 3.32 (ddd, *J* = 12.5, 11.4, 2.3 Hz, 1H), 3.99 (ddd, *J* = 11.4, 4.6, 1.6 Hz, 1H) ppm.

^{13}C-NMR (100.6 MHz, $CDCl_3$): δ = 2.8, 14.2, 17.3, 22.8, 25.7, 29.4, 29.7, 29.9, 32.0, 32.7, 36.7, 38.5, 40.7, 68.2, 77.8 ppm.

APCI-HRMS: *m/z* calcd for $C_{16}H_{31}O$ [M+H]$^+$ 239.2375 found 239.2369.

Synthesis of *syn* 2-octyl-4-(4-phenylbutyl)tetrahydro-2H-pyran 57

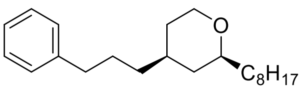

$C_{22}H_{36}O$
316.53

The reaction was performed according to **general procedure 10** with 3-(3-phenylpropyl)trideca-5,6-dien-1-ol (98 mg, 0.3 mmol, 1.0 equiv.). The reaction mixture was filtered, concentrated and the **d.r.** ratio (**d.r. = 92/8**) was determined by ^{1}H-NMR spectroscopy of the crude product. After purification by flash chromatography on silica gel (pentane/ether 80/1 to 40/1) the product was obtained as colorless liquid (96 mg, 0.29 mmol, 98%).

Analytical Data

^{1}H-NMR (400.1 MHz, $CDCl_3$): δ = 0.87 (t, *J* = 7.0 Hz, 3H), 1.13 – 1.21 (m, 1H), 1.25 – 1.29 (m, 14iii), 1.34 – 1.42 (m, 2H), 1.44 – 1.53 (m, 2H), 1.56 (ddd, *J* = 12.8, 4.1, 2.0 Hz, 1H), 1.60 – 1.69 (m, 3H), 2.58 – 2.63 (m, 2H), 3.20 (dddd, *J* = 11.0, 7.1, 4.8, 2.0 Hz, 1H), 3.38 (ddd, *J* = 12.5, 11.4, 2.2 Hz, 1H), 3.99 (ddd, *J* = 11.4, 4.6, 1.6 Hz, 1H), 7.16 – 7.20 (m, 3H), 7.26 – 7.31 (m, 2H) ppm.

^{13}C-NMR (100.6 MHz, $CDCl_3$): δ = 14.2, 22.8, 25.7, 28.4, 29.4, 29.7, 29.9, 32.0, 33.1, 35.4, 36.2, 36.8, 36.9, 38.9, 68.2, 77.7, 125.7, 128.3, 128.4, 142.7 ppm.

APCI-HRMS: *m/z* calcd for $C_{22}H_{37}O$ $[M+H]^+$ 317.2838 found 317.2838.

Synthesis of *syn*-4-([1,1'-biphenyl]-4-yl)-2-octyltetrahydro-2H-pyran 58

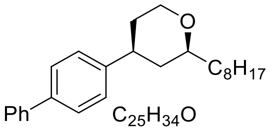

$C_{25}H_{34}O$
350.55

The reaction was performed according to **general procedure 10** with 3-([1,1'-biphenyl]-4-yl)trideca-5,6-dien-1-ol (105 mg, 0.3 mmol, 1.0 equiv.). The reaction mixture was filtered, concentrated and the **d.r.** ratio (**d.r. = 94/6**) was determined by ^{1}H-NMR spectroscopy of the crude product. After purification by flash chromatography on silica gel (pentane/ether 80/1 to 40/1) the product was obtained as colorless liquid (97 mg, 0.28 mmol, 92%).

Analytical Data

^{1}H-NMR (400.1 MHz, $CDCl_3$): δ = 0.87 – 0.91 (m, 3H), 1.25 – 1.35 (m, 11H), 1.42 – 1.52 (m, 3H), 1.53 – 1.64 (m, 1H), 1.77 – 1.84 (m, 2H), 1.85 – 1.91 (m, 1H), 2.77 – 2.87 (m, 1H), 3.41 (dddd, *J* = 12.0, 7.0, 4.8, 2.0 Hz, 1H), 3.55 – 3.64 (m, 1H), 4.12 – 4.17 (m, 1H), 7.29 – 7.32 (m, 2H), 7.33 – 7.36 (m, 1H), 7.40 – 7.46 (m, 2H), 7.54 – 7.60 (m, 4H) ppm.

^{13}C-NMR (100.6 MHz, $CDCl_3$): δ = 14.2, 22.8, 25.6, 29.4, 29.7, 29.9, 32.0, 33.8, 36.7, 39.7, 41.7, 68.3, 78.0, 127.1, 127.2, 127.3, 127.3, 128.8, 139.4, 141.1, 145.2 ppm.

APCI-HRMS: *m/z* calcd for $C_{25}H_{35}O$ $[M+H]^+$ 351.2681 found 351.2682.

Synthesis of *syn*-4-(4-methoxyphenyl)-2-(4-phenylbutyl)tetrahydro-2H-pyran 59

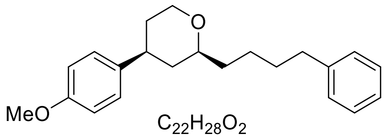

$C_{22}H_{28}O_2$
324.46

The reaction was performed according to **general procedure 10** with 3-(4-methoxyphenyl)-9-phenylnona-5,6-dien-1-ol (97 mg, 0.3 mmol, 1.0 equiv.). The reaction mixture was filtered, concentrated and the **d.r.** ratio (**d.r. = 93/7**) was determined by ^{1}H-NMR spectroscopy of the crude product. After purification by flash chromatography on silica gel (pentane/ether 80/1 to 40/1) the product was obtained as colorless liquid (87 mg, 0.28 mmol, 93%).

Analytical Data

^{1}H-NMR (500.1 MHz, CDCl$_3$): δ = 1.34 – 1.53 (m, 4H), 1.64 (dddd, *J* = 11.9, 7.3, 3.6, 1.7 Hz, 2H), 1.72 (ddd, *J* = 10.8, 8.5, 3.7 Hz, 2H), 1.79 (ddd, *J* = 13.1, 3.8, 1.5 Hz, 1H), 2.62 (dd, *J* = 7.8, 6.9 Hz, 2H), 2.66 – 2.76 (m, 1H), 3.36 (dd, *J* = 4.7, 2.4 Hz, 1H), 3.51 – 3.58 (m, 1H), 3.80 (s, 3H), 4.08 – 4.14 (m, 1H), 6.84 – 6.88 (m, 2H), 7.12 – 7.16 (m, 2H), 7.16 – 7.19 (m, 3H), 7.26 – 7.29 (m, 2H) ppm.

^{13}C-NMR (125.6 MHz, CDCl$_3$): δ = 25.4, 31.7, 34.0, 36.0, 36.5, 39.9, 41.0, 55.4, 68.3, 77.8, 114.0, 125.7, 127.7, 128.3, 128.5, 138.3, 142.8, 158.1 ppm.

APCI-HRMS: *m/z* calcd for $C_{22}H_{29}O_2$ $[M+H]^+$ 325.2168 found 325.2169.

9 Total synthesis of (-)-centrolobine

Synthesis of 5-(4-methoxyphenyl)-5-oxopentanal 61

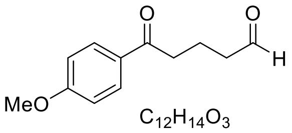

$C_{12}H_{14}O_3$
206.24

A solution of 1-(cyclopent-1-en-1-yl)-4-methoxybenzene[1] (5.0 g, 29 mmol) in MeOH (200 mL) was cooled to −78 °C. Ozone was bubbled through the solution until the solution showed a blue colour (approx. 30 min) then, the reaction vessel was degassed with nitrogen until disappearance of the blue colour occurred. Me_2S (15 mL, 10 equiv.) was added and the reaction mixture was allowed to warm to room temperature over night. The solution was concentrated under recued pressure crude product was purified by flash chromatography on silica gel (pentane/ether 10/1 to 4/1). The desired product as obtained as white solid (5.0 g, 24 mmol, 83%).

Analytical Data

^{1}H-NMR (400.1 MHz, $CDCl_3$): δ = 2.07 (tt, *J* = 7.0, 7.1 Hz, 2H), 2.58 (td, *J* = 7.1, 1.4 Hz, 2H), 2.99 (t, *J* = 7.1 Hz, 2H), 3.87 (s, 3H), 6.90 – 6.97 (m, 2H), 7.91 – 7.98 (m, 2H), 9.80 (s, 1H) ppm.

^{13}C-NMR (100.6 MHz, $CDCl_3$): δ = 16.8, 37.0, 43.3, 55.6, 113.8, 130.4, 130.4, 163.6, 198.0, 202.1 ppm.

ESI-HRMS: *m/z* calcd for $C_{12}H_{15}O_3$ $[M+H]^+$ 207.1016 found 207.1013.

[1] L. En-Chih, J. Topczewski, *J. Am. Chem. Soc.* **2019**, *14*, 5135-5138.

Synthesis of 7-(4-methoxyphenyl)-7-oxohept-1-yn-3-yl acetate

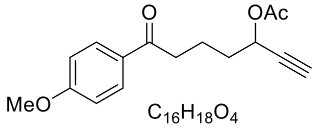

$C_{16}H_{18}O_4$
274.31

Ethynylmagnesium bromide (0,5 M in THF, 15 mL, 7.8 mmo, 1.0 equiv,) was cooled to 0 °C. 5-(4-methoxyphenyl)-5-oxopentanal (1.6 g, 7.8 mmol) was added dropwise and the solution was stirred for 1 h at rt. Then cooled to 0 °C and acetyl chloride (0.6 mL, 8.5 mmol) was added dropwise. The solution was warmed to rt. And stirred for 1 h. The reaction was quenched with H_2O (10 mL) and the aqueous layer was extracted with Et_2O (3 × 25 mL). The combined organic layer was dried over Na2SO4, the solvent was removed under reduced pressure and the resulting crude product was purified by flash chromatography on silica gel (pentane/ether 10/1 to 4/1). The desired product as obtained as colorless oil (1.9 g, 19 mmol, 85%)

Analytical Data

^{1}H-NMR (400.1 MHz, $CDCl_3$): δ = 1.84 – 1.95 (m, 4H), 2.07 (s, 3H), 2.46 (d, *J* = 2.2 Hz ppm, 1H), 2.90 – 3.01 (m, 2H), 3.86 (s, 3H), 5.36 – 5.42 (m, 1H), 6.90 – 6.97 (m, 2H), 7.90 – 7.97 (m, 2H) ppm.

^{13}C-NMR (100.6 MHz, $CDCl_3$): δ = 19.8, 21.0, 34.1, 37.4, 55.5, 63.5, 73.8, 81.1, 113.8, 130.1, 130.3, 163.5, 169.9, 198.0 ppm.

ESI-HRMS: *m/z* calcd for $C_{16}H_{19}O_4$ $[M+H]^+$ 275.1278 found 275.1280.

Synthesis of 7-(4-(benzyloxy)phenyl)-1-(4-methoxyphenyl)hepta-5,6-dien-1-one 62

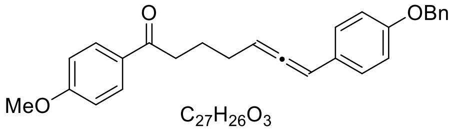

$C_{27}H_{26}O_3$
398.50

To a solution of 7-(4-(benzyloxy)phenyl)-1-(4-methoxyphenyl)hepta-5,6-dien-1-one (1.0 M in THF, 4.1 mL, 4.1 mmol, 1.1 equiv.), CuBr (61mg, 0.4 mmol, 10 mol%) in THF (10 mL) was cooled to 0 °C. At this temperature (4-(benzyloxy)phenyl)magnesium bromide (1.1 g, 4.0 mmol, 1.0 equiv.) was added dropwise. The reaction was allowed to warm to rt. and stirred for 2 h. Saturated ammonium chloride (10 mL) was added and the aqueous layer was separated and extracted with Et_2O (2 × 20 mL). The combined organic layers were dried over Na_2SO_4, the solvent was removed under reduced pressure and the two Diastereomers were separated by flash Chromatography (SiO2, pentane/Et2O = 10/1 to 2/1). The desired product as obtained as colorless oil (0.78 g, 2.0 mmol, 48%)

Analytical Data

^{1}H-NMR (400.1 MHz, $CDCl_3$): δ = 1.89 – 1.98 (m, 2H), 2.19 – 2.28 (m, 2H), 2.95 – 3.03 (m, 2H), 3.85 (s, 3H), 5.05 (s, 2H), 5.55 – 5.78 (m, 1H), 6.11 (dt, *J* = 6.2, 3.0 Hz, 1H), 6.89 – 6.93 (m, 4H), 7.21 (dt, *J* = 8.9, 0.5 Hz, 2H), 7.31 – 7.47 (m, 6H), 7.89 – 7.92 (m, 2H) ppm.

^{13}C-NMR (100.6 MHz, $CDCl_3$): δ = 23.8, 28.6, 37.6, 55.5, 70.2, 94.4, 94.5, 113.8, 115.2, 127.5, 127.6, 127.8, 128.0, 128.6, 130.3, 130.4, 137.1, 158.0, 163.4, 198.7, 204.9 ppm.

ESI-HRMS: *m/z* calcd for $C_{12}H_{15}O_3$ $[M+H]^+$ 207.1016 found 207.1013.

Synthesis of (1S)-7-(4-(benzyloxy)phenyl)-1-(4-methoxyphenyl)hepta-5,6-dien-1-ol 63

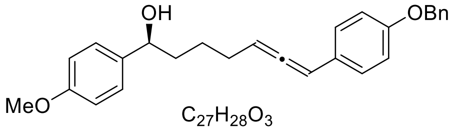

$C_{27}H_{28}O_3$
400.52

7-(4-(benzyloxy)phenyl)-1-(4-methoxyphenyl)hepta-5,6-dien-1-one (0.42 g, 1.1 mmol, 1.0 equiv.) was solved in THF (7 mL) and (+)-CBS-Cat. (1 M in toluene, 1.0 mL, 2.0 mmol, 1.0 equiv.) was added before it was cooled to −20 °C. Then BH_3·THF (1 M, 2.2 mL, 2.2 mml, 2.0 equiv.) was added dropwise at −20 °C and the solution was stirred for 5 h. Afterwards, MeOH (2 mL) was added and the reaction was warmed to room temperature. The mixture was quenched by adding aq. HCl (1 M, 10 mL) and the layers were separated. The aqueous layer was extracted with Et_2O (3 × 50 mL) and the combined organic layers were dried over Na_2SO_4. The solvent was removed under reduced pressure and the crude product was purified by column chromatography (pentane: Et_2O = 2:1) to afford the desired product (374 mg, 0.94 mmol, 85 %) as a yellow liquid.

Analytical Data

^{1}H-NMR (400.1 MHz, $CDCl_3$): δ = 1.44 – 1.92 (m, 6H), 2.15 (dddd, *J* = 7.2, 7.2, 7.1, 3.0 Hz, 2H), 3.80 (d, *J* = 0.9 Hz, 3H), 4.62 (ddd, *J* = 7.9, 5.7 Hz, 2.8, 1H), 5.06 (s, 2H), 5.45 – 5.56 (m, 1H), 6.08 (ddd, *J*=6.2, 3.0, 2.9, 1.0 Hz, 1H), 6.82 – 6.94 (m, 4H), 7.17 – 7.26 (m, 4H), 7.29 – 7.48 (m, 5H) ppm.

^{13}C-NMR (100.6 MHz, $CDCl_3$): δ = 25.5, 25.5, 28.8, 38.5, 55.4, 70.2, 74.1, 74.2, 94.3, 94.7, 113.9, 113.9, 115.2, 127.2, 127.5, 127.7, 127.8, 128.0, 128.6, 137.0, 157.9, 204.7, 205.7 ppm.

^{1}H-NMR (400.13 MHz, $CDCl_3$):

HR-MS ($C_{27}H_{28}O_3$; $[M+Na]^+$, pos. ESI): calculated: 423.1931, found: 423.1935.

Synthesis of (-)-Centrolobine

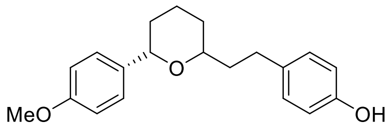

$C_{20}H_{24}O_3$
312.41

I) A screw-cap flask was flame-dried, cooled to room temperature under vacuum and backfilled with argon using a standard SCHLENK line apparatus. The screw-cap flask was charged with (1*S*)-7-(4-(benzyloxy)phenyl)-1-(4-methoxyphenyl)hepta-5,6-dien-1-ol 63 (120 mg, 0.3 mmol, 1.0 equiv.) the flask was evacuated for 15 min and backfilled with argon three times. Then $[Rh(COD)Cl]_2$ (3.7 mg, 0.075 mmol, 2.5 mol%), dppf (8.3 mg, 0.015 mmol, 5.0 mol%) and HOAcCl (19 mg, 0.09 mmol, 30 mol%) was added under a flow of argon followed by freshly distilled DCE. The flask was sealed and stirred at 80 °C overnight. The reaction mixture concentrated and the **d.r.** ratio (**d.r. = 90/10**) was determined by ^{1}H-NMR spectroscopy of the crude product. The product was obtained as yellow oil (99 mg, 0.25 mmol, 83%).

II) (2*R*,6*S*)-2-((E)-4-(benzyloxy)styryl)-6-(4-methoxyphenyl)tetrahydro-2H-pyran (99.0 mg, 0.25 mmol, 1.0 equiv.) and Pd (5 wt% on carbon, 5.0 mol%) were stirred in MeOH (1.1 mL) under a H_2-atomosphere (1 atm) at room temperature for 72 h. The reaction mixture was then filtrated over a pad of celite, rinsed with Et_2O (5 mL) and concentrated under reduced pressure. The residue was purified by column chromatography (SiO_2, pentane:Et_2O = 5:1 → 1:1) to afford the desired product (64 mg, 0.21 mmol, 80%).

Analytical data:

^{1}H-NMR (400.13 MHz; $CDCl_3$): δ = 0.83 – 0.90 (m, 2H), 1.00 – 1.22 (m, 3H), 1.47 (m, 3H), 1.77 (m, 1H), 2.23 – 2.33 (m, 1H), 2.44 – 2.56 (m, 1H), 3.22 (s, 1H), 3.77 (s, 3H), 4.31 (m, 1H), 6.76 – 6.86 (m, 4H), 7.09 – 7.22 (m, 4H) ppm.

^{13}C-NMR (100.6 MHz, $CDCl_3$): δ = 24.2, 30.9, 31.4, 33.5, 38.4, 55.4, 113.7, 115.2, 127.1, 129.6, 134.9, 136.0, 153.6,, 158.8.

HR-MS ($C_{20}H_{24}O_3$; $[M+Na]^+$, pos. ESI): calculated: 335.1618, found: 335.1617.

10 Determination of relative configuration

Exemplary determination of the relative configuration of 4-phenyl-2-vinyltetrahydro-2H-pyran

NOE-Experiment ***syn*-Product:** (major compound)

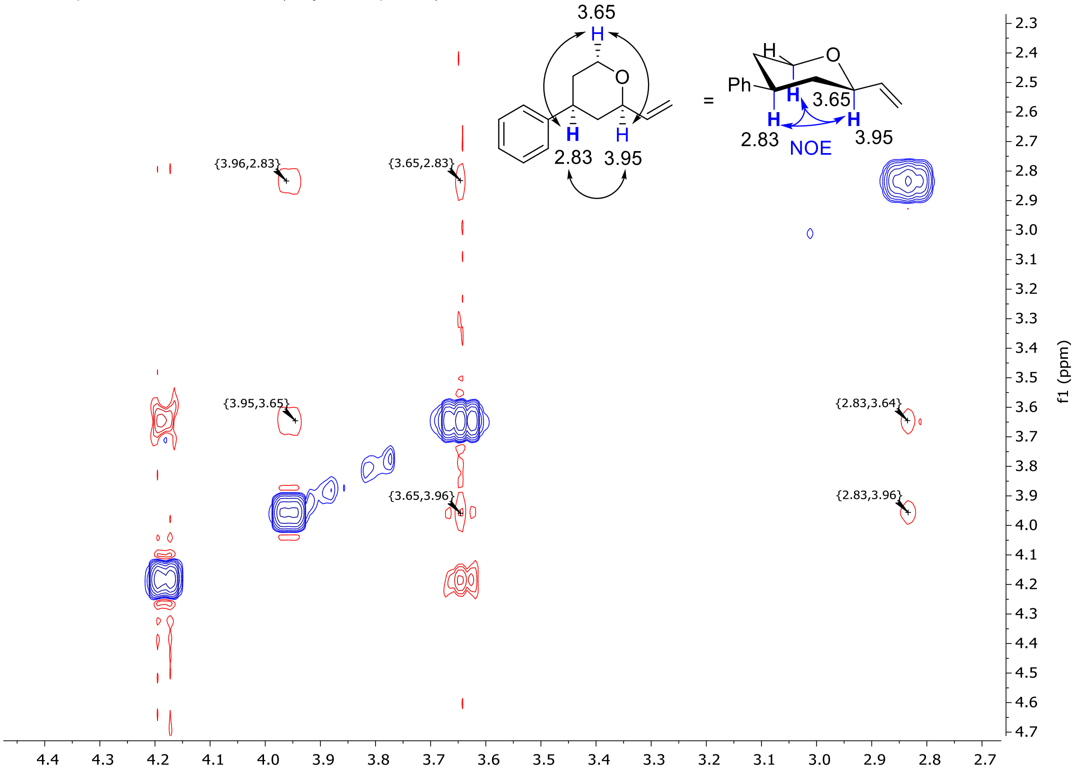

NOE-Experiment *anti*-Product: (minor-compound)

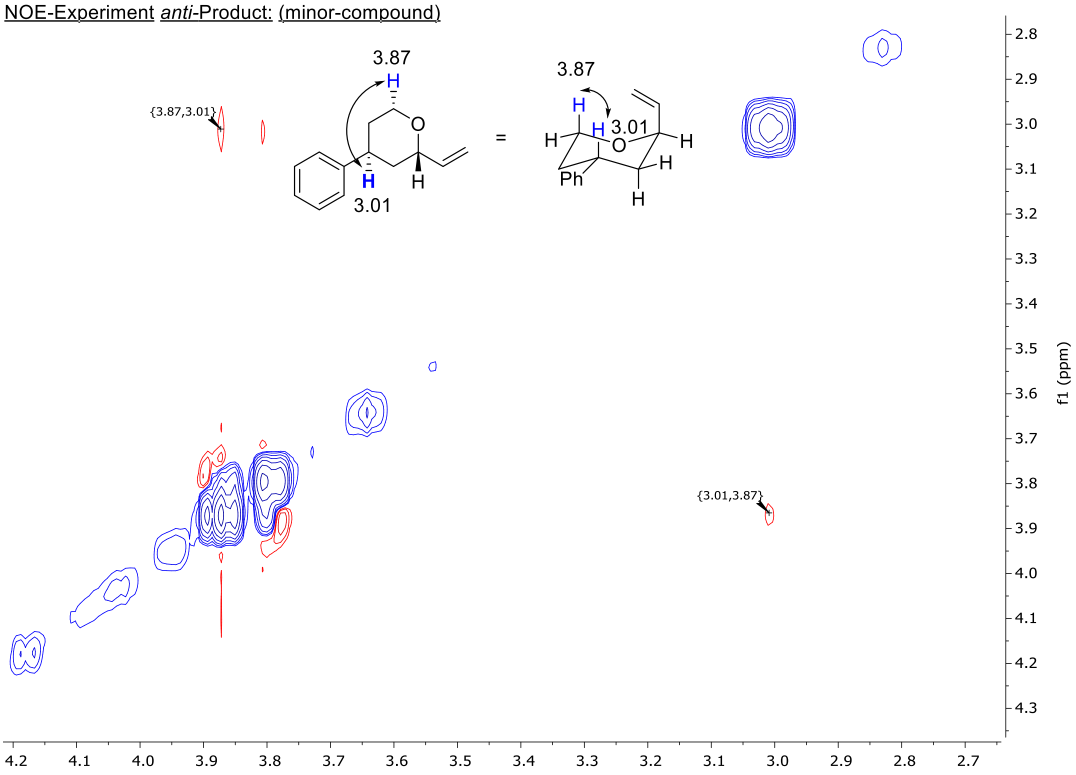

Exemplary determination of the relative configuration of 2,4-diphenyl-6-vinyltetrahydro-2H-pyran

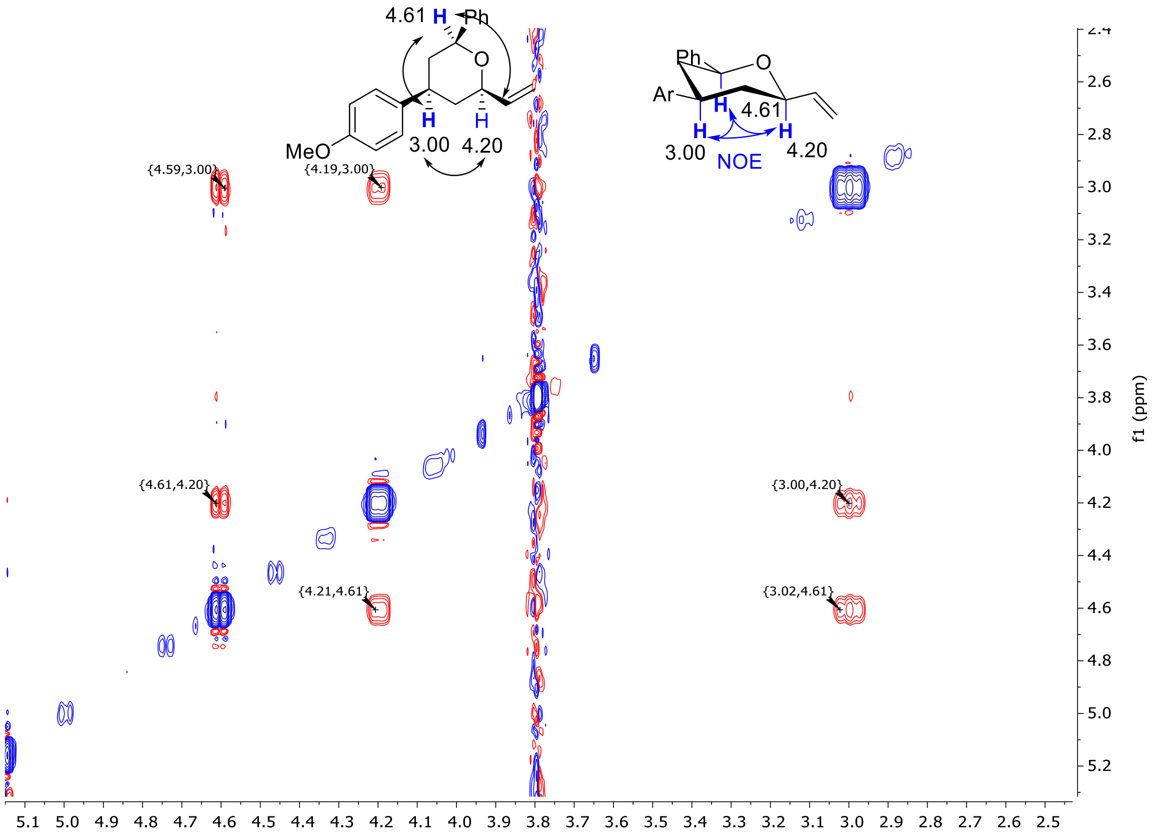

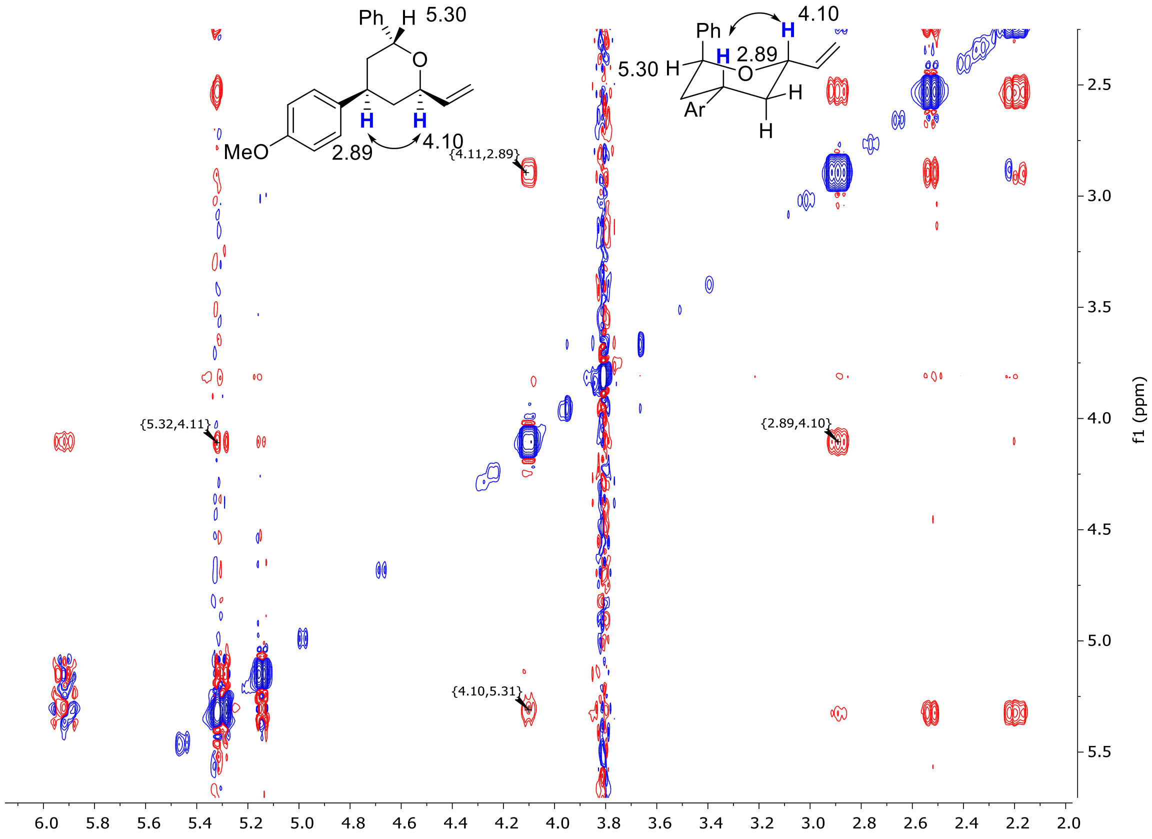

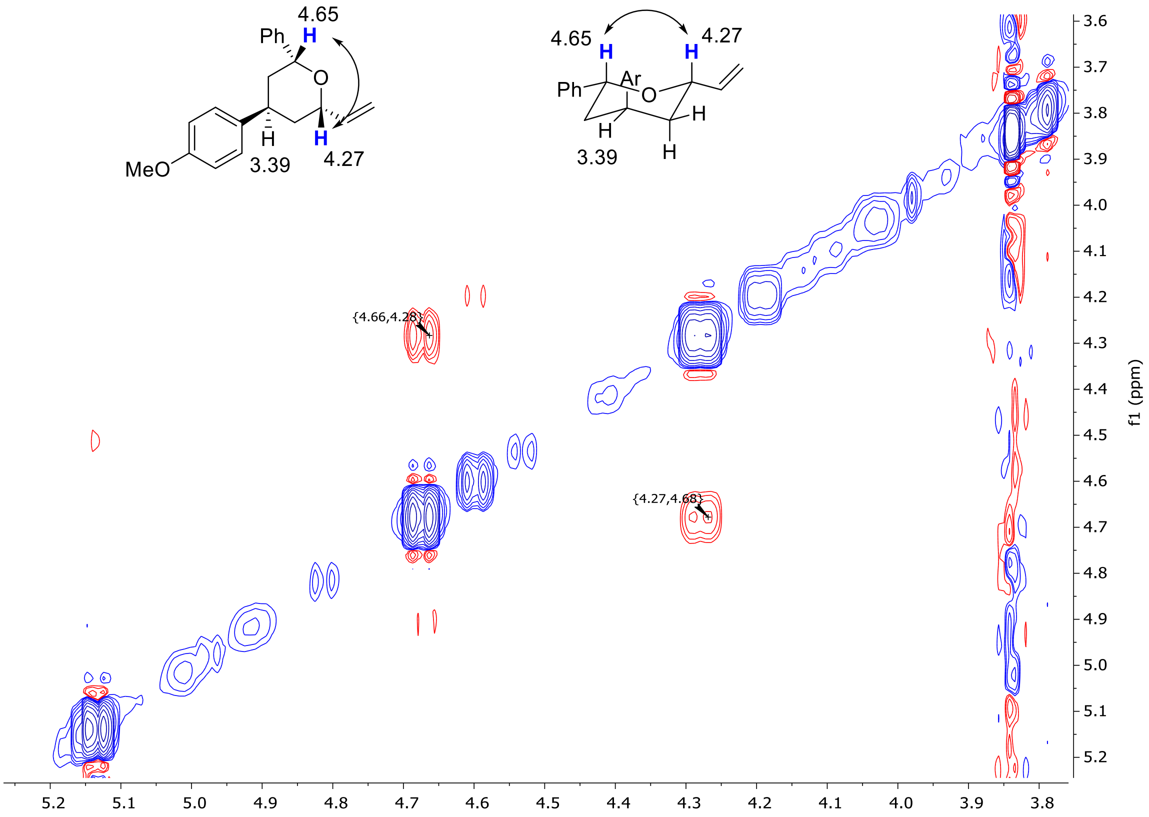
4.65
Ph
H
O
MeO
H
3.39
H
4.27
4.65
H
4.27
H
Ph
Ar
O
H
H
3.39
H
{4.66,4.28}
{4.27,4.68}
f1 (ppm)

Exemplary determination of the relative configuration of (E)-2-(oct-1-en-1-yl)-4,6-diphenyltetrahydro-2H-pyran

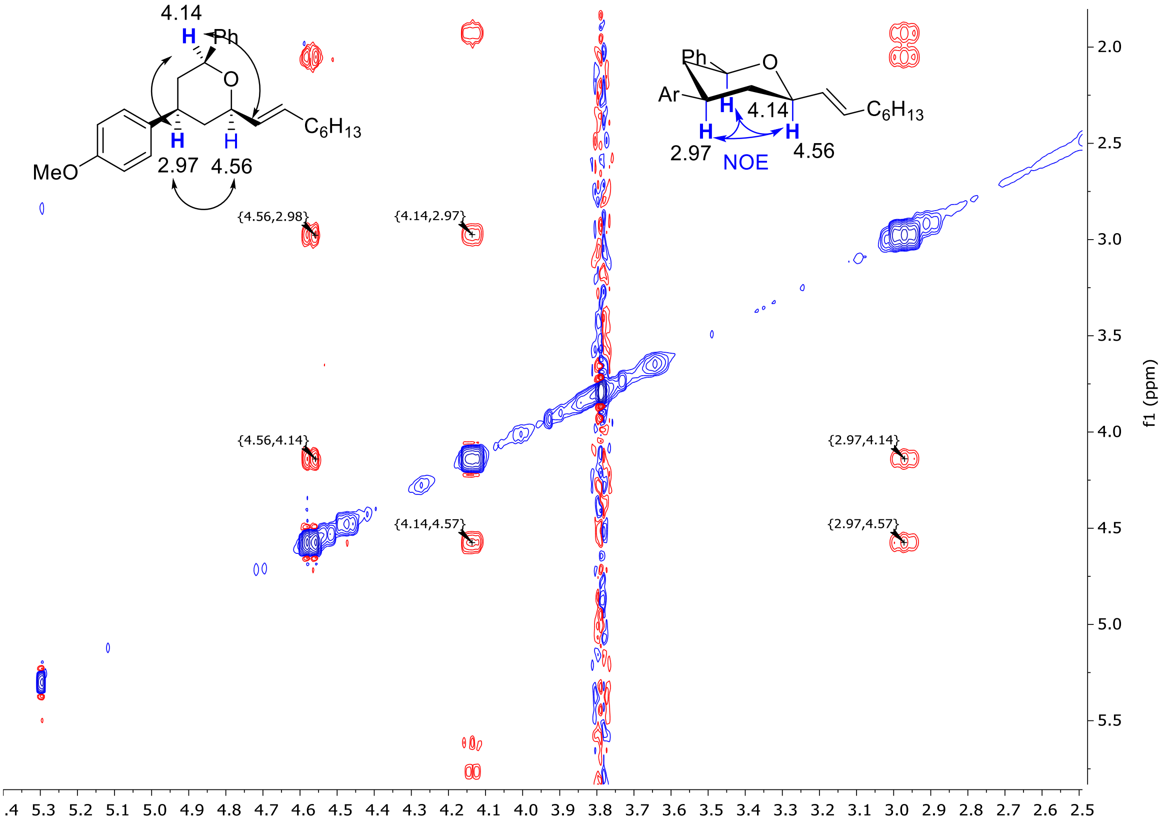

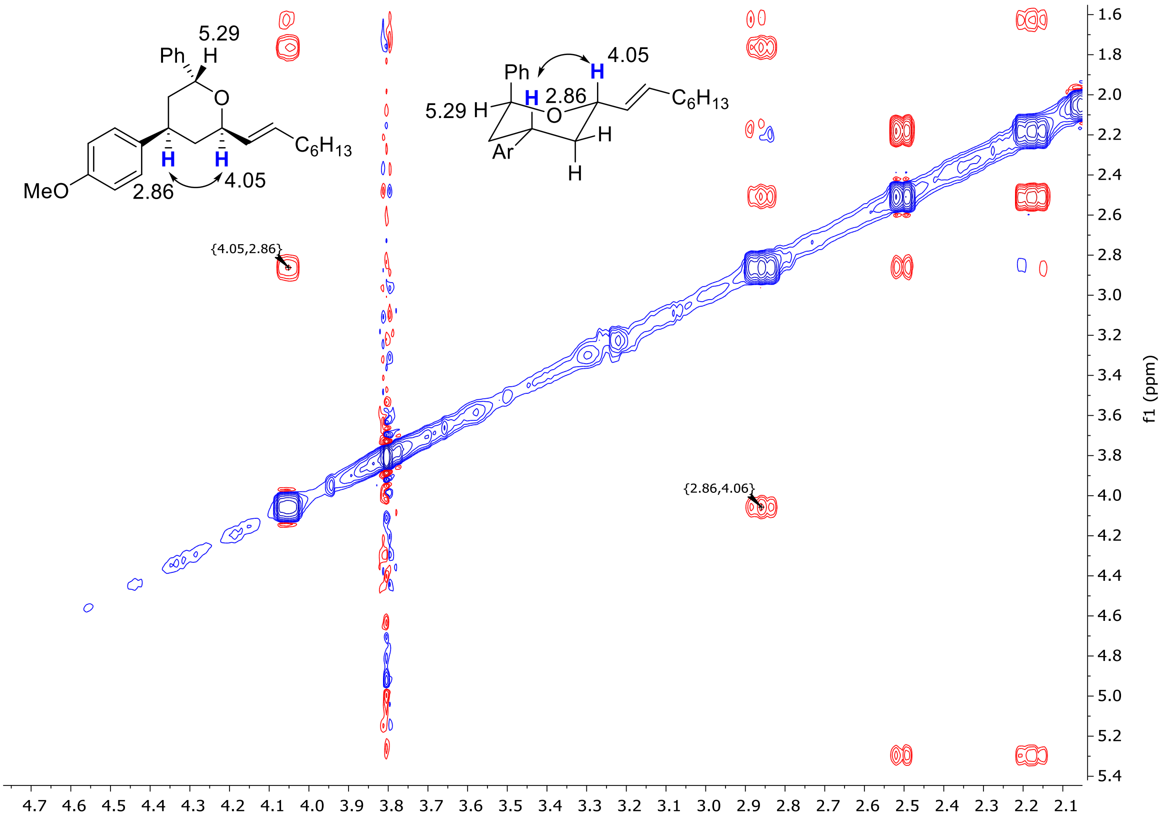

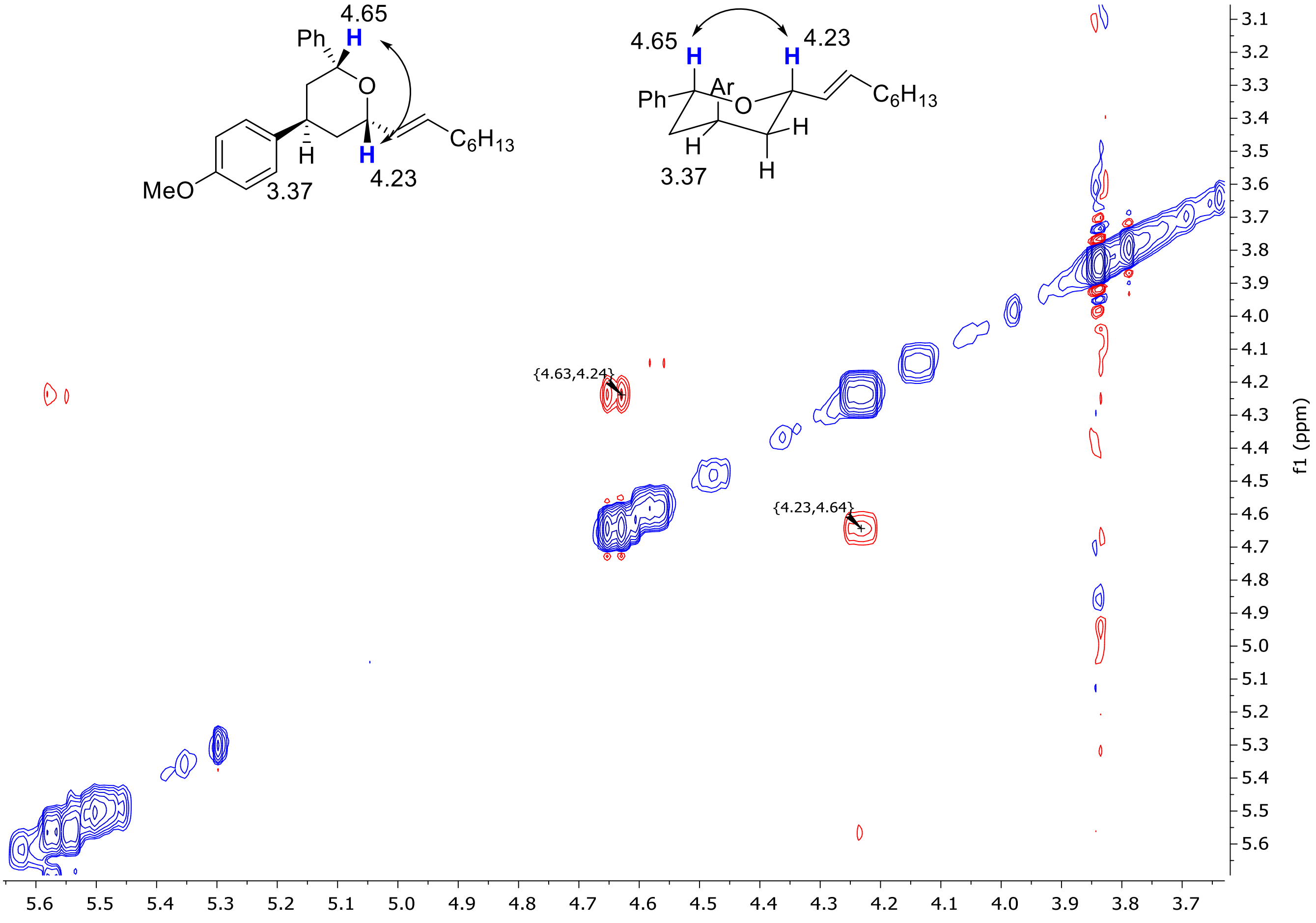

11 Ligand synthesis

Synthesis of 1, 1`-Bis[bis[*4*-(trifluoromethyl)phenyl]phosphino]ferrocene

Synthesis of bis(4-(trifluoromethyl)phenyl)phosphine oxide 134

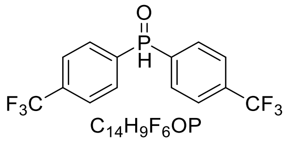

$C_{14}H_9F_6OP$
338.19

I) To a suspension of magnesium (2.60 g, 108 mmol, 2.1 equiv.) and iodide (catalytic amount) in Et_2O (88 mL, 1.0 M) 1-bromo-4-(trifluoromethyl)benzene (22.1 g, 13.8 mL, 98.2 mmol, 2.0 equiv.) was added dropwise. After the reaction stopped refluxing, the mixture was heated to 80 °C for 1 h.

II) To a suspension of NaH (2.16 g, 54.0mmol, 1.1 equiv. (60%)) in dry THF (30 mL) was carefully added diethylphosphite (67.9 g, 63.3 mL, 49.1 mmol, 1.0 equiv) dropwise. After completed addition the mixture was stirred for 1 h and then cooled to 0 °C. The Grignard solution (**step I**) was added the reaction was allowed to warm to room temperature and was then refluxed for 30 minutes. The reaction mixture was cooled to room temperature and stirred for 3 h before it was quenched through the addition of H_2O (100

mL) nad conc. HCl (100mL). The layers were separated, and the aqueous layer was extracted with EE (3 x 50 mL). The combined organic phases were dried over Na_2SO_4 and the solvent was removed under reduced pressure. The crude product was purified by flash chromatography on silica gel (DCM to DCM/EE = 90/10) the residue was recrystallized from Et_2O to obtain the pure title combined as white solid (12.5 g, 36.8 mol, 75%) using a mixture of pentane/ether.

Analytical Data

^{1}H-NMR (500.1 MHz, CDCl$_3$): δ = 7.77 – 7.82 (m, 4H), 7.83 – 7.91 (m, 4H), 8.19 (d, J = 491.6 Hz 1H) ppm.

^{13}C-NMR (125.6 MHz, CDCl$_3$): δ = 126.05, 126.0, 126.1, 126.1, 126.1, 131.2, 131.3, 134.5 ppm.

^{19}F-NMR (470.5, CDCl$_3$): δ = -63.4 ppm.

^{31}P-NMR (201.4, CDCl$_3$): δ = 17.8 ppm.

ESI-HRMS: *m/z* calcd for $C_{15}H_{10}OF_3$ $[M+H]^+$ 339.0373 found 339.0385.

Synthesis of 1, 1`-Bis[bis[*4*-(trifluoromethyl)phenyl]phosphino]ferrocene

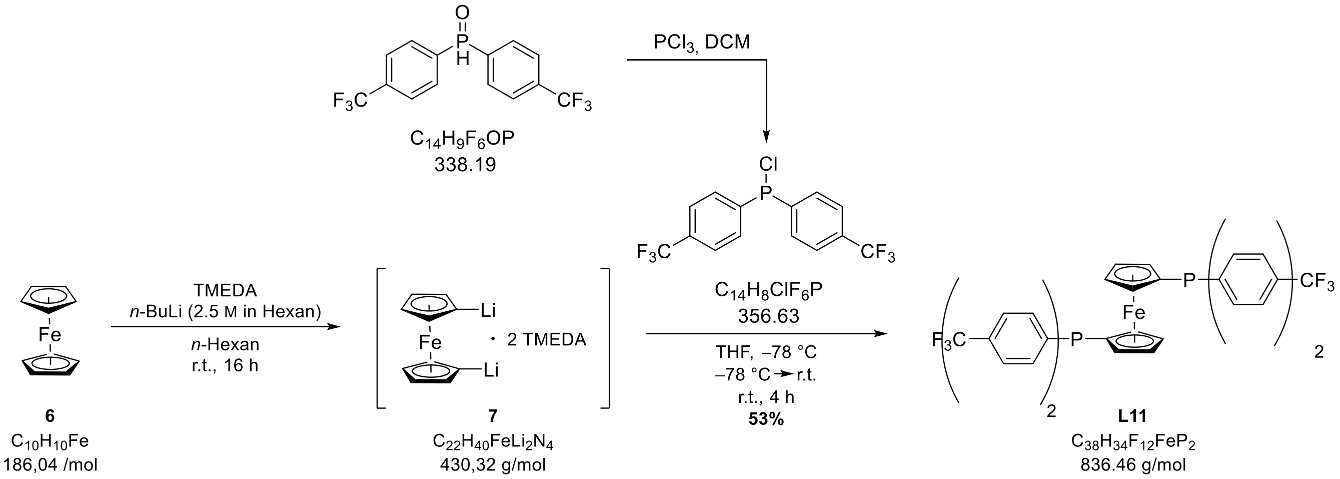

I) To a solution of bis(4-(trifluoromethyl)phenyl)phosphine oxide (548 mg, 1.62 mmol, 1.0 equiv.) in dry DCM (10 mL) was added PCl_3 (111mg, 71 μL, 0.81 mmol, 0.50 equiv.) at room temperature. The mixture was stirred at this temperature until the reaction was complete as indicated by TLC. The solvent was removed under reduced pressure and the crude chlorobis(4-(trifluoromethyl)phenyl)phosphane was used without further purification in the net reaction step.

II) N,N,N',N'-Tetramethylethylenediamine (290 μL, 1.34 mmol, 2.50 equiv.) and n-BuLi (2.5 M in Hexane, 480 μL, 1.19 mmol, 2.2 equiv.) were added dropwise to a stirred solution of $(C_5H_5)_2Fe$ (100 mg, 0.540 mmol, 1.0 equiv.) in hexane (10 mL) under a argon atmosphere at room temperature. The solution was stirred at room temperature overnight. The orange slurry was allowed to settle, and the hexane layer was removed with a syringe. The remaining orange powder was washed with dry hexane (5 mL) and dissolved in dry THF (10 mL). Chlorobis(4-(trifluoromethyl)phenyl)phosphane (578 mg, 1.62 mmol, 3.0

equiv.) was diluted with dry THF (2 mL) and added to the orange solution at –78 °C. The solution was slowly warmed to room temperature and stirred for 4 hours. The reaction was quenched with water and diluted with DCM (10 mL). The layers were separated and the aqueous layer was extracted with DCM (3 x 10 mL). The combined organic phases were dried over Na_2SO_4 and the solvent was removed under reduced pressure. The crude product was purified by flash chromatography on silica gel (pentane/Et_2O = 60/1) the orange residue was recrystallized in heptane to obtain the pure title combined as orange needles (149 mg, 0.178 mmol, 33%) using a mixture of pentane/ether.

Analytical Data

^{1}H-NMR (500.1 MHz, $CDCl_3$): δ = 3.99 (t, *J* = 1.8 Hz, 2H), 4.29 – 4.37 (m, 2H), 7.38 (d, *J* = 8.2 Hz, 4H), 7.55 (d, *J* = 8.1 Hz, 4H) ppm.

^{13}C-NMR (125.6 MHz, $CDCl_3$): δ = 72.9, 72.9, 74.0, 75.0, 125.1, 125.1, 125.2, 125.2, 131.2, 133.6, 133.8, 142.9, 143.0 ppm.

^{19}F-NMR (470.5, $CDCl_3$): δ = -62.8 ppm.

^{31}P-NMR (201.4, $CDCl_3$): δ = -17.2 ppm.

ESI-HRMS: *m/z* calcd for $C_{38}H_{35}O_2F_{12}FeP_2$ $[M+H]^+$ 827.0589 found 827.0561.

Synthesis of 1, 1`-Bis[bis[3,5-bis(trifluoromethyl)phenyl]phosphino]ferrocene

Synthesis of bis(3,5-bis(trifluoromethyl)phenyl)phosphine oxide 135

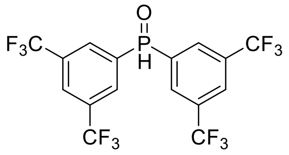

$C_{16}H_7F_{12}OP$
474.19

I) To a suspension of magnesium (2.60 g, 108 mmol, 2.1 equiv.) and iodide (catalytic amount) in Et_2O (88 mL, 1.0 M) 1-bromo-3,5-bis(trifluoromethyl)benzene (28.7 g, 18.6 mL, 98.2 mmol, 2.0 equiv.) was added dropwise. After the reaction stopped refluxing, the mixture was heated to 80 °C for 1 h.

II) To a suspension of NaH (2.16 g, 54.0mmol, 1.1 equiv. (60%)) in dry THF (30 mL) was carefully added diethylphosphite (67.9 g, 63.3 mL, 49.1 mmol, 1.0 equiv) dropwise. After completed addition the mixture was stirred for 1 h and then cooled to 0 °C. The Grignard solution (**step I**) was added the reaction was allowed to warm to room temperature and was then refluxed for 30 minutes. The reaction mixture was cooled to room temperature and stirred for 3 h before it was quenched through the addition of H2O (100 mL) nad conc. HCl (100mL). The layers were separated, and the aqueous layer was extracted with EE (3 x 50 mL). The combined organic phases were dried over Na_2SO_4 and the solvent was removed under reduced pressure. The crude product was purified by flash chromatography on silica gel (DCM to

DCM/EE = 90/10) the residue was recrystallized from Et_2O to obtain the pure title combined as white solid (16.3g, 34.7 mol, 70%) using a mixture of pentane/ether.

Analytical Data

^{1}H-NMR (500.1 MHz, $CDCl_3$): δ =8.3 (d, *J* = 504.4 Hz, 1H), 8.13 – 8.16 (m, 2H), 8.21 (d, *J* = 1.6 Hz, 2H), 8.81 (s, 1H) ppm.

^{13}C-NMR (125.6 MHz, $CDCl_3$): δ = 19.3, 121.5, 123.6, 125.8, 127.3, 127.3, 127.4, 127.4, 130.8, 130.8, 130.9, 130.9, 132.7, 133.5 ppm.

^{19}F-NMR (470.5, $CDCl_3$): δ = -63.1 ppm.

^{31}P-NMR (201.4, $CDCl_3$): δ = -14.3 ppm.

ESI-HRMS: *m/z* calcd for $C_{16}H_8OF_3P$ $[M+H]^+$ 475.01116 found 475.01113.

Synthesis of 1,1′-Bis[bis[3,5-bis(trifluormethyl)phenyl]phosphino]ferrocen (L5) 136

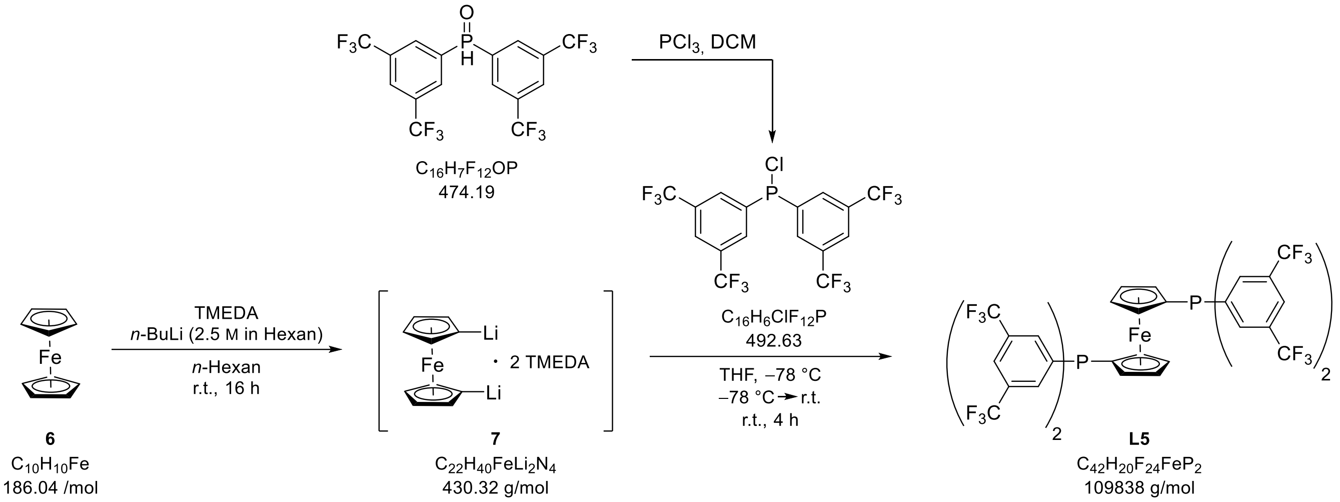

I) To a solution of bis(3,5-bis(trifluoromethyl)phenyl)phosphine oxide (769 mg, 1.62 mmol, 1.0 equiv.) in dry DCM (10 mL) was added PCl_3 (111mg, 71 μL, 0.81 mmol, 0.50 equiv.) at room temperature. The mixture was stirred at this temperature until the reaction was complete as indicated by TLC. The solvent was removed under reduced pressure and the crude bis(3,5-bis(trifluoromethyl)phenyl)chlorophosphane was used without further purification in the net reaction step.

II) N,N,N’,N’-Tetramethylethylenediamine (290 μL, 1.34 mmol, 2.50 equiv.) and n-BuLi (2.5 M in Hexane, 480 μL, 1.19 mmol, 2.2 equiv.) were added dropwise to a stirred solution of $(C_5H_5)_2Fe$ (100 mg, 0.540 mmol, 1.0 equiv.) in hexane (10 mL) under a argon atmosphere at room temperature. The solution was stirred at room temperature overnight. The orange slurry was allowed to settle, and the hexane layer was removed with a syringe. The remaining orange powder was washed with dry hexane (5 mL) and dissolved in dry THF (10 mL). Bis(3,5-bis(trifluoromethyl)phenyl)chlorophosphane (798 mg, 1.62 mmol, 3.0 equiv.) was diluted with dry THF (2 mL) and added to the orange solution at –78 °C. The solution

was slowly warmed to room temperature and stirred for 4 hours. The reaction was quenched with water and diluted with DCM (10 mL). The layers were separated, and the aqueous layer was extracted with DCM (3 x 10 mL). The combined organic phases were dried over Na_2SO_4 and the solvent was removed under reduced pressure. The crude product was purified by flash chromatography on silica gel (pentane/Et_2O = 60/1) the orange residue was recrystallized in heptane to obtain the pure title combined as orange needles (178 mg, 0.162 mmol, 30%) using a mixture of pentane/ether.

^{1}H NMR (500.1 MHz, $CDCl_3$): δ = 4.01 (m, 4H), 4.43 – 4.45 (m, 4H), 7.69 (d, J = 1.6 Hz, 4H), 7.70 (d, J = 1.6 Hz, 4H), 7.88 – 7.90 (m, 4H) ppm.
^{13}C NMR (125.6 MHz, $CDCl_3$): δ = 73.4, 73.7, 73.9, 119.8, 121.9, 123.4, 123.5, 123.5, 123.5, 123.6, 124.1, 126.3, 131.8, 132.0, 132.1, 132.3, 132.3, 132.6, 132.9, 133.0, 133.1, 133.1, 140.3.
^{19}F NMR (470.5 MHz, $CDCl_3$): δ = -63.0 ppm.
^{31}P NMR (201.4 MHz, $CDCl_3$) δ = -15.6 ppm.
ESI-HRMS: *m/z* calcd for $C_{42}H_{21}O_2F_{24}FeP_2$ $[M+H]^+$ 1130.9977 found 1130.9980.

12 NMR

ethyl penta-3,4-dienoate 60

^{1}H-NMR

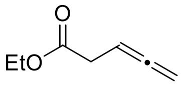

$C_7H_{10}O_2$
126.16

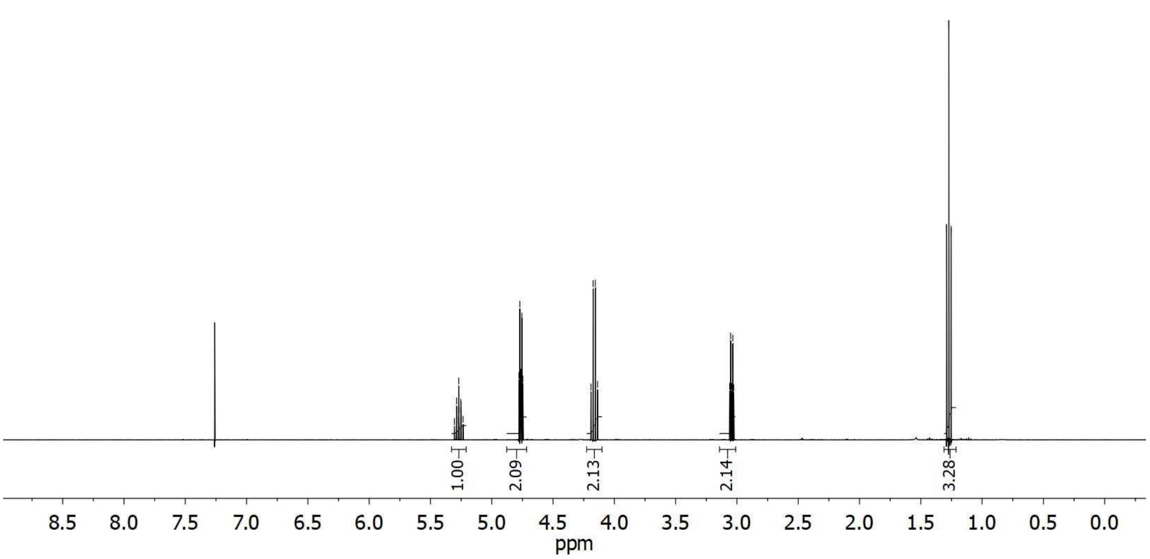

^{13}C-NMR

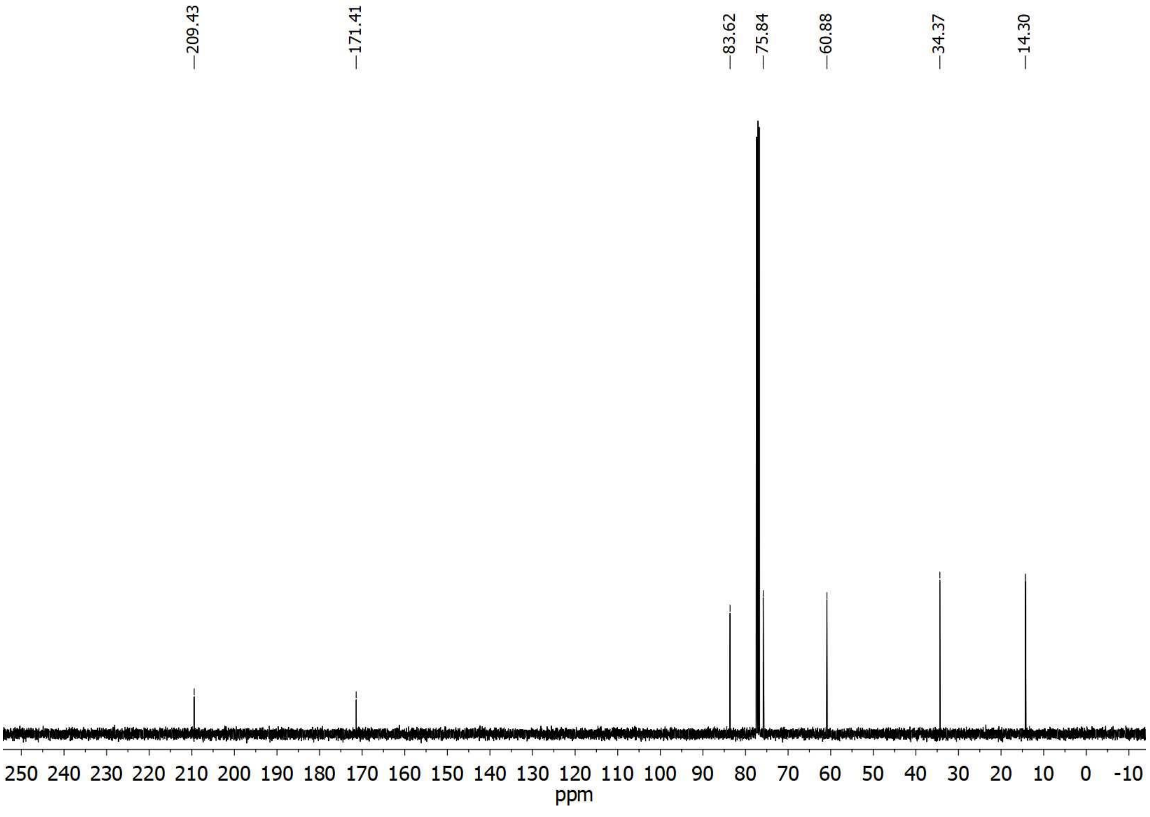

penta-3,4-dienal 61

^{1}H-NMR

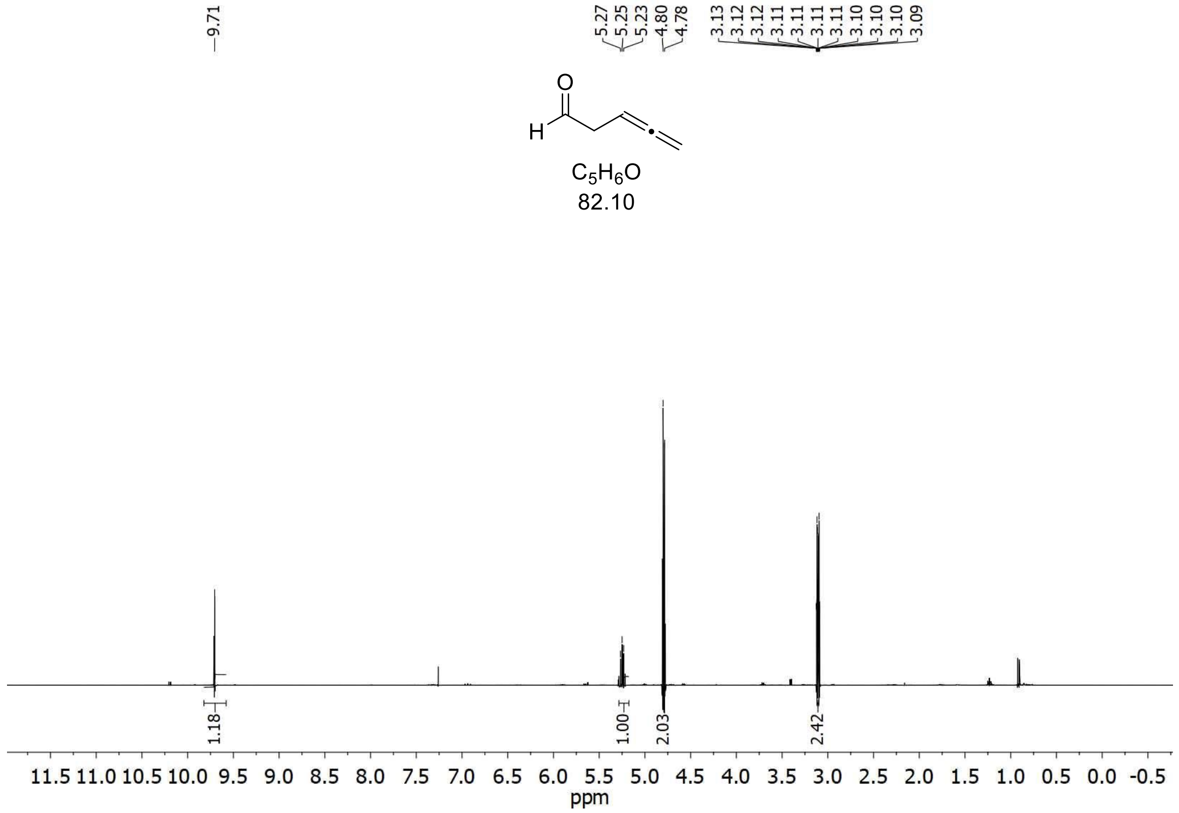

^{13}C-NMR

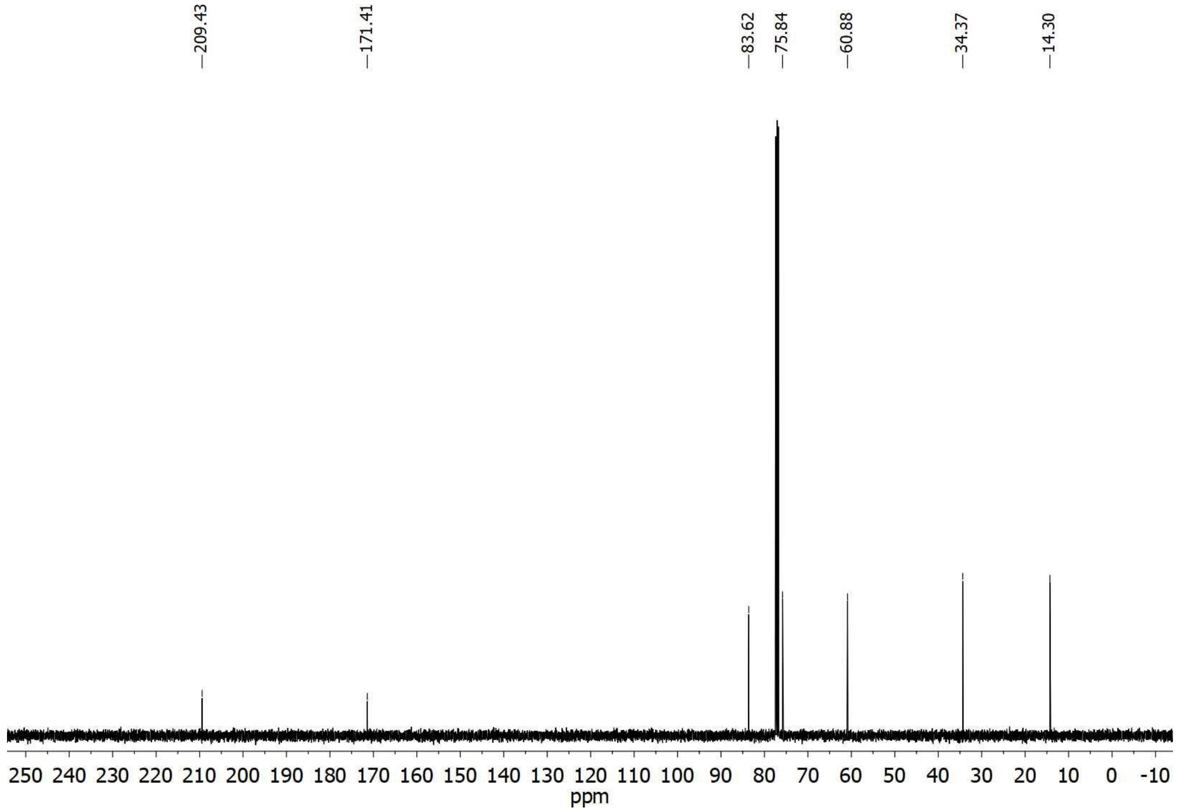

ethyl (*E*)-hepta-2,5,6-trienoate 62

^{1}H-NMR

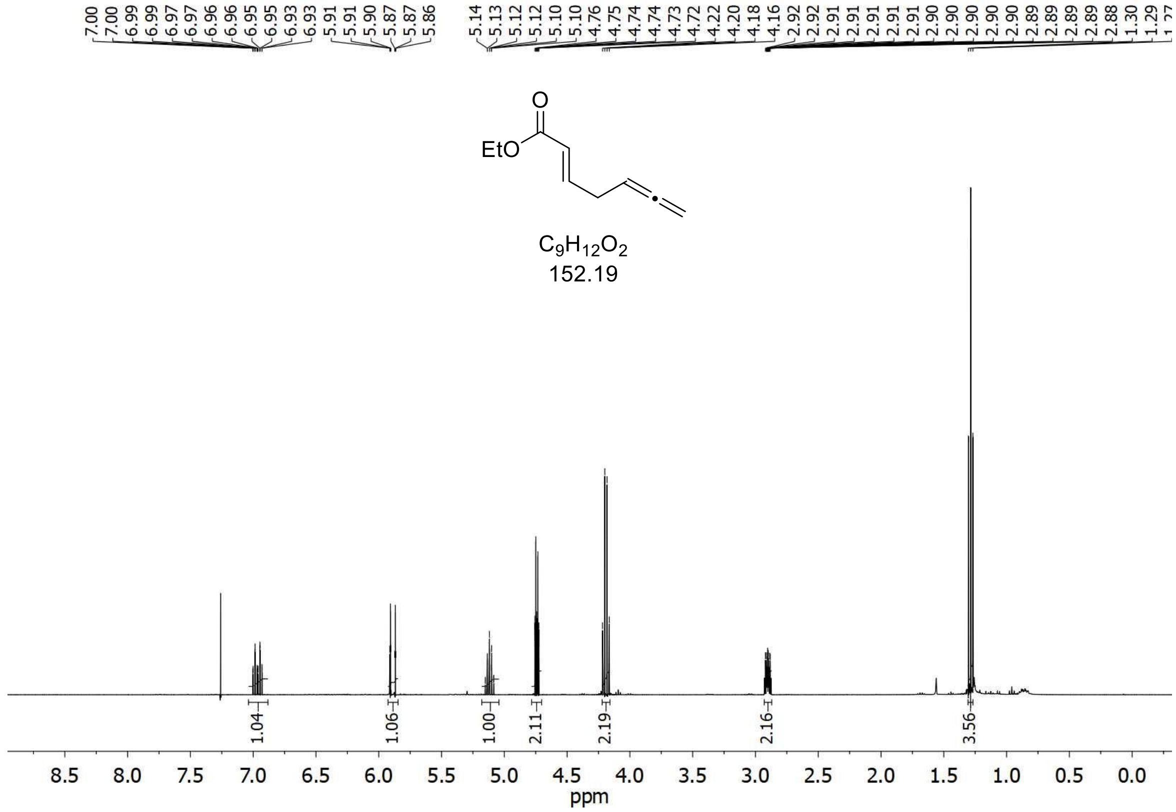

^{13}C-NMR

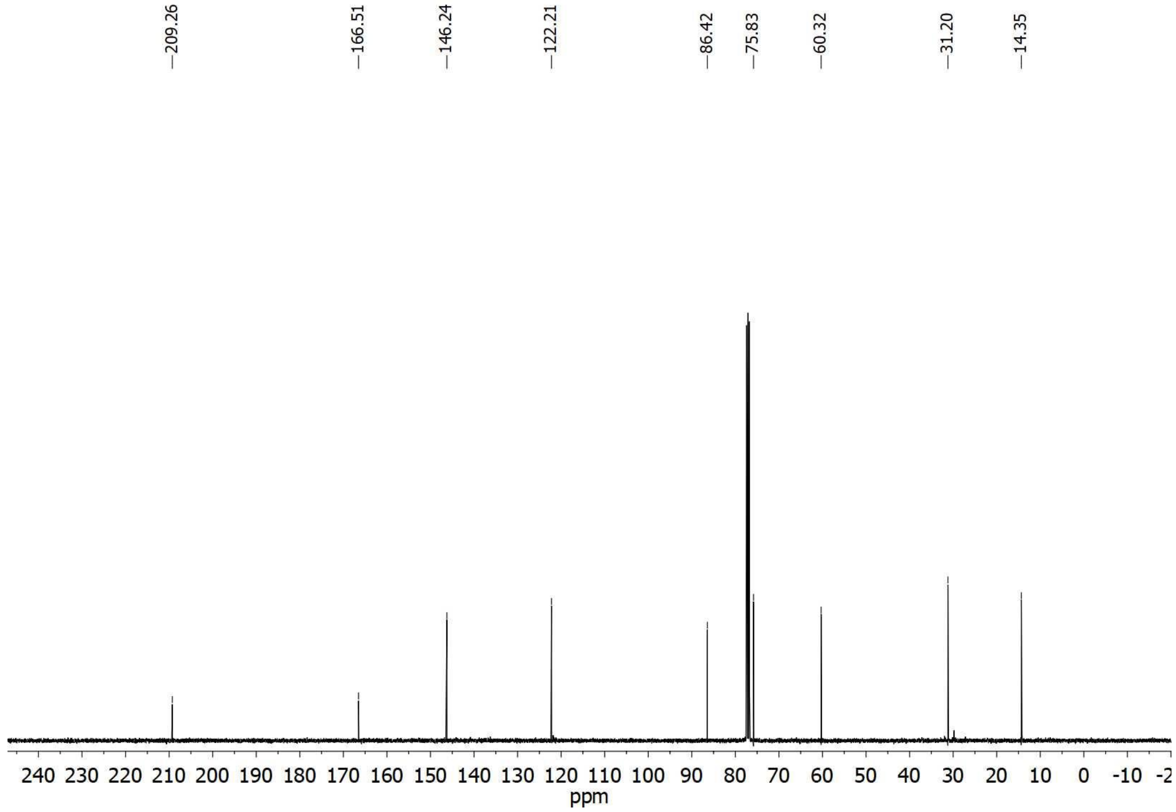

ethyl 3-methylhepta-5,6-dienoate 63

^{1}H-NMR

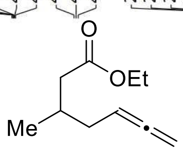

$C_{10}H_{16}O_2$
168.24

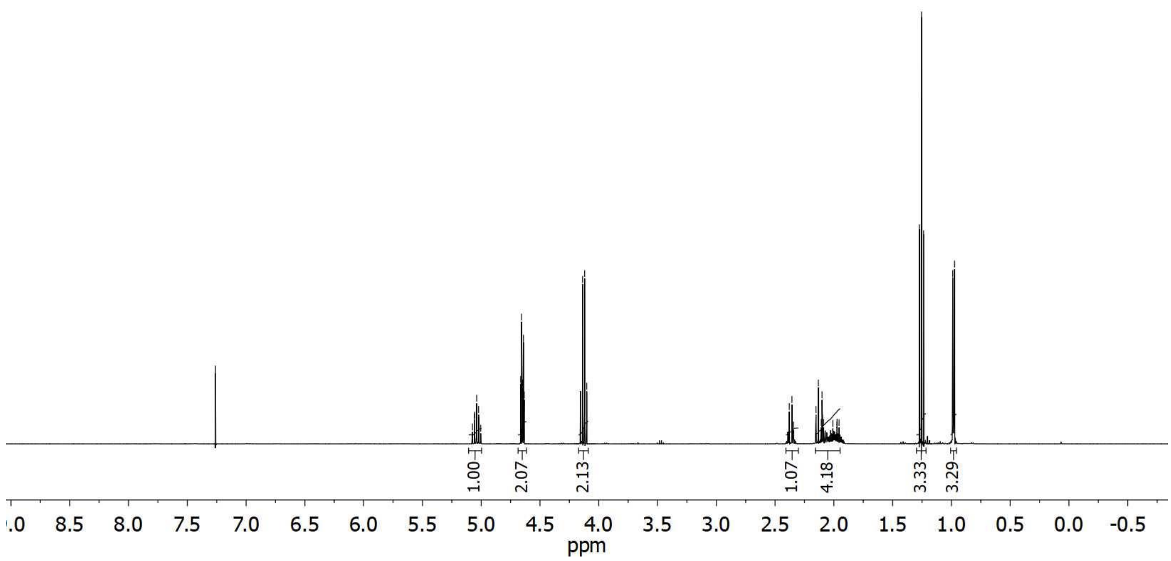

^{13}C-NMR

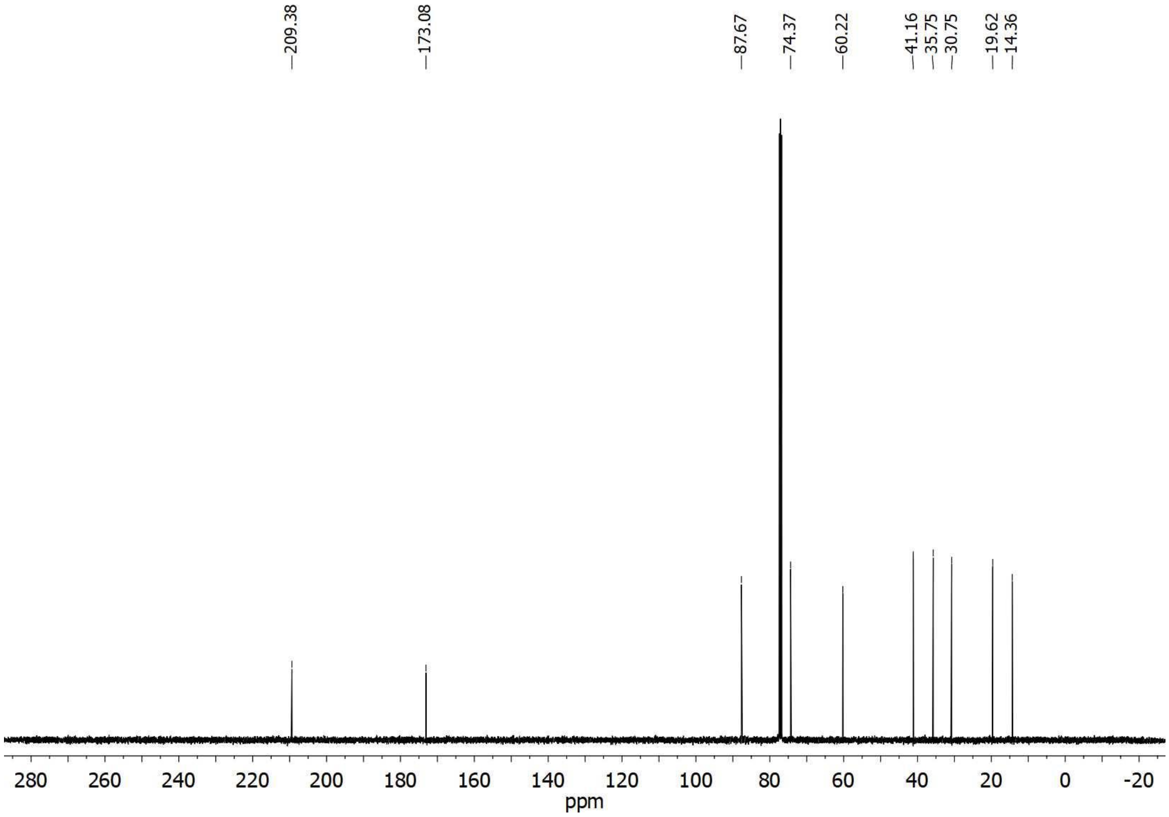

3-methylhepta-5,6-dien-1-ol 53rac

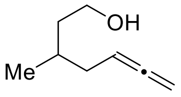

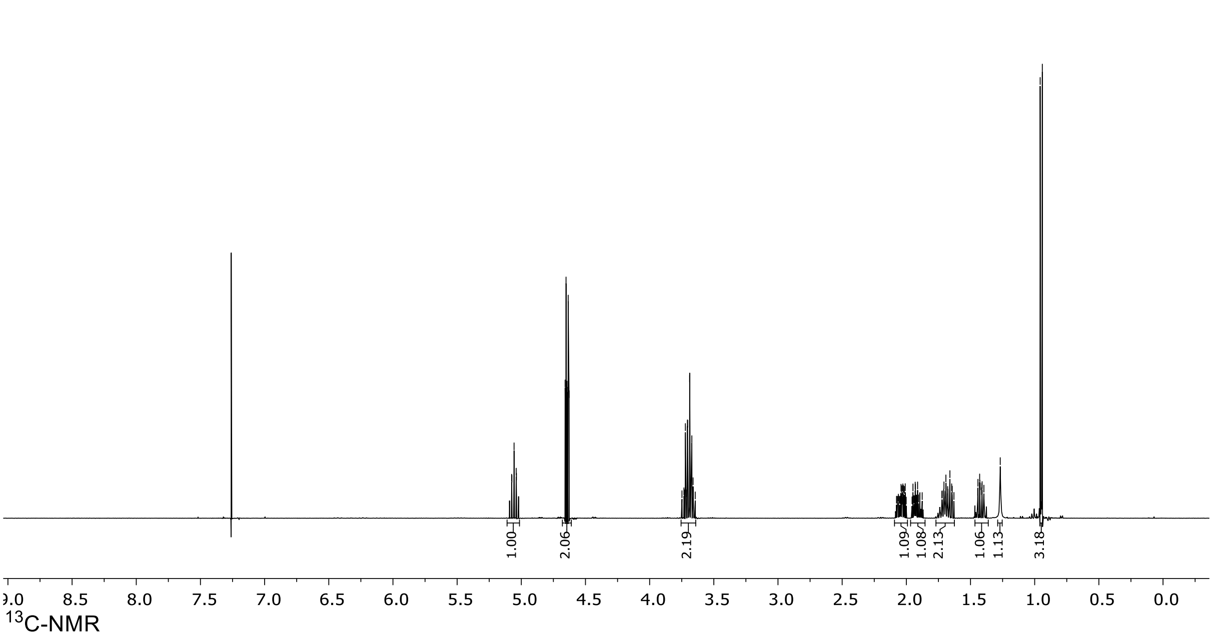

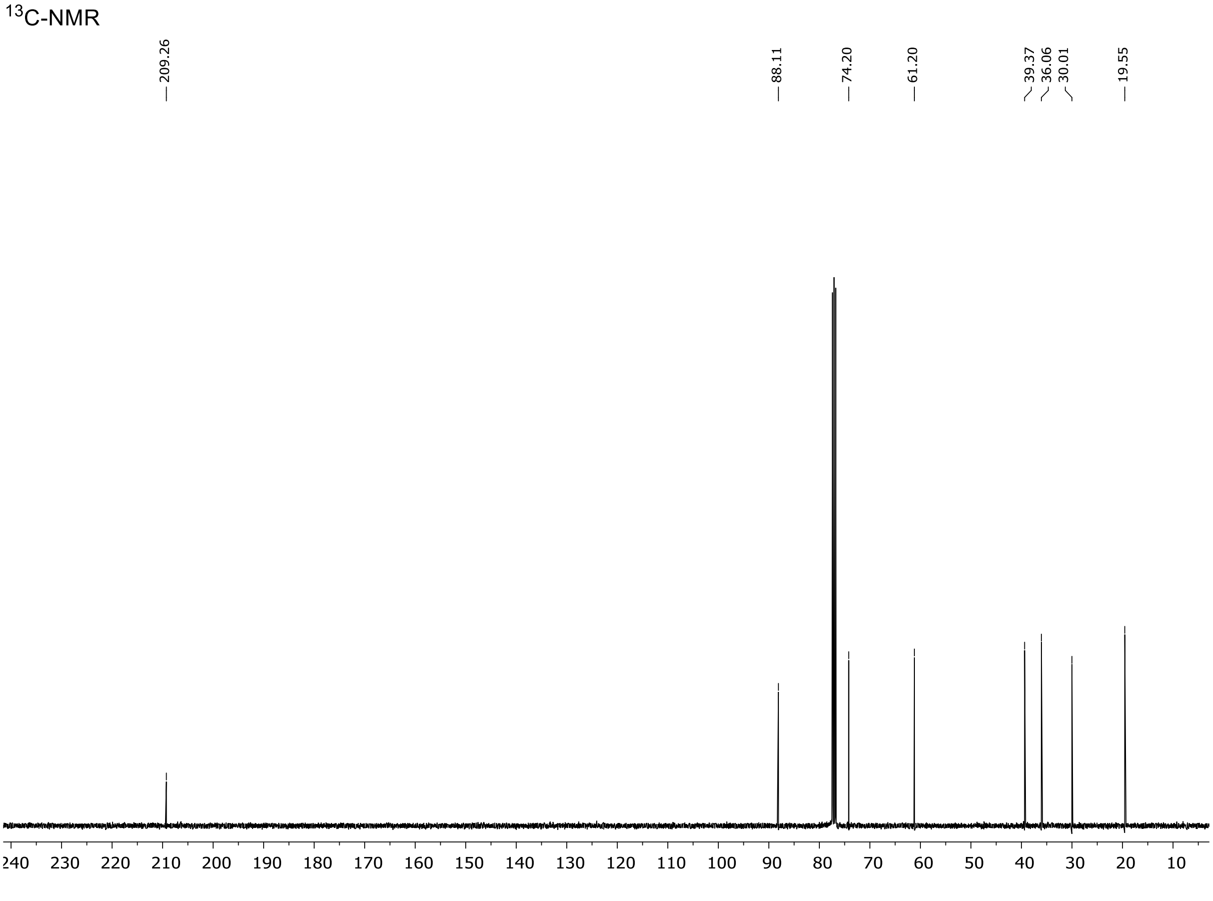

3-(buta-2,3-dien-1-yl)tetradecanol 64

^{1}H-NMR

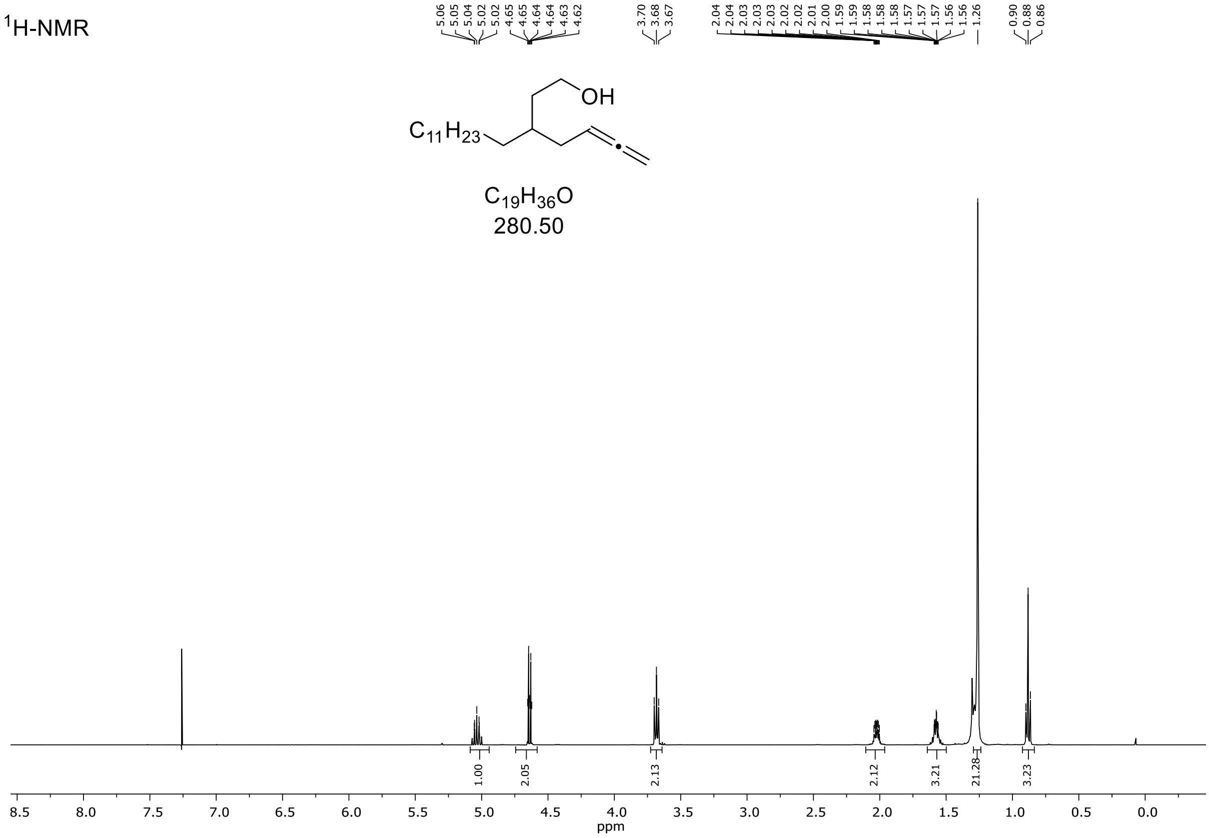

^{13}C-NMR

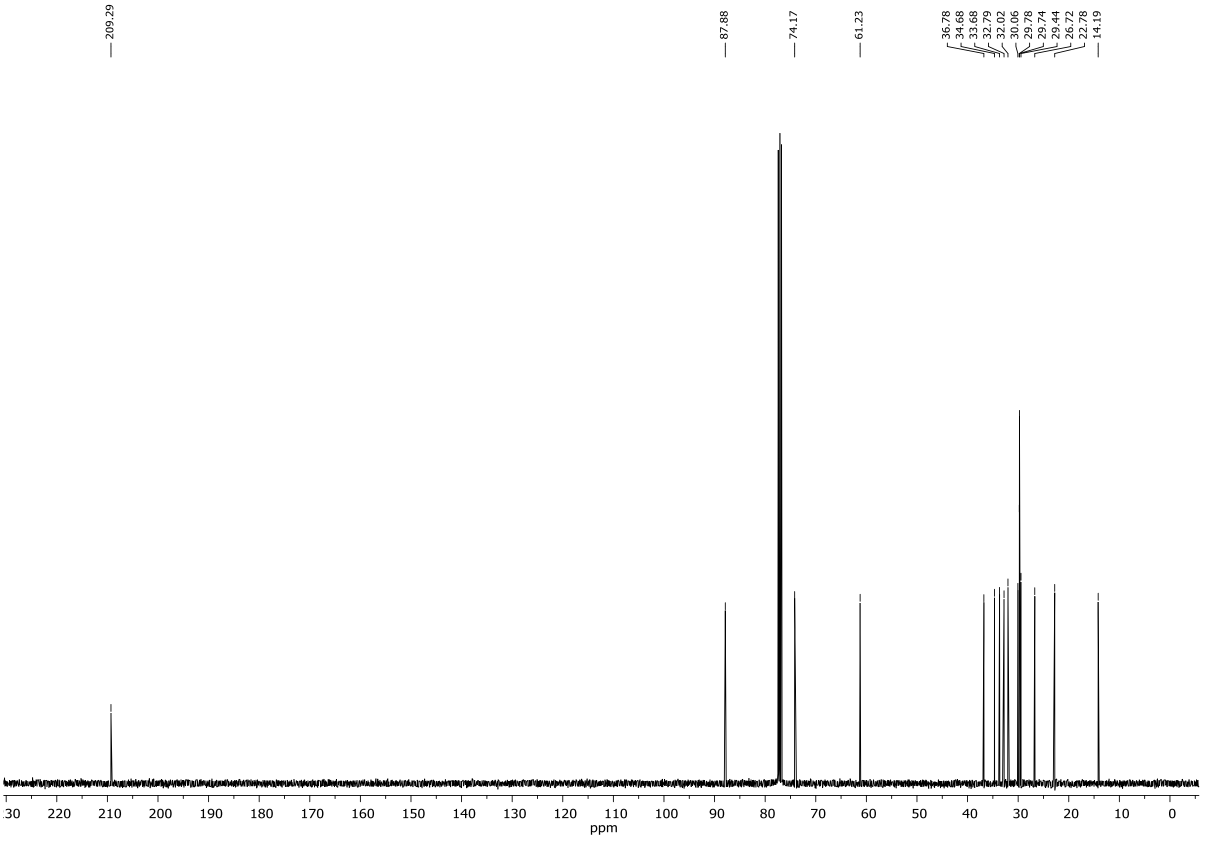

3-neopentylhepta-5,6-dien-1-ol 65

^{1}H-NMR

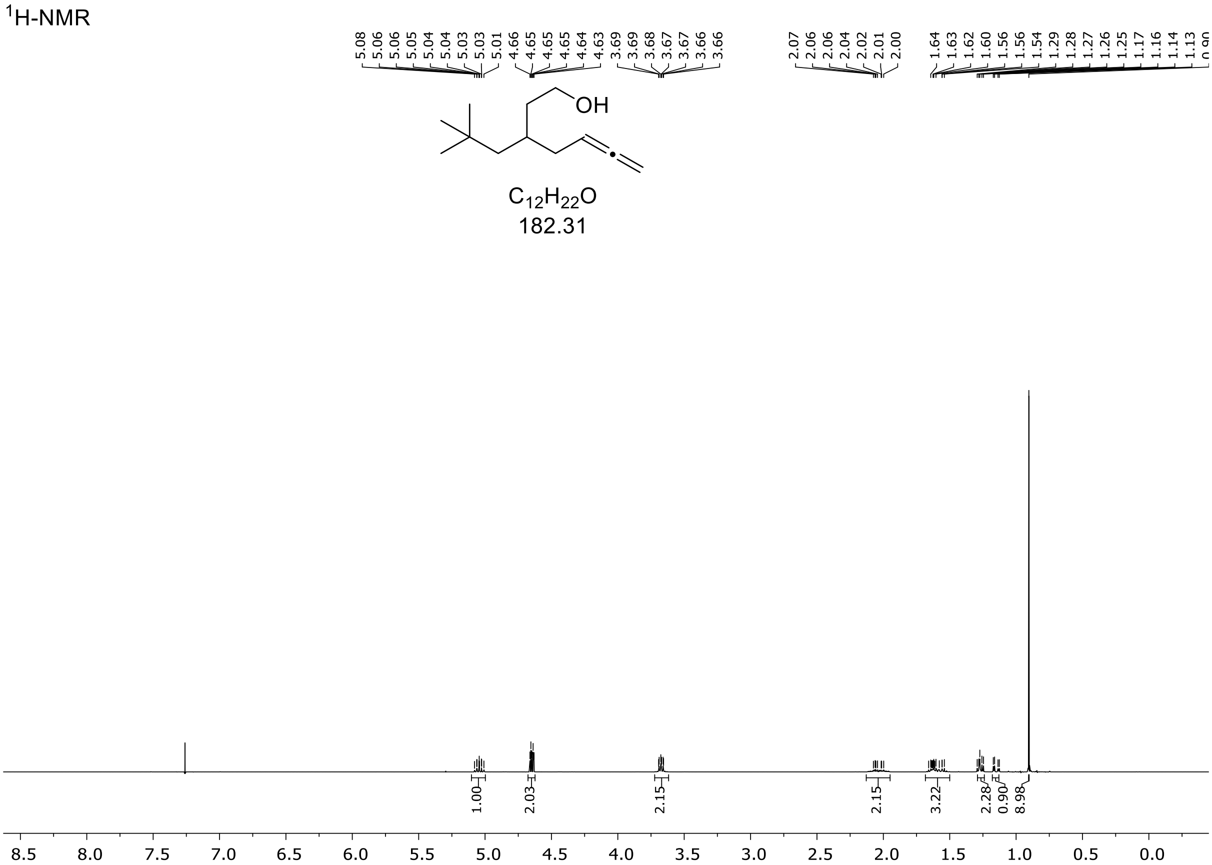

^{13}C-NMR

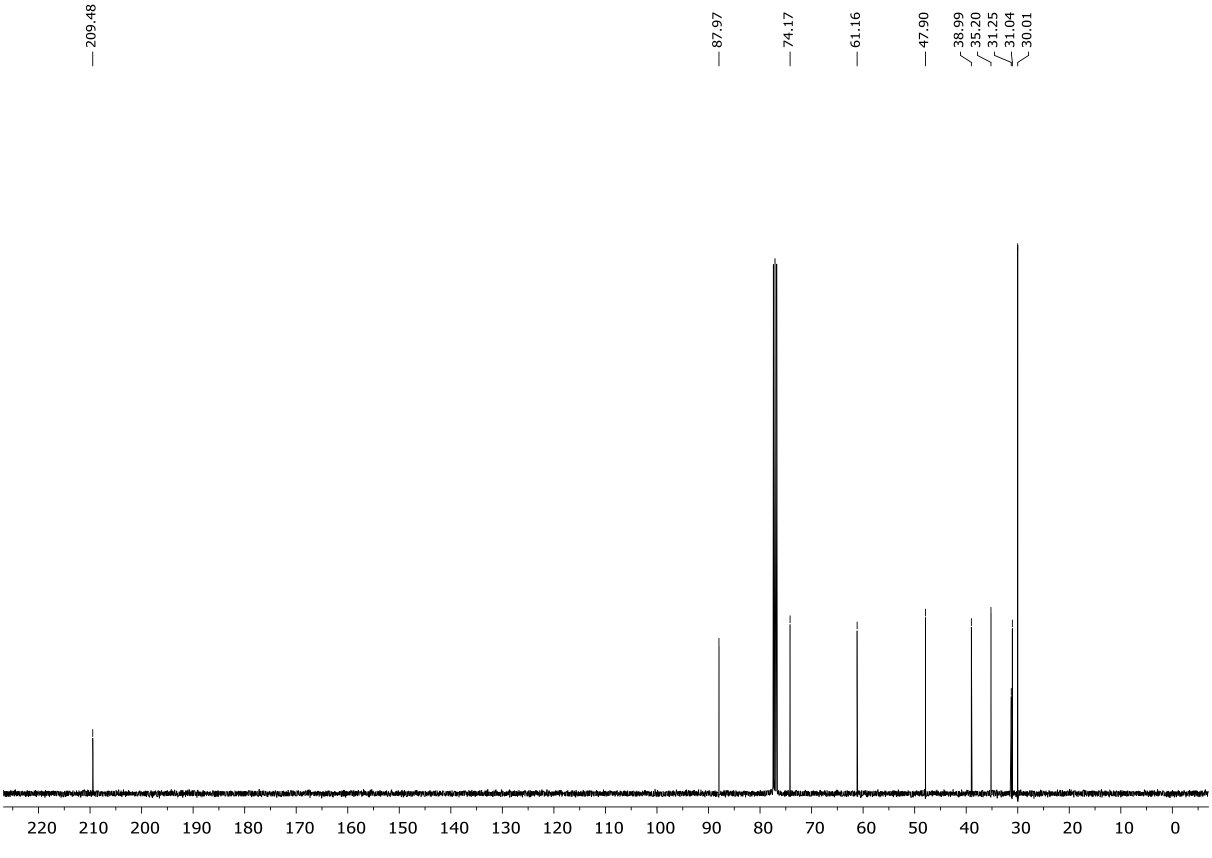

ethyl 3-cyclopropylhepta-5,6-dienoate 66

^{1}H-NMR

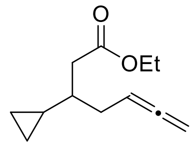

$C_{12}H_{18}O_2$
194.27

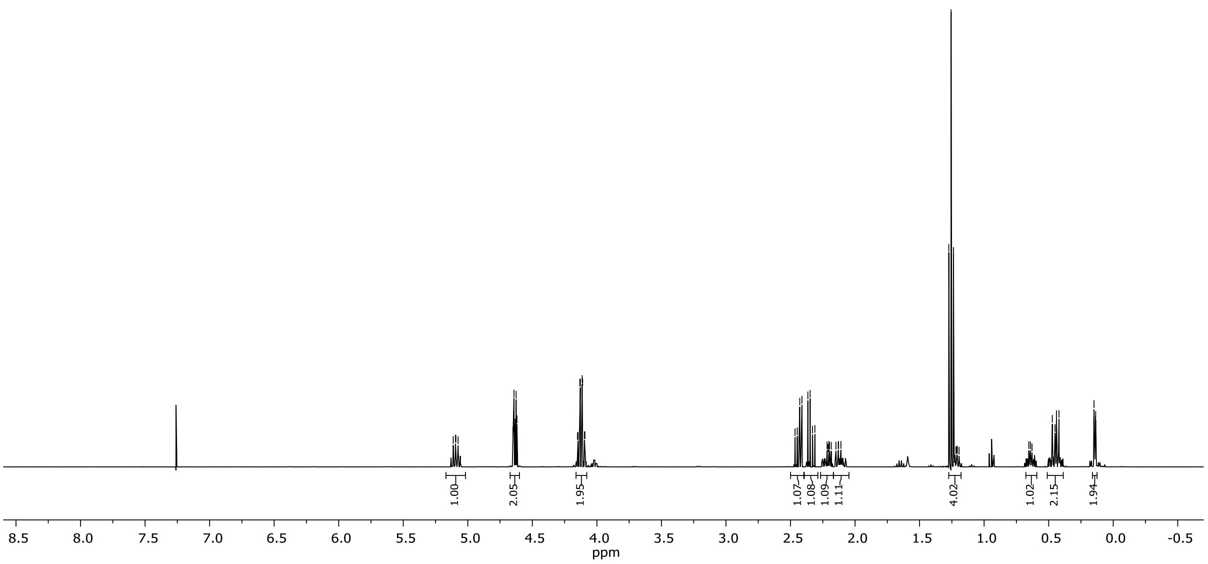

^{13}C-NMR

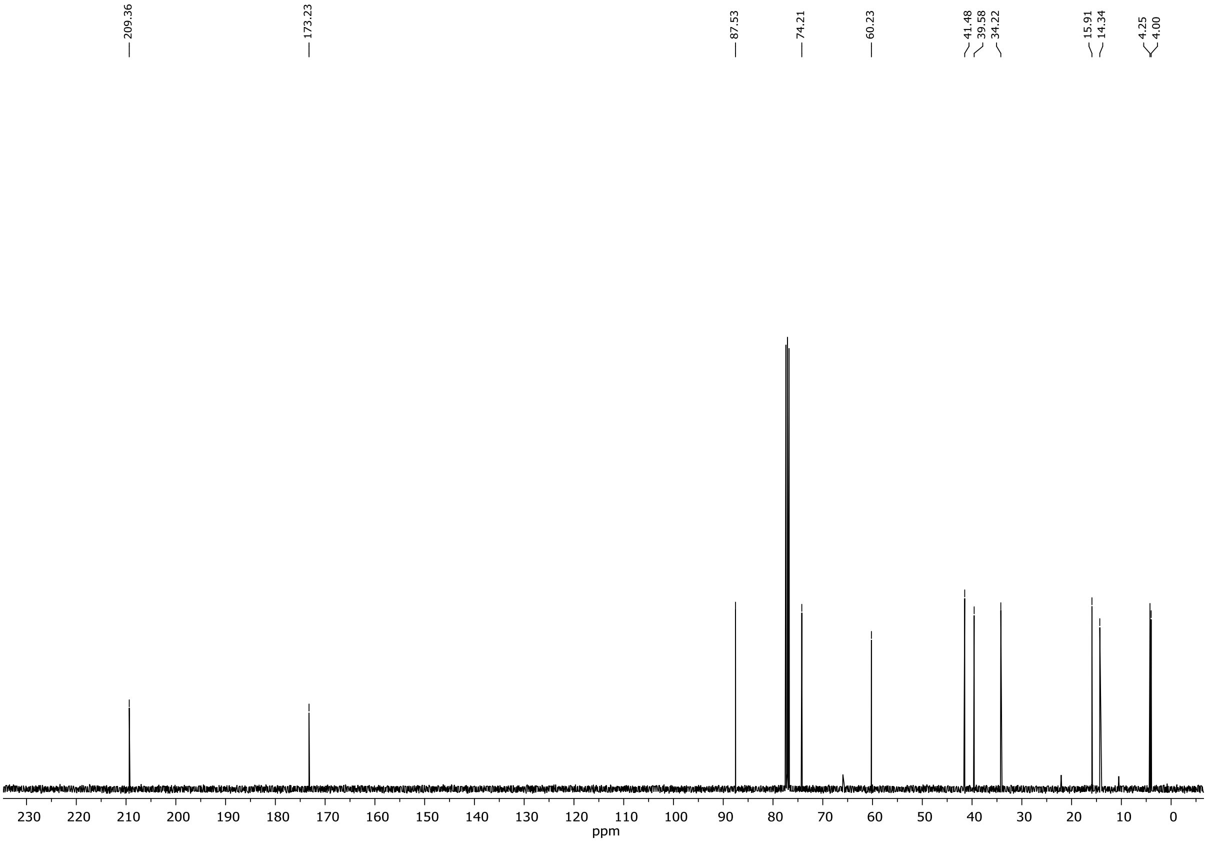

3-cyclopropylhepta-5,6-dienol 67

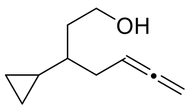

$C_{10}H_{16}O$
152.24

^{1}H-NMR

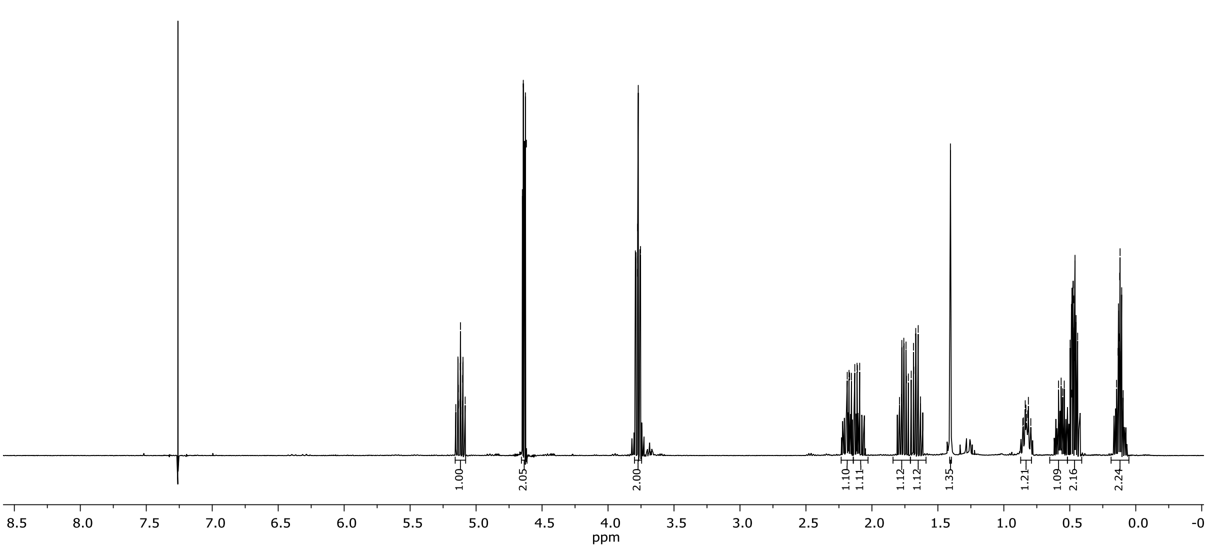

^{13}C-NMR

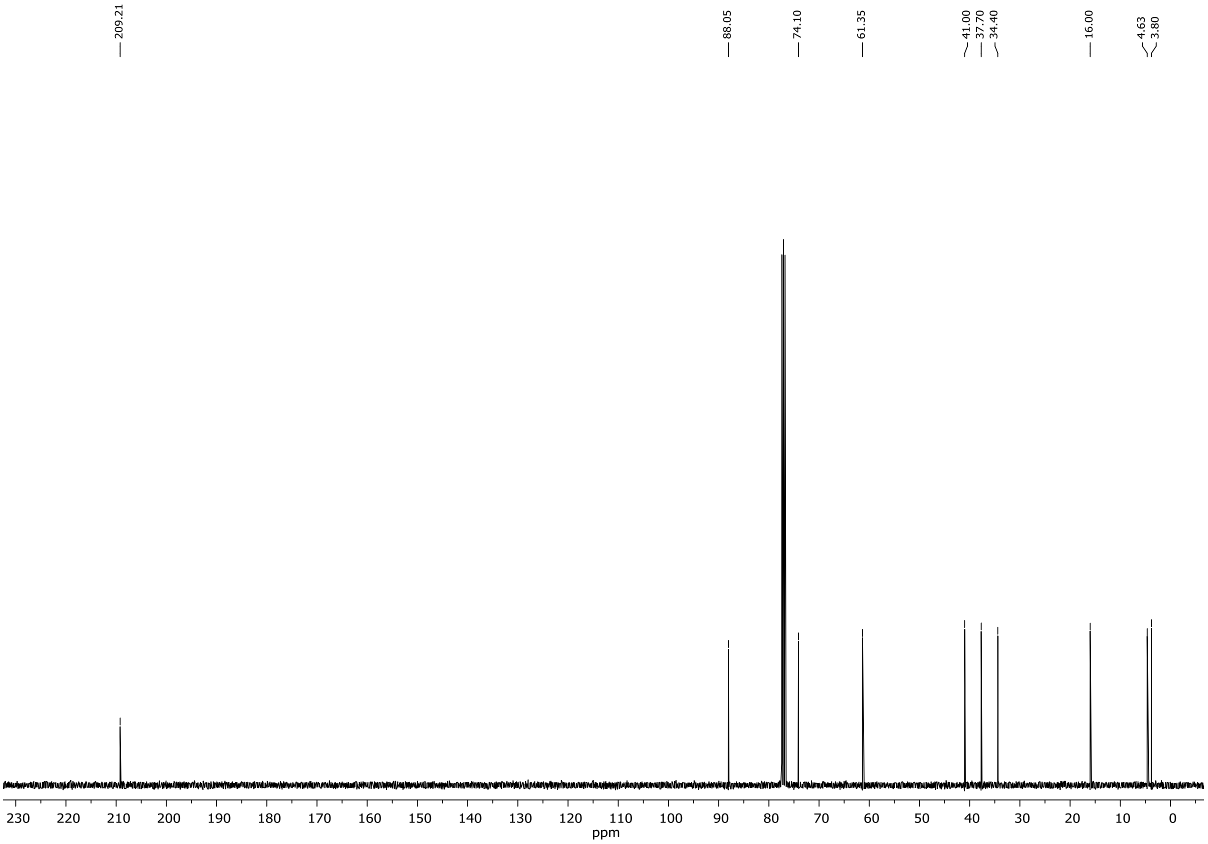

3-cyclohexylhepta-5,6-dien-1-ol 68

^{1}H-NMR

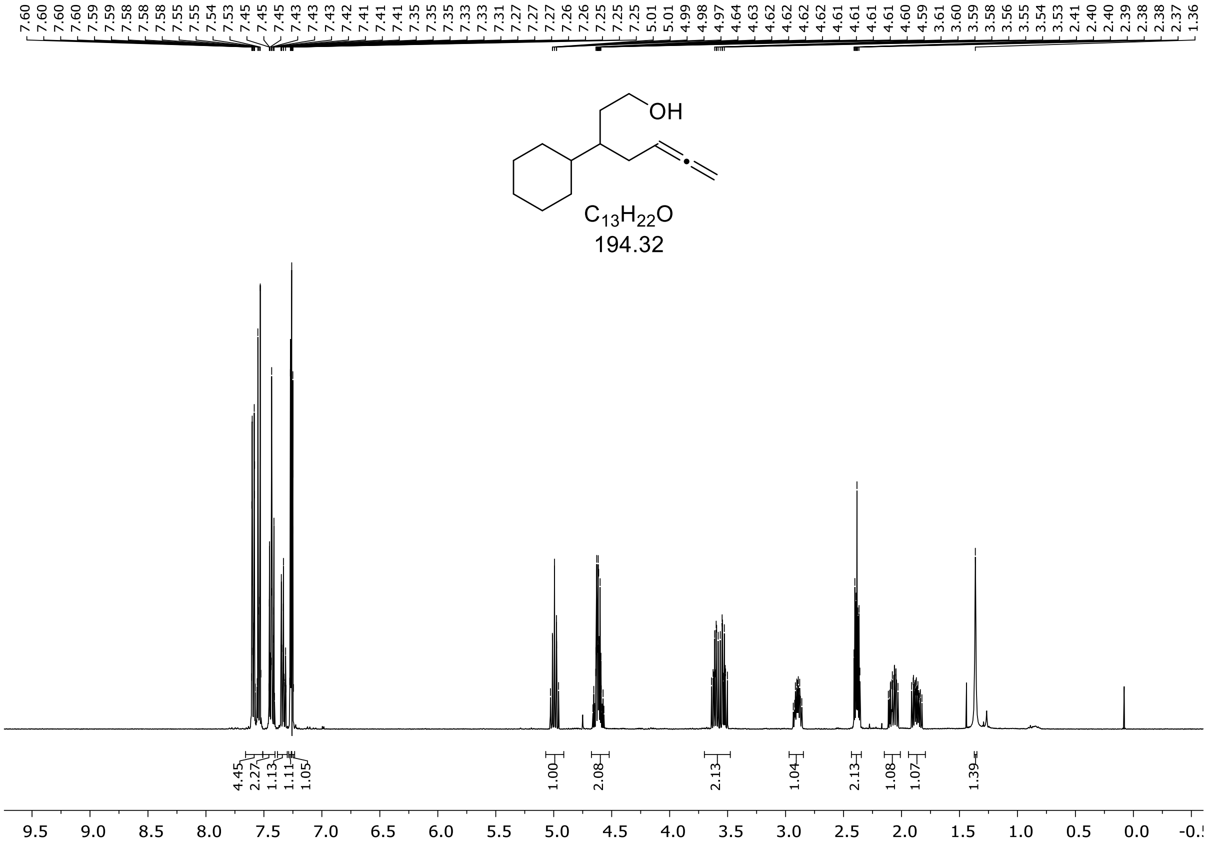

^{13}C-NMR

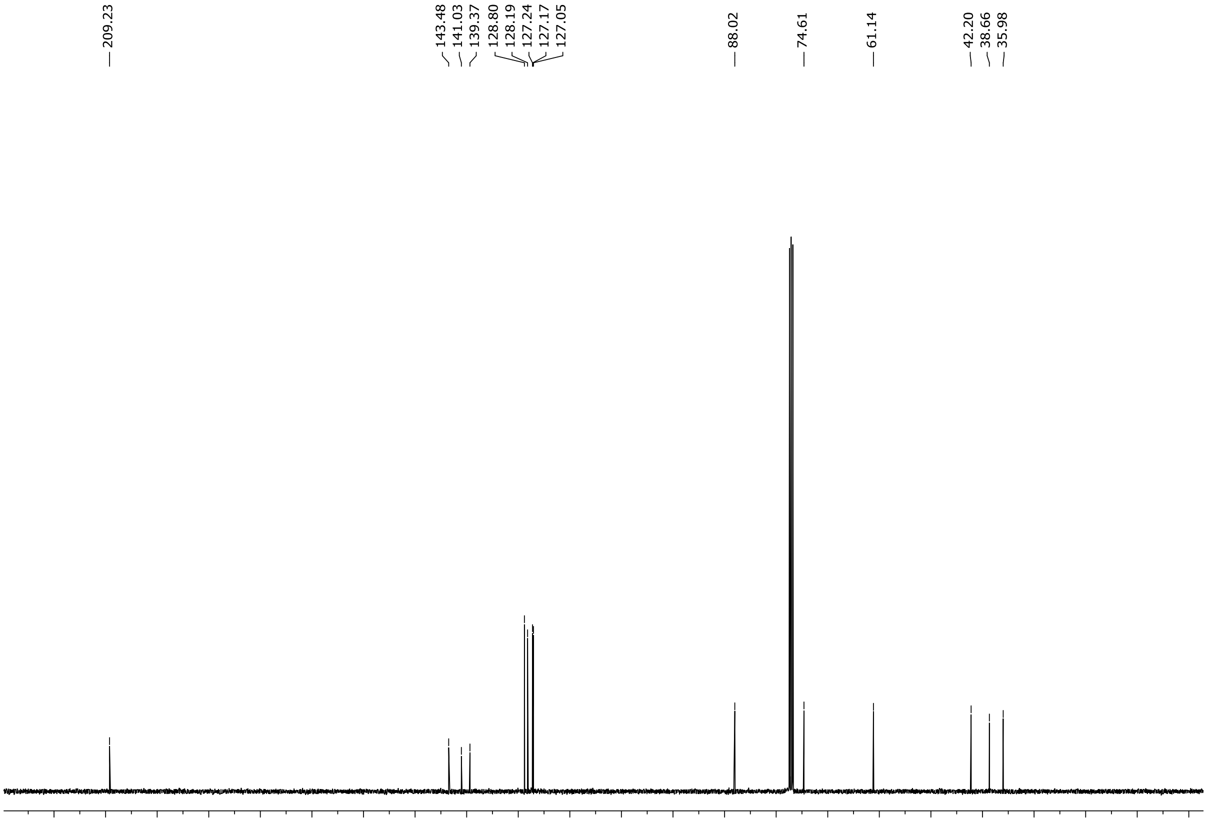

ethyl 3-(3-phenylpropyl)hepta-5,6-dienoate 69

^{1}H-NMR

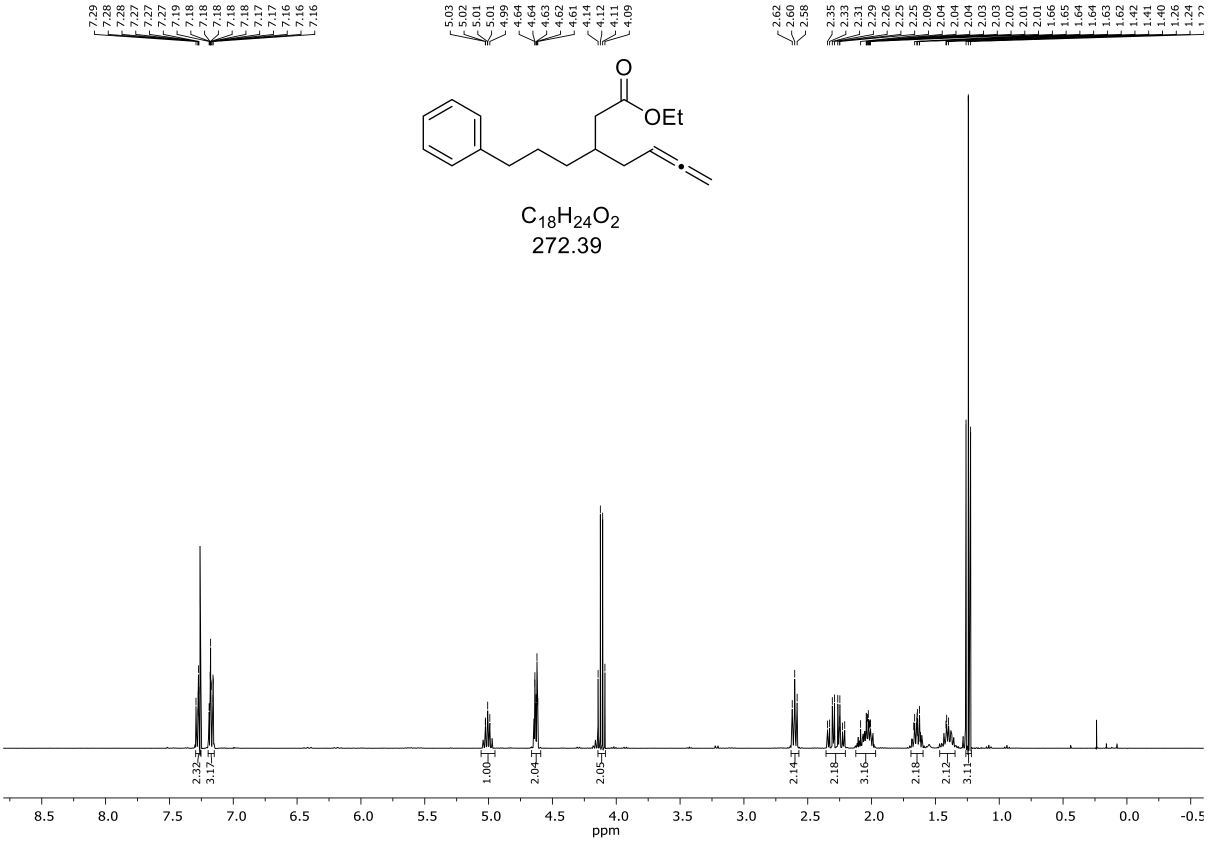

$C_{18}H_{24}O_2$
272.39

^{13}C-NMR

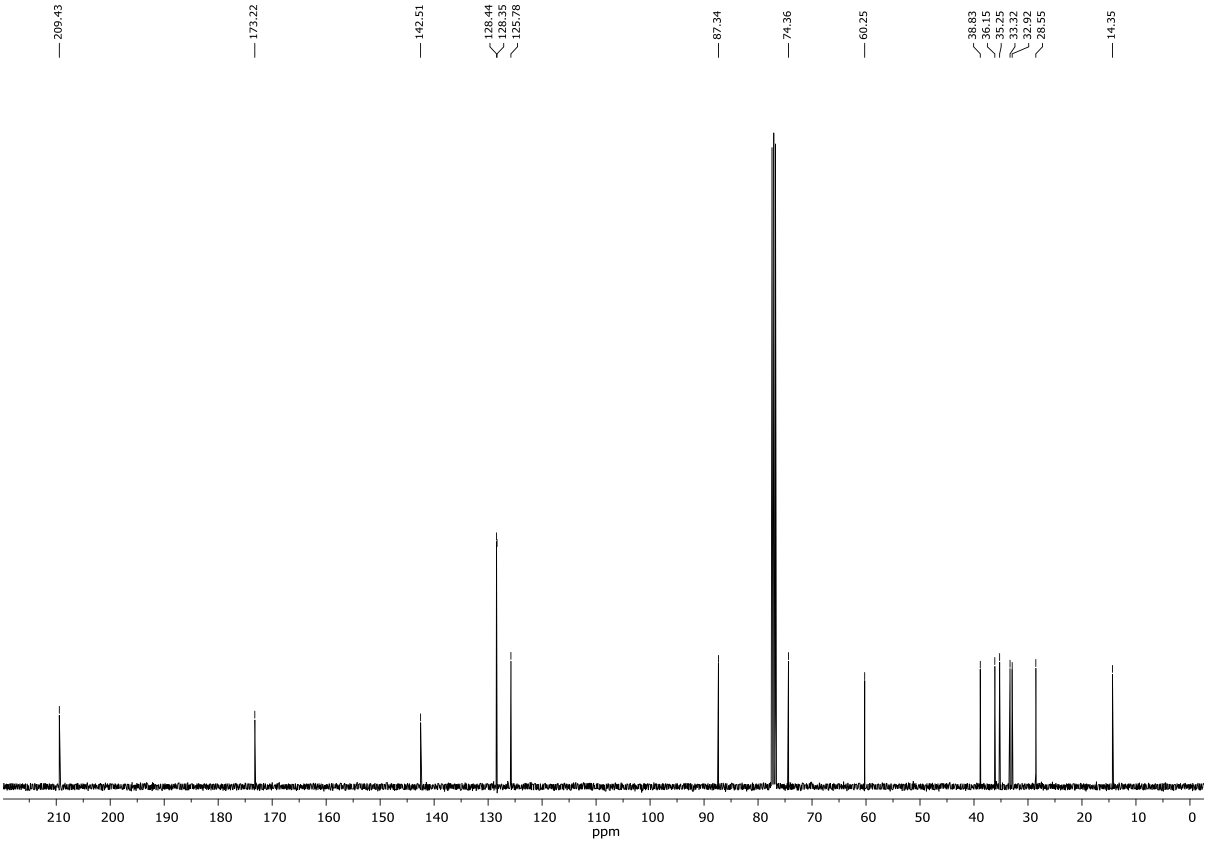

3-(3-phenylpropyl)hepta-5,6-dienol 70

^{1}H-NMR

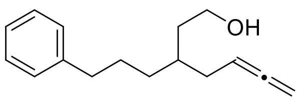

$C_{16}H_{22}O$
230.35

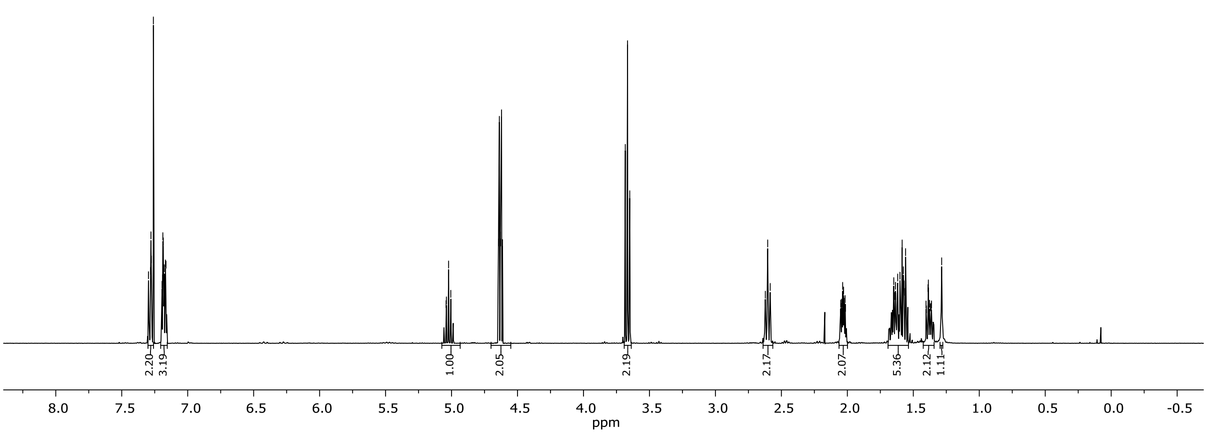

^{13}C-NMR

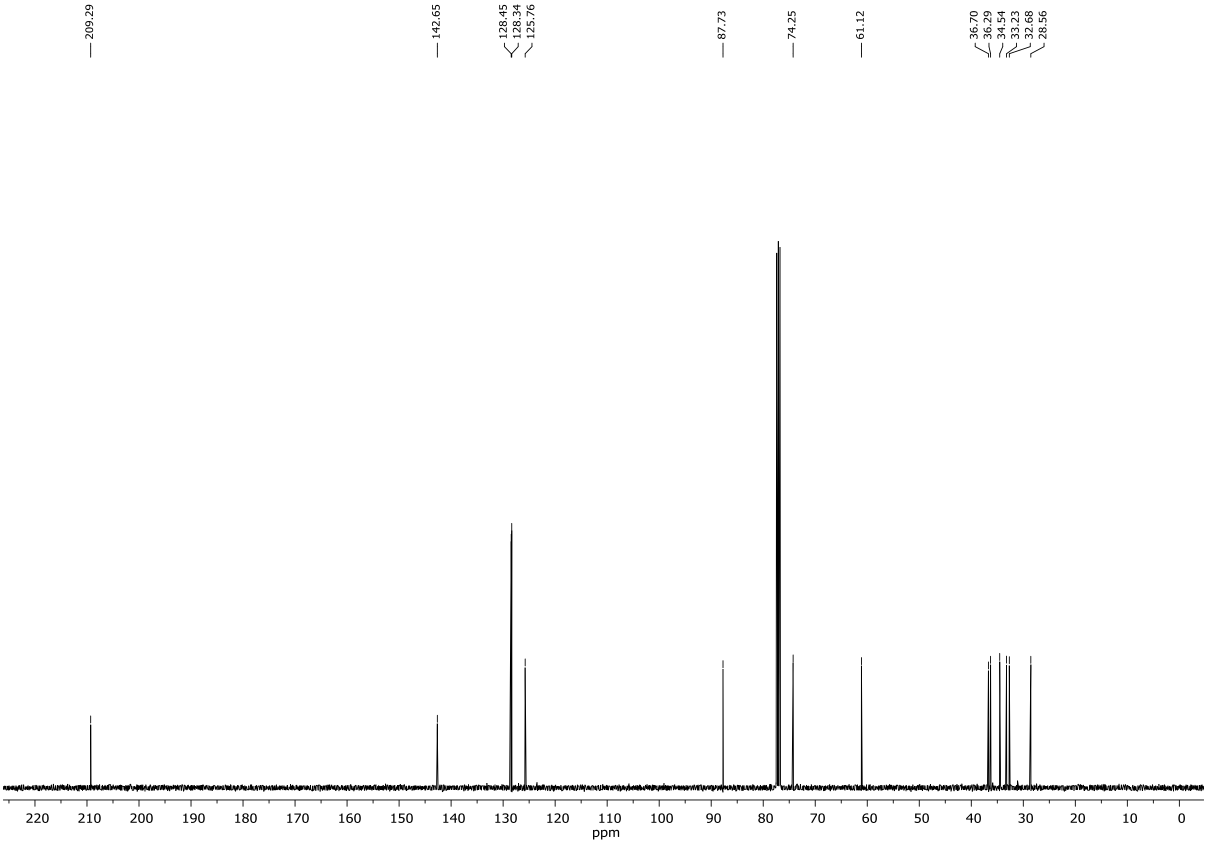

ethyl 3-phenylhepta-5,6-dienoate 71

^{1}H-NMR

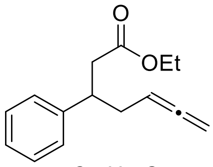

$C_{15}H_{18}O_2$
230.31

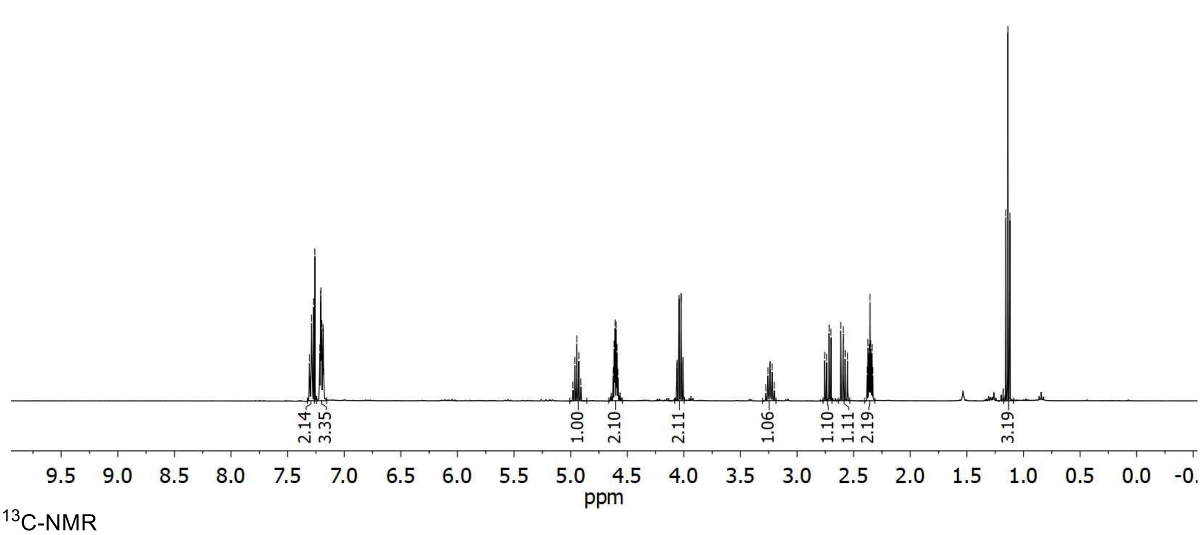

^{13}C-NMR

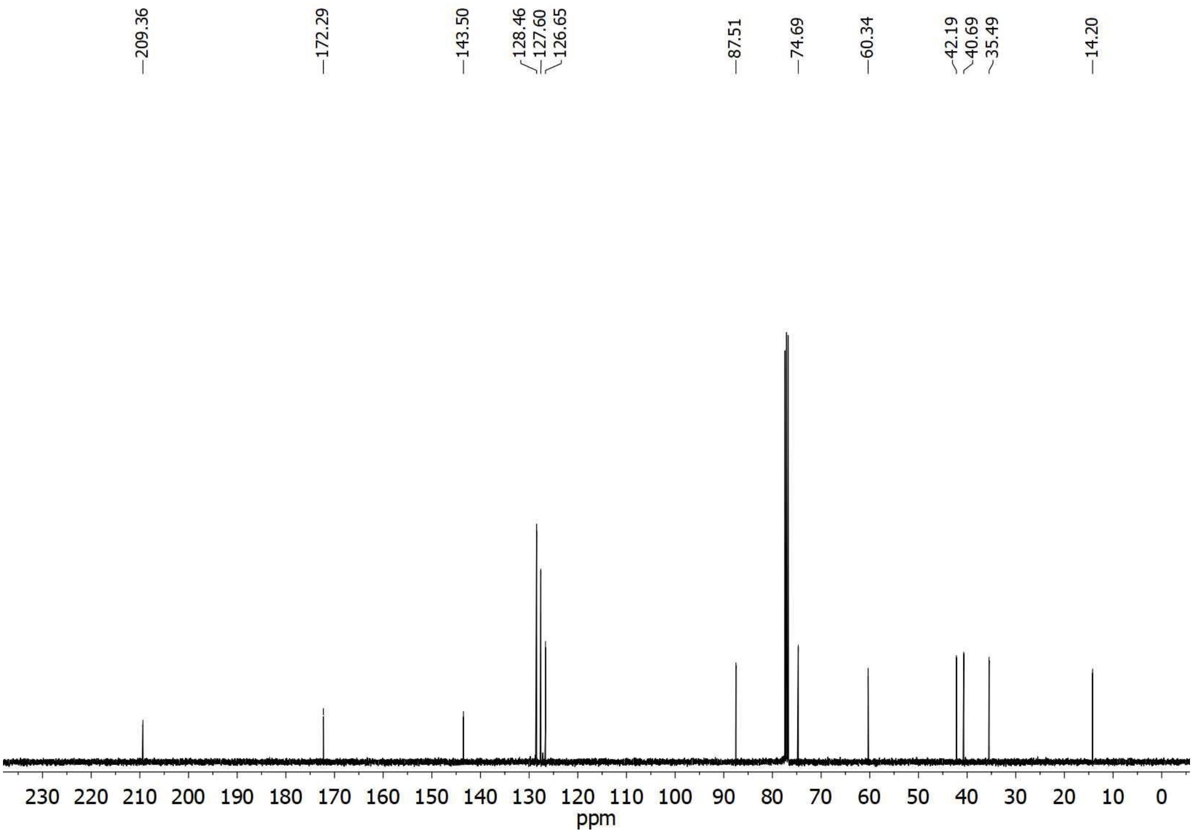

3-phenylhepta-5,6-dien-1-ol 72

^{1}H-NMR

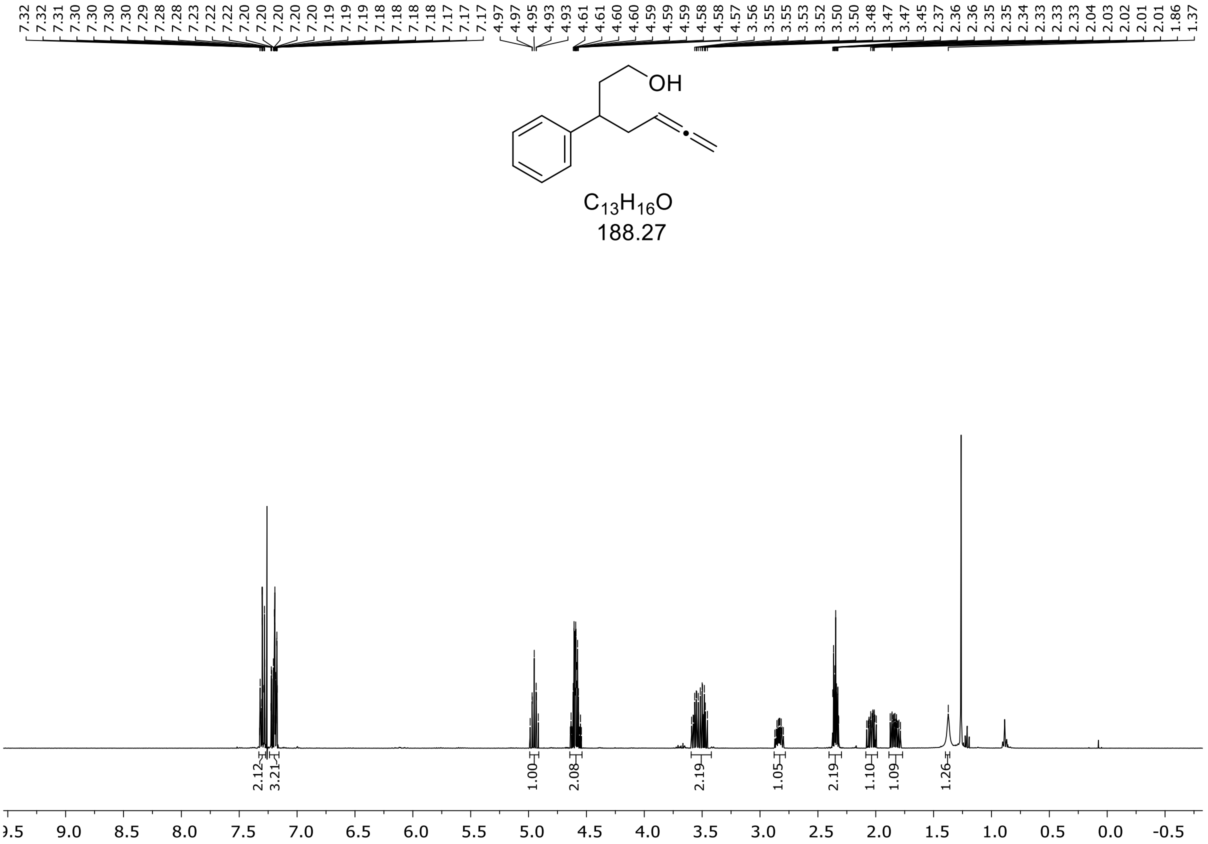

^{13}C-NMR

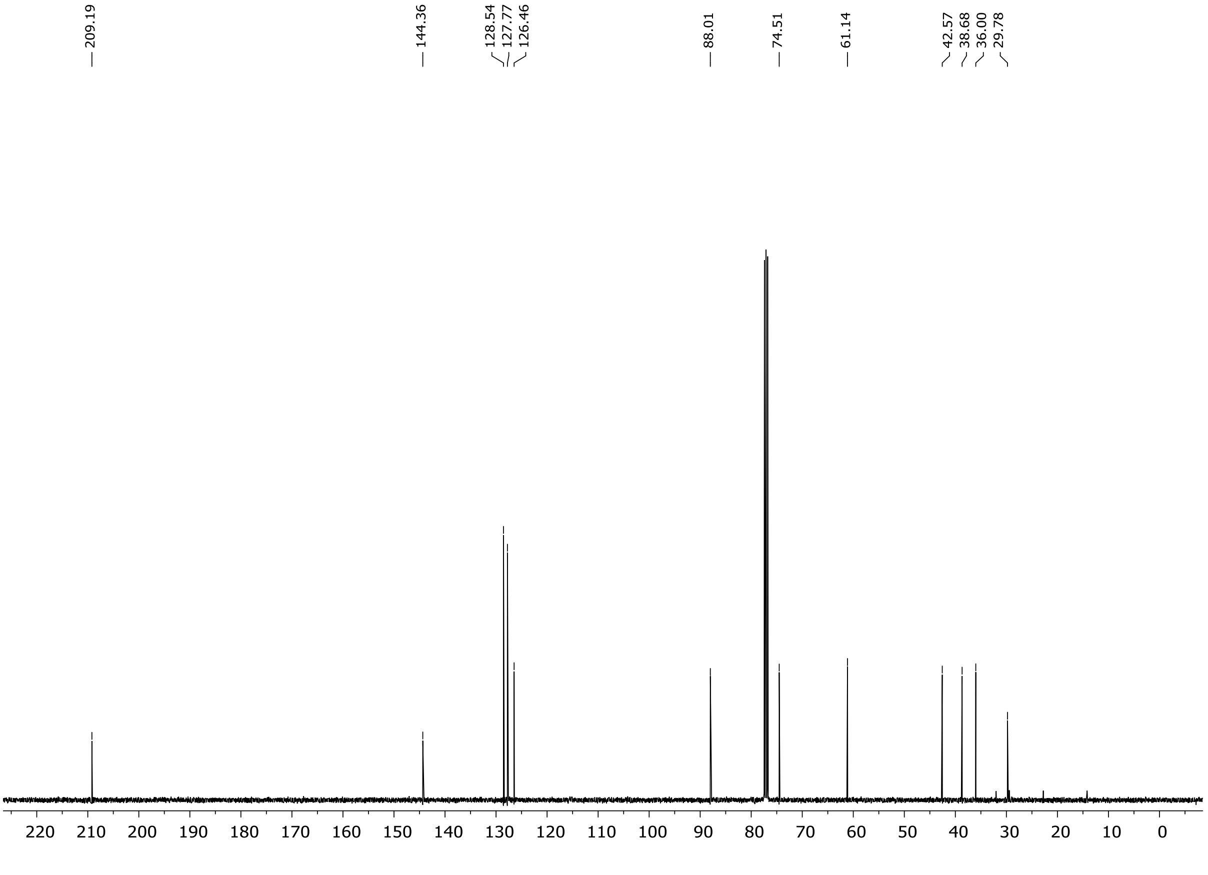

ethyl 3-(naphthalene-2-yl)hepta-5,6-dienoate 73

^{1}H-NMR

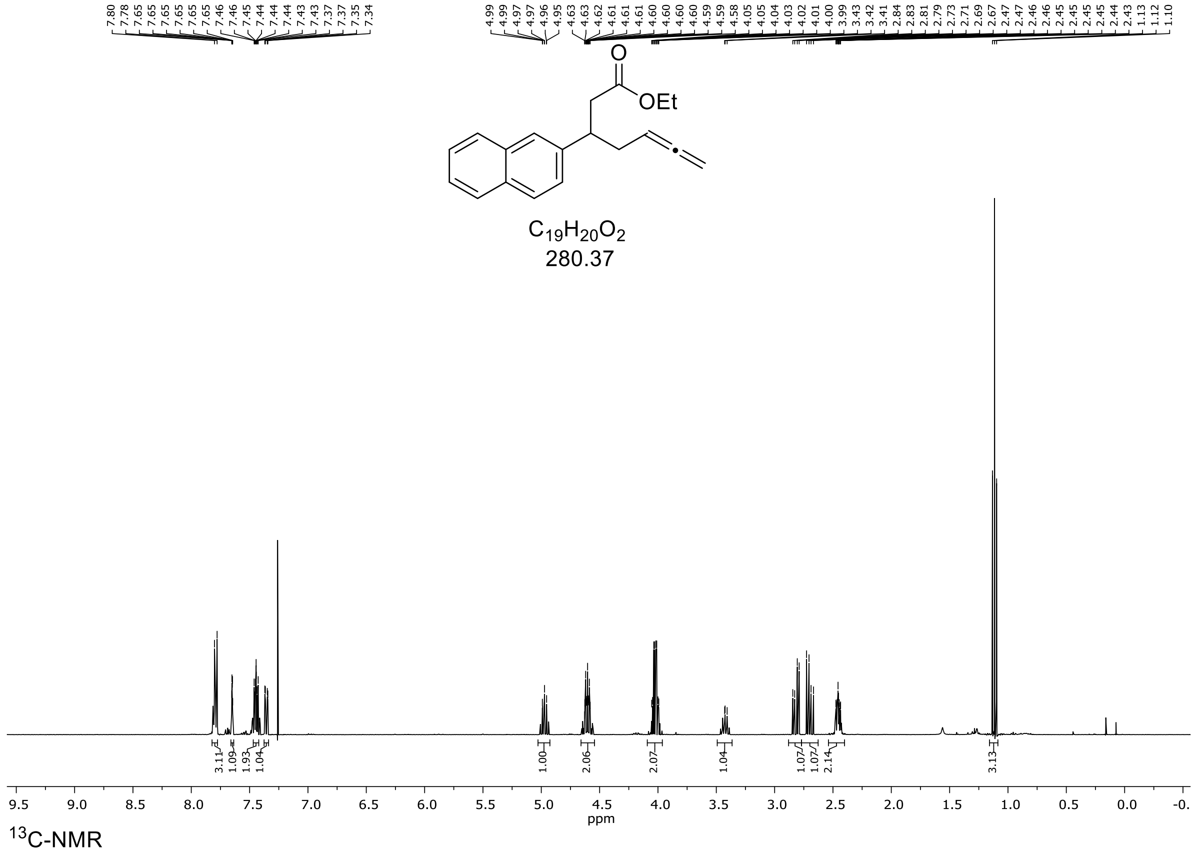

^{13}C-NMR

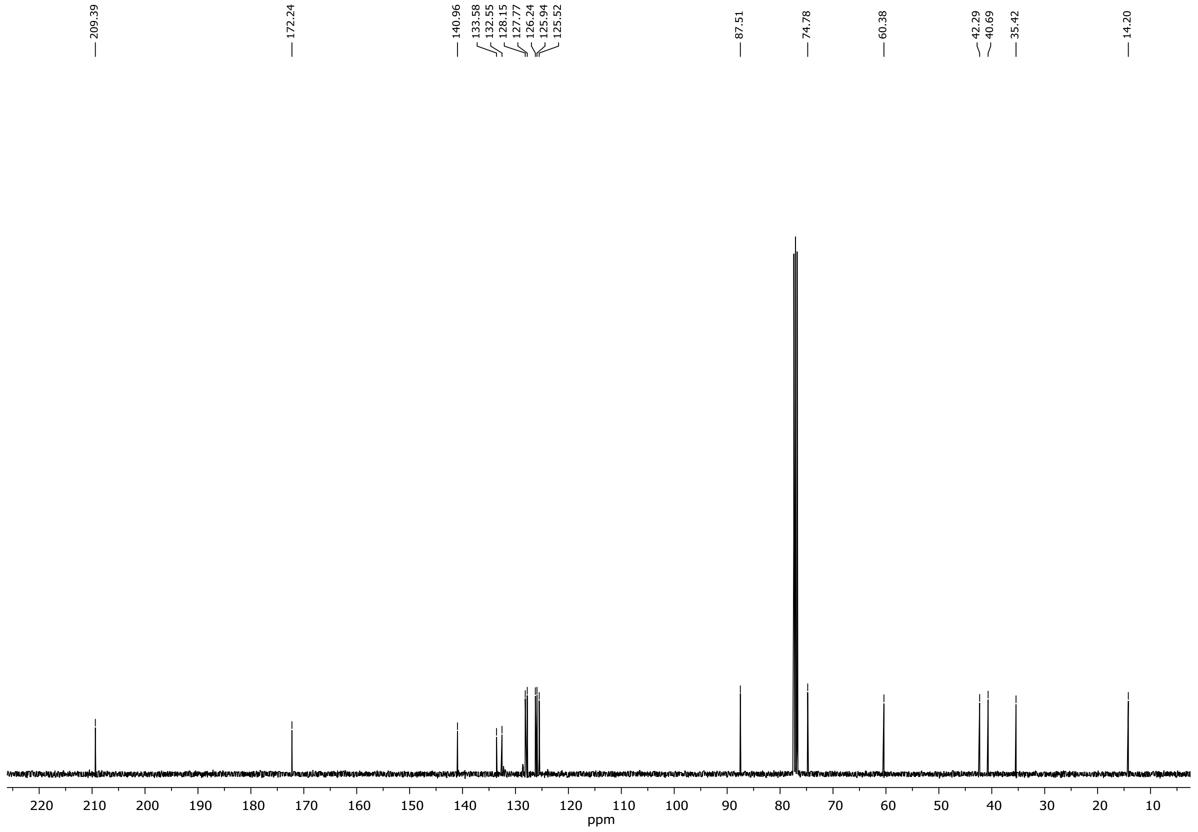

ethyl 3-([1,1'-biphenyl]-4-yl)hepta-5,6-dienoate 75

^{1}H-NMR

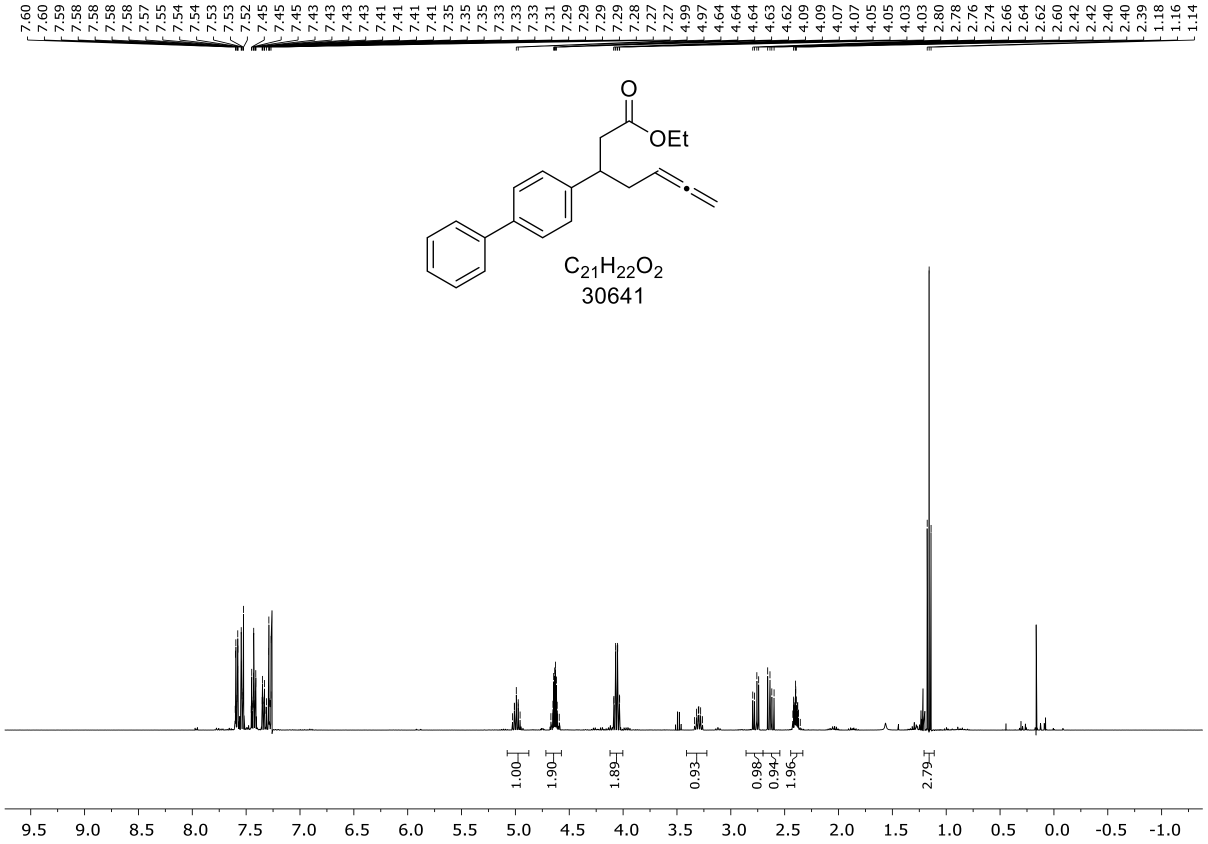

^{13}C-NMR

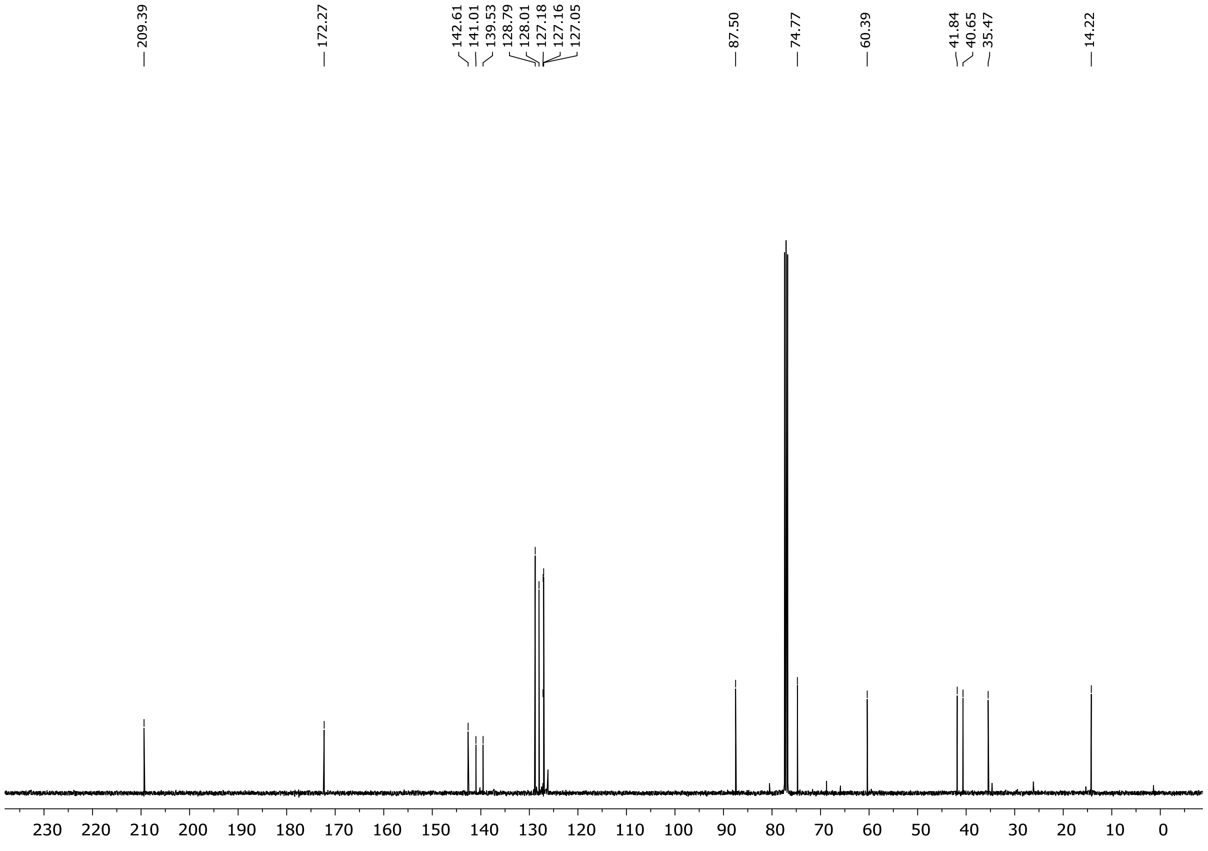

3-([1,1'-biphenyl]-4-yl)hepta-5,6-dien-1-ol 76

^{1}H-NMR

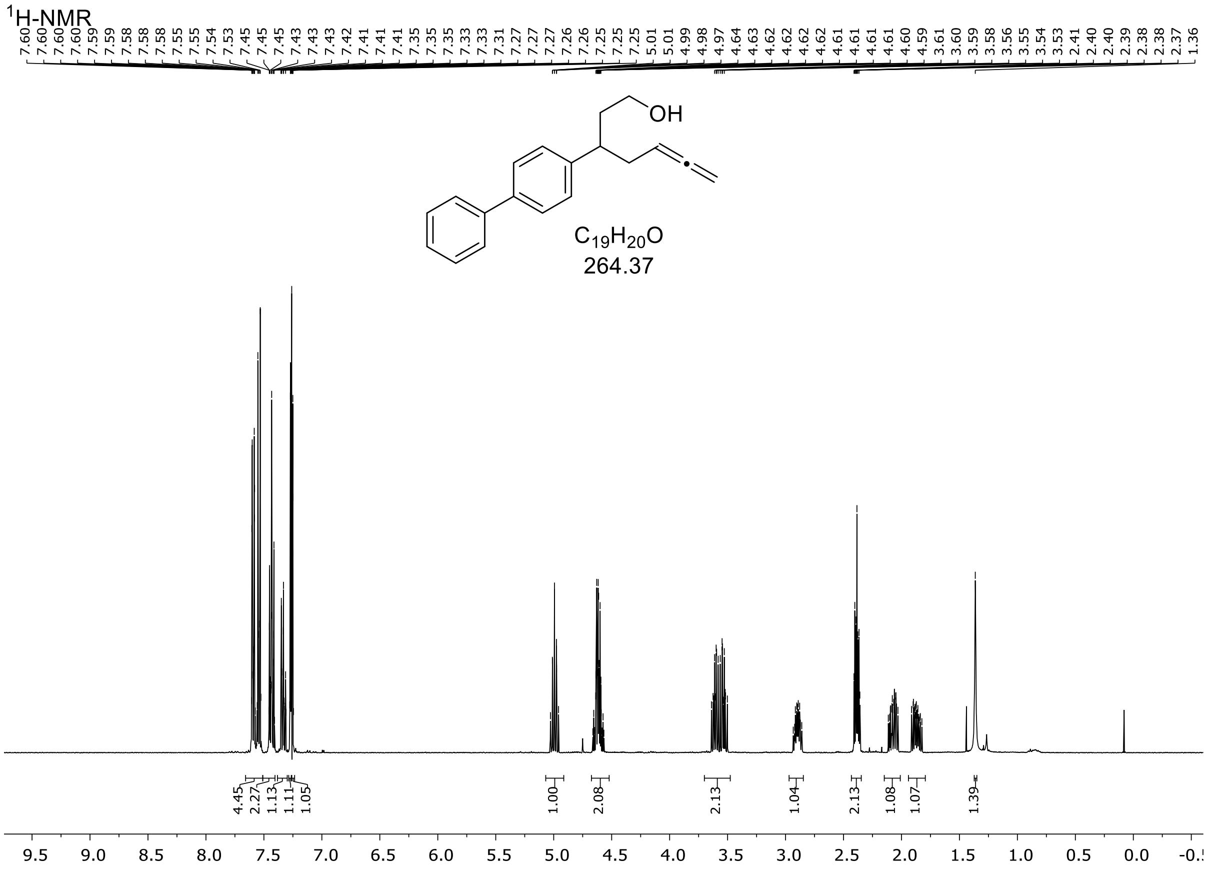

^{13}C-NMR

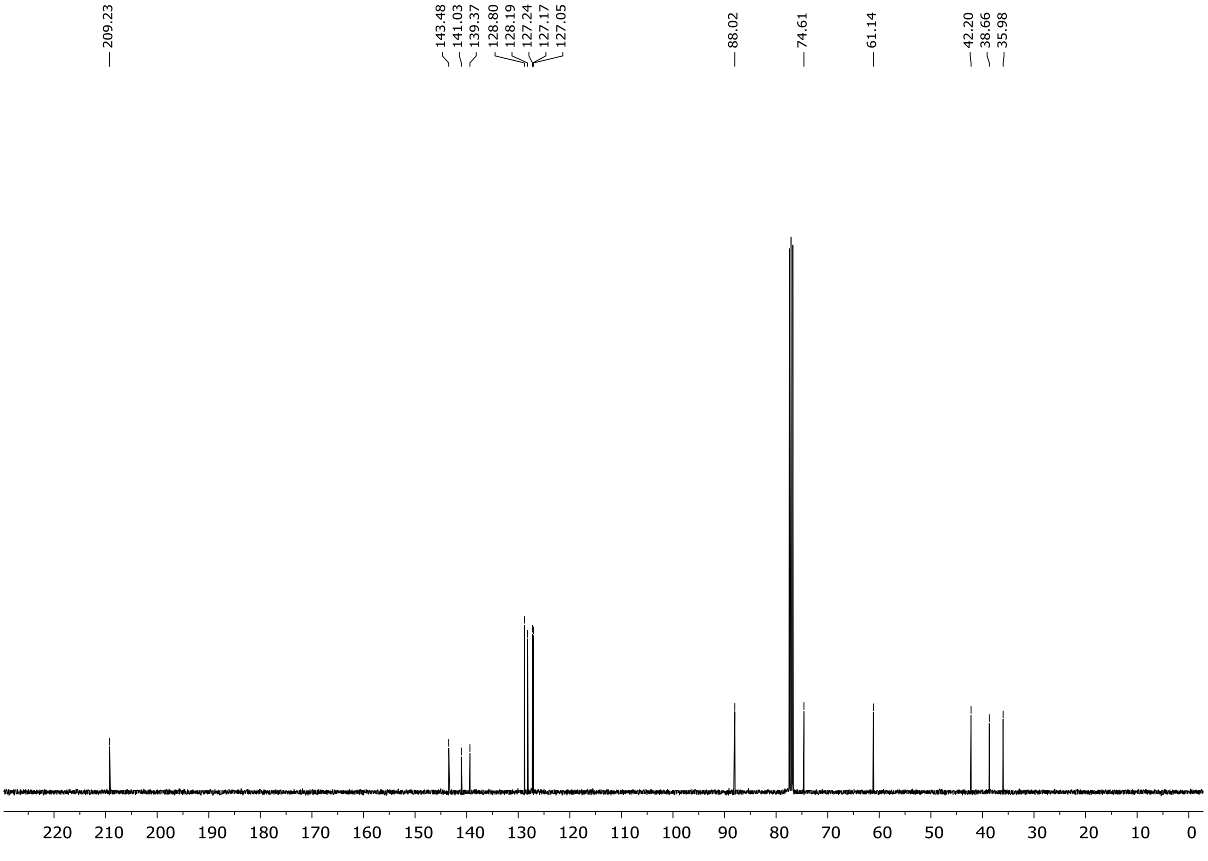

ethyl 3-(*p*-tolyl)hepta-5,6-dienoate 77

^{1}H-NMR

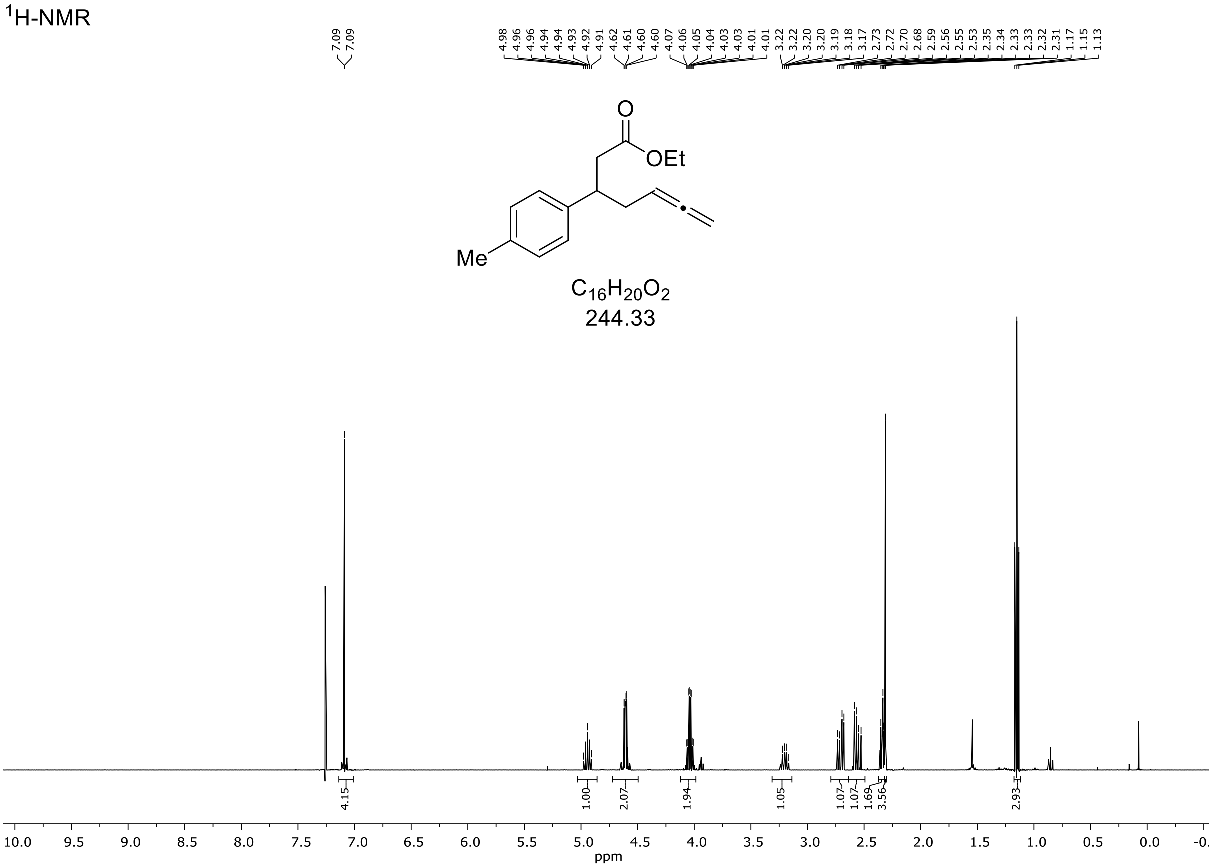

^{13}C-NMR

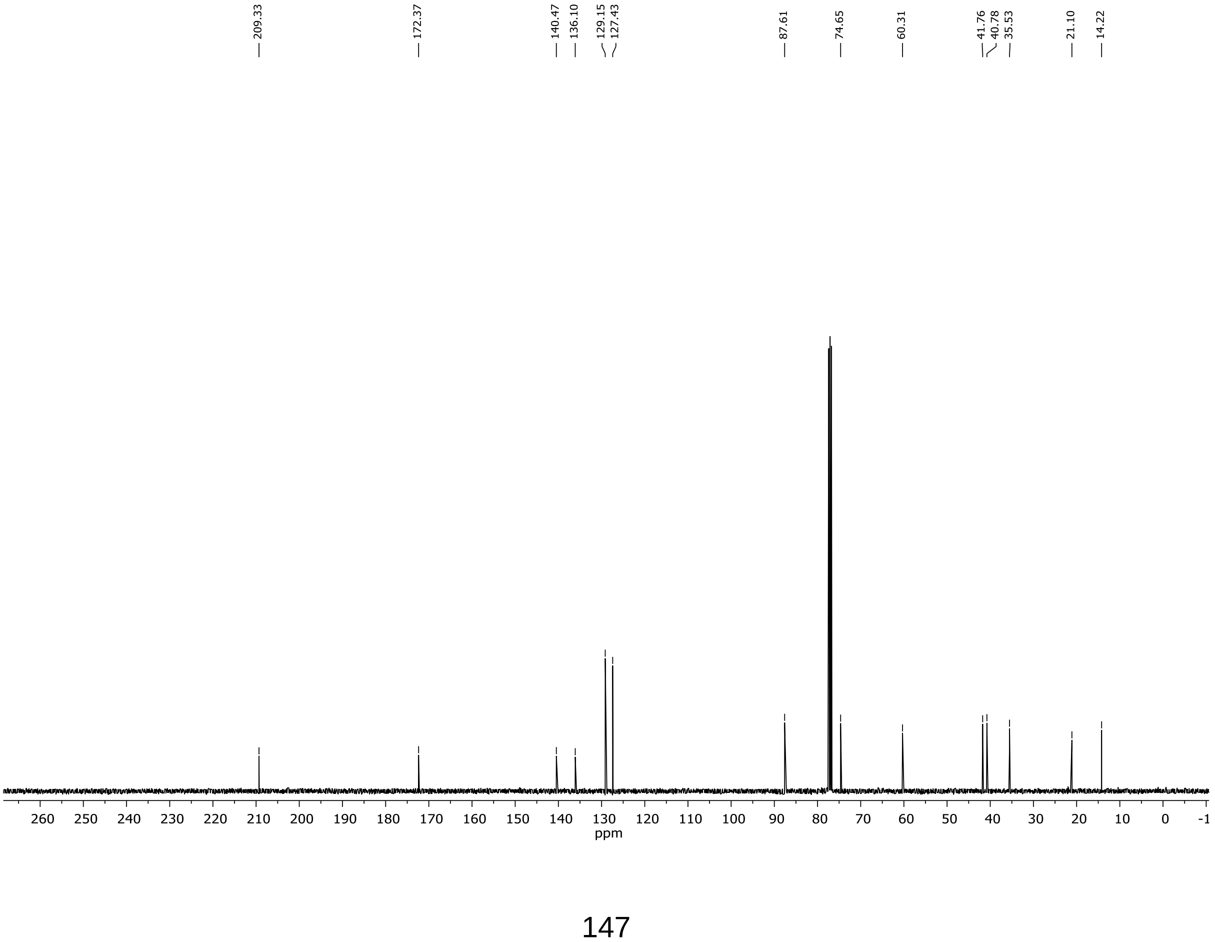

3-(*p*-tolyl)hepta-5,6-dien-1-ol 78

^{1}H-NMR

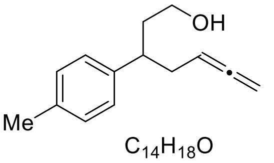

$C_{14}H_{18}O$
202.30

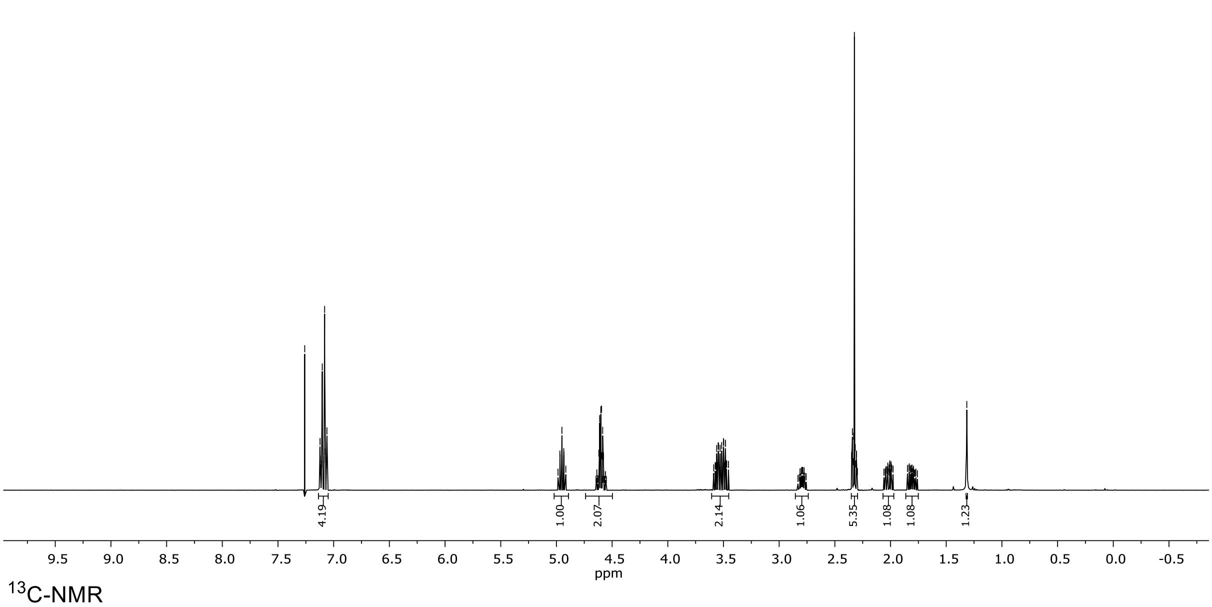

^{13}C-NMR

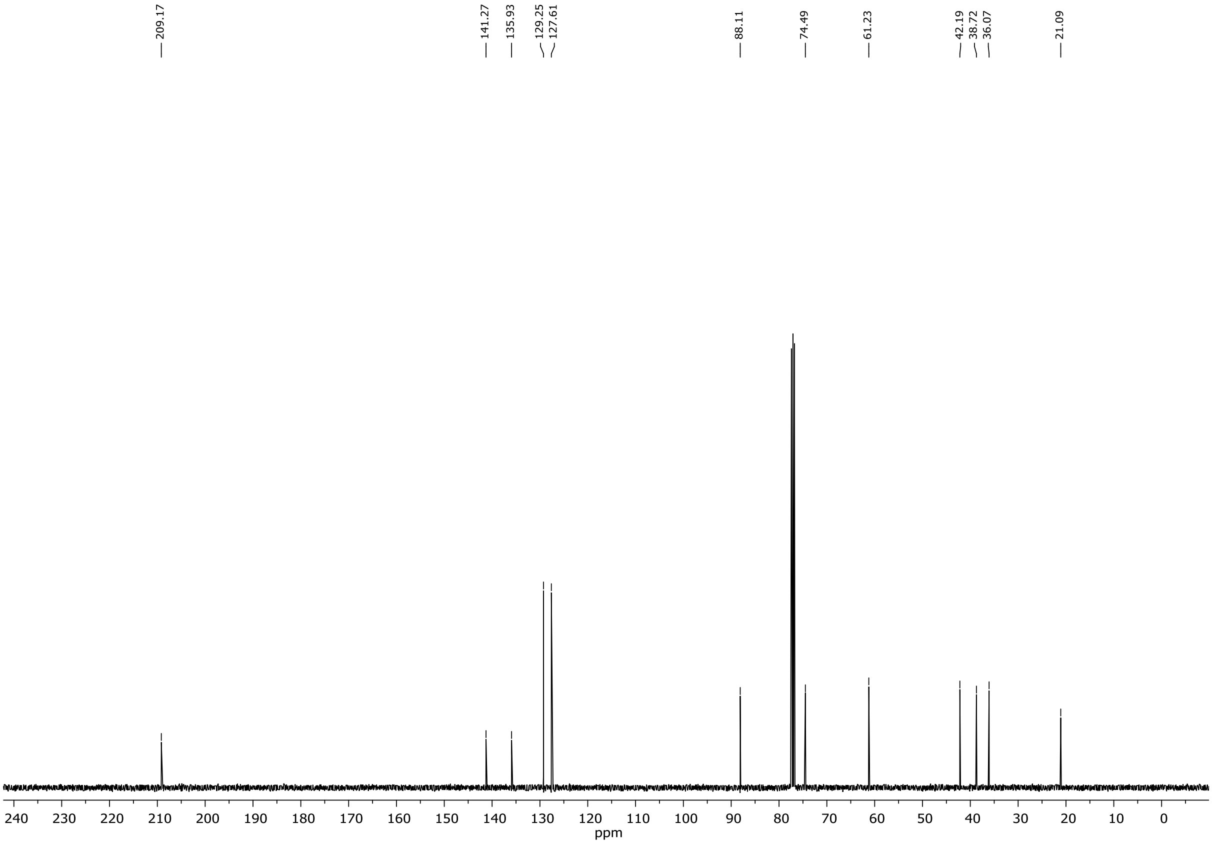

ethyl 3-(*m*-tolyl)hepta-5,6-dienoate 79

^{1}H-NMR

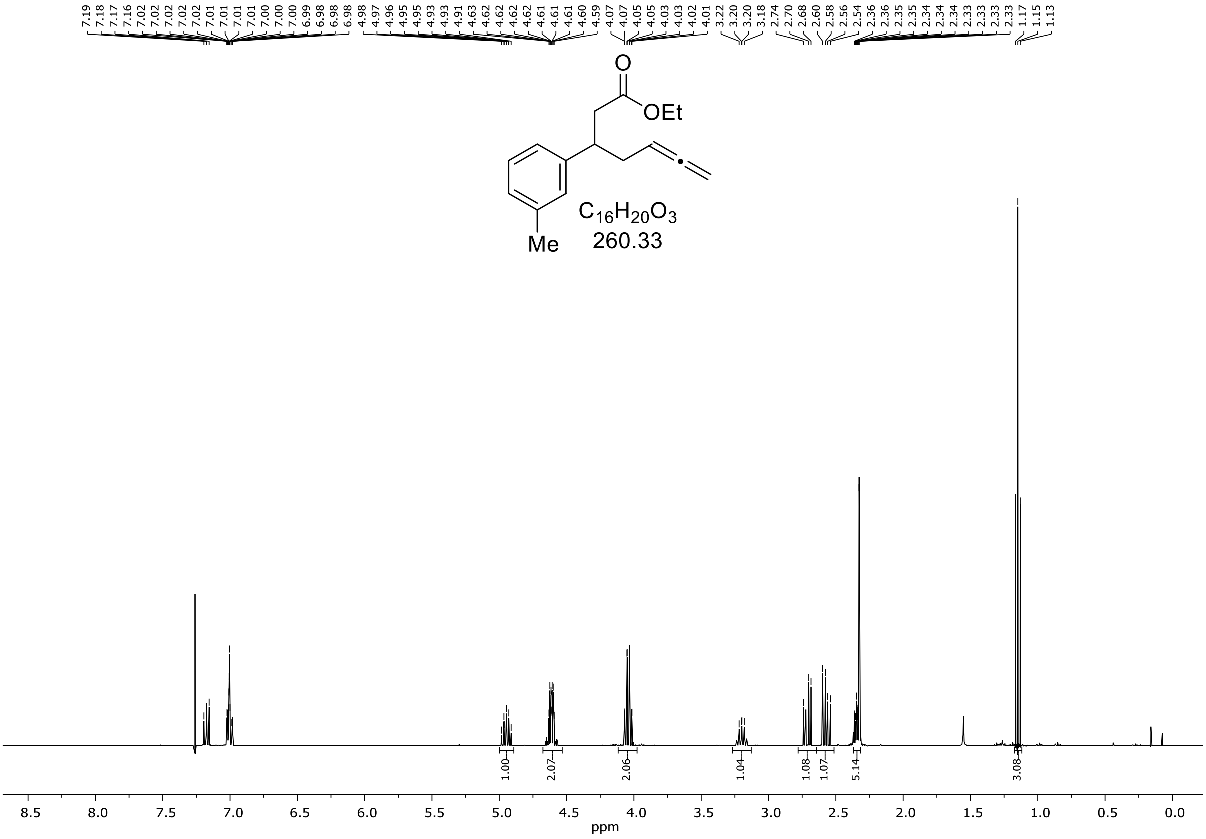

^{13}C-NMR

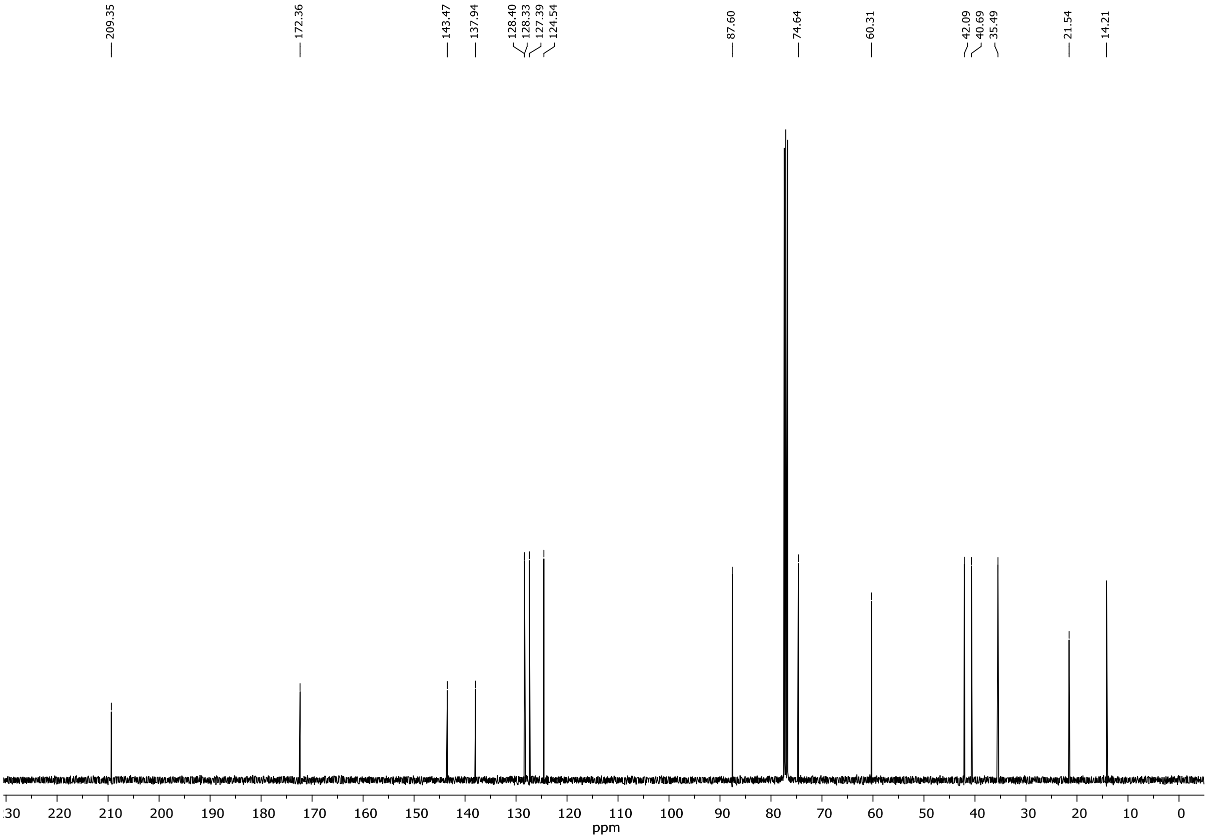

3-(*m*-tolyl)hepta-5,6-dien-1-ol 80

^{1}H-NMR

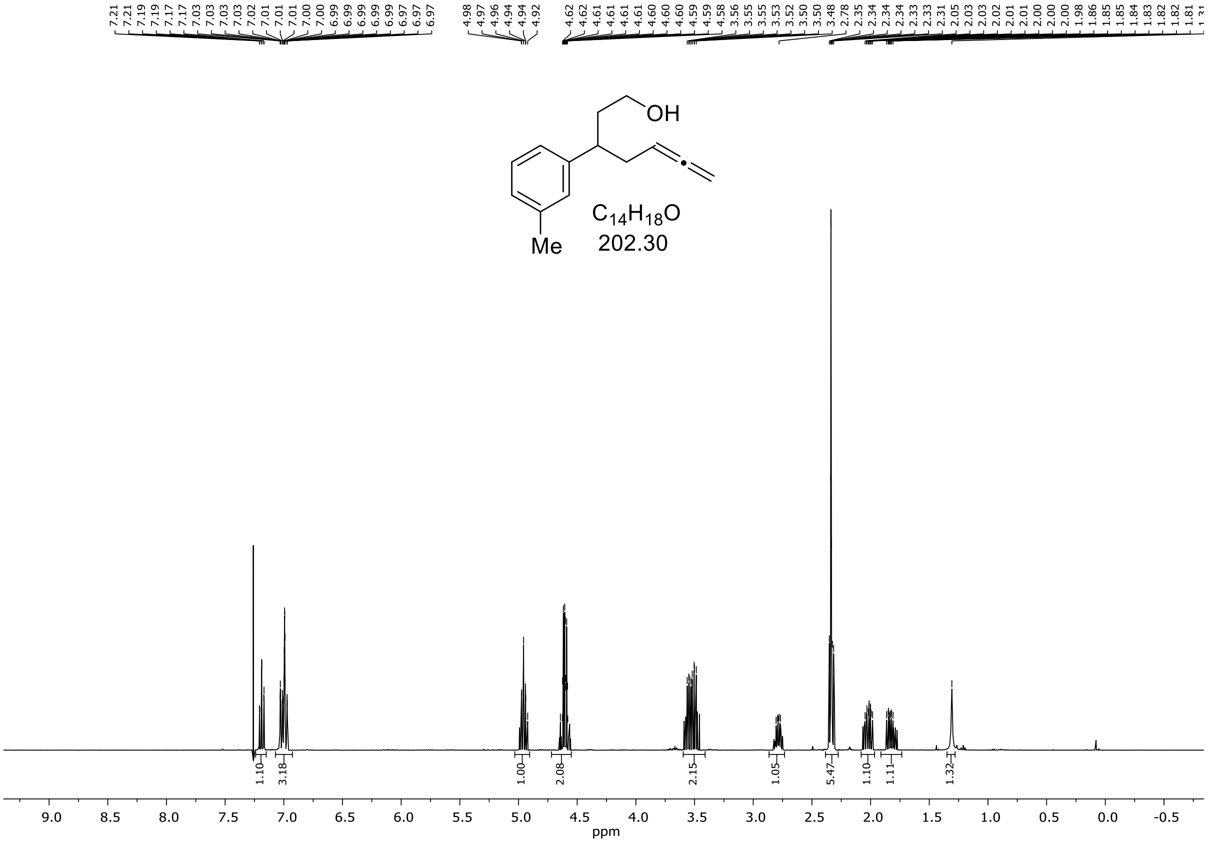

^{13}C-NMR

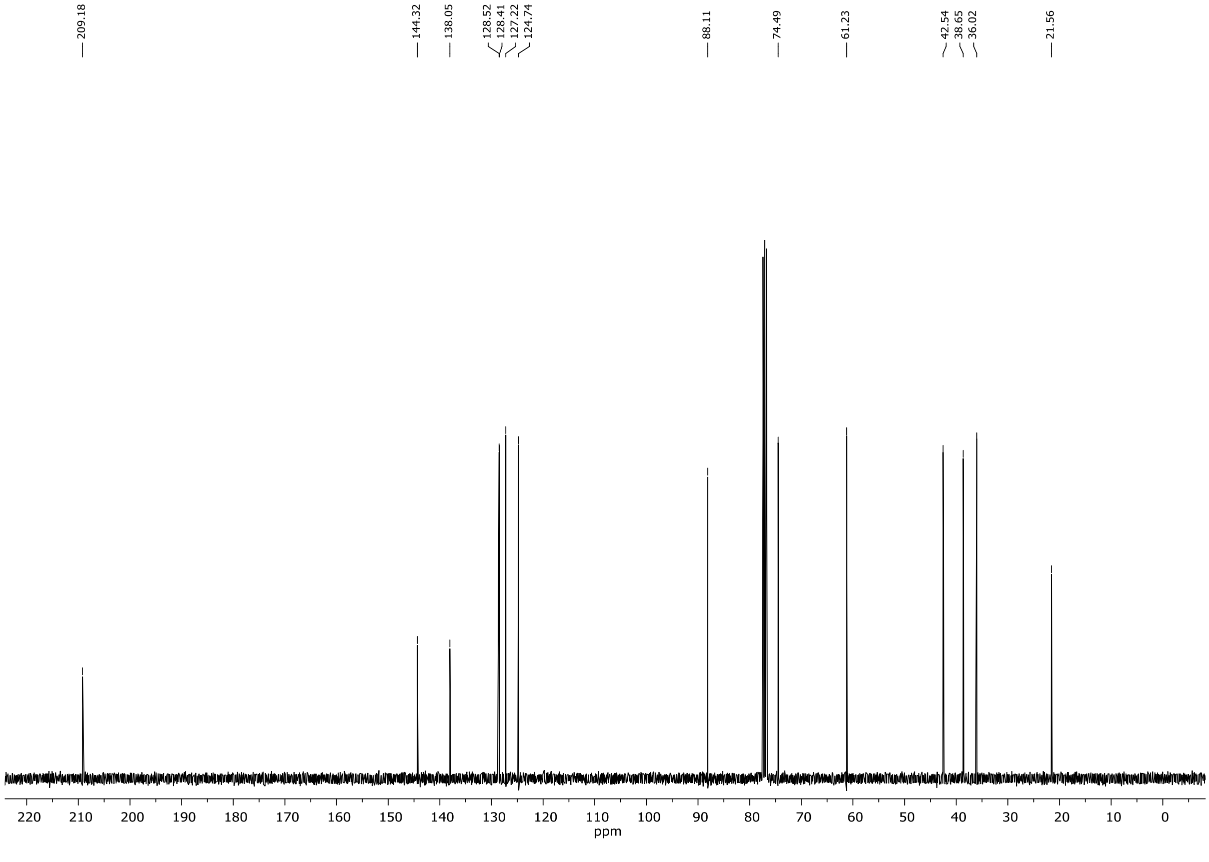

3-(*o*-tolyl)hepta-5,6-dienoate 81

^{1}H-NMR

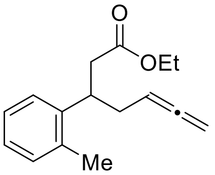

$C_{16}H_{20}O_2$
244.33

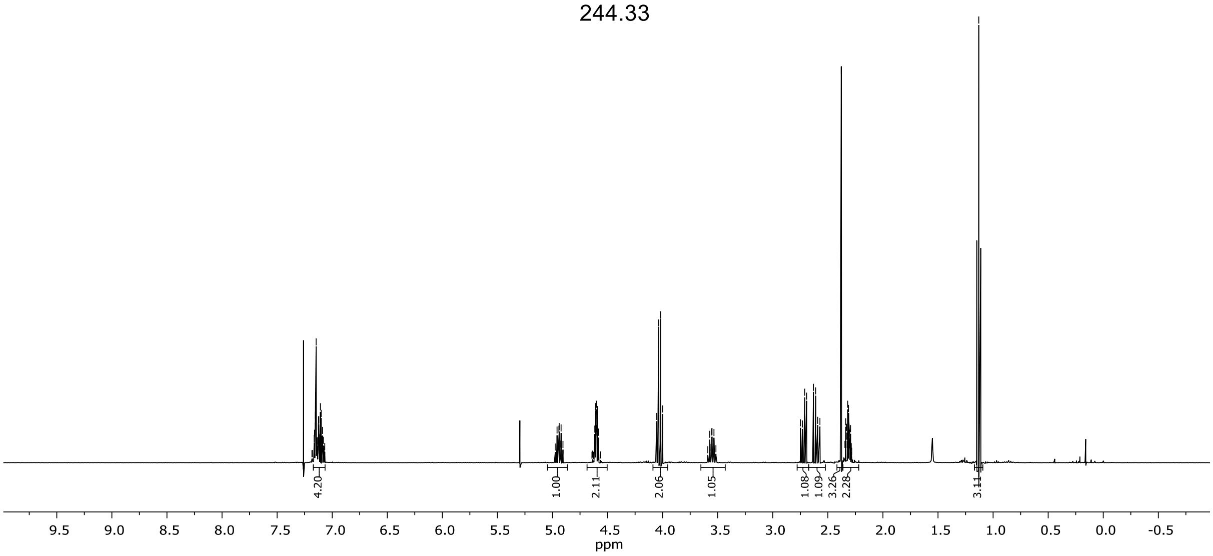

^{13}C-NMR

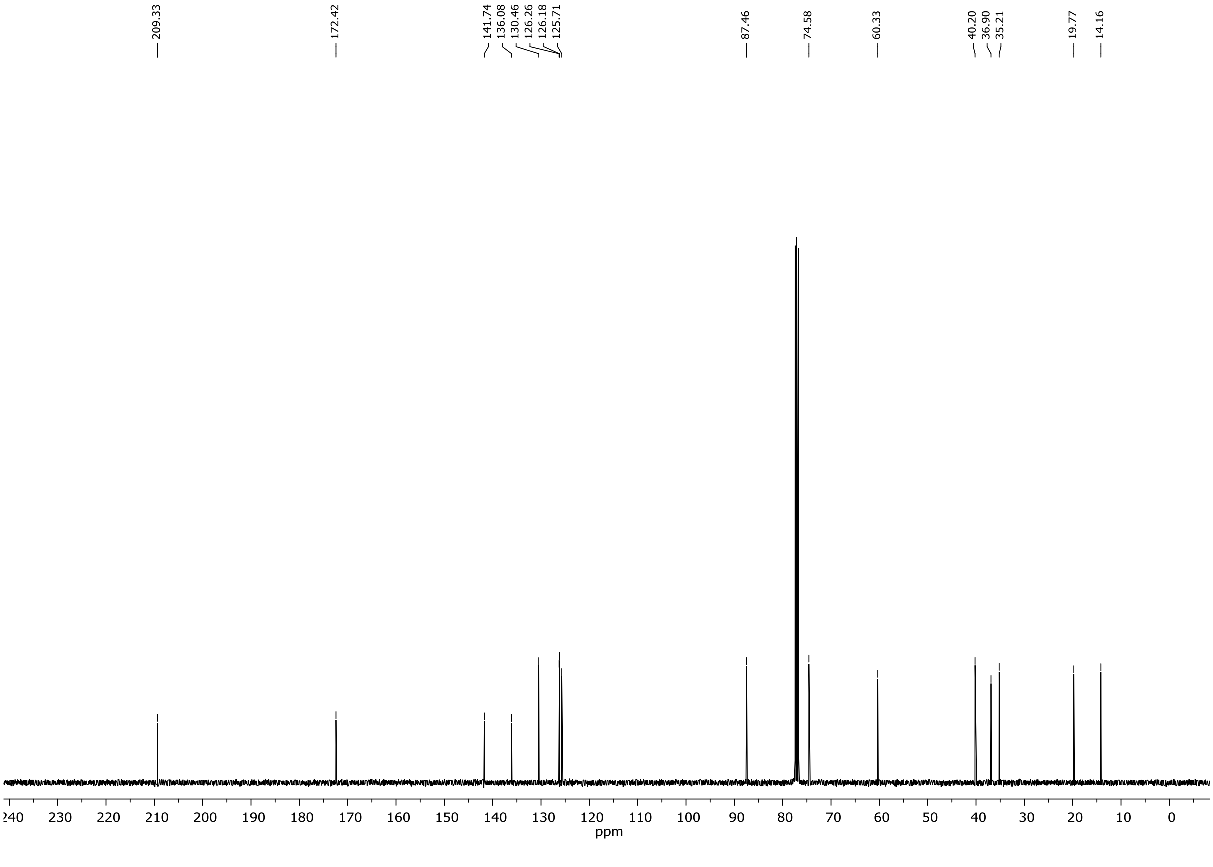

3-(*o*-tolyl)hepta-5,6-dien-1-ol 82

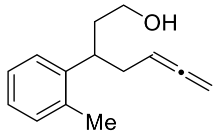

$C_{14}H_{18}O$

202.29

^{1}H-NMR

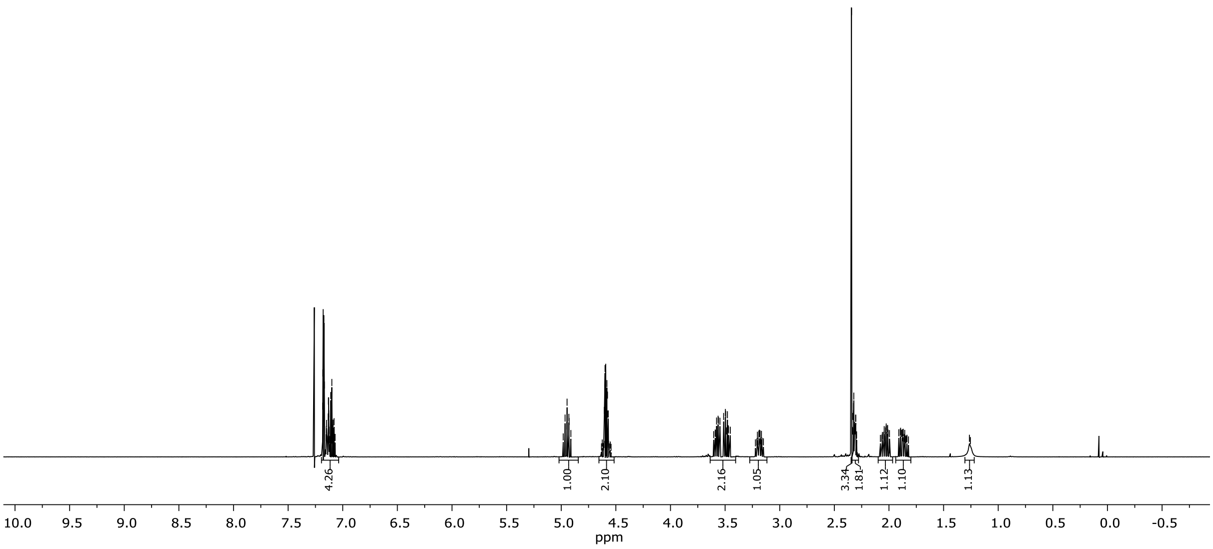

^{13}C-NMR

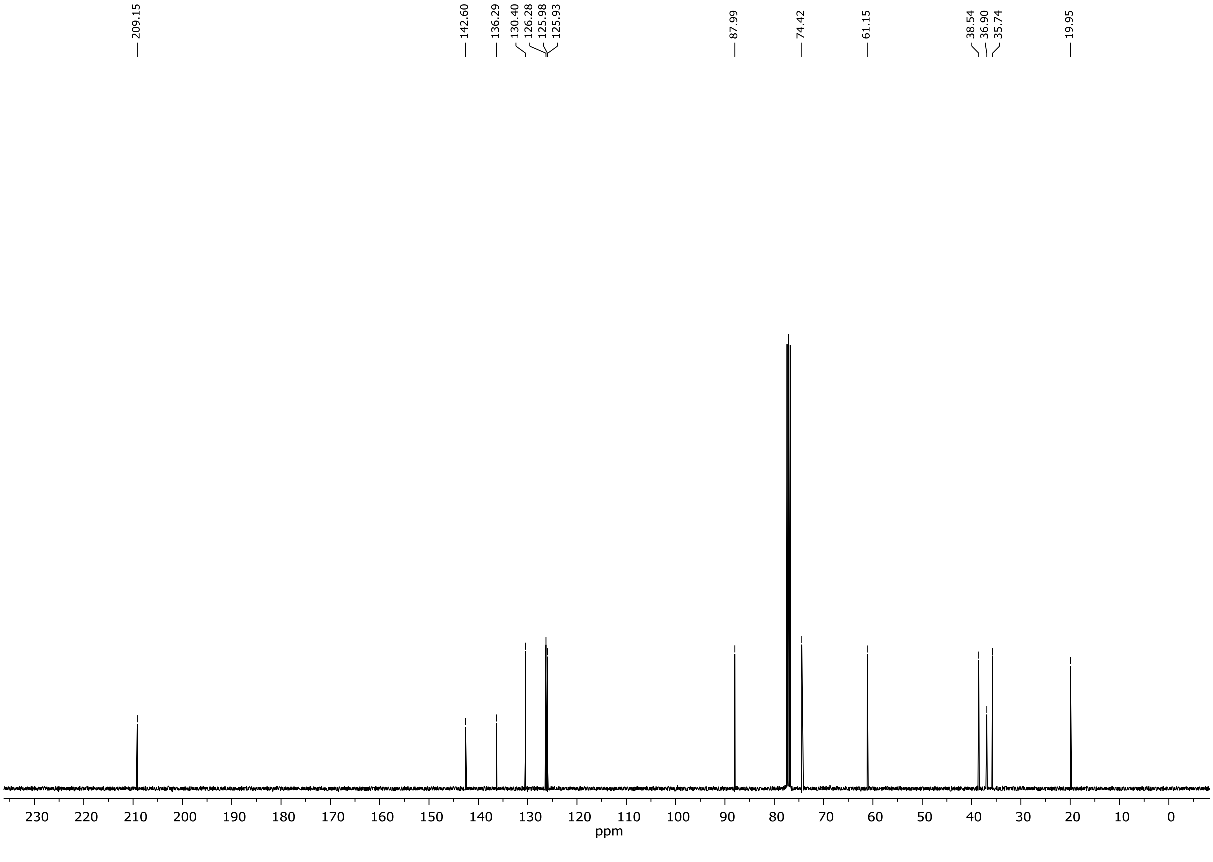

ethyl 3-mesitylhepta-5,6-dienoate 83

^{1}H-NMR

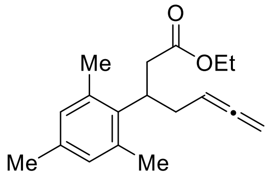

$C_{18}H_{24}O_2$
272.39

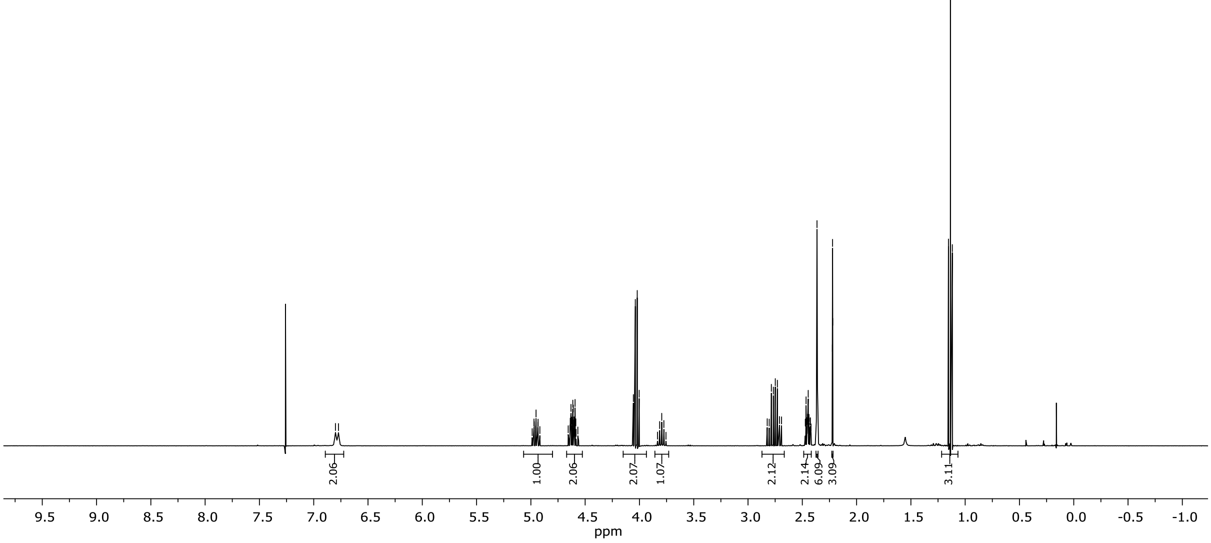

^{13}C-NMR

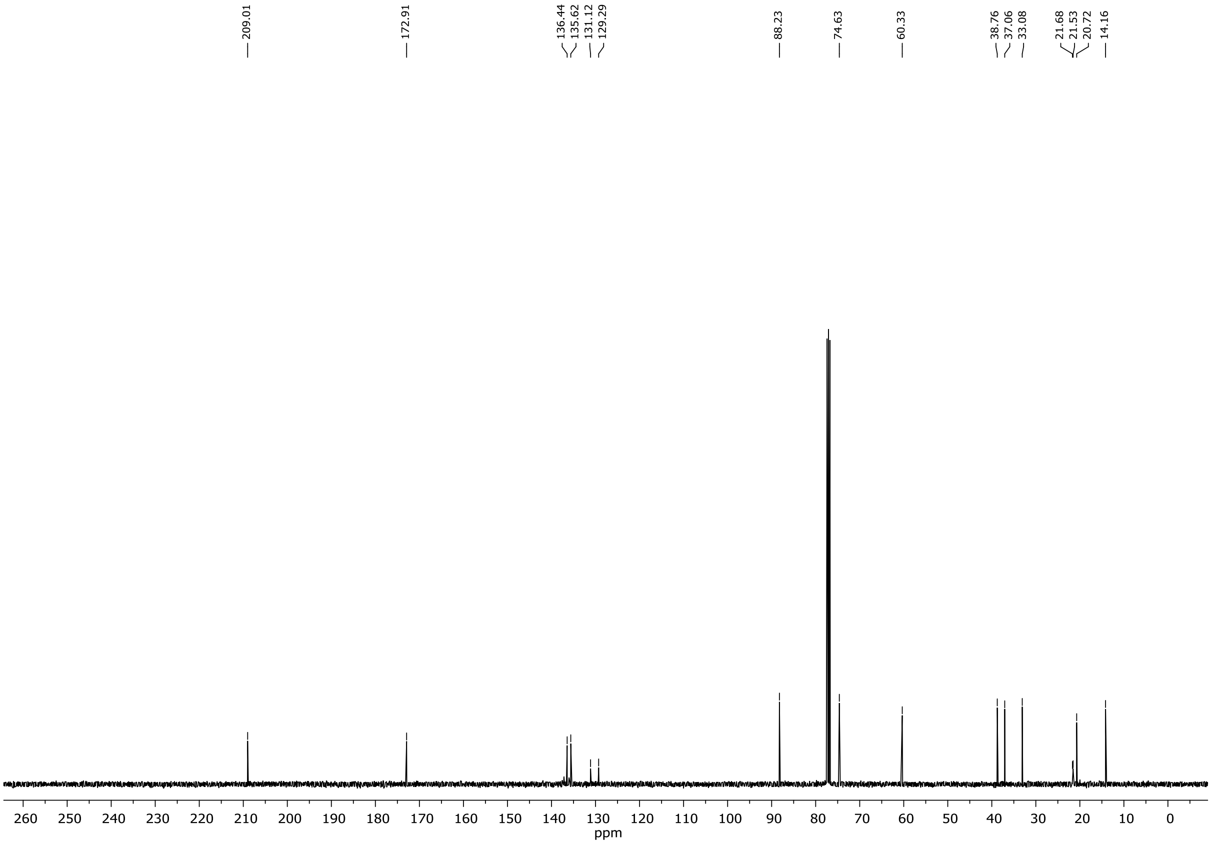

3-mesitylhepta-5,6-dien-1-ol 84

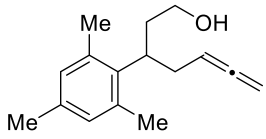

$C_{16}H_{22}O$

230.35

^{1}H-NMR

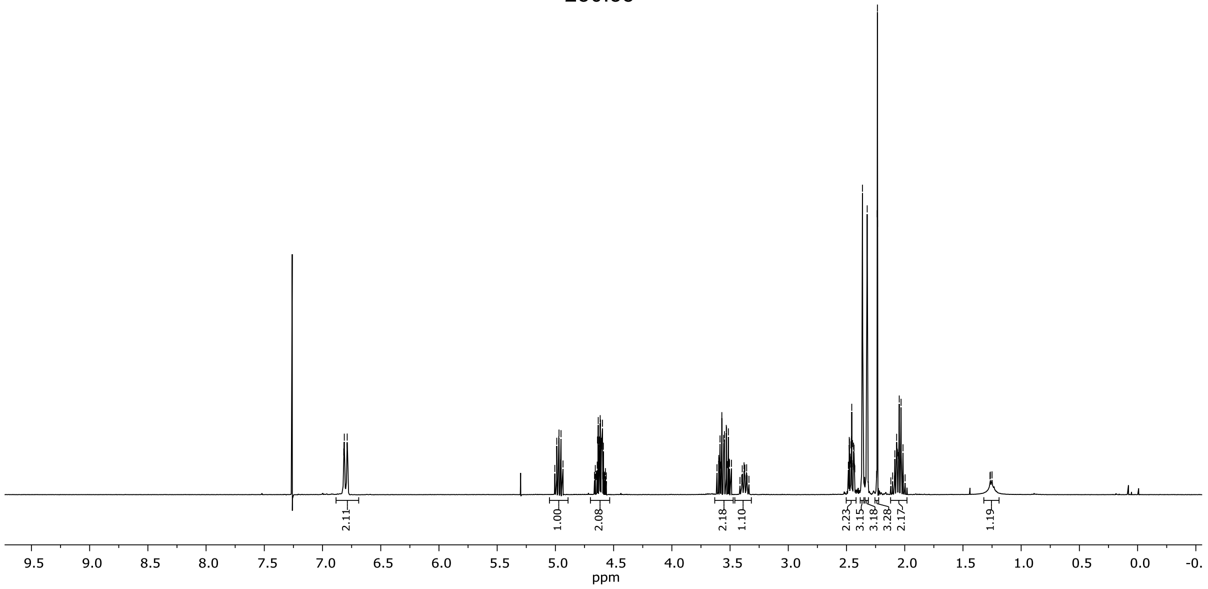

^{13}C-NMR

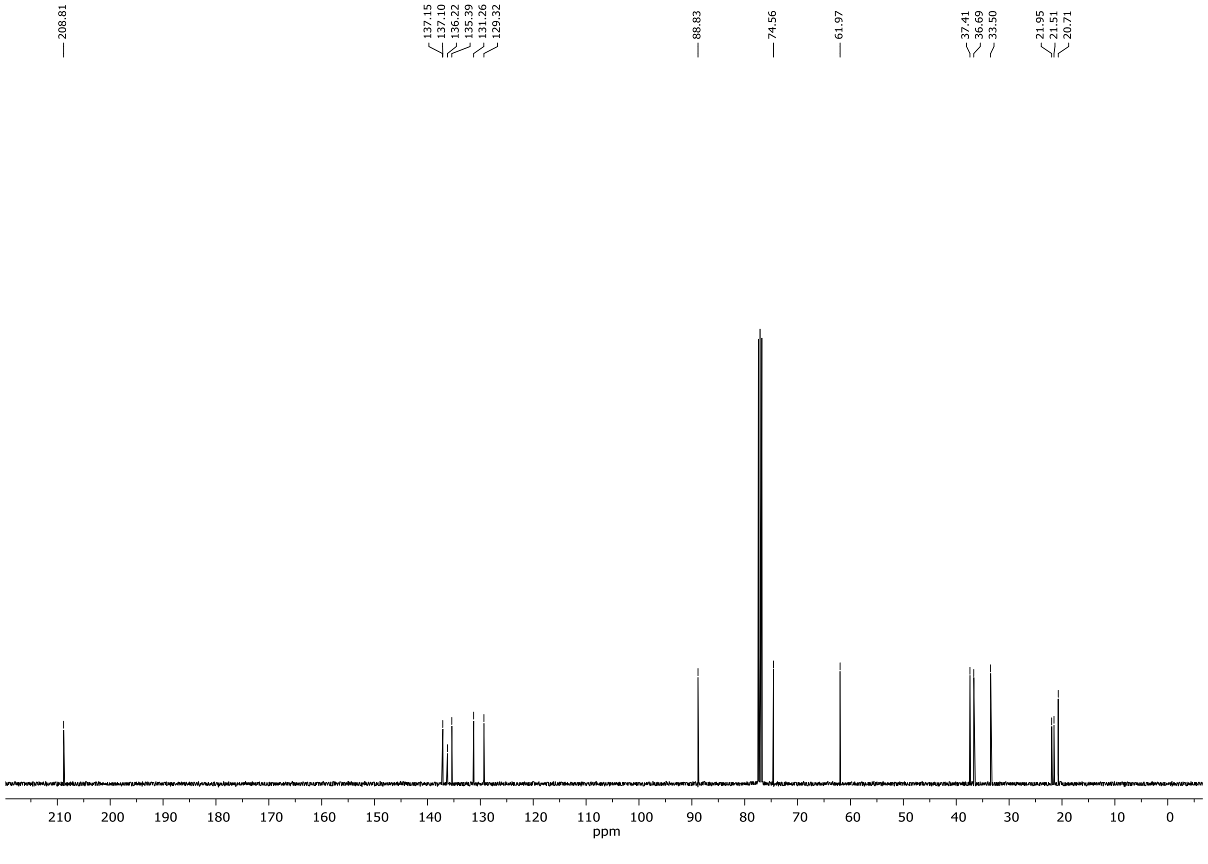

ethyl 3-(4-vinylphenyl)hepta-5,6-dienoate 85

^{1}H-NMR

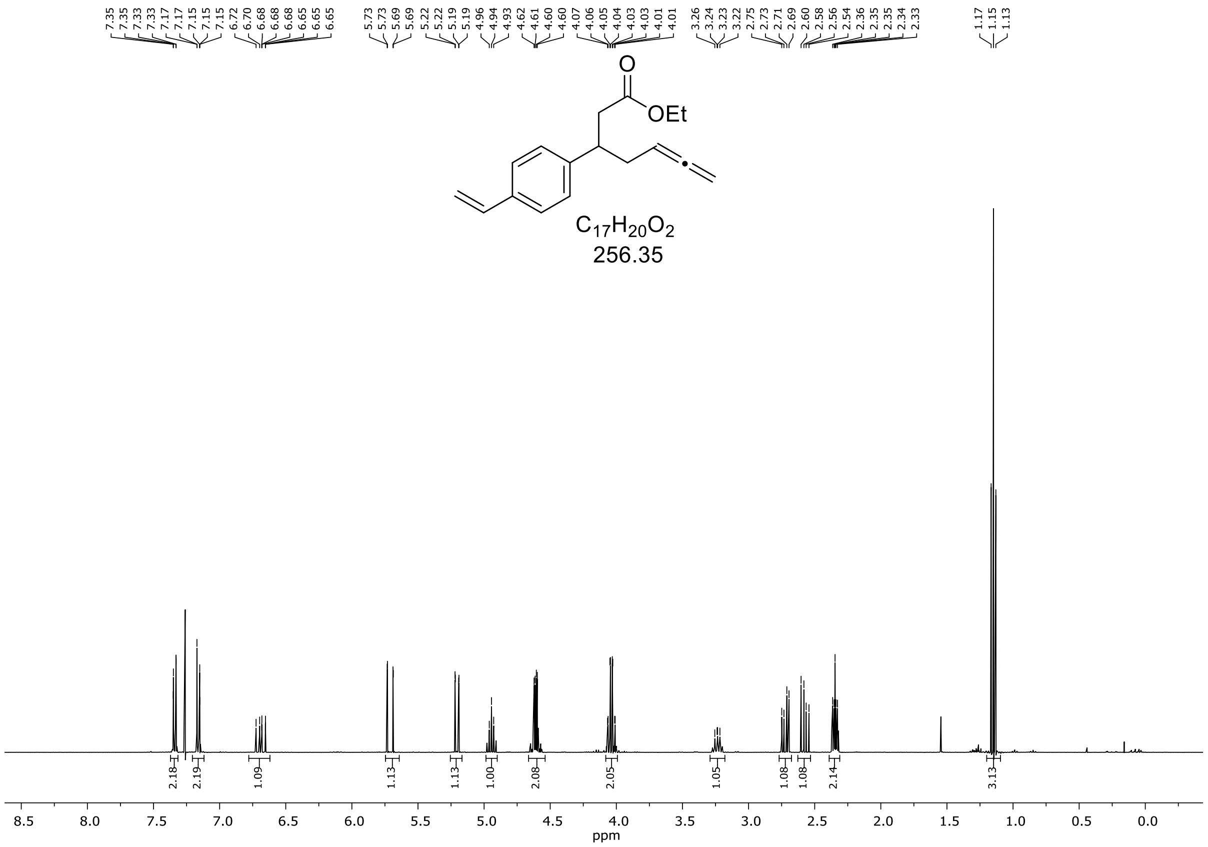

^{13}C-NMR

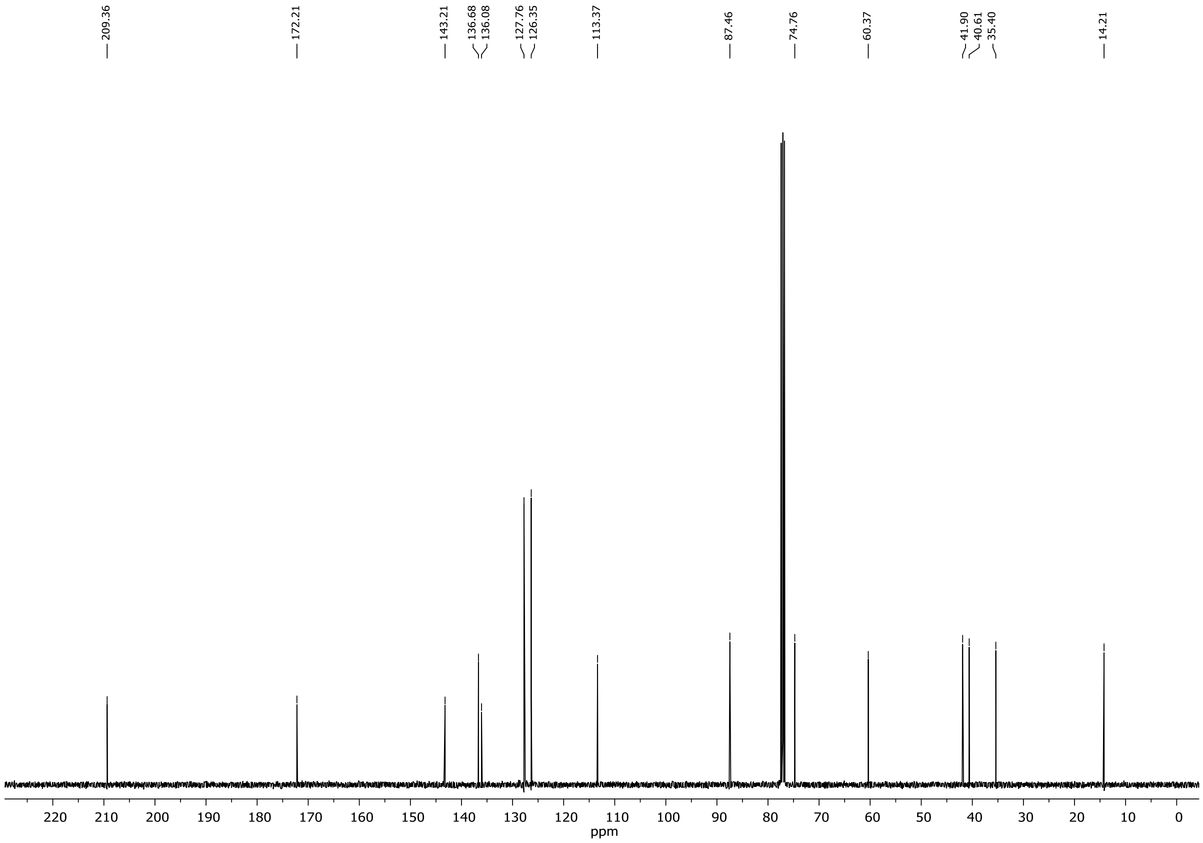

3-(-4-vinylphenyl)hepta-5.6-dien-1-ol 86

^{1}H-NMR

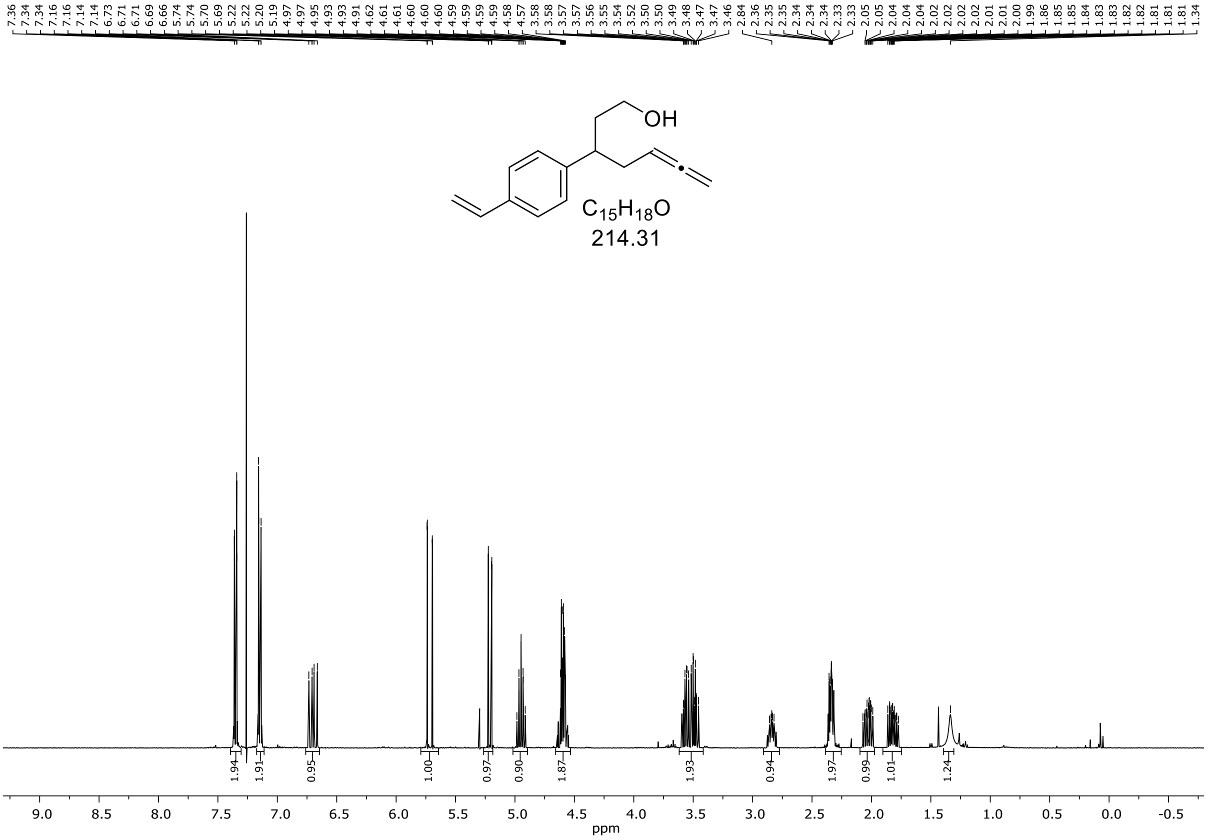

^{13}C-NMR

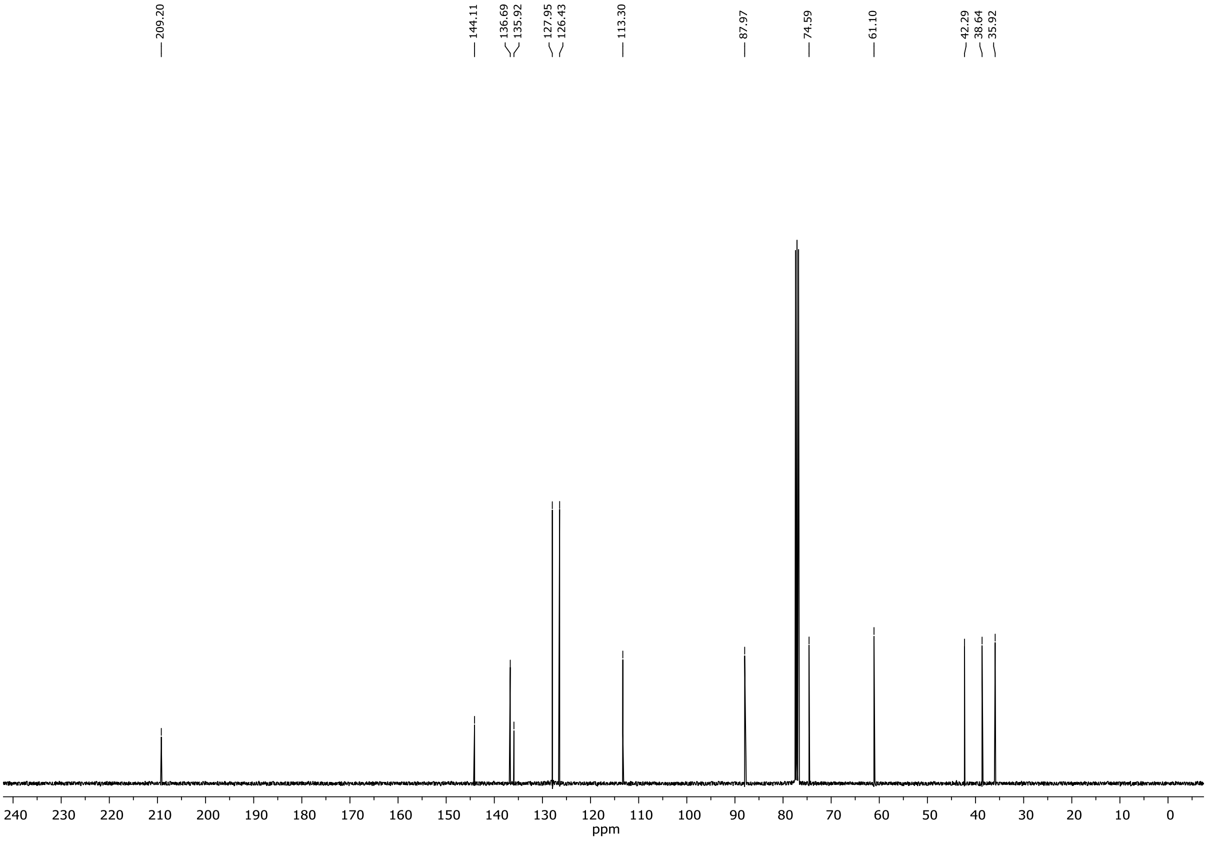

ethyl 3-(4-trifluoromethyl)phenyl)hepta-5,6-dienoate 87

^{1}H-NMR

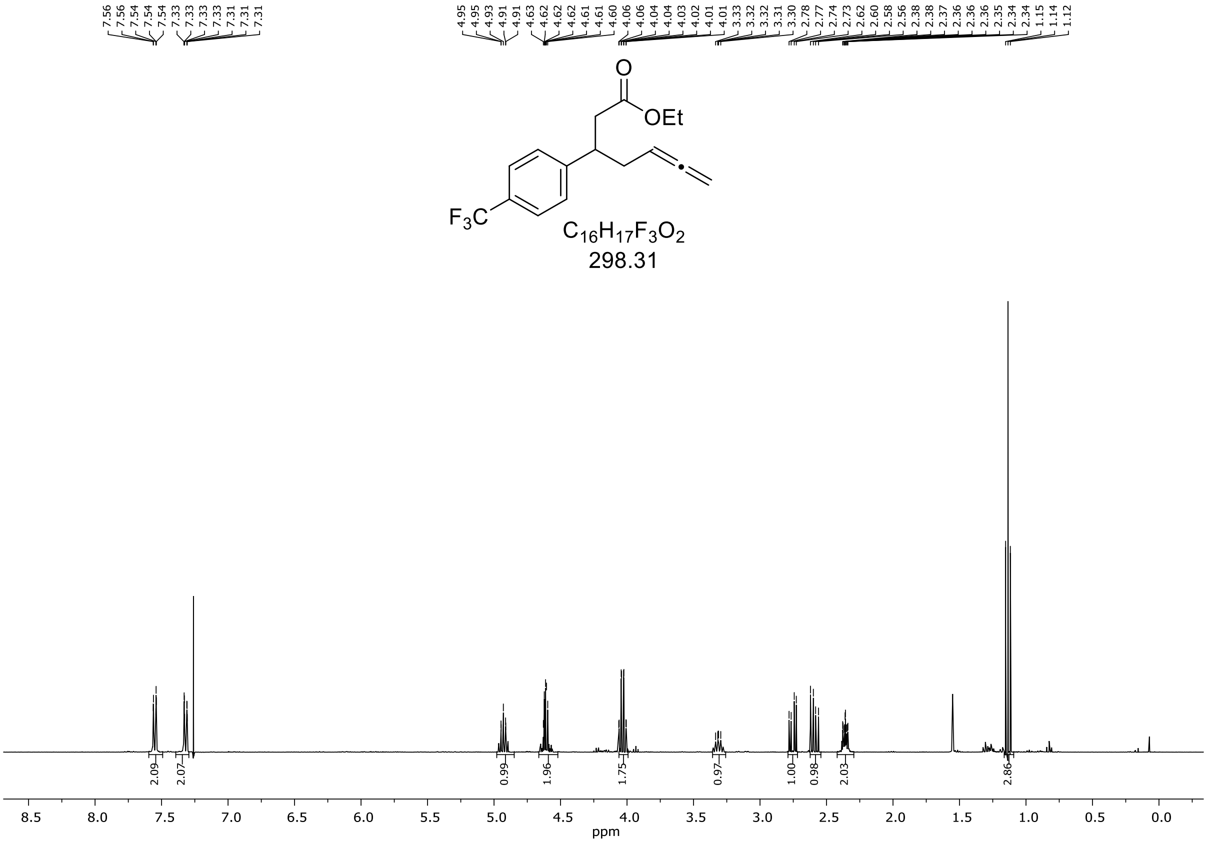

$C_{16}H_{17}F_3O_2$
298.31

^{13}C-NMR

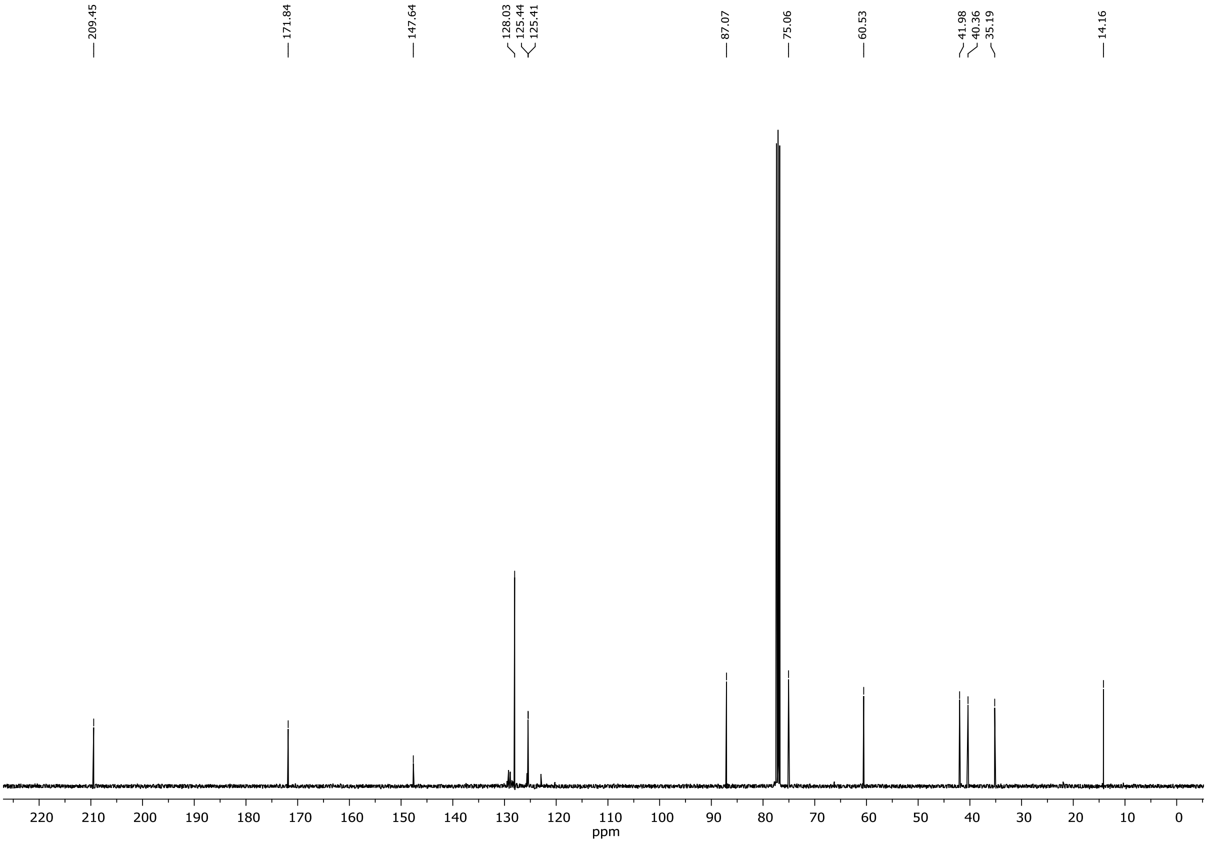

3-(4-trifluoromethyl)phenyl)hepta-5,6-dienol 88

^{1}H-NMR

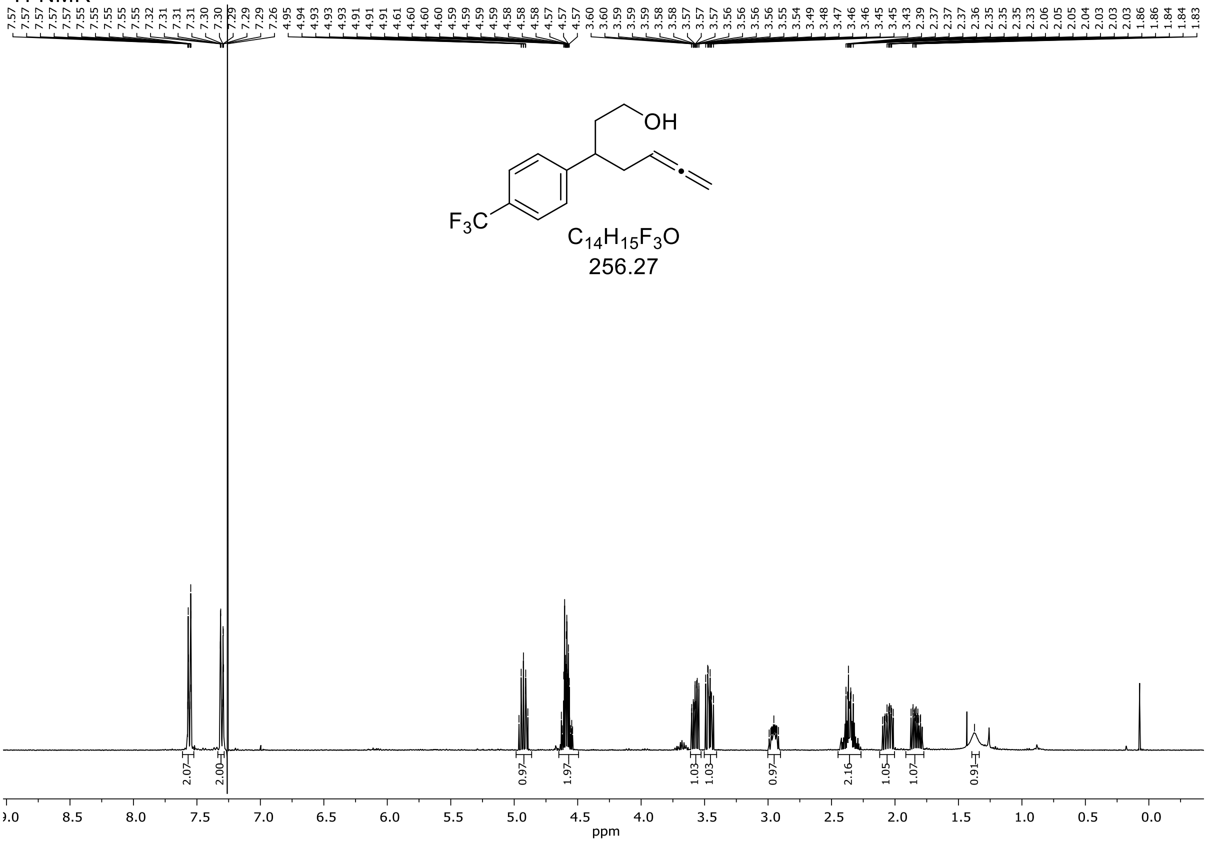

^{13}C-NMR

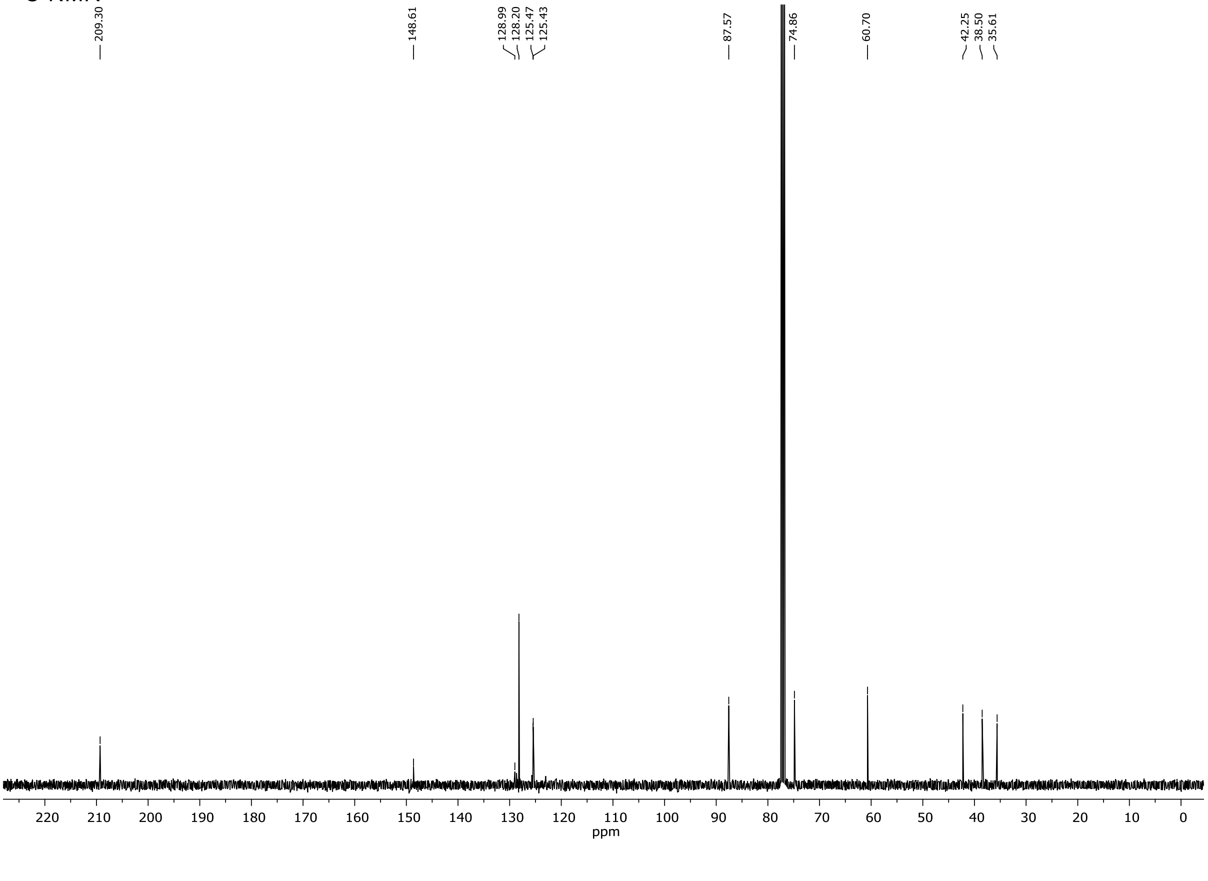

ethyl 3-(4-bromophenyl)hepta-5,6-dienoate 89

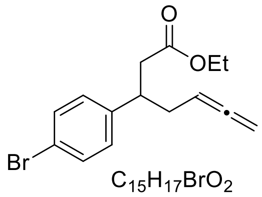

$C_{15}H_{17}BrO_2$
309.20

^{1}H-NMR

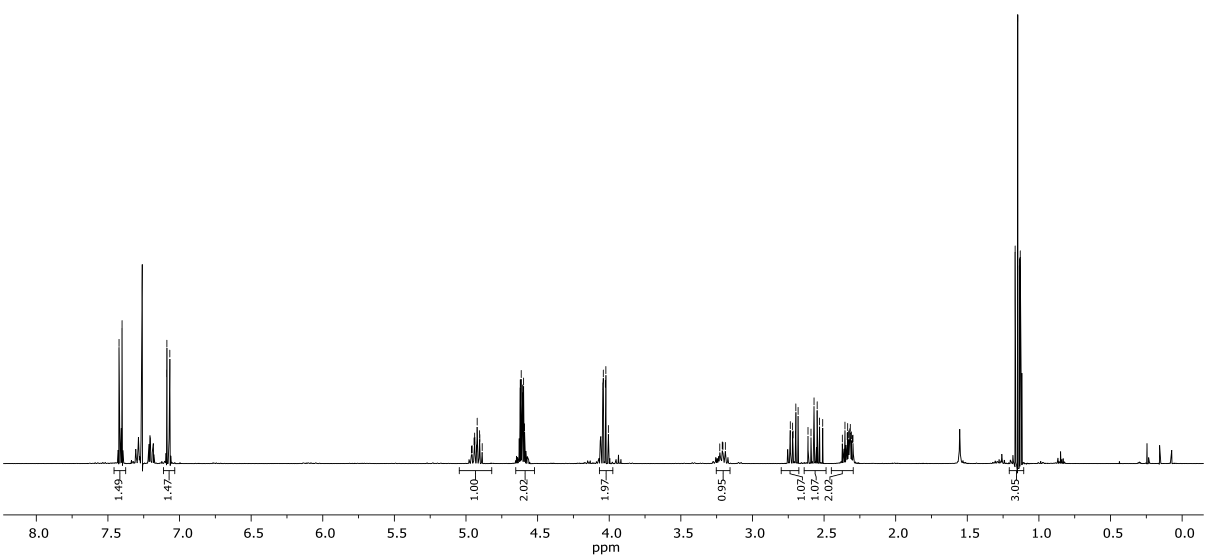

^{13}C-NMR

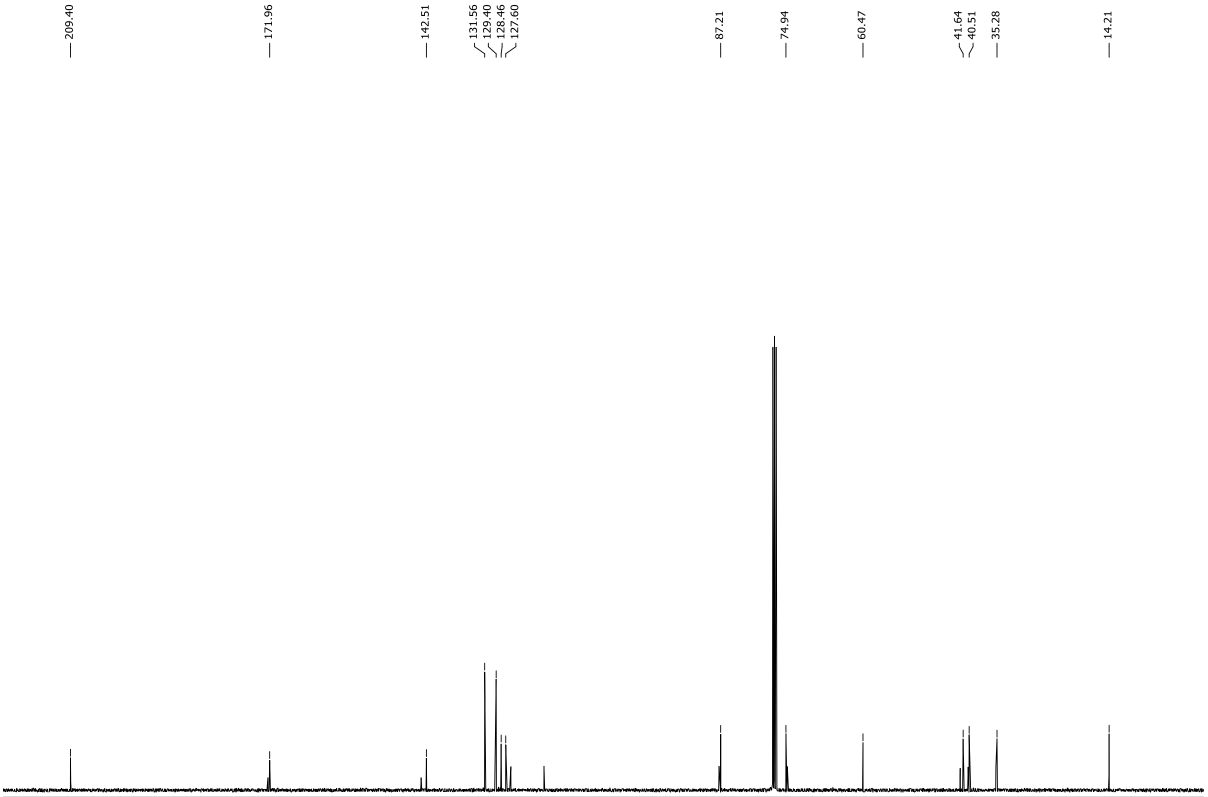

3-(4-bromophenyl)hepta-5,6-dienol 90

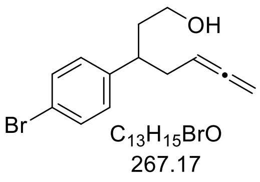

^{1}H-NMR

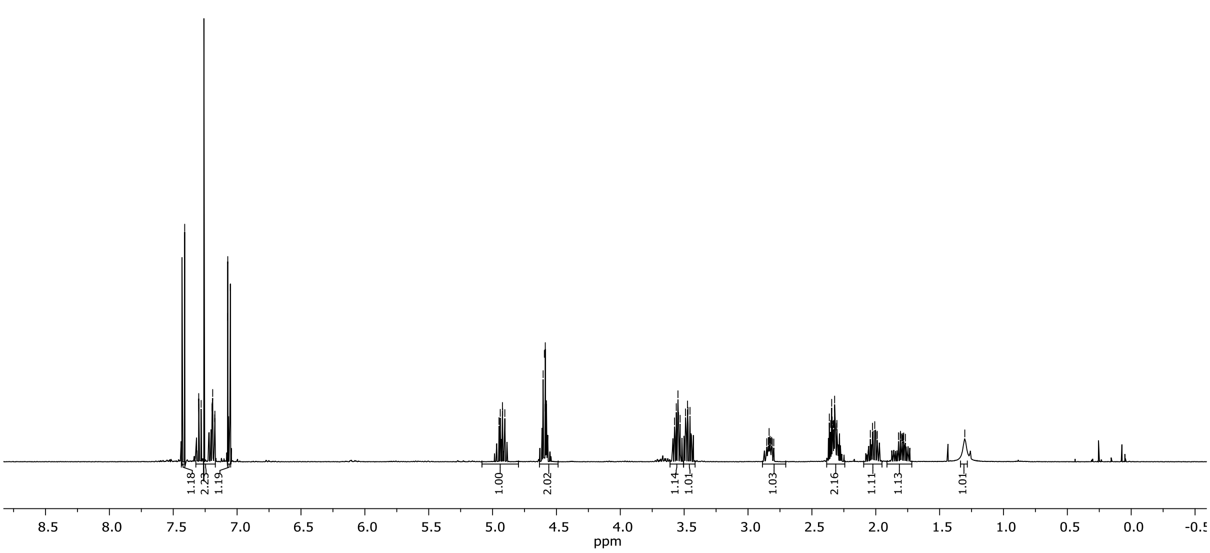

^{13}C-NMR

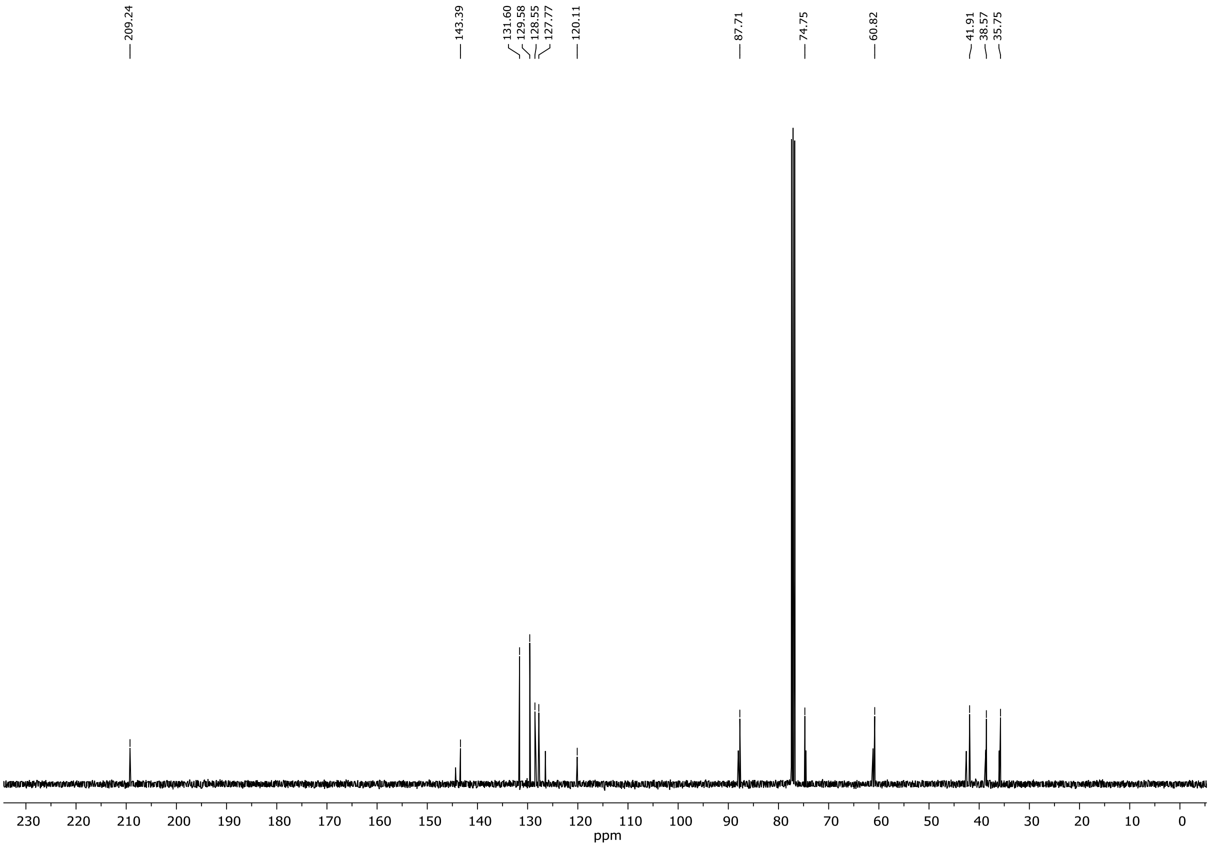

ethyl 3-(**methoxyphenyl)hepta-5,6-dienoate 91**

^{1}H-NMR

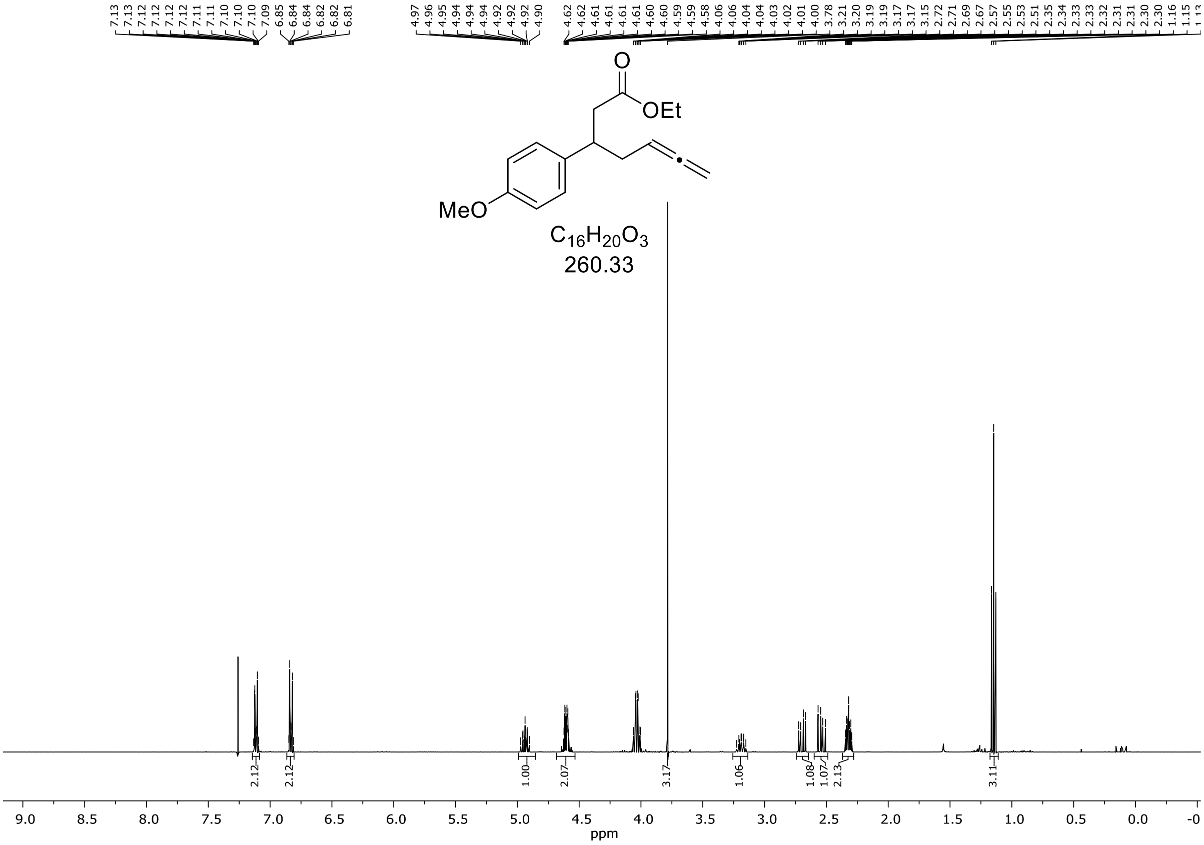

^{13}C-NMR

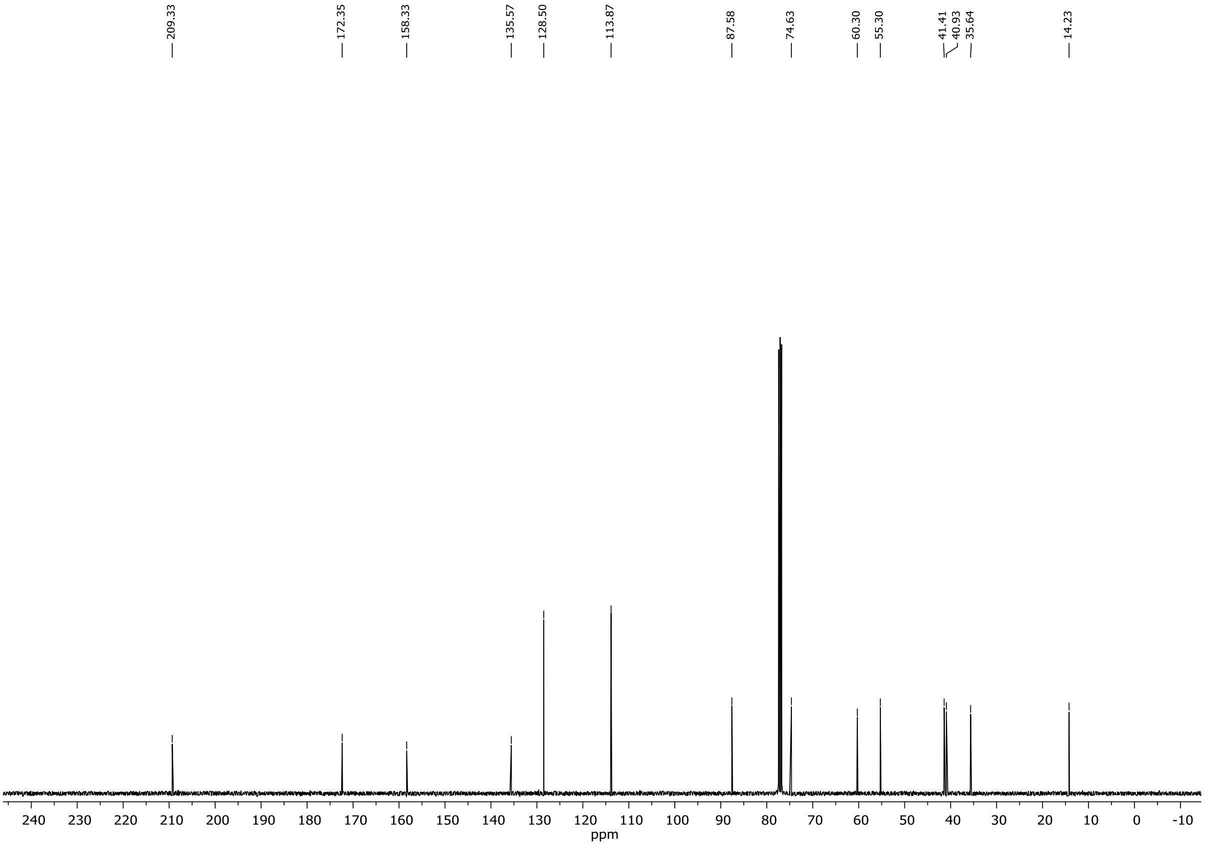

3-(4-methoxyphenyl)hepta-5,6-dienol 92

^{1}H-NMR

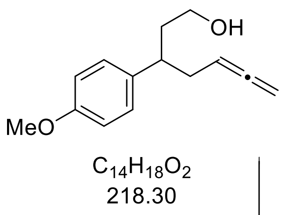

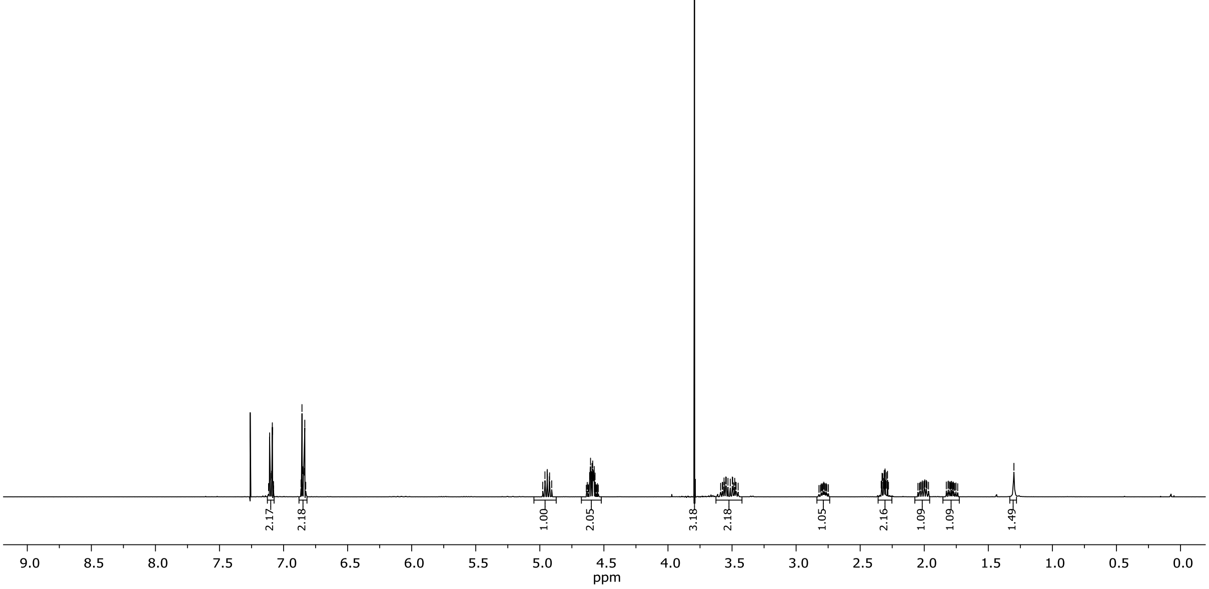

^{13}C-NMR

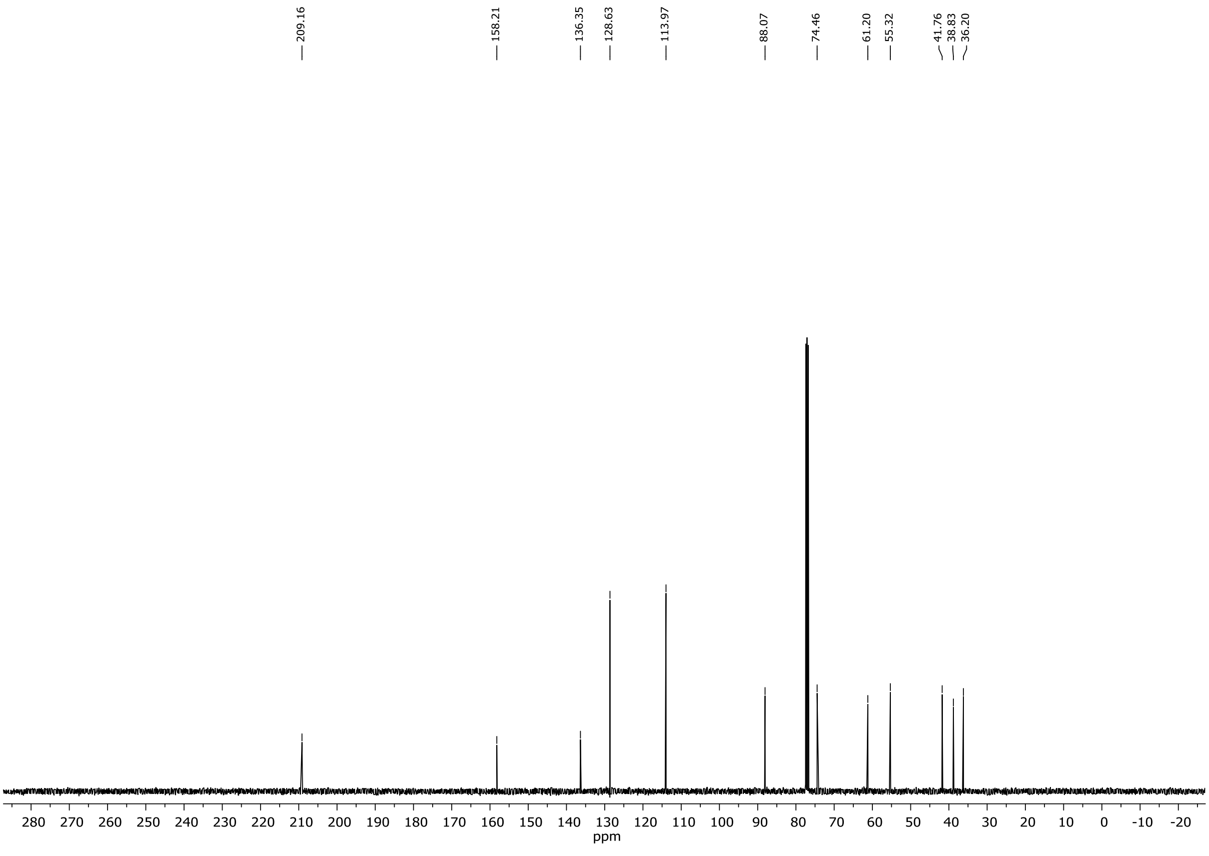

ethyl 3-(4-methylthio)phenyl)hepta-5,6-dienoate 93

^{1}H-NMR

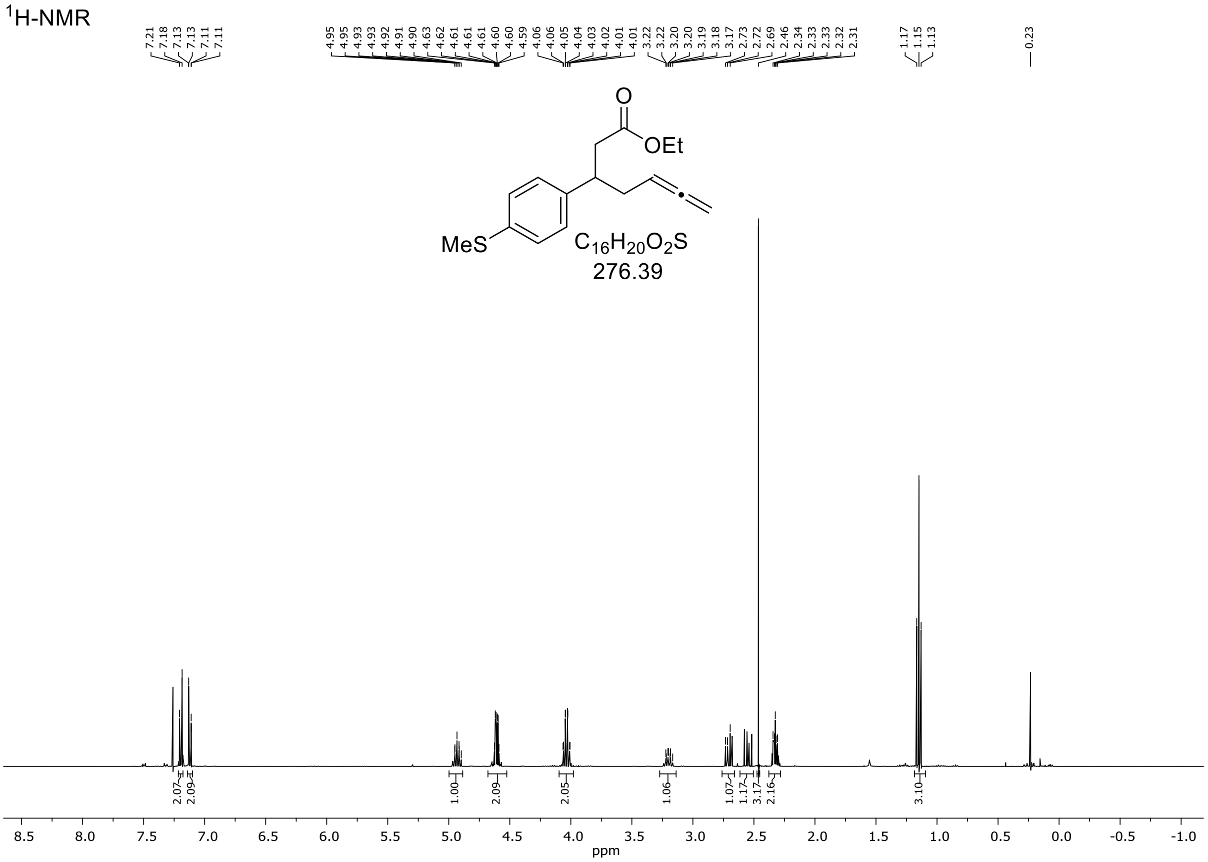

^{13}C-NMR

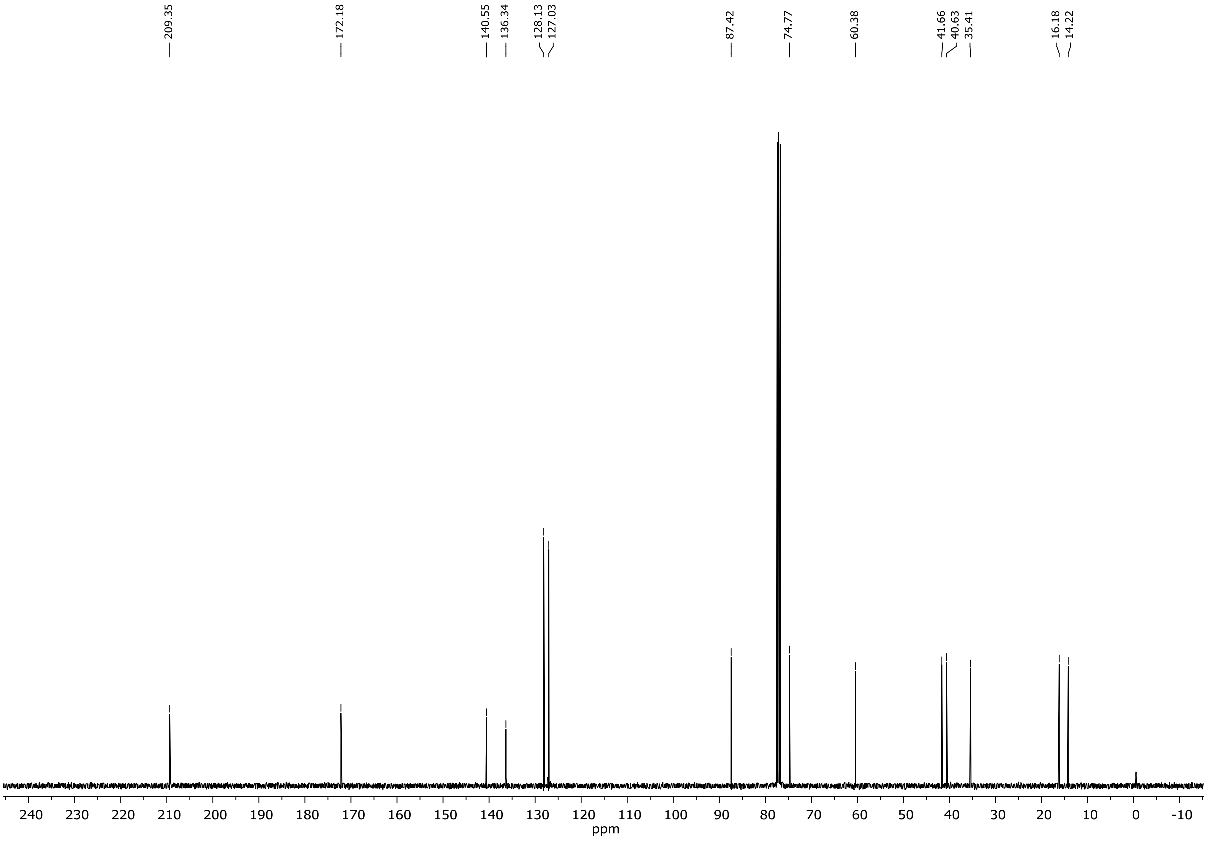

3-(4-methylthio)phenyl)hepta-5,6-dienol 94

^{1}H-NMR

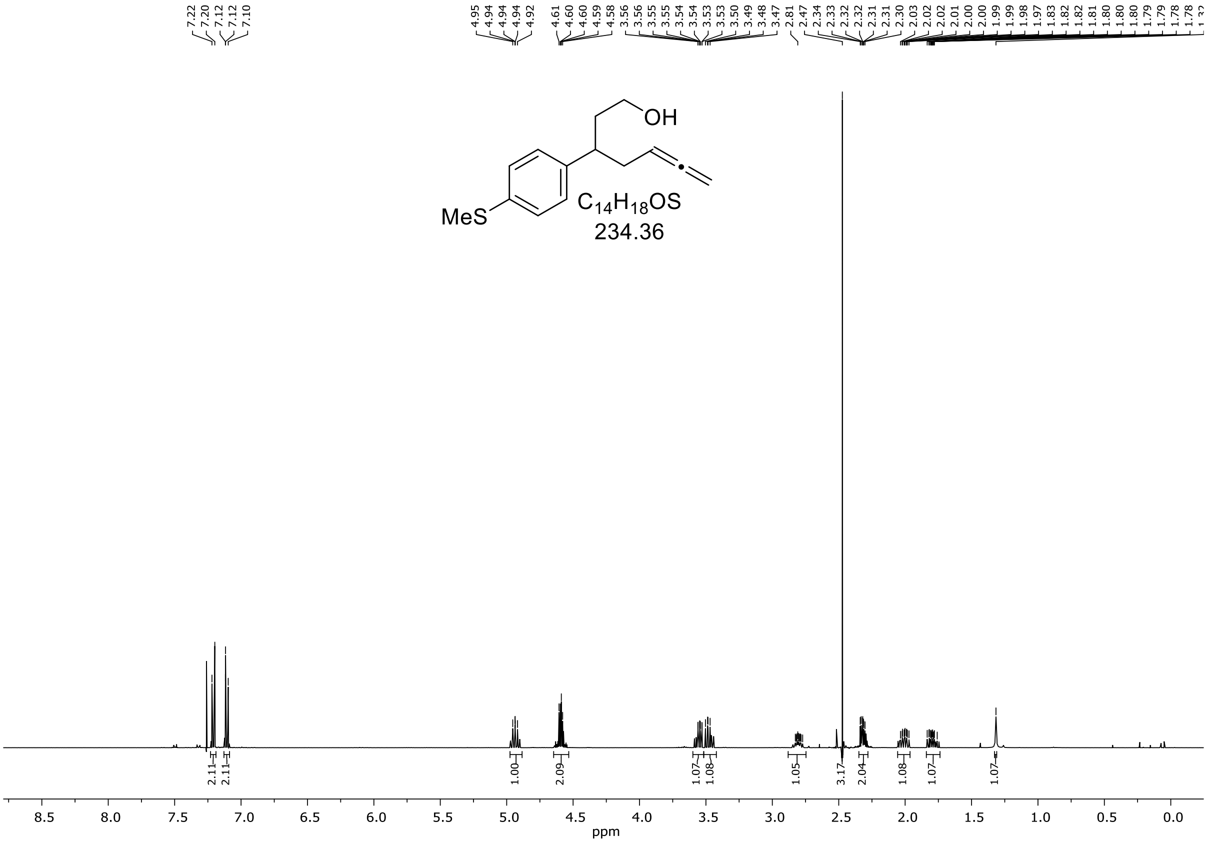

^{13}C-NMR

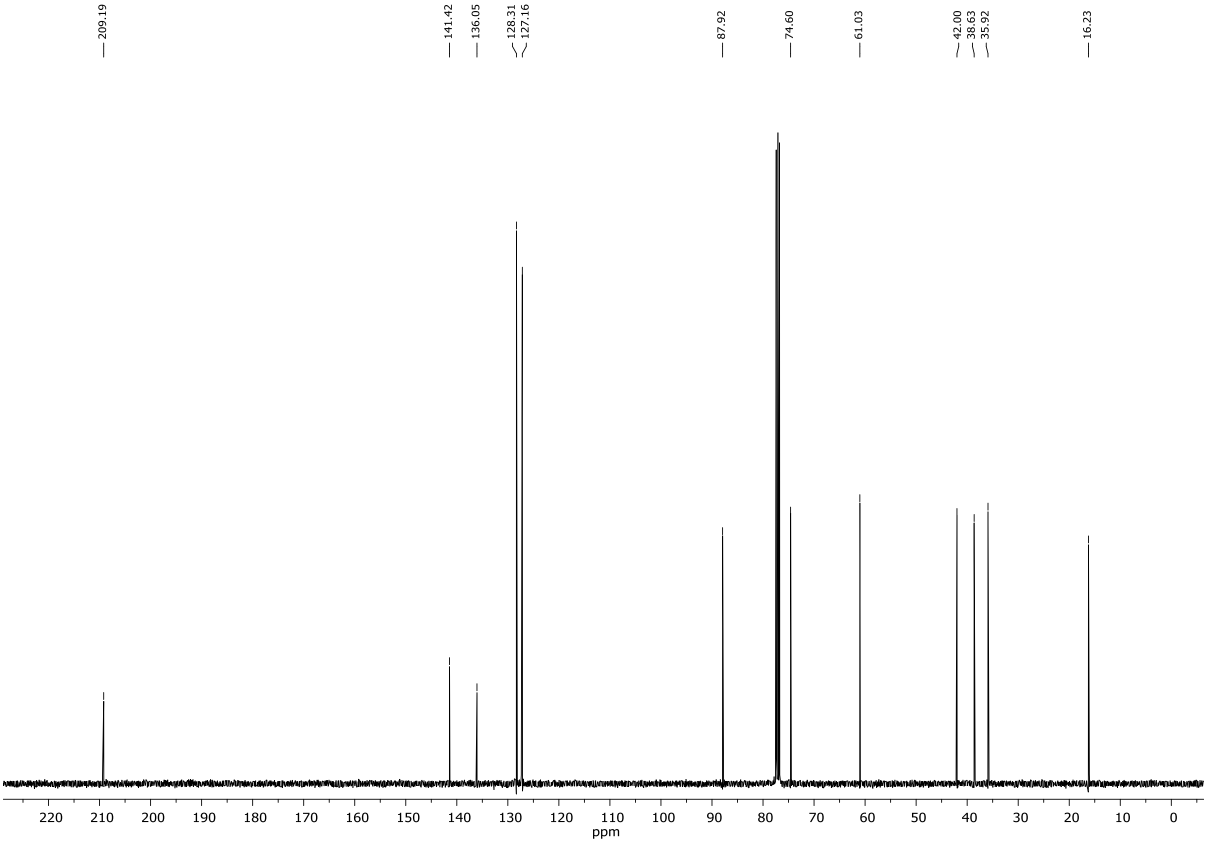

ethyl 3-(4-methoxyhenyl)-4-dimethylhepta-5,6-dienoate 95

^{1}H-NMR

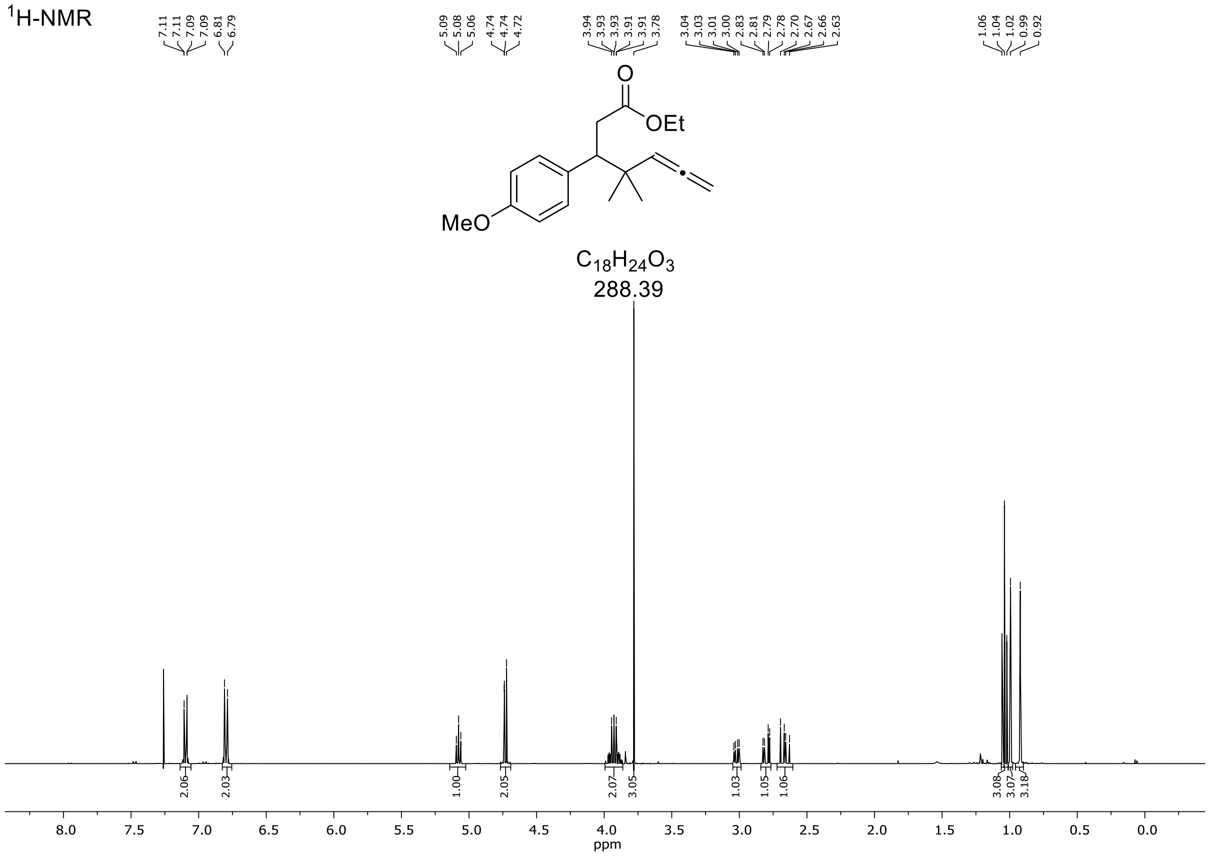

^{13}C-NMR

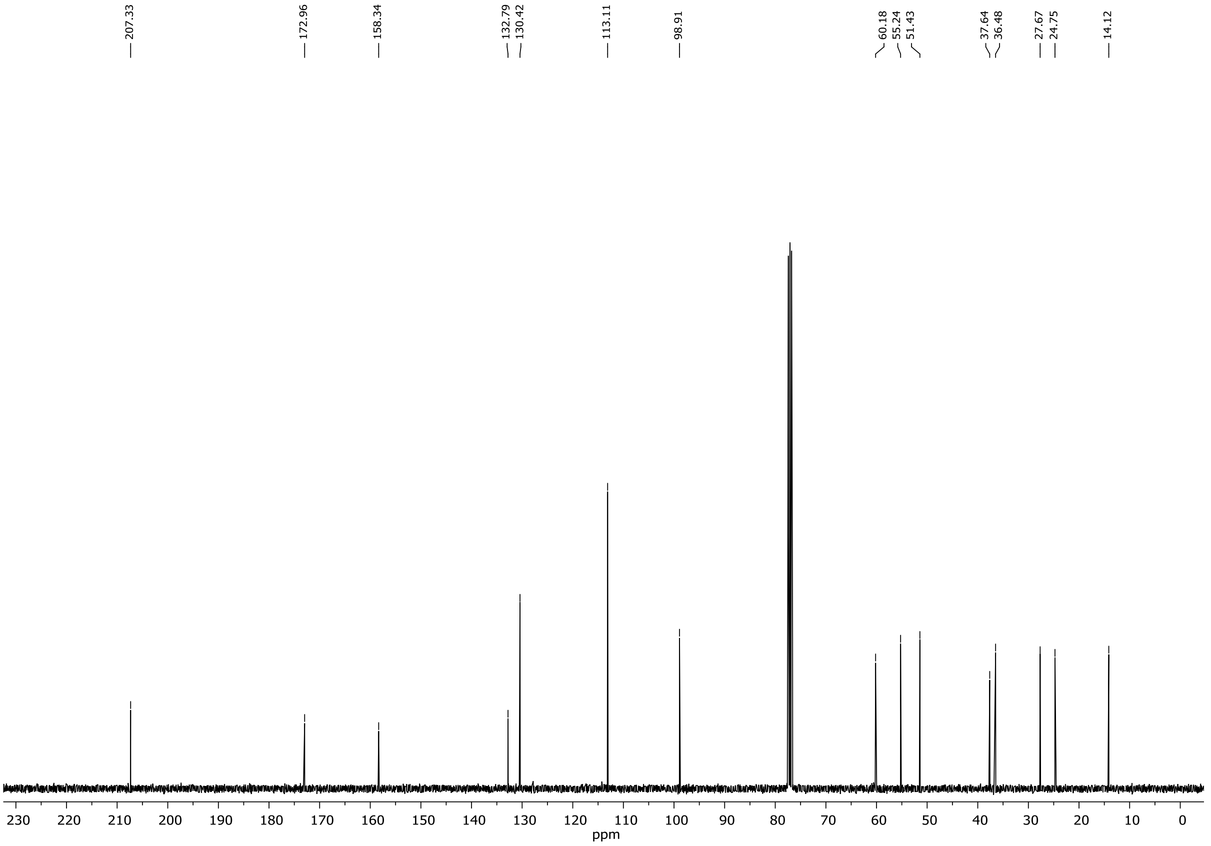

3-(4-methoxyhenyl)-4-dimethylhepta-5,6-dienol 96

^{1}H-NMR

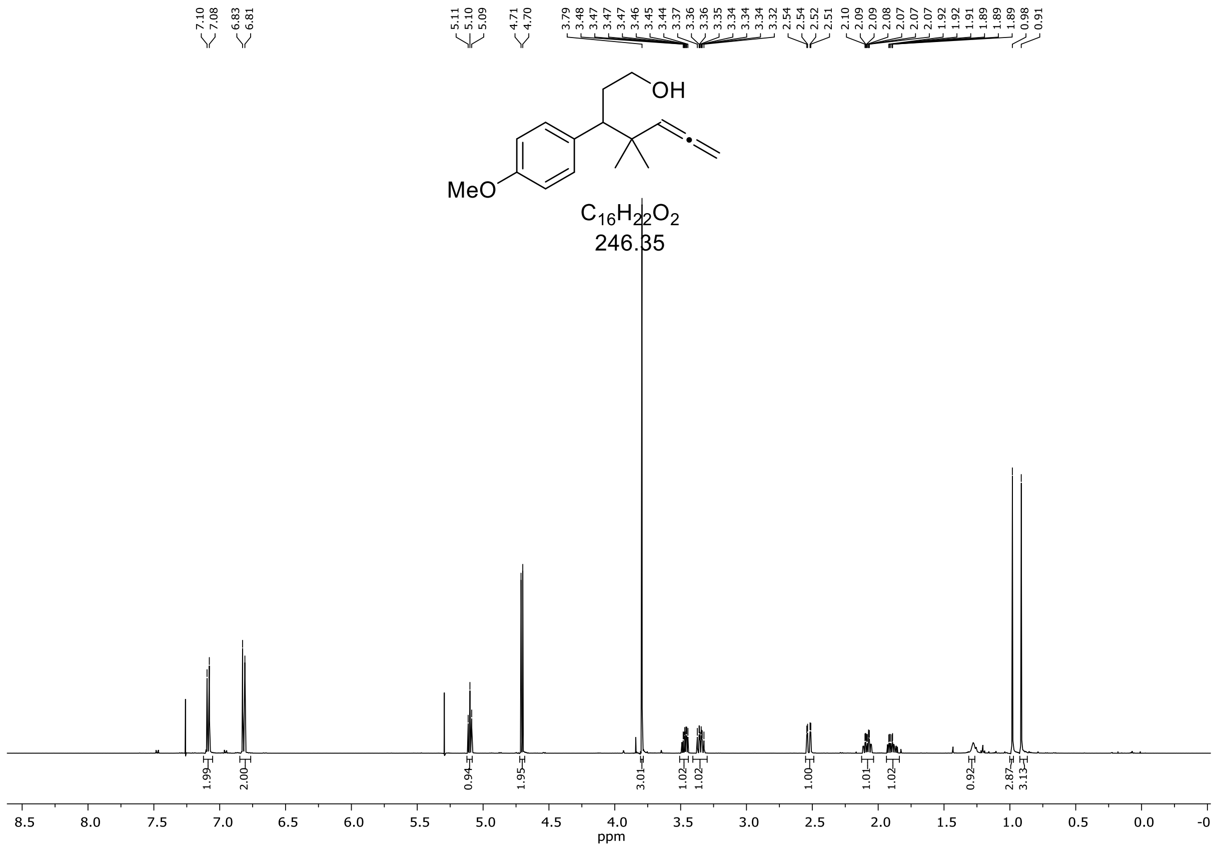

^{13}C-NMR

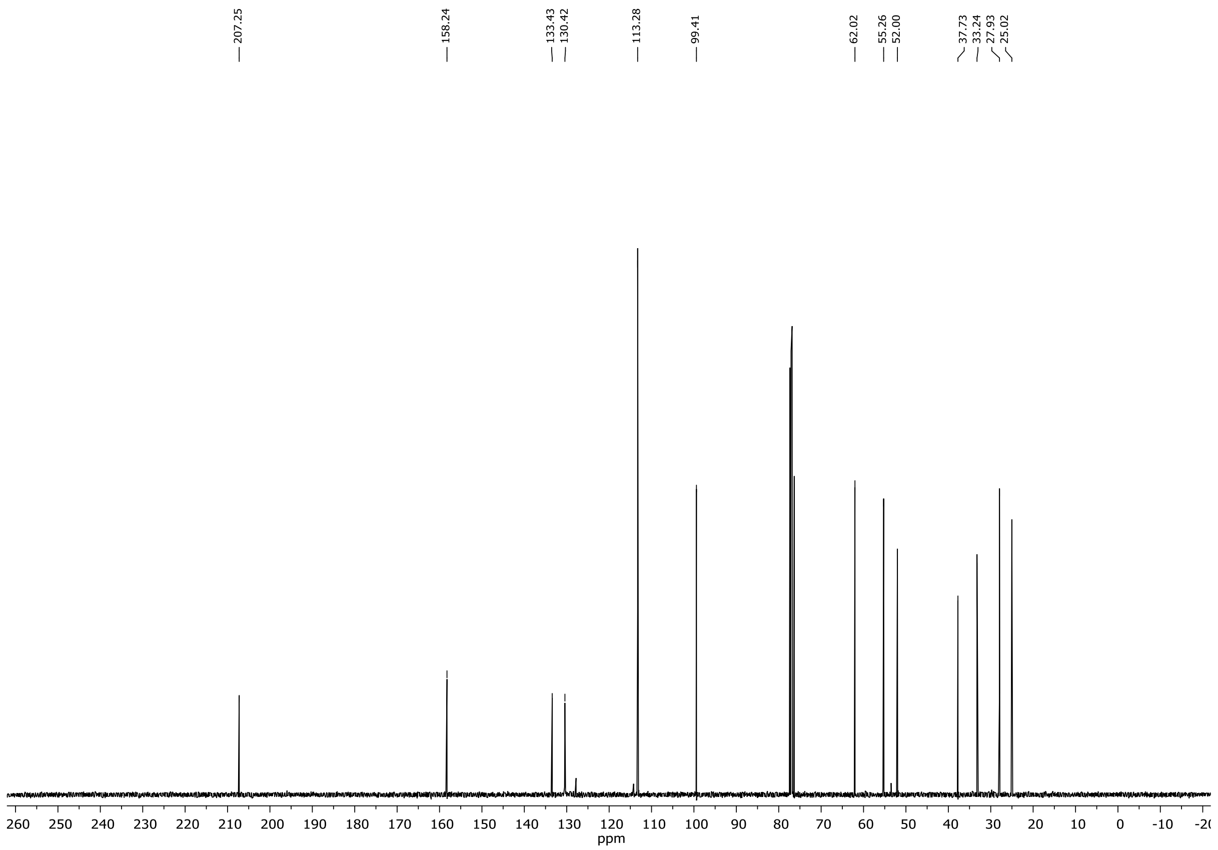

ethyl undeca-3,4-dienoate 97

^{1}H-NMR

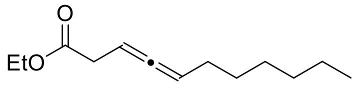

$C_{13}H_{22}O_2$
210.32

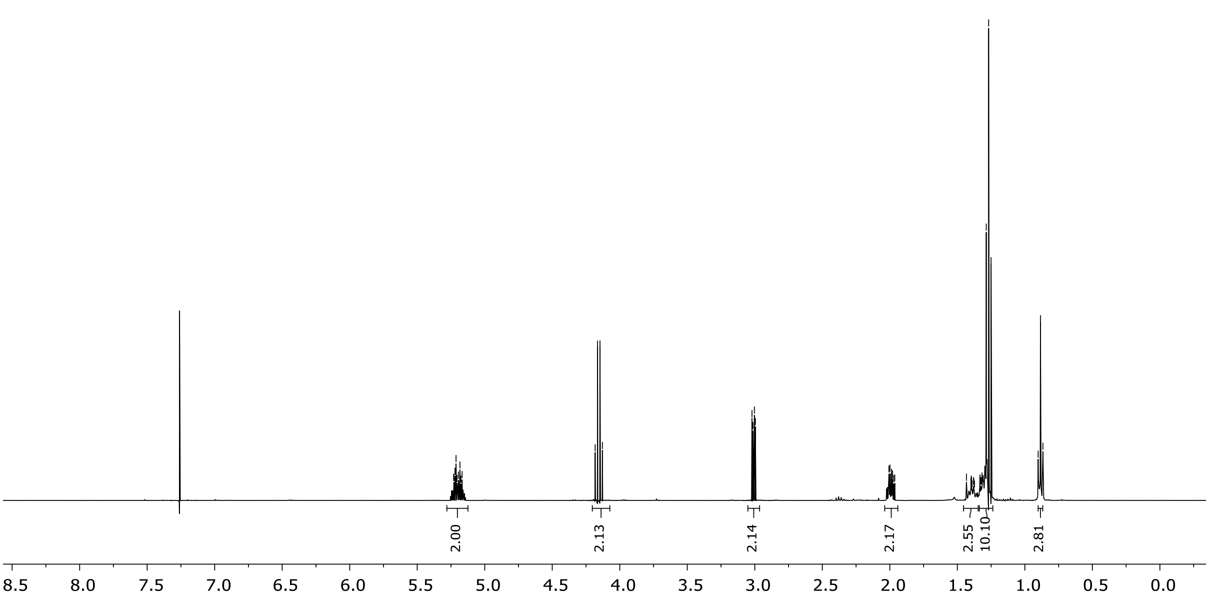

^{13}C-NMR

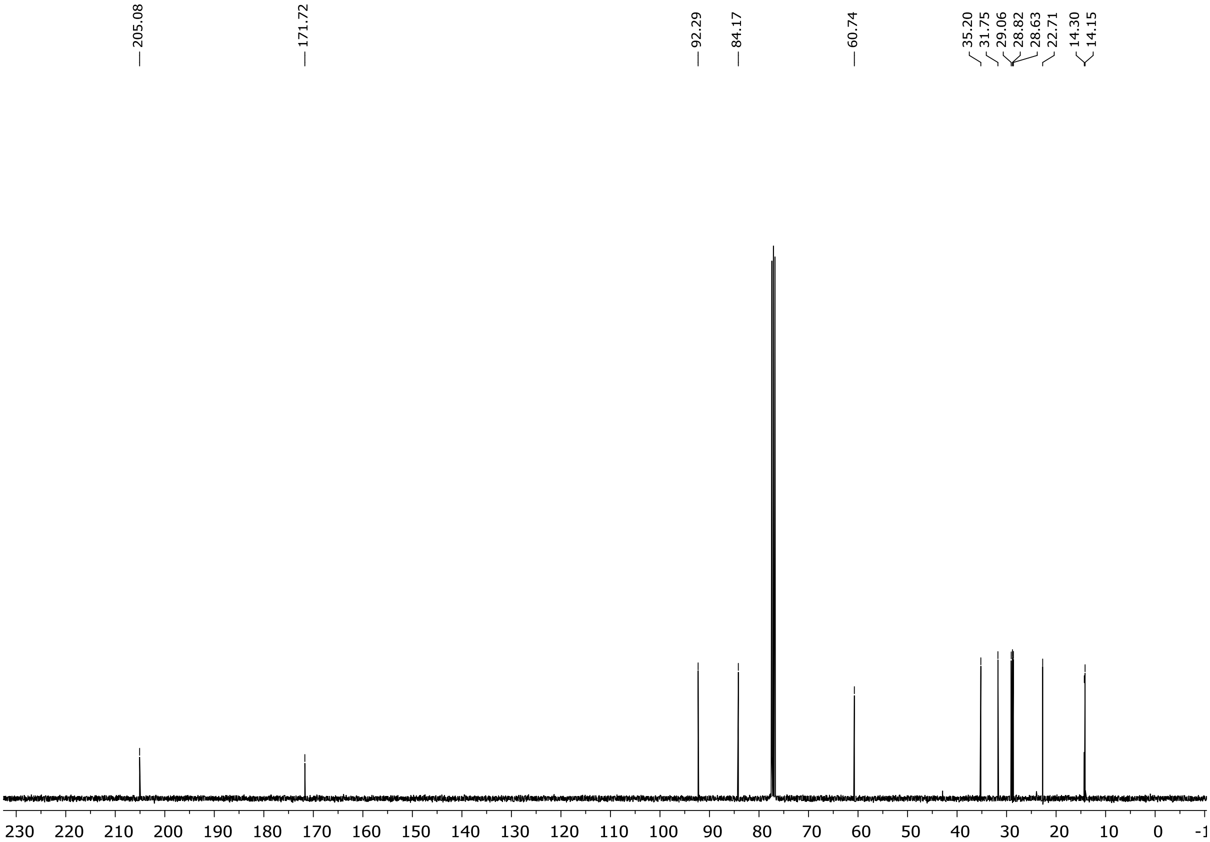

ethyl (E)-trideca-2,5,6-trienoate 98

^{1}H-NMR

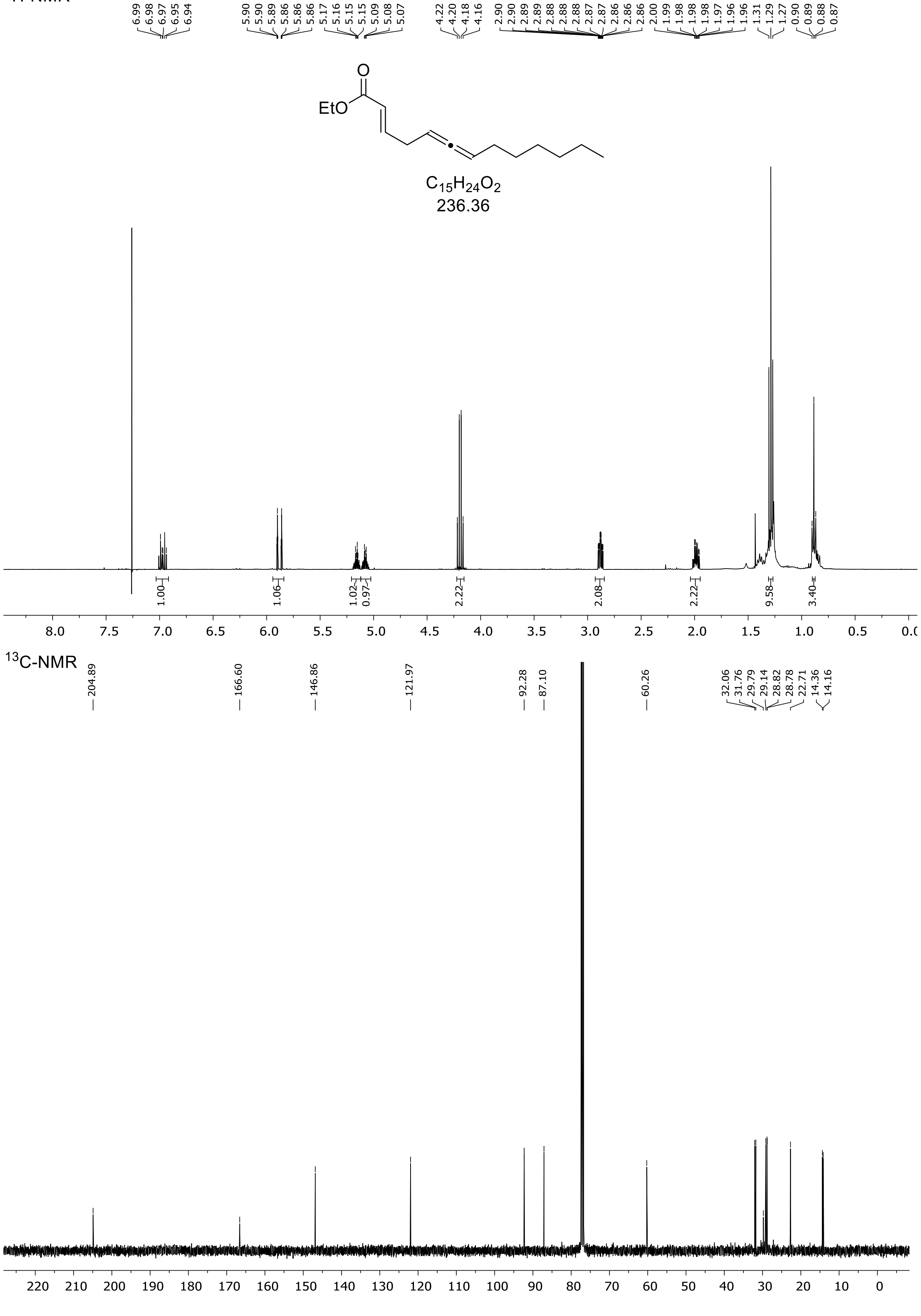

3-methyltrideca-5,6-dien-1-ol 99

^{1}H-NMR

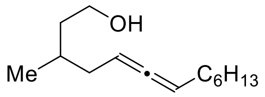

$C_{14}H_{26}O$
210.36

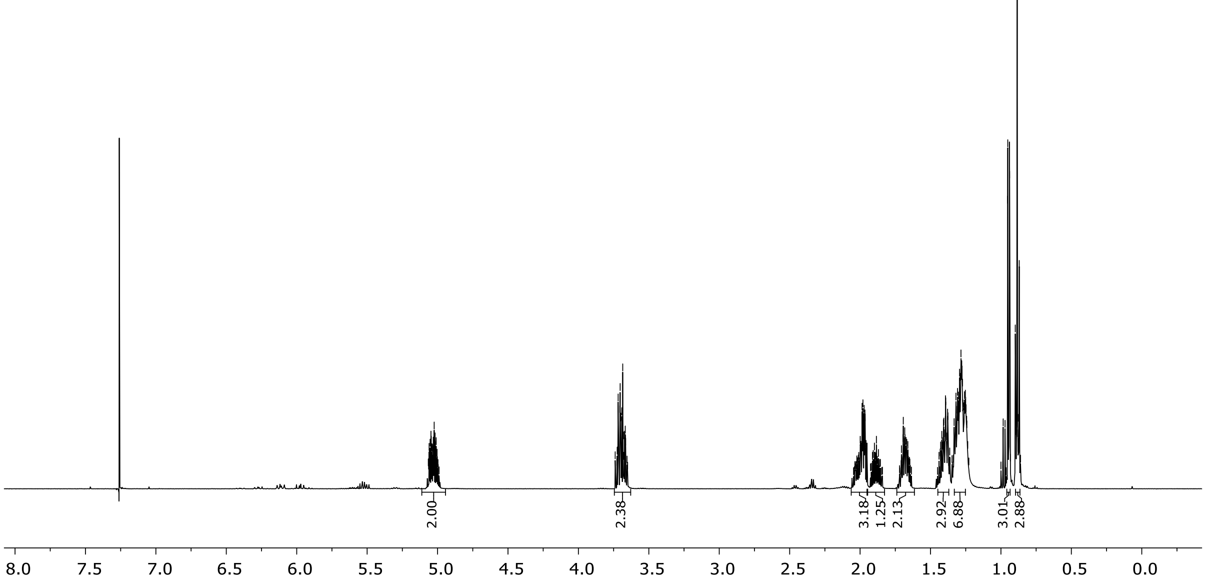

^{13}C-NMR

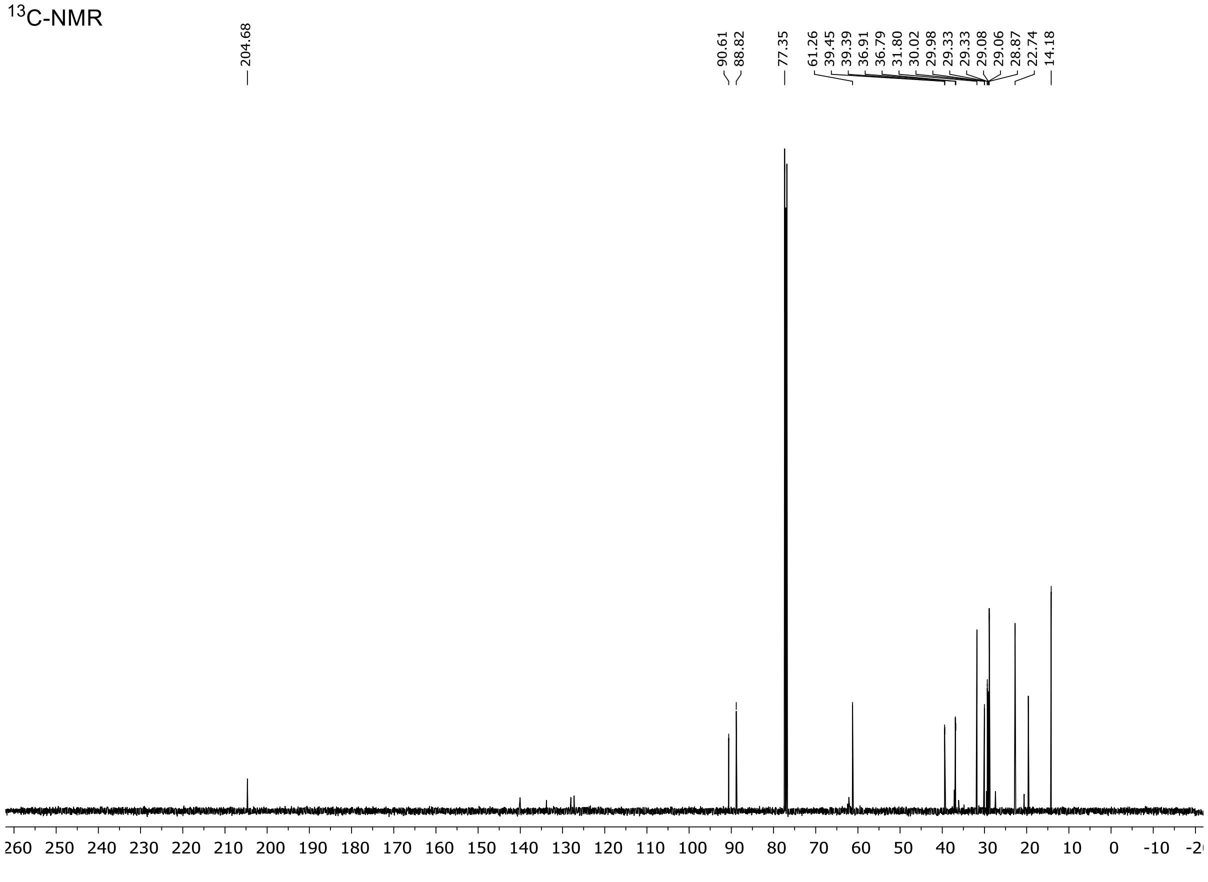

3-(deca-2,3-dien-1-yl)pentadecan-1-ol 100

^{1}H-NMR

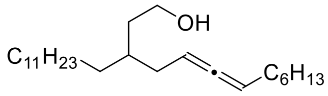

$C_{25}H_{48}O$
364.66

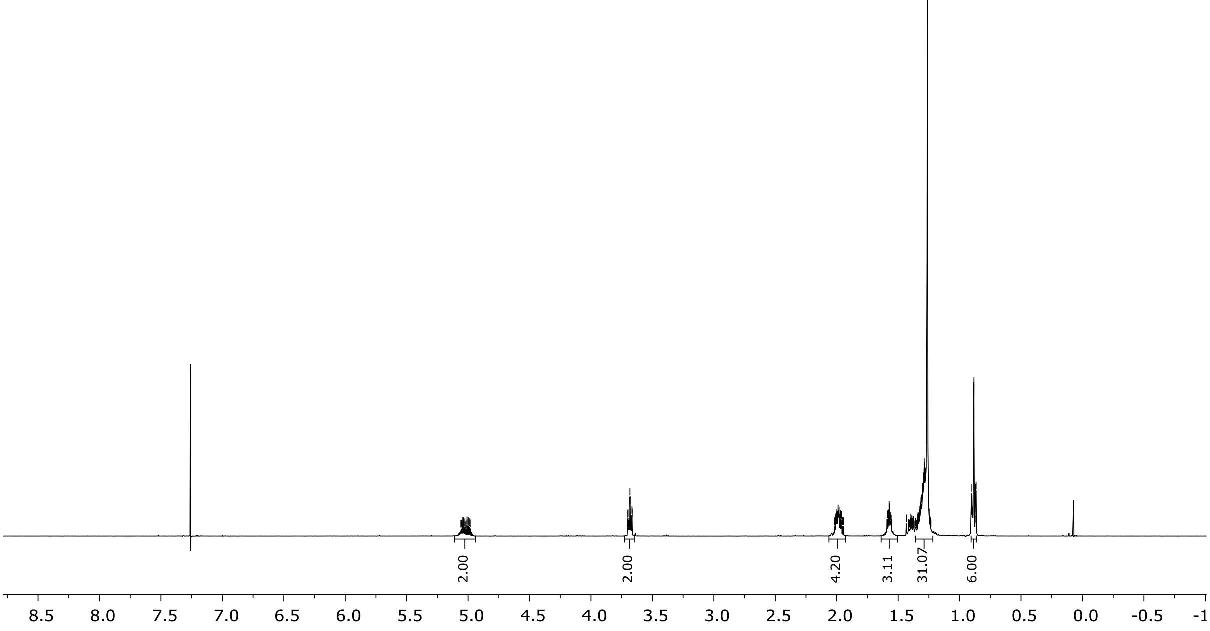

^{13}C-NMR

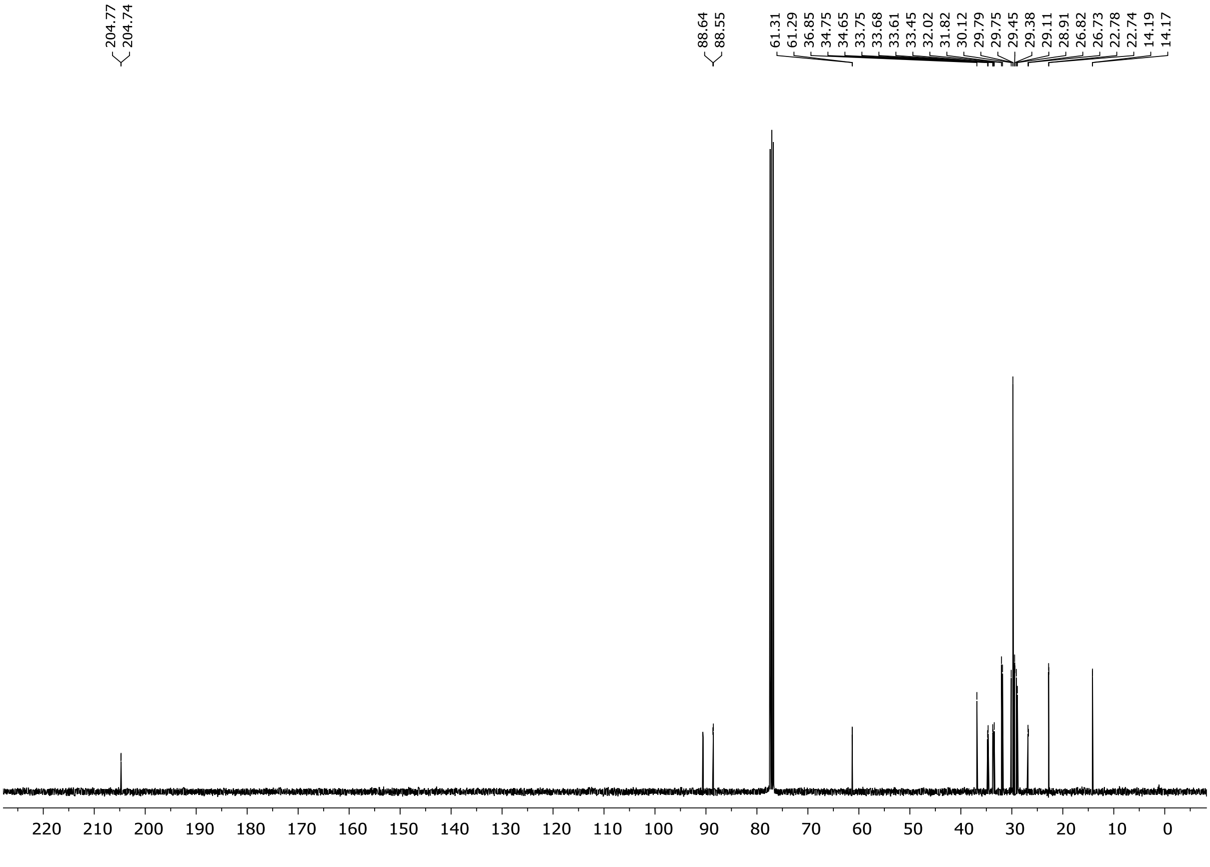

3-neopentyltrideca-5,6-dien-1-ol 101

^{1}H-NMR

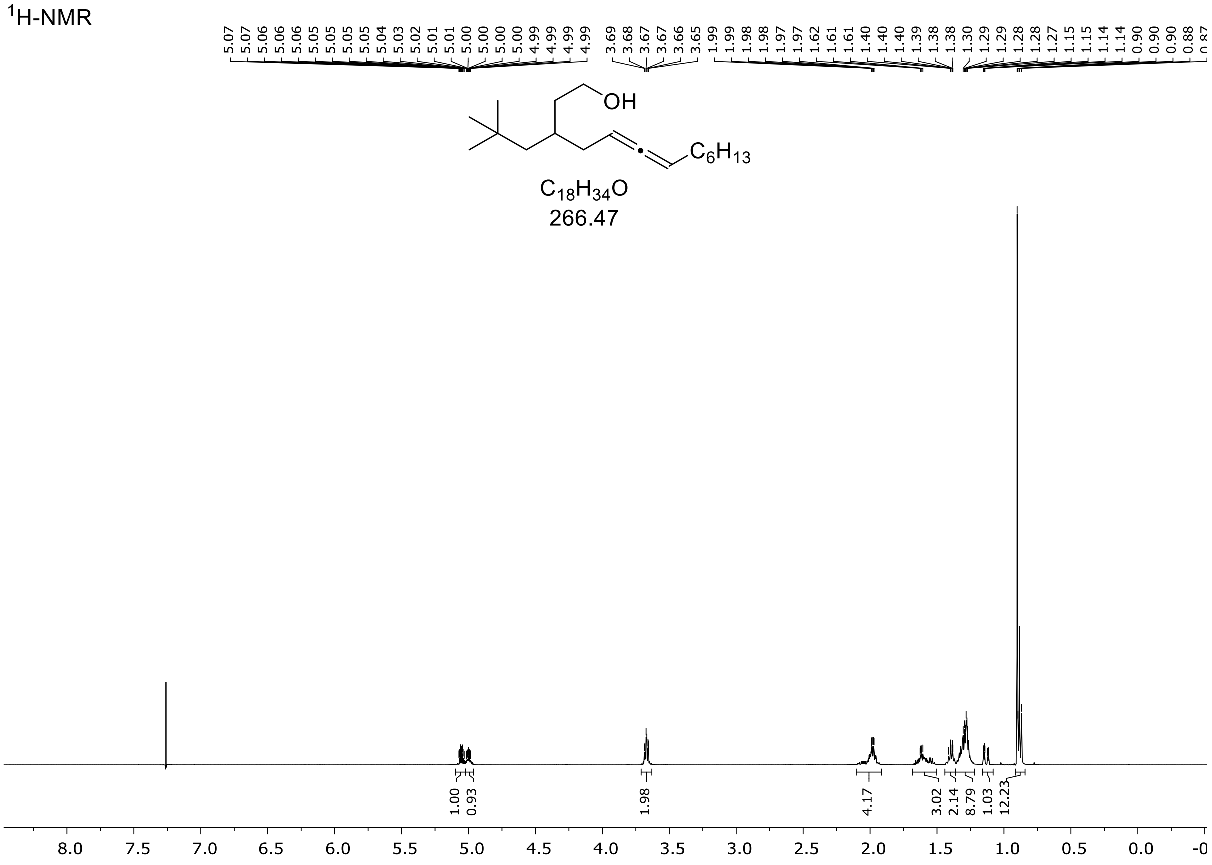

^{13}C-NMR

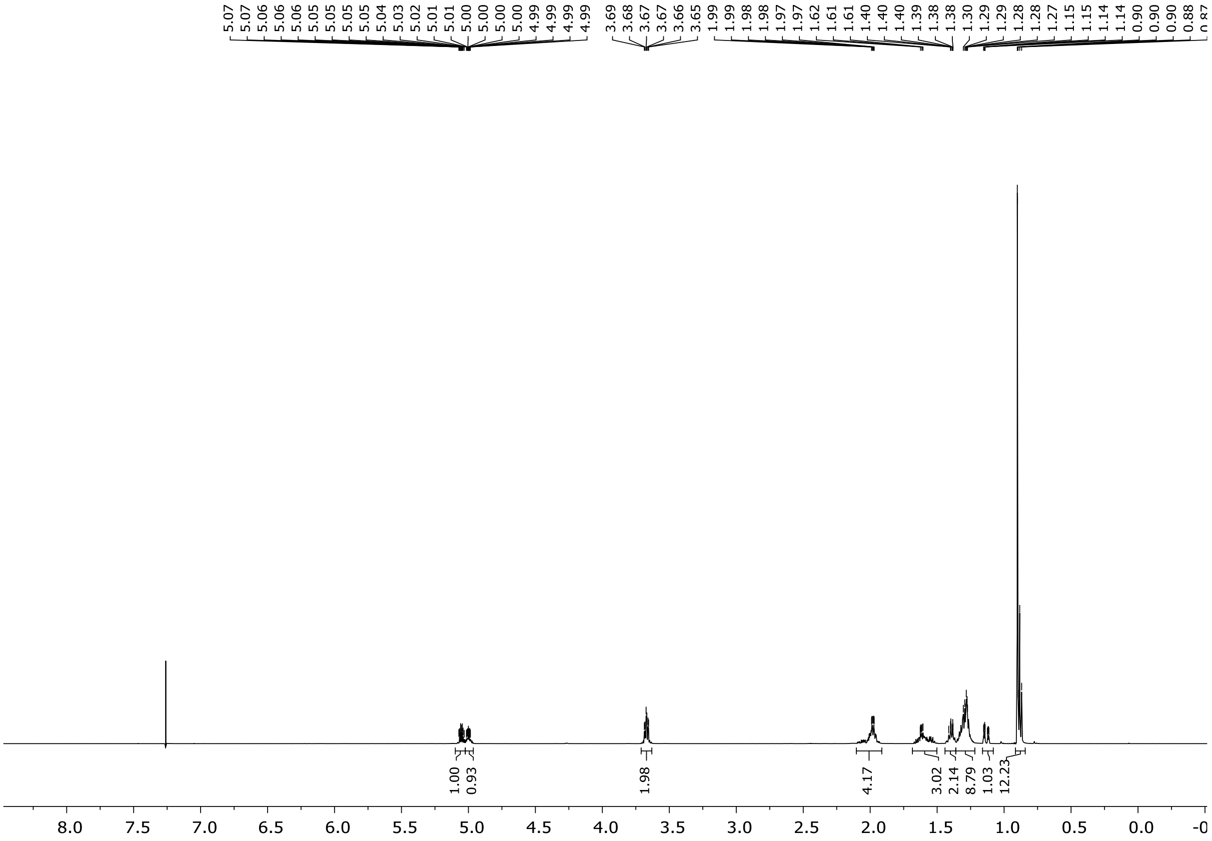

3-cyclopropyltrideca-5,6-dien-1-ol 102

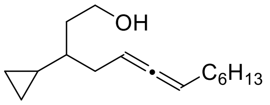

$C_{16}H_{28}O$
236.40

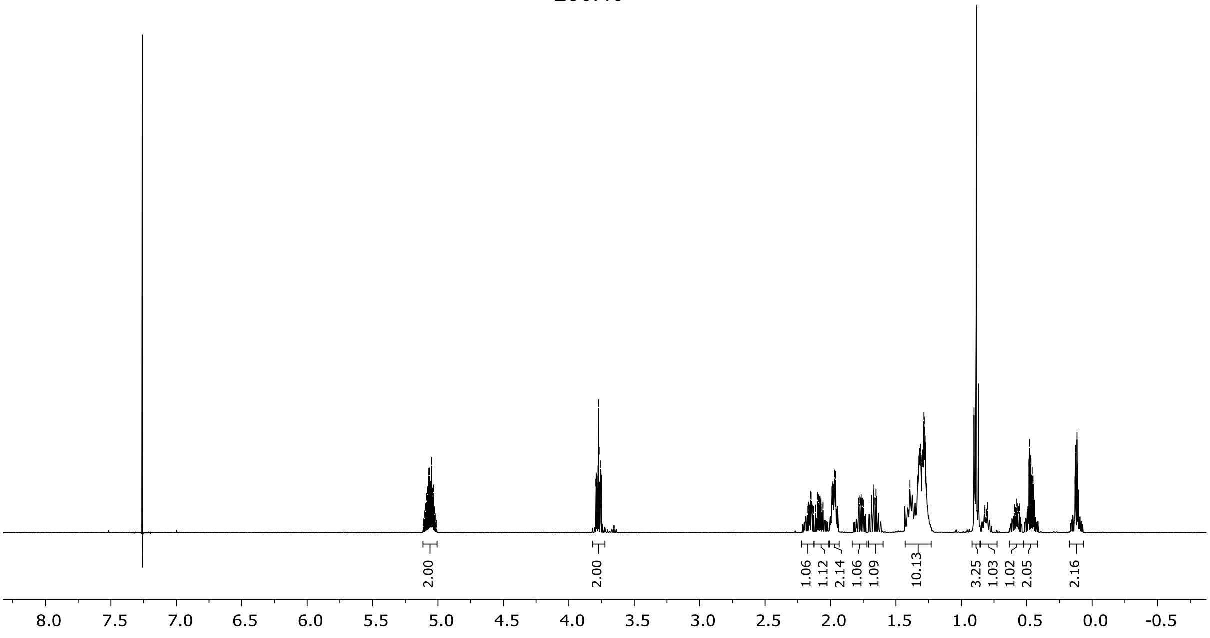

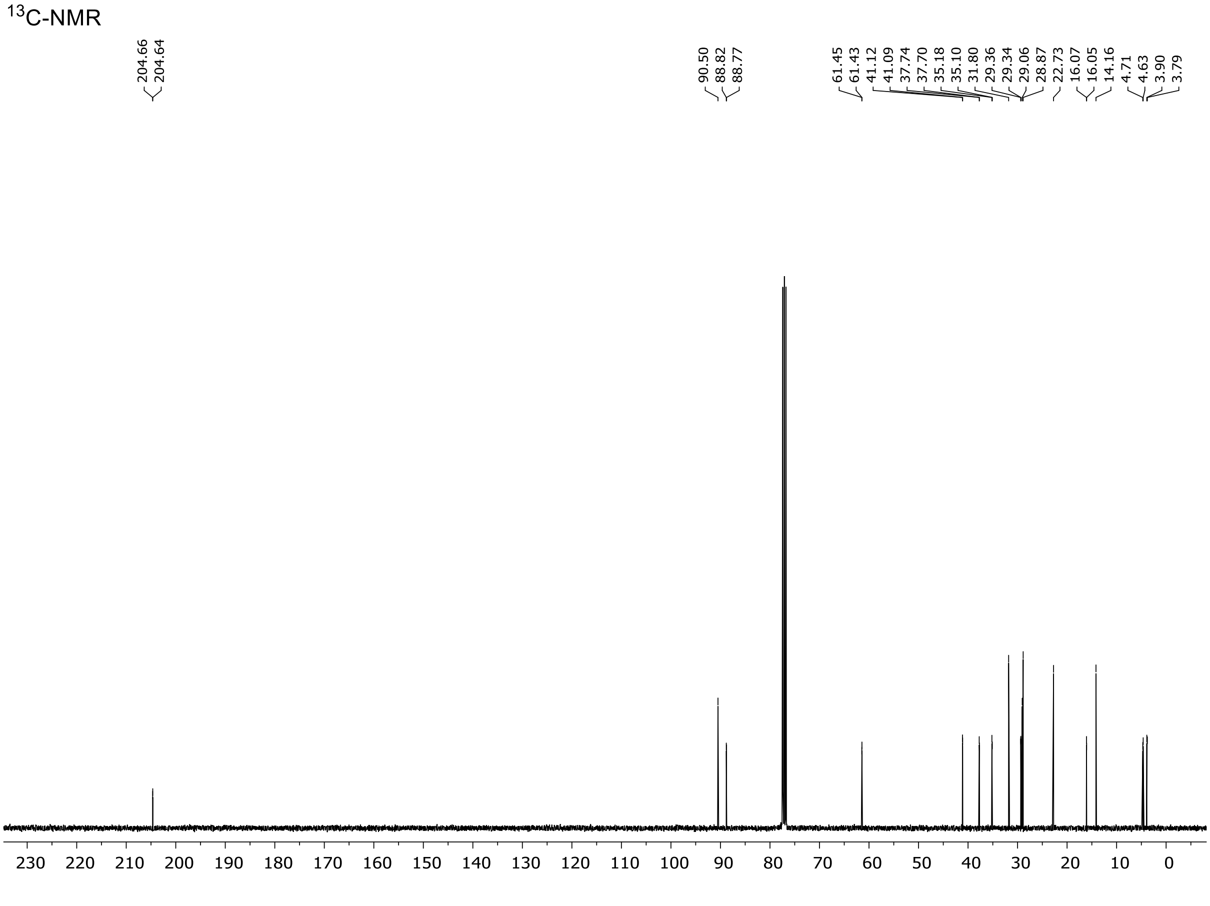

3-cyclohexyltrideca-5,6-dien-1-ol 103

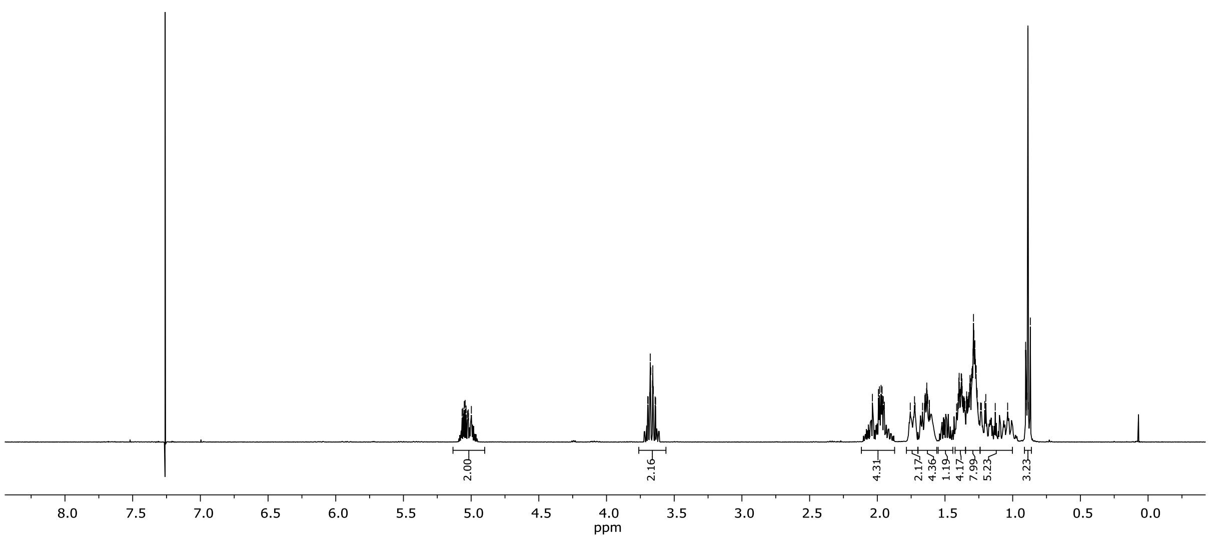

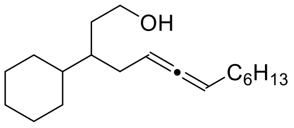

$C_{19}H_{34}O$
278.48

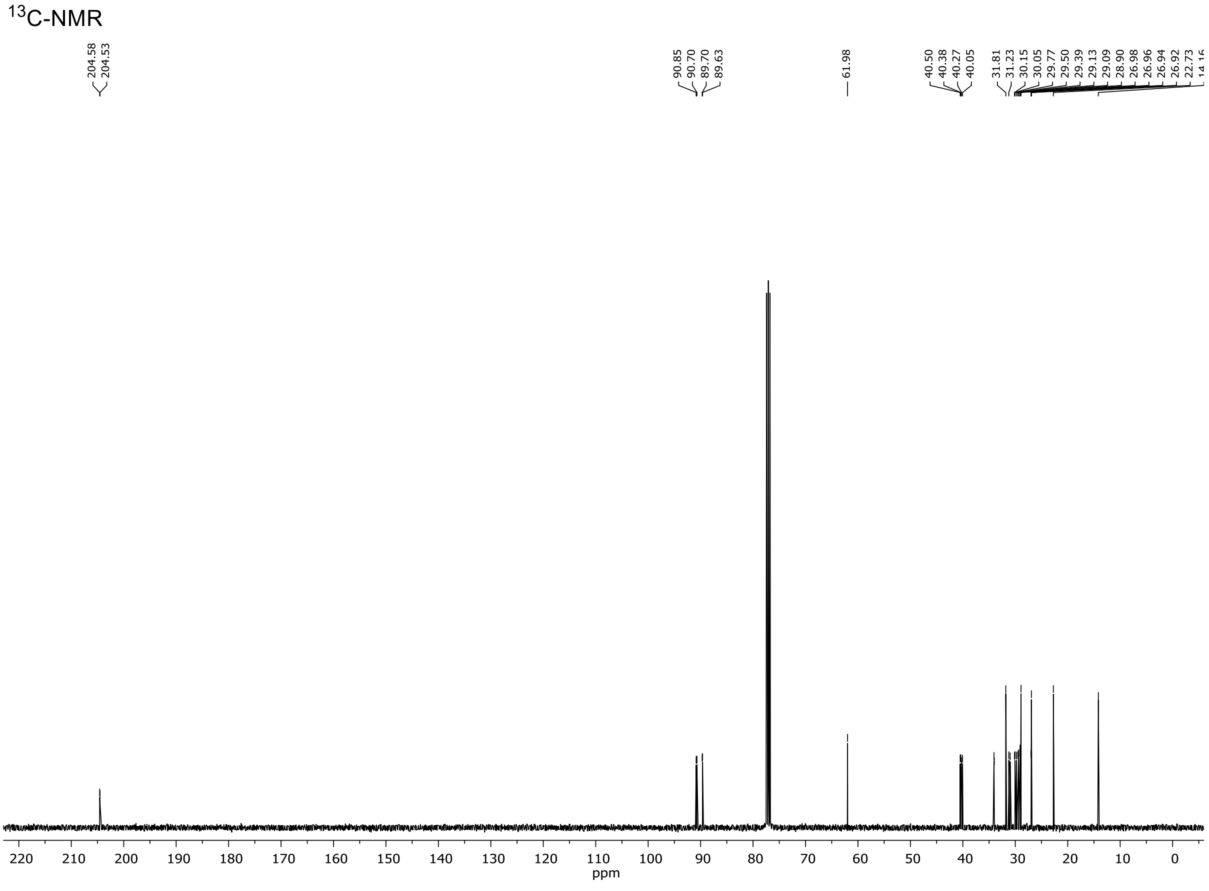

3-(3-phenylpropyl)trideca-5,6-dien-1-ol 104

^{1}H-NMR

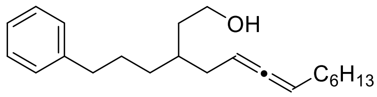

$C_{22}H_{34}O$
314.51

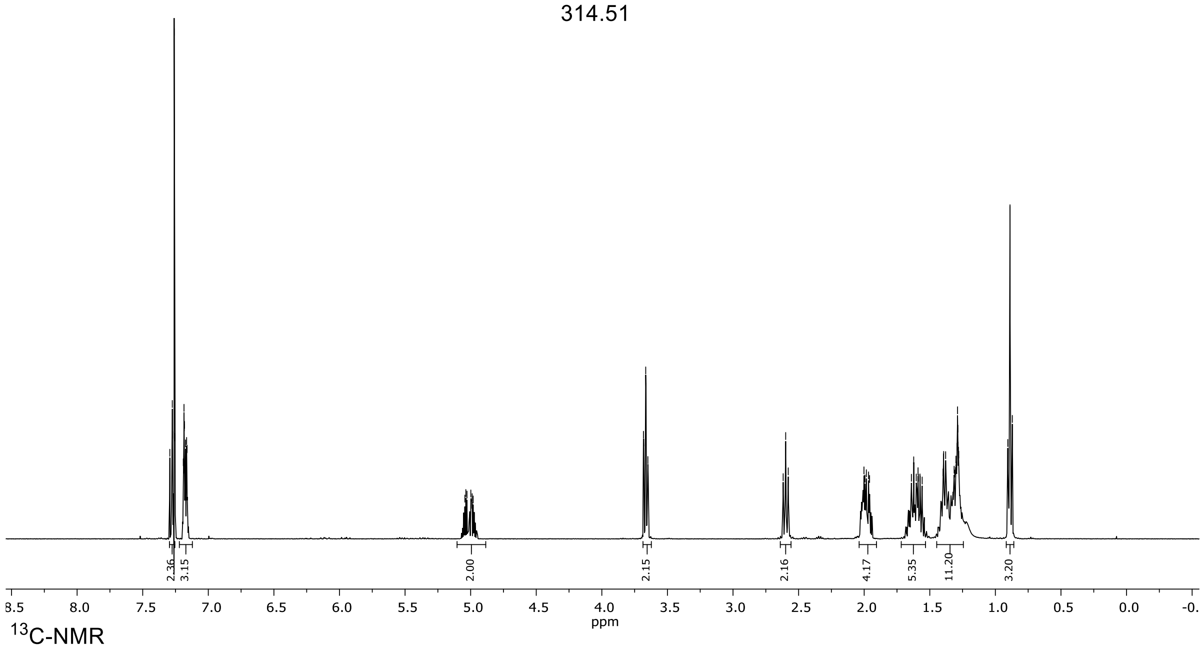

^{13}C-NMR

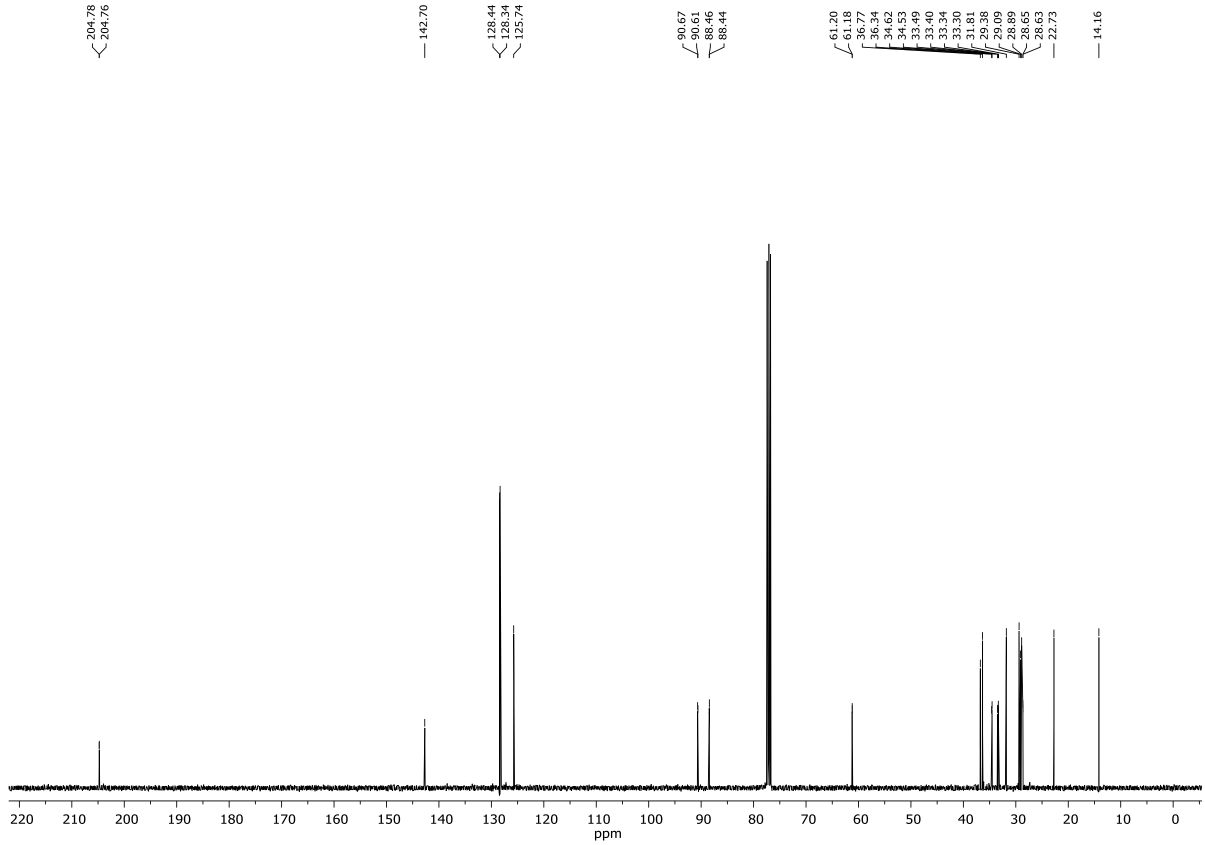

3-phenyltrideca-5,6-dien-1-ol 105

^{1}H-NMR

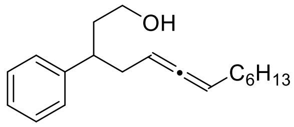

$C_{19}H_{28}O$
272.43

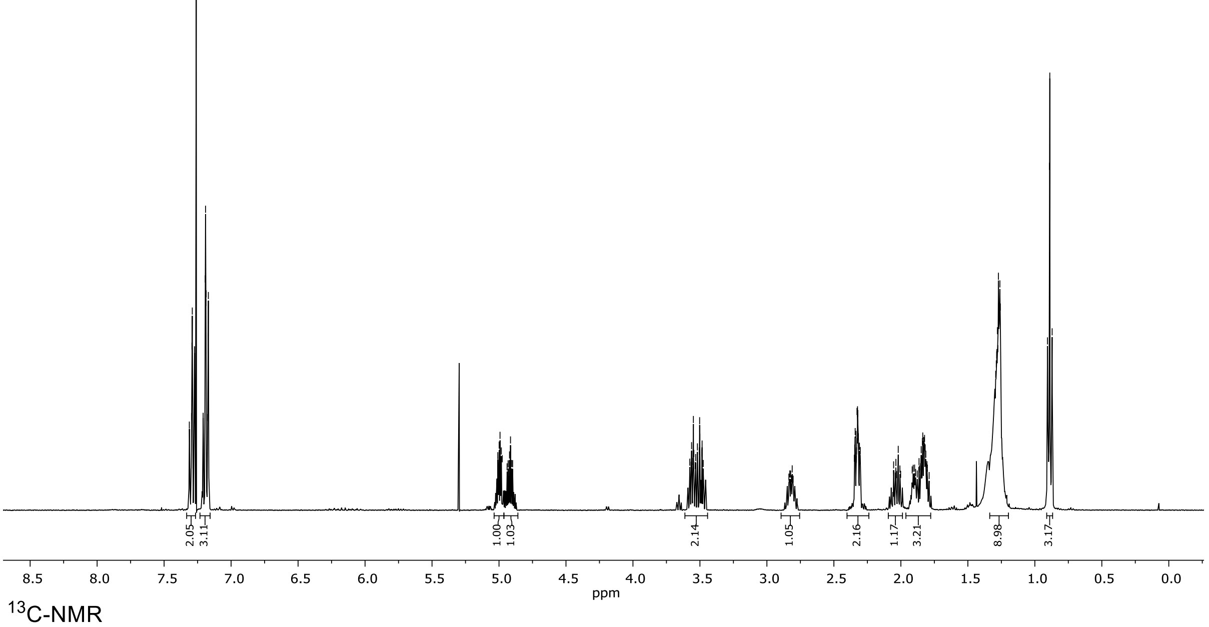

^{13}C-NMR

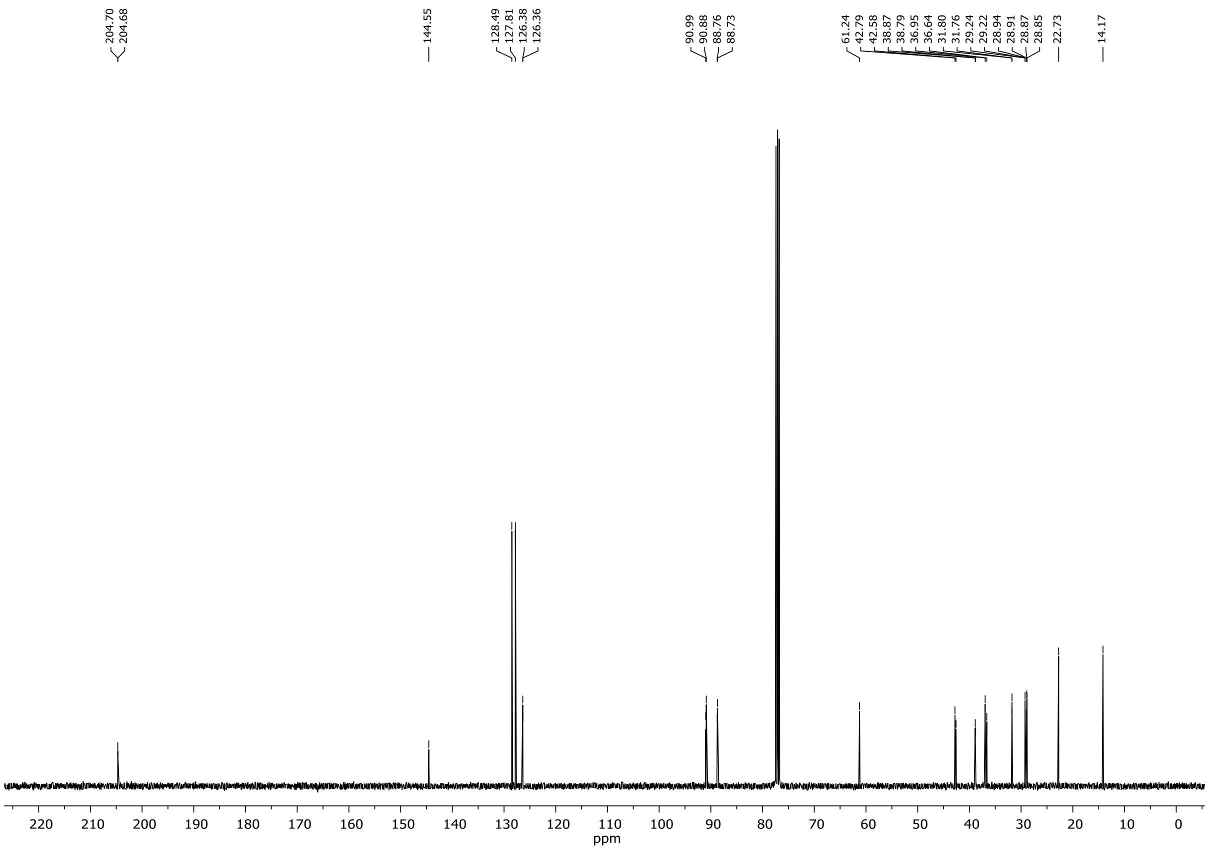

3-(naphthalen-2-yl)trideca-5,6-dien-1-ol 106

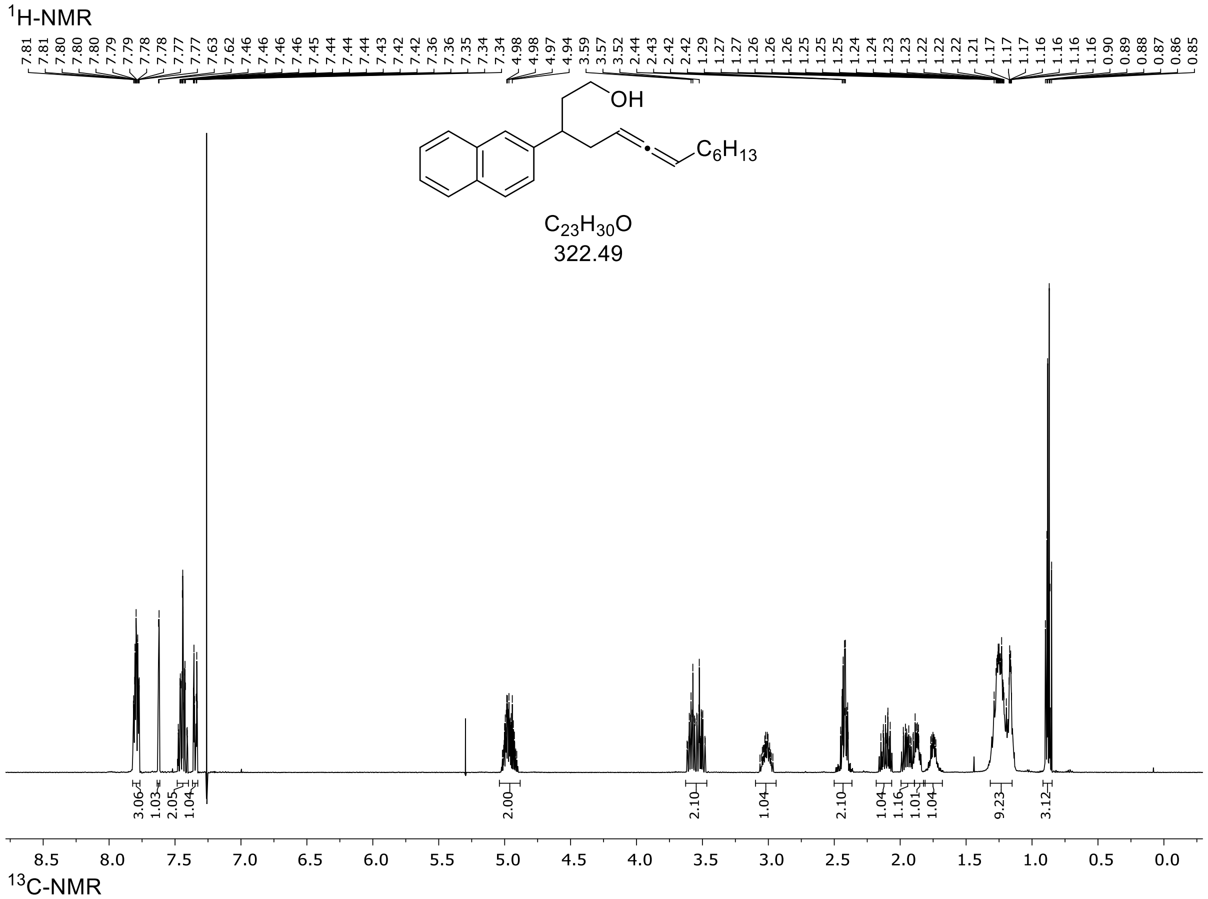

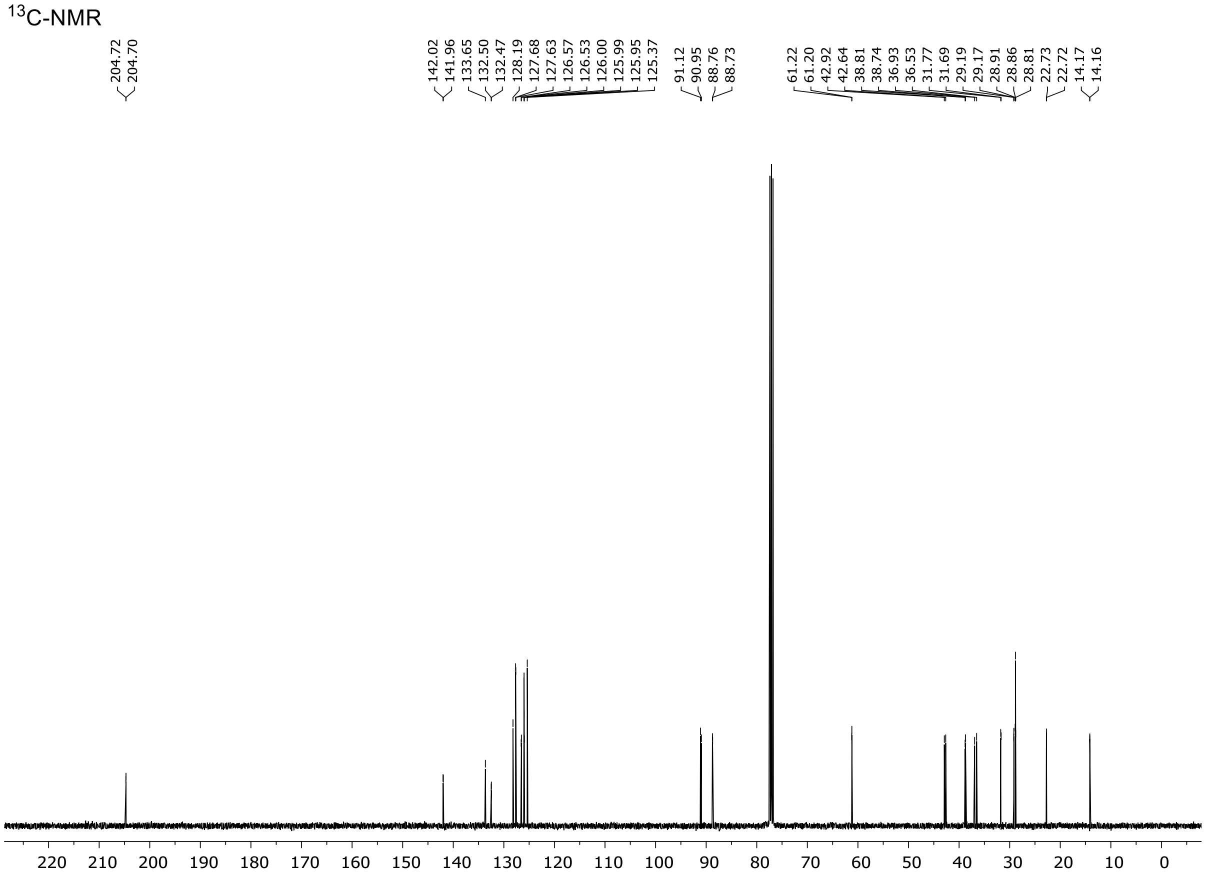

3-([1,1'-biphenyl]-4-yl)trideca-5,6-dien-1-ol 107

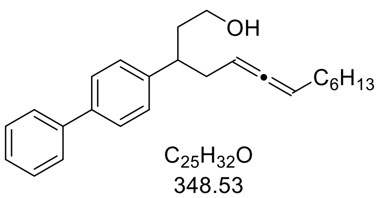

^{1}H-NMR

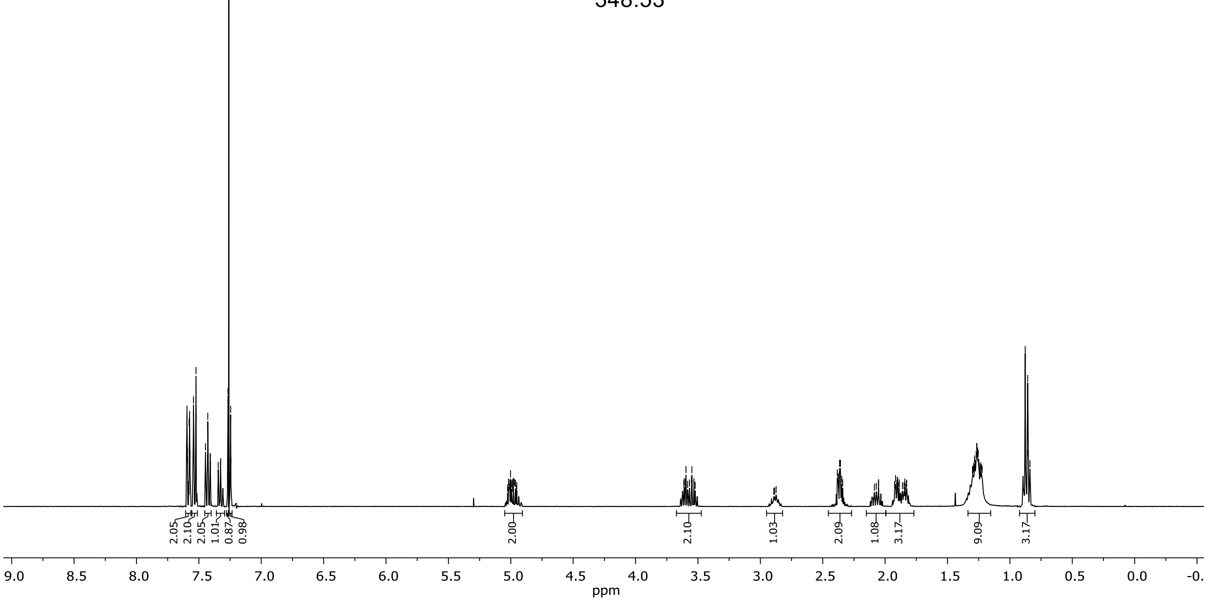

^{13}C-NMR

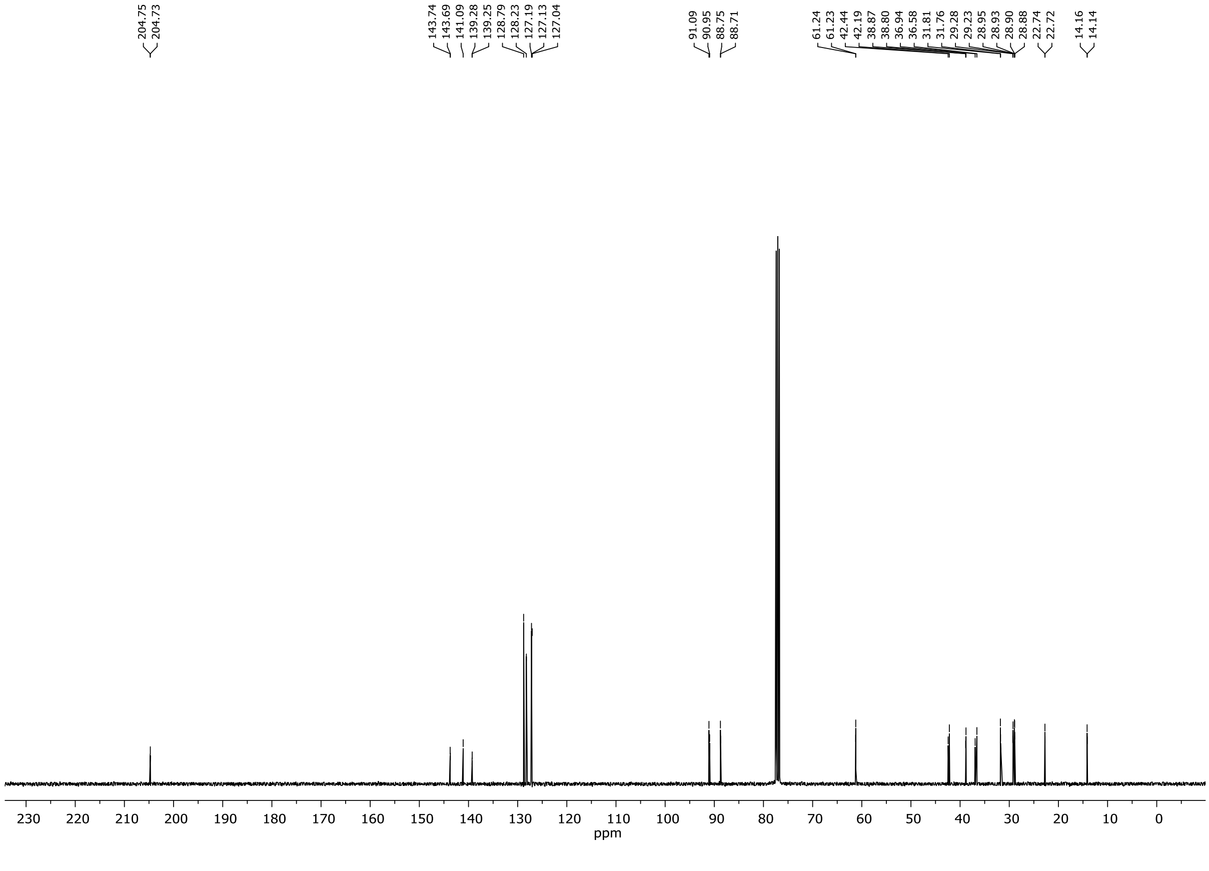

3-(p-tolyl)trideca-5,6-dien-1-ol 108

^{1}H-NMR

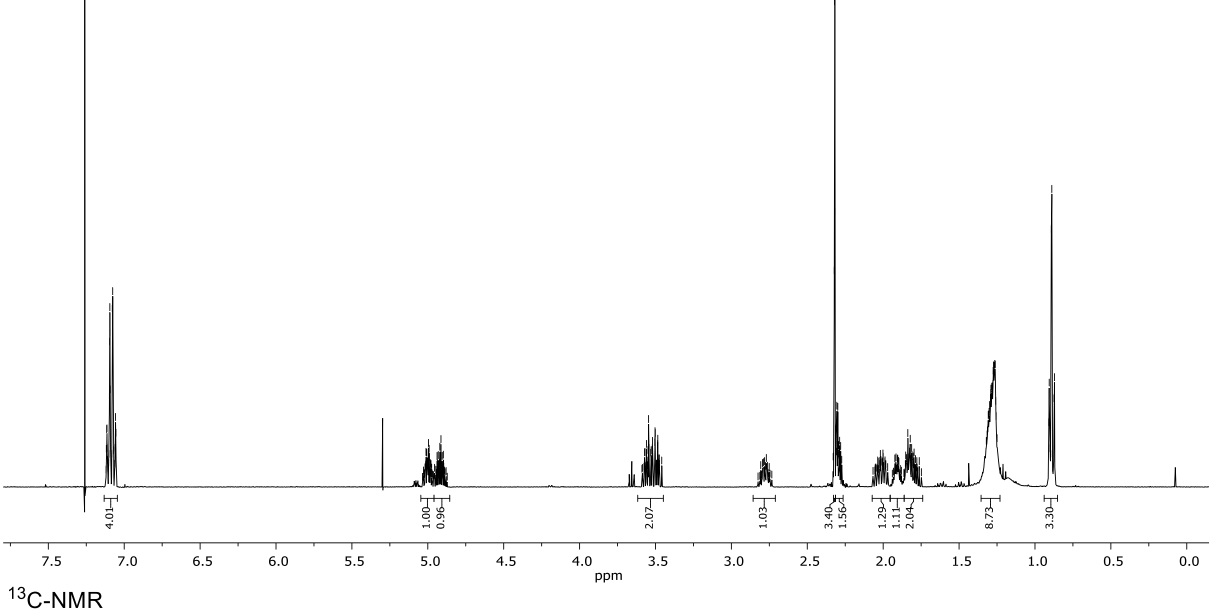

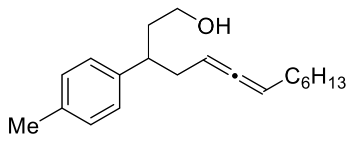

$C_{20}H_{30}O$
286.46

^{13}C-NMR

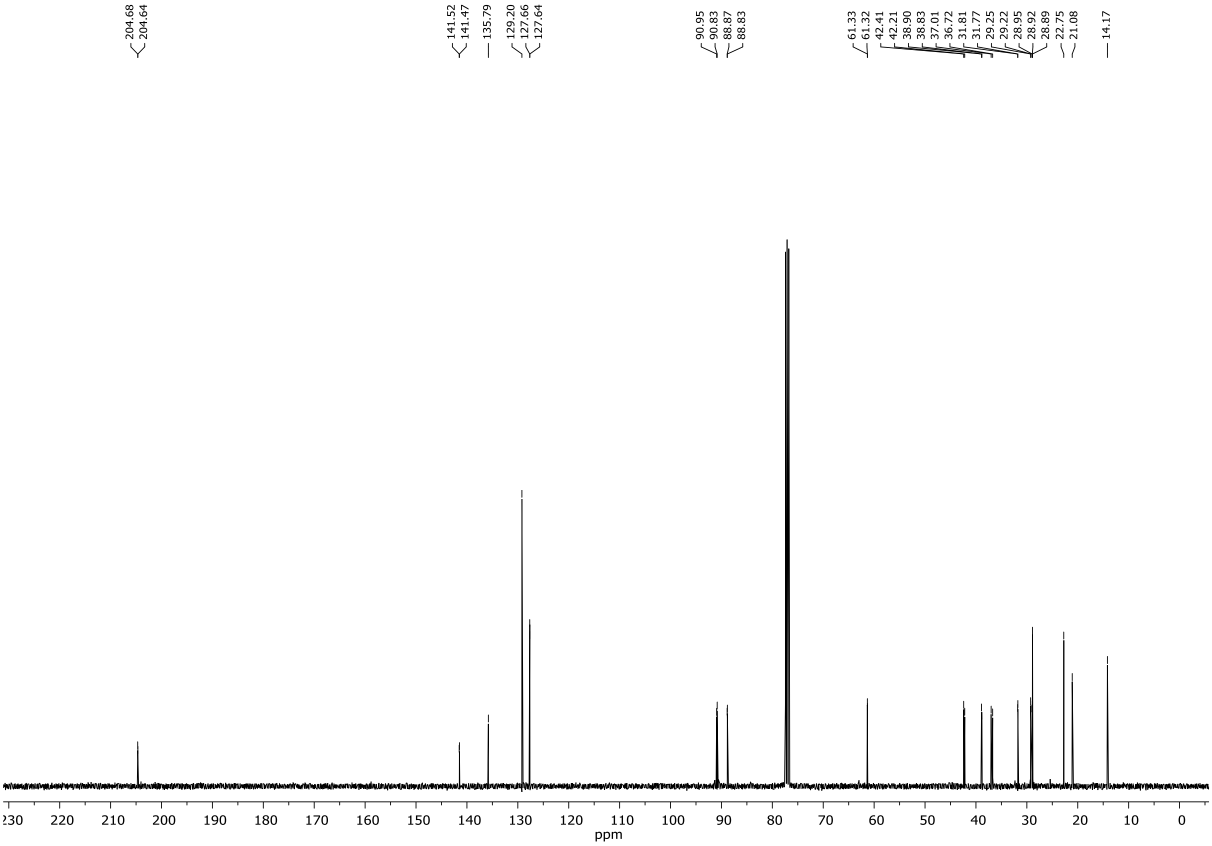

3-(m-tolyl)trideca-5,6-dien-1-ol 109

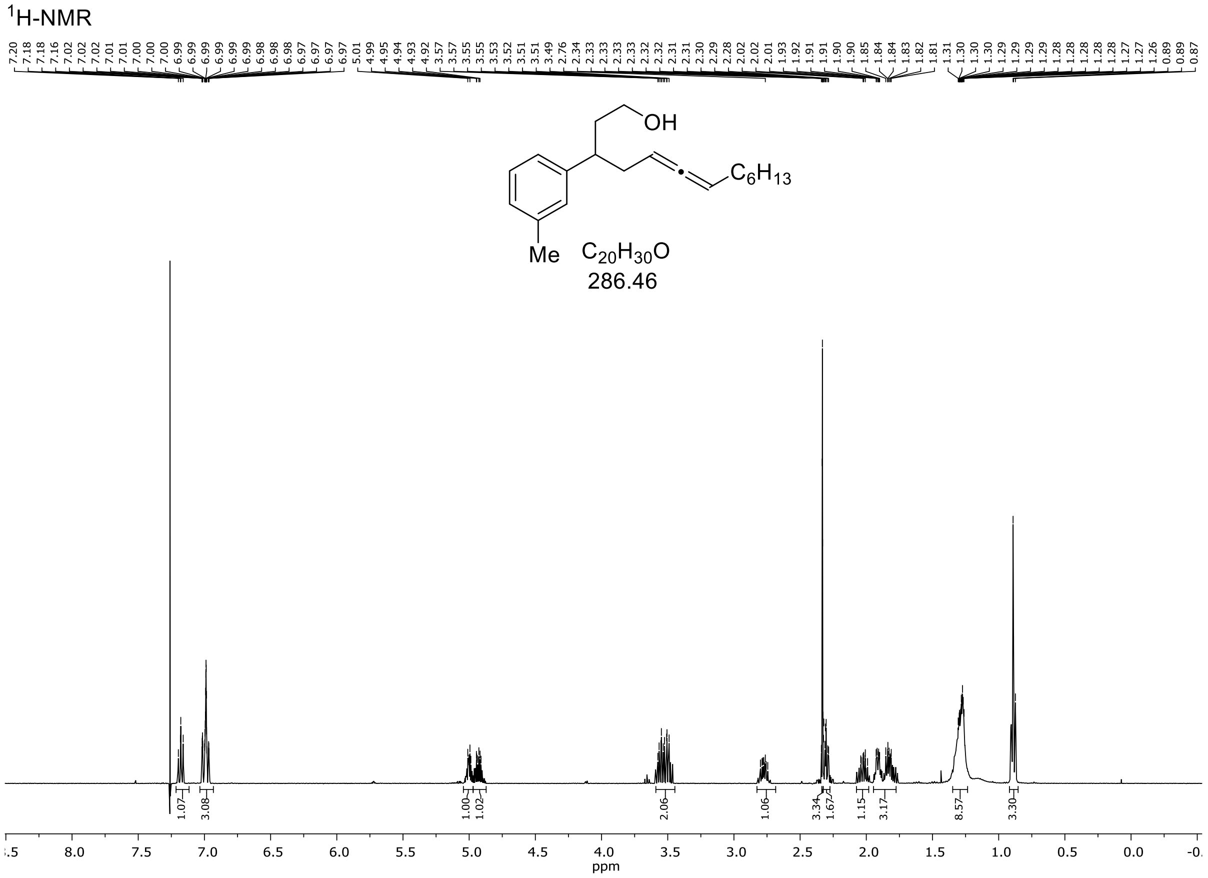

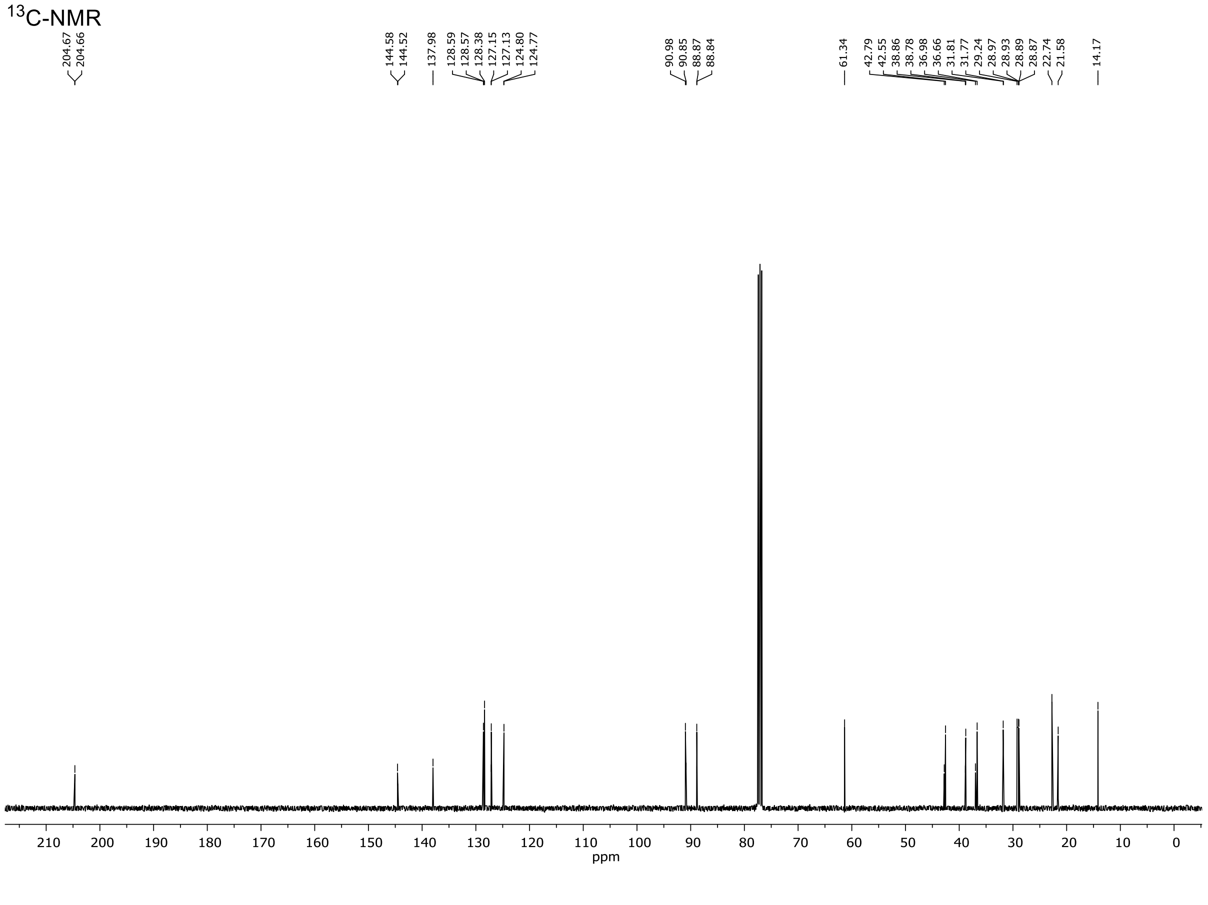

3-(o-tolyl)trideca-5,6-dien-1-ol 110

^{1}H-NMR

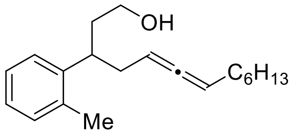

$C_{20}H_{30}O$
286.46

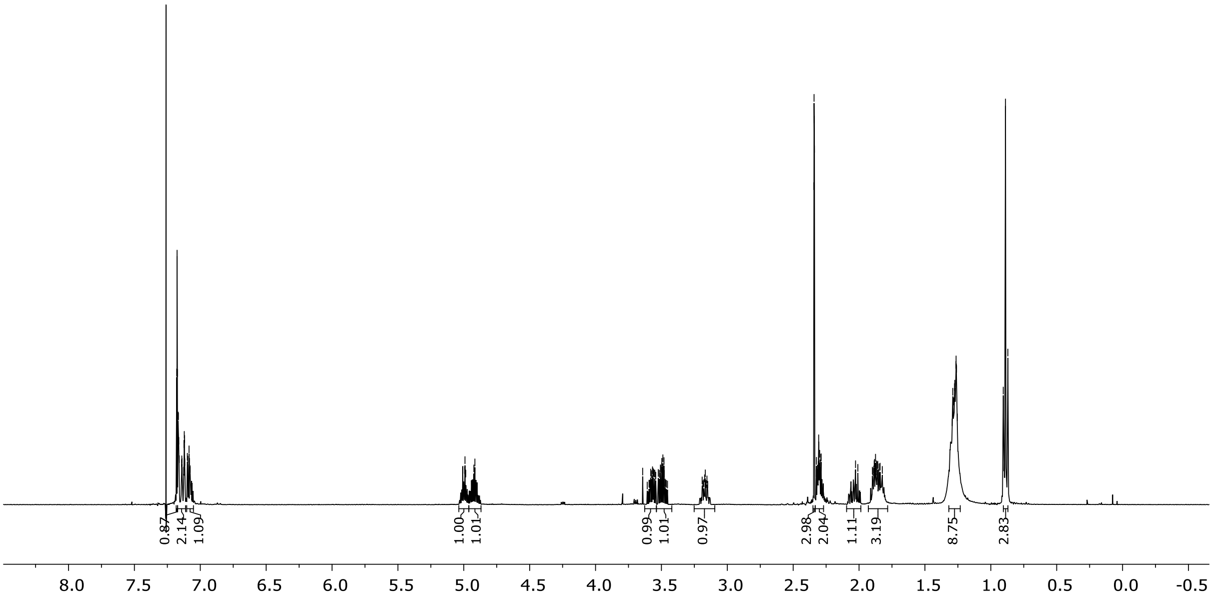

^{13}C-NMR

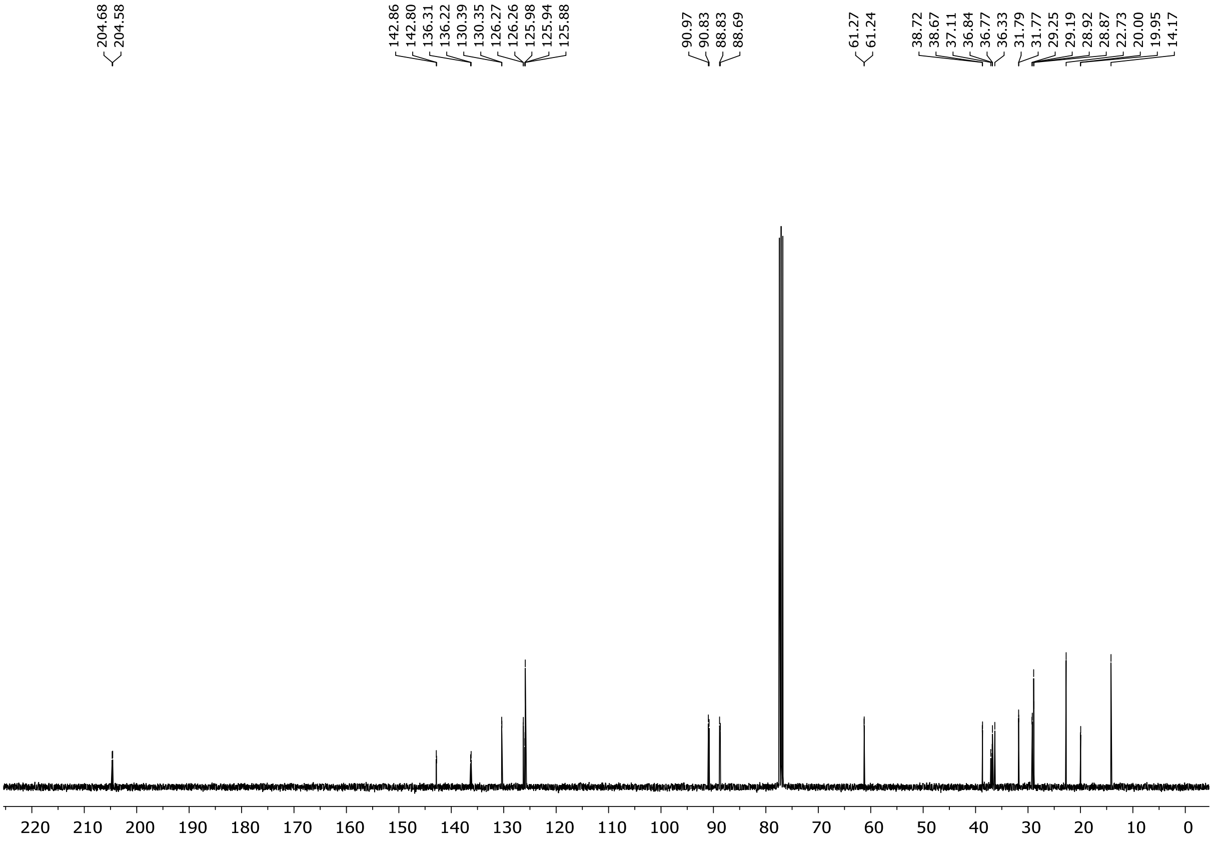

3-mesityltrideca-5,6-dien-1-ol 111

^{1}H-NMR

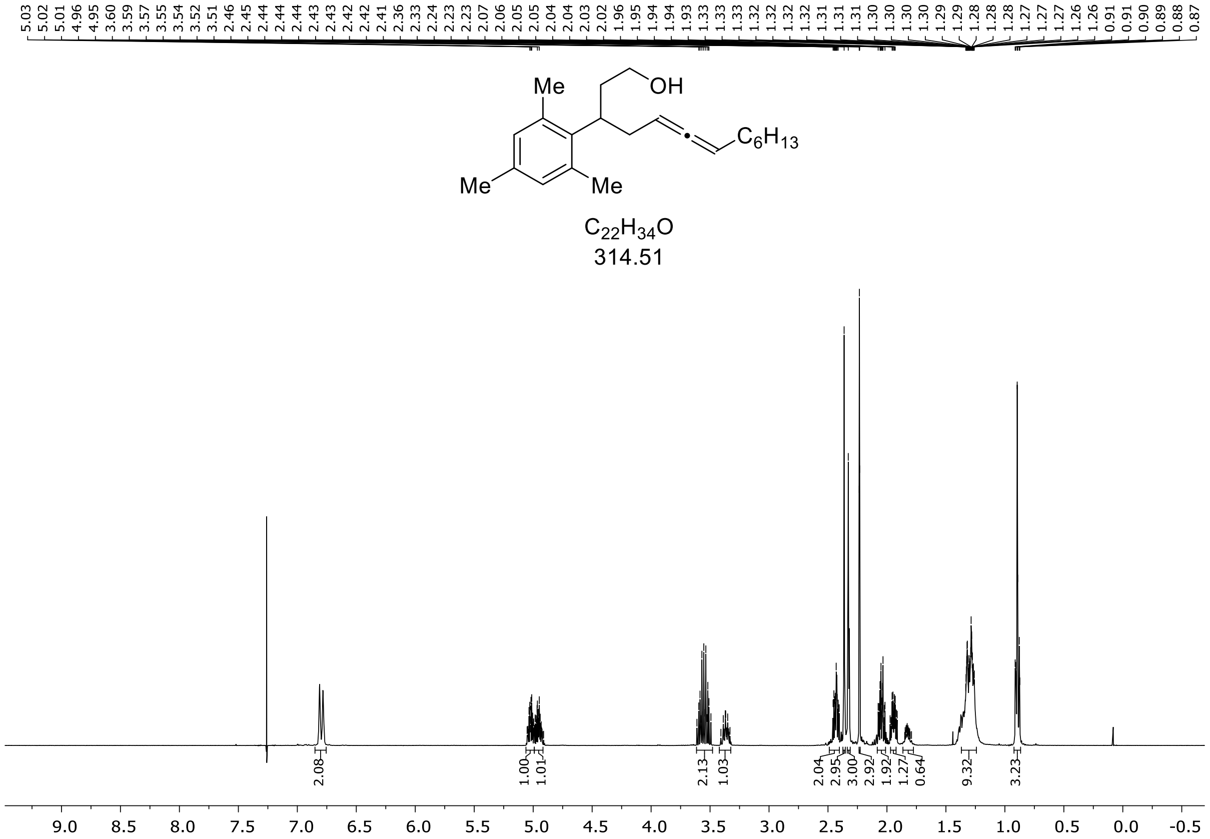

^{13}C-NMR

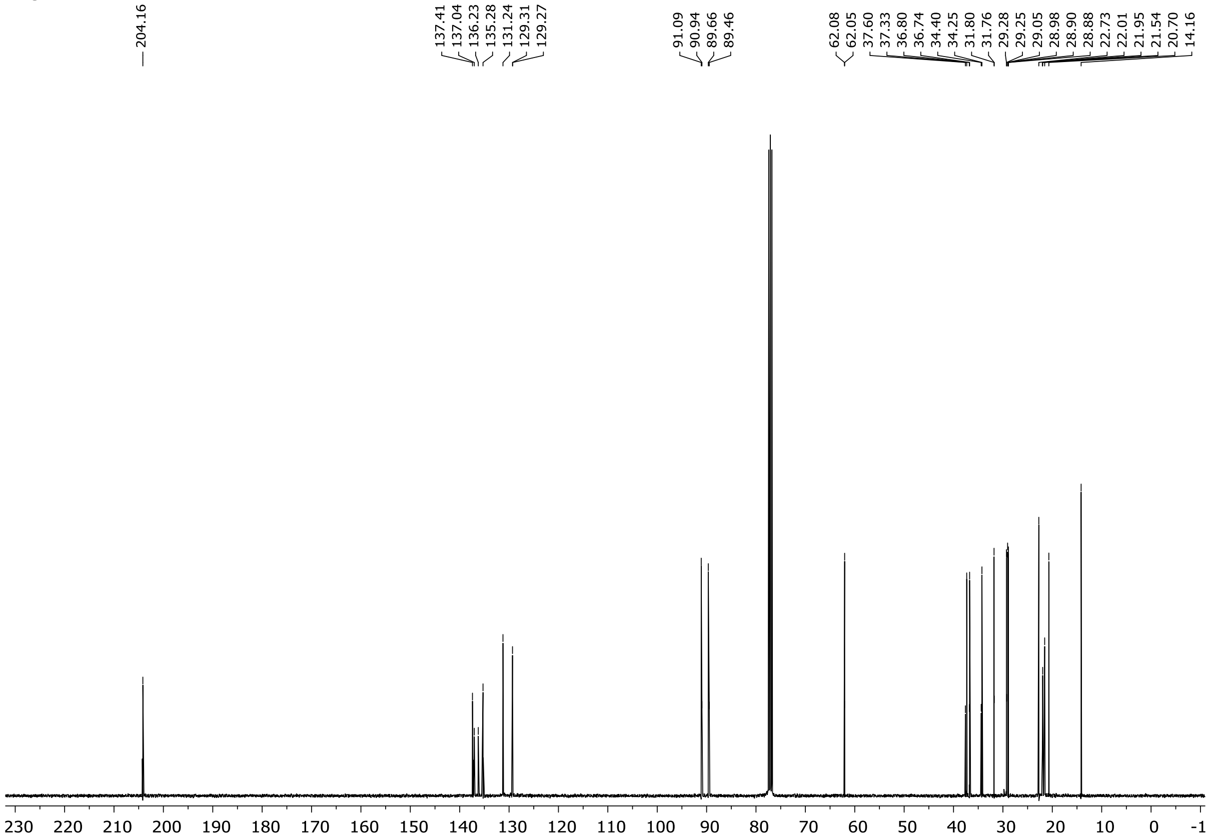

3-(4-vinylphenyl)trideca-5,6-dien-1-ol 112

^{1}H-NMR

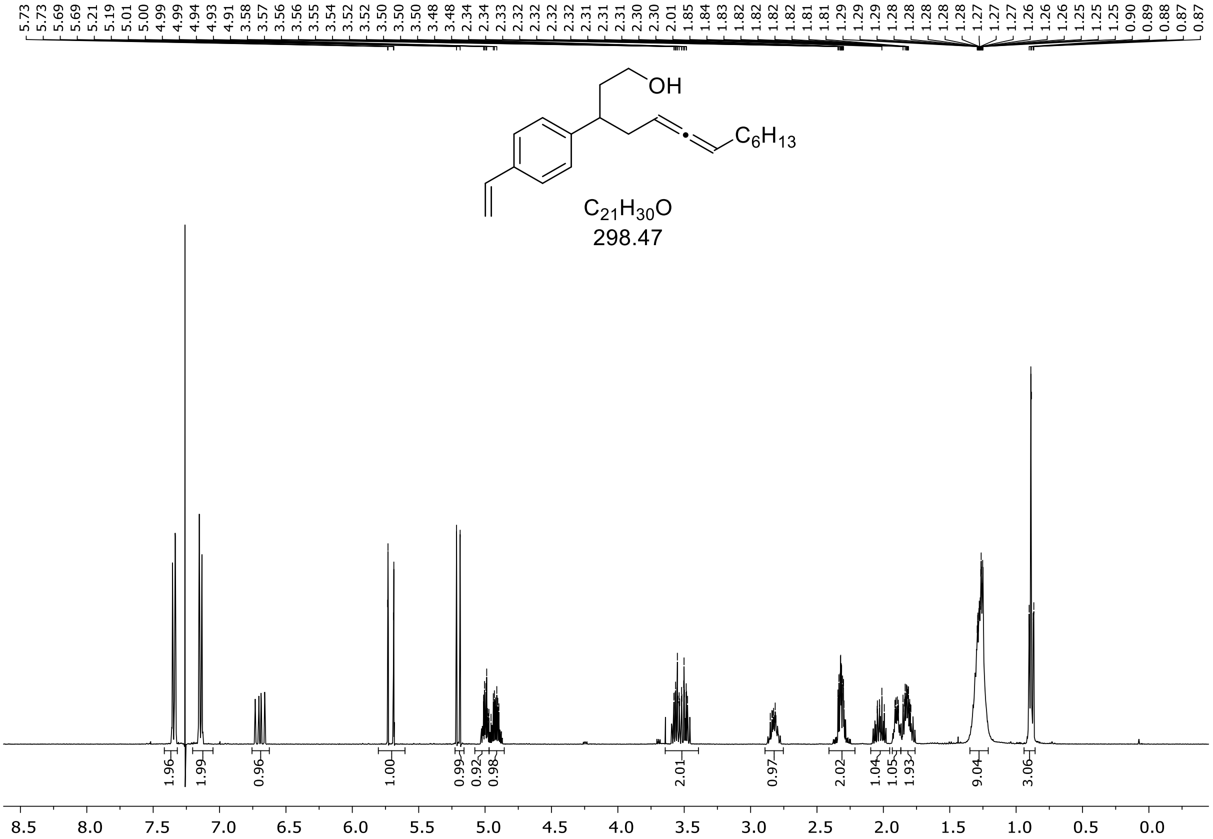

$C_{21}H_{30}O$
298.47

^{13}C-NMR

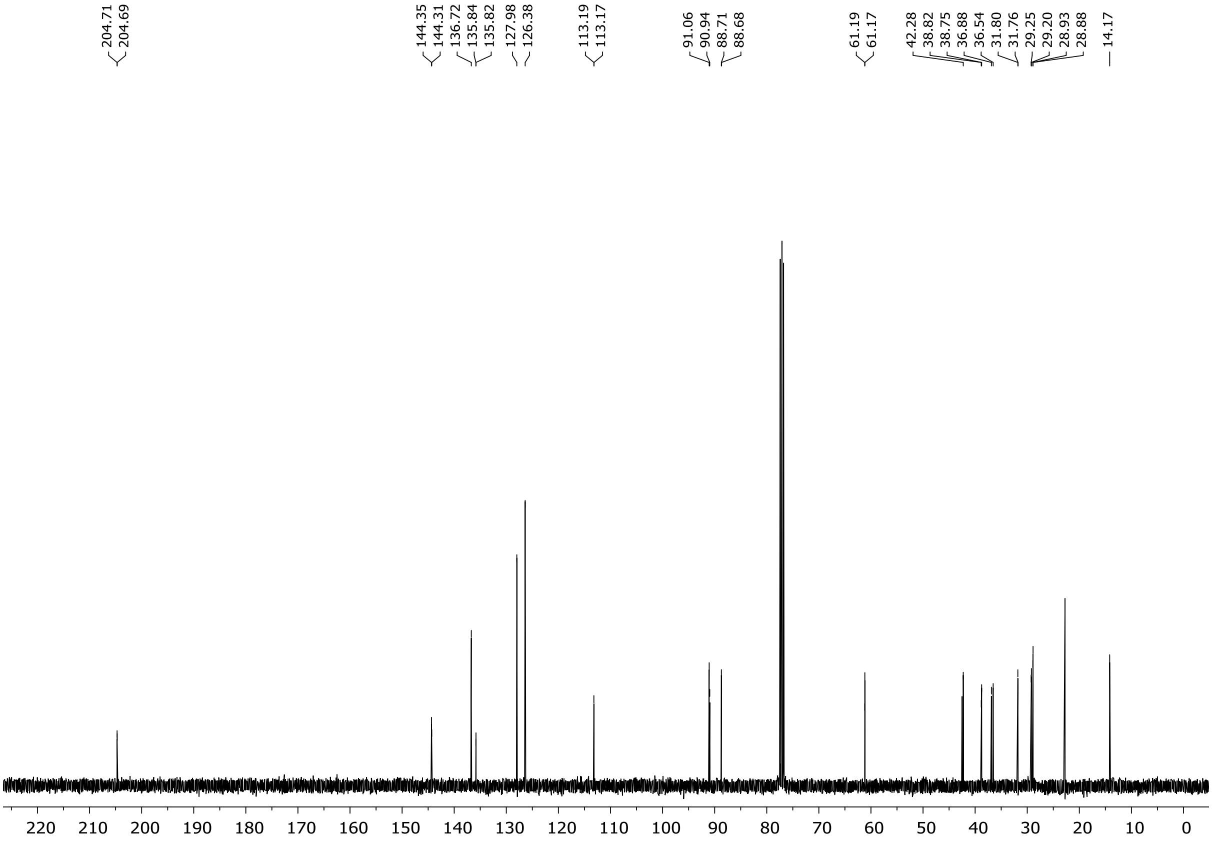

3-(4-bromophenyl)trideca-5,6-dien-1-ol 113

^{1}H-NMR

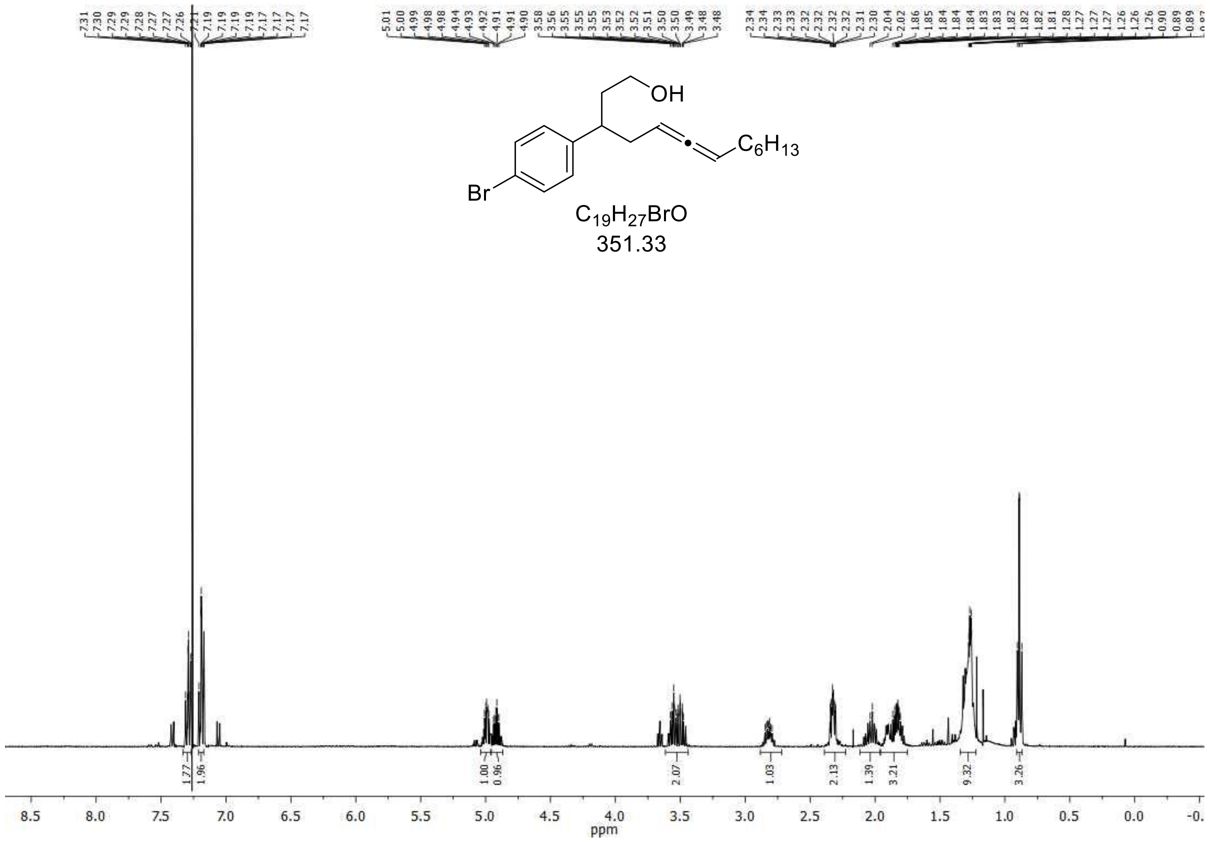

^{13}C-NMR

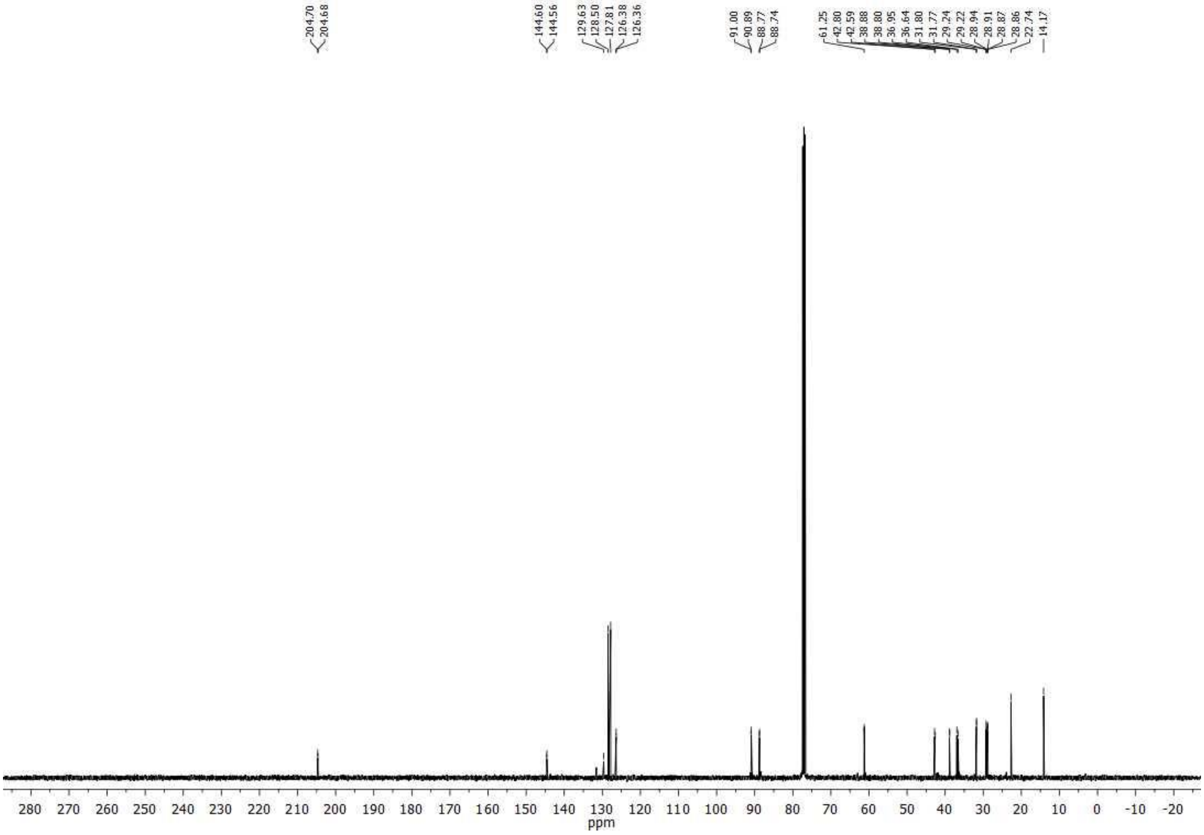

3-(4-methoxyphenyl)trideca-5,6-dien-1-ol 114

^{1}H-NMR

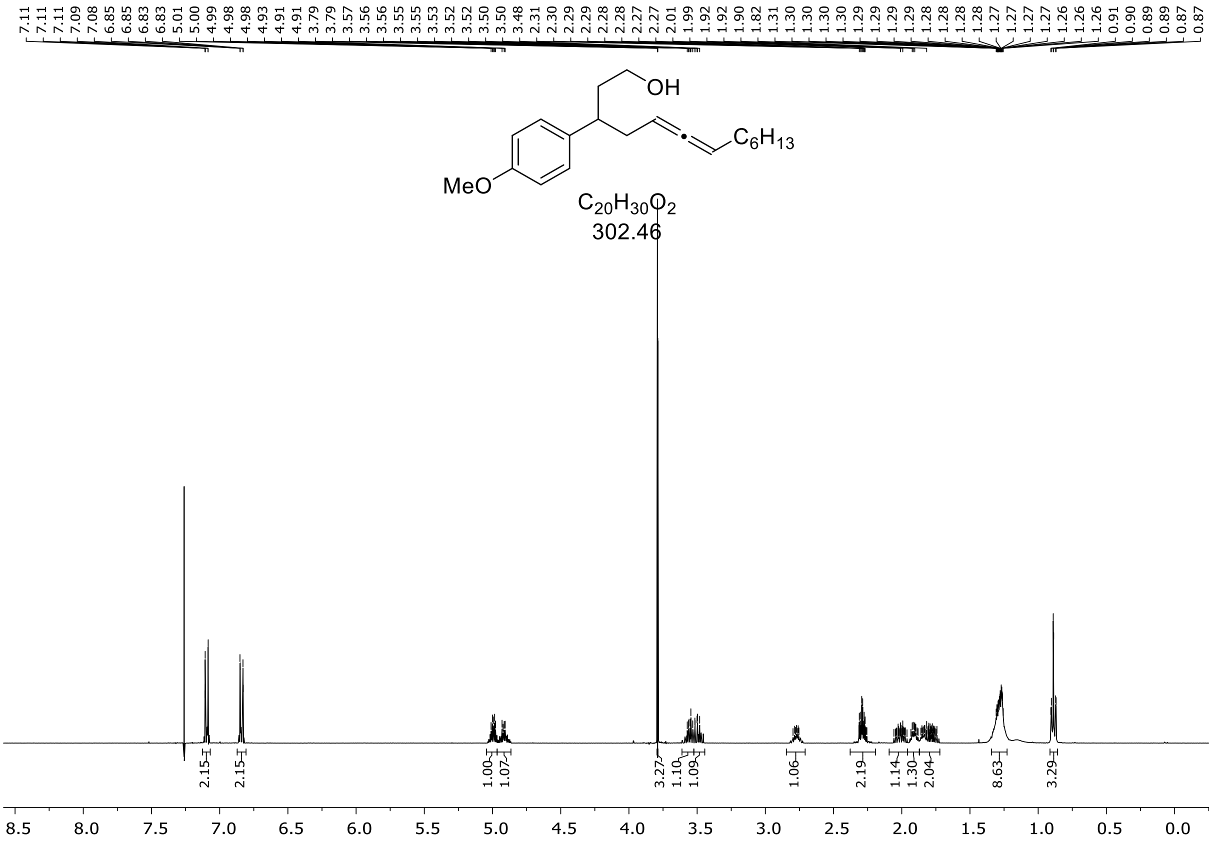

^{13}C-NMR

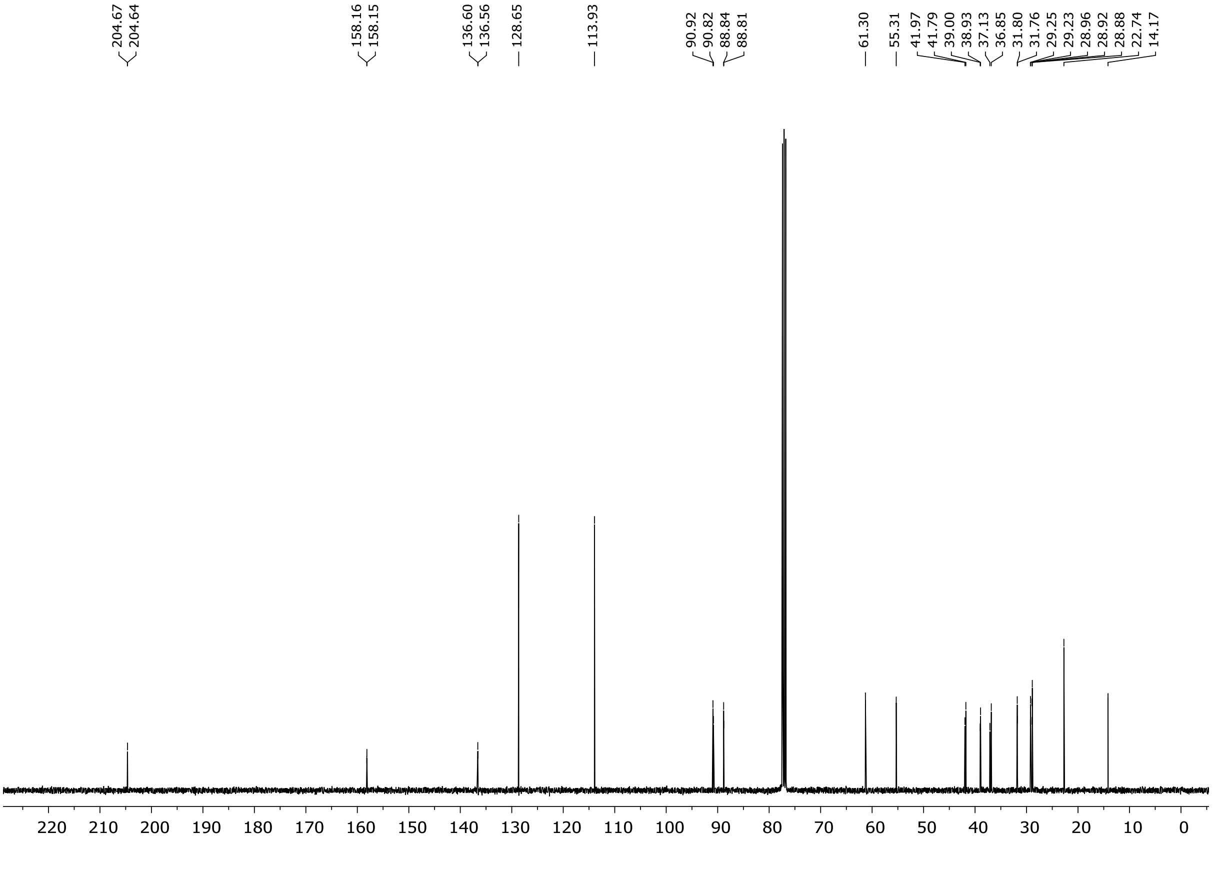

3-(4-(methylthio)phenyl)trideca-5,6-dien-1-ol 115

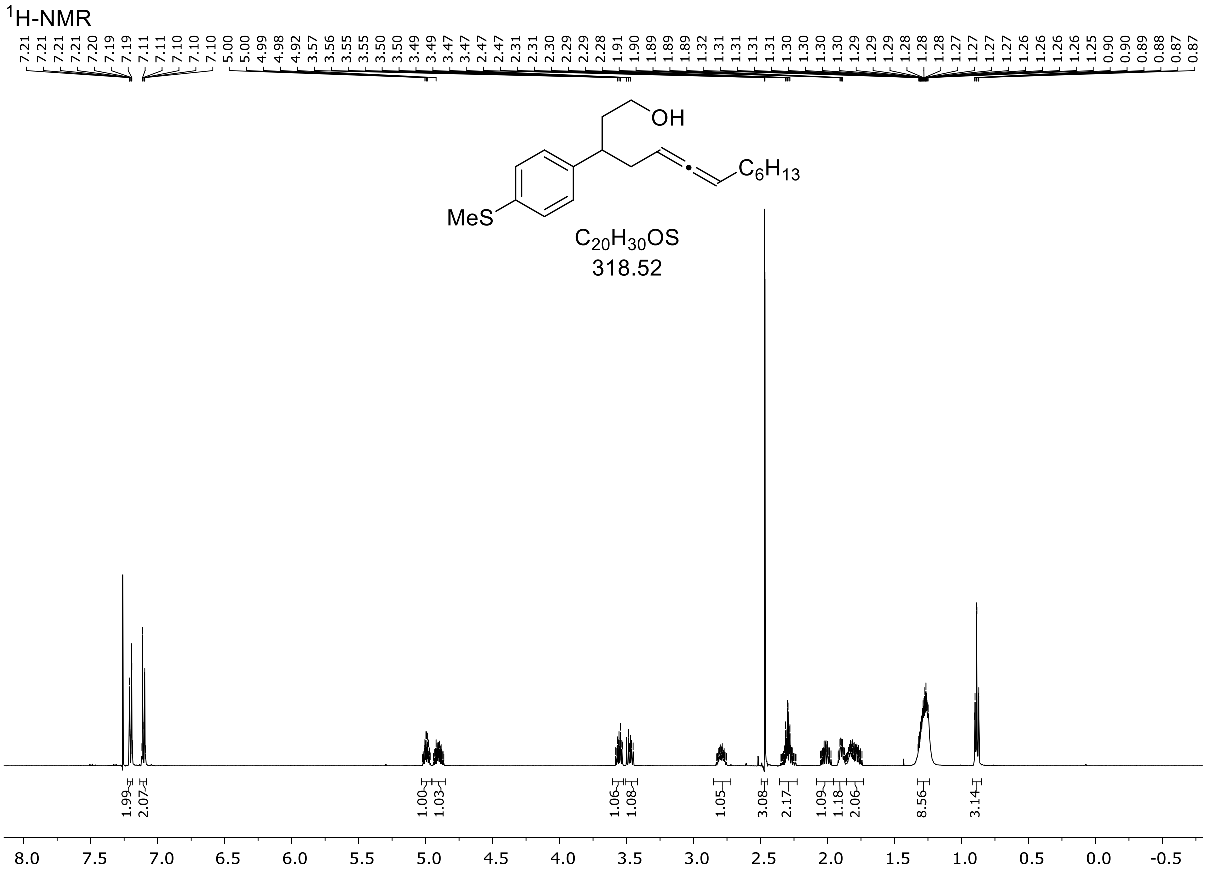

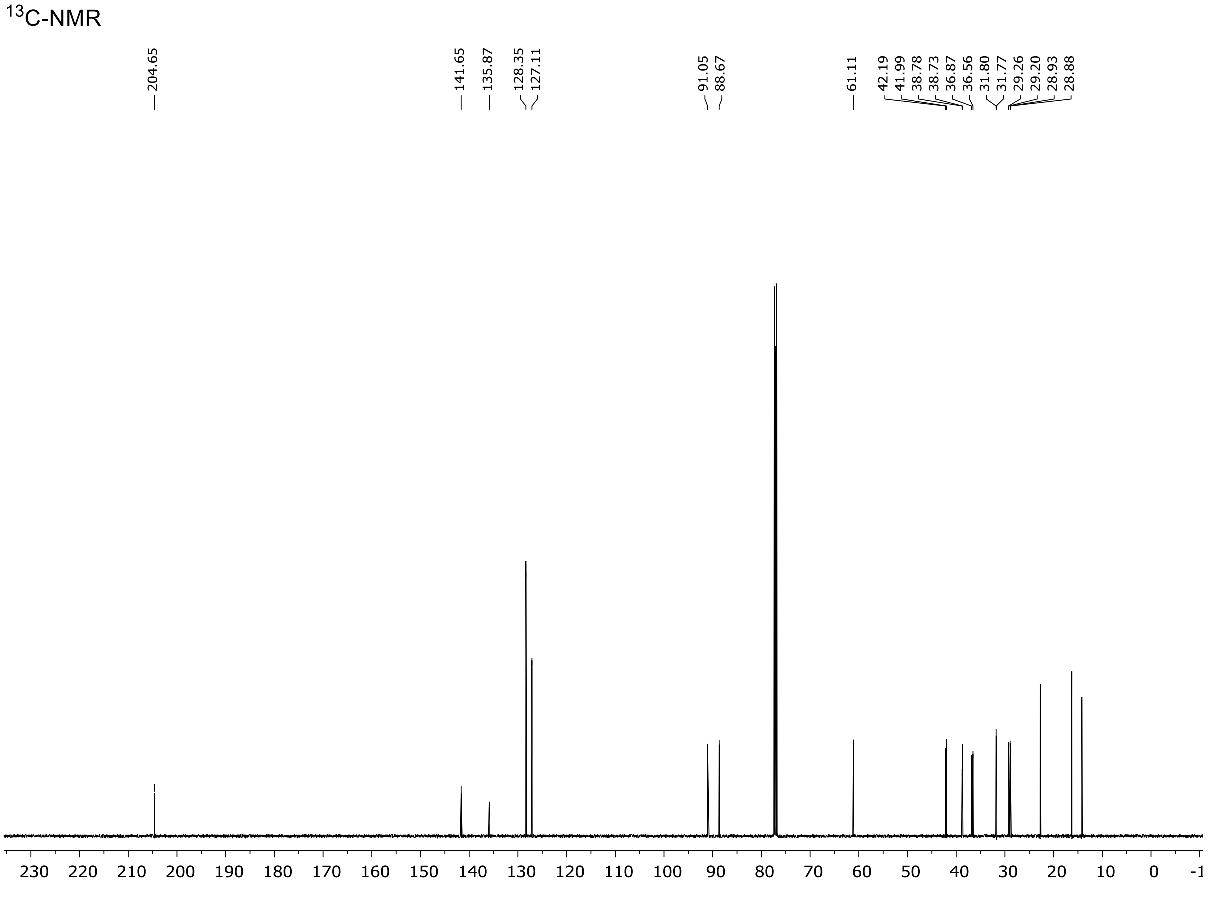

3-(4-(trifluoromethyl)phenyl)trideca-5,6-dien-1-ol 116

^{1}H-NMR

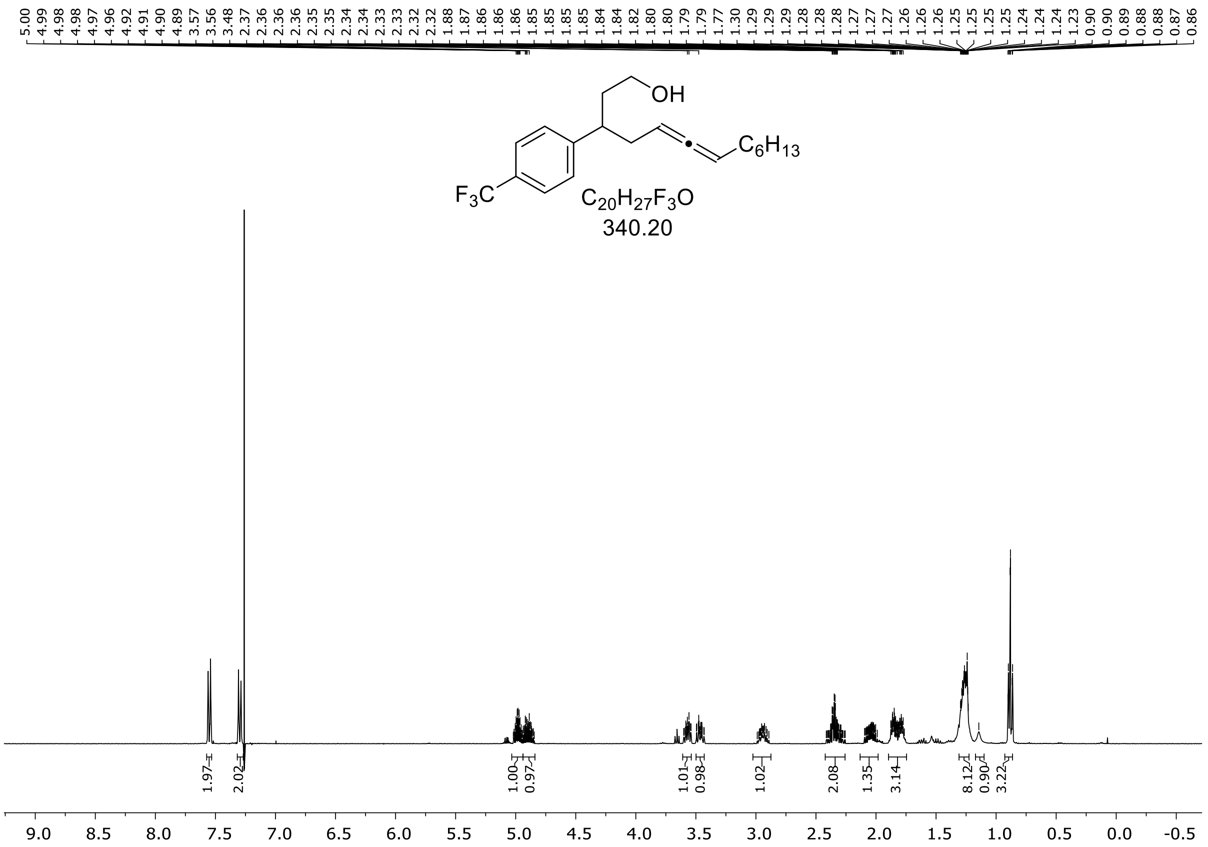

^{13}C-NMR

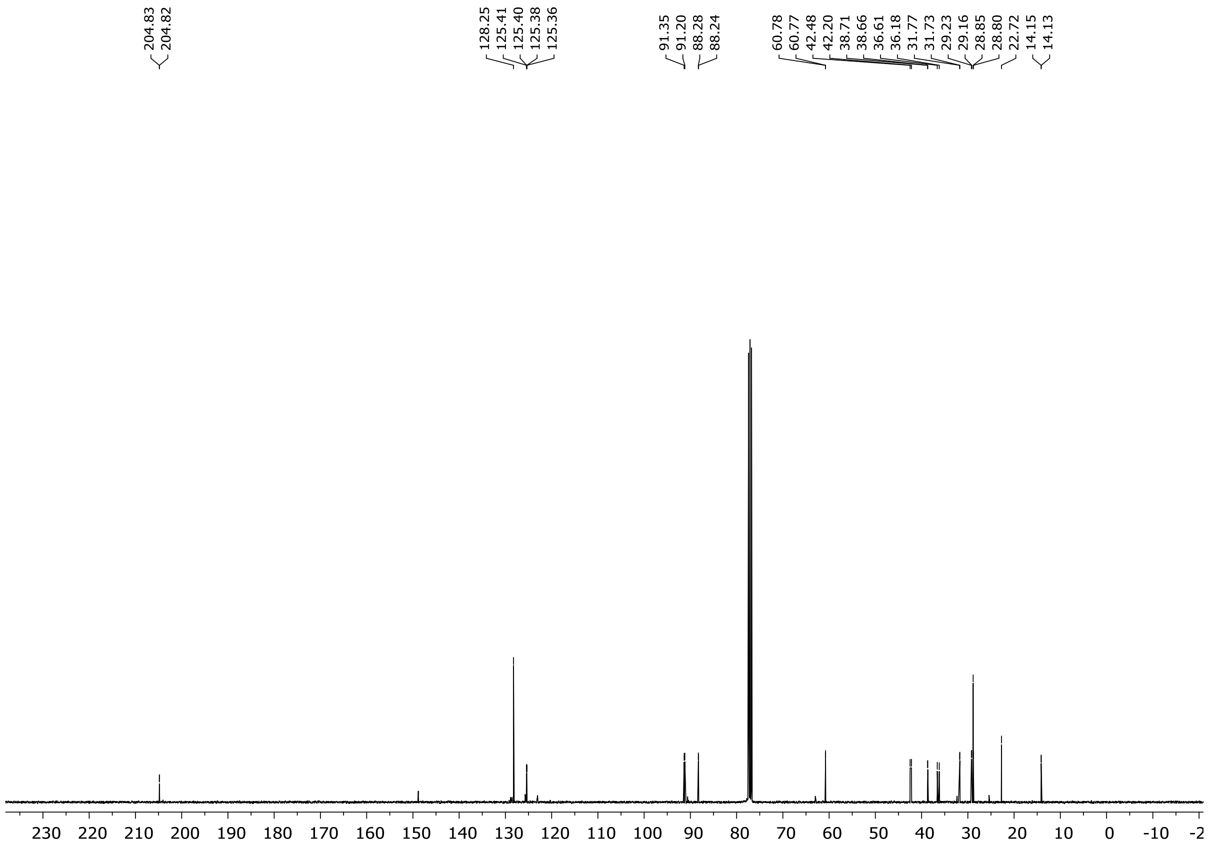

3-(4-(trifluoromethyl)phenyl)trideca-5,6-dien-1-ol 117

^{1}H-NMR

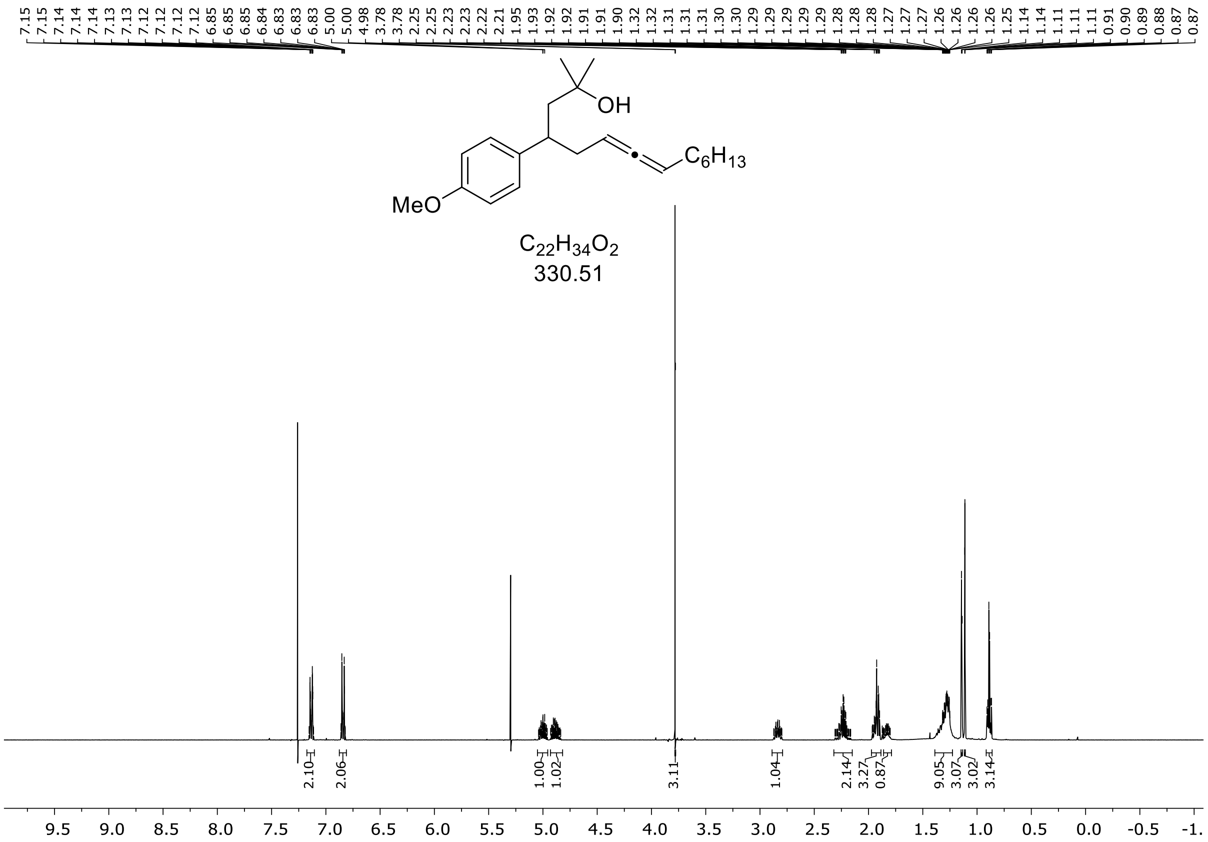

^{13}C-NMR

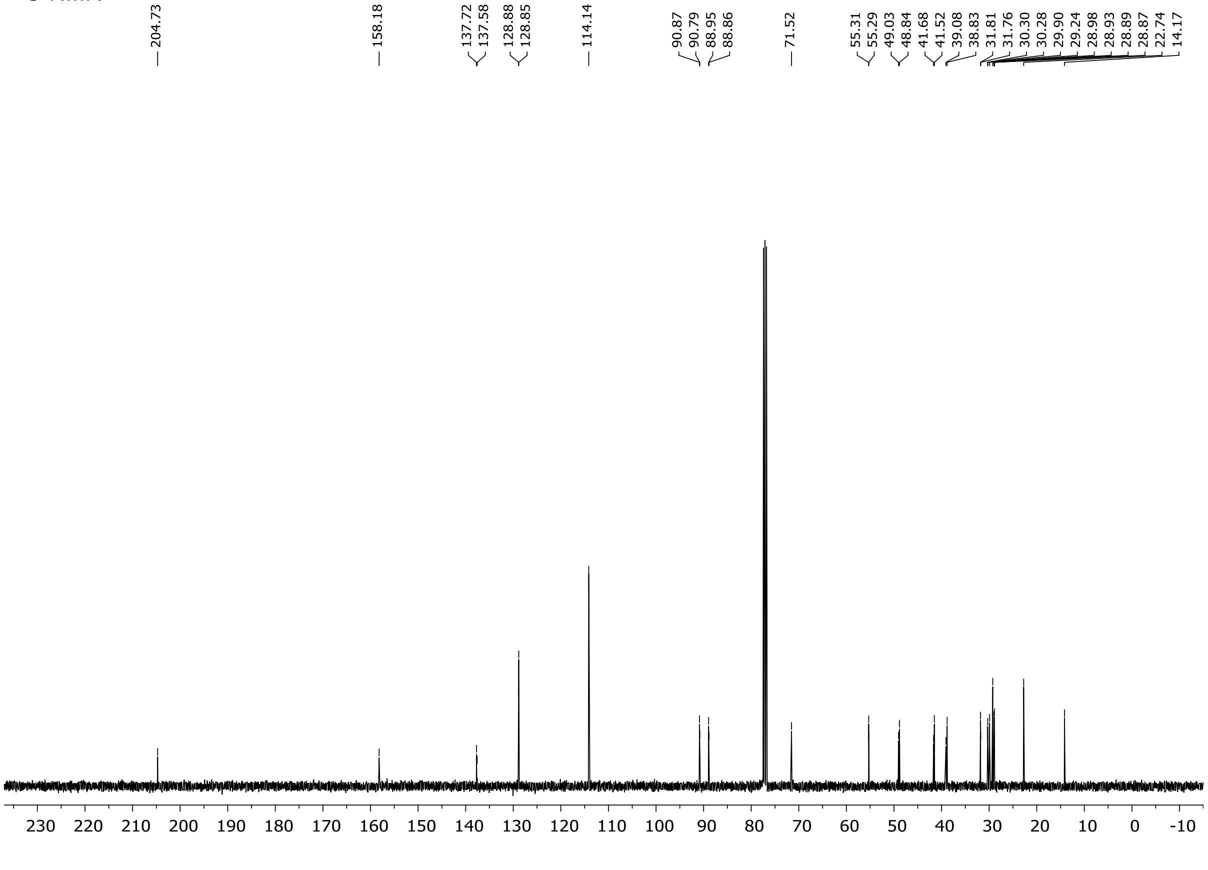

ethyl 5-cyclohexylpenta-3,4-dienoate. 118

^{1}H-NMR

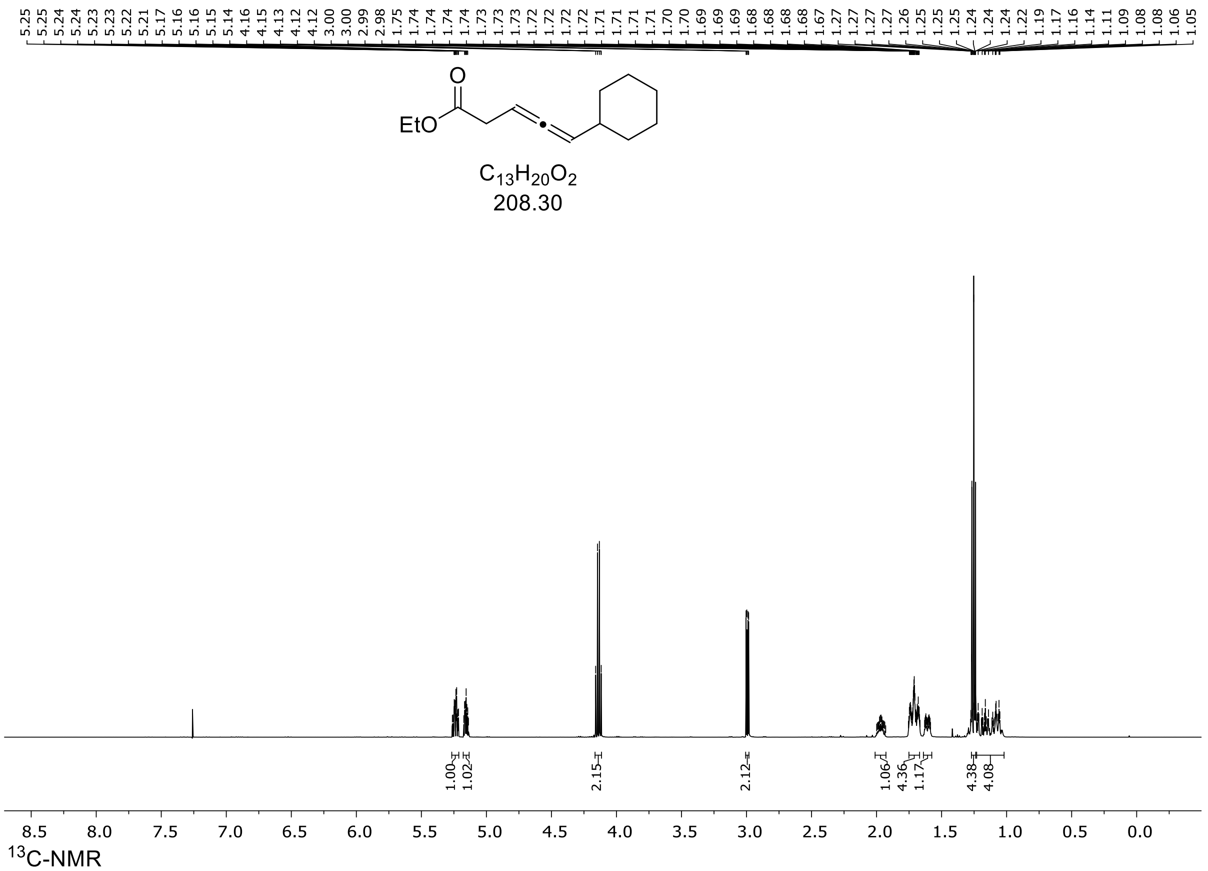

^{13}C-NMR

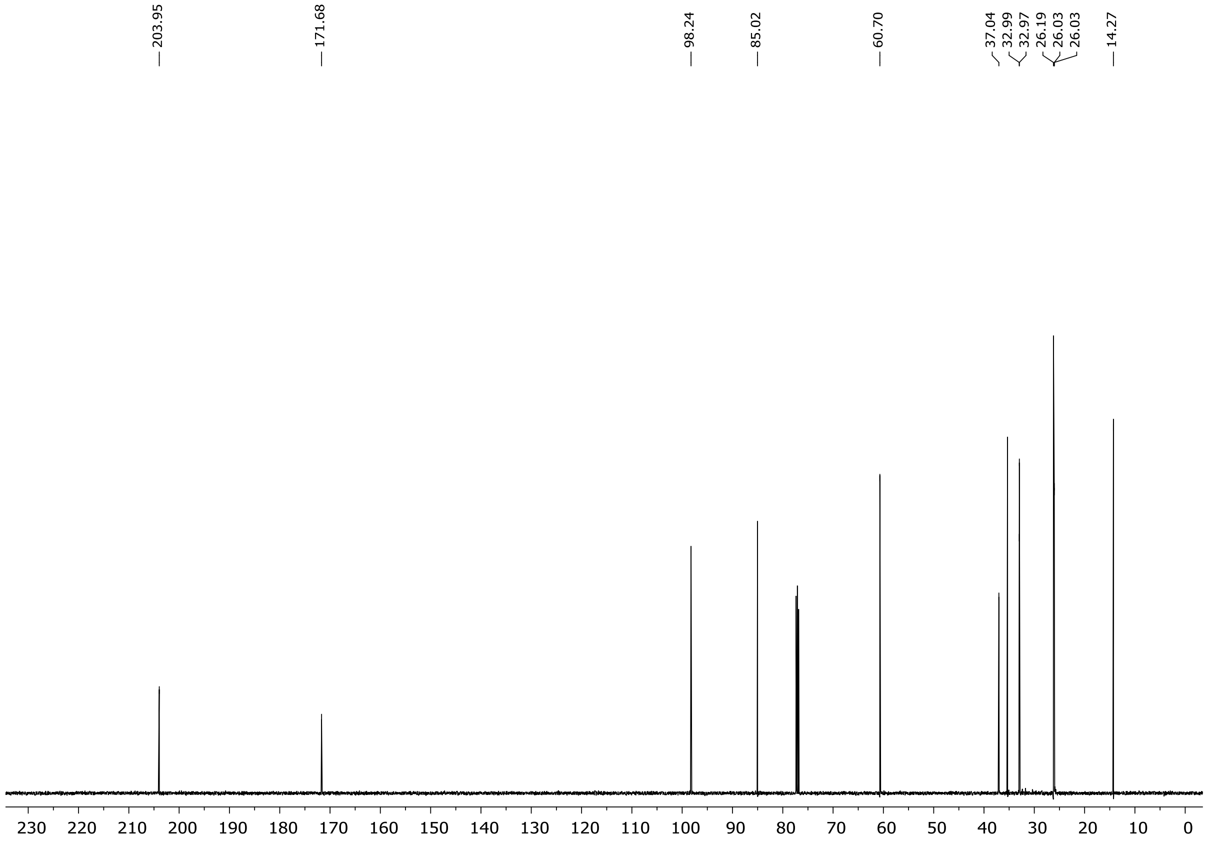

ethyl (E)-7-cyclohexylhepta-2,5,6-trienoat 119

^{1}H-NMR

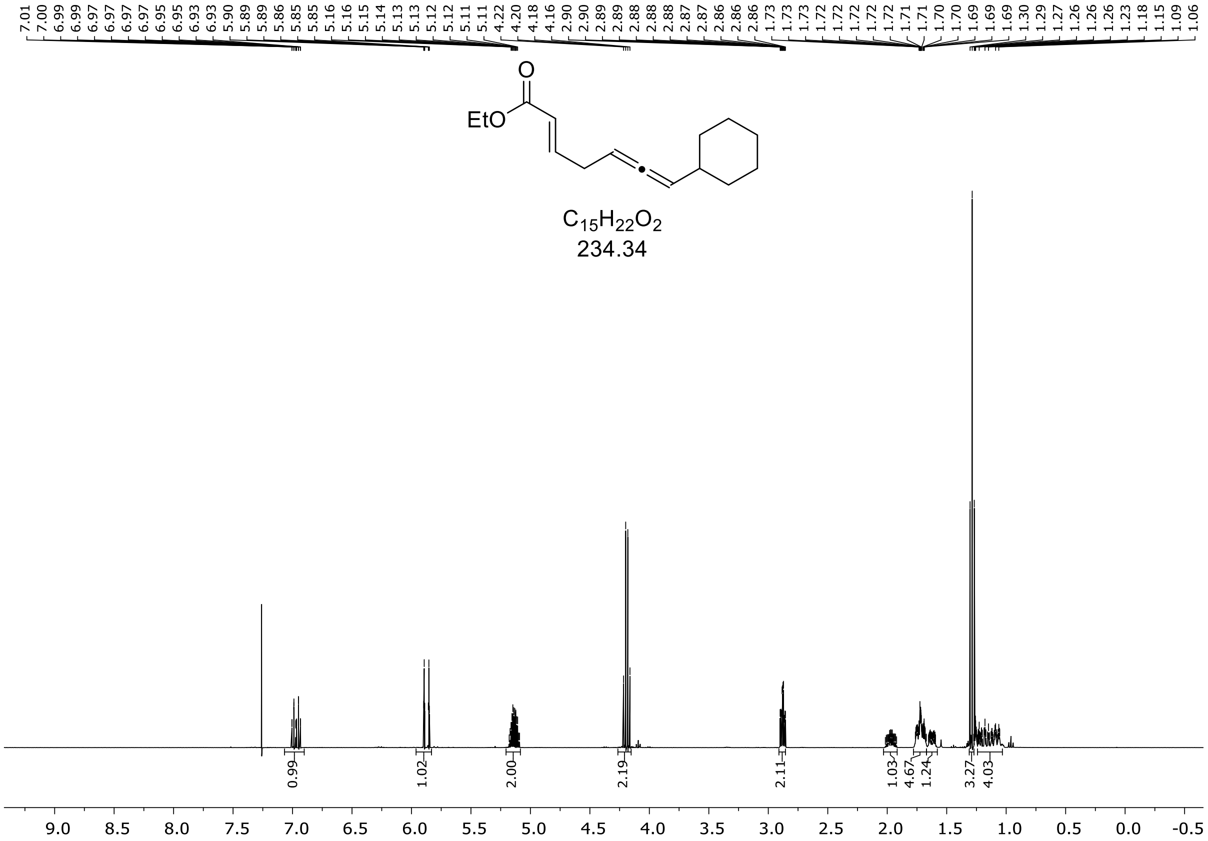

^{13}C-NMR

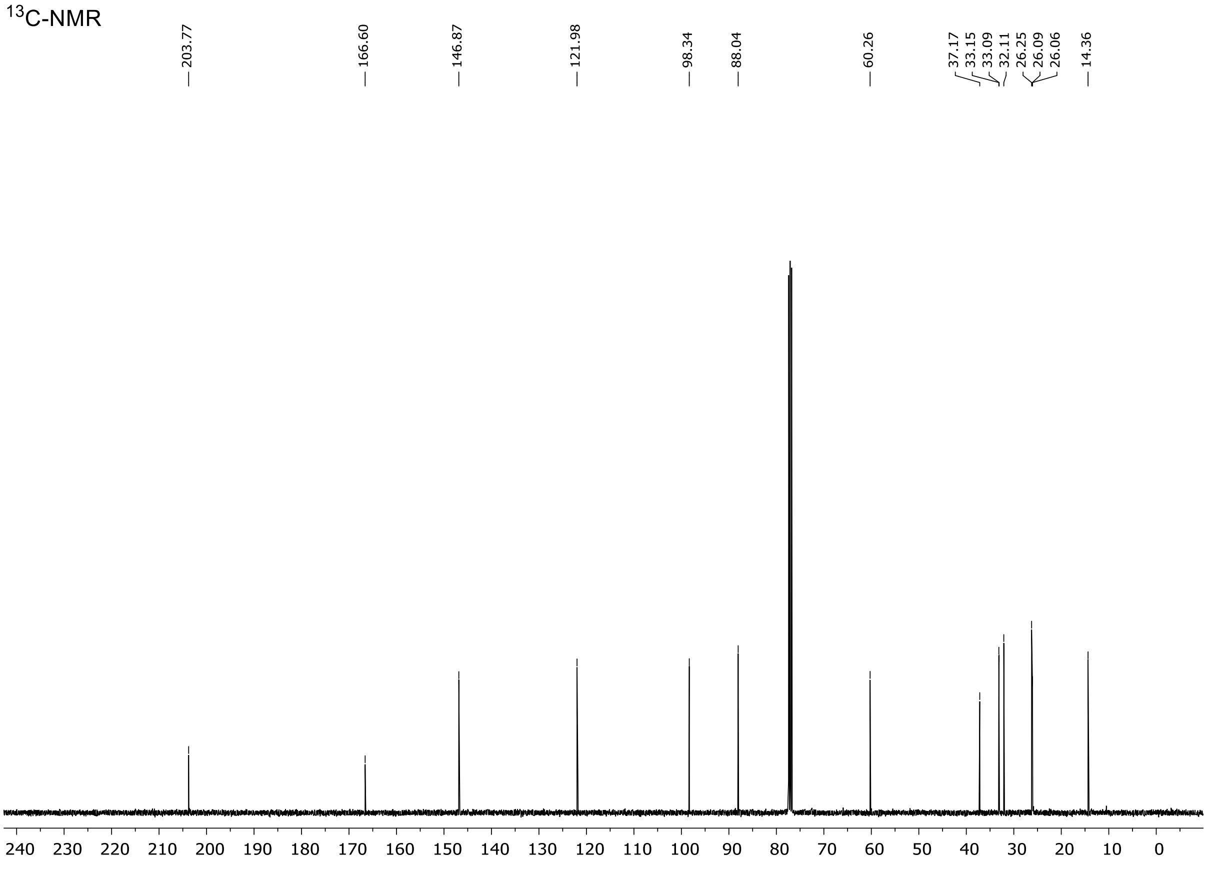

7-cyclohexyl-3-(4-methoxyphenyl)hepta-5,6-dien-1-ol 120

^{1}H-NMR

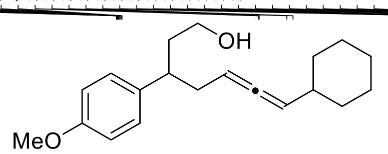

$C_{20}H_{28}O_2$
300.44

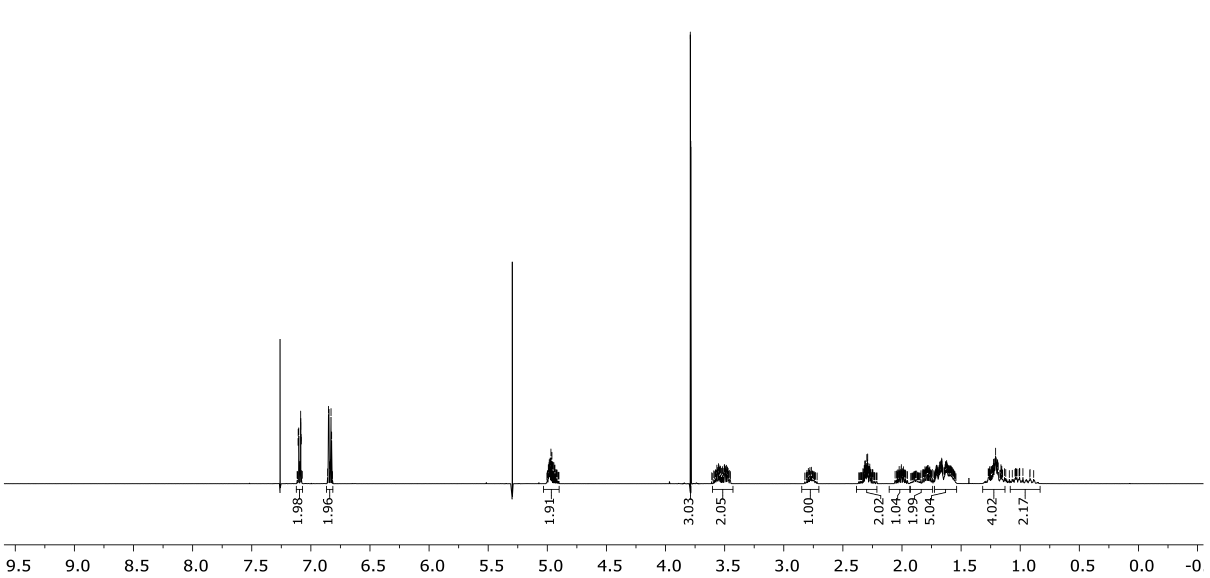

^{13}C-NMR

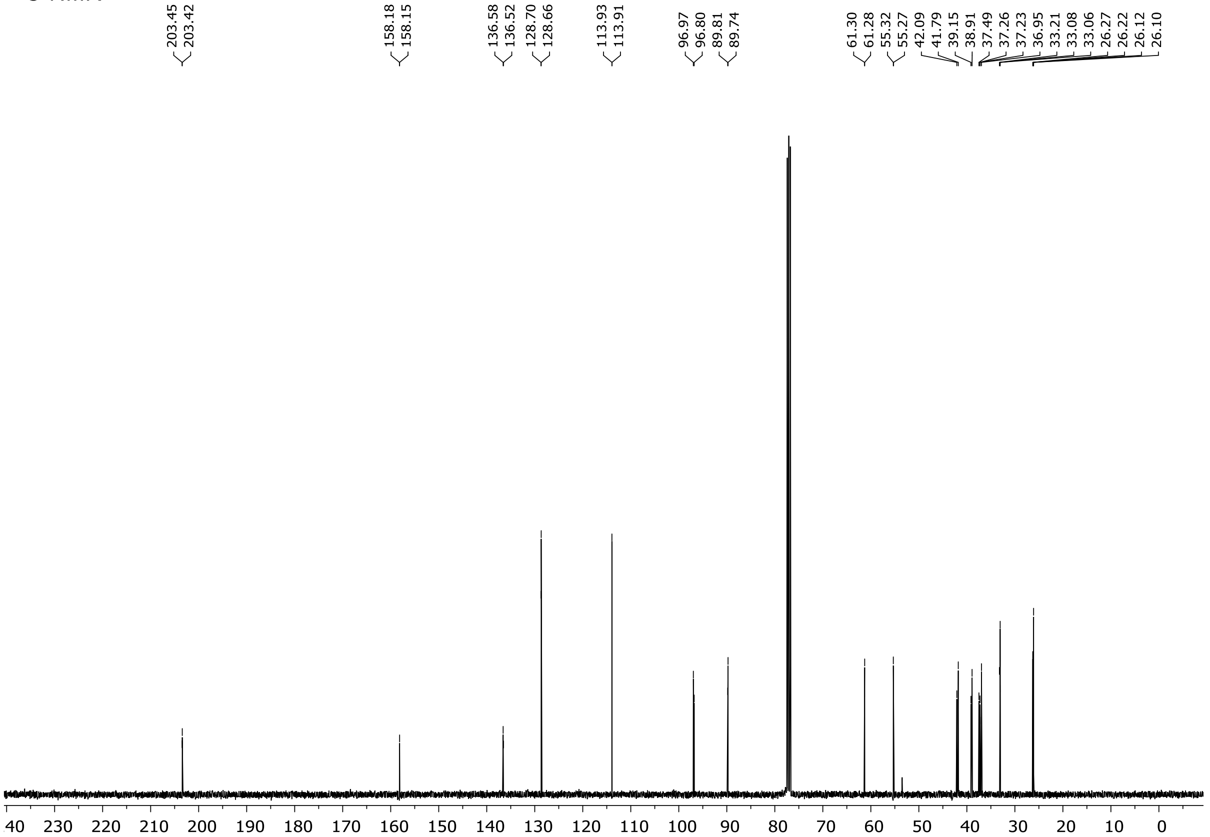

ethyl 7-phenylhepta-3,4-dienoate 121

^{1}H-NMR

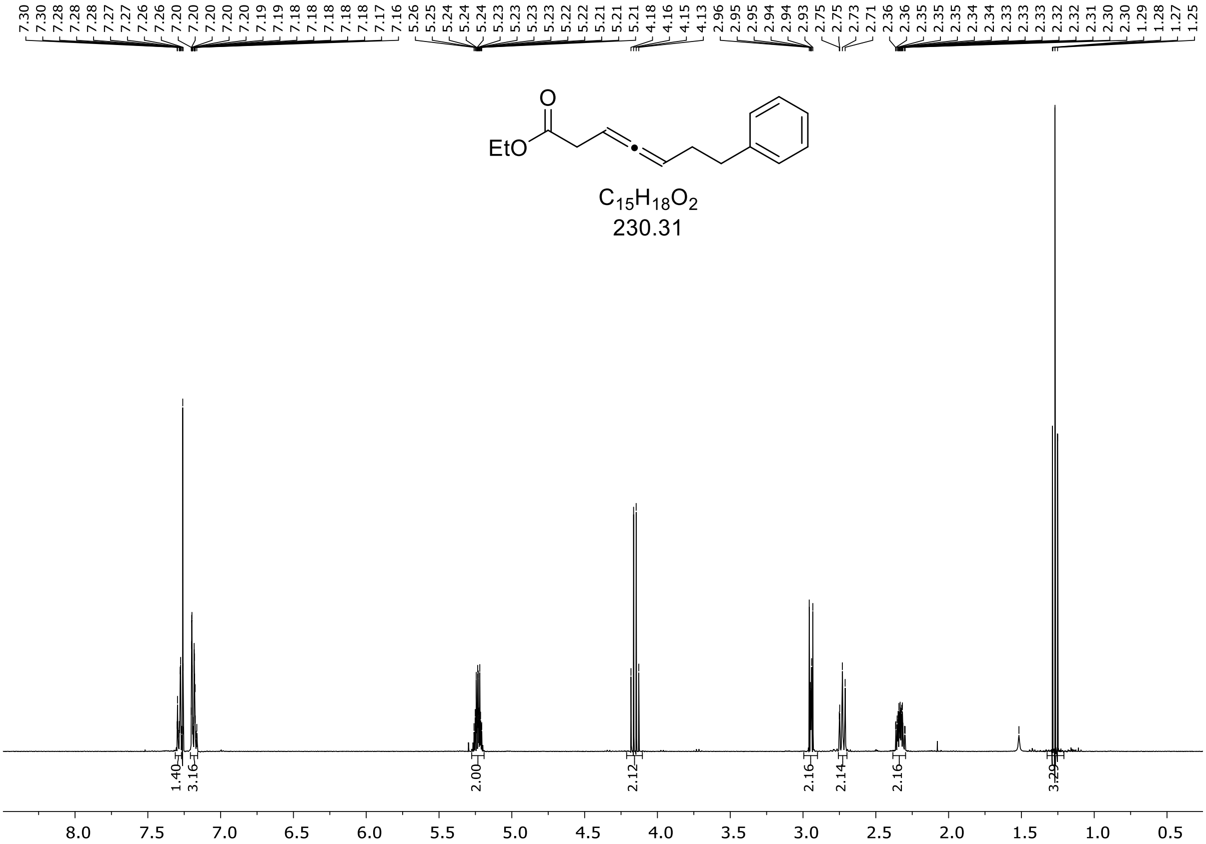

^{13}C-NMR

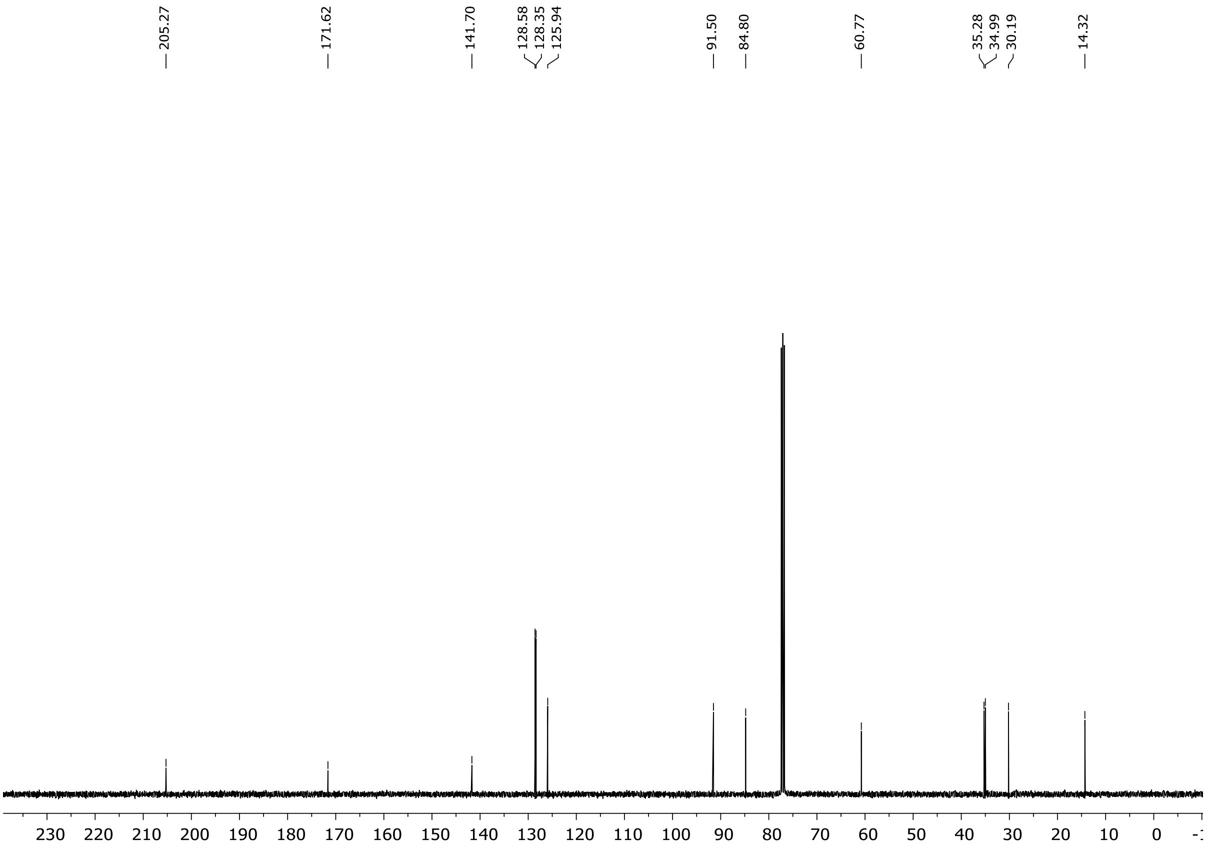

ethyl (E)-9-phenylnona-2,5,6-trienoate 122

^{1}H-NMR

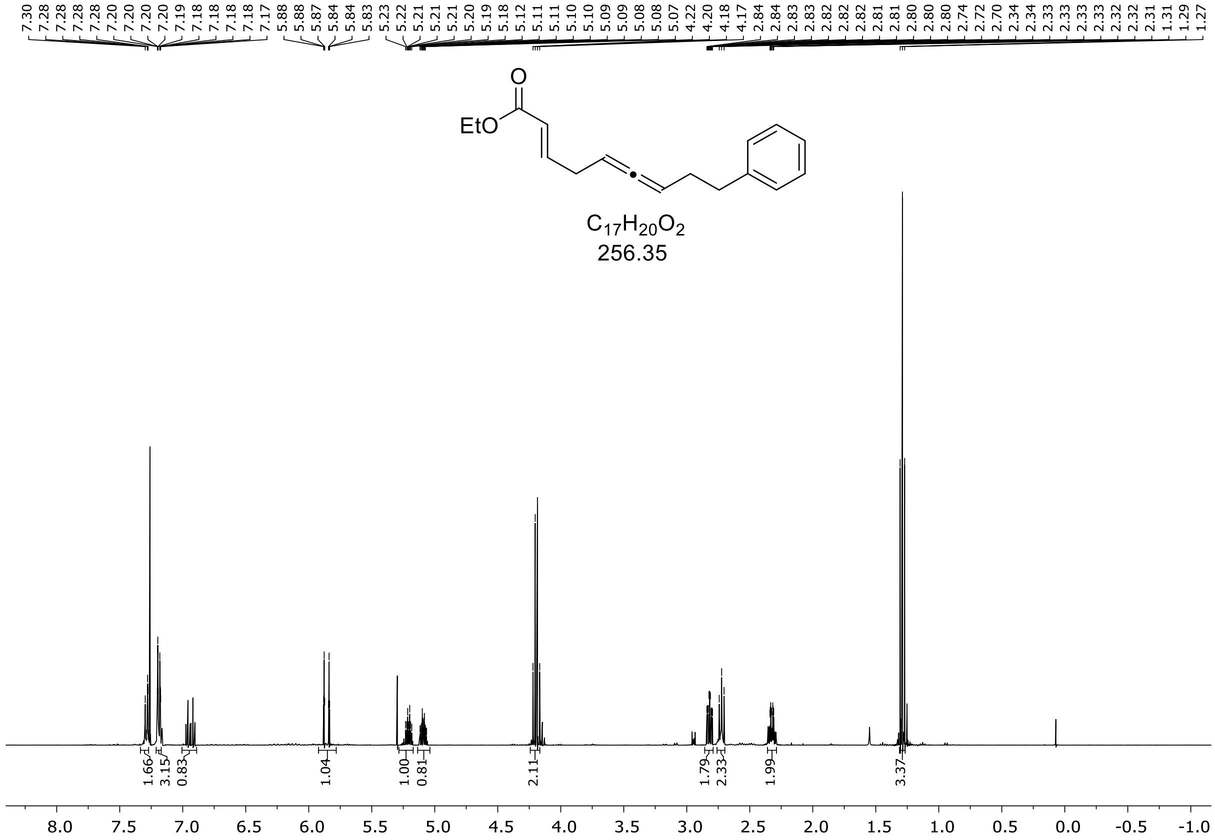

^{13}C-NMR

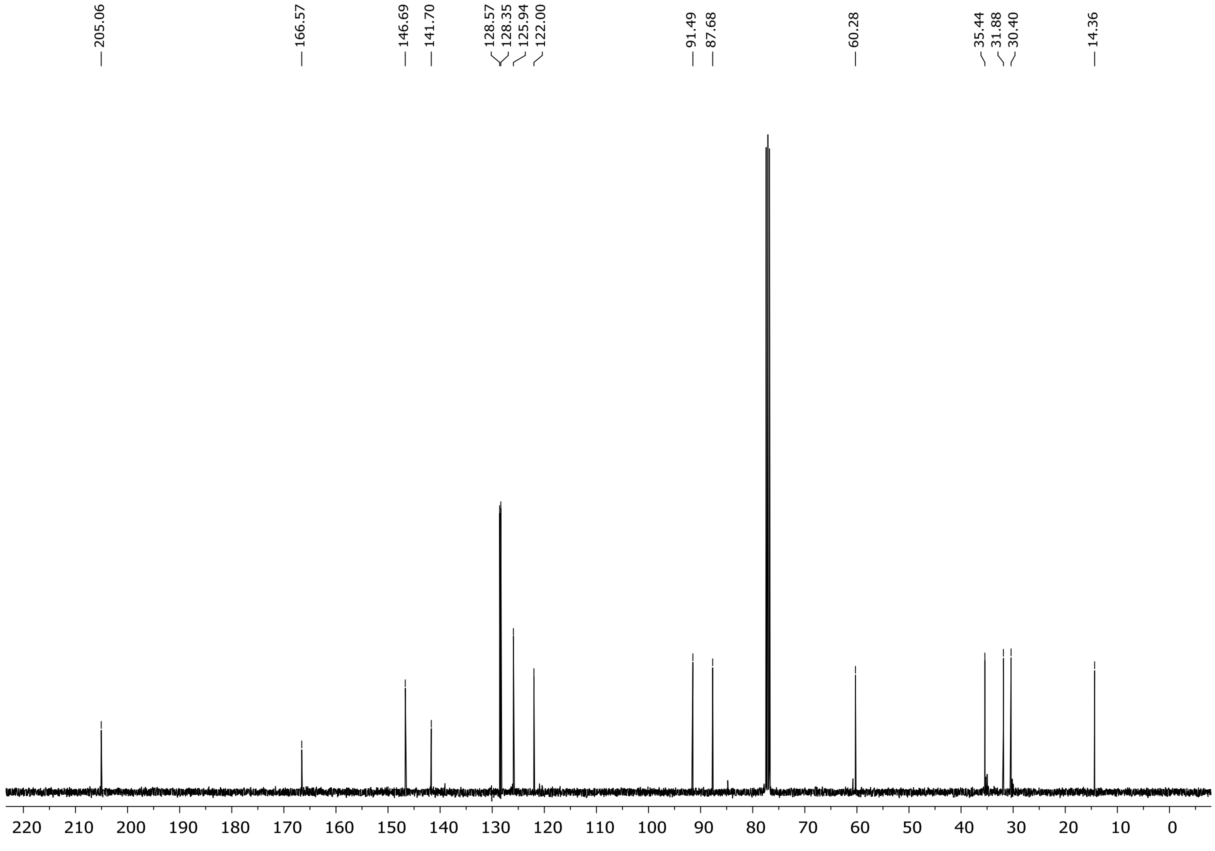

3-(4-methoxyphenyl)-9-phenylnona-5,6-dien-1-ol 123

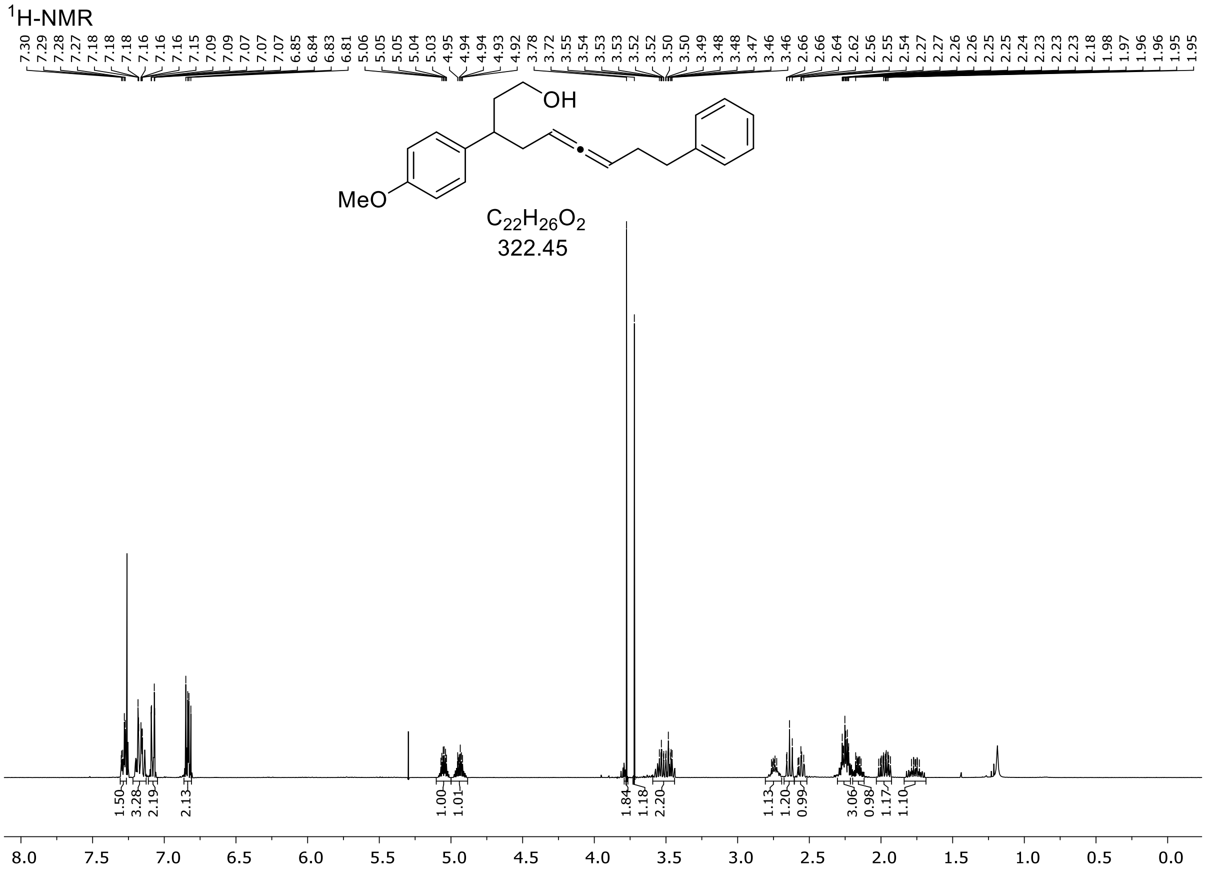

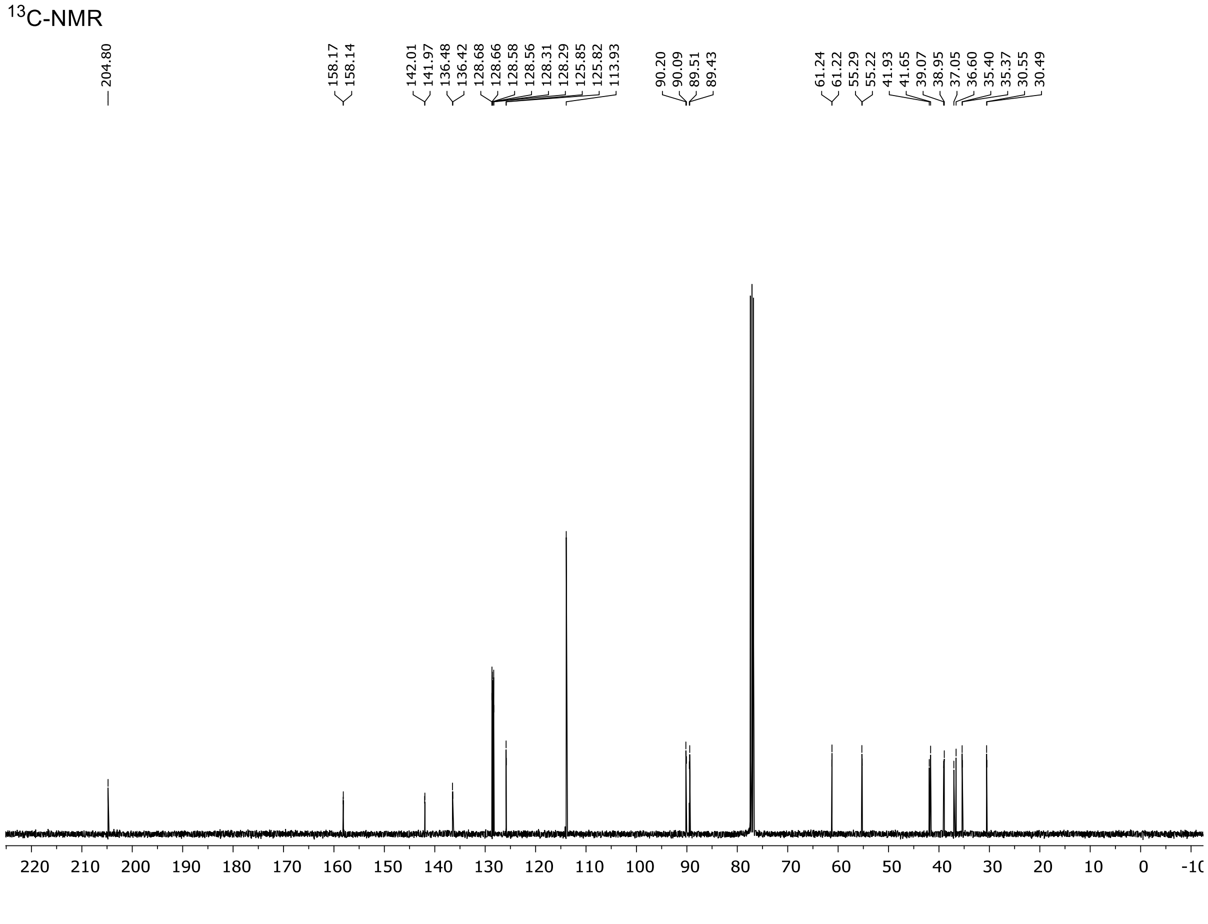

ethyl 6-phenylhexa-3,4-dienoate 124

^{1}H-NMR

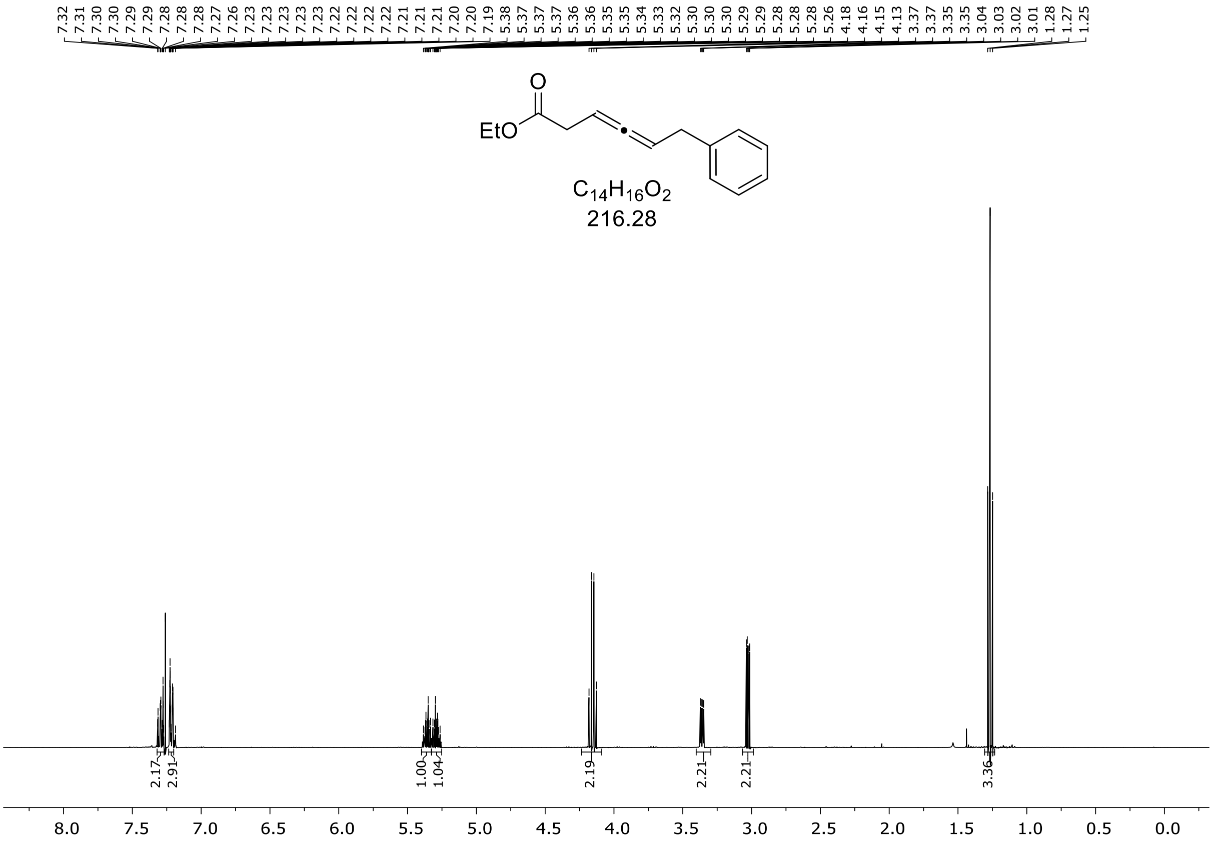

^{13}C-NMR

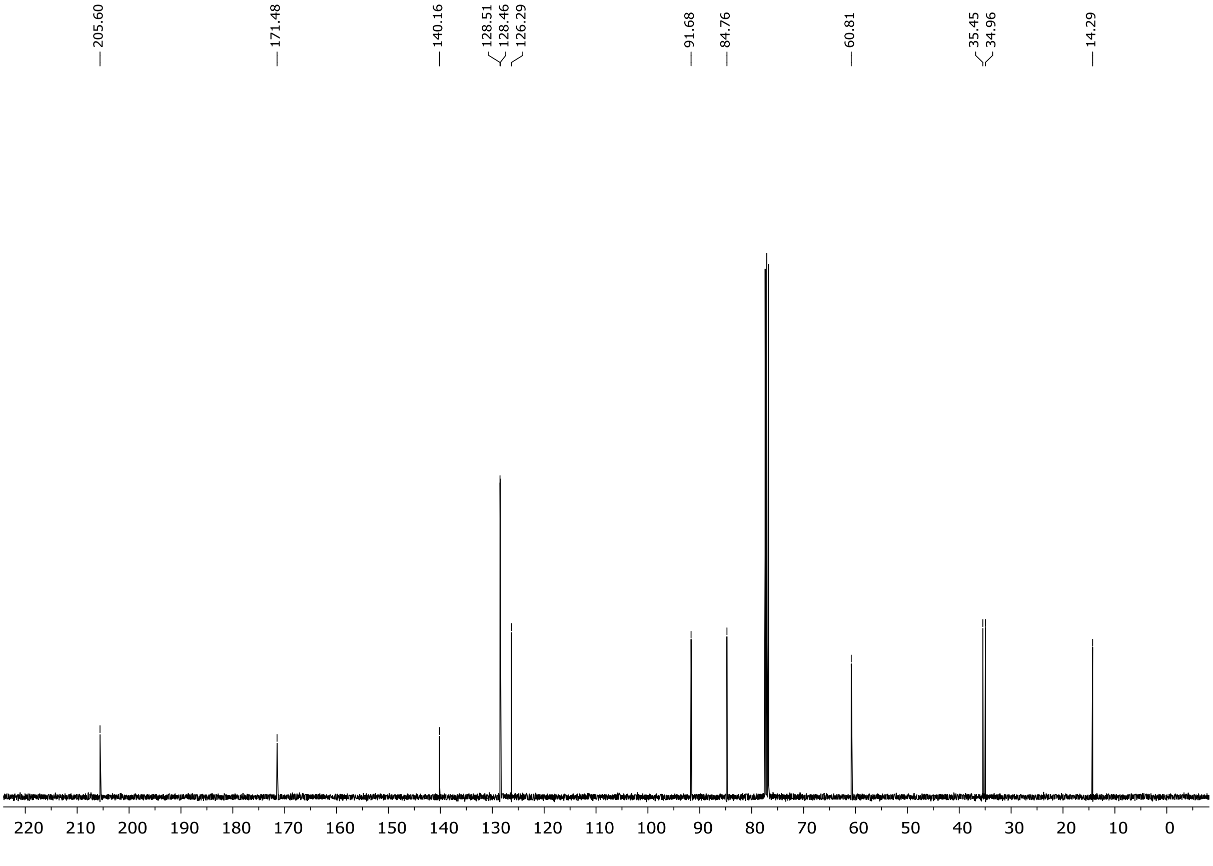

ethyl (E)-8-phenylocta-2,5,6-trienoate 125

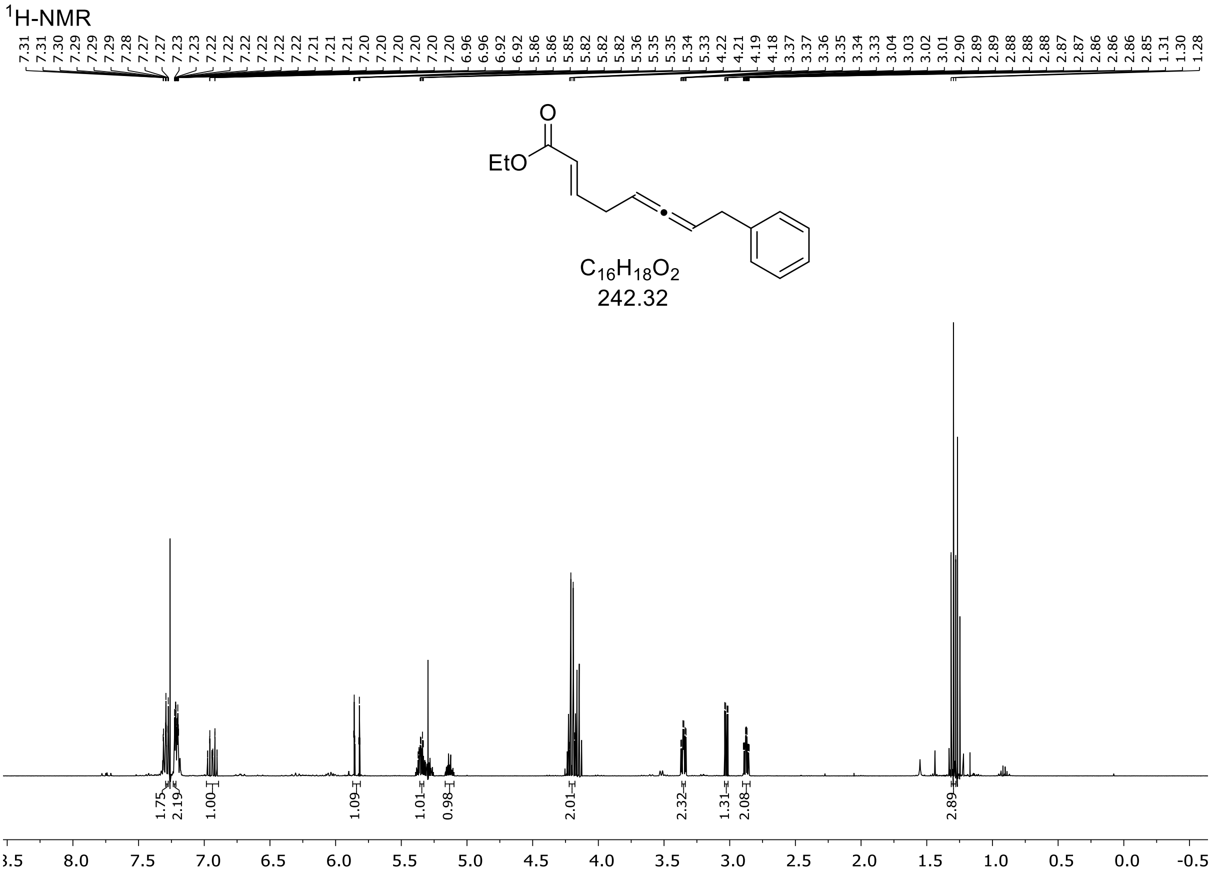

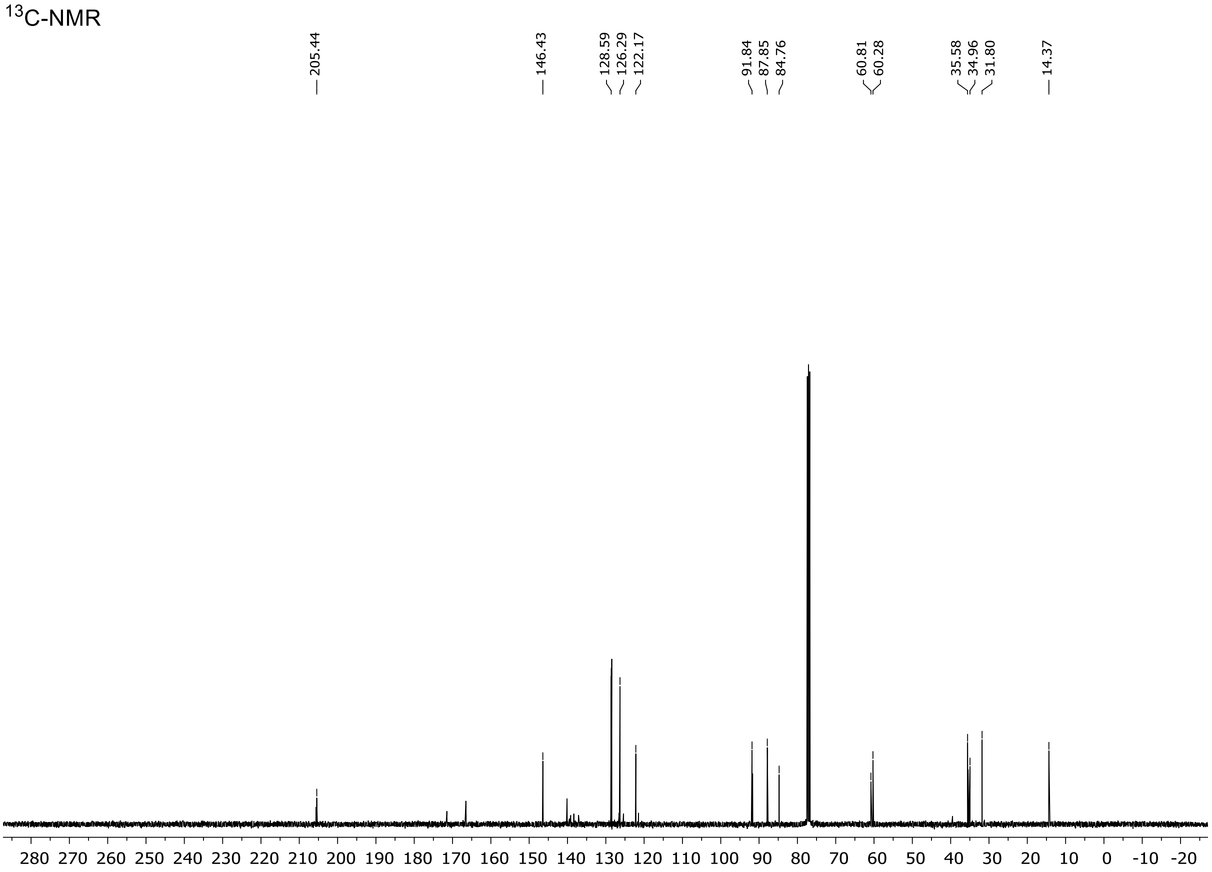

3-(4-methoxyphenyl)-8-phenylocta-5,6-dien-1-ol 126

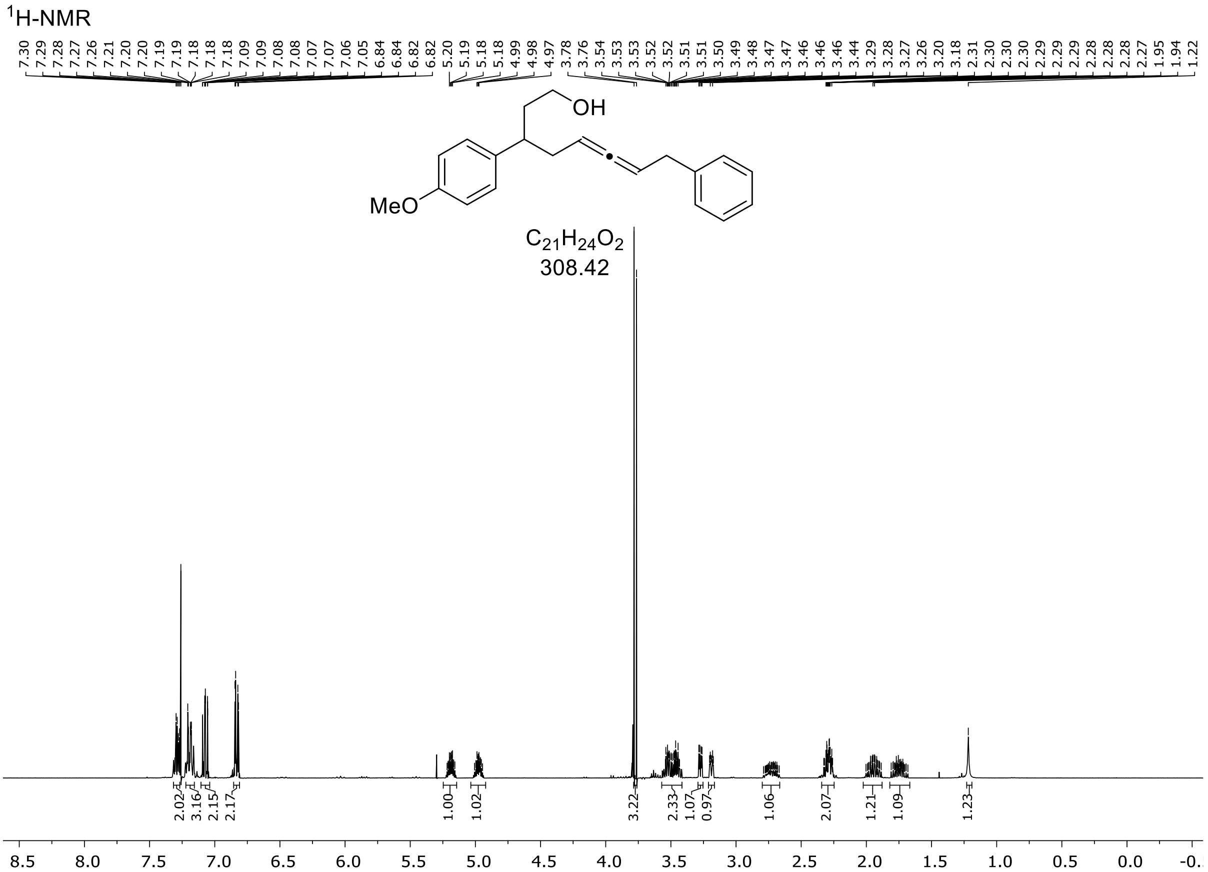

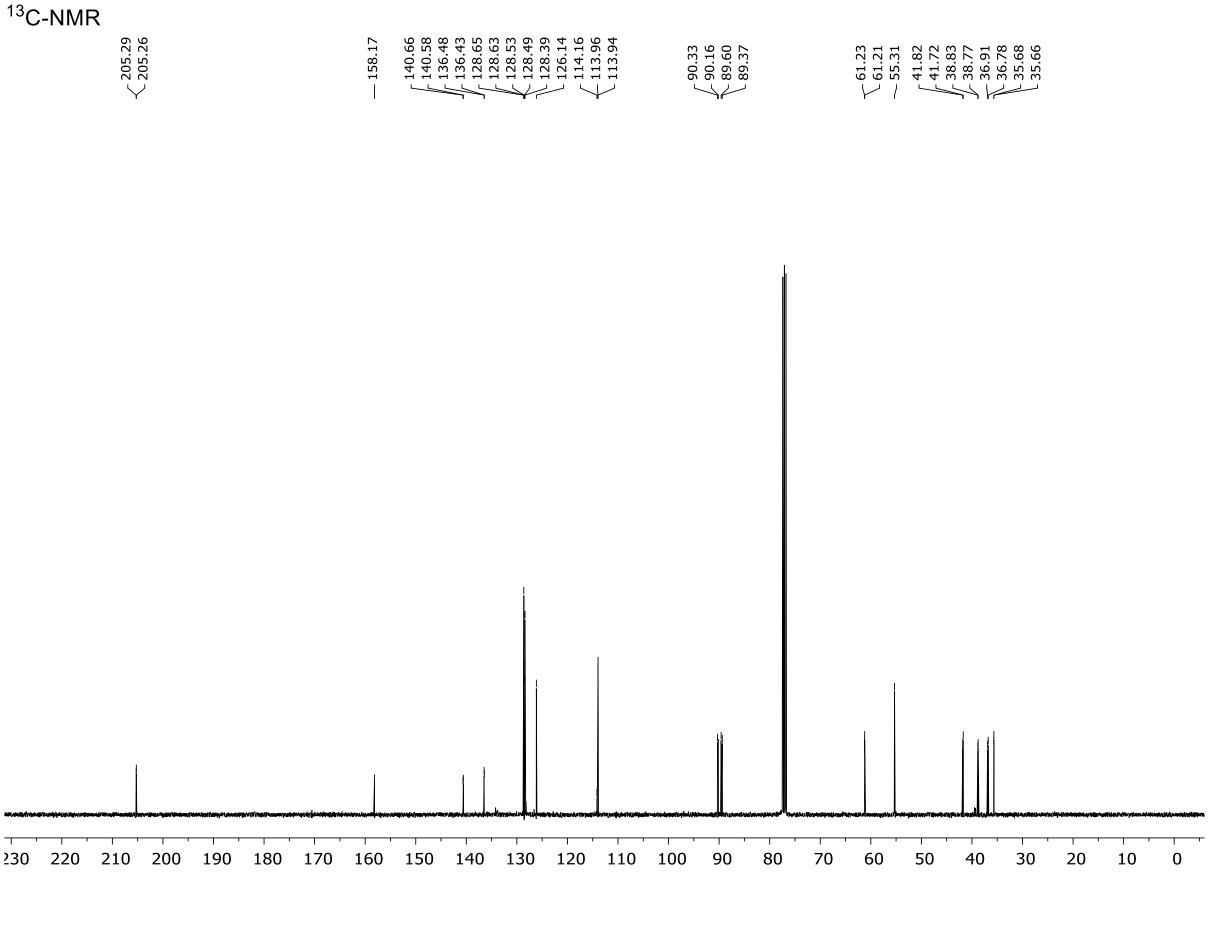

ethyl 7-(methylthio)hepta-3,4-dienoate 127

^{1}H-NMR

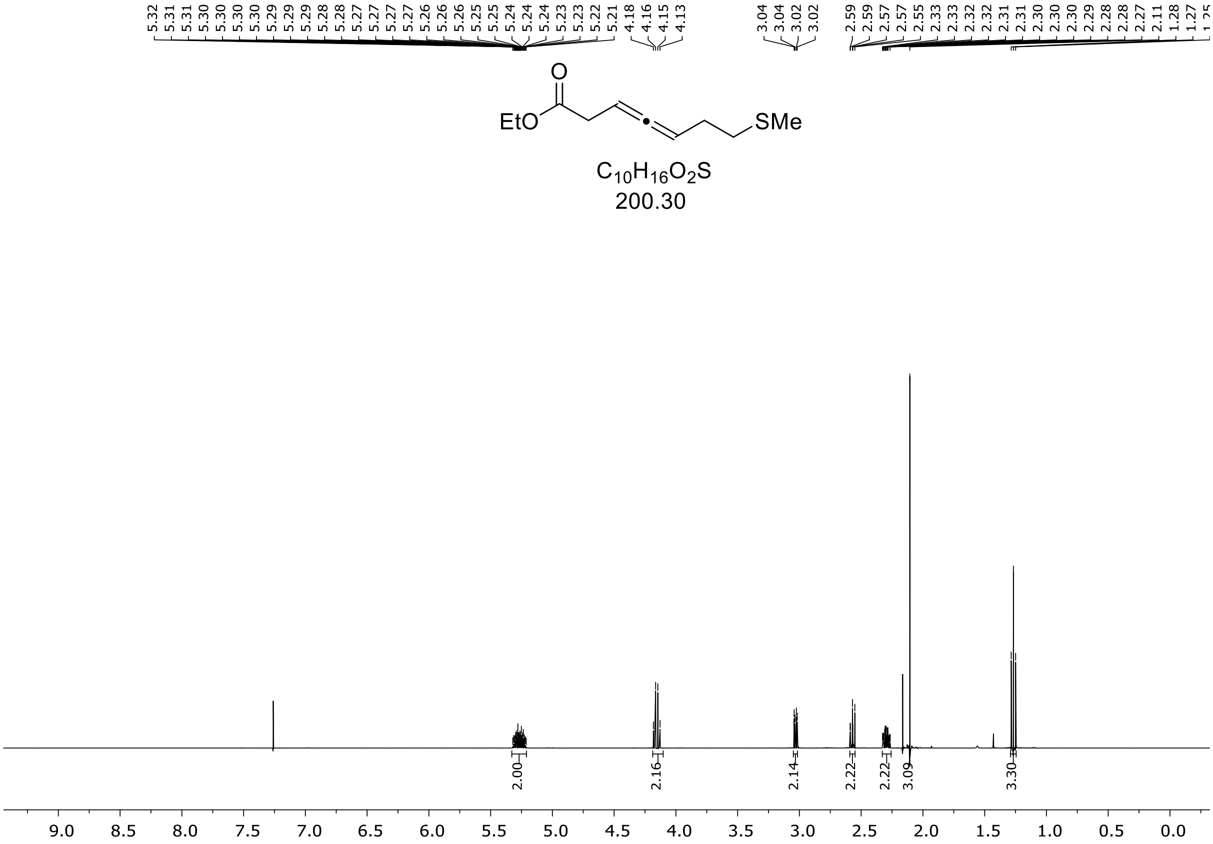

^{13}C-NMR

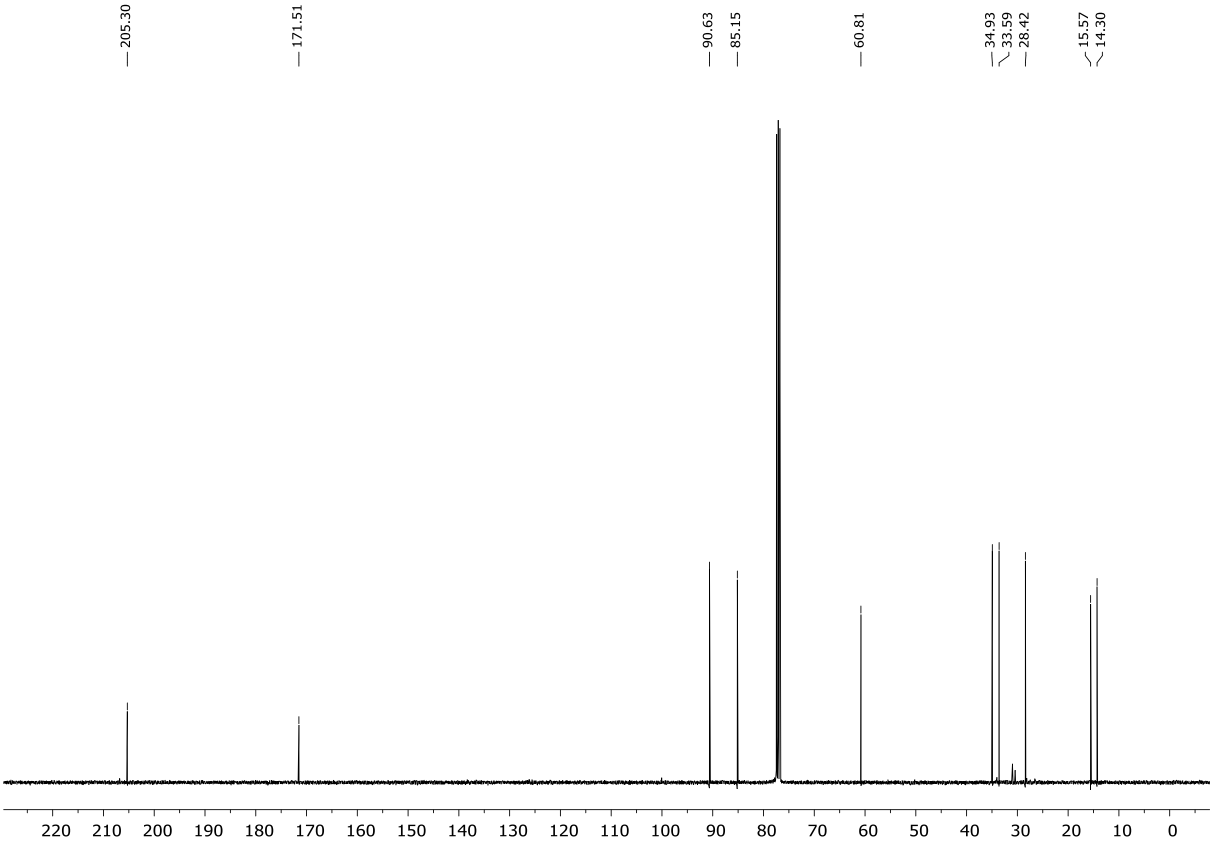

ethyl (E)-9-(methylthio)nona-2,5,6-trienoate 128

^{1}H-NMR

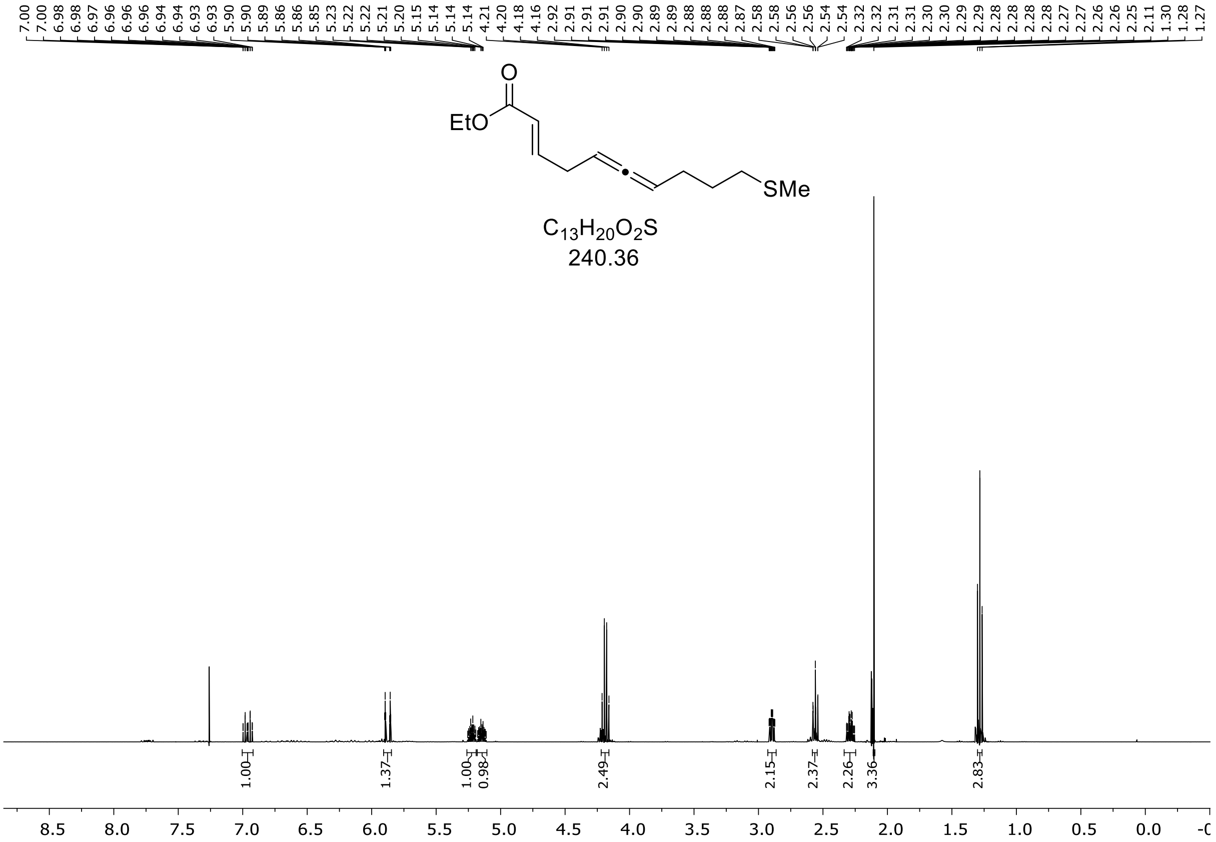

^{13}C-NMR

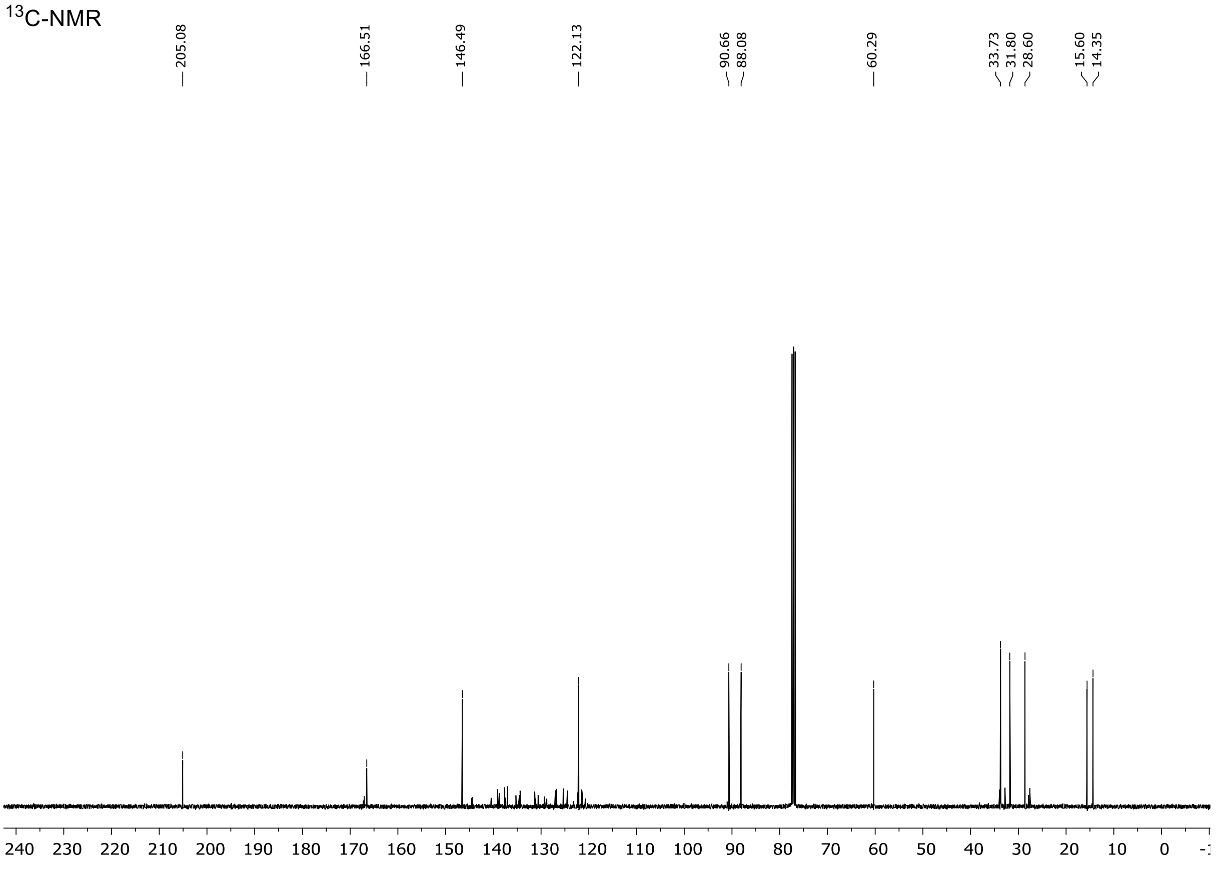

3-(4-methoxyphenyl)-9-(methylthio)nona-5,6-dien-1-ol 129

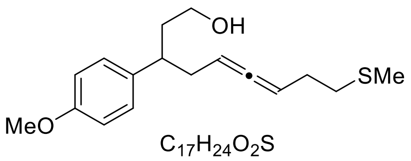

$C_{17}H_{24}O_2S$
292.44

^{1}H-NMR

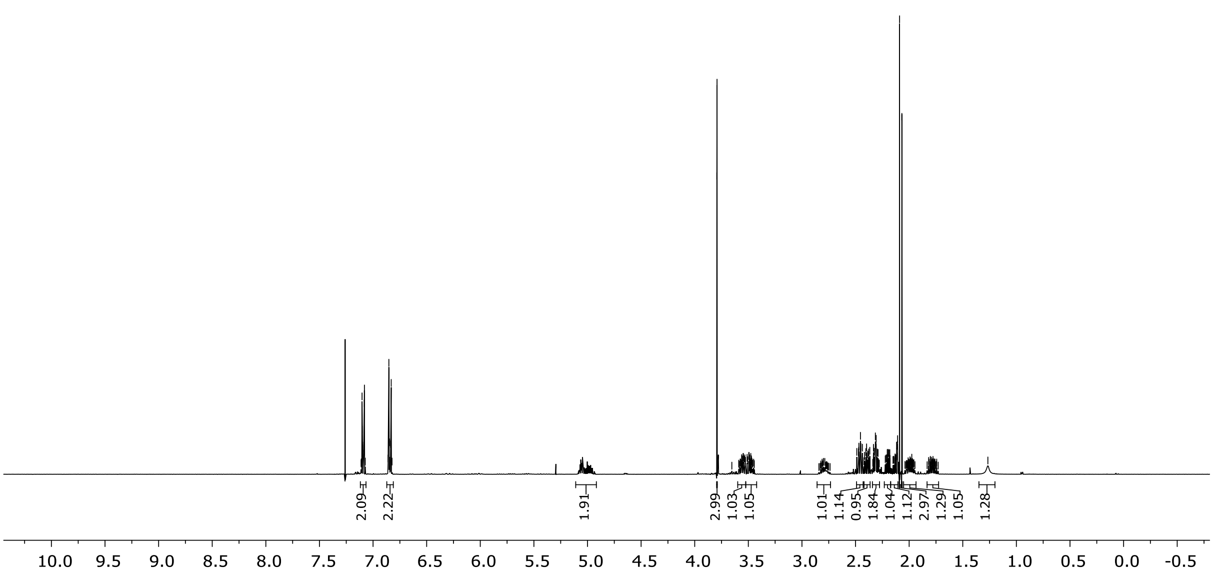

^{13}C-NMR

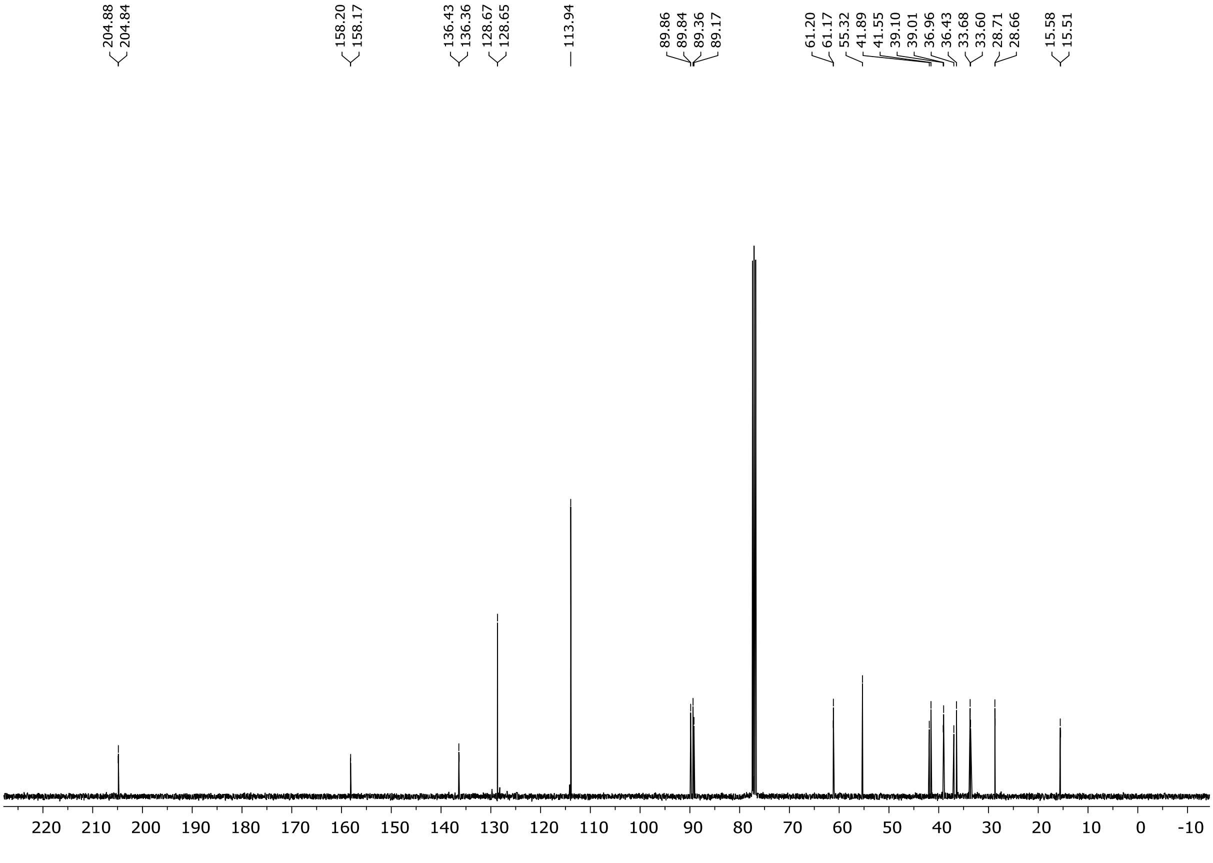

syn-4-(4-methoxyphenyl)octa-6,7-dien-2-ol 45

^{1}H-NMR

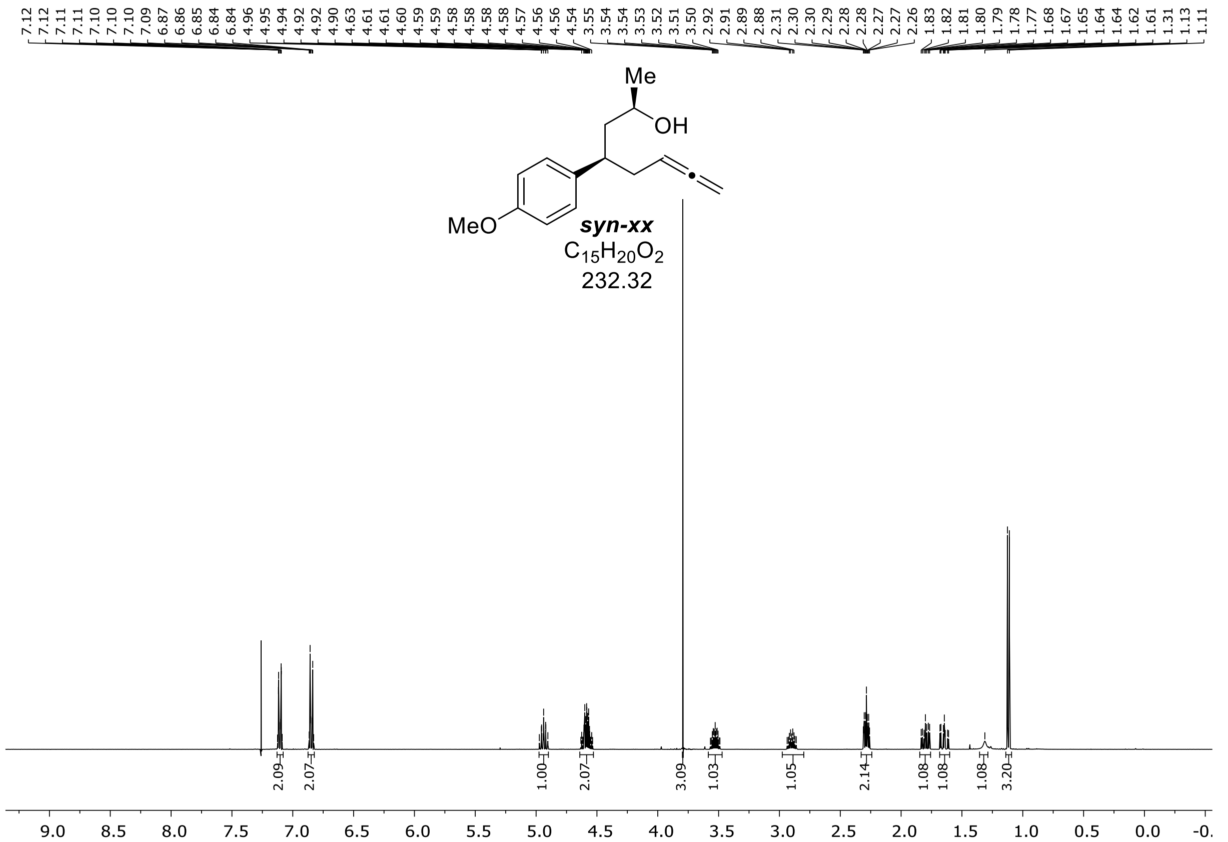

^{13}C-NMR

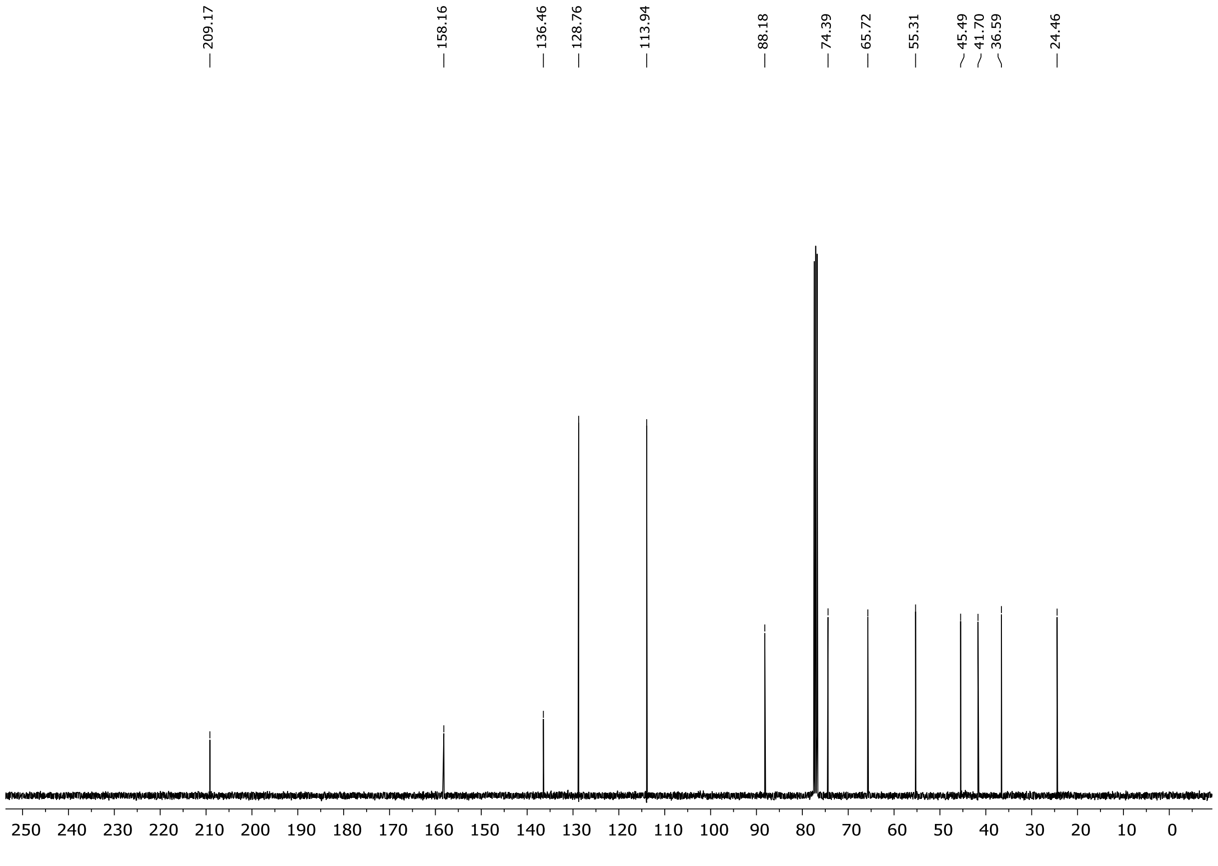

anti-4-(4-methoxyphenyl)octa-6,7-dien-2-ol 49

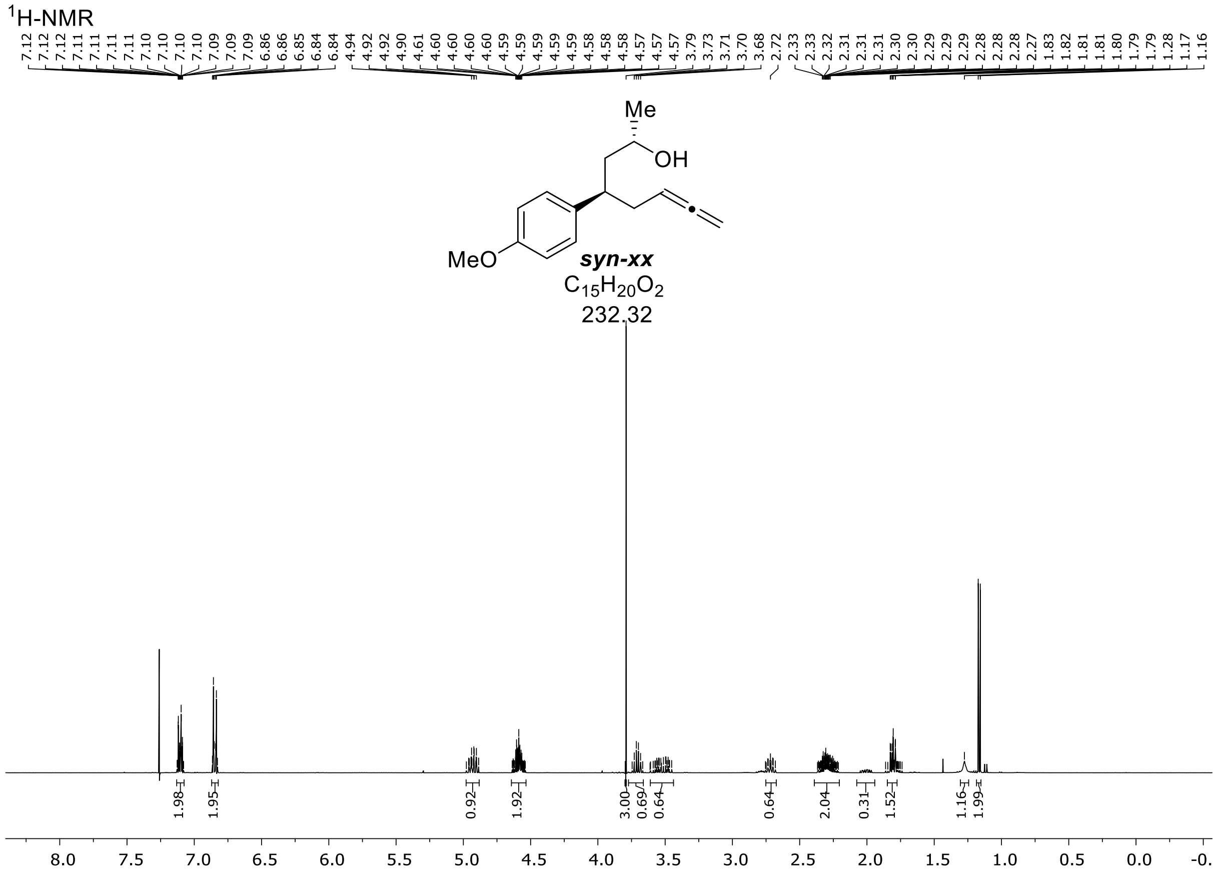

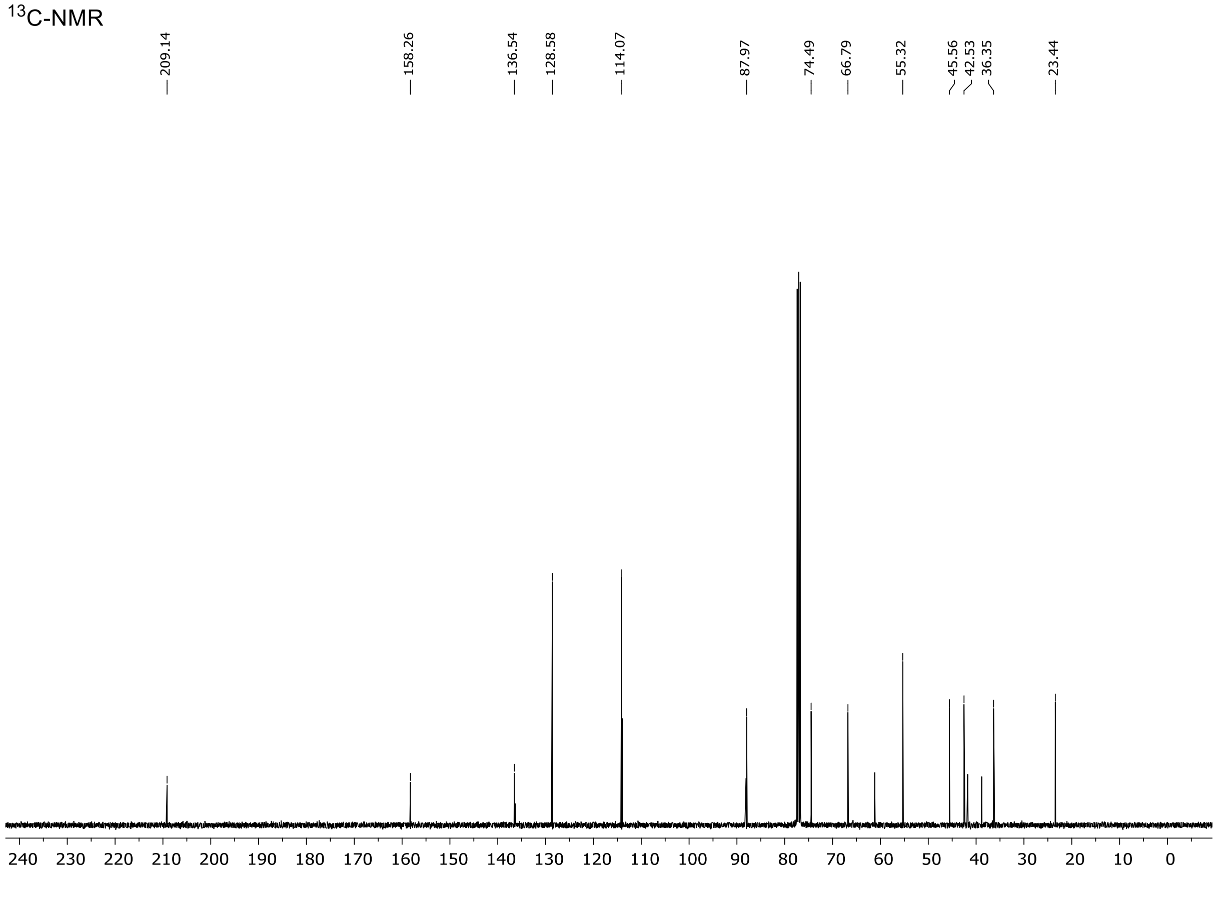

syn - 4-(4-methoxyphenyl)octa-6,7-dien-2-ol 46

^{1}H-NMR

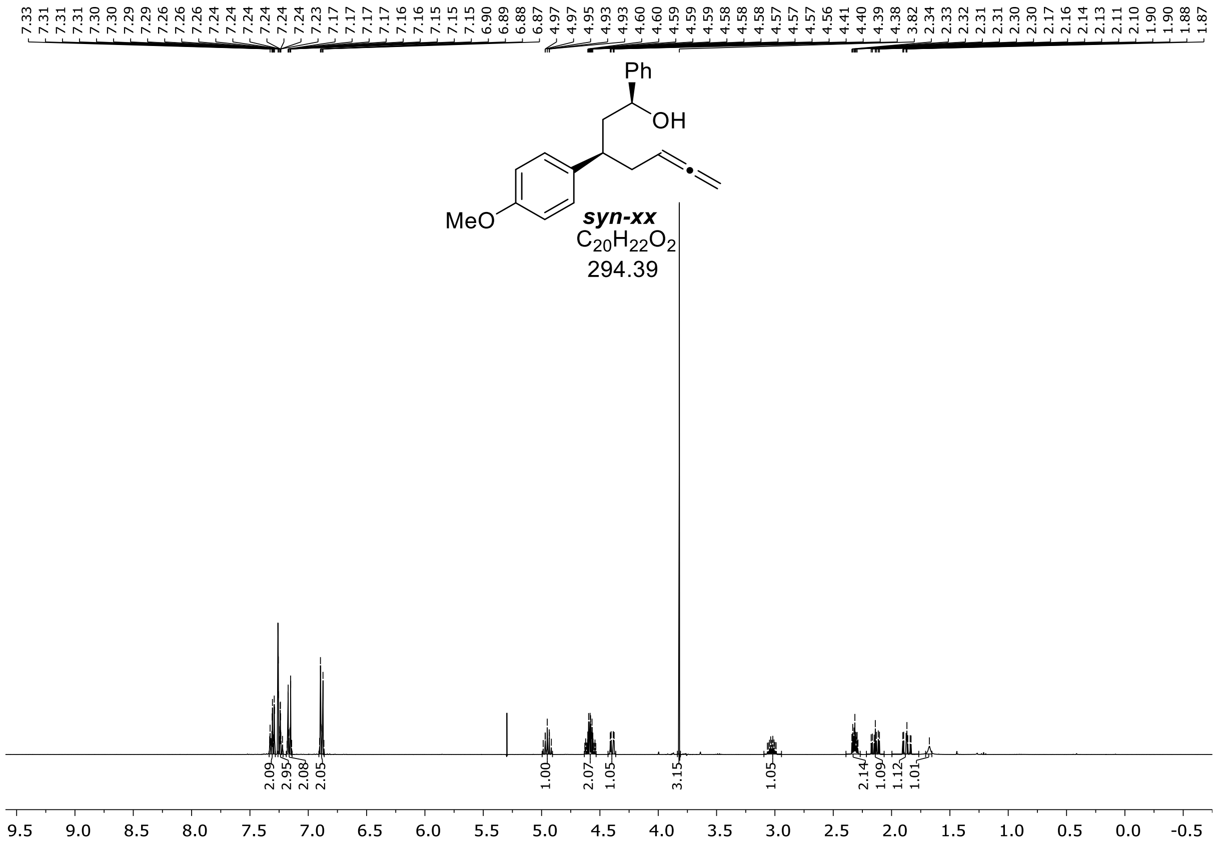

^{13}C-NMR

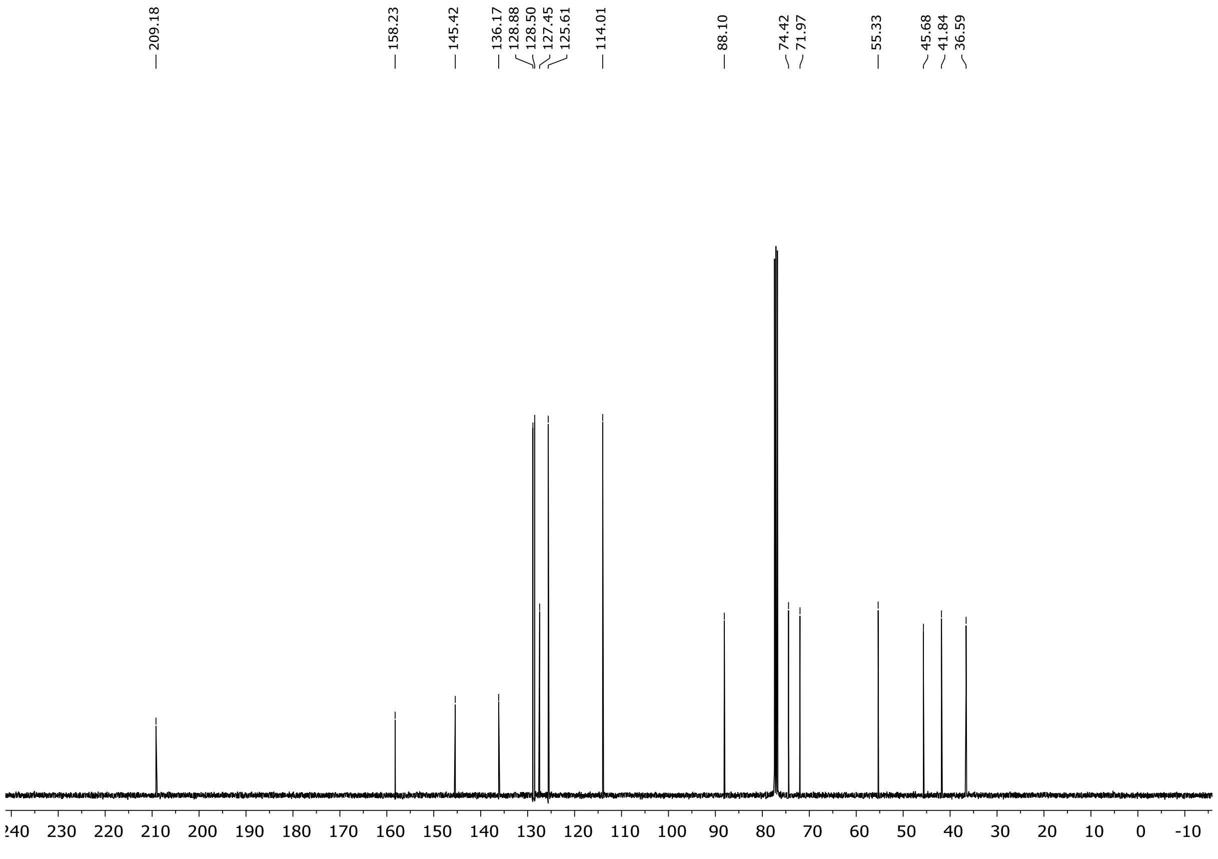

anti- 4-(4-methoxyphenyl)octa-6,7-dien-2-ol 50

^{1}H-NMR

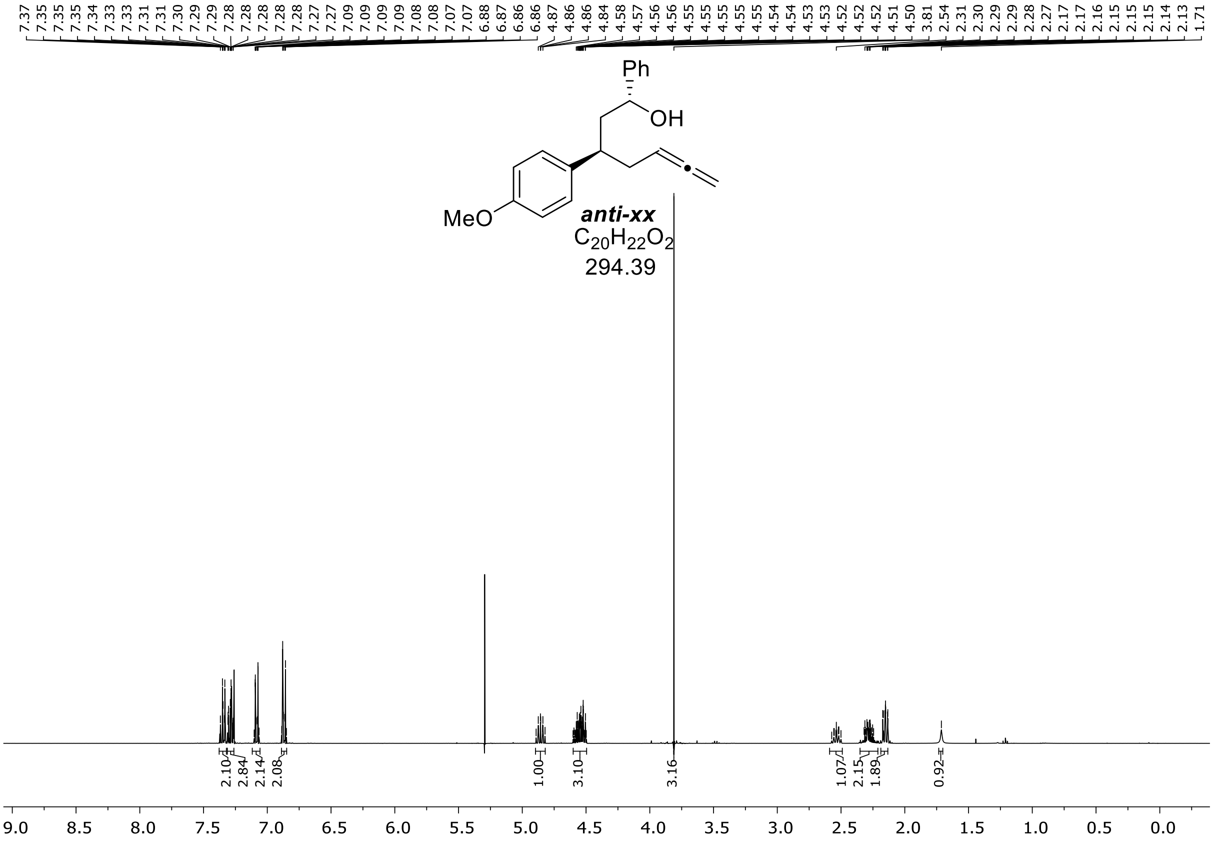

^{13}C-NMR

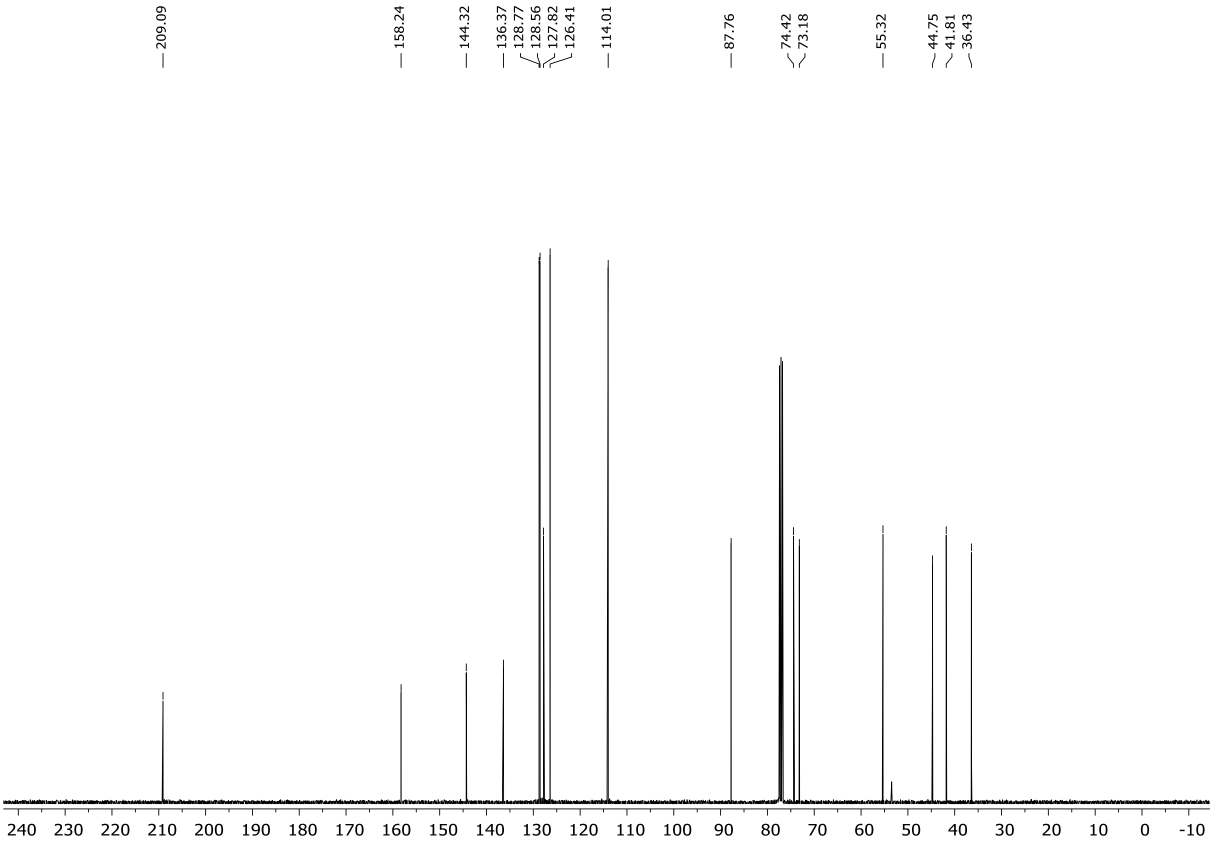

syn-4-(4-methoxyphenyl)tetradeca-6,7-dien-2-ol 47

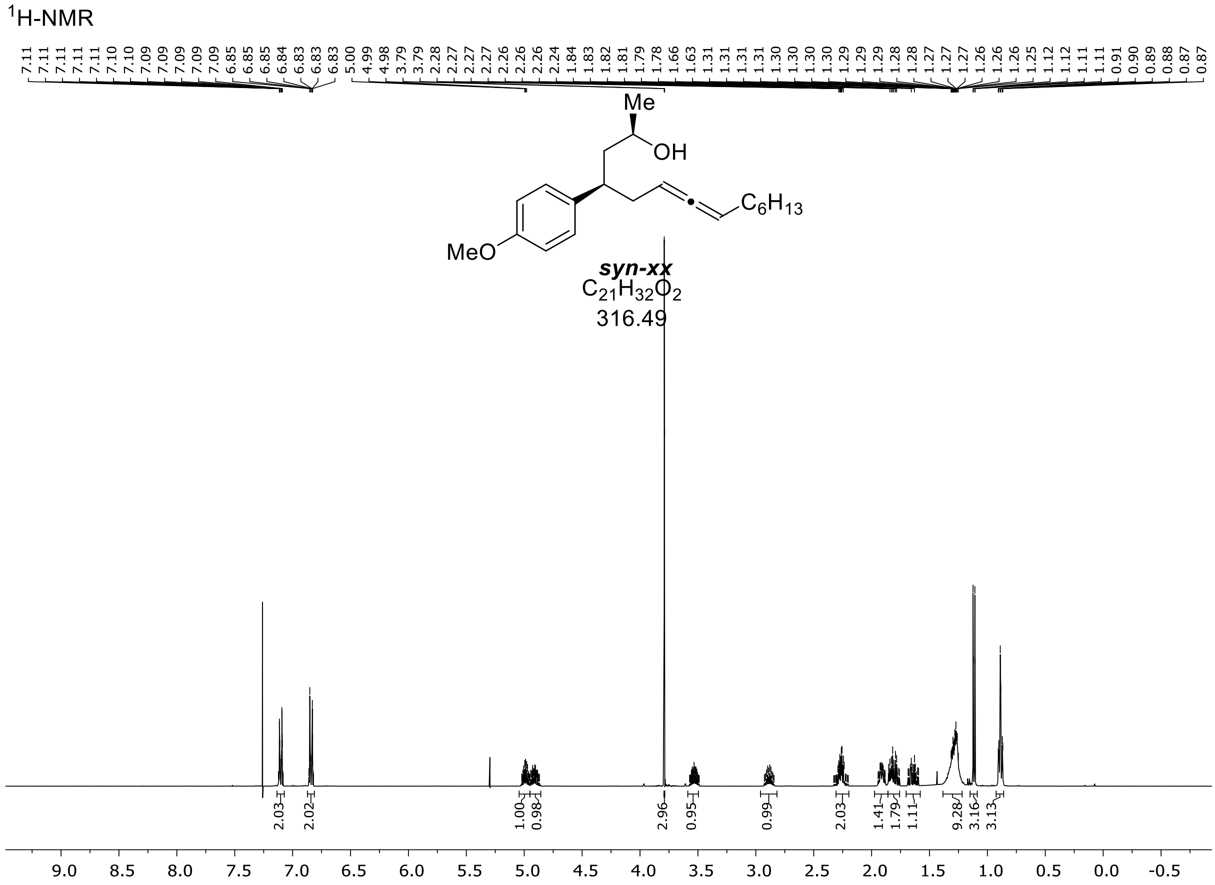

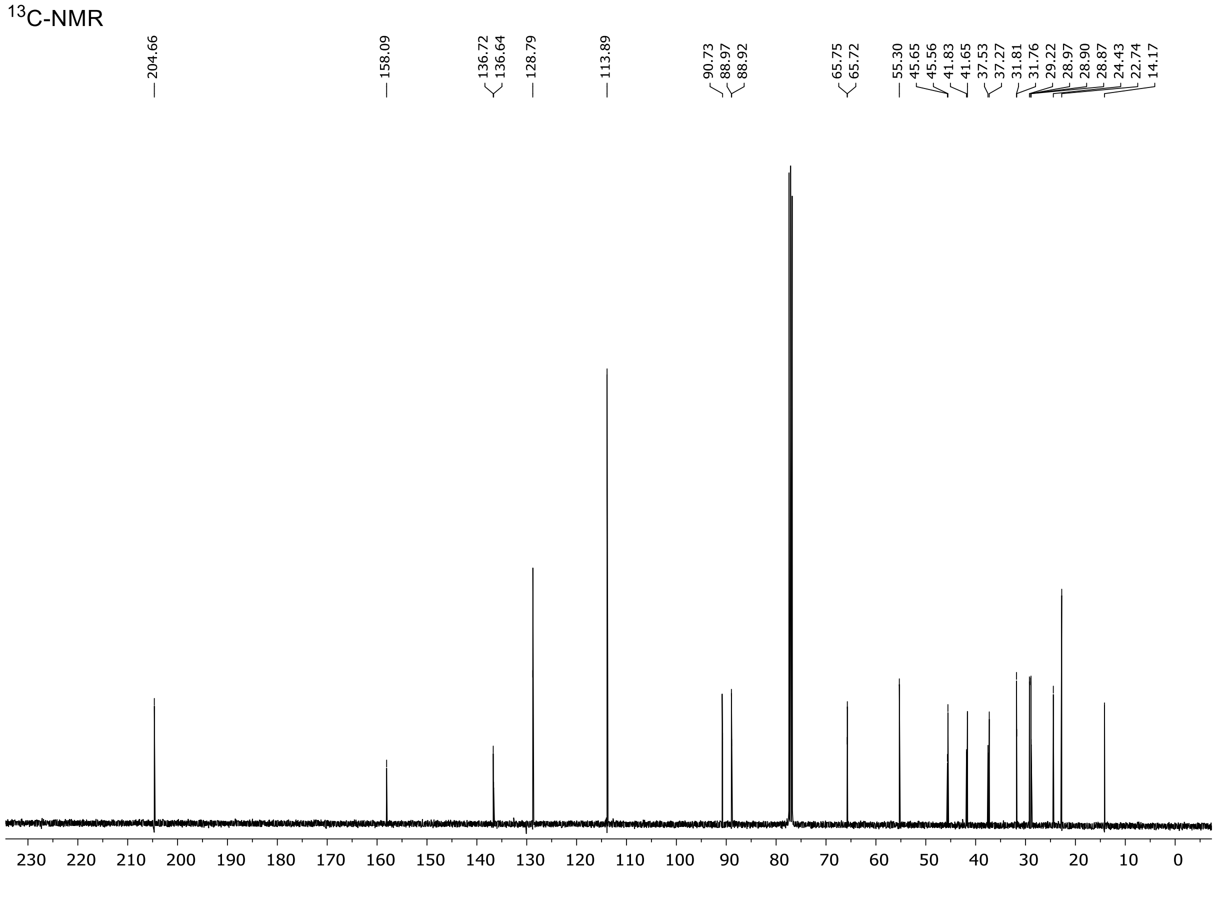

anti-4-(4-methoxyphenyl)tetradeca-6,7-dien-2-ol 51

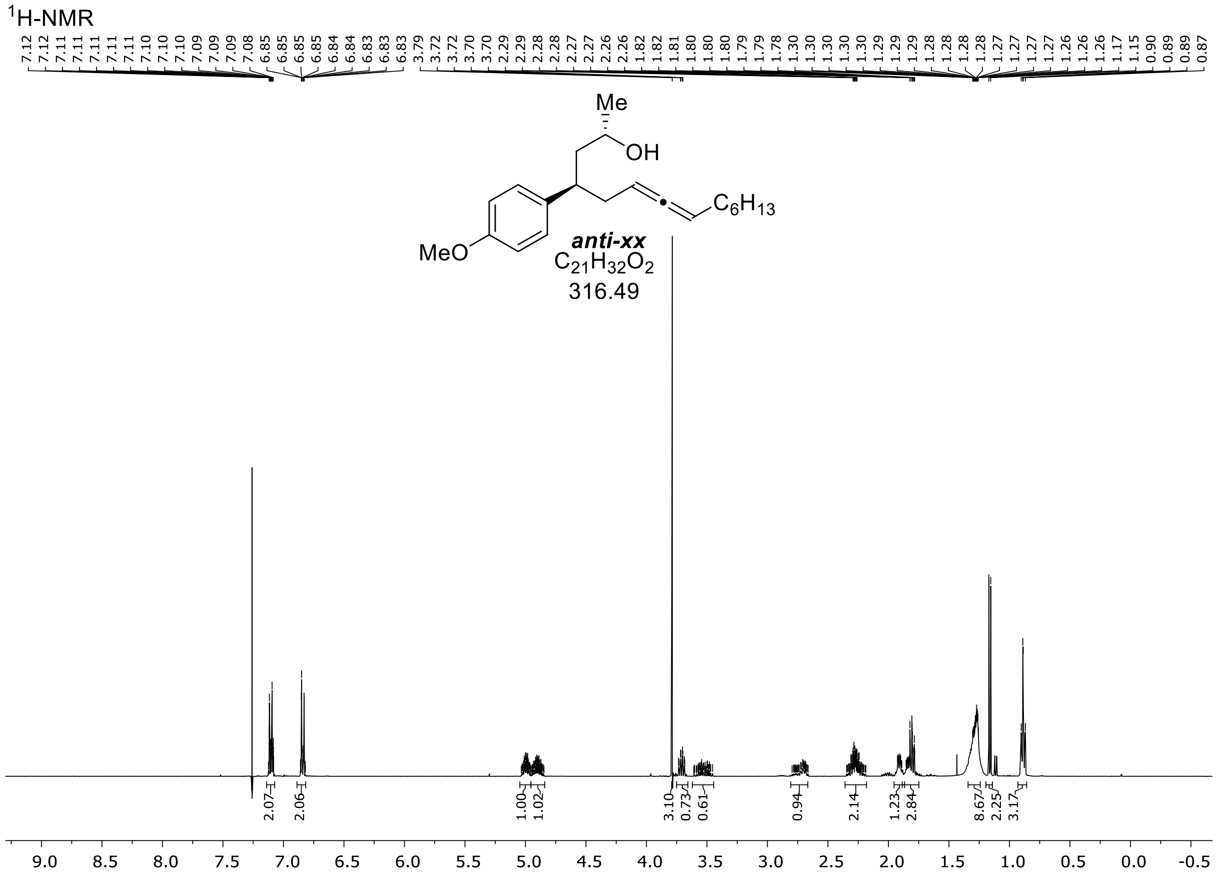

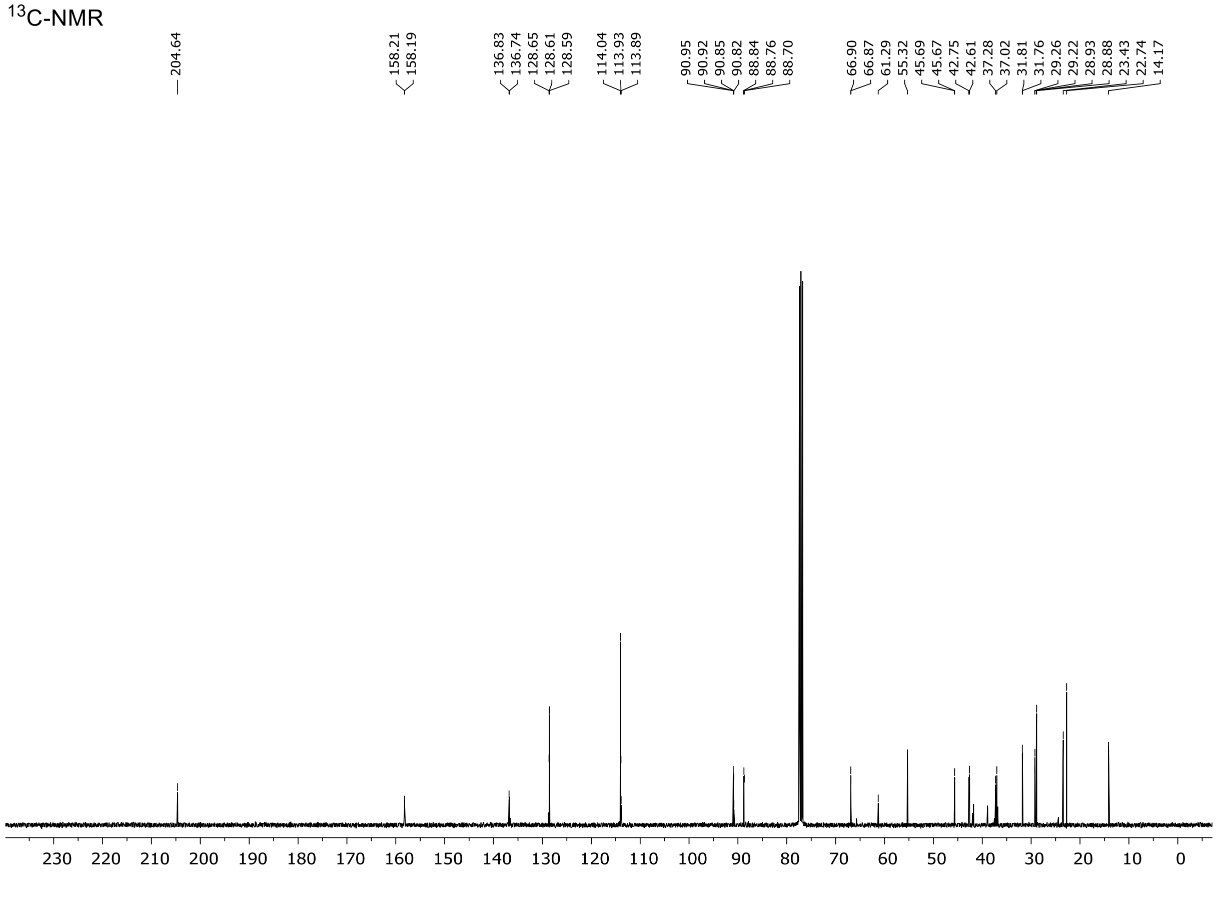

syn - 3-(4-methoxyphenyl)-1-phenyltrideca-5,6-dien-1-ol 48

^{1}H-NMR

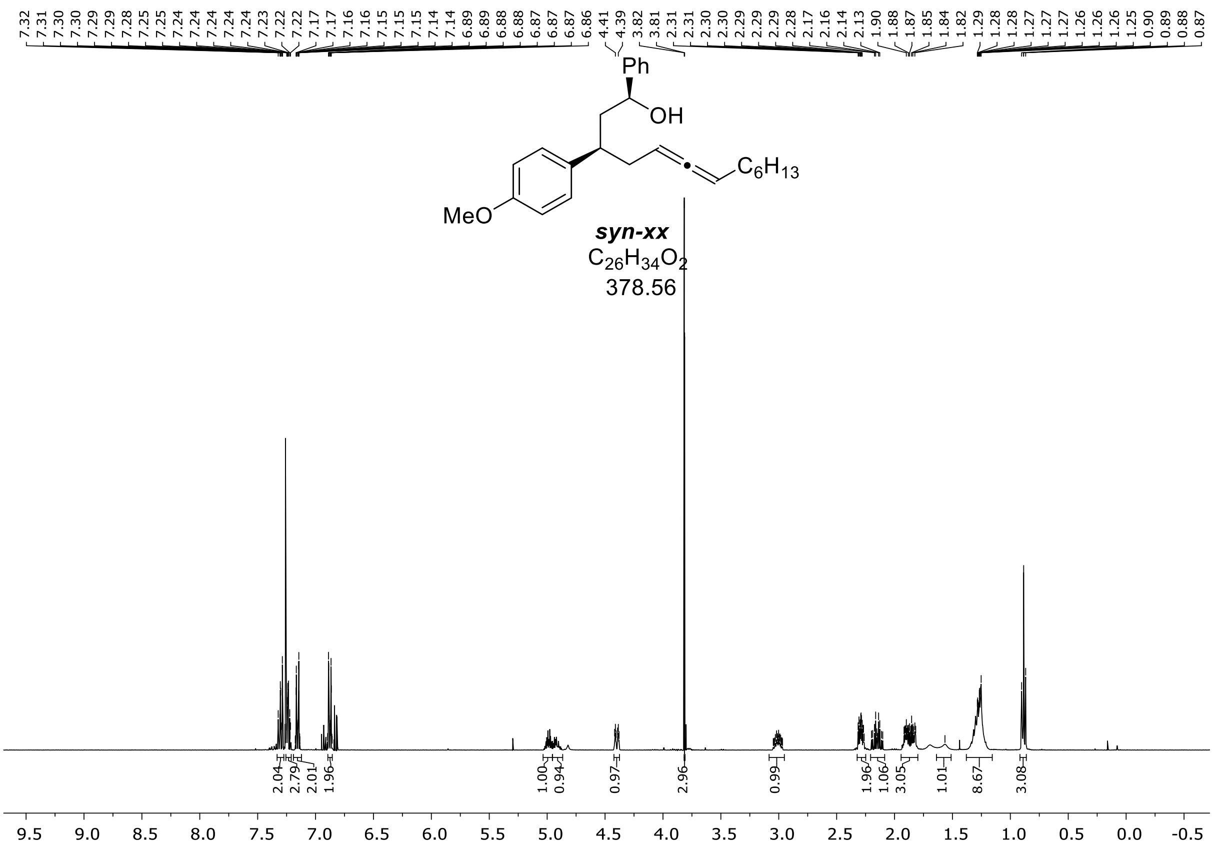

^{13}C-NMR

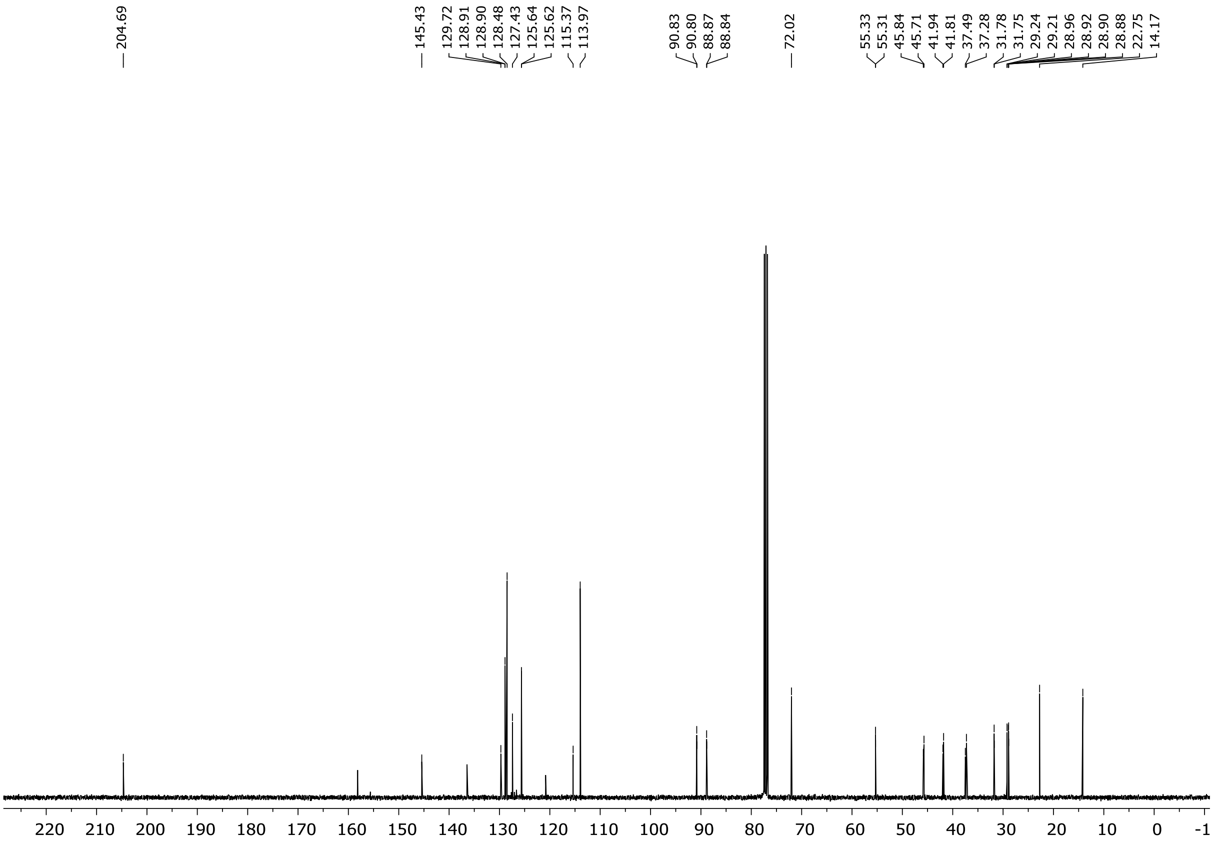

anti **- 3-(4-methoxyphenyl)-1-phenyltrideca-5,6-dien-1-ol 52**

^{1}H-NMR

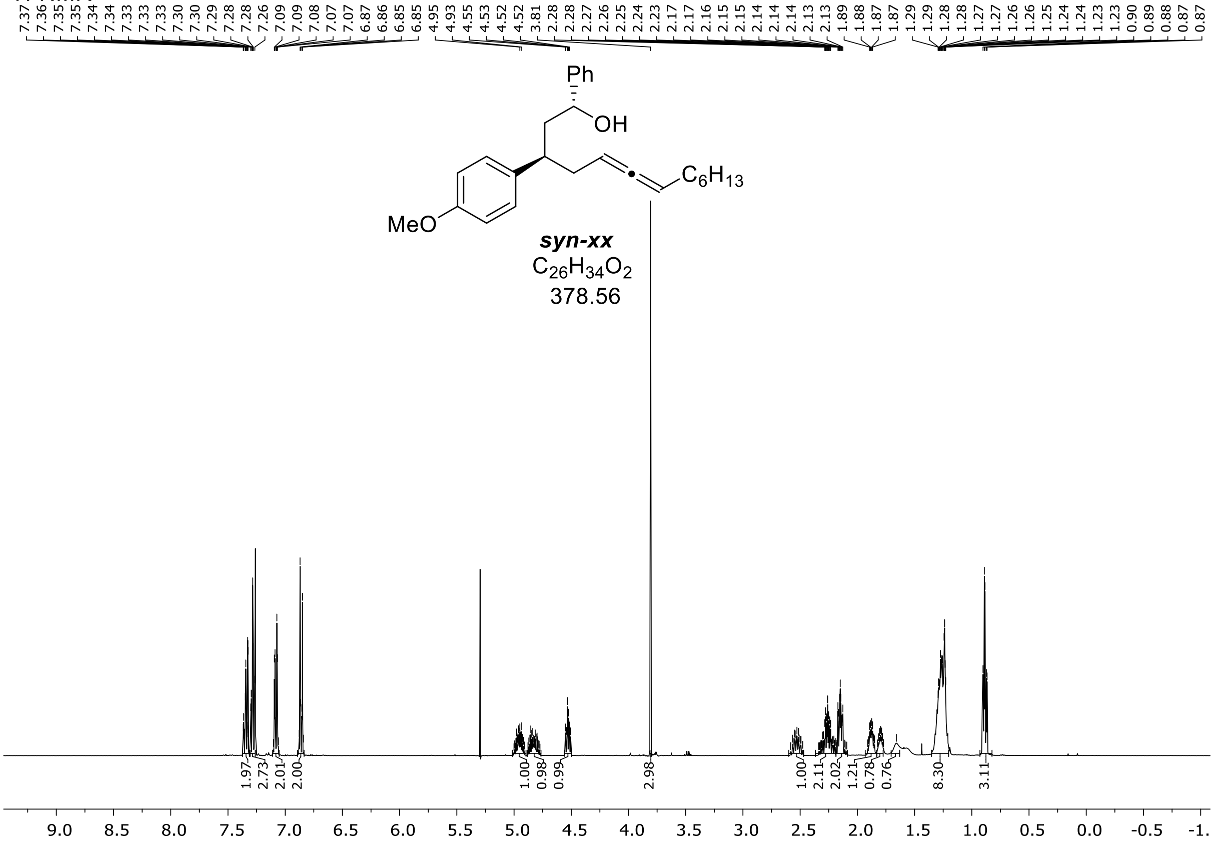

^{13}C-NMR

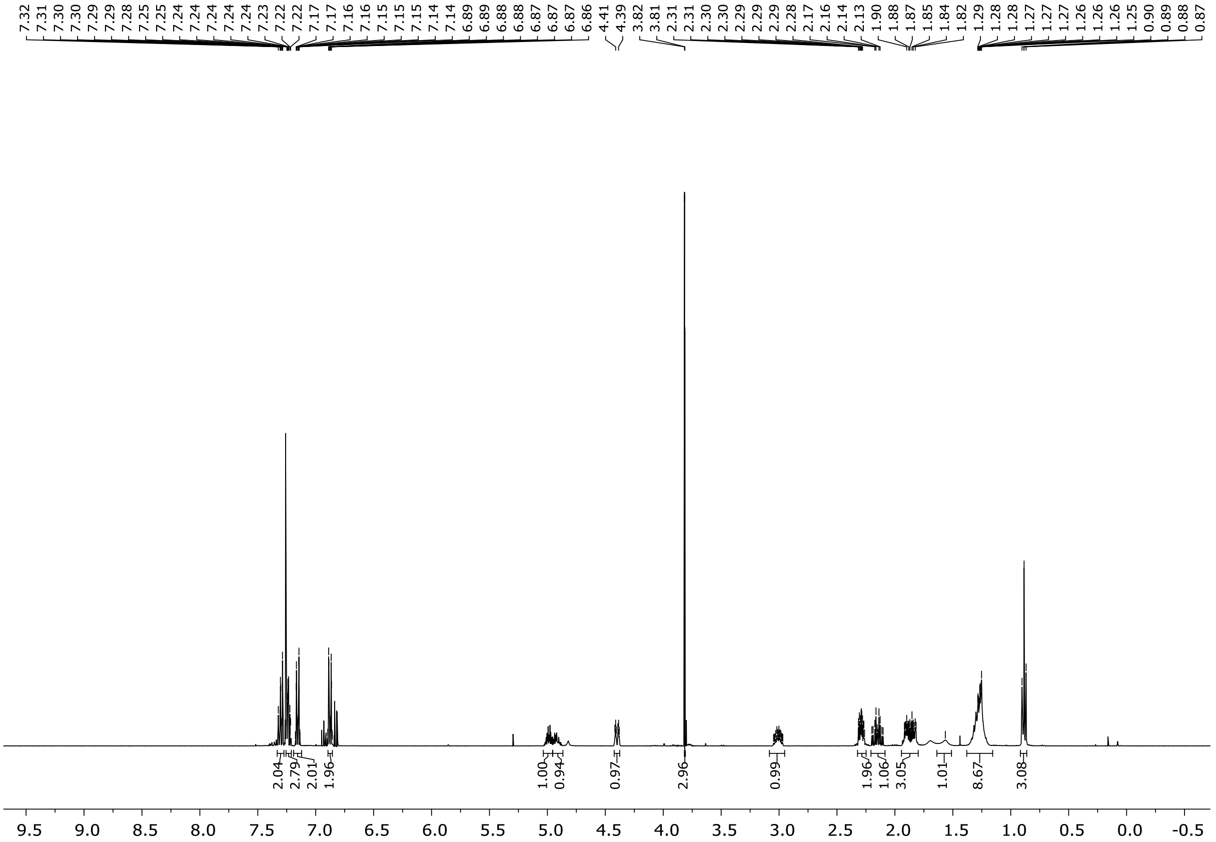

syn-4-methyl-2-vinyltetrahydro-2H-pyran 5

^{1}H-NMR

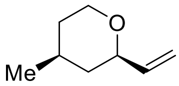

$C_8H_{14}O$
126.19

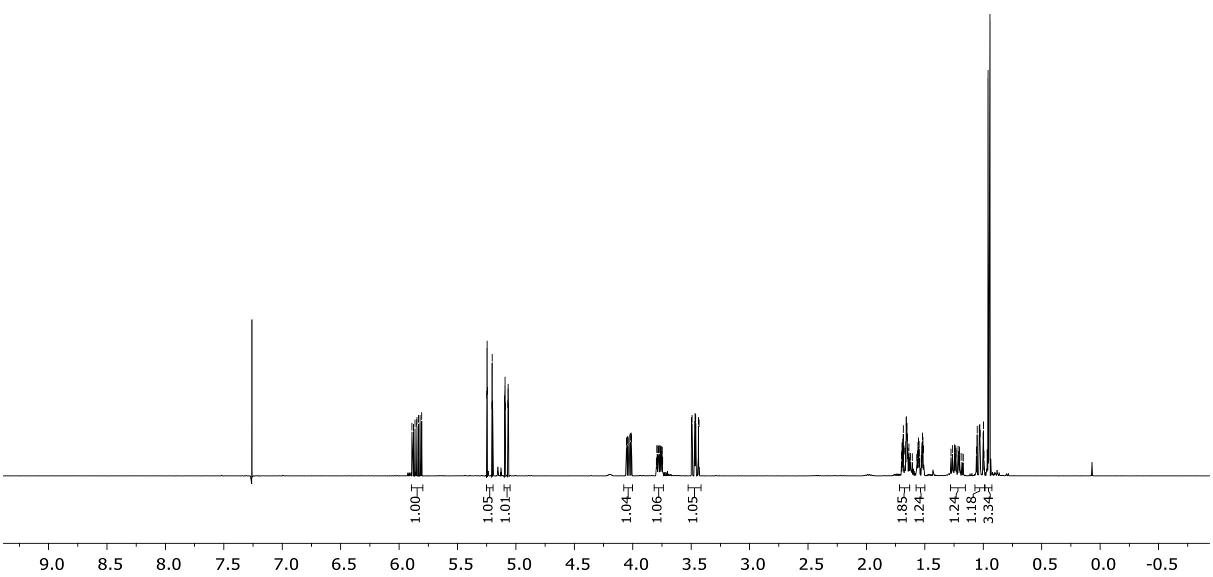

^{13}C-NMR

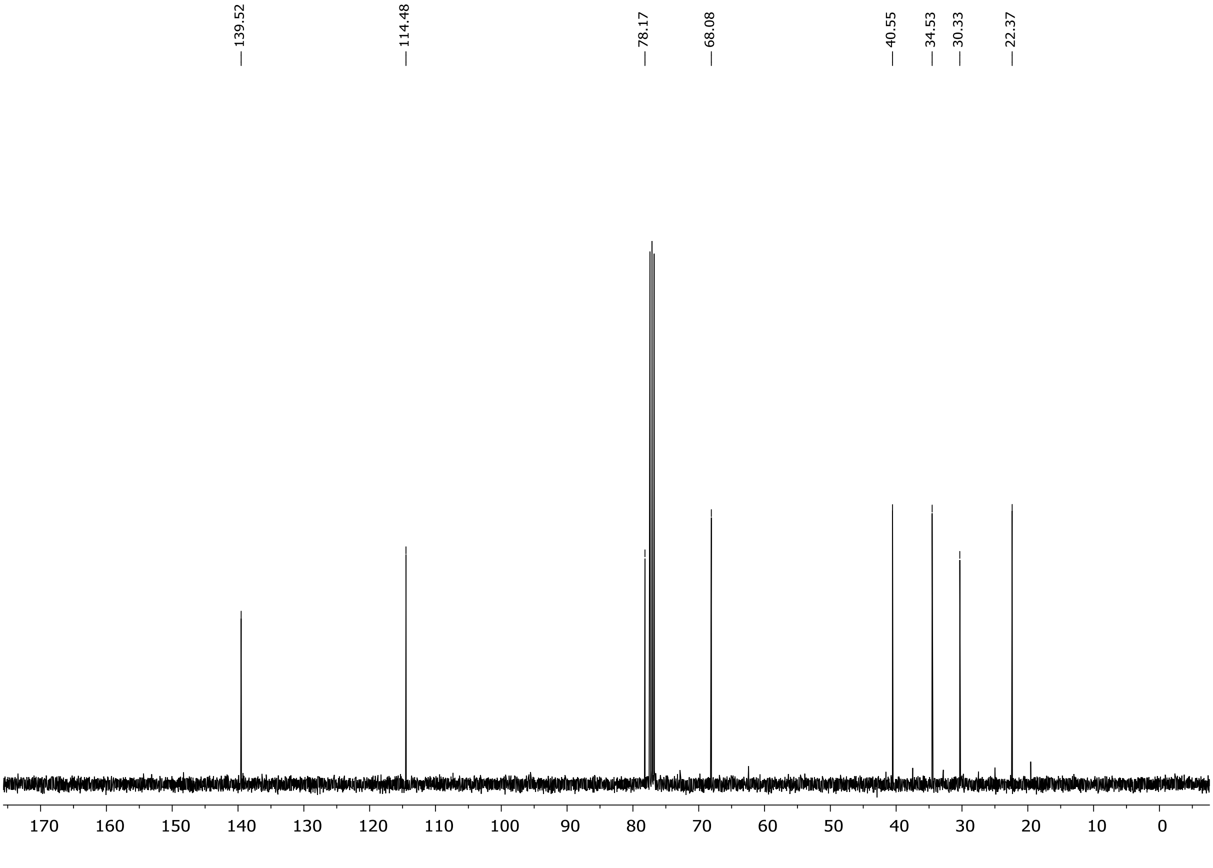

syn-4-dodecyl-2-vinyltetrahydro-2H-pyran 6

^{1}H-NMR

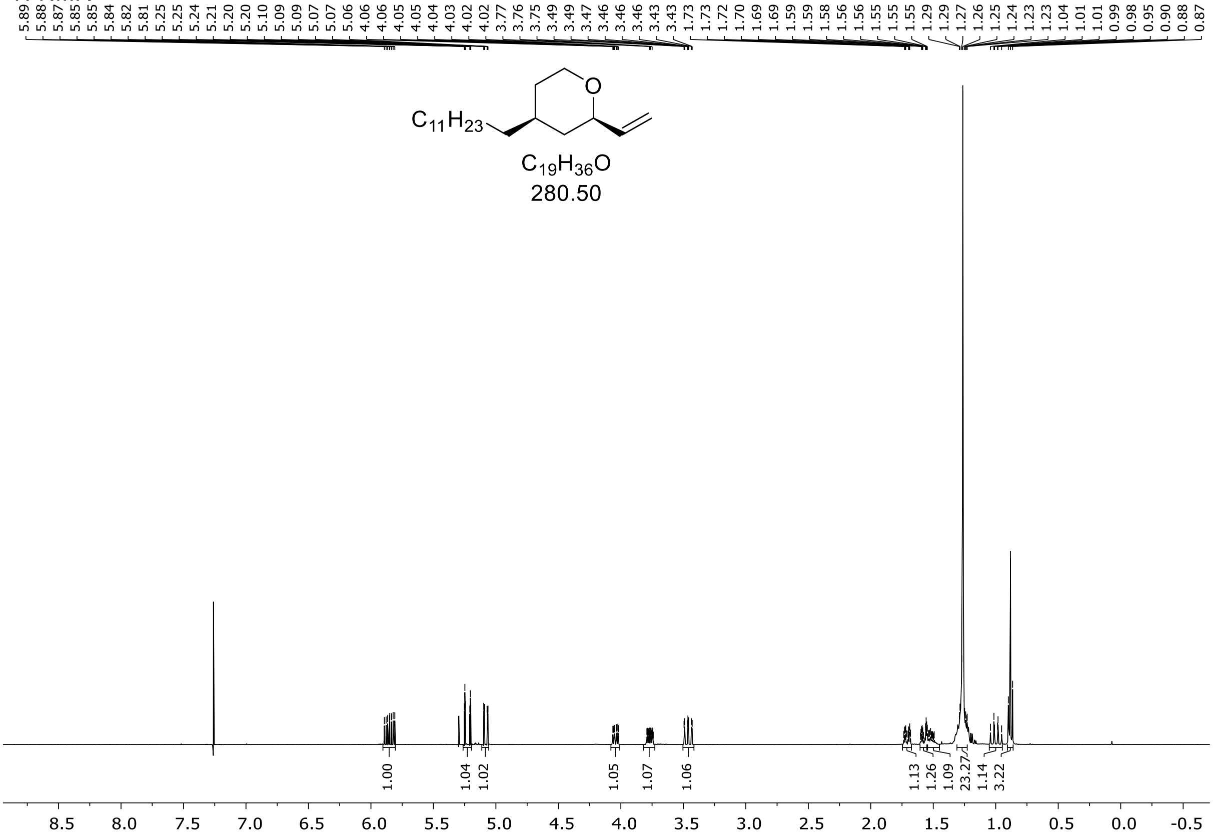

^{13}C-NMR

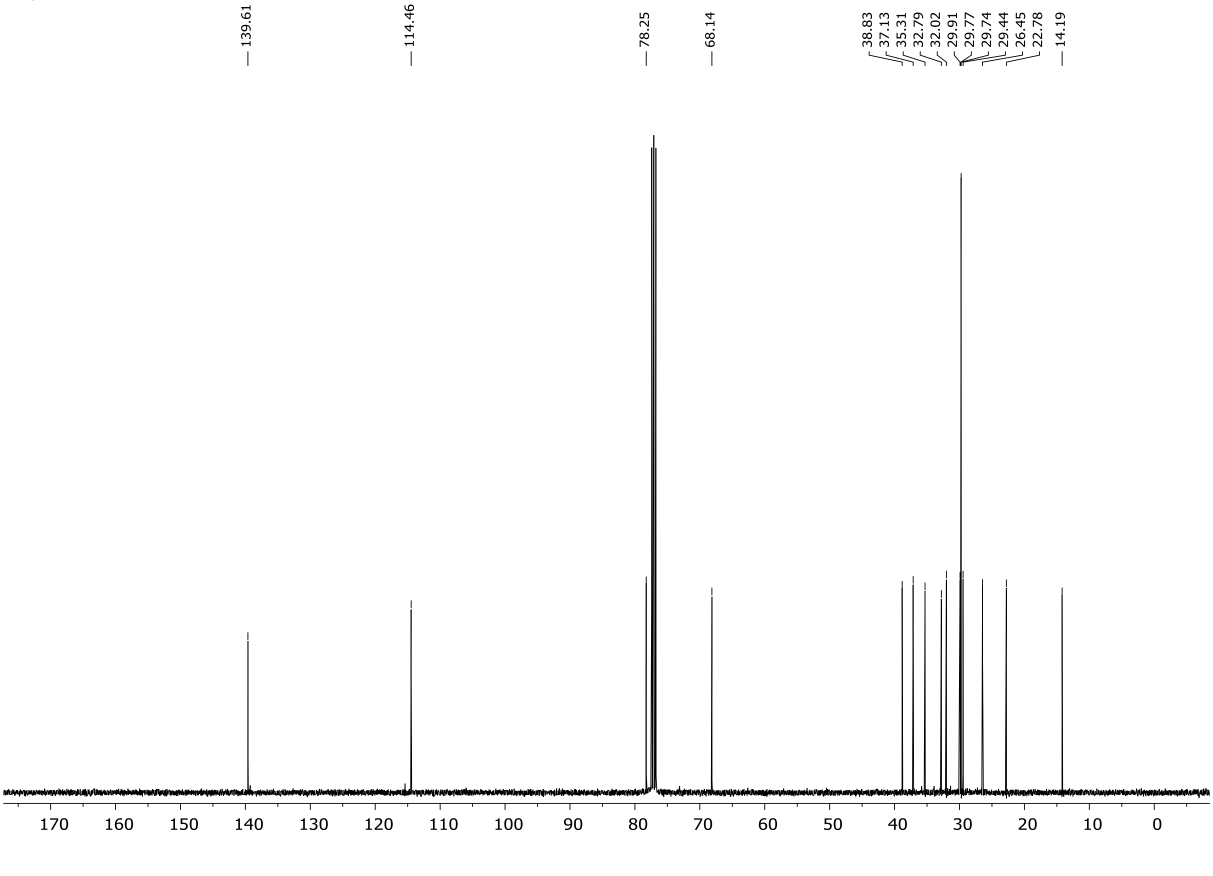

syn-4-neopentyl-2-vinyltetrahydro-2H-pyran 7

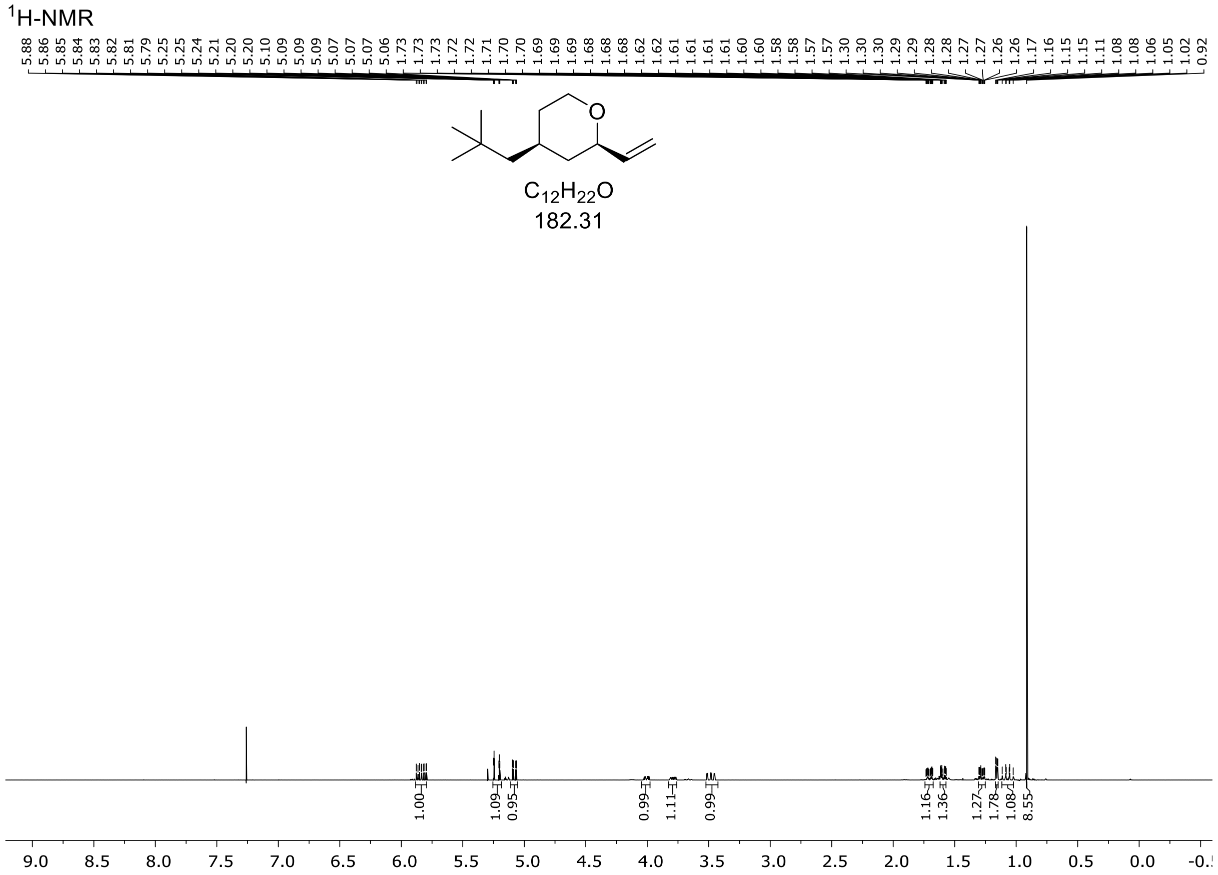

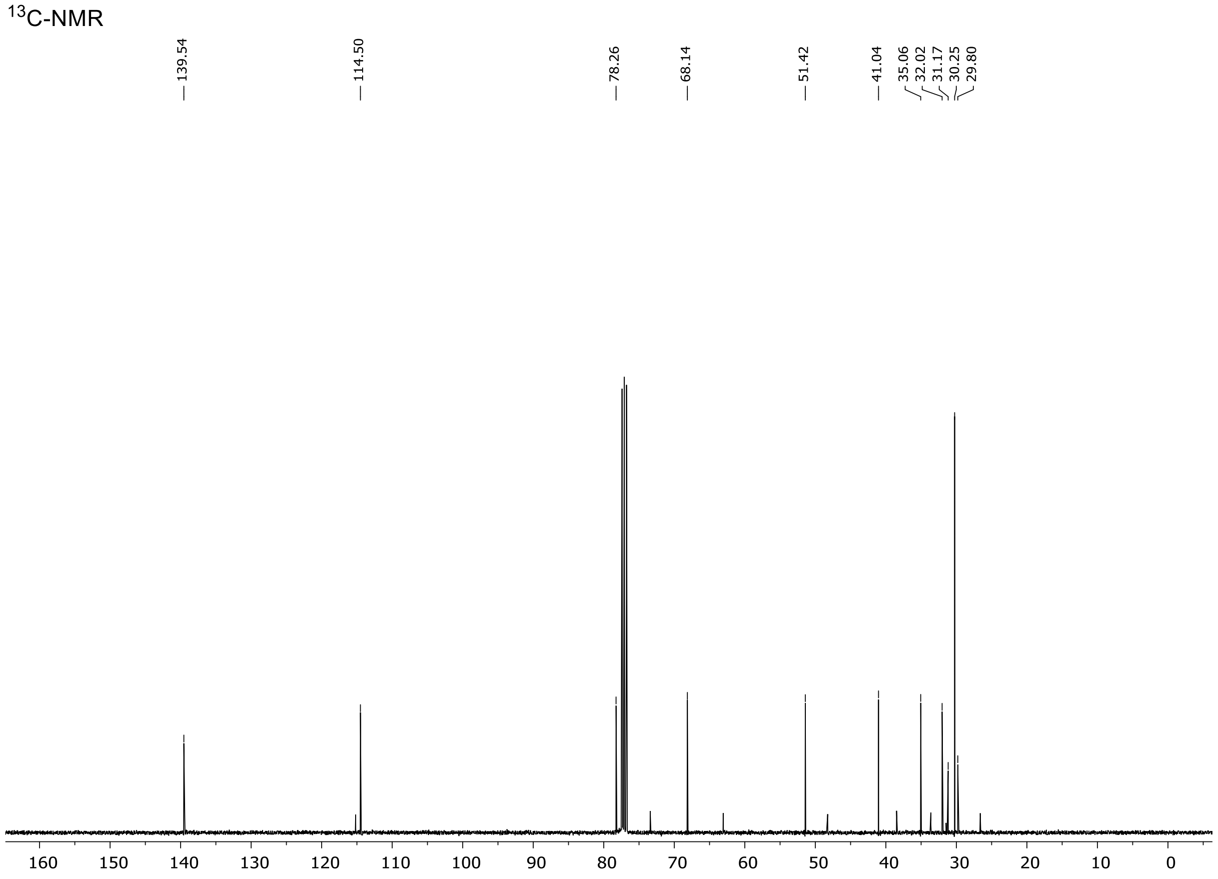

syn-4-cyclopropyl-2-vinyltetrahydro-2H-pyran 8

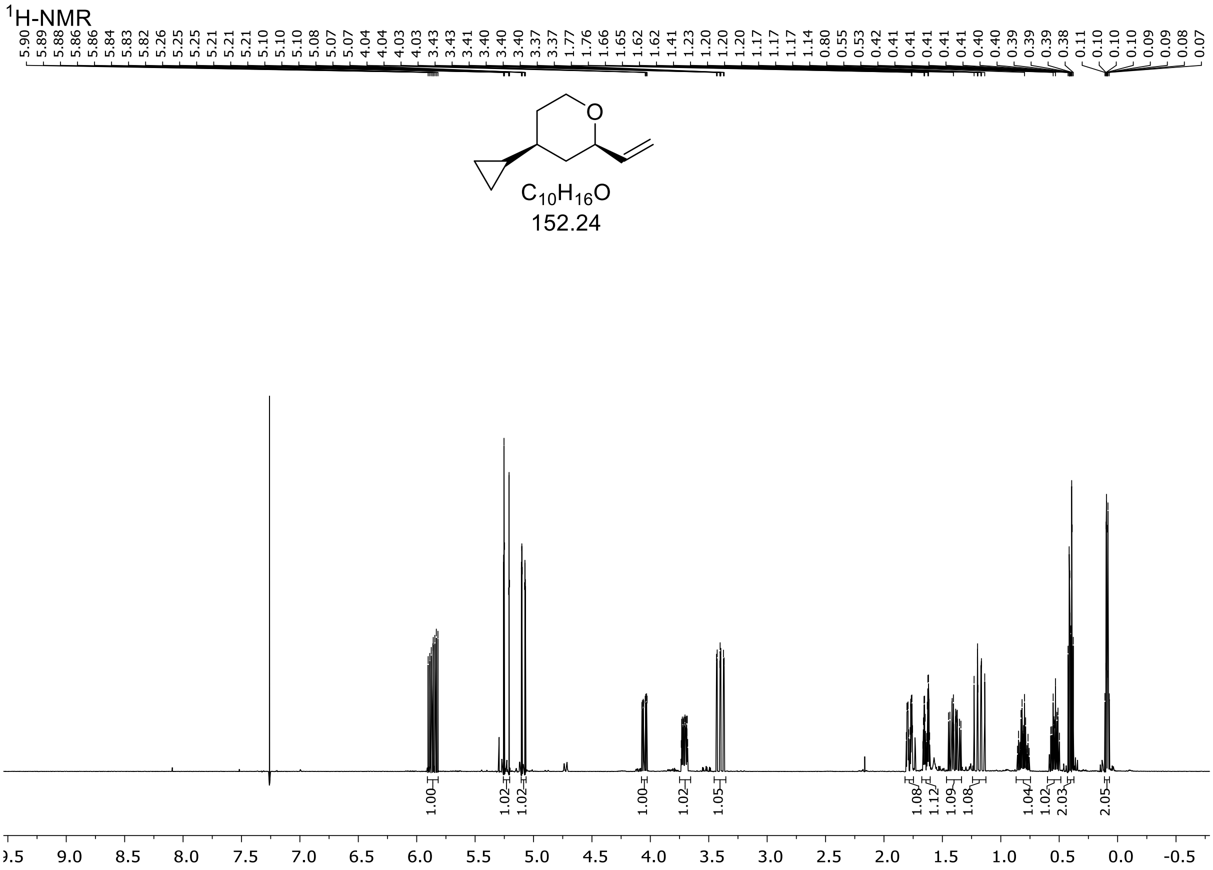

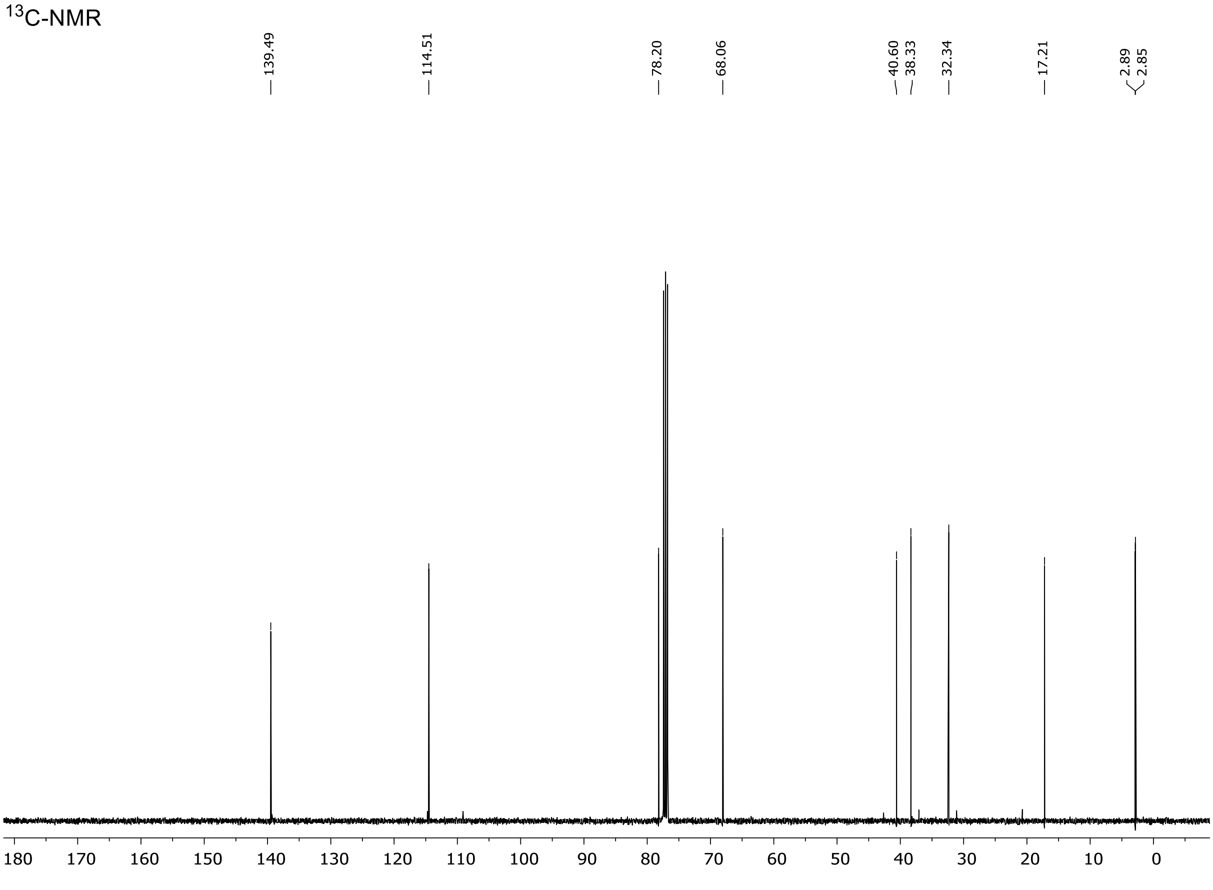

syn-4-cyclohexyl-2-vinyltetrahydro-2H-pyran 9

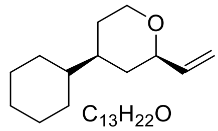

$C_{13}H_{22}O$
194.32

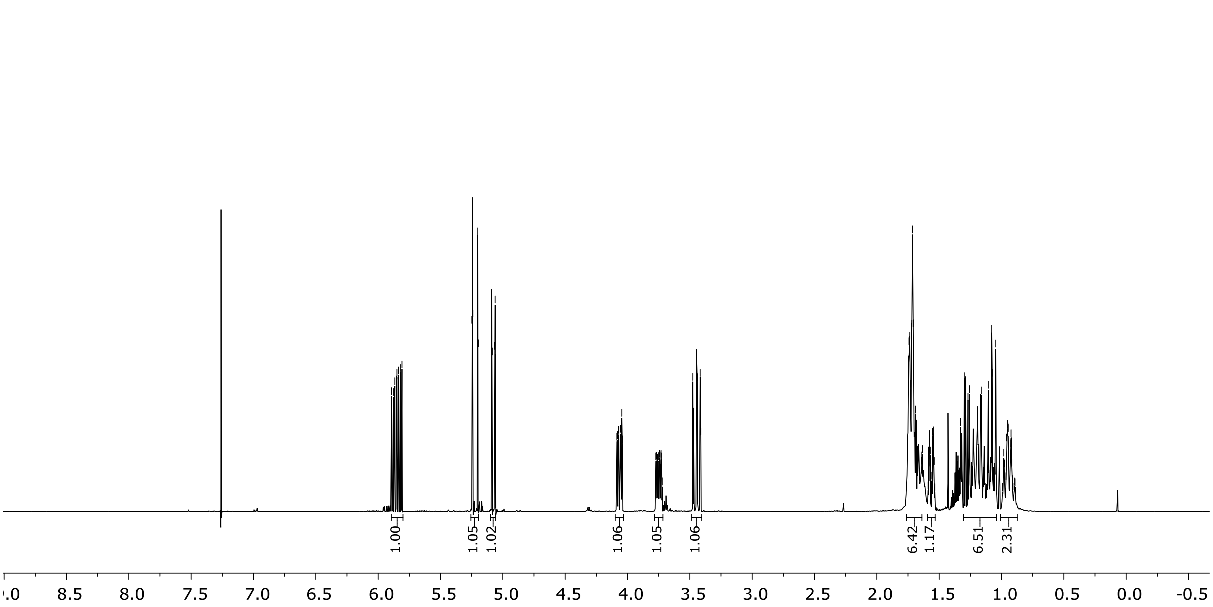

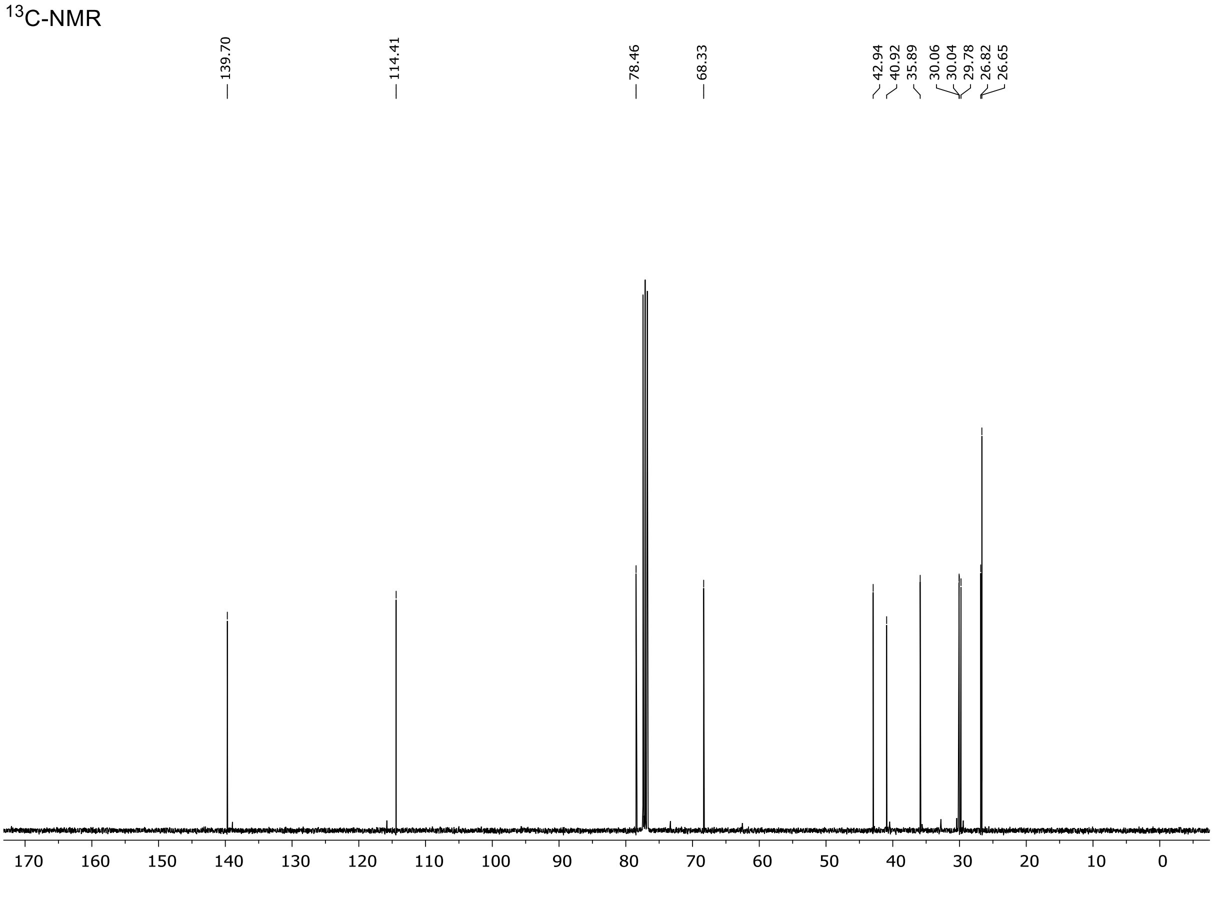

syn-4-(3-phenylpropyl)-2-vinyltetrahydro-2H-pyran 10

^{1}H-NMR

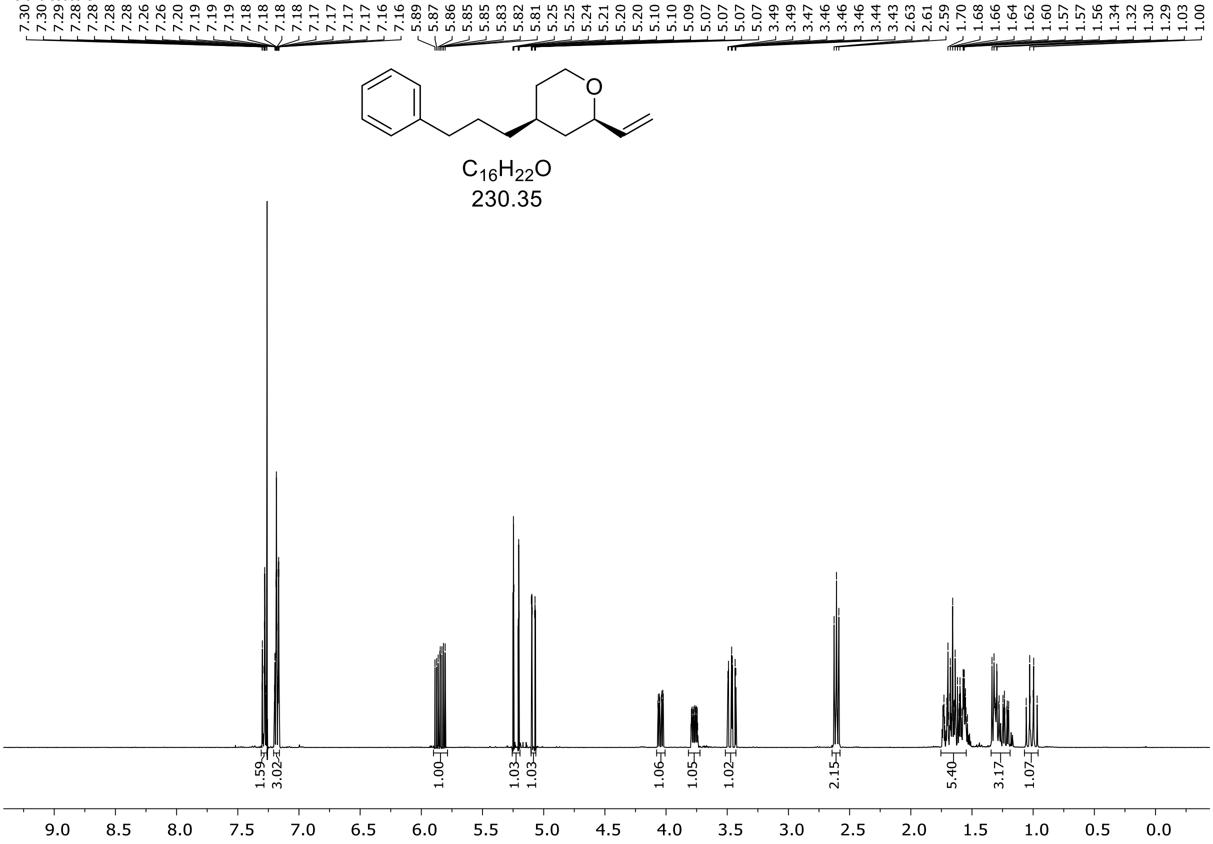

^{13}C-NMR

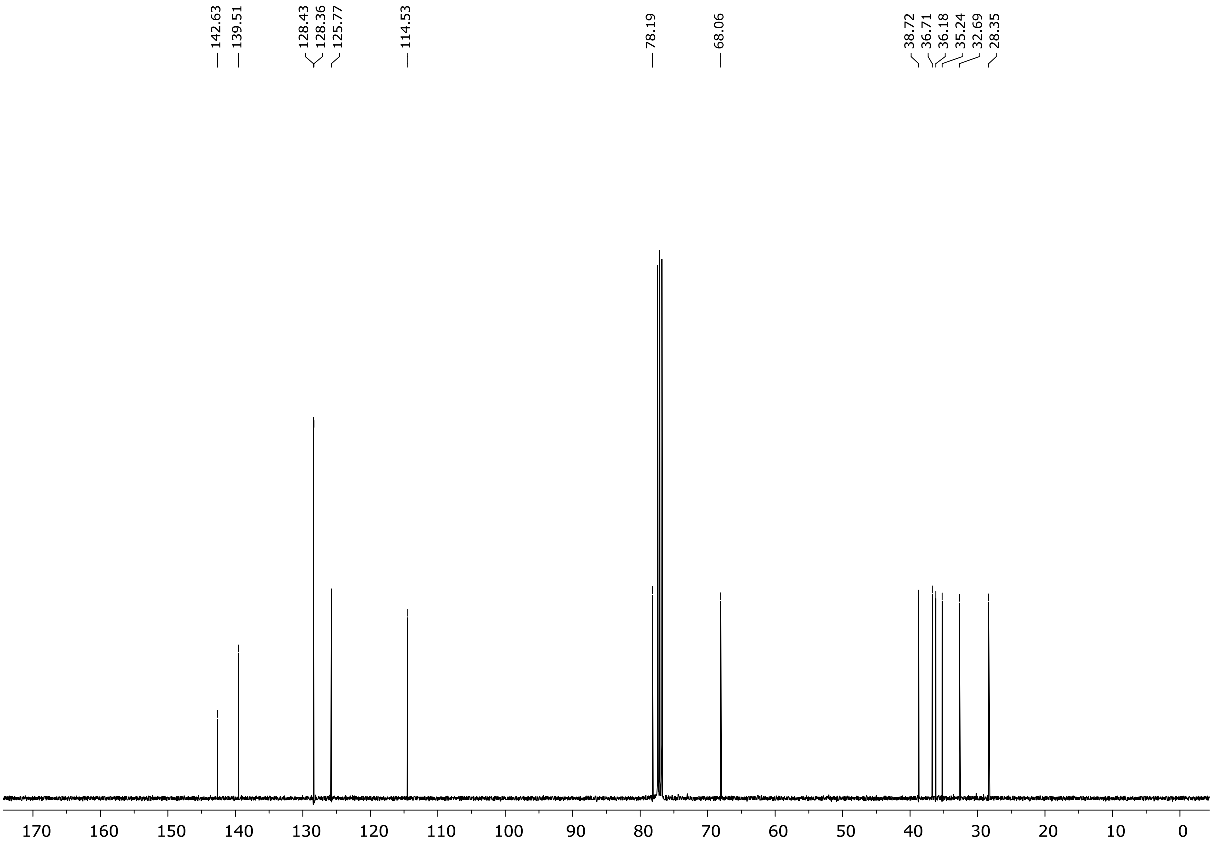

syn-4-phenyl-2-vinyltetrahydro-2H-pyran 2

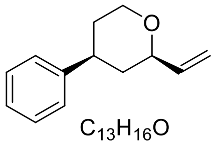

$C_{13}H_{16}O$
188.27

^{1}H-NMR

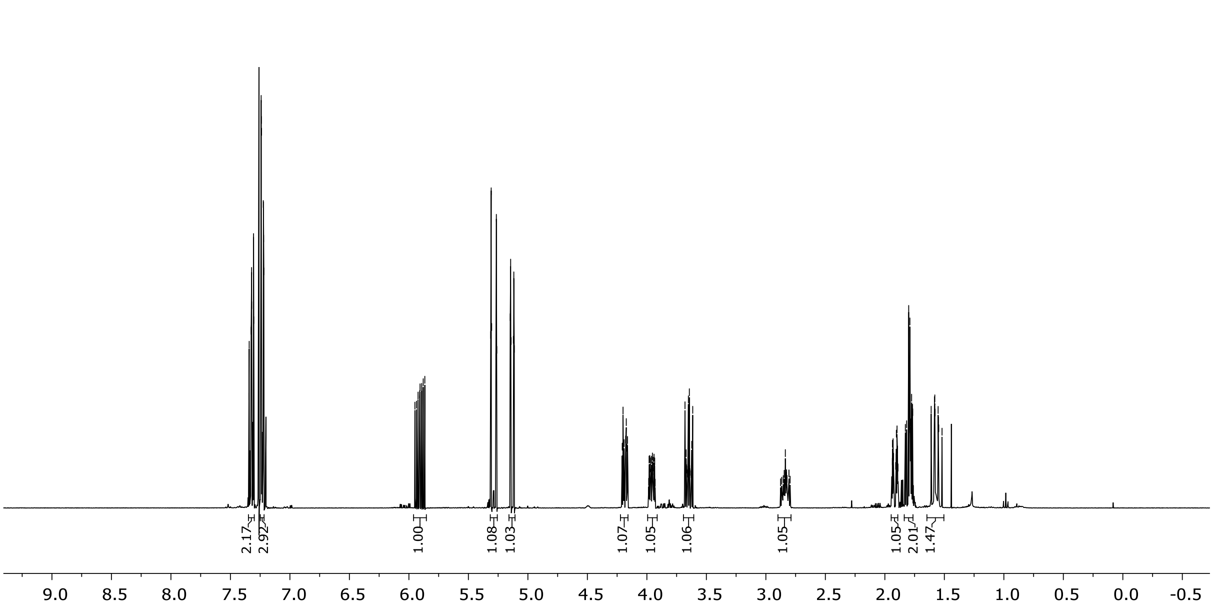

^{13}C-NMR

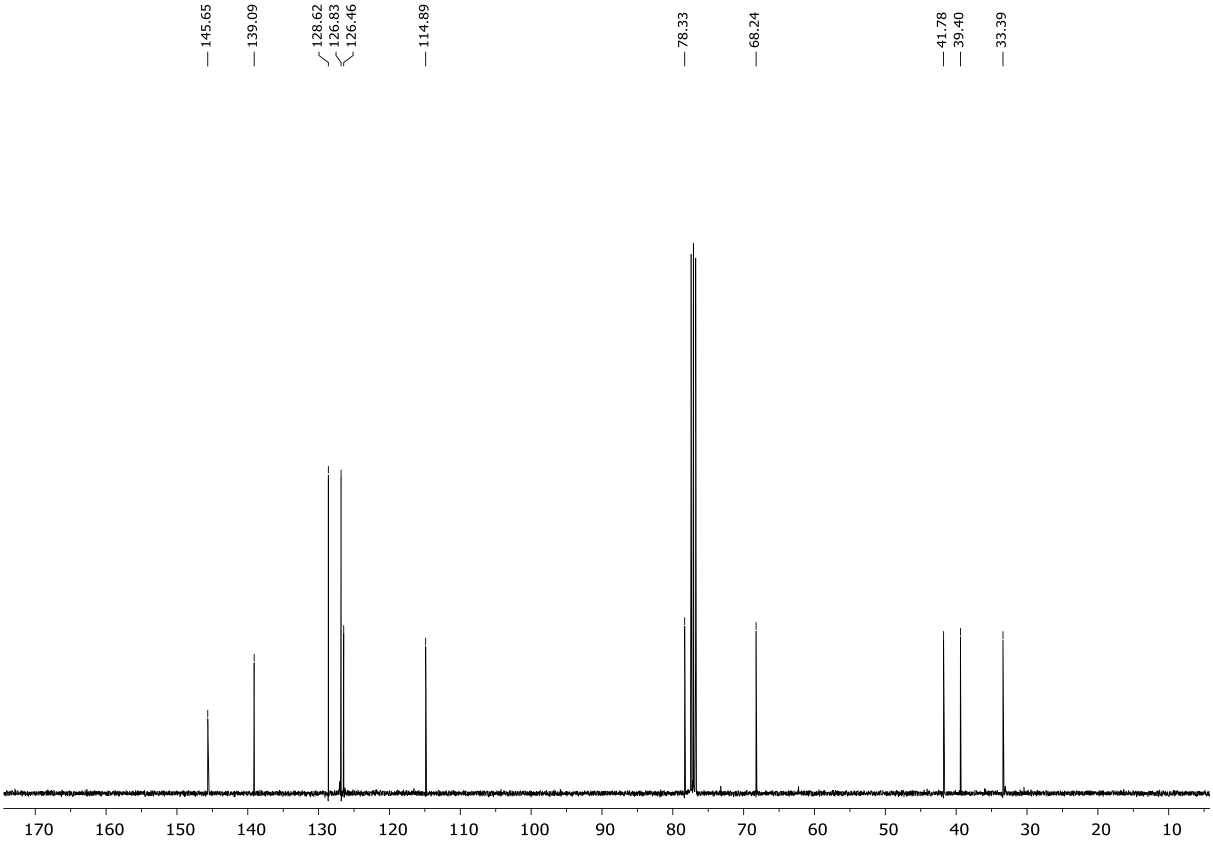

syn-4-(naphthalen-2-yl)-2-vinyltetrahydro-2H-pyran 11

^{1}H-NMR

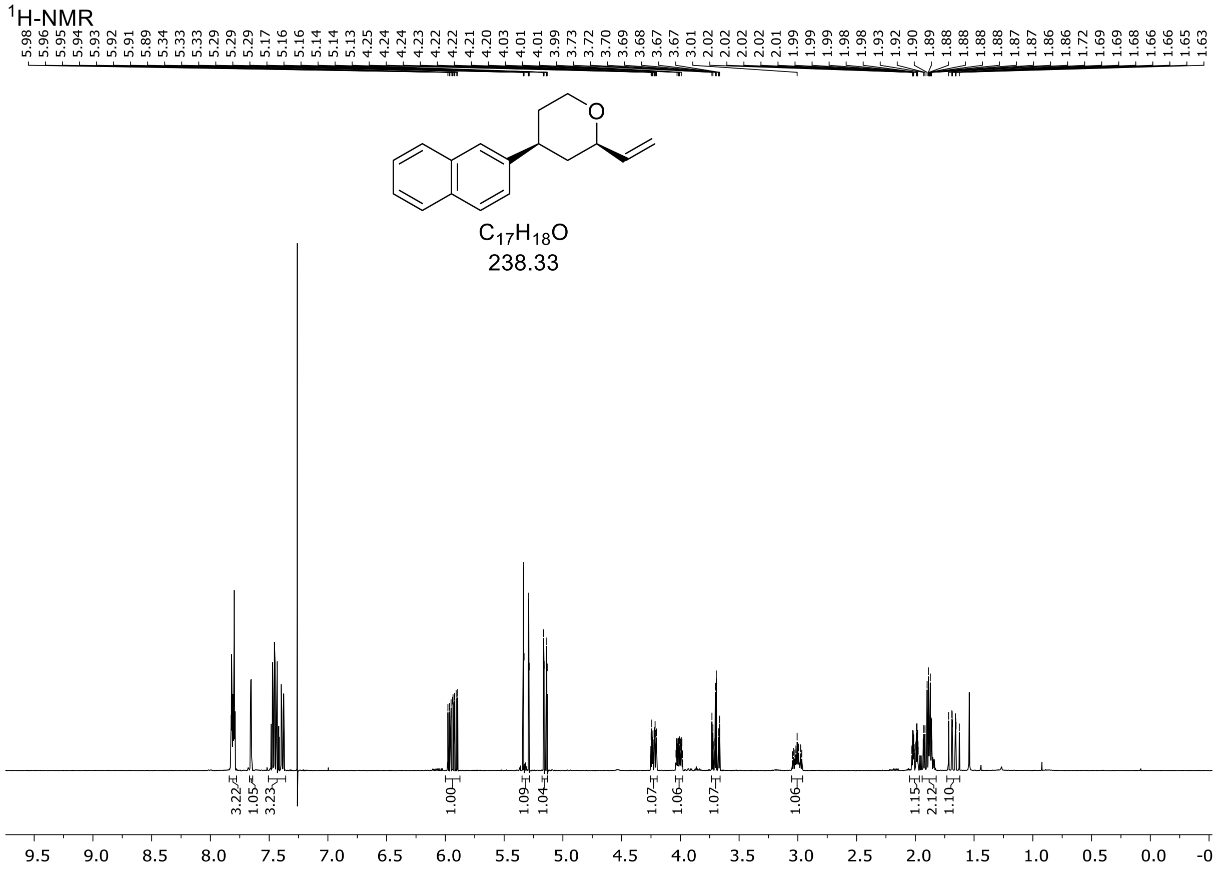

^{13}C-NMR

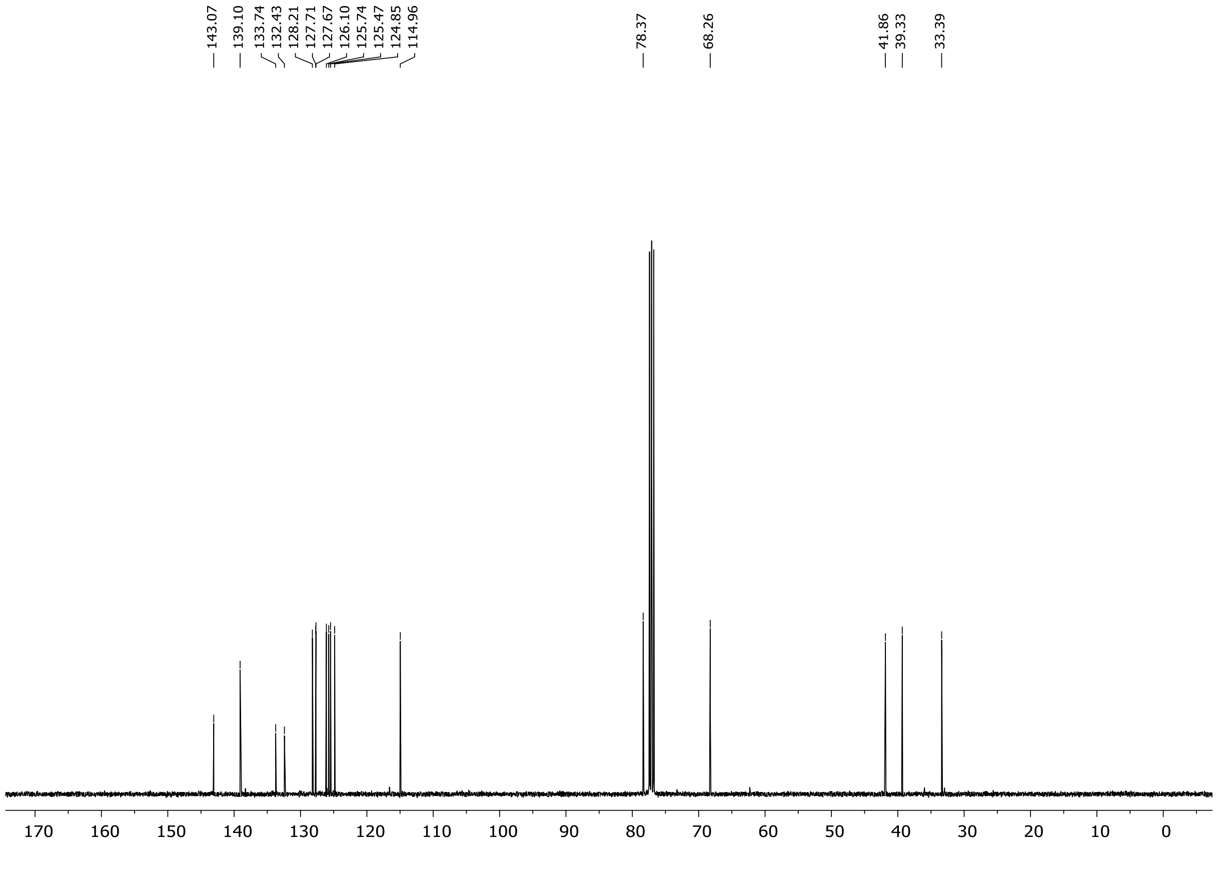

syn-4-([1,1'-biphenyl]-4-yl)-2-vinyltetrahydro-2H-pyran 12

^{1}H-NMR

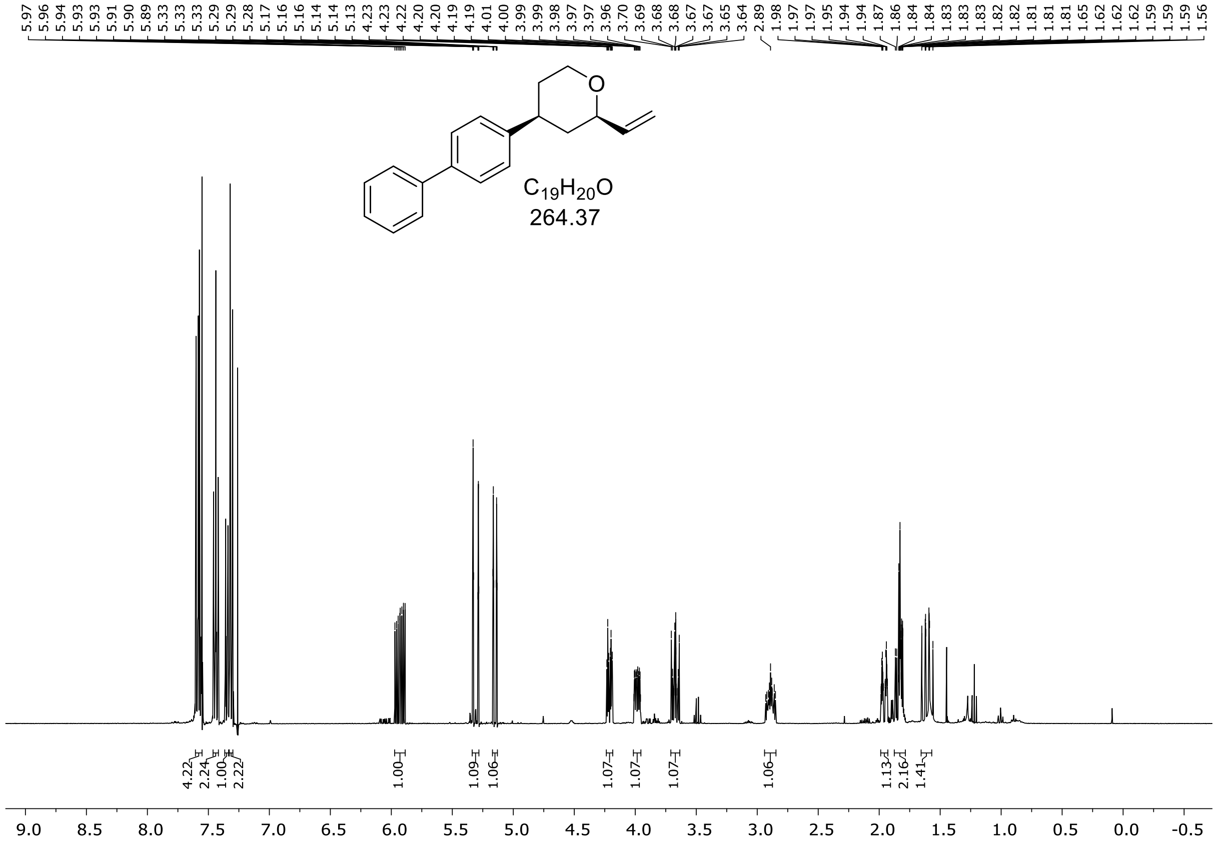

$C_{19}H_{20}O$
264.37

^{13}C-NMR

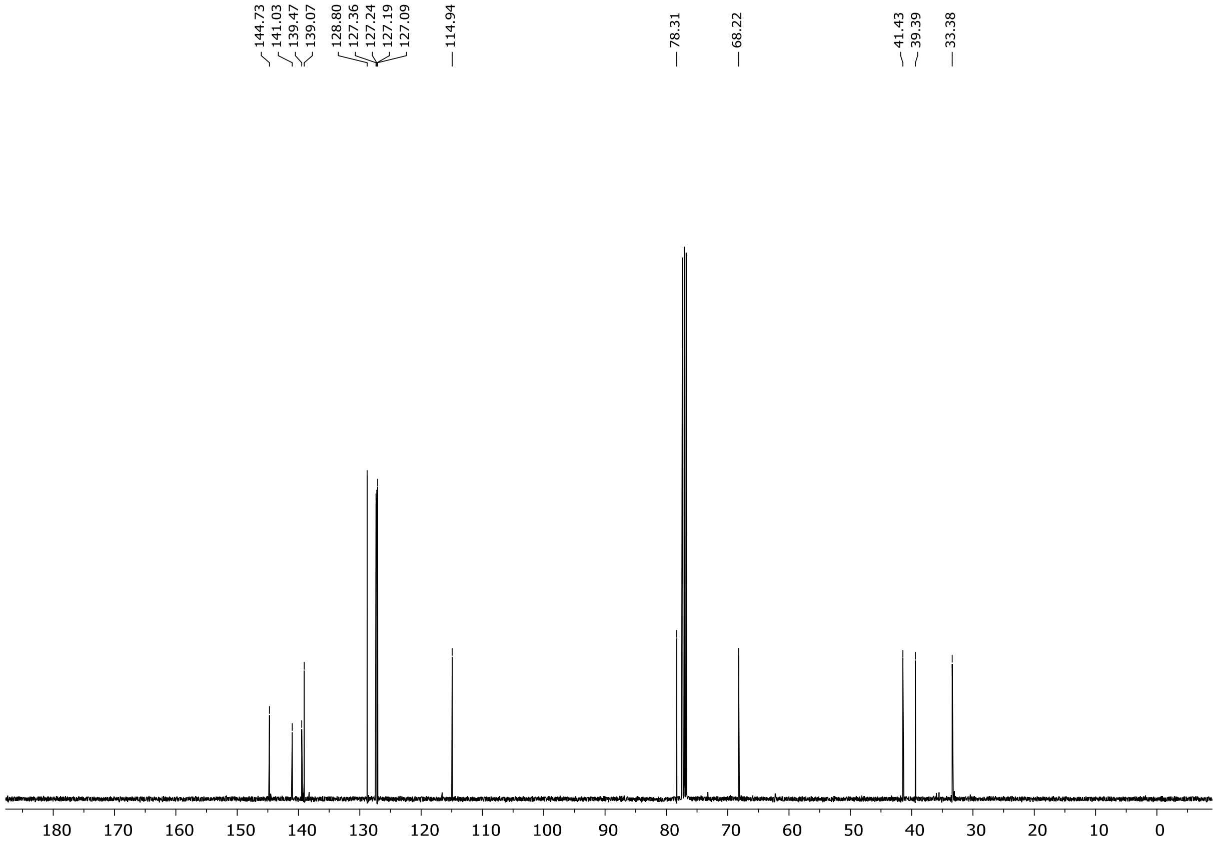

syn-4-(p-tolyl)-2-vinyltetrahydro-2H-pyran 13

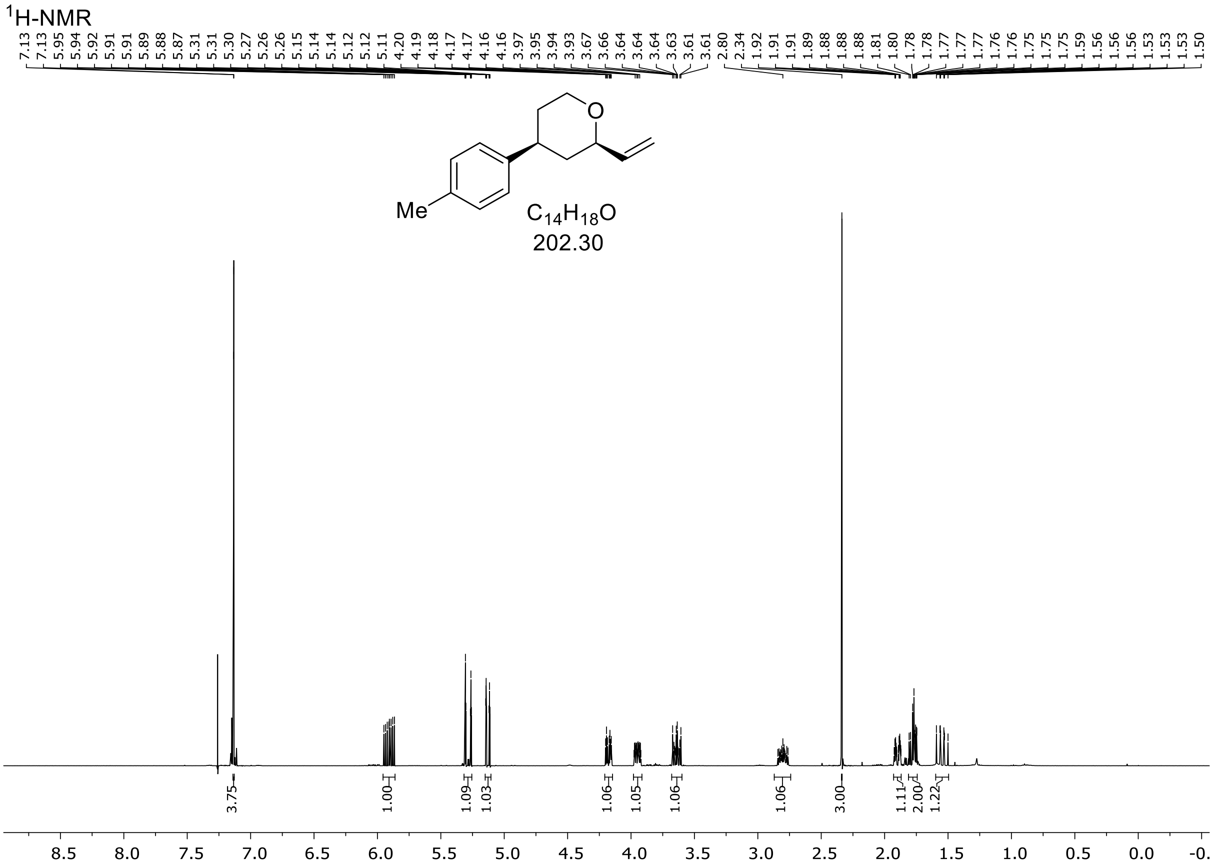

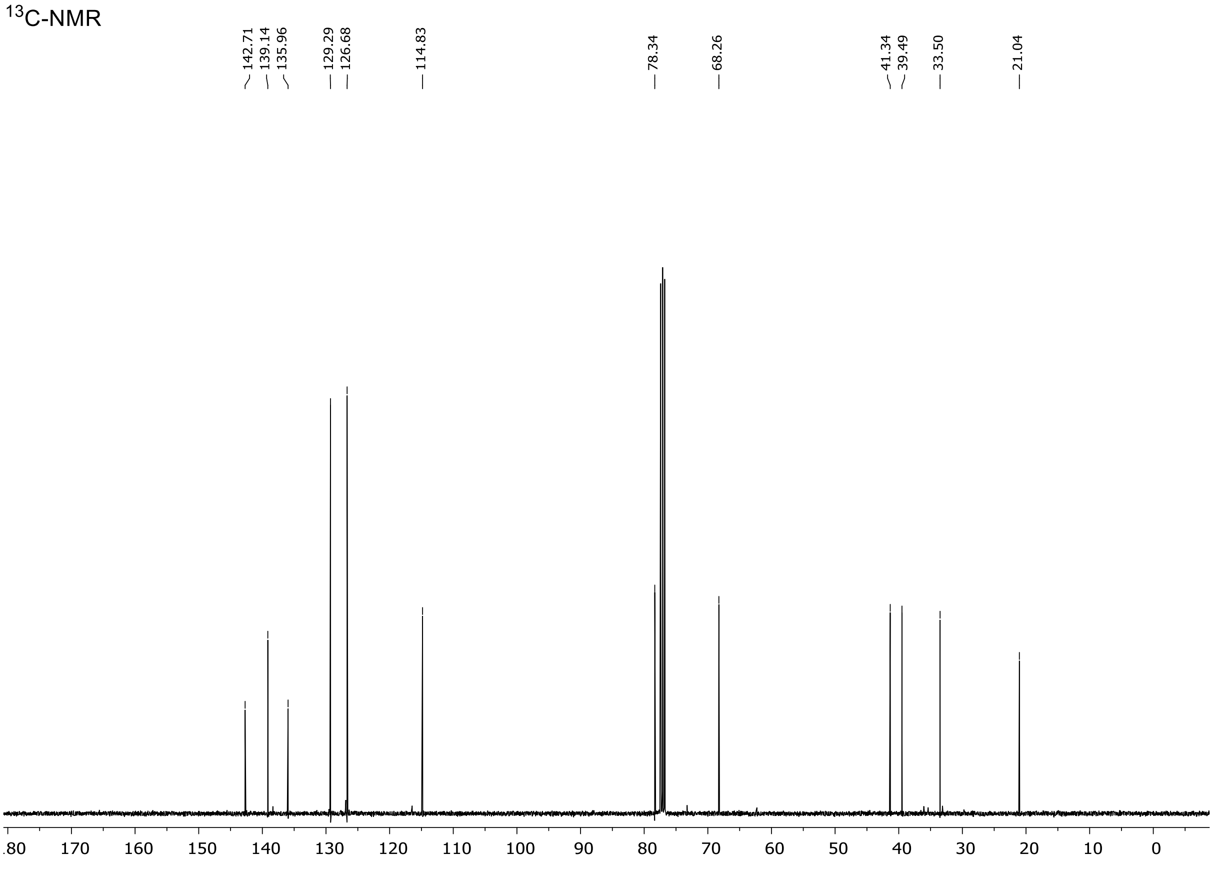

syn-4-(m-tolyl)-2-vinyltetrahydro-2H-pyran 14

^{1}H-NMR

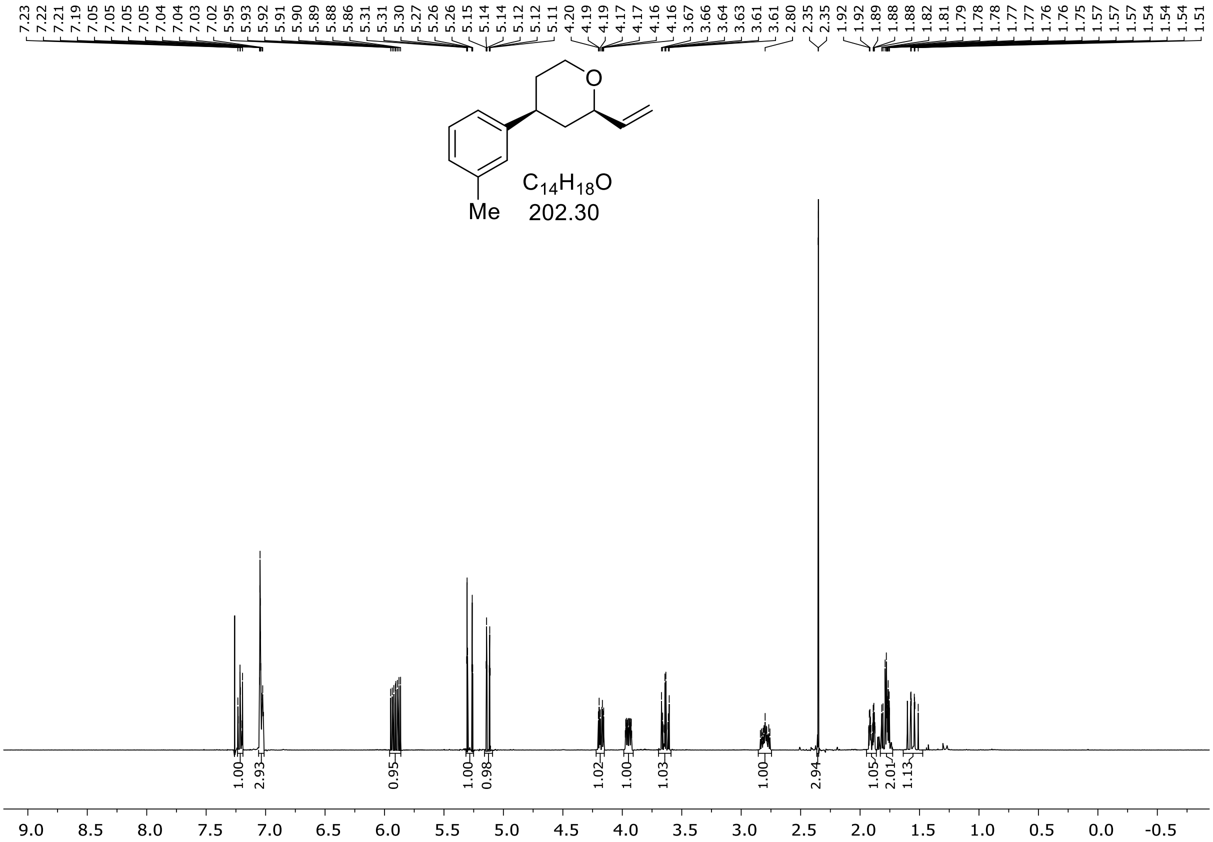

^{13}C-NMR

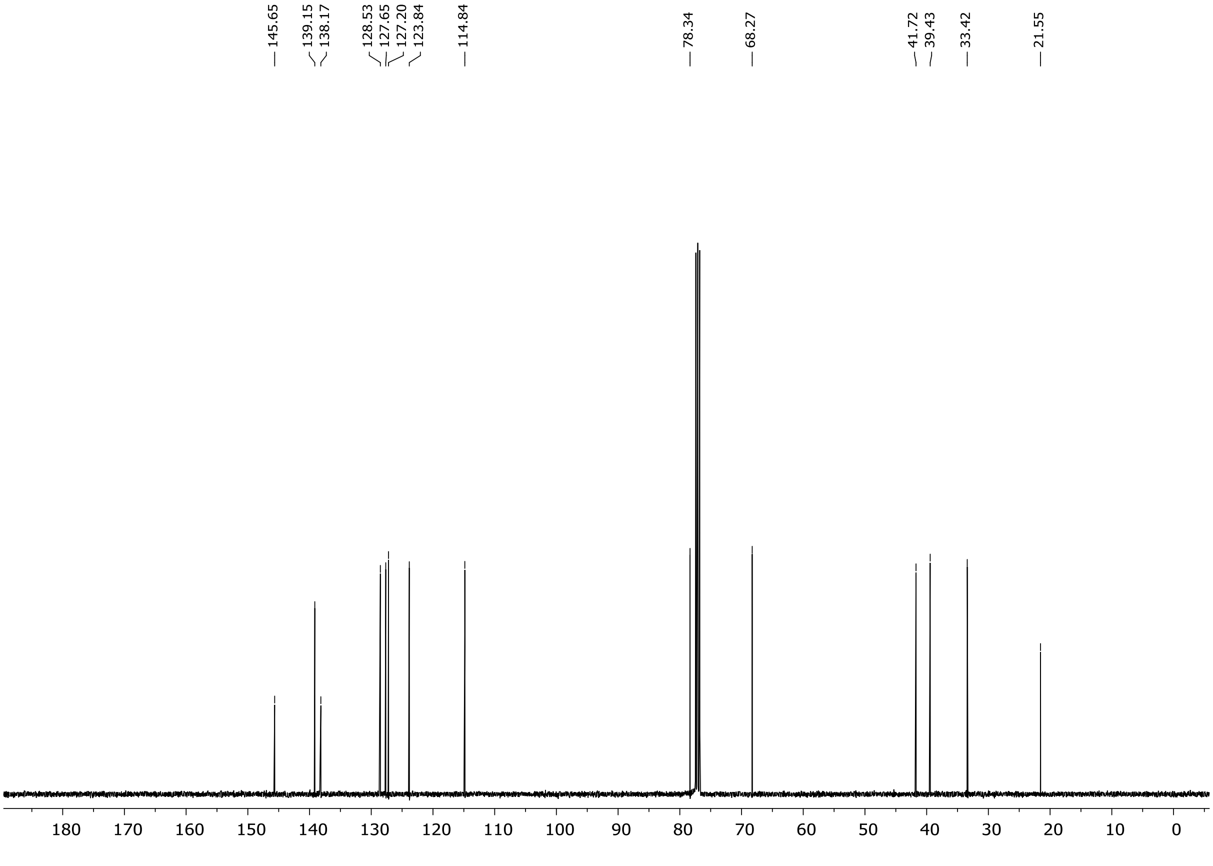

syn-4-(o-tolyl)-2-vinyltetrahydro-2H-pyran 15

^{1}H-NMR

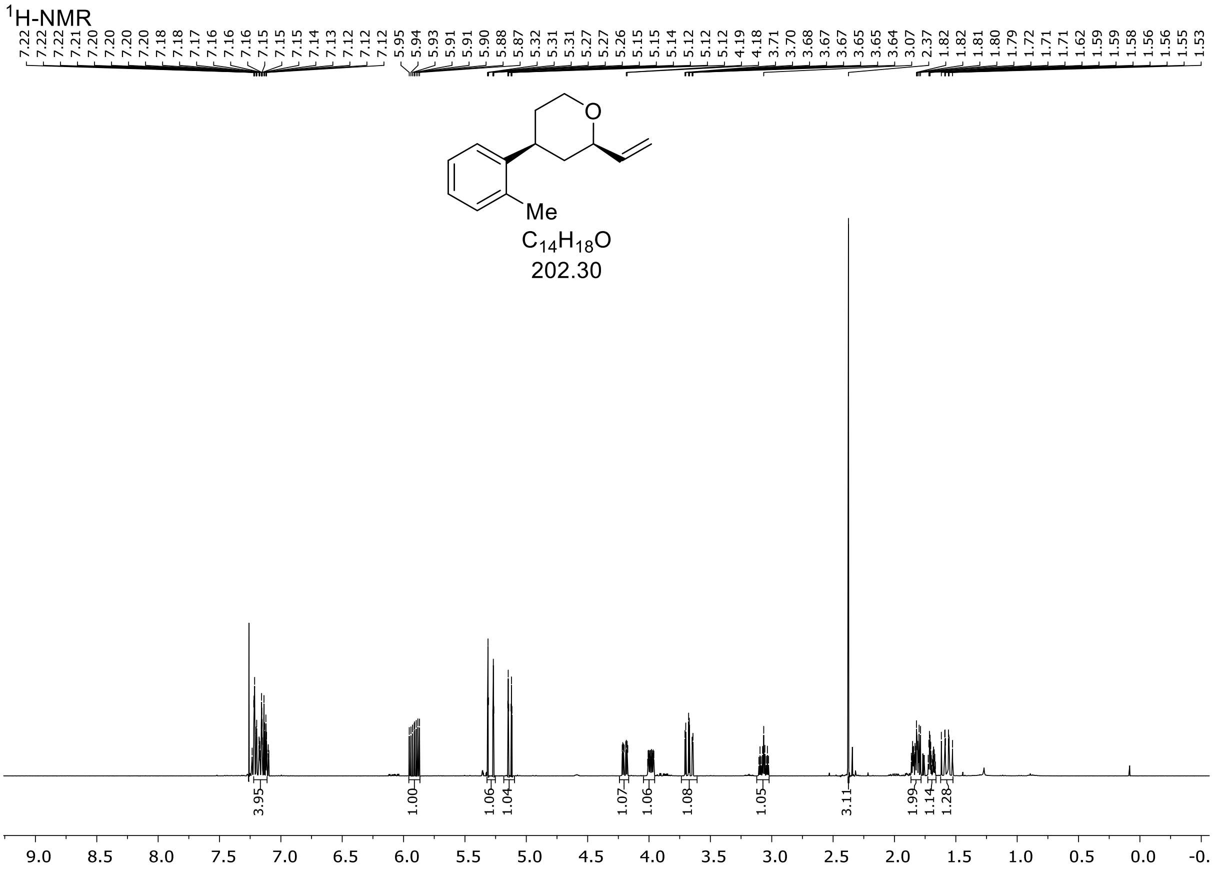

^{13}C-NMR

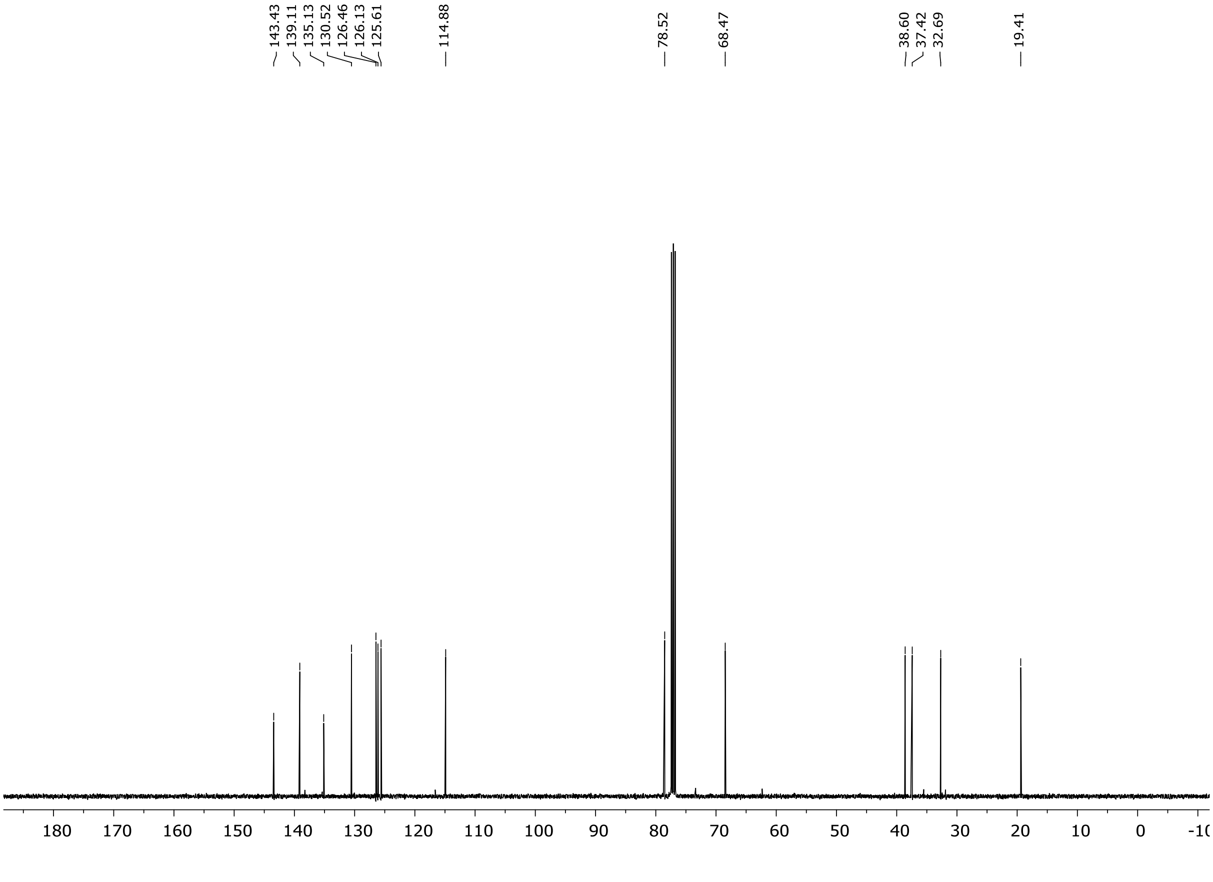

syn-4-mesityl-2-vinyltetrahydro-2H-pyran 16

^{1}H-NMR

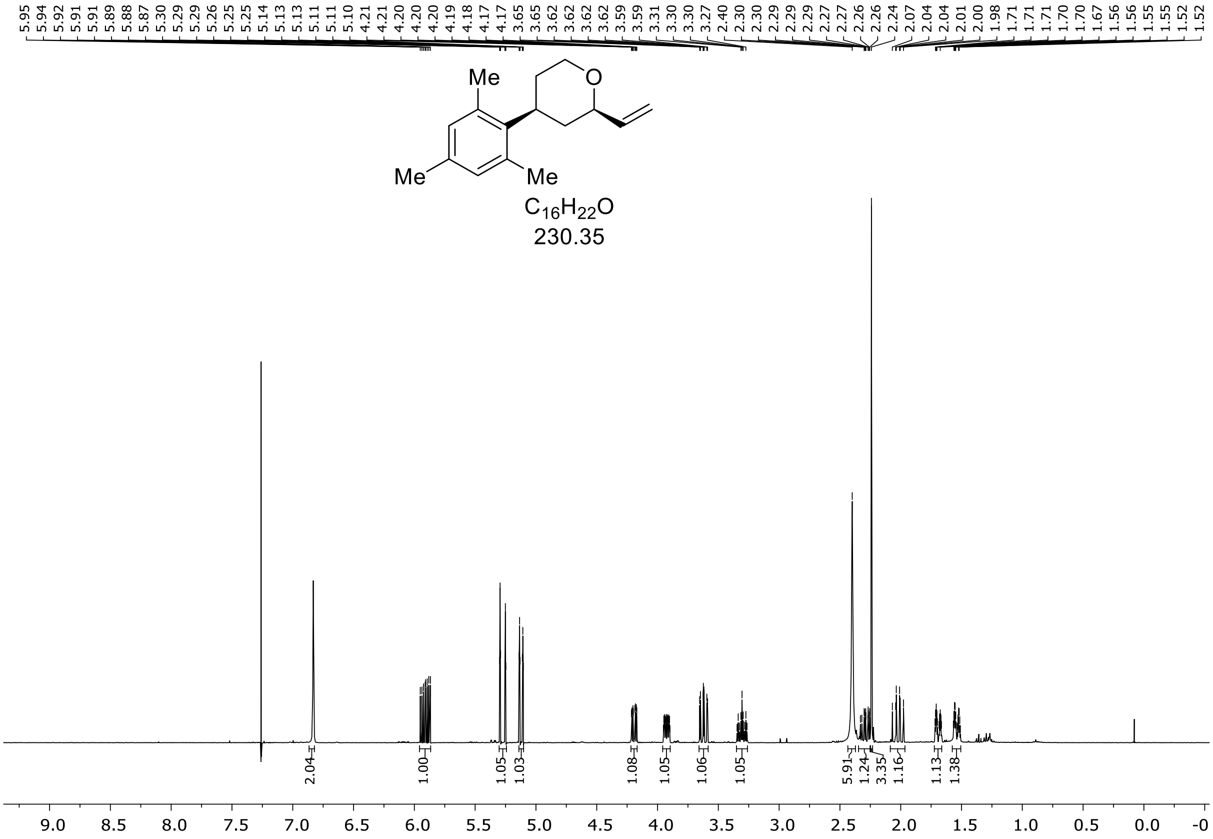

^{13}C-NMR

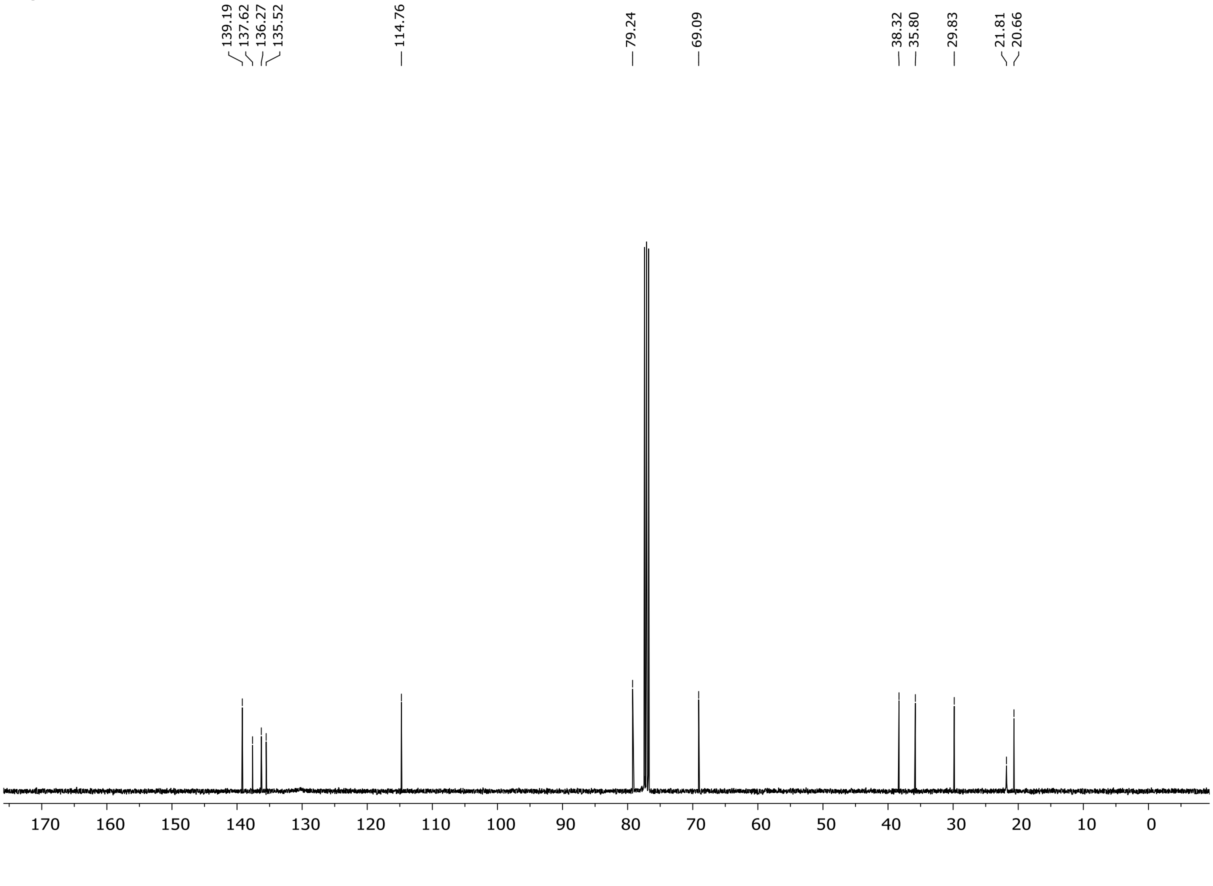

syn-2-vinyl-4-(4-vinylphenyl)tetrahydro-2H-pyran 17

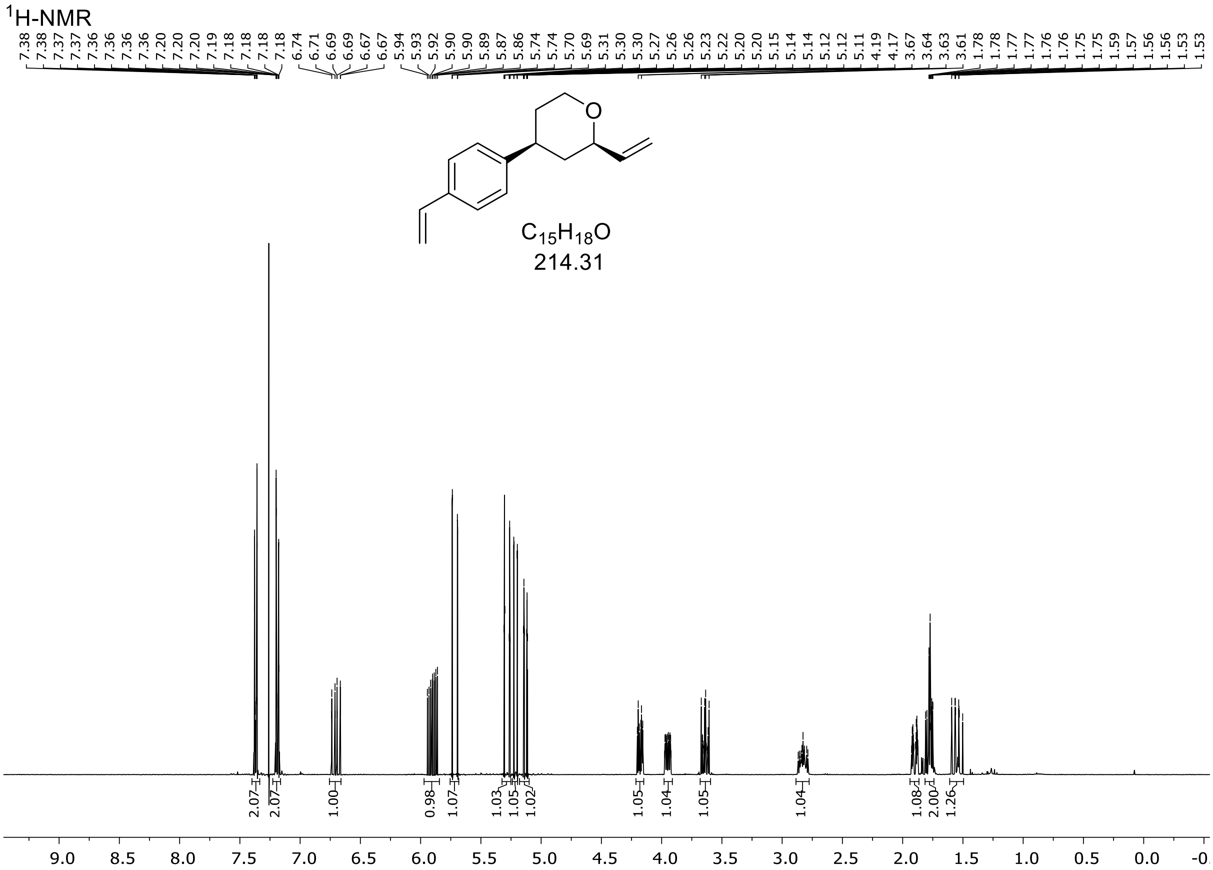

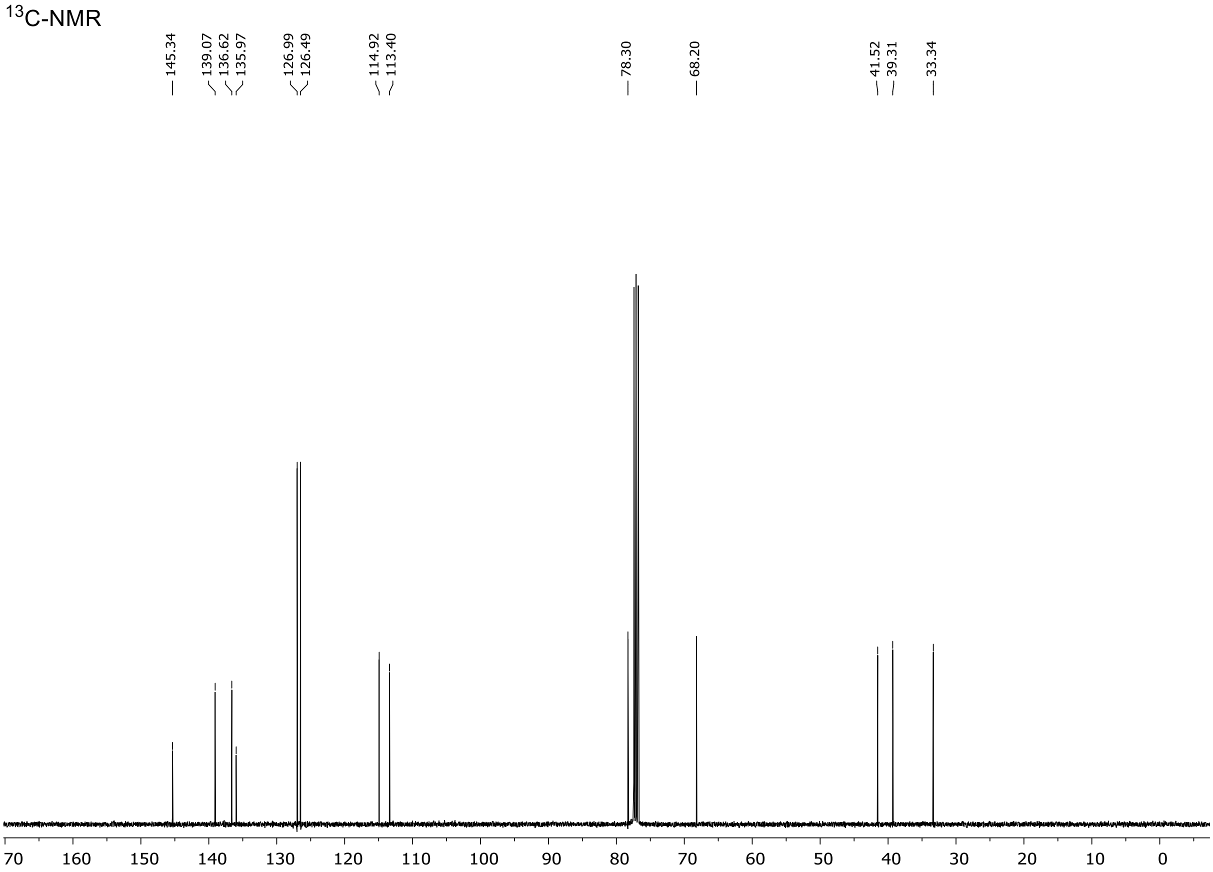

syn-4-(4-(trifluoromethyl)phenyl)-2-vinyltetrahydro-2H-pyran 18

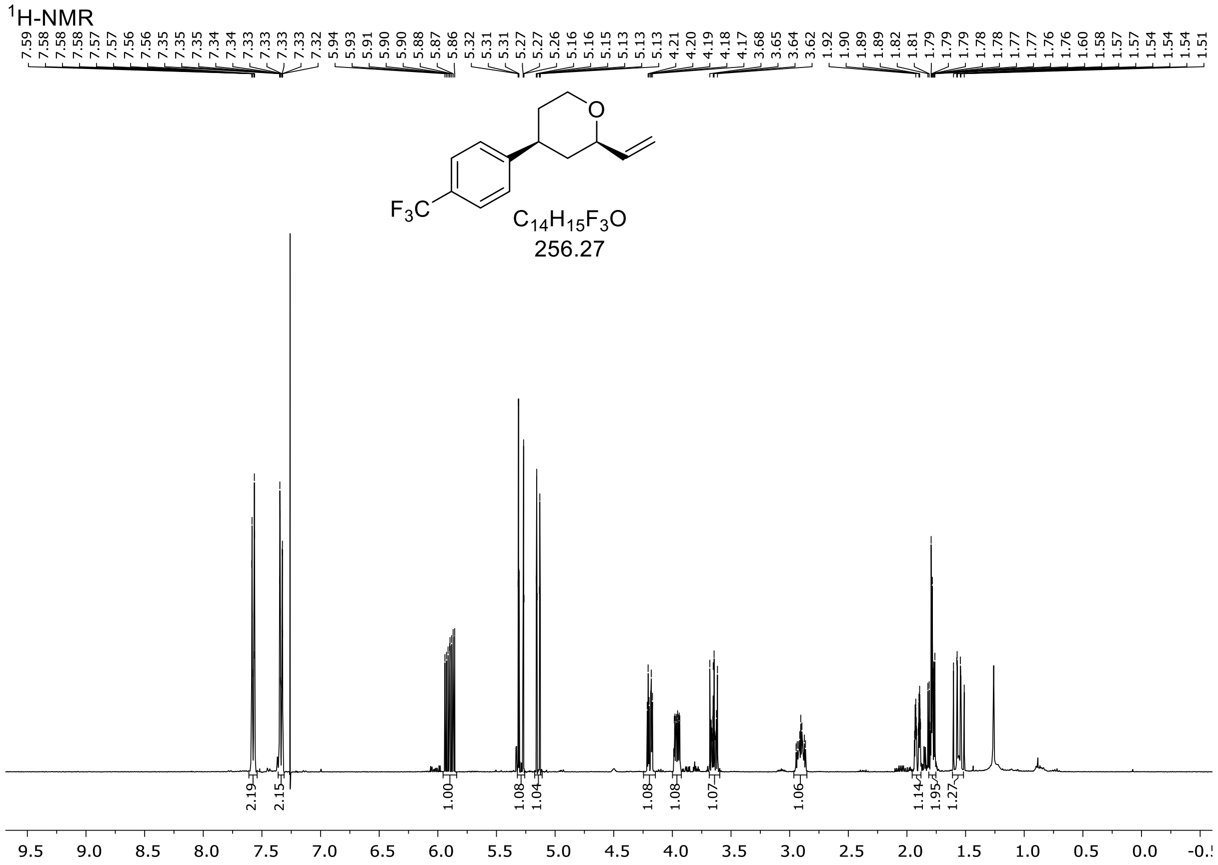

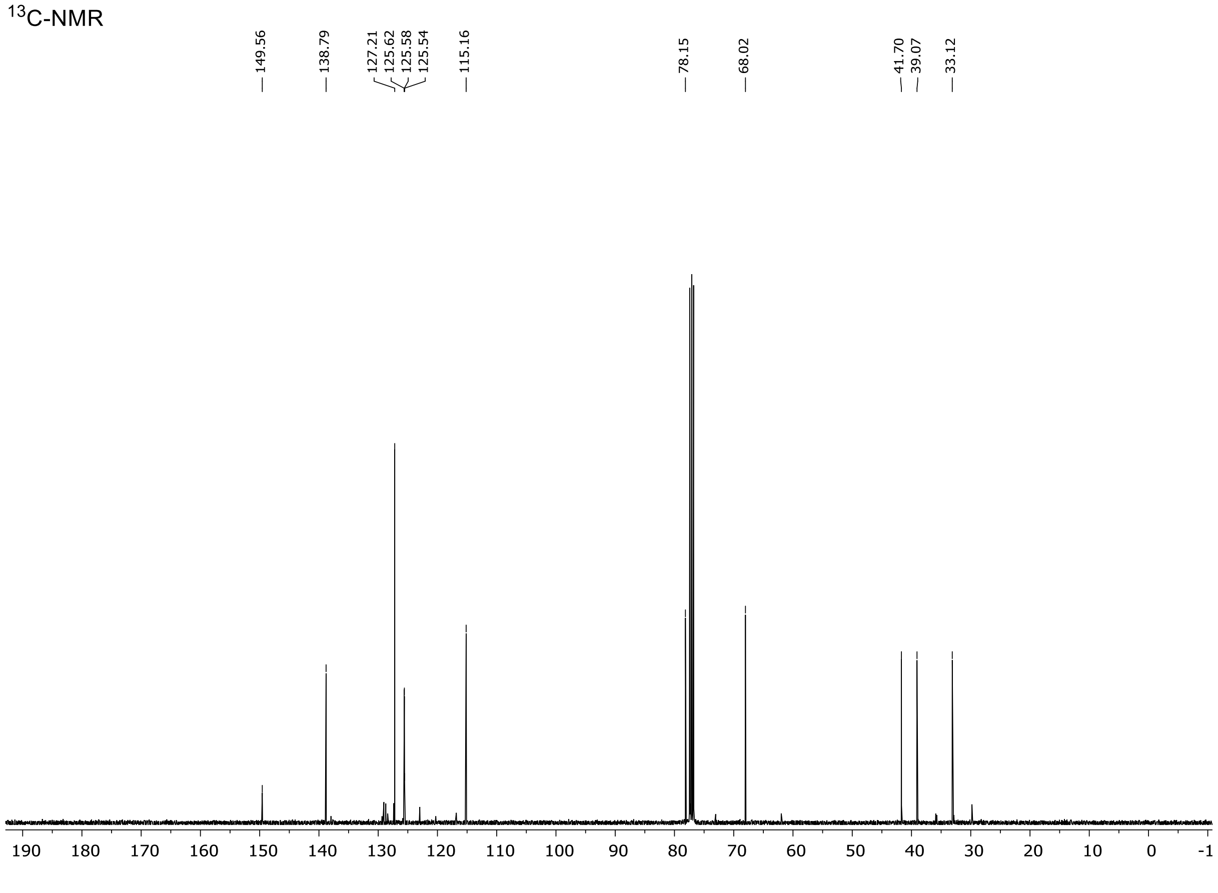

syn-4-(4-bromophenyl)-2-vinyltetrahydro-2H-pyran 19

^{1}H-NMR

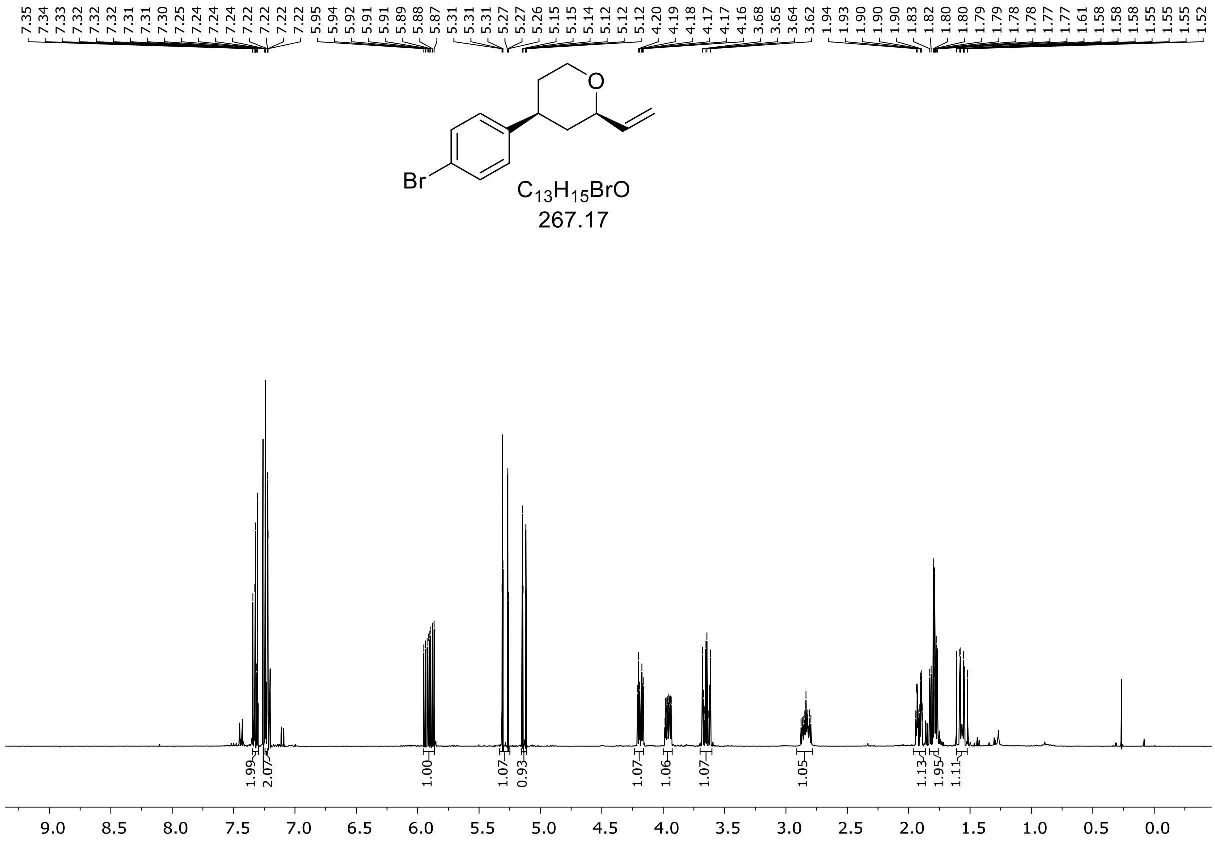

^{13}C-NMR

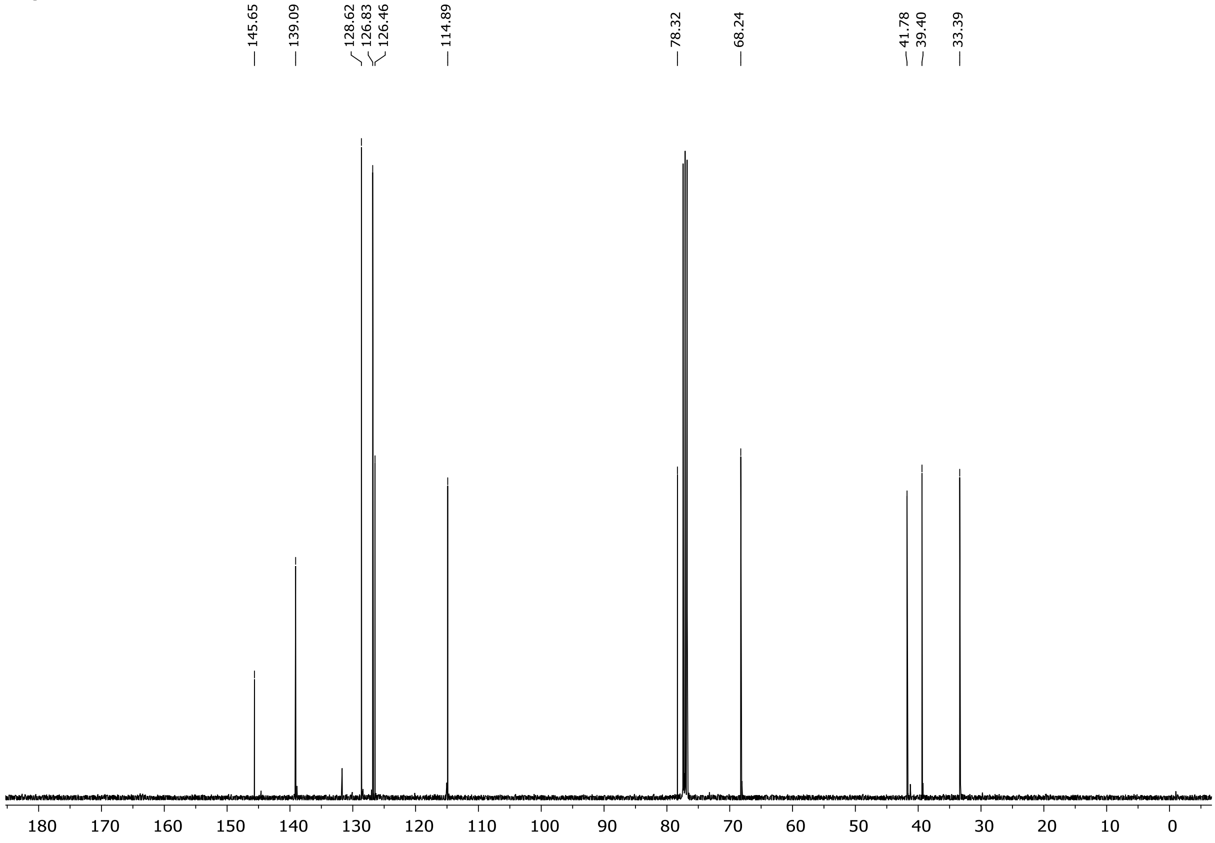

syn-4-(4-methoxyphenyl)-2-vinyltetrahydro-2H-pyran 20

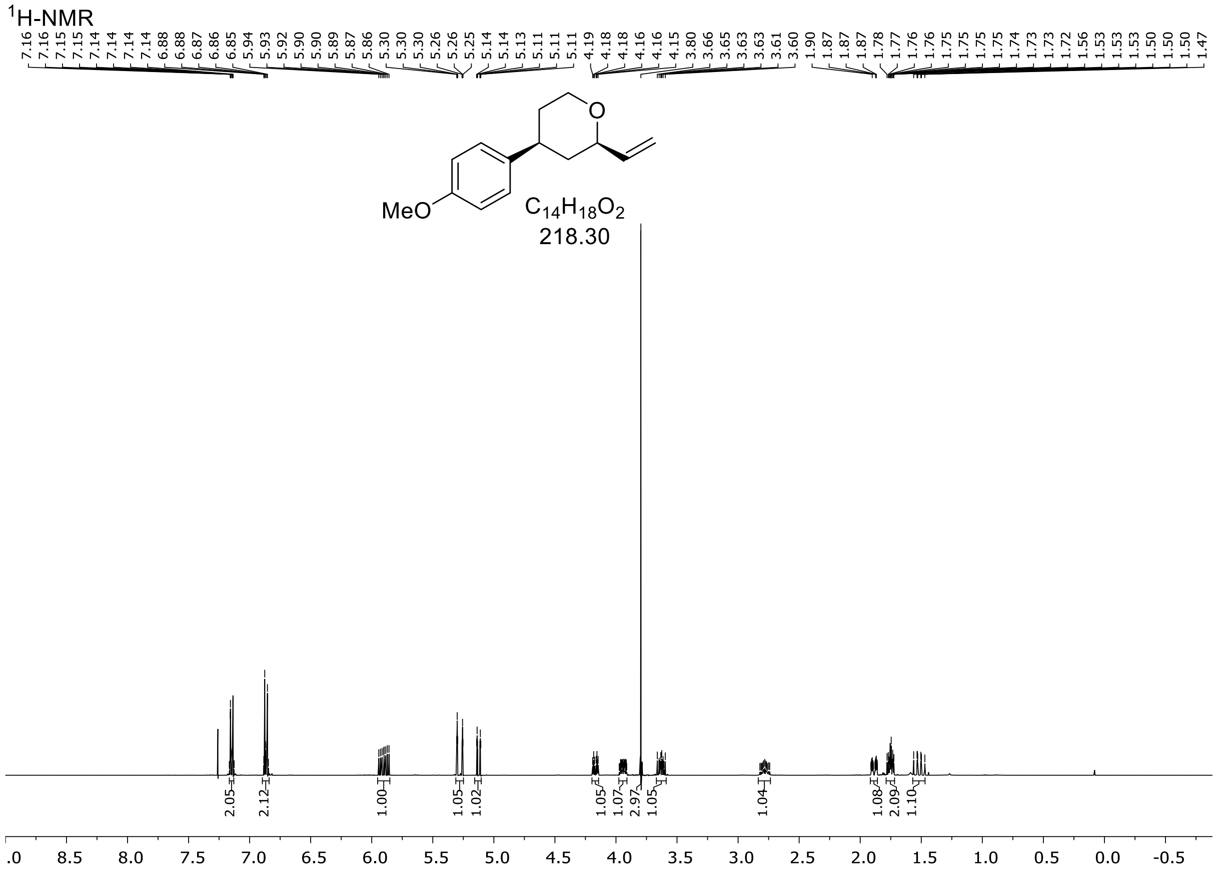

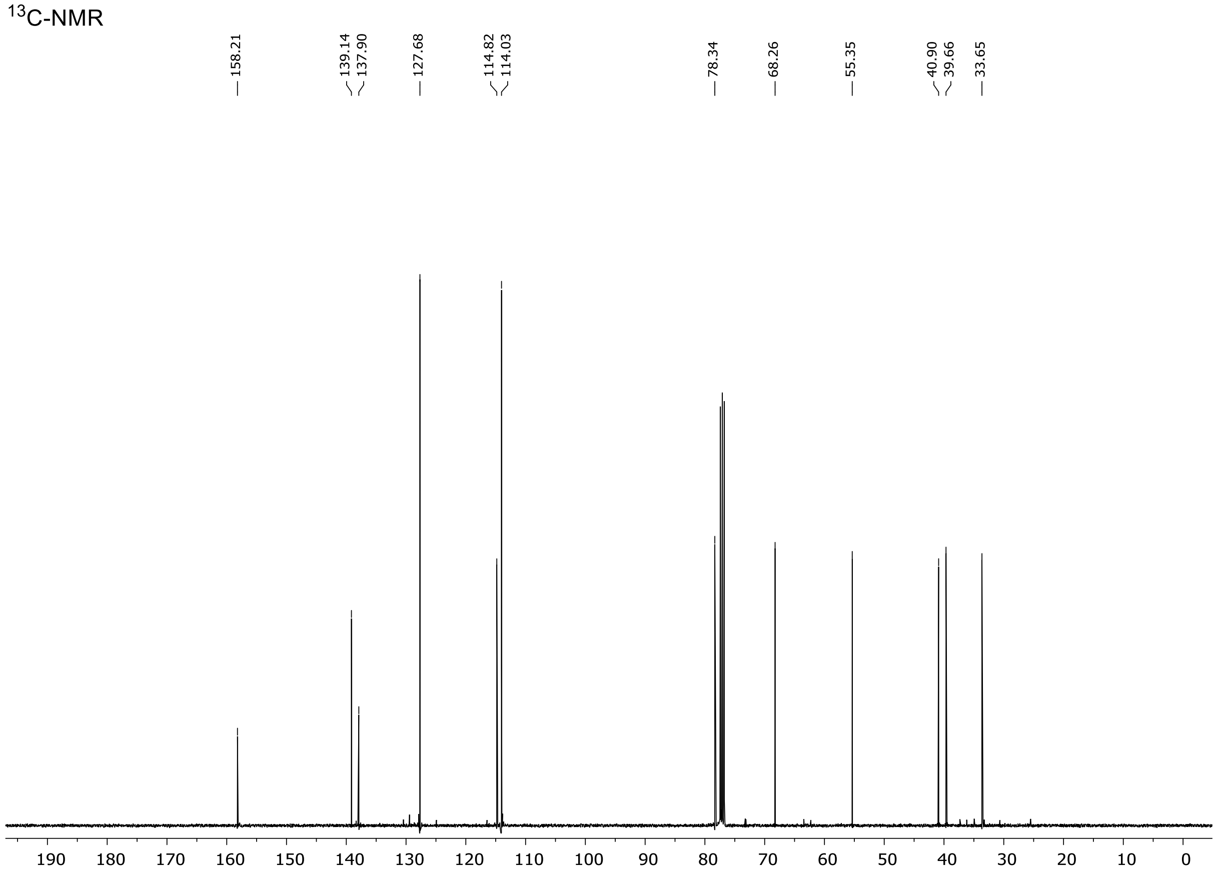

syn-4-(4-(methylthio)phenyl)-2-vinyltetrahydro-2H-pyran 21

^{1}H-NMR

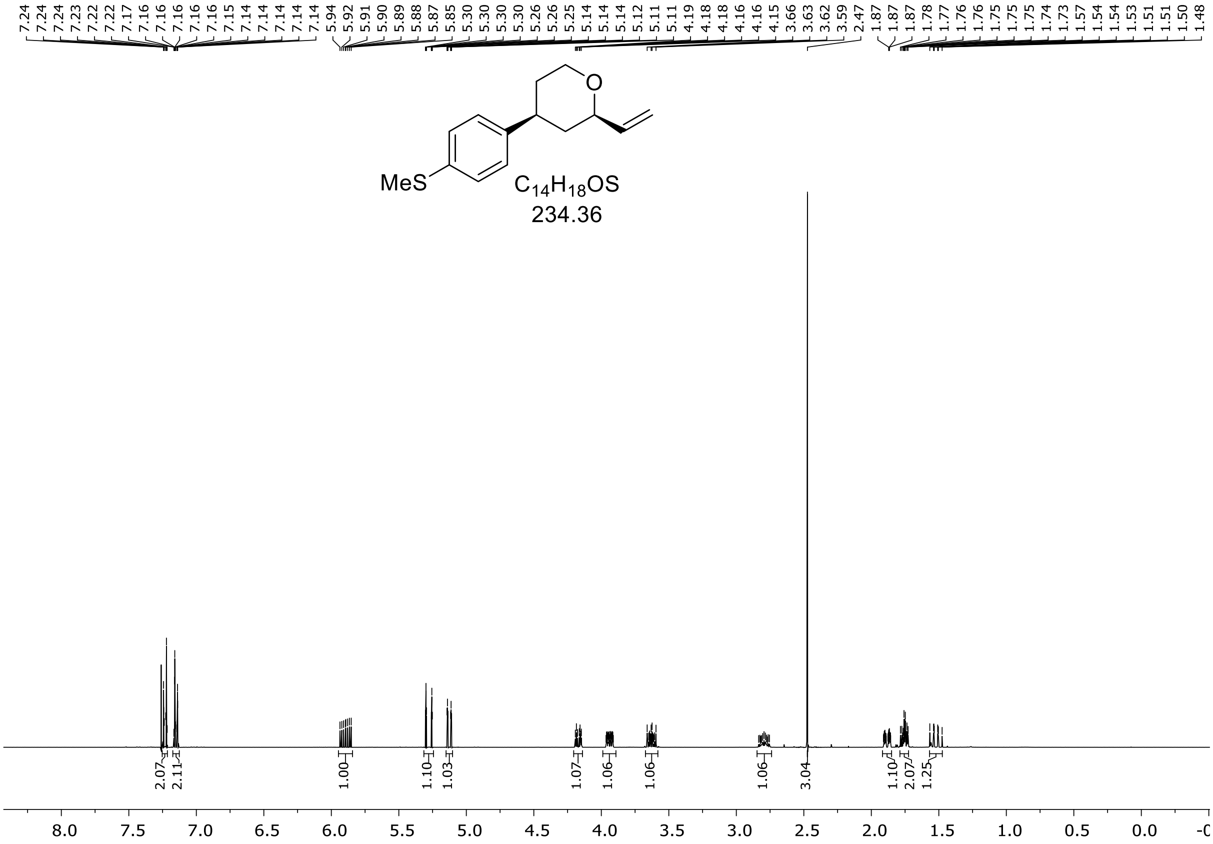

^{13}C-NMR

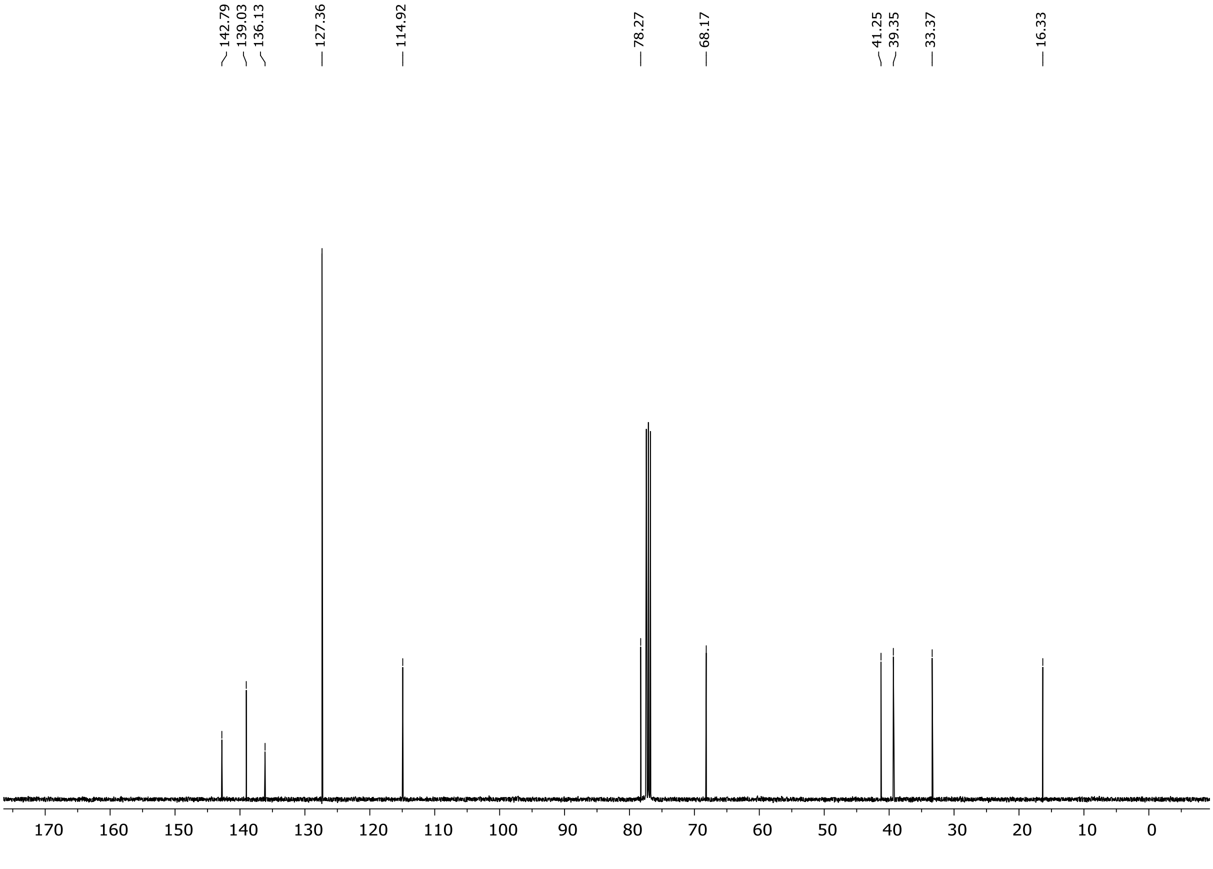

syn-4-(4-methoxyphenyl)-3,3-dimethyl-2-vinyltetrahydro-2H-pyran 22

^{1}H-NMR

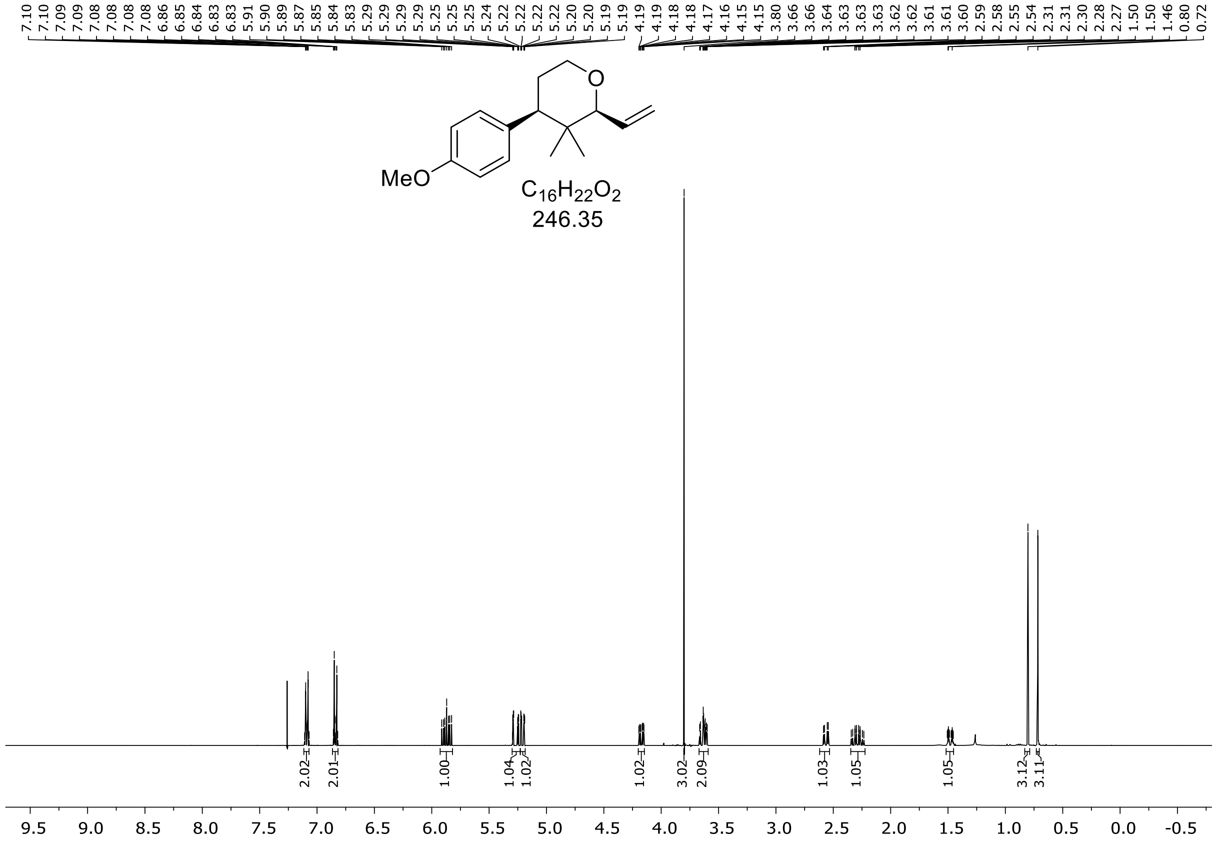

^{13}C-NMR

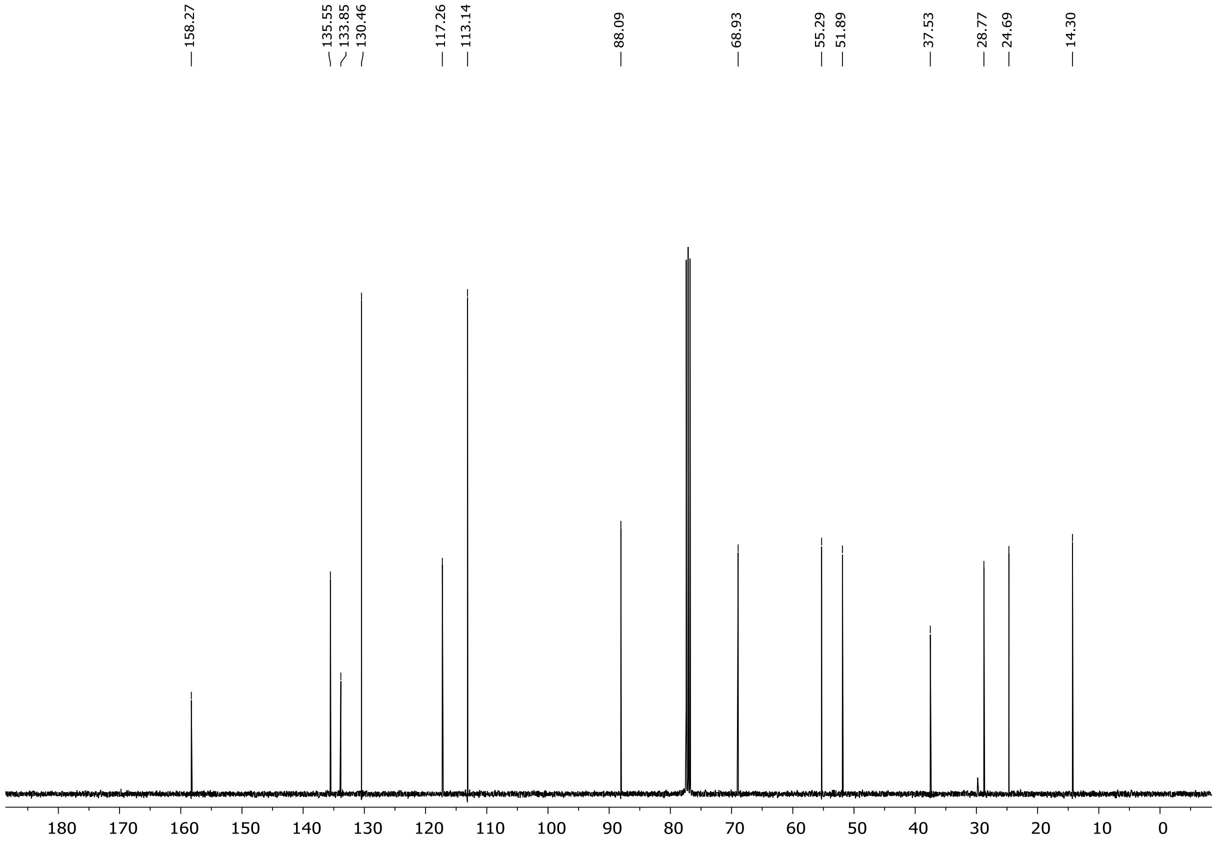

syn-4-dodecyl-2-(E)-oct-1-en-1-yl)tetrahydro-2H-pyran 23

^{1}H-NMR

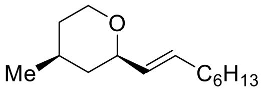

$C_{14}H_{26}O$
210.36

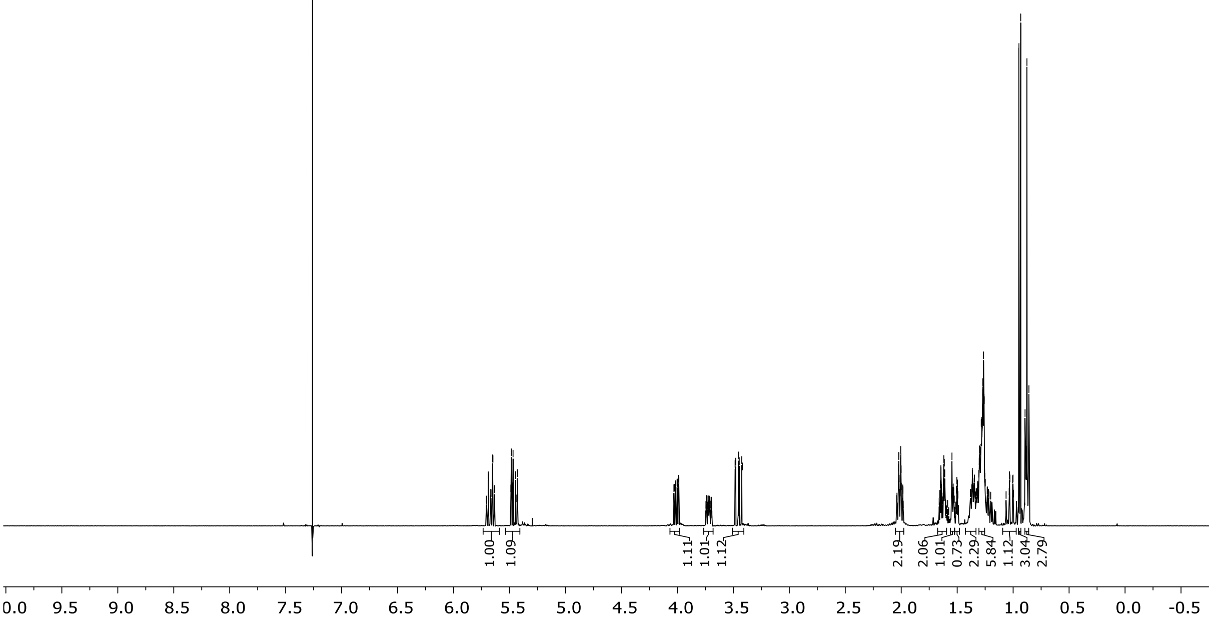

^{13}C-NMR

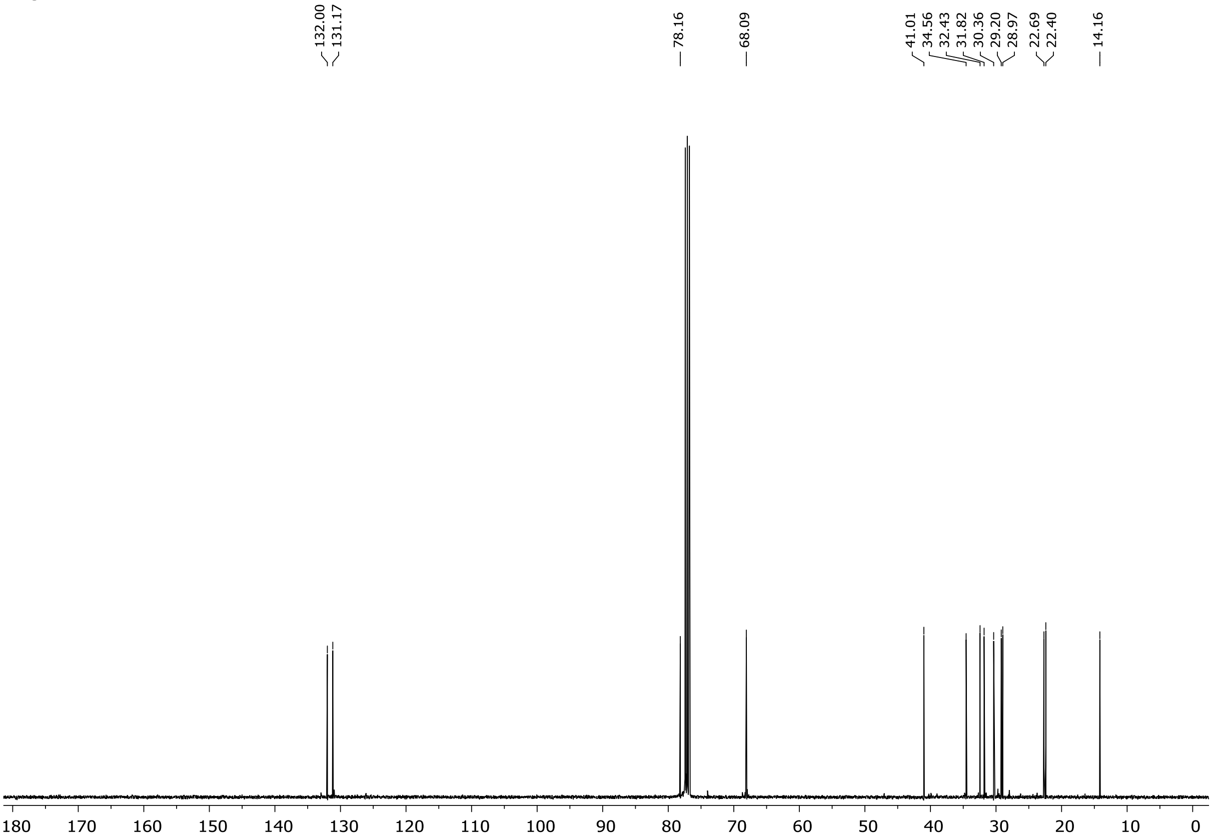

syn-4-neopentyl-2-(E)-oct-1-en-1-yl)tetrahydro-2H-pyran 25

^{1}H-NMR

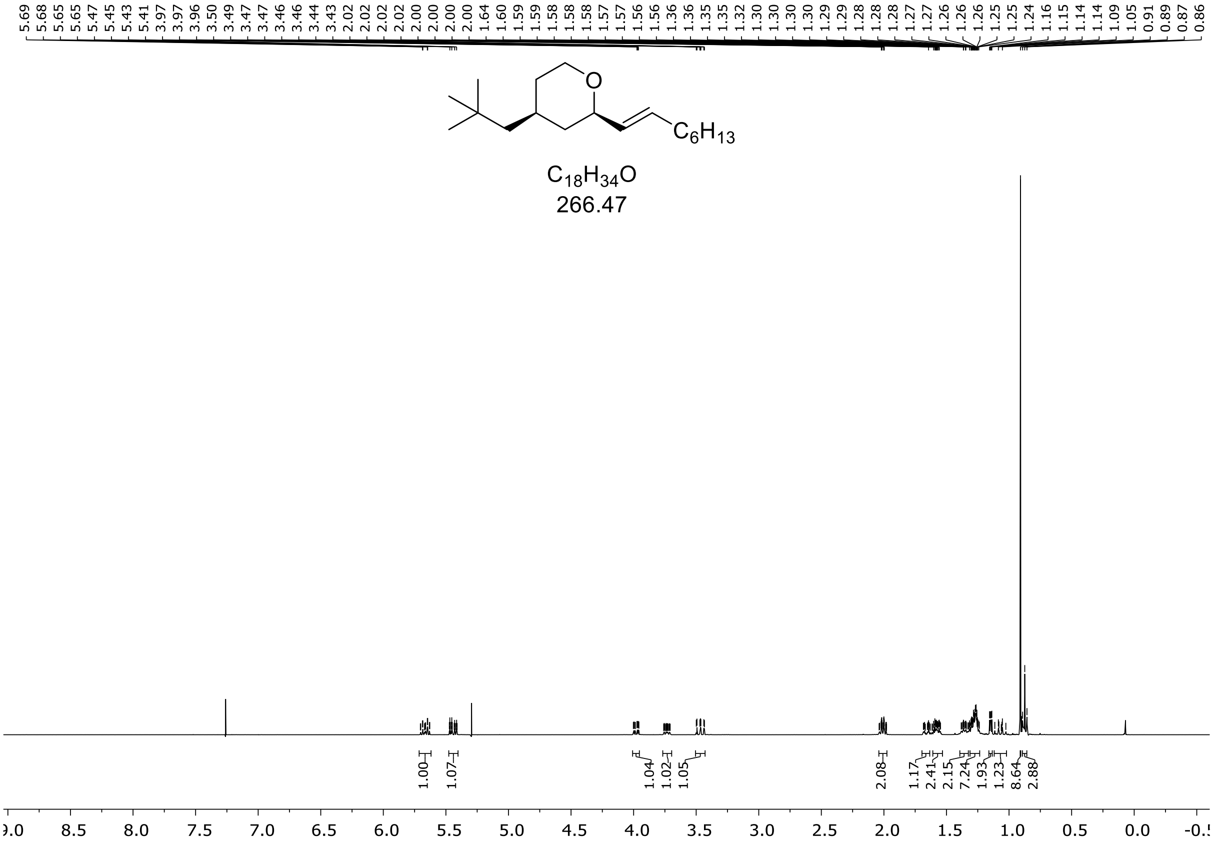

^{13}C-NMR

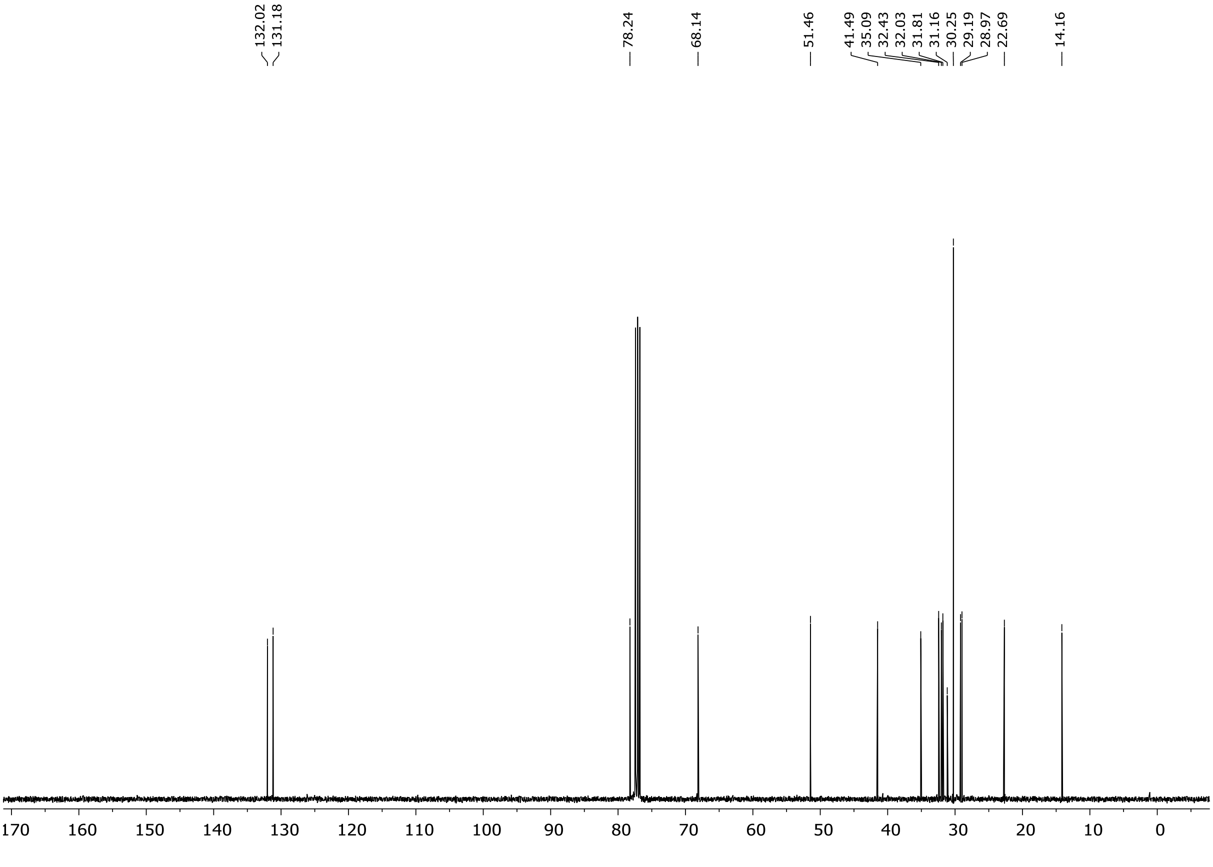

syn-4 -cyclopropyl-2-(E)-oct-1-en-1-yl)tetrahydro-2H-pyran 26

^{1}H-NMR

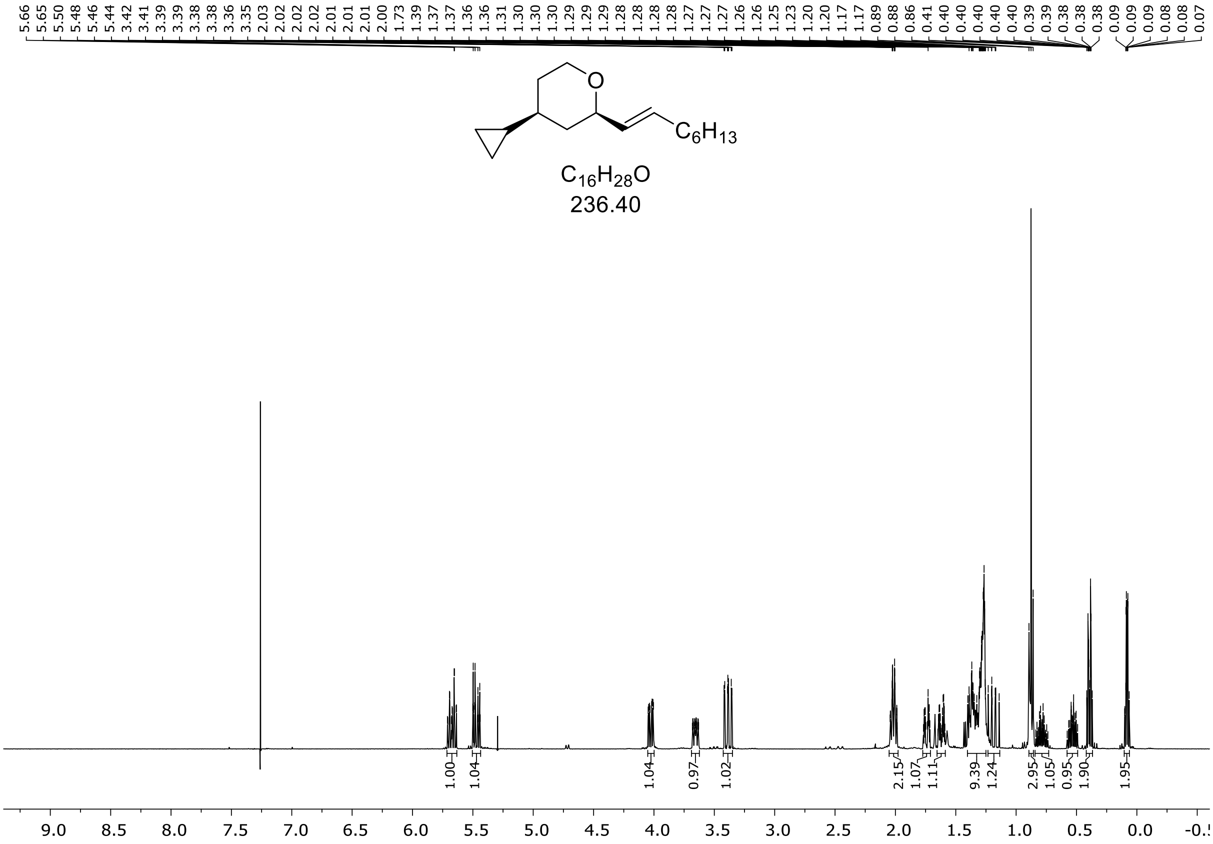

^{13}C-NMR

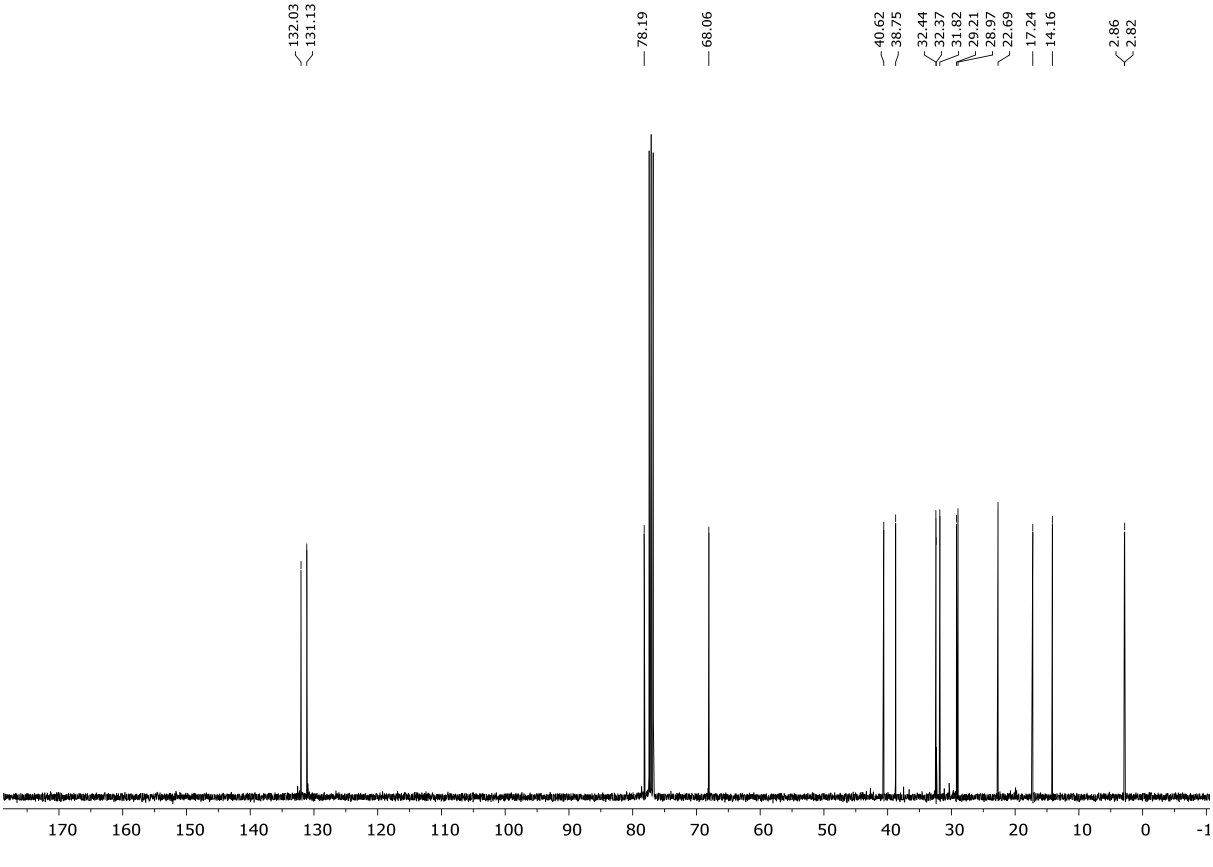

syn-4-cyclohexyl-2-(E)-oct-1-en-1-yl)tetrahydro-2H-pyran 27

^{1}H-NMR

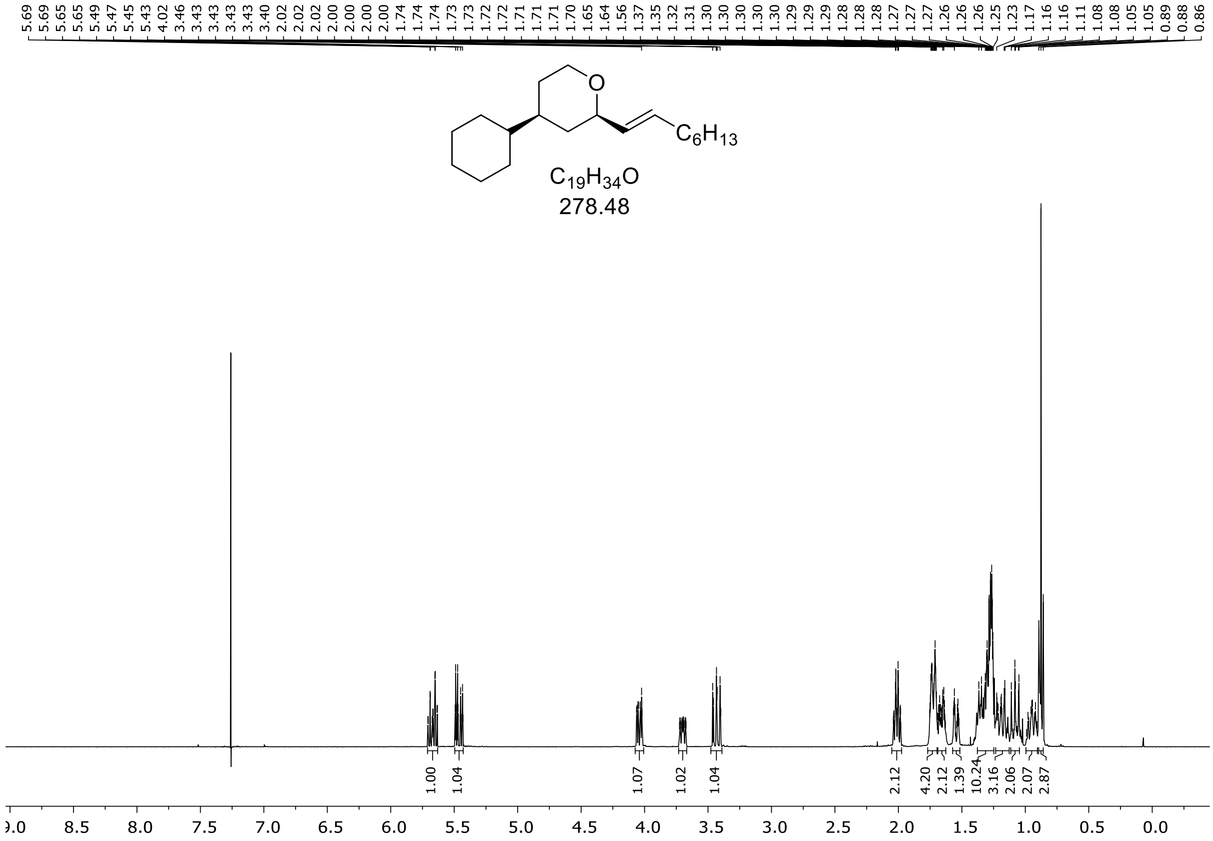

^{13}C-NMR

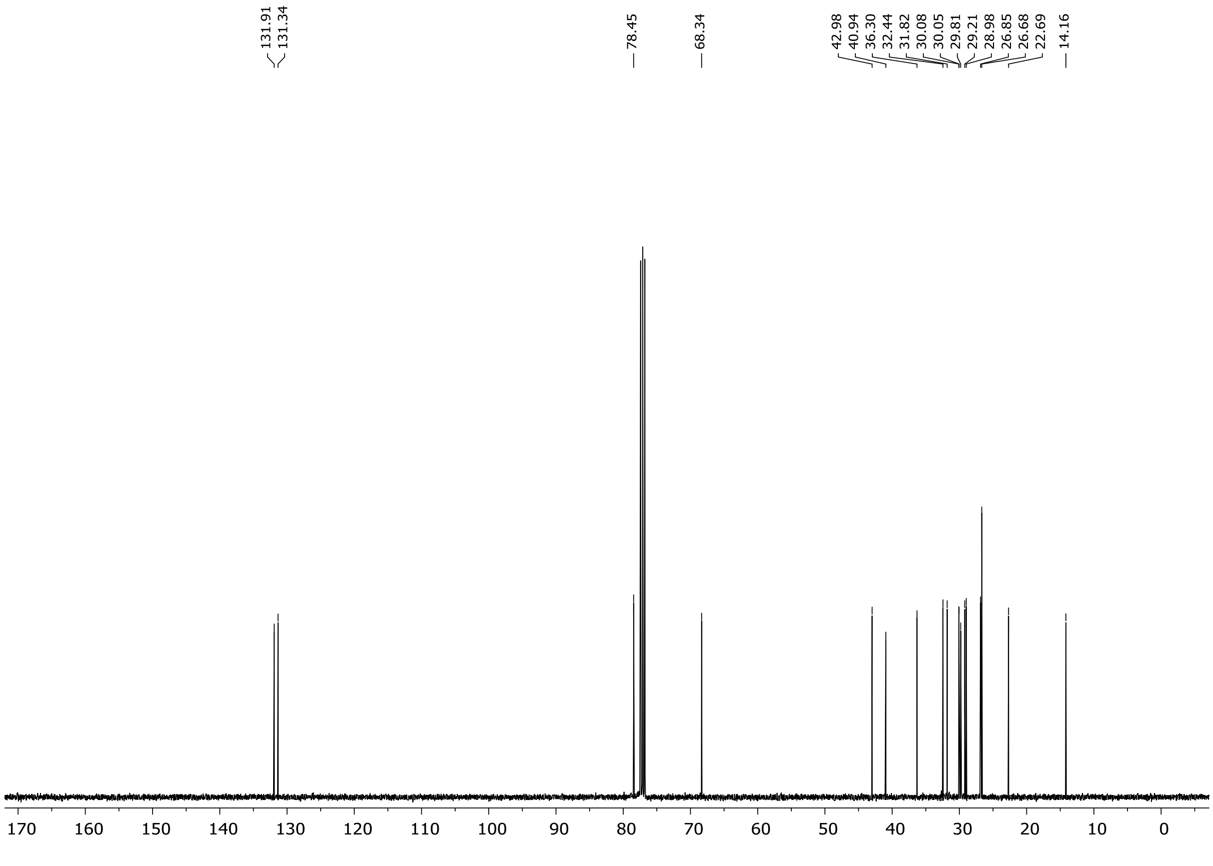

syn-2-(E)-oct-1-en-1-yl)-4-(3-phenylpropyl)tetrahydro-2H-pyran 28

^{1}H-NMR

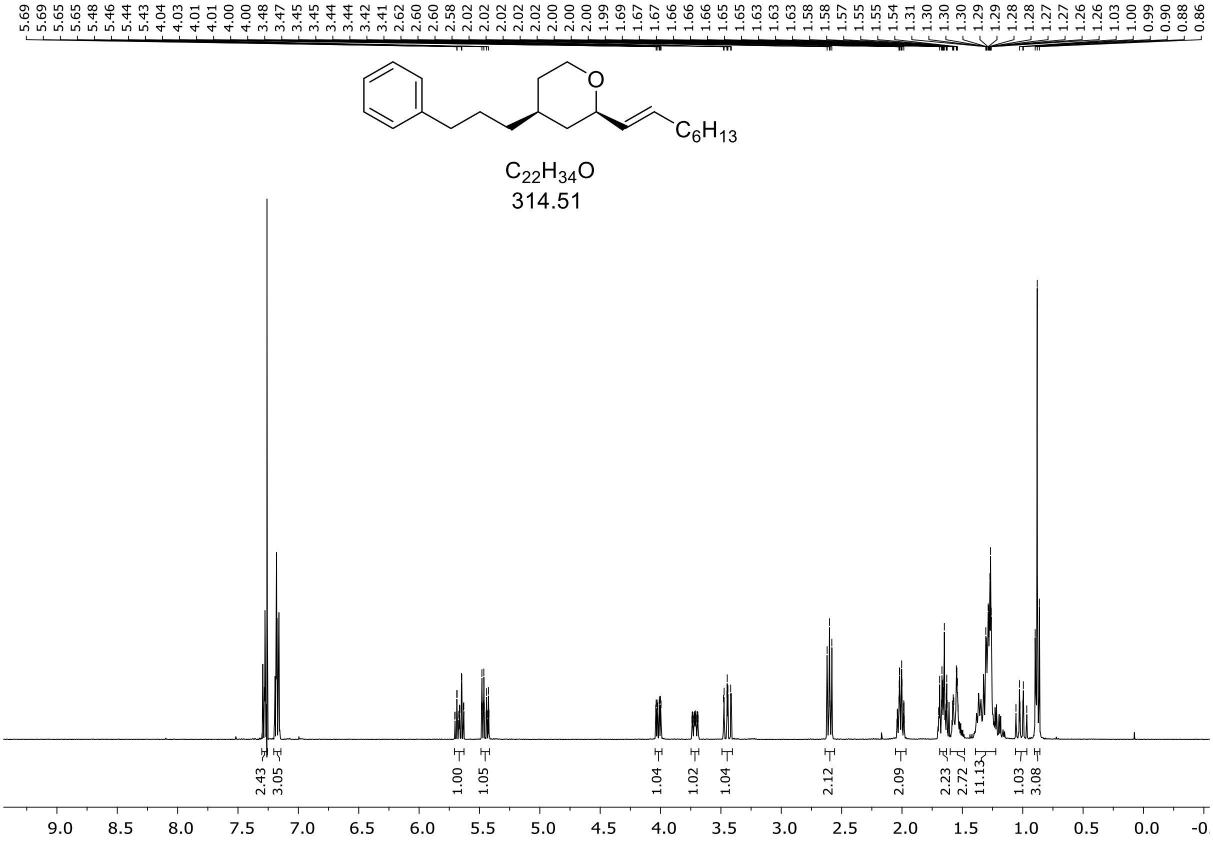

^{13}C-NMR

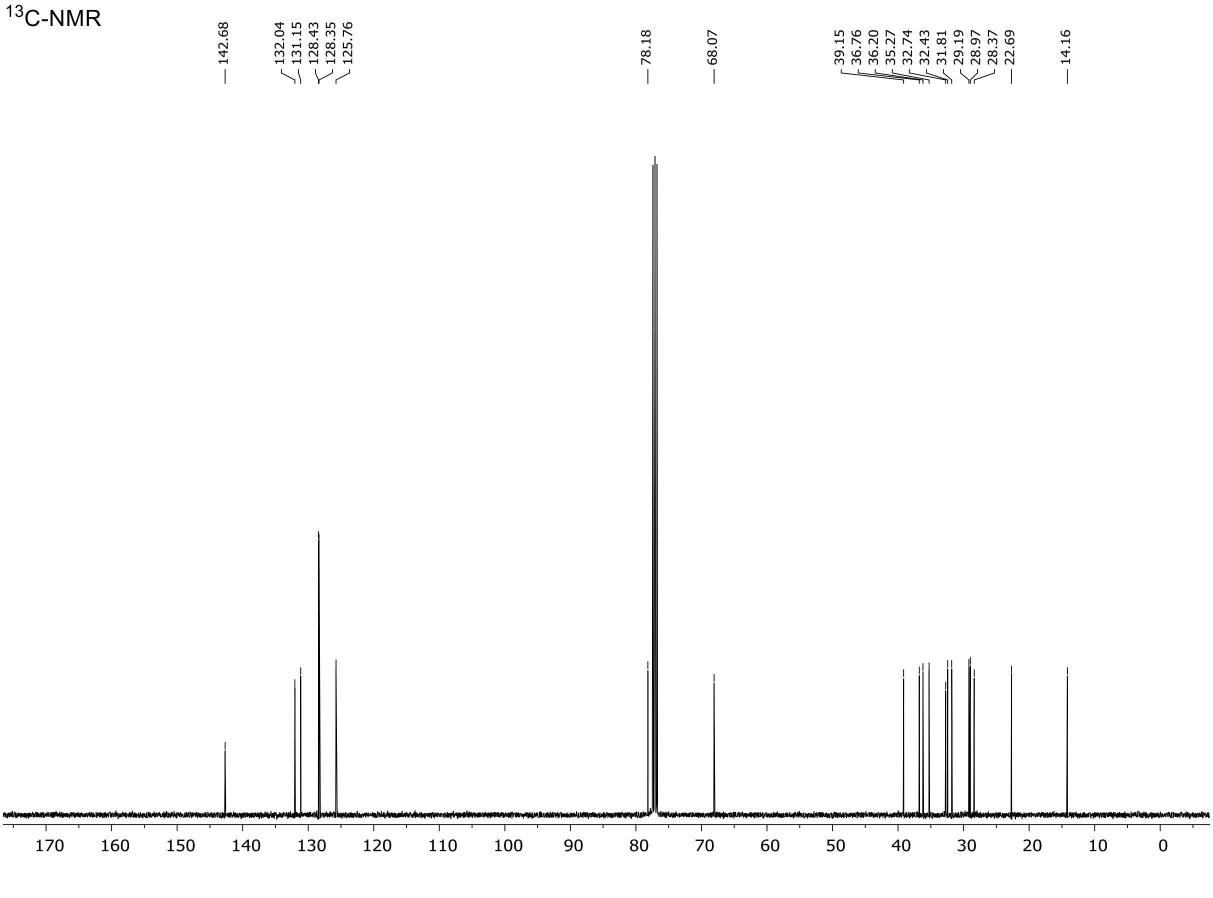

syn-2-(E)-oct-1-en-1-yl)-4-phenyltetrahydro-2H-pyran 29

^{1}H-NMR

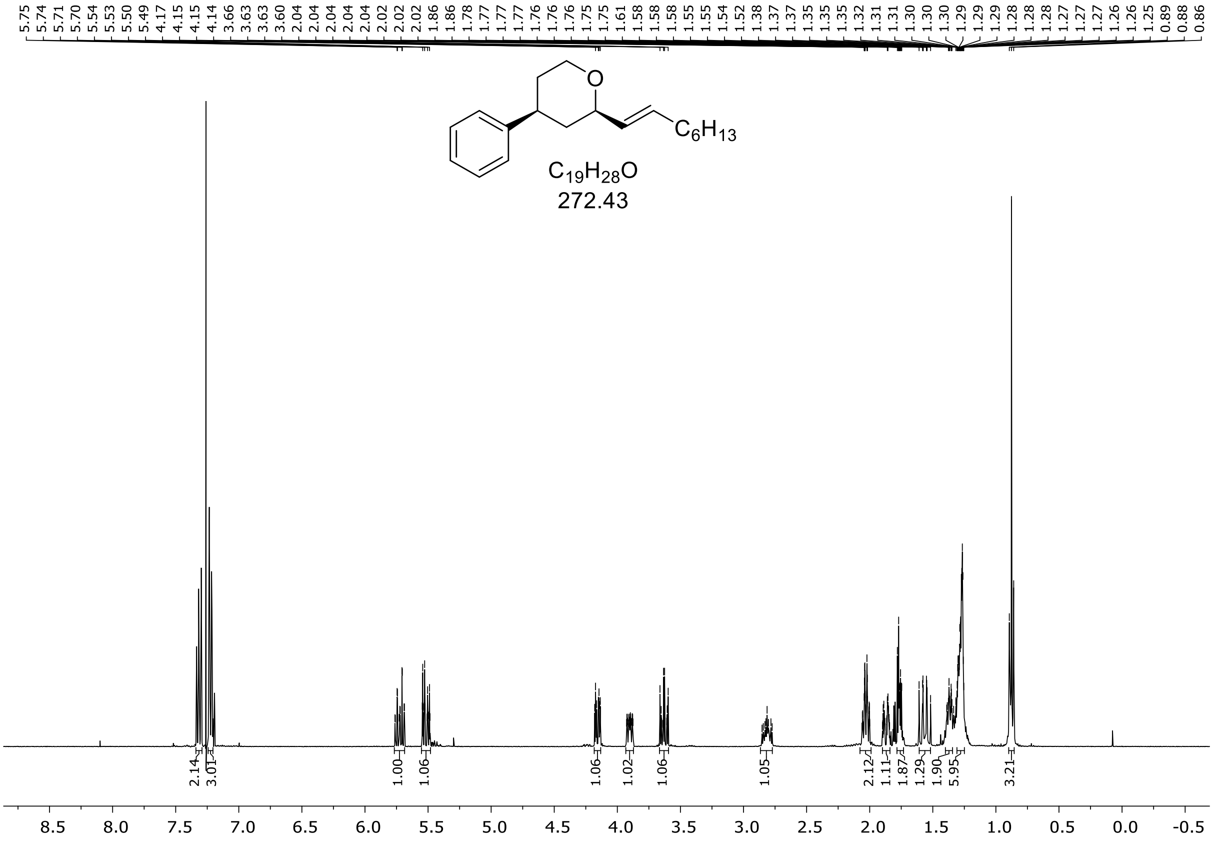

^{13}C-NMR

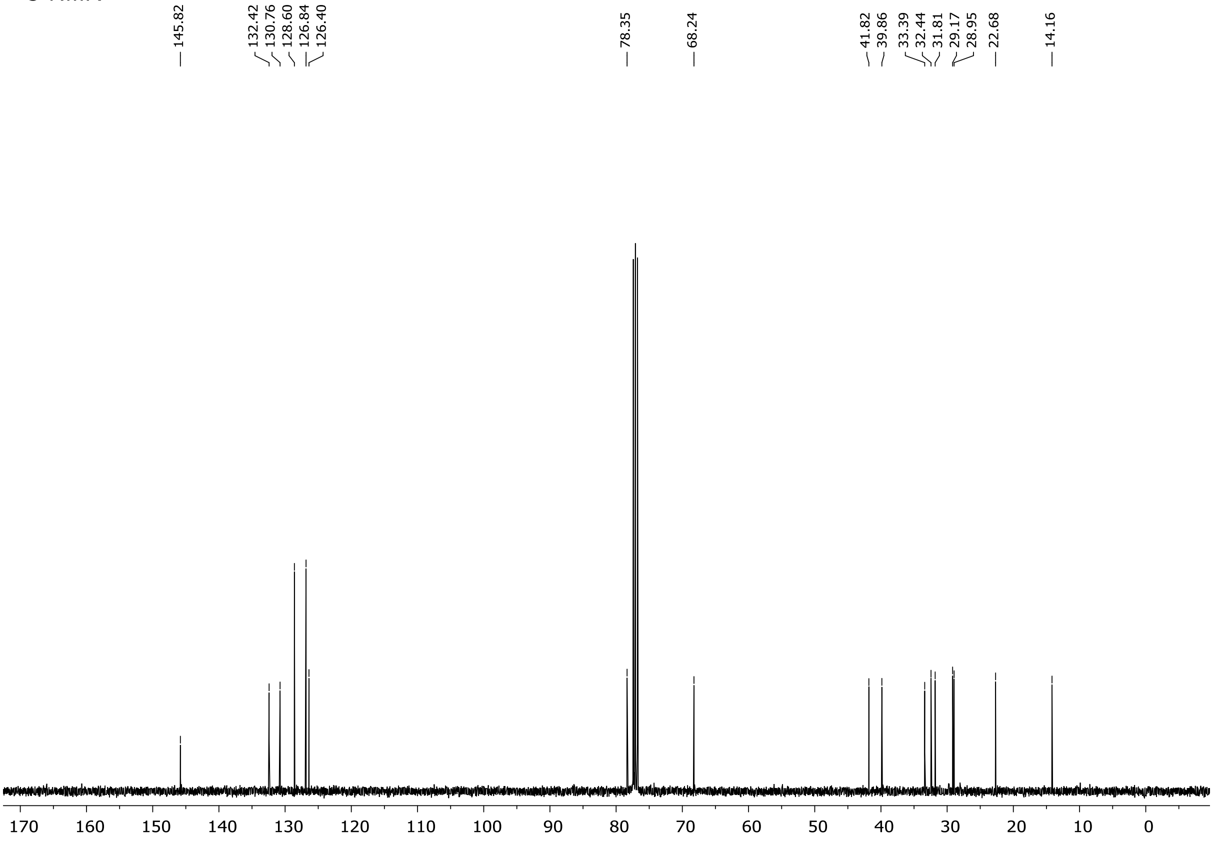

syn-4-(naphthalen-2-yl)-2-(E)-oct-1-en-1-yl)tetrahydro-2H-pyran 30

^{1}H-NMR

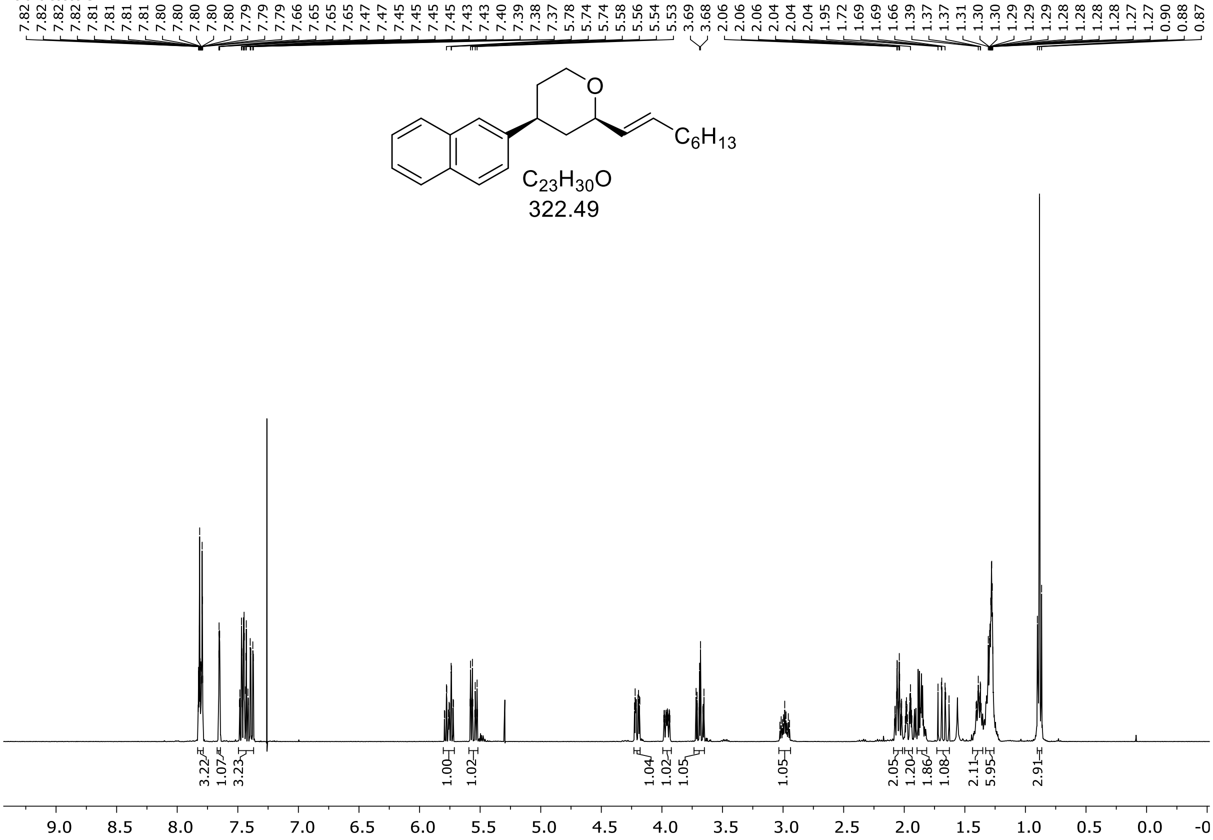

^{13}C-NMR

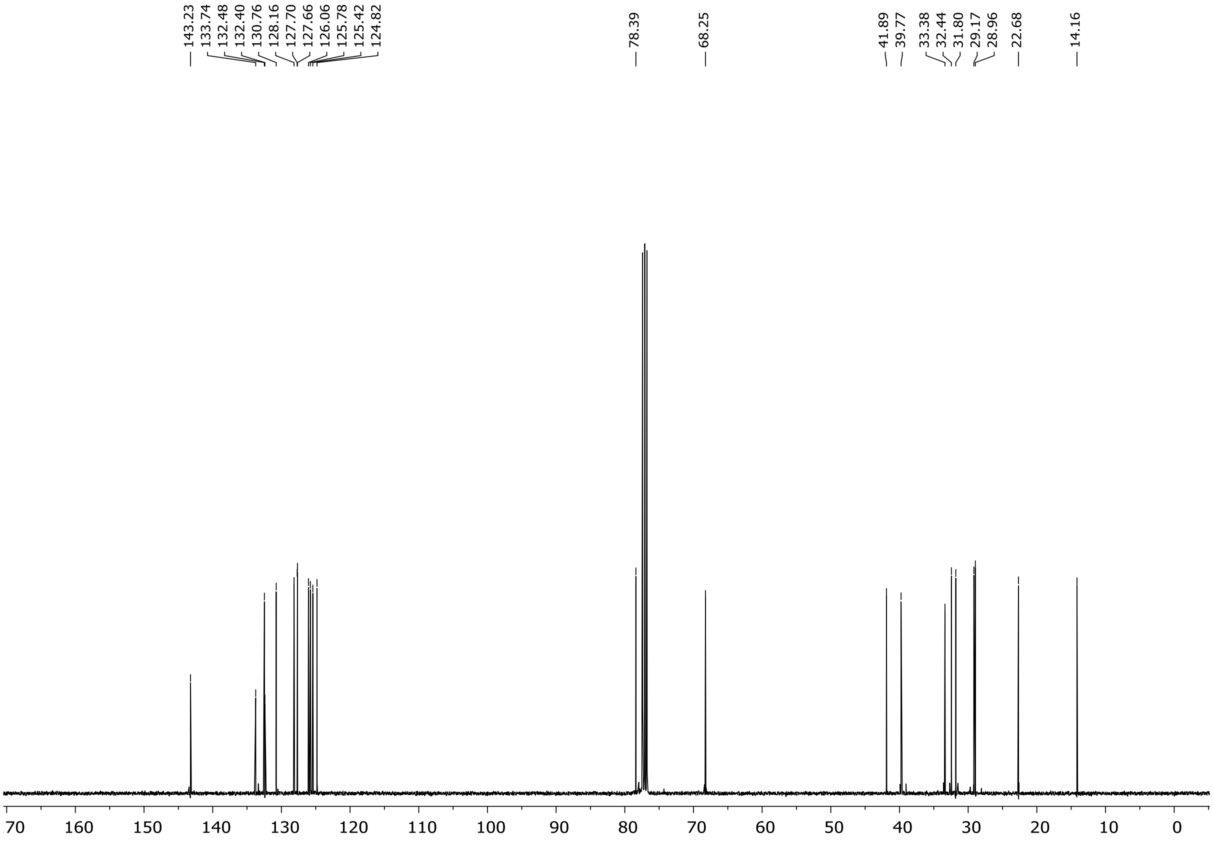

syn-4-([1,1'-biphenyl]-4-yl)-2-((E)-oct-1-en-1-yl)tetrahydro-2H-pyran 31

^{1}H-NMR

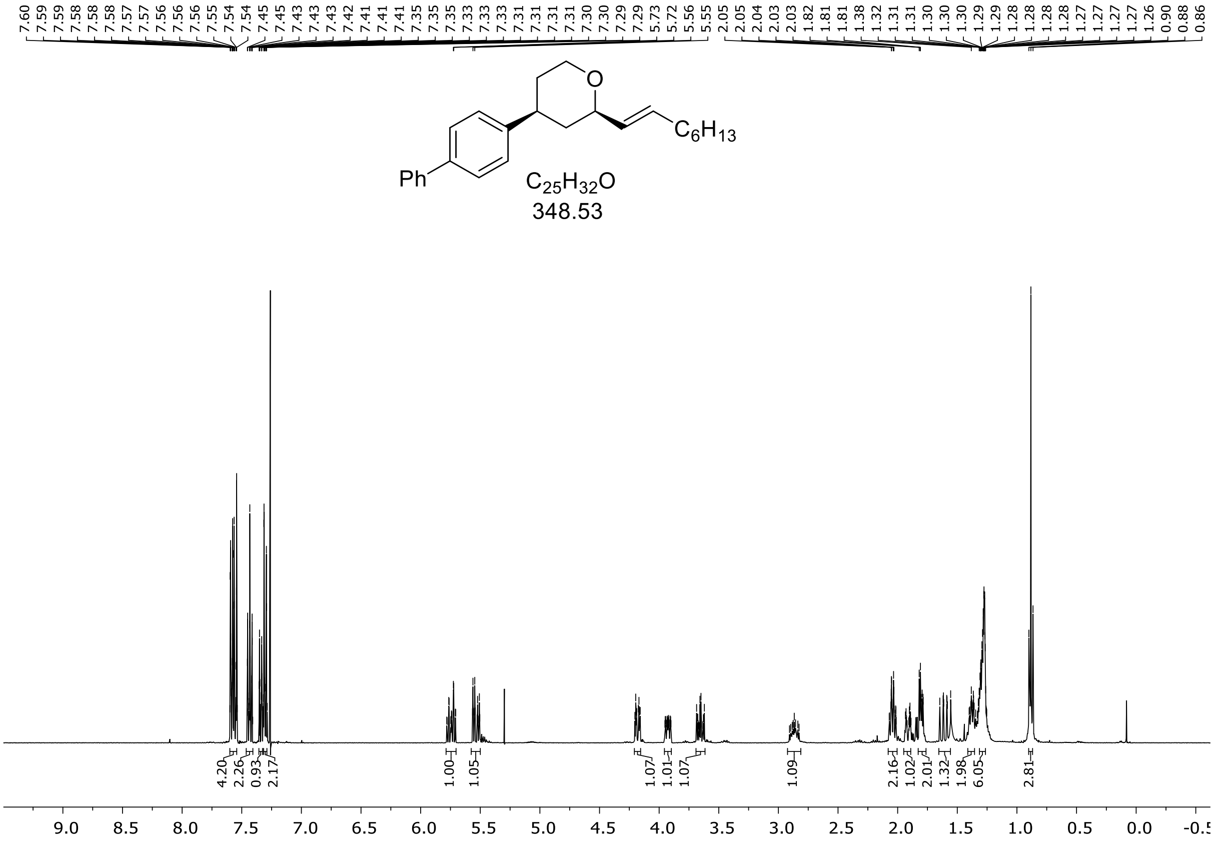

^{13}C-NMR

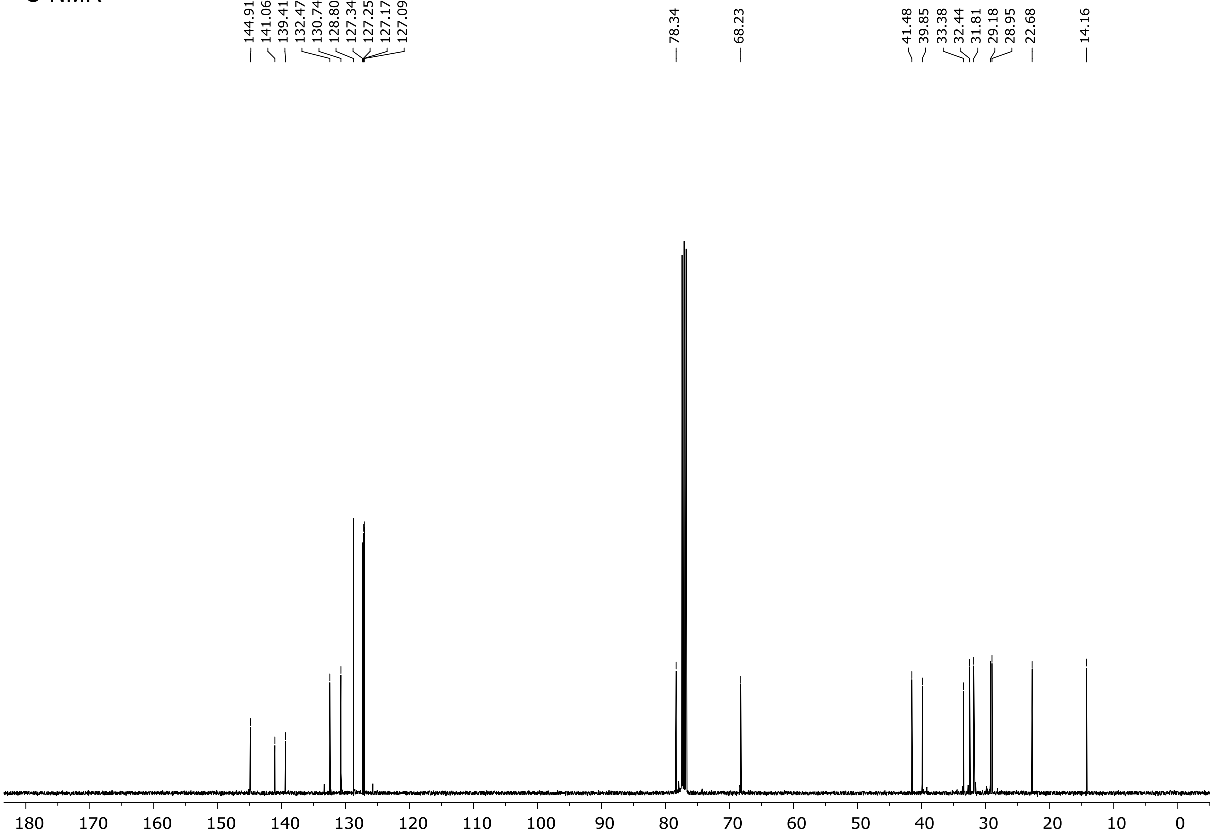

syn-2-((E)-oct-1-en-1-yl)-4-(p-tolyl)tetrahydro-2H-pyran 32

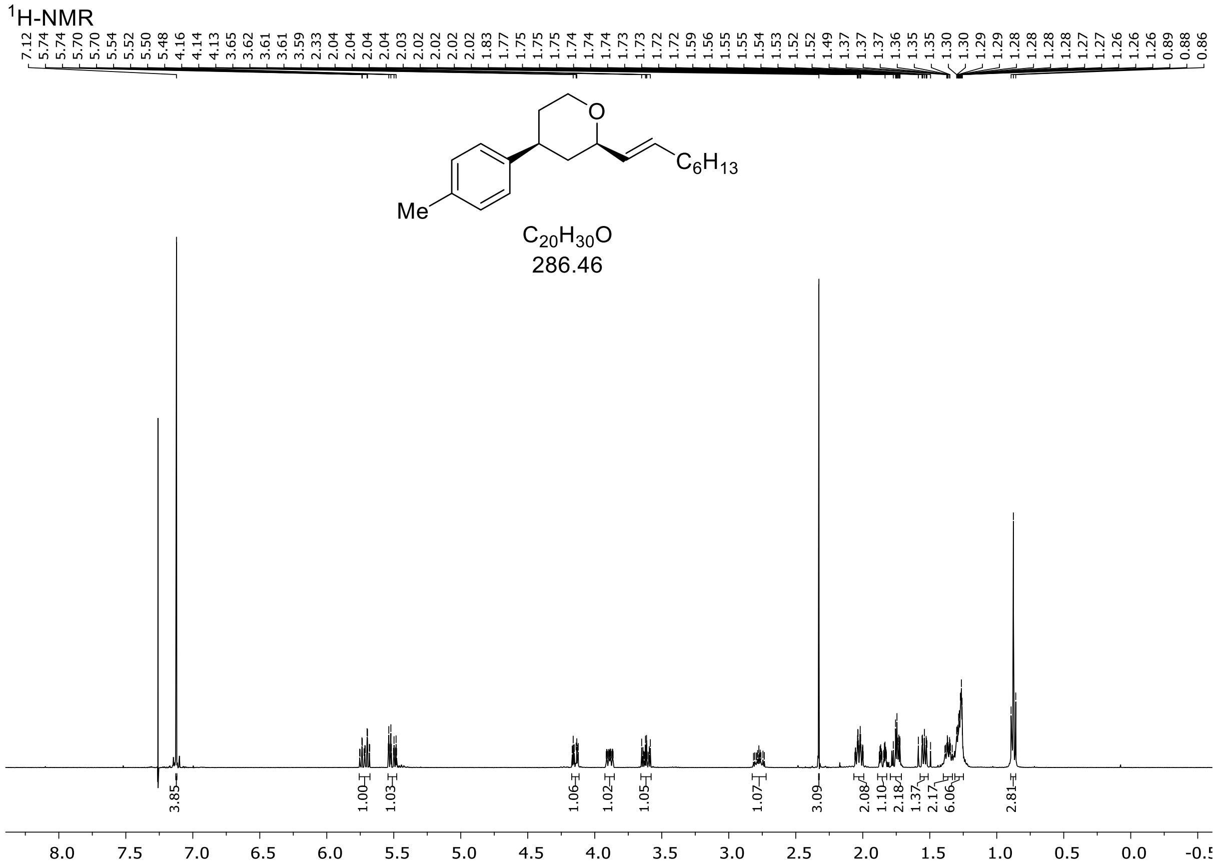

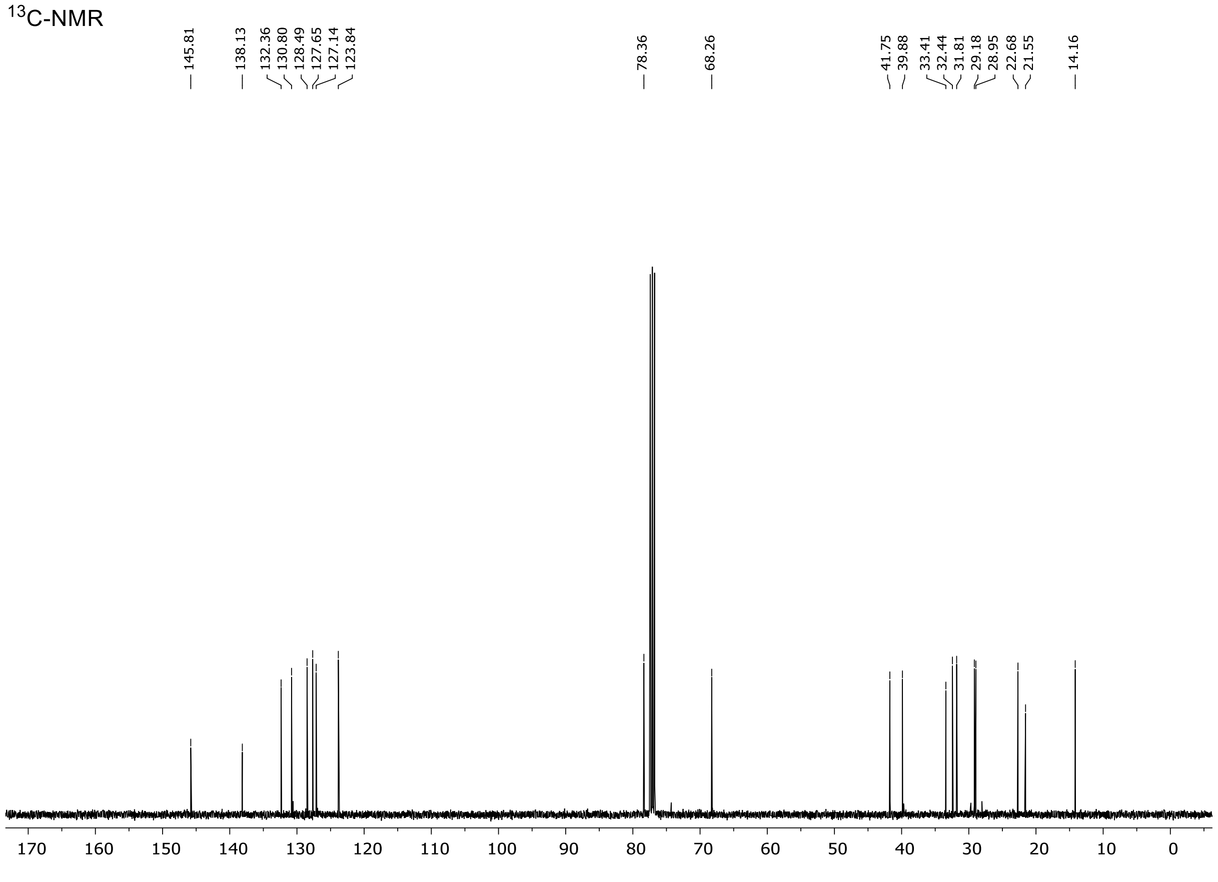

syn-2-((E)-oct-1-en-1-yl)-4-(m-tolyl)tetrahydro-2H-pyran 33

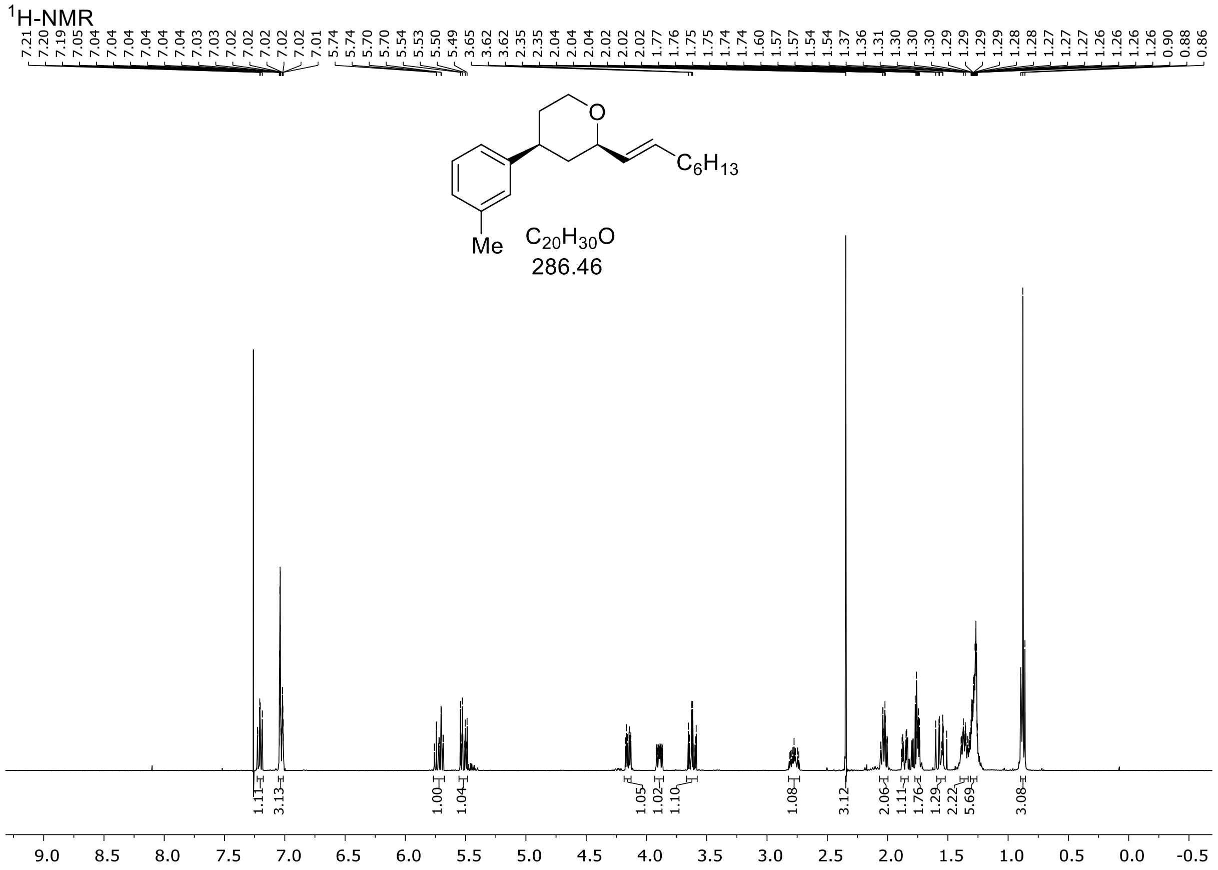

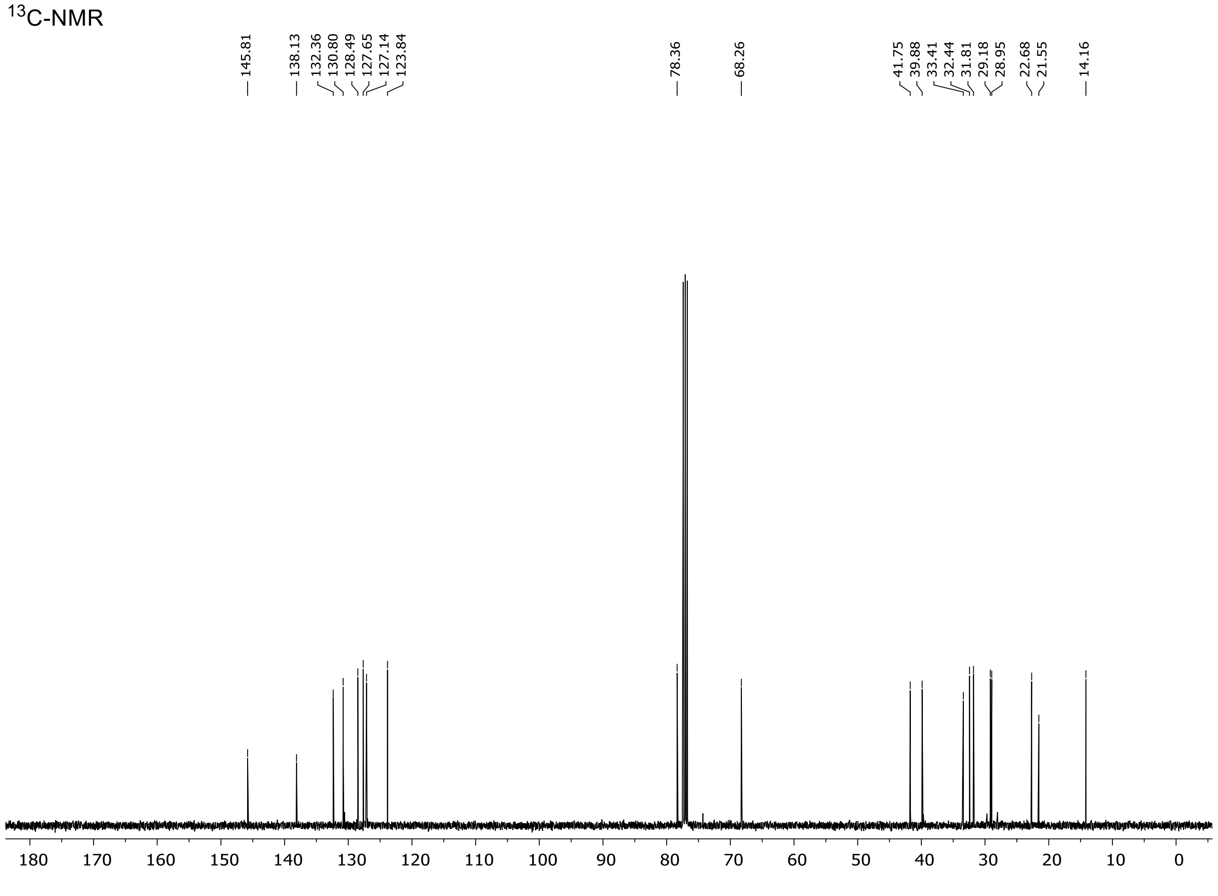

syn-2-((E)-oct-1-en-1-yl)-4-(o-tolyl)tetrahydro-2H-pyran 34

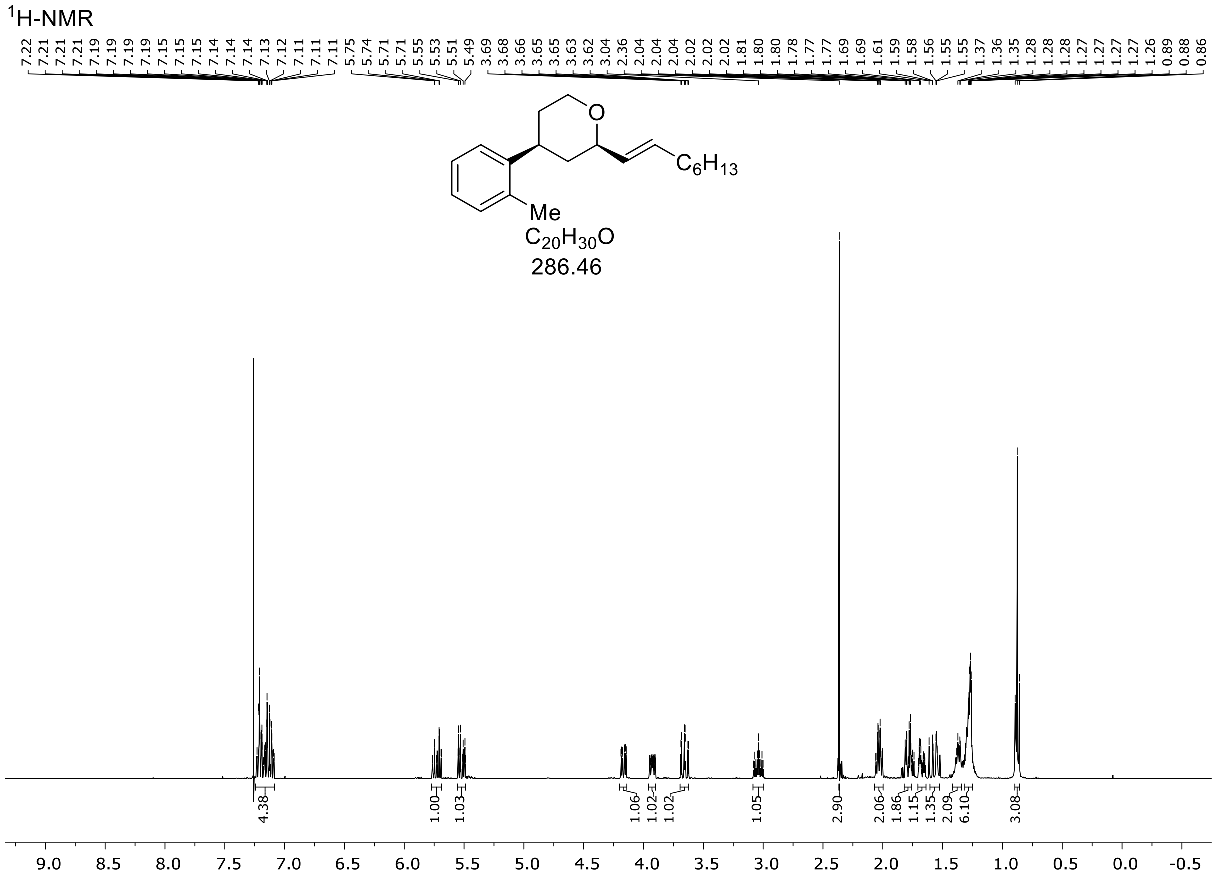

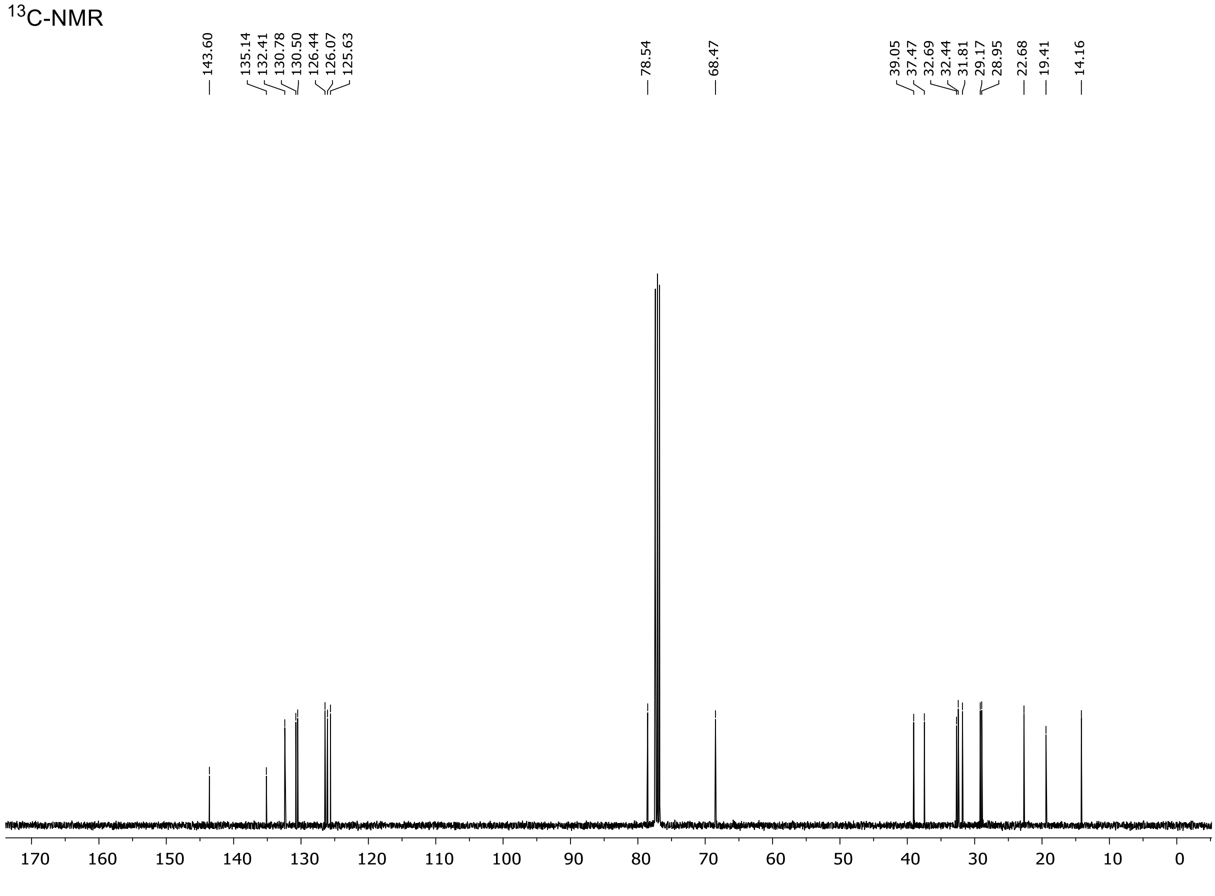

syn-4-mesityl-2-((E)-oct-1-en-1-yl)tetrahydro-2H-pyran 35

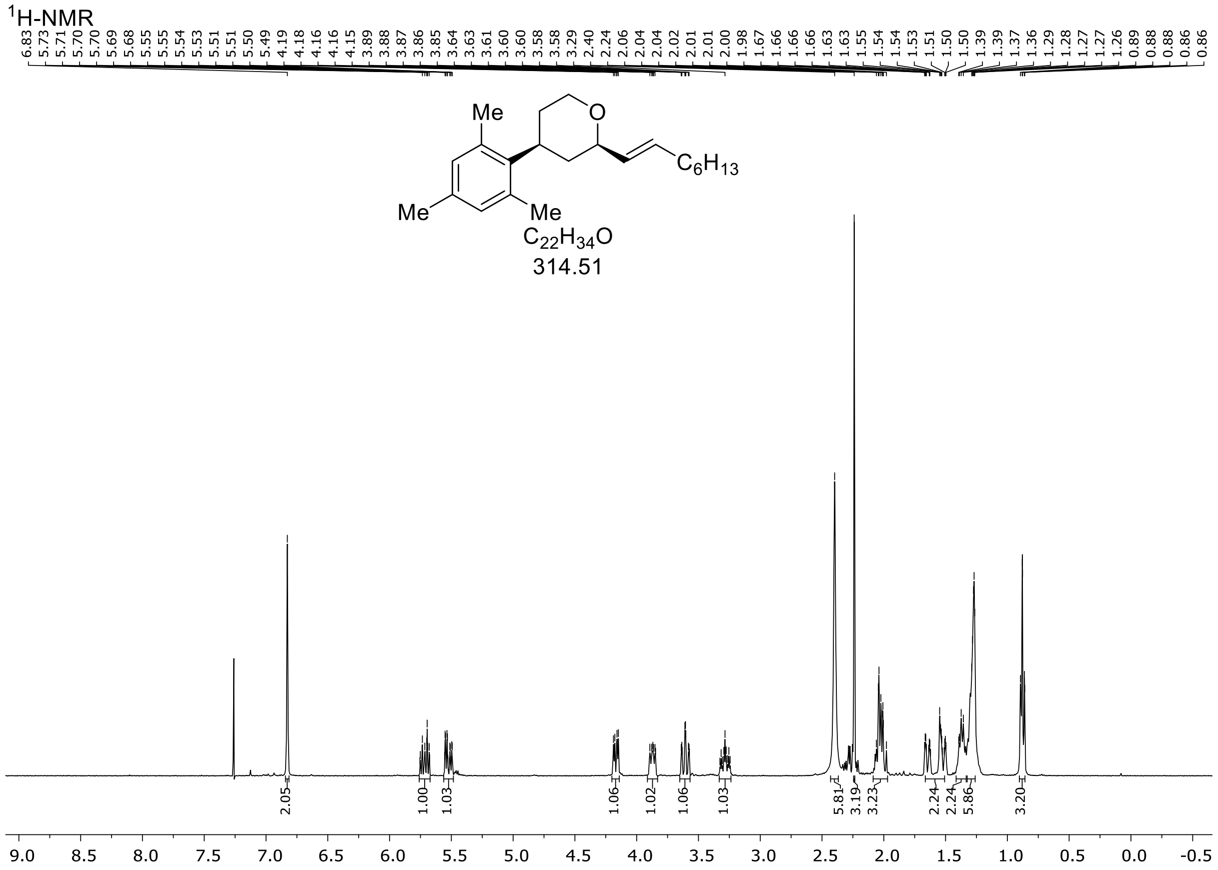

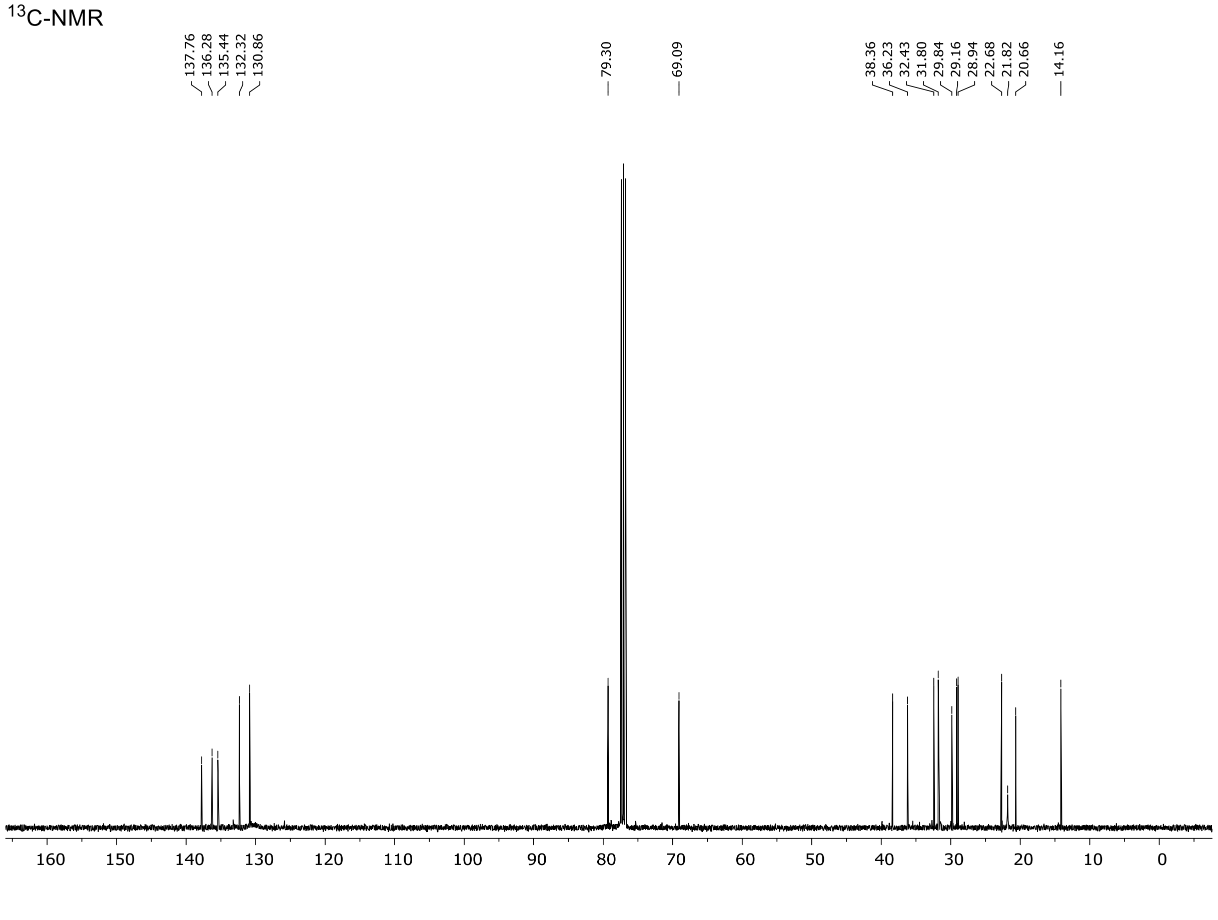

syn-2-((E)-oct-1-en-1-yl)-4-(4-vinylphenyl)tetrahydro-2H-pyran 36

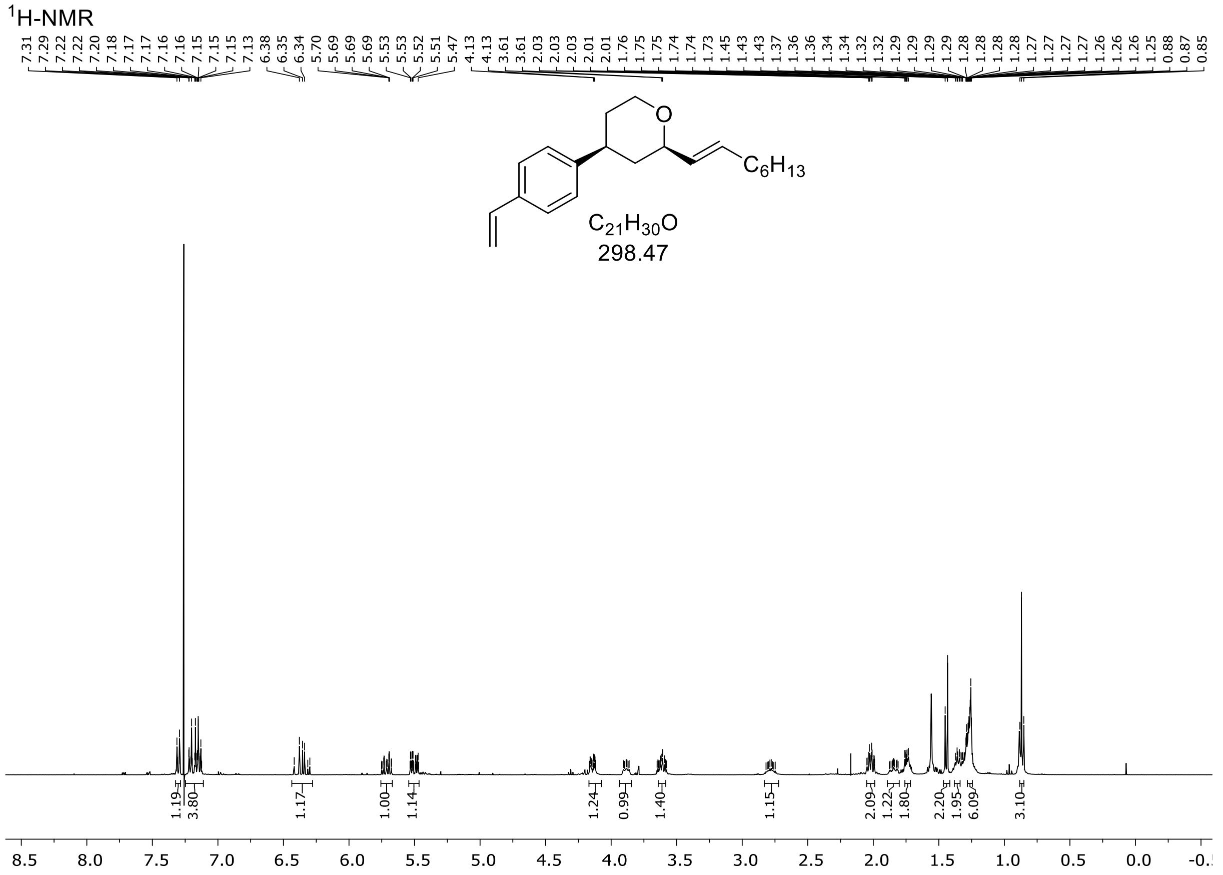

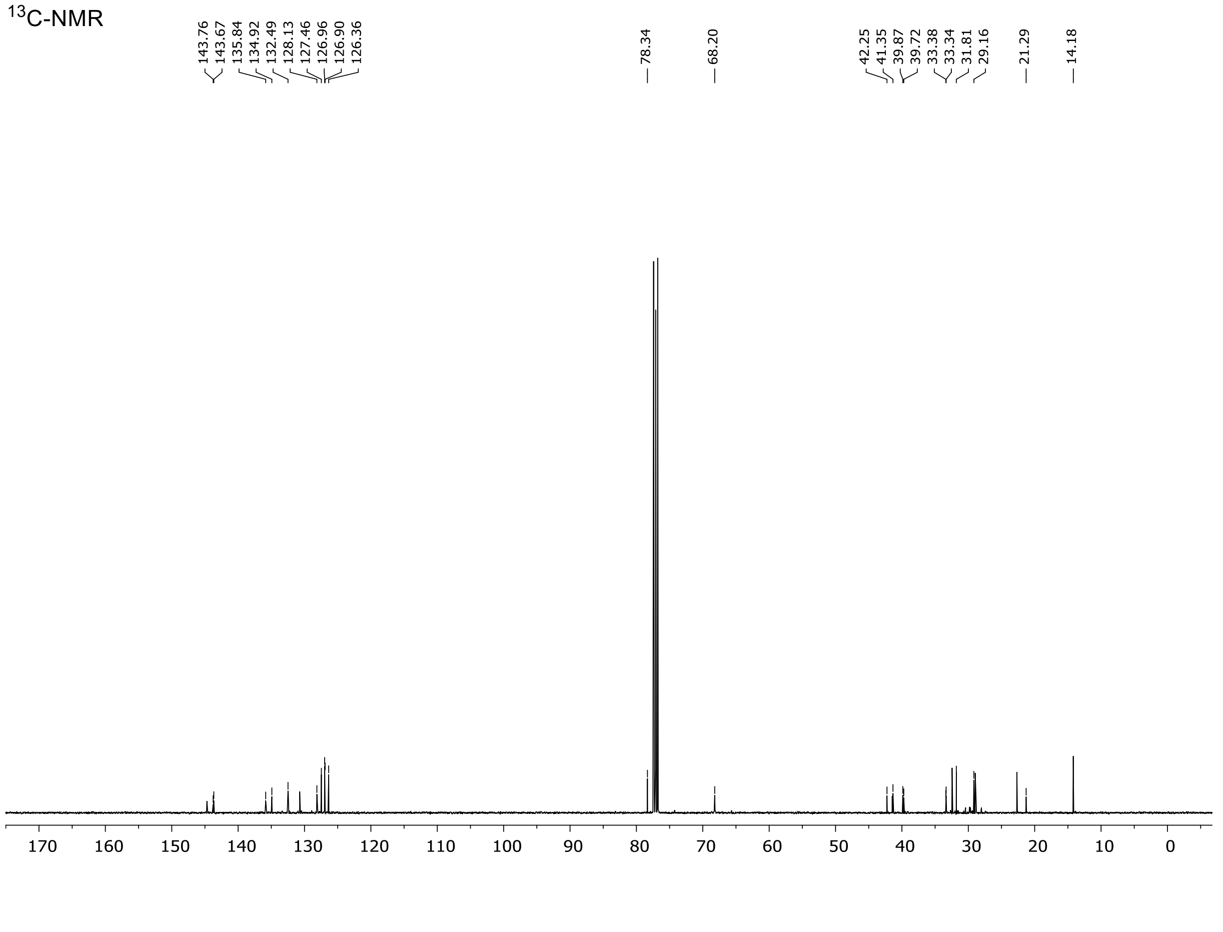

syn-4-(4-bromophenyl)-2-((E)-oct-1-en-1-yl)tetrahydro-2H-pyran 37

^{1}H-NMR

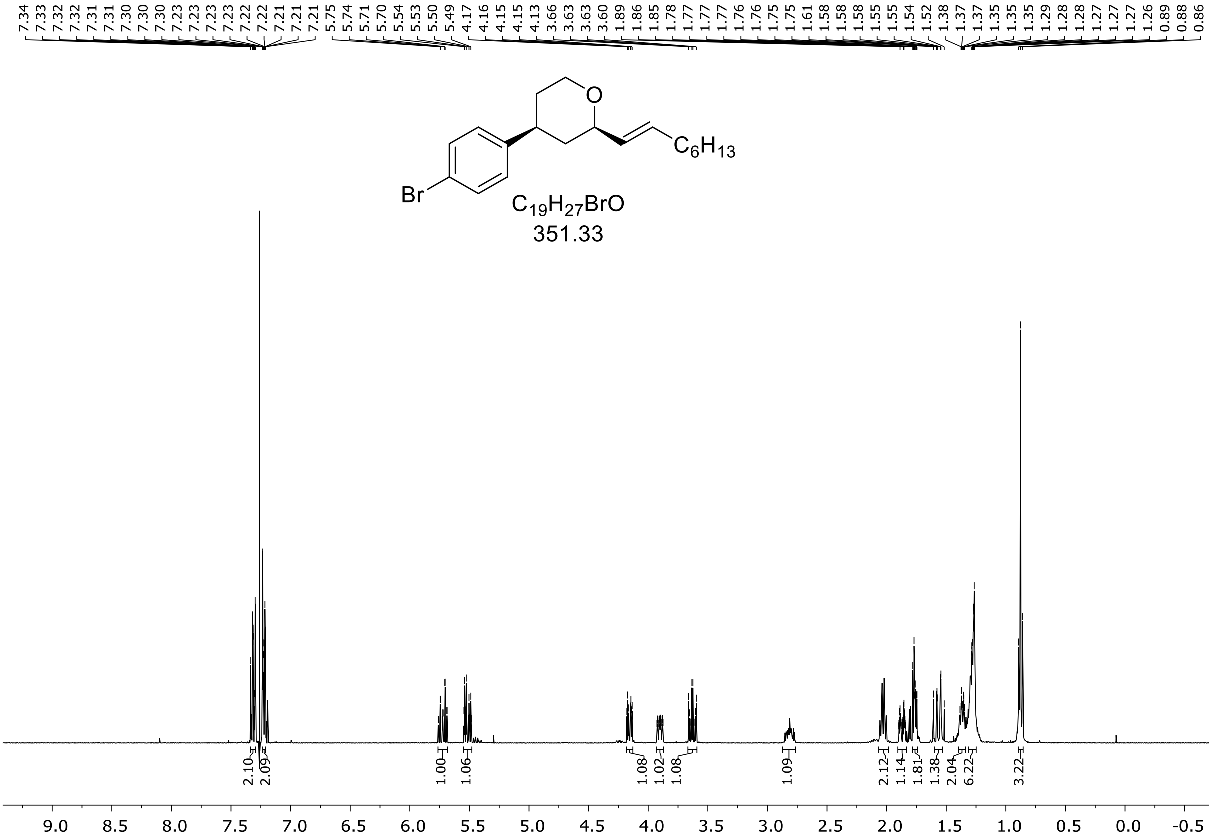

^{13}C-NMR

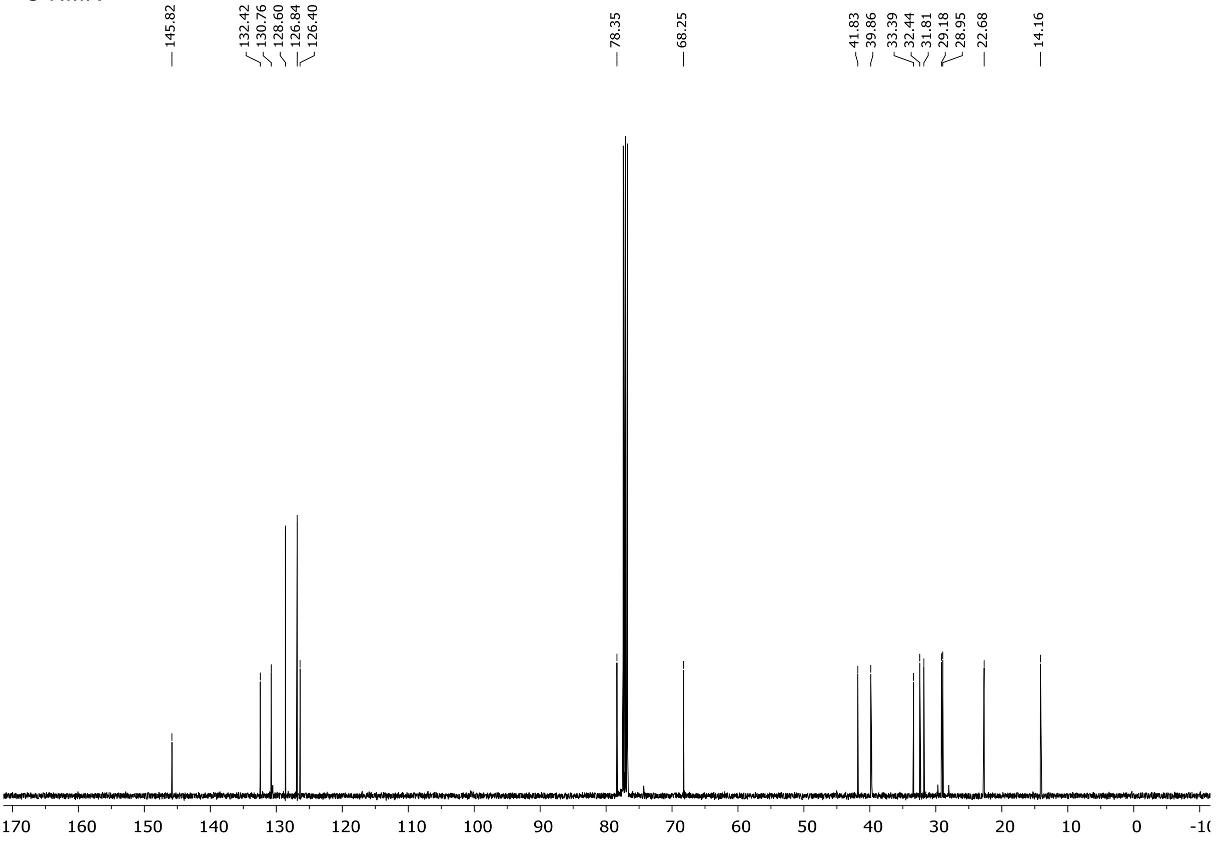

syn-4-(4-methoxyphenyl)-2-((E)-oct-1-en-1-yl)tetrahydro-2H-pyran 4

^{1}H-NMR

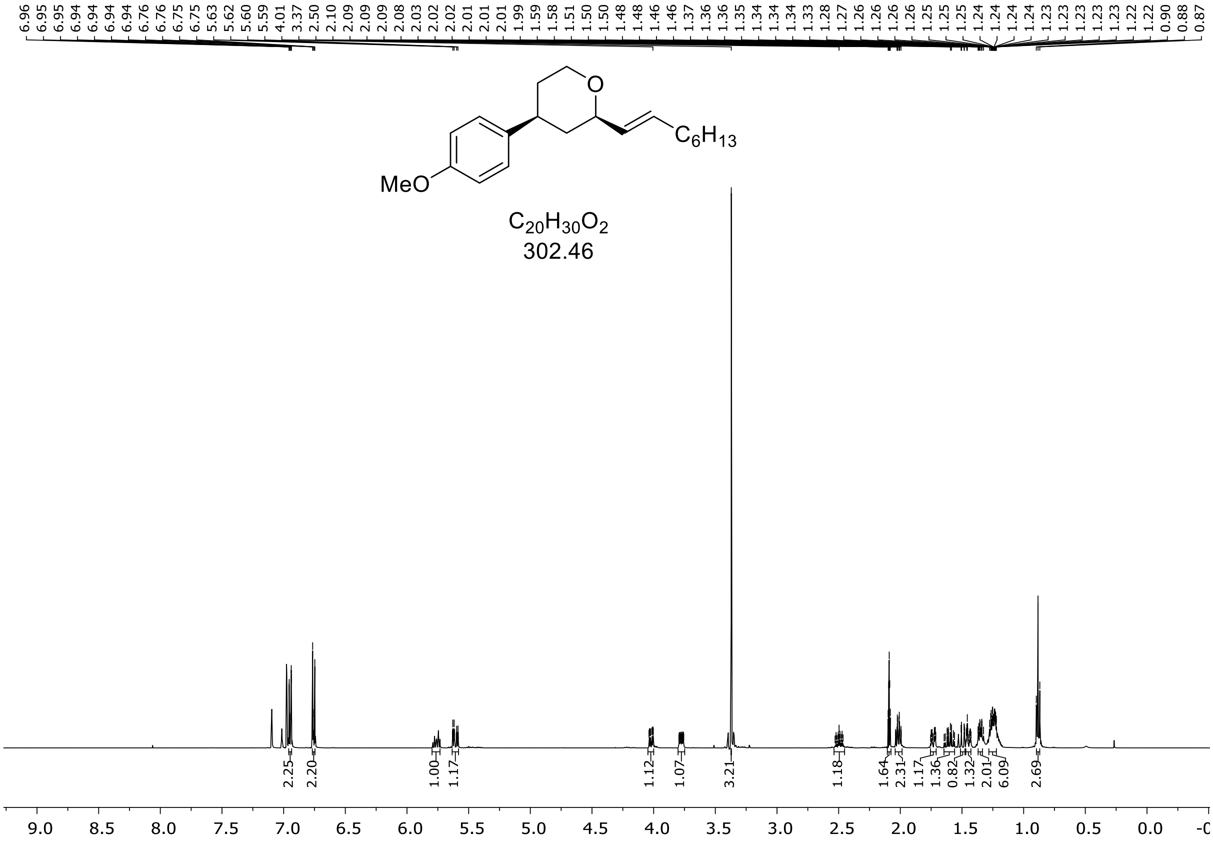

^{13}C-NMR

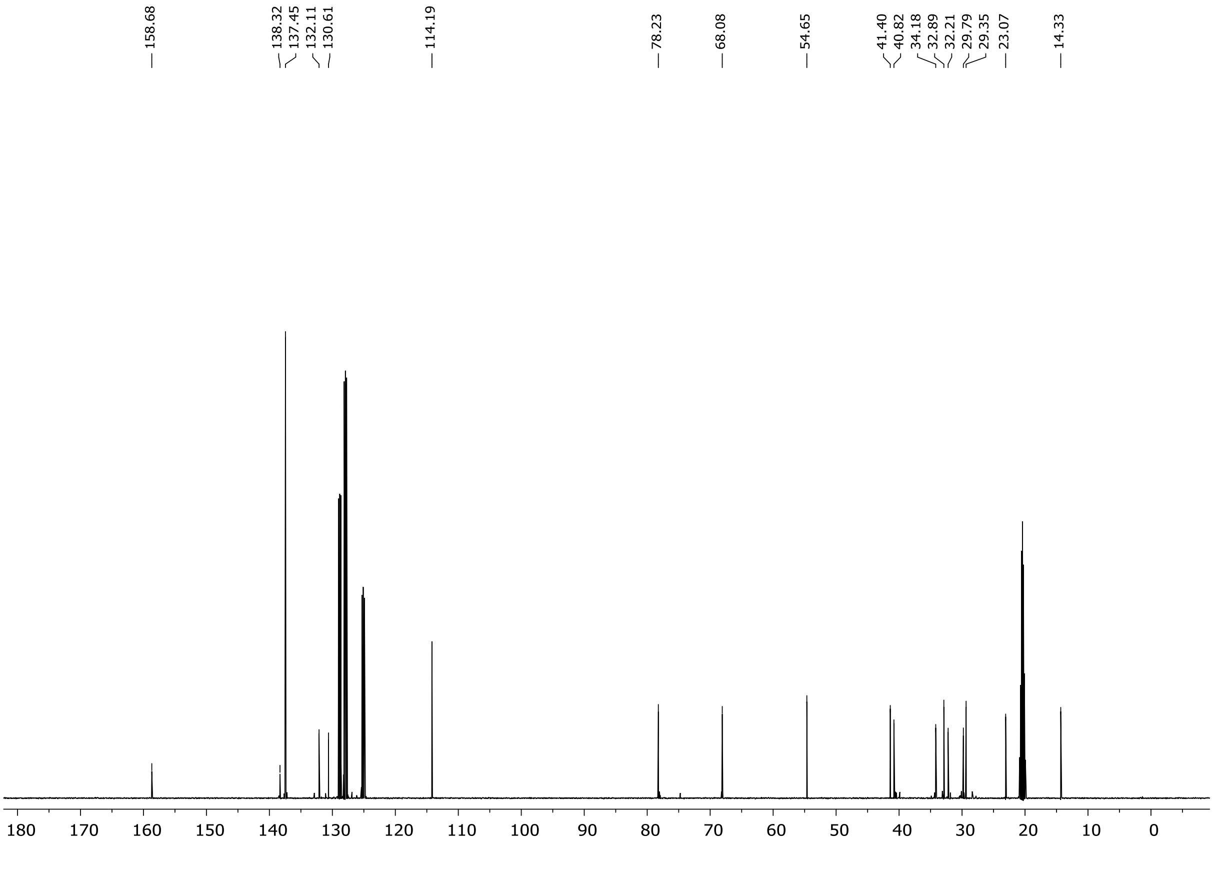

syn-4-(4-(methylthio)phenyl)-2-((E)-oct-1-en-1-yl)tetrahydro-2H-pyran 38

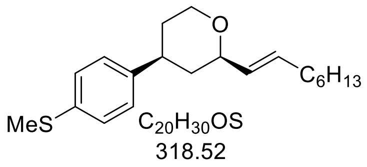

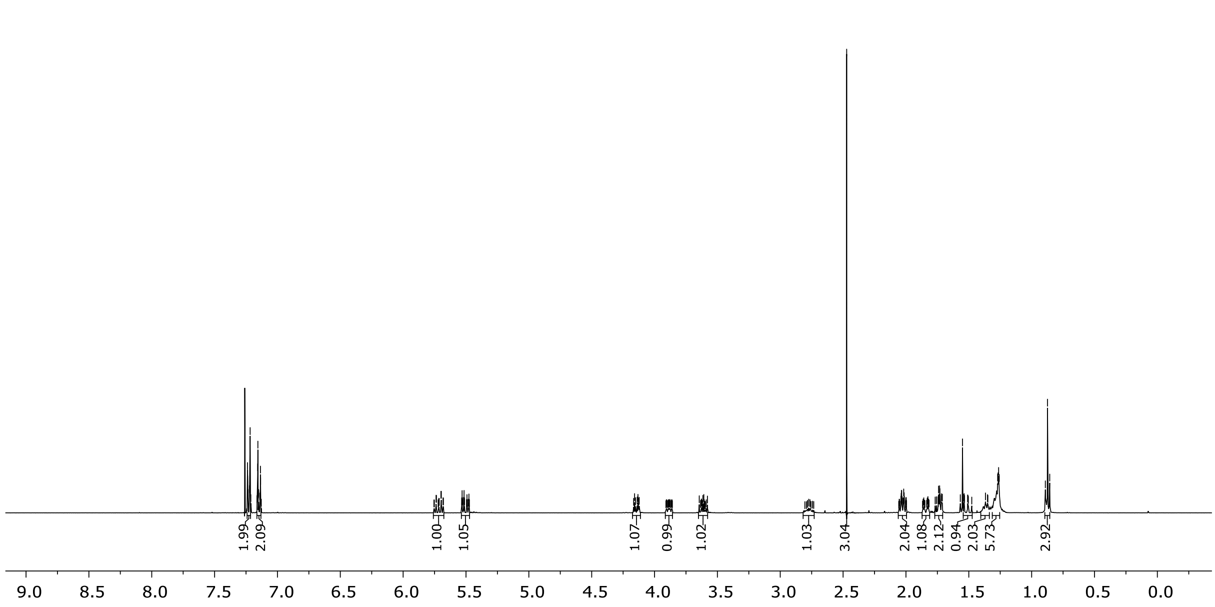

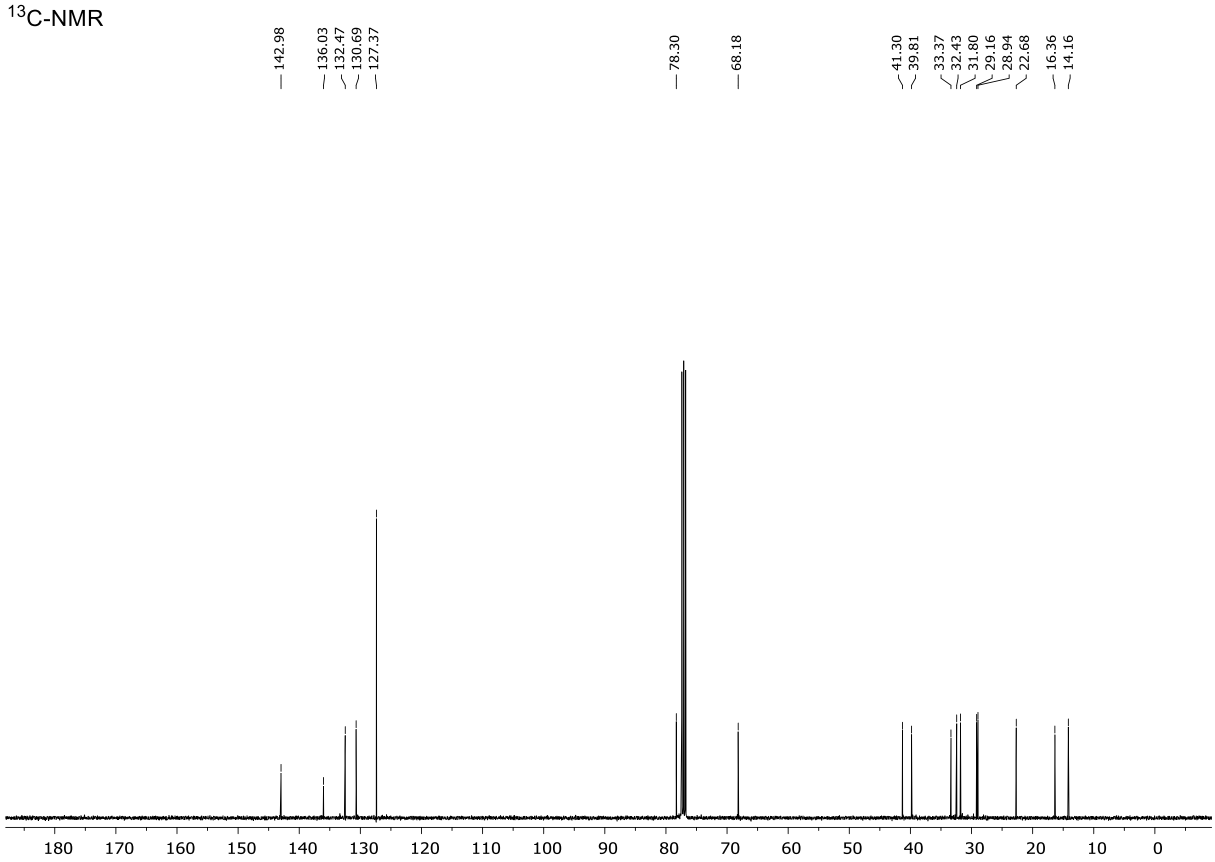

syn-2-((E)-oct-1-en-1-yl)-4-(4-(trifluoromethyl)phenyl)tetrahydro-2H-pyran 39

^{1}H-NMR

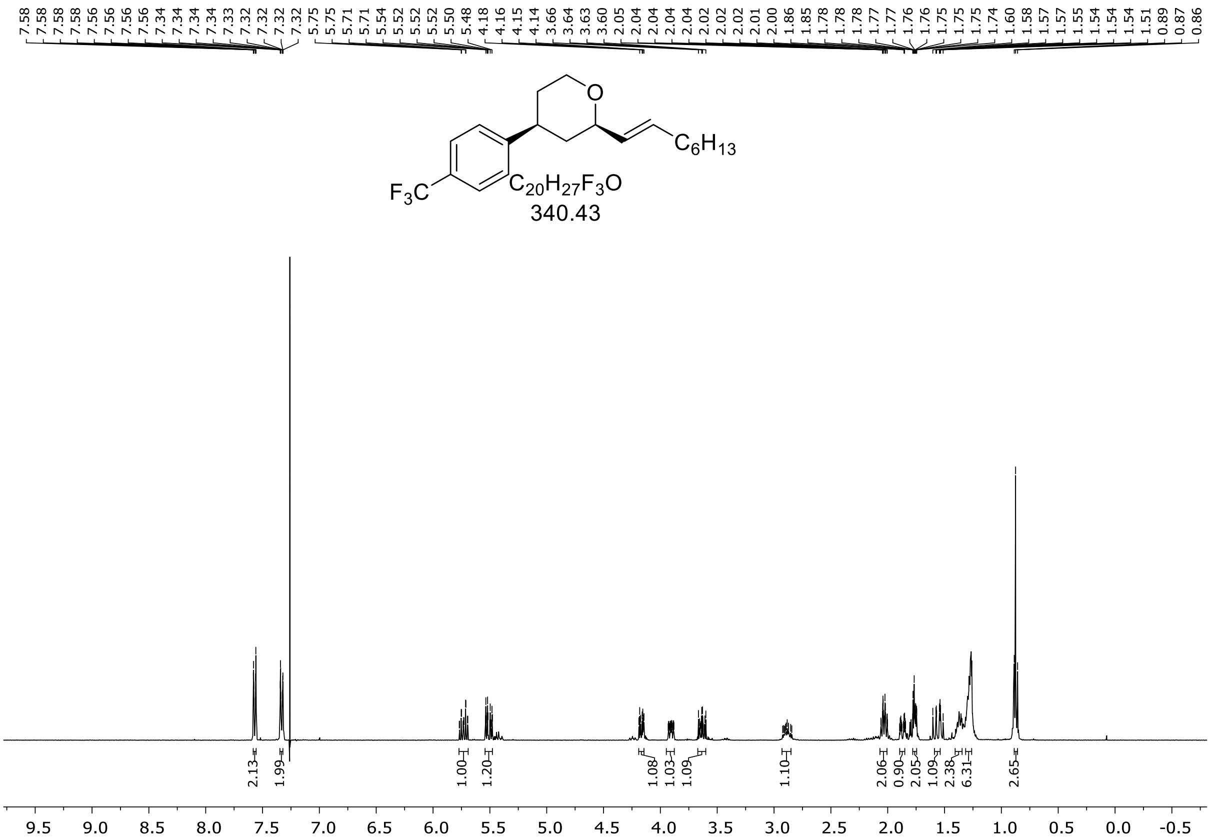

^{13}C-NMR

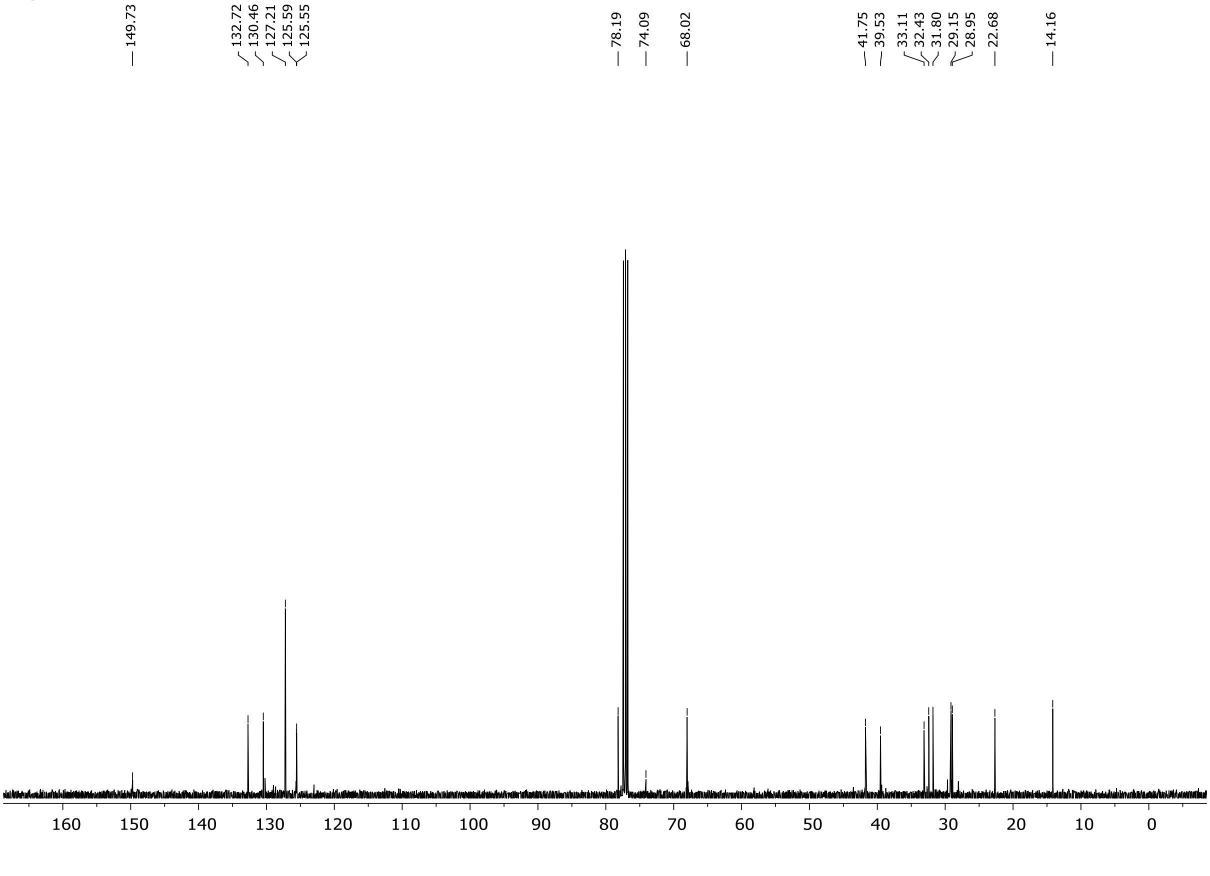

syn-2,2-dimethyl-6-((E)-oct-1-en-1-yl)-4-(4-(trifluoromethyl)phenyl)tetrahydro-2H-pyran 40

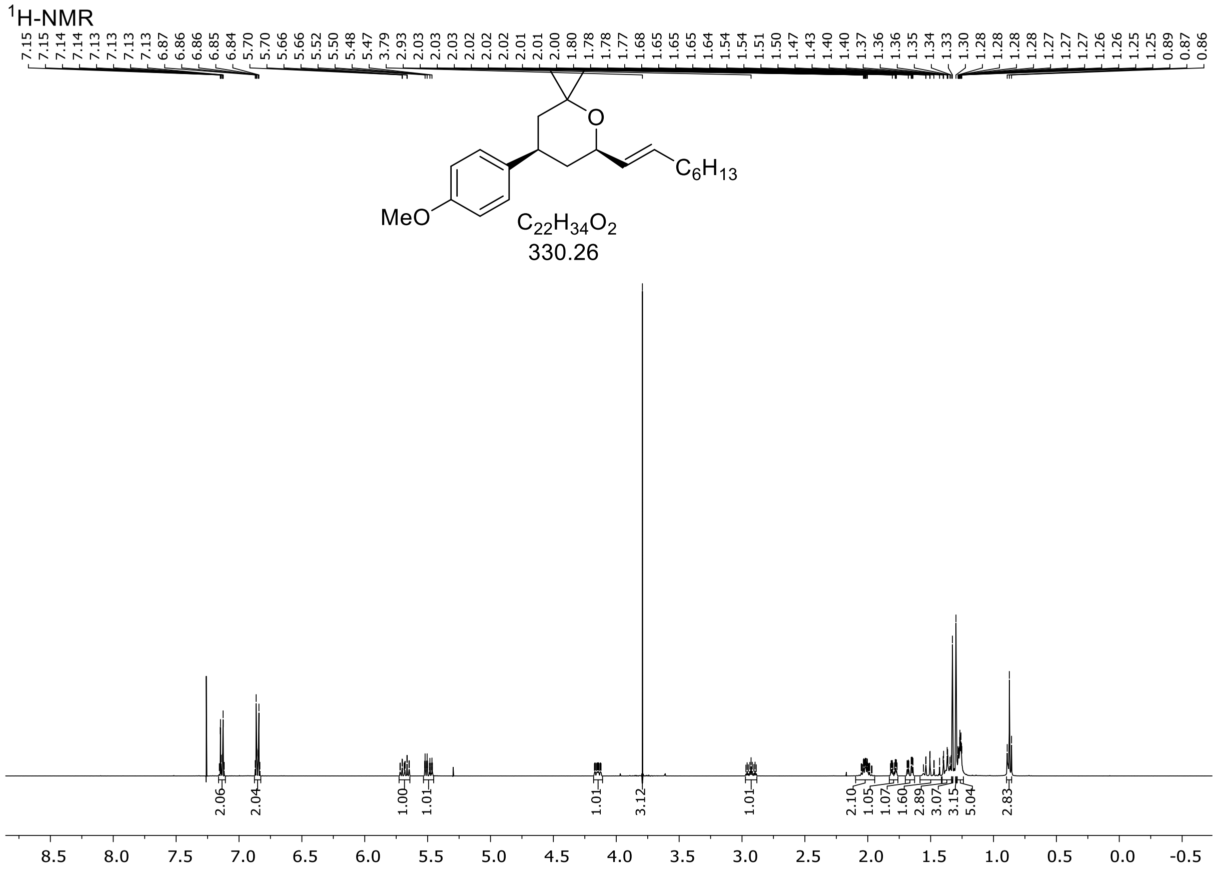

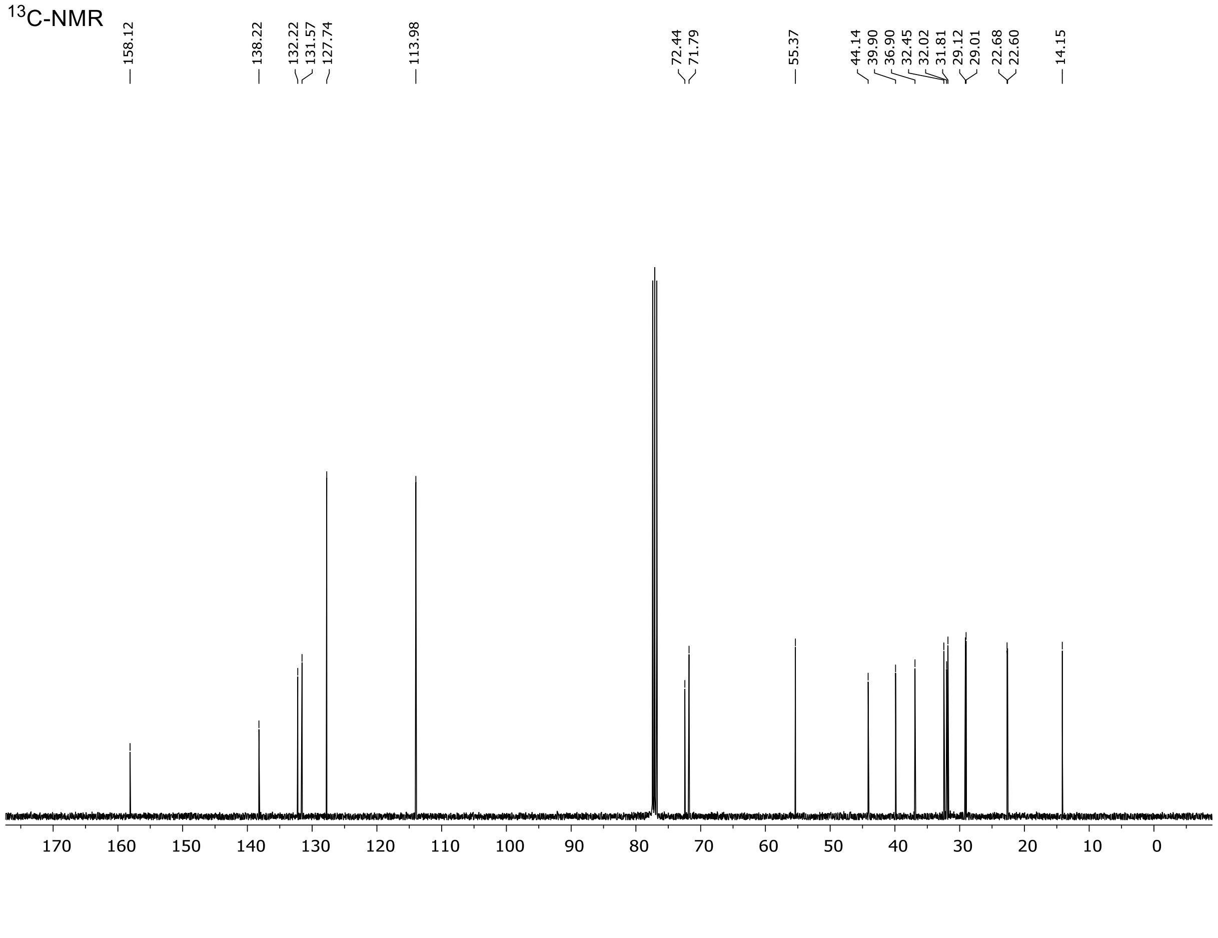

syn-2-((E)-2-cyclohexylvinyl)-4-(4-methoxyphenyl)tetrahydro-2H-pyran 41

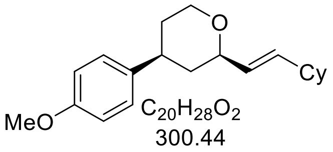

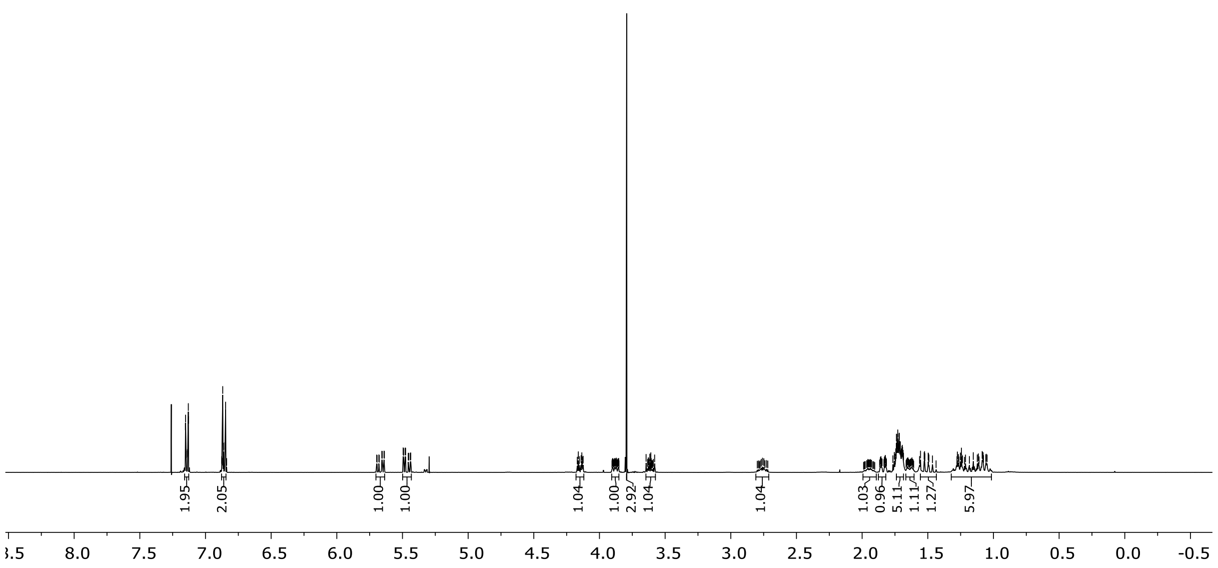

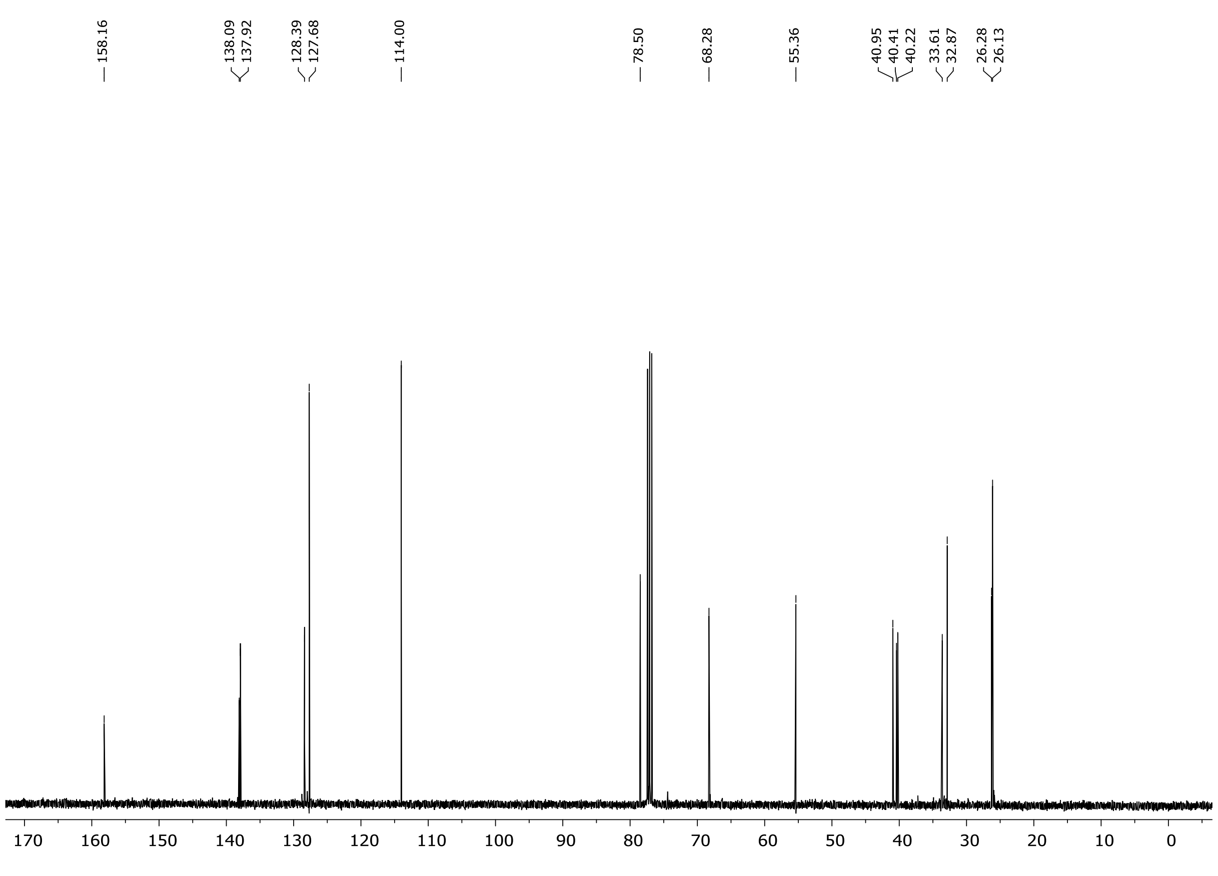

syn-4-(4-methoxyphenyl)-2-((E)-4-phenylbut-1-en-1-yl)tetrahydro-2H-pyran 42

^{1}H-NMR

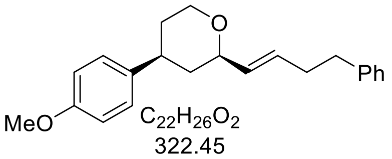

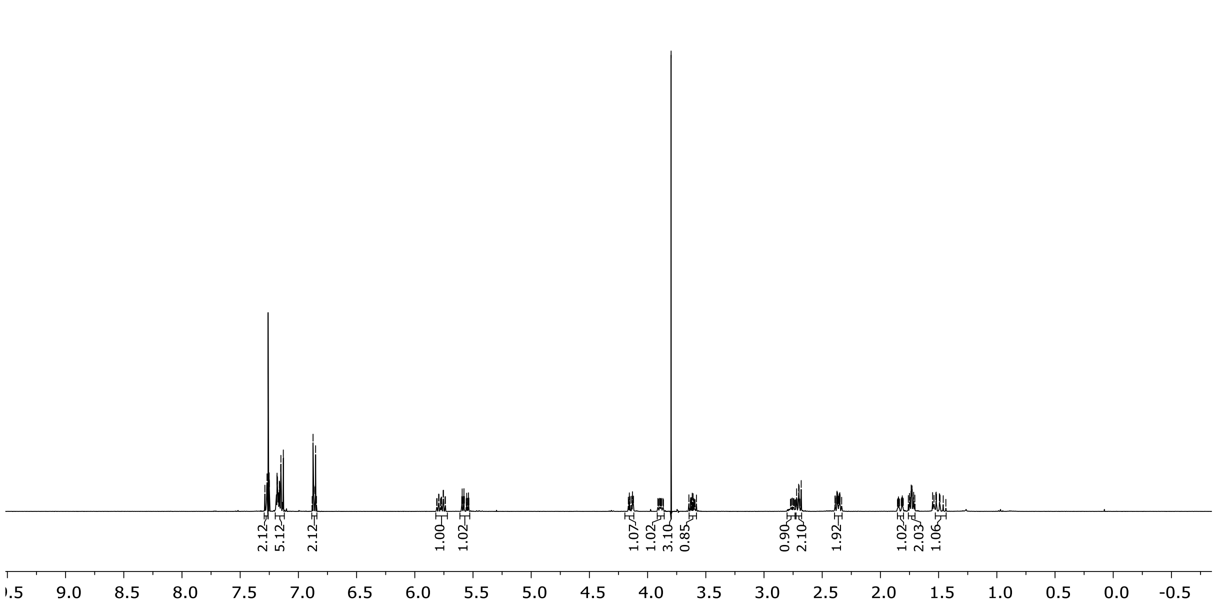

^{13}C-NMR

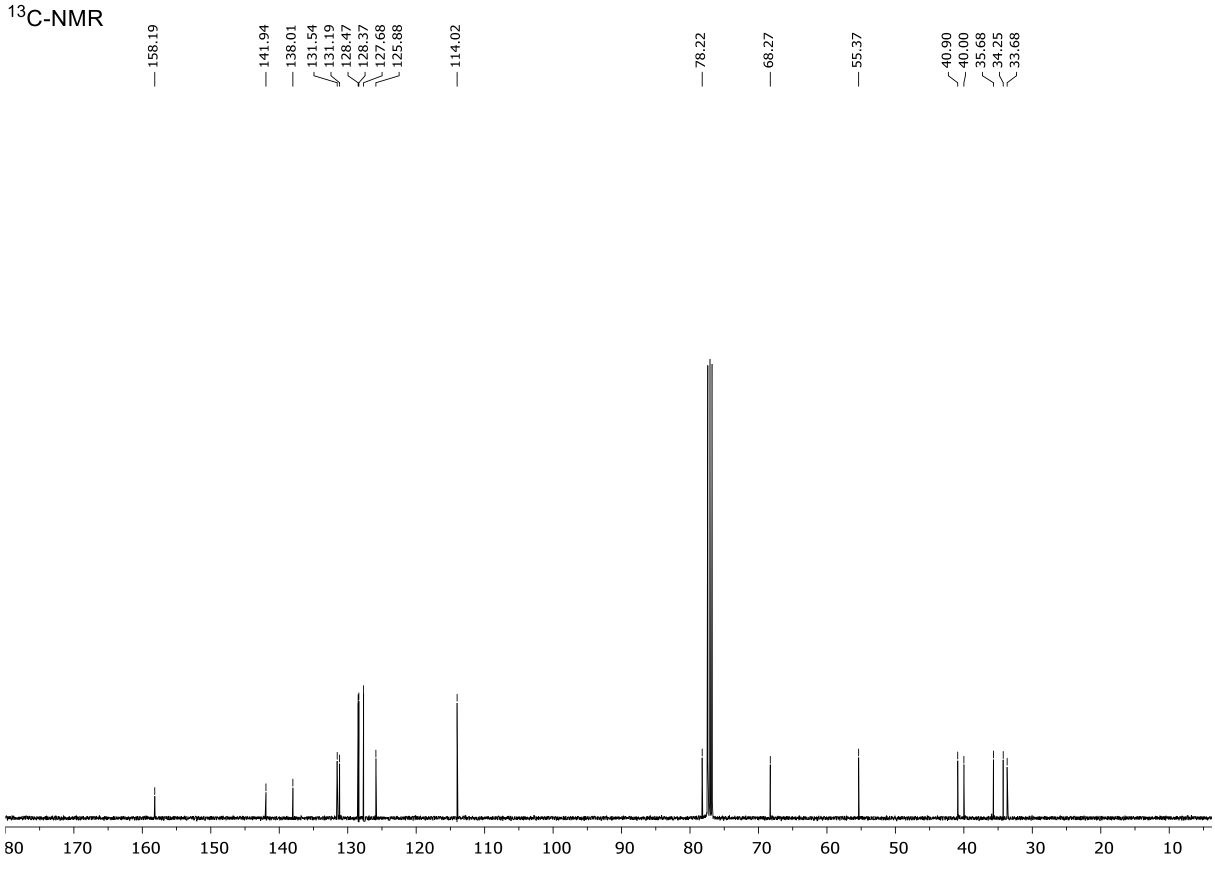

syn-4-(4-methoxyphenyl)-2-((E)-3-phenylprop-1-en-1-yl)tetrahydro-2H-pyran 43

^{1}H-NMR

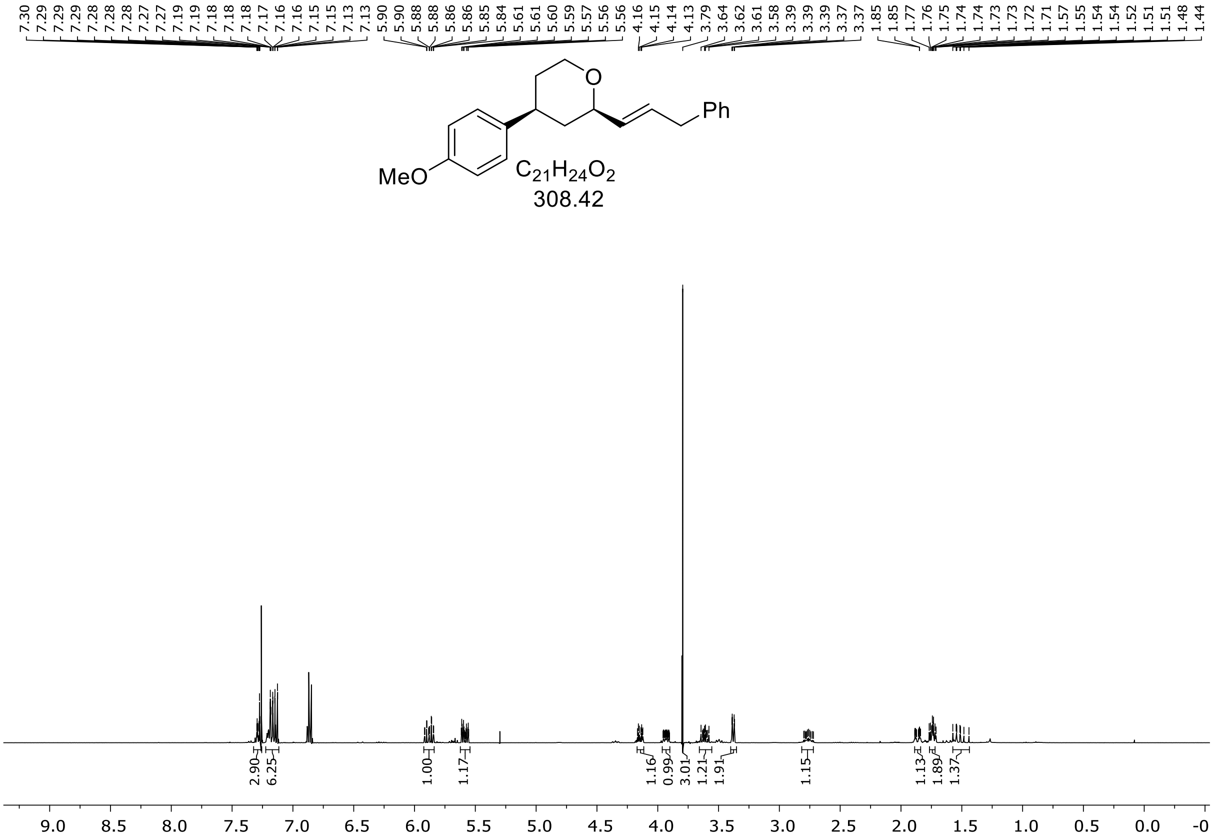

^{13}C-NMR

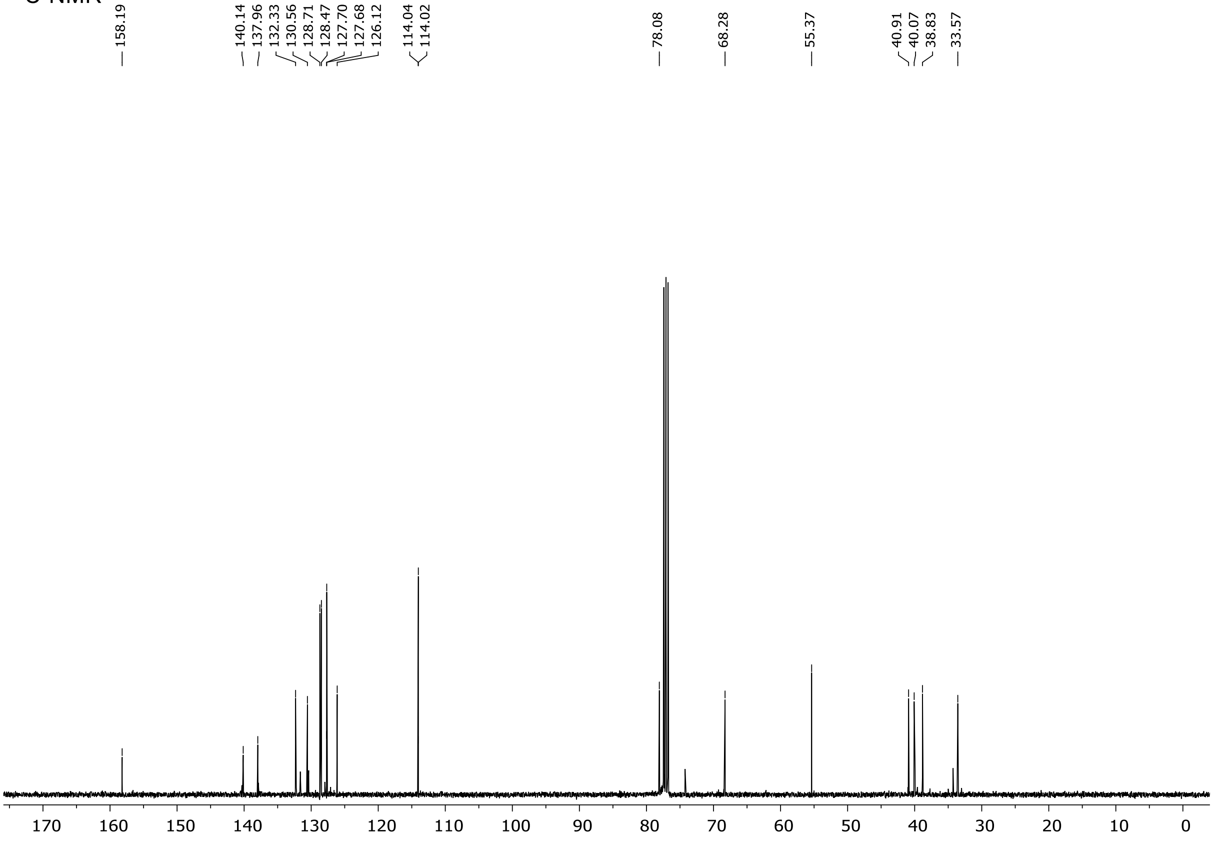

syn-4-(4-methoxyphenyl)-2-((E)-5-(methylthio)pent-1-en-1-yl)tetrahydro-2H-pyran 44

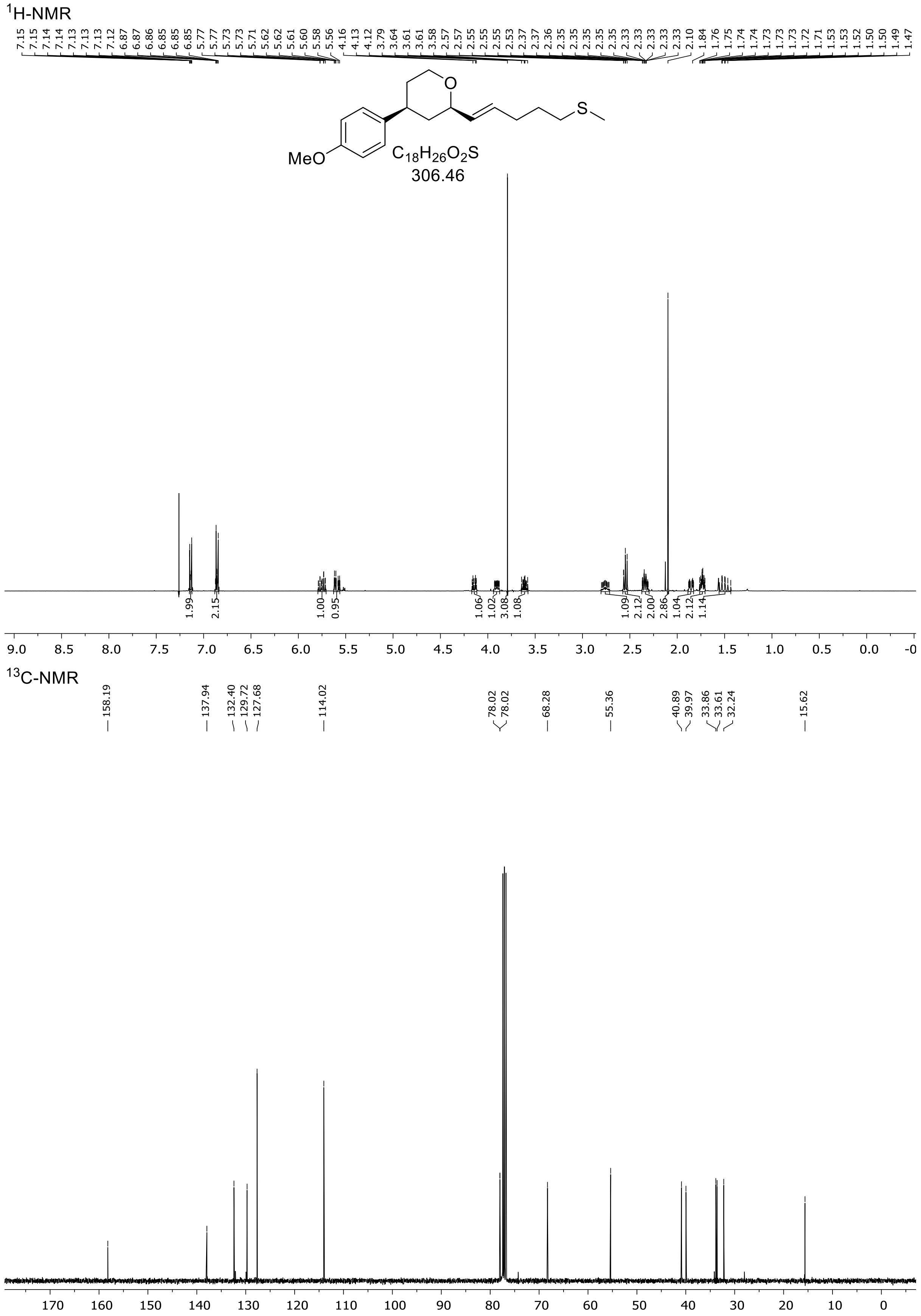

4-(4-methoxyphenyl)-3,3-dimethyl-2-vinyltetrahydro-2H-pyran 45A

^{1}H-NMR

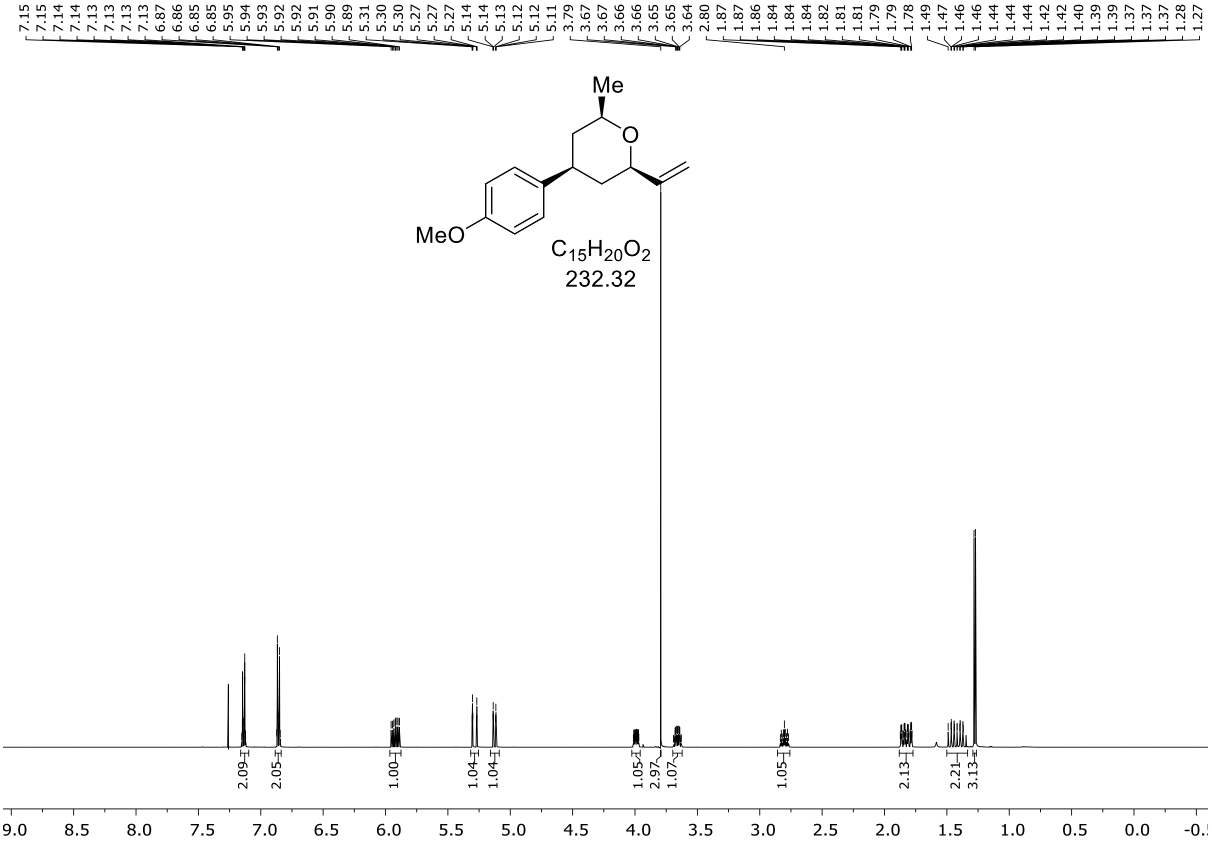

^{13}C-NMR

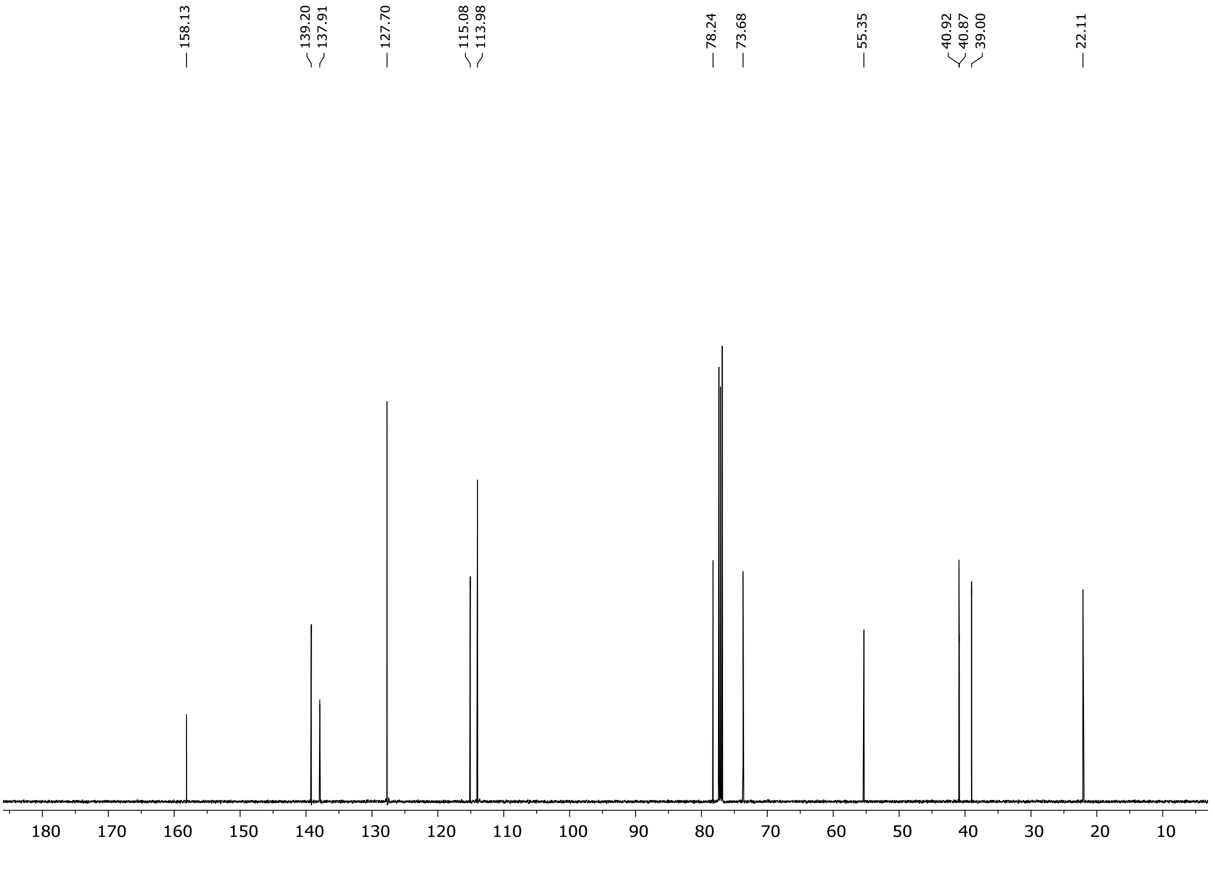

4-(4-methoxyphenyl)-3,3-dimethyl-2-vinyltetrahydro-2H-pyran 49A

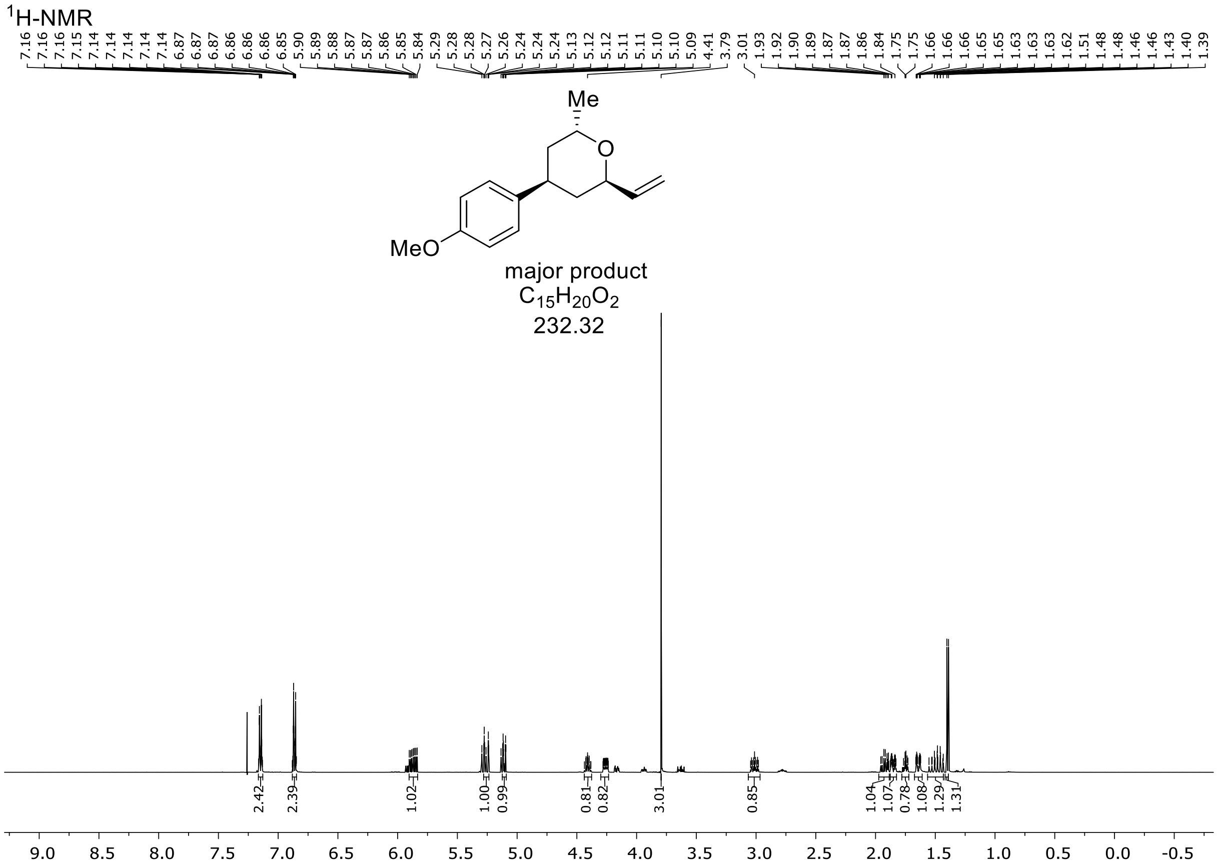

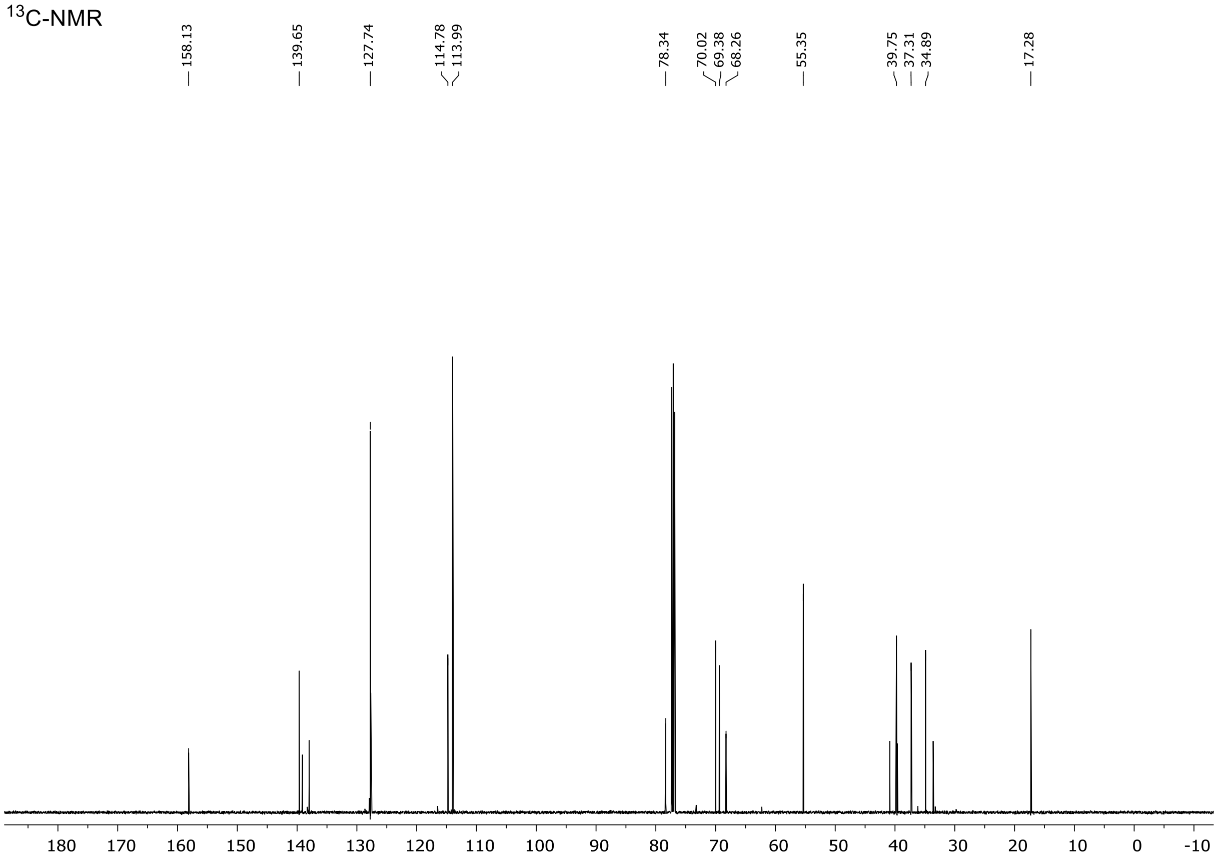

4-(4-methoxyphenyl)-3,3-dimethyl-2-vinyltetrahydro-2H-pyran 49B

^{1}H-NMR

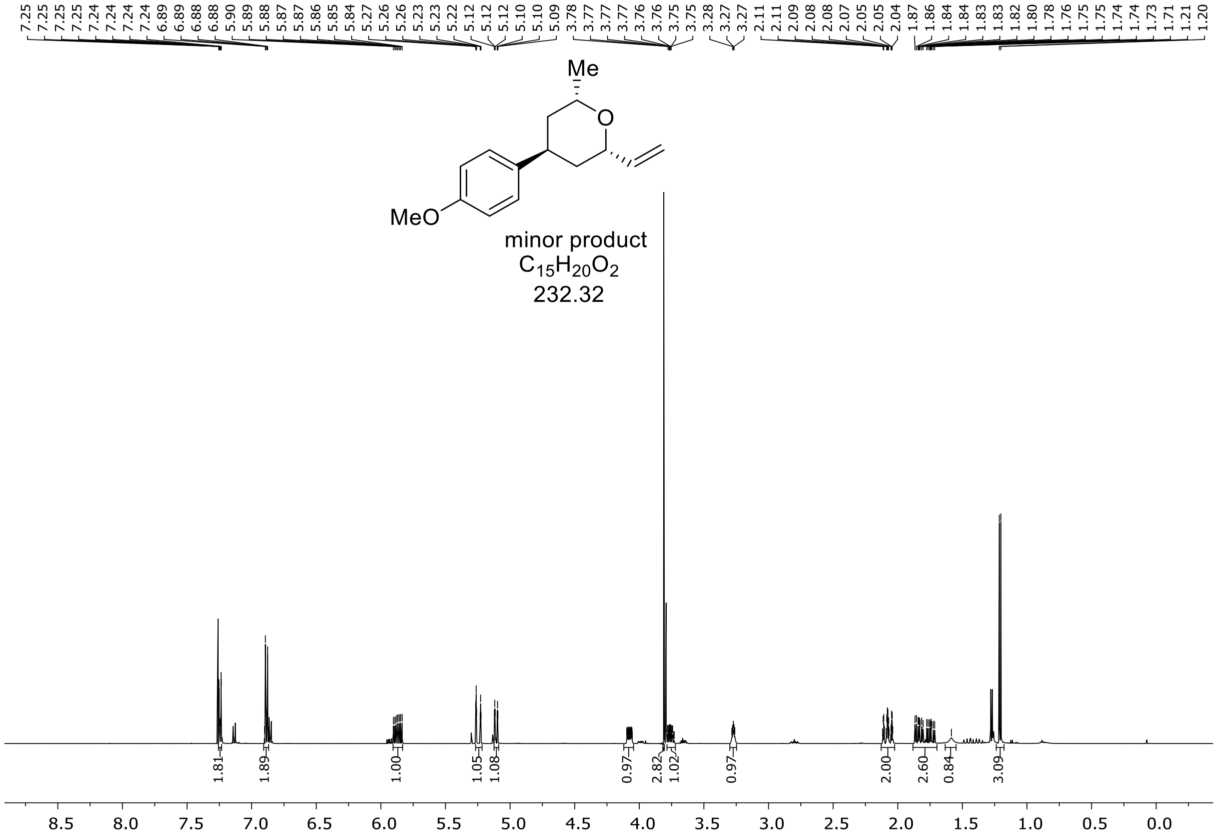

^{13}C-NMR

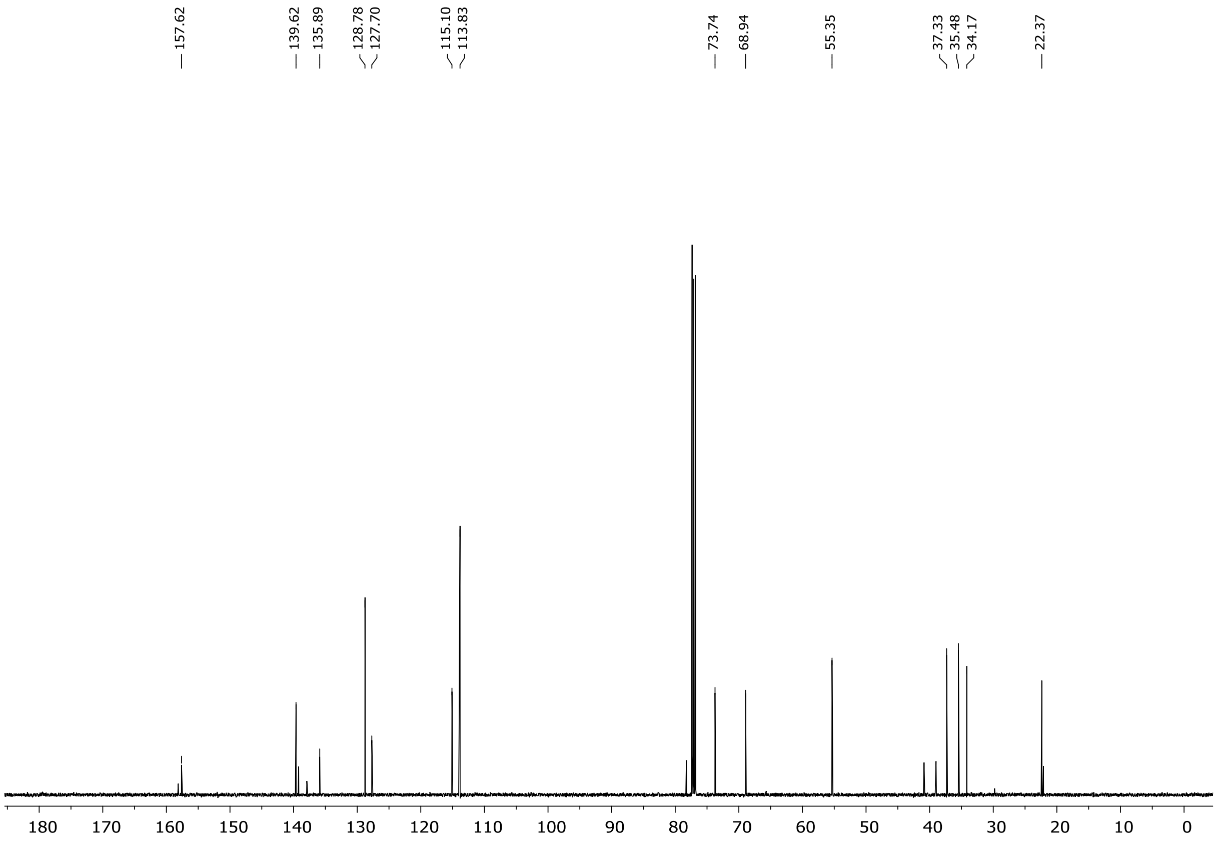

4-(4-methoxyphenyl)-2-phenyl-6-vinyltetrahydro-2H-pyran 46A

^{1}H-NMR

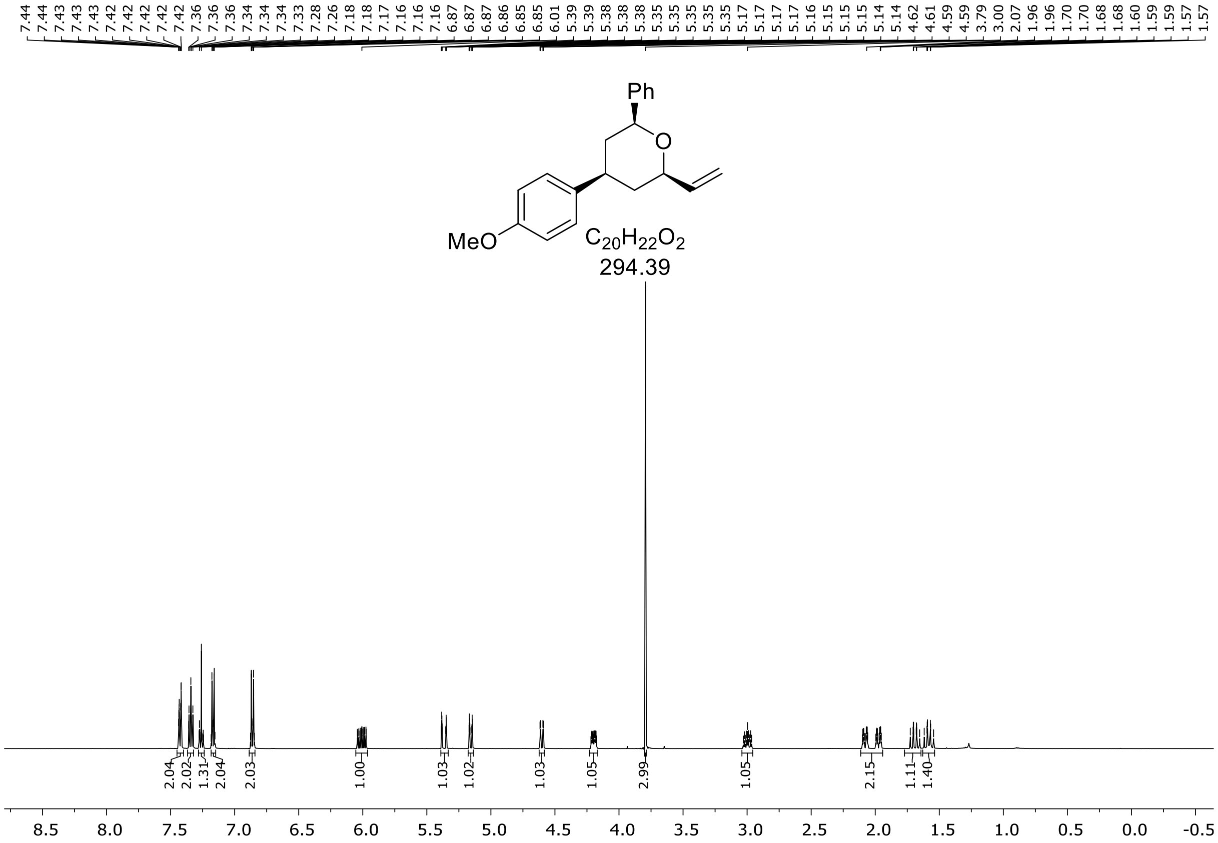

^{13}C-NMR

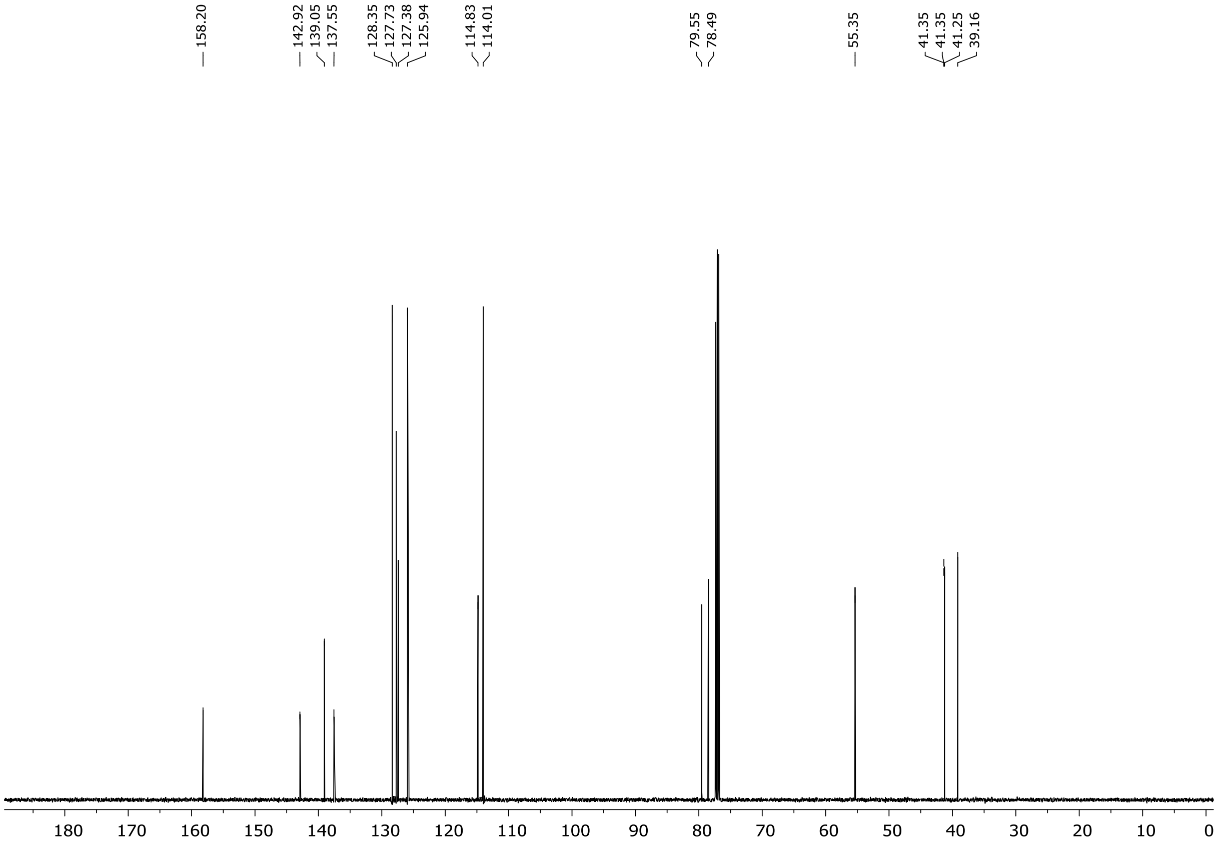

4-(4-methoxyphenyl)-2-phenyl-6-vinyltetrahydro-2H-pyran 50A

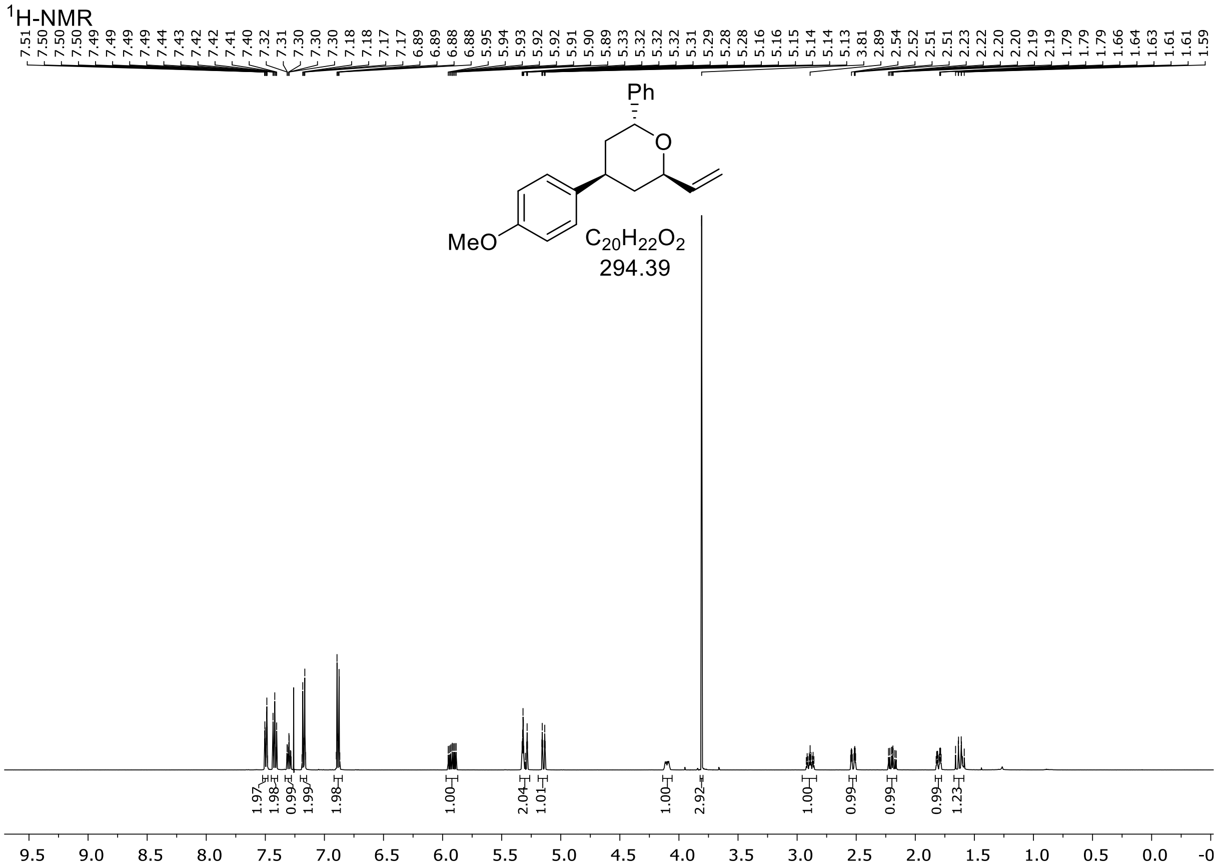

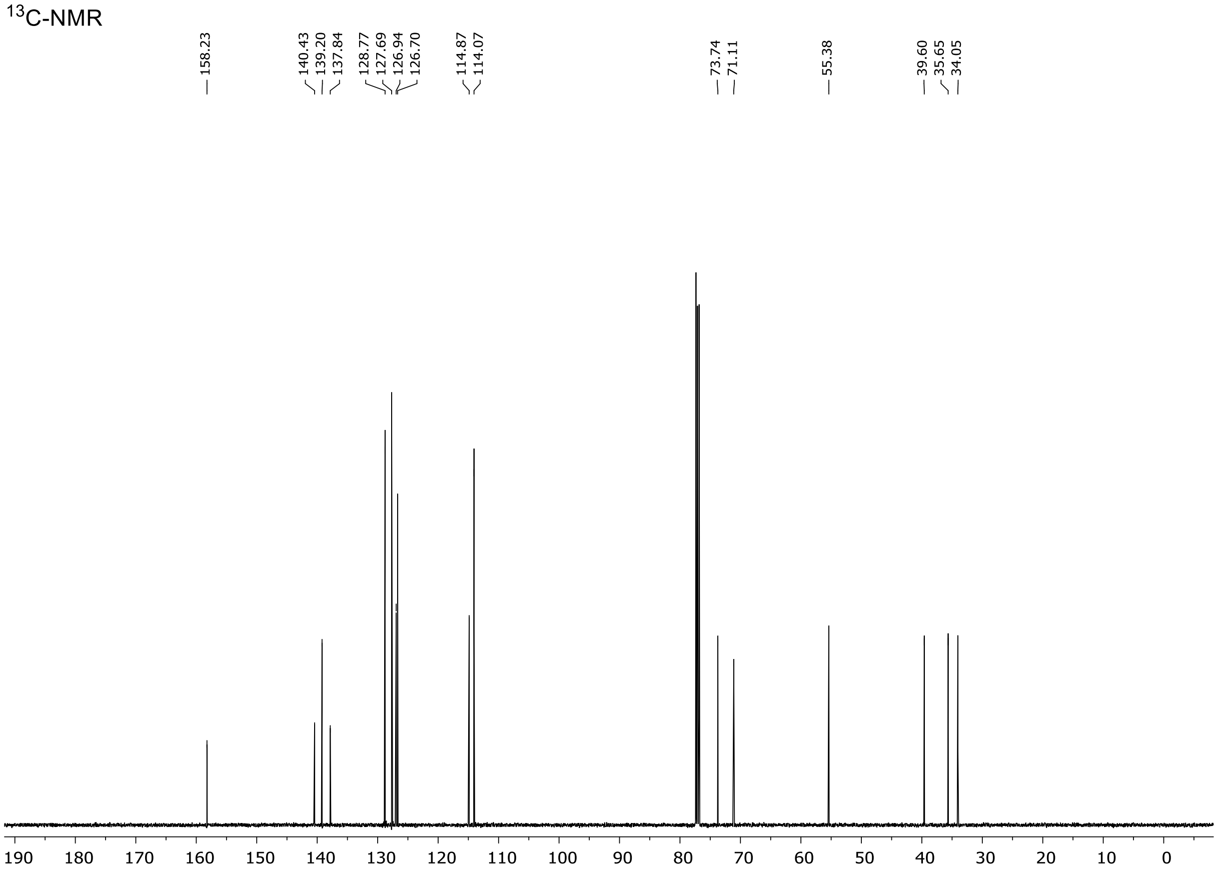

4-(4-methoxyphenyl)-2-phenyl-6-vinyltetrahydro-2H-pyran 50B

^{1}H-NMR

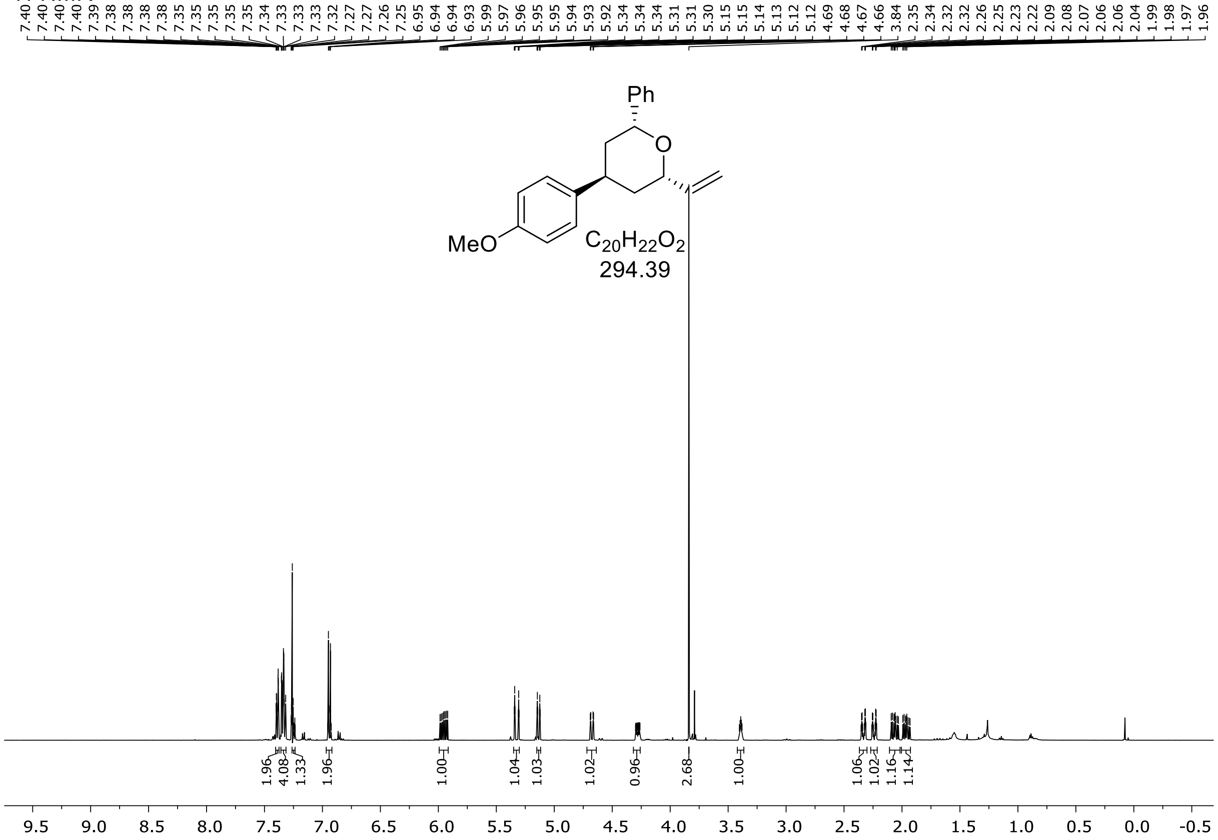

^{13}C-NMR

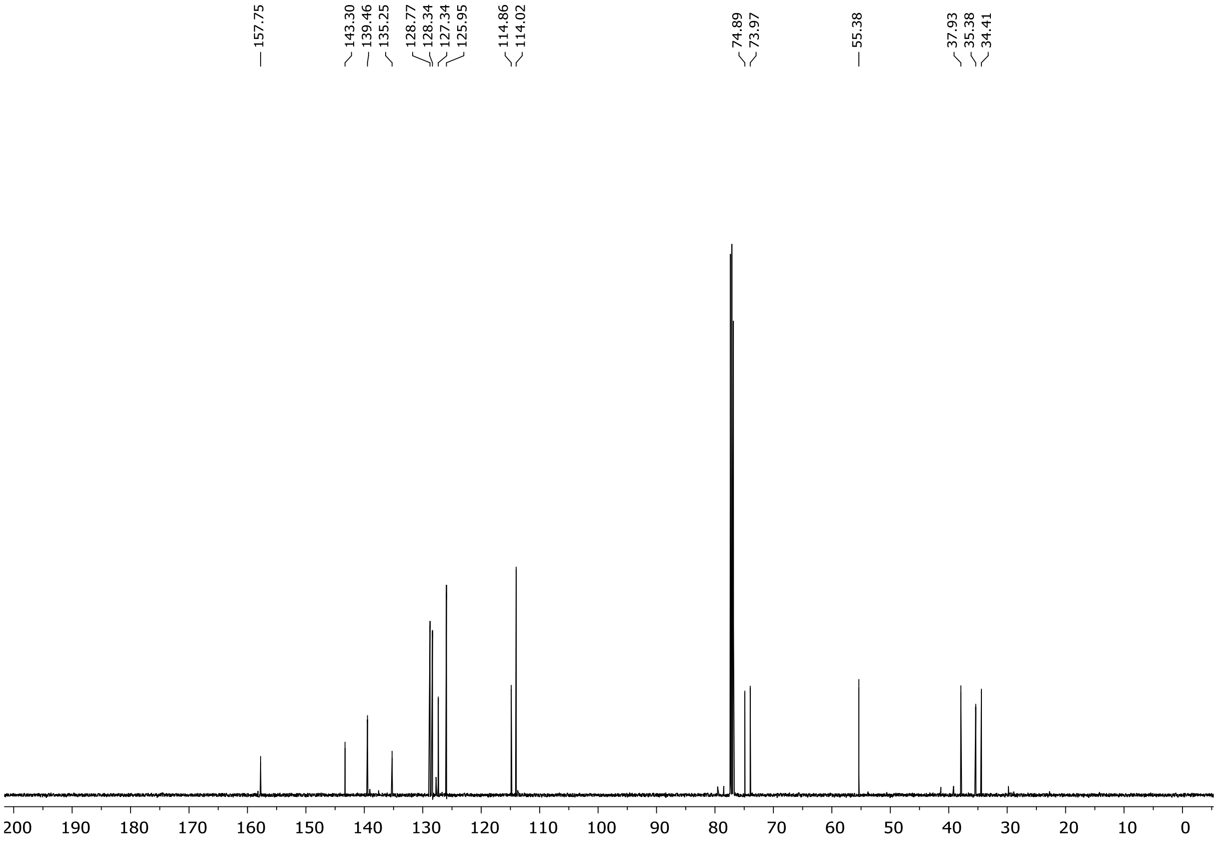

4-(4-methoxyphenyl)-2-methyl-6-((E)-oct-1-en-1-yl)tetrahydro-2H-pyran 47A

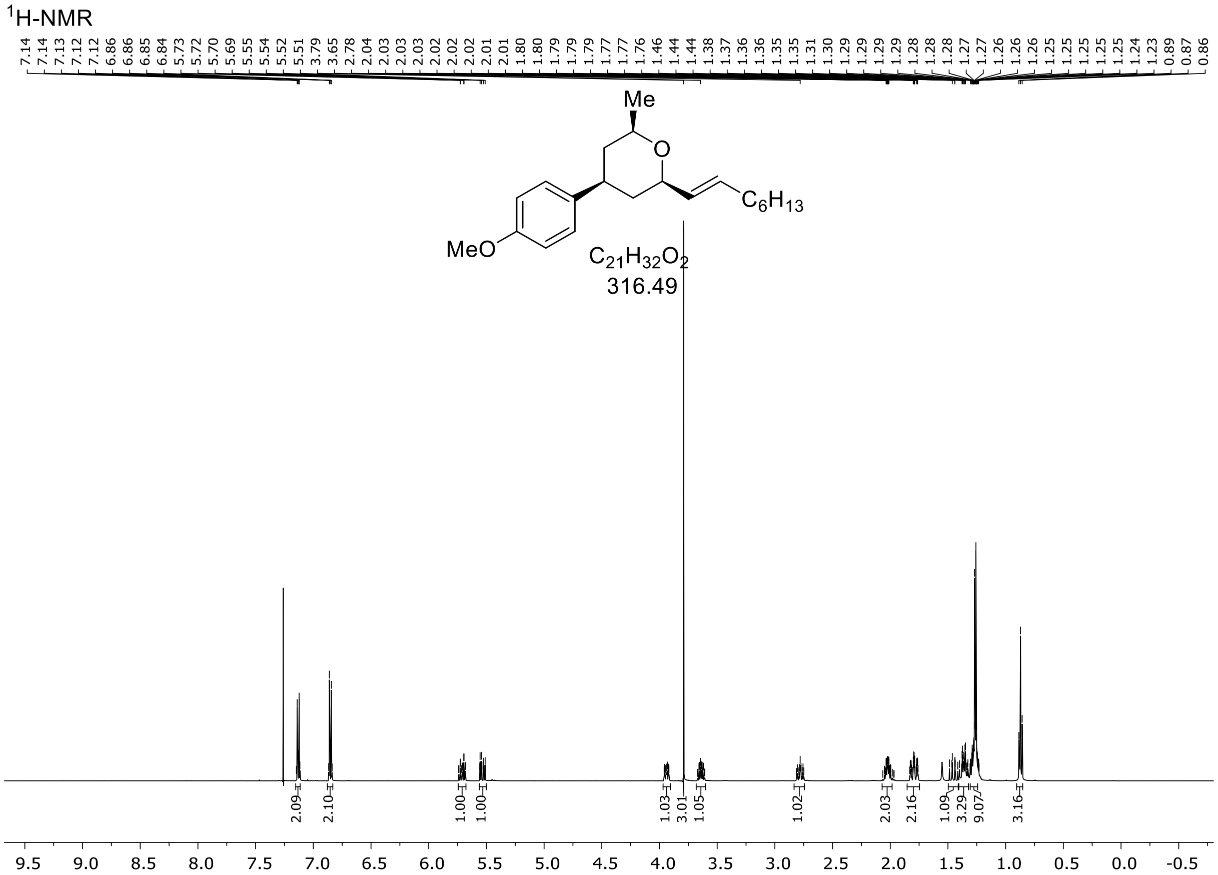

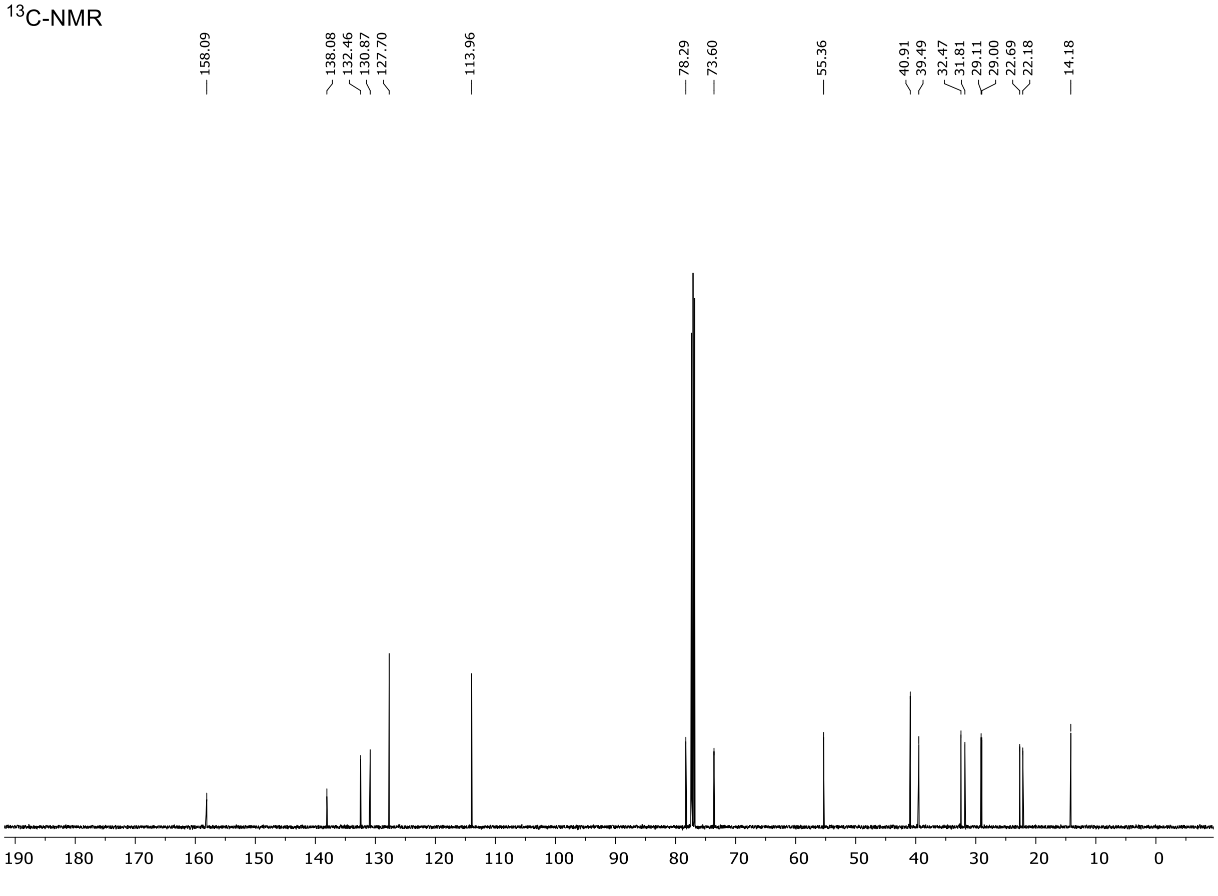

4-(4-methoxyphenyl)-2-methyl-6-((E)-oct-1-en-1-yl)tetrahydro-2H-pyran 51A

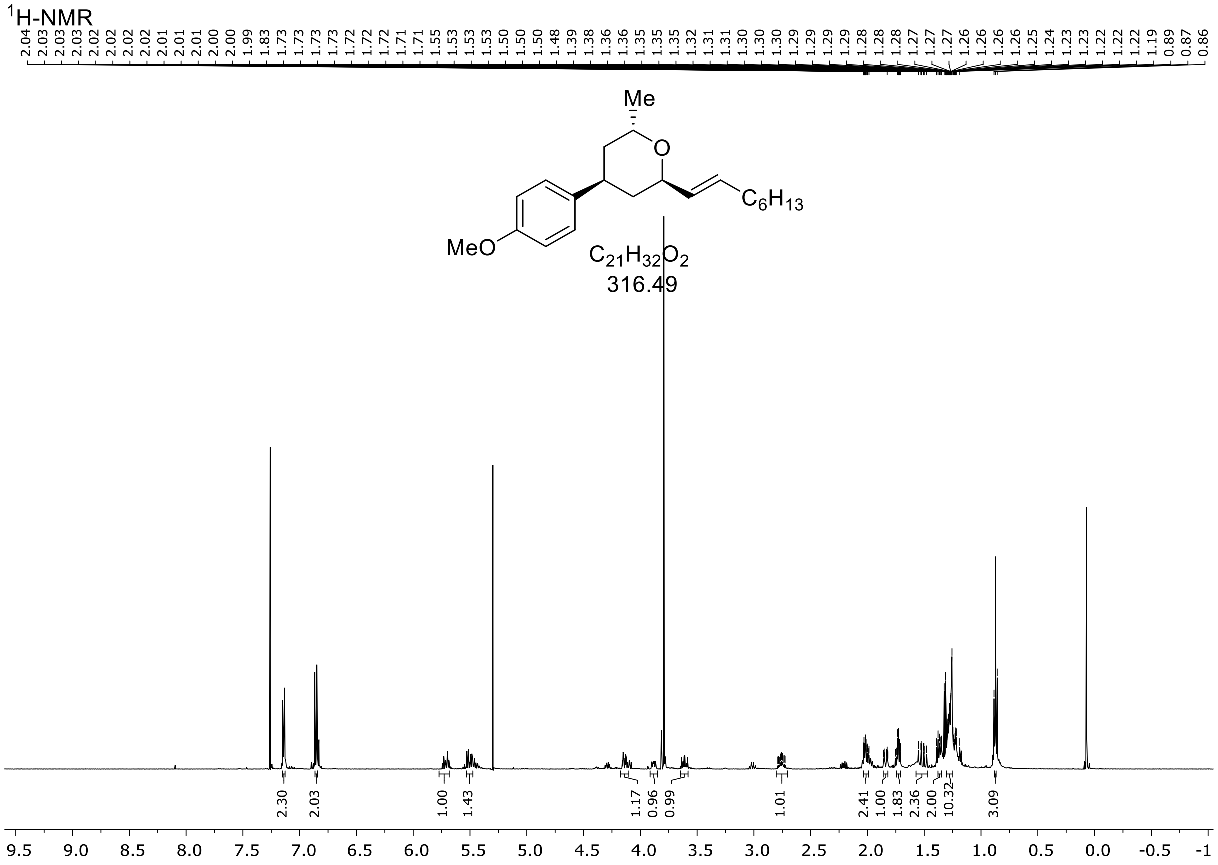

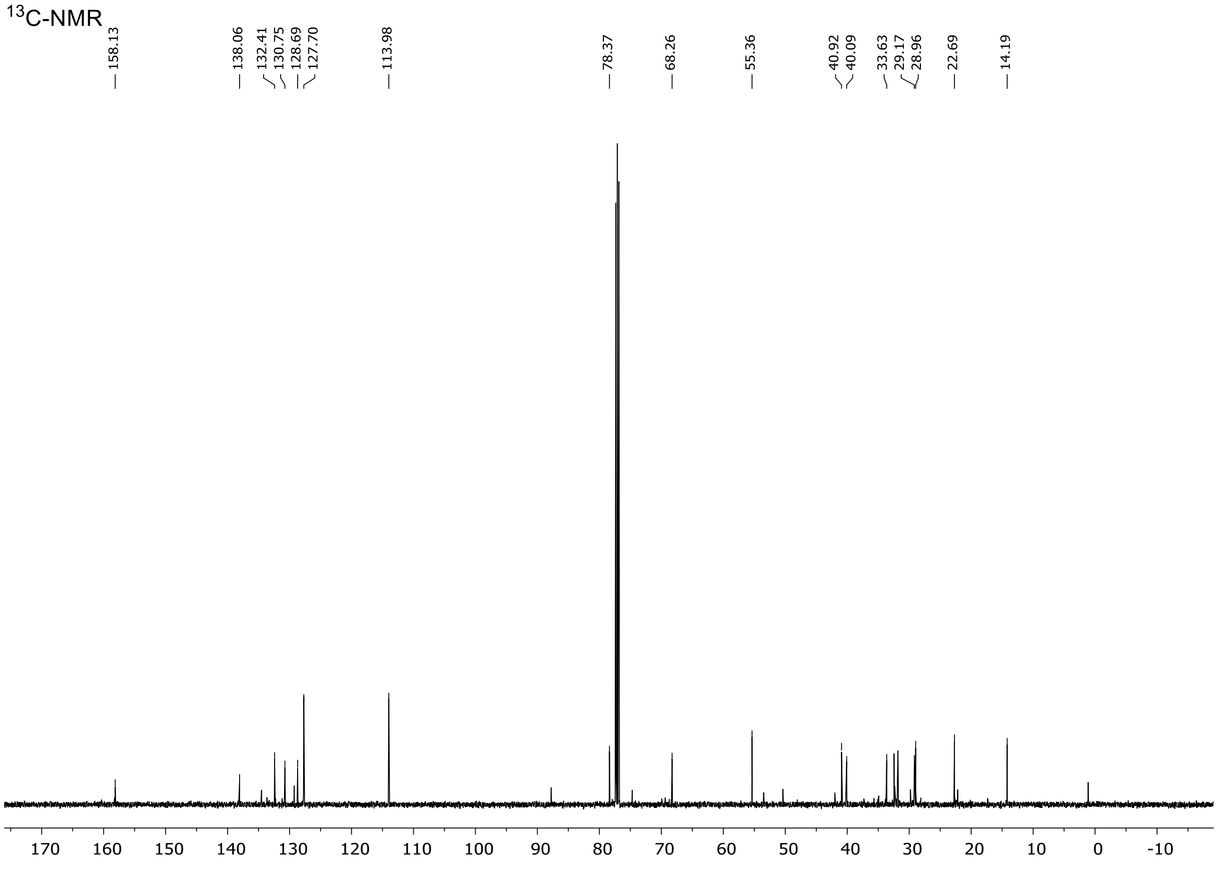

4-(4-methoxyphenyl)-2-methyl-6-((E)-oct-1-en-1-yl)tetrahydro-2H-pyran 51B

^{1}H-NMR

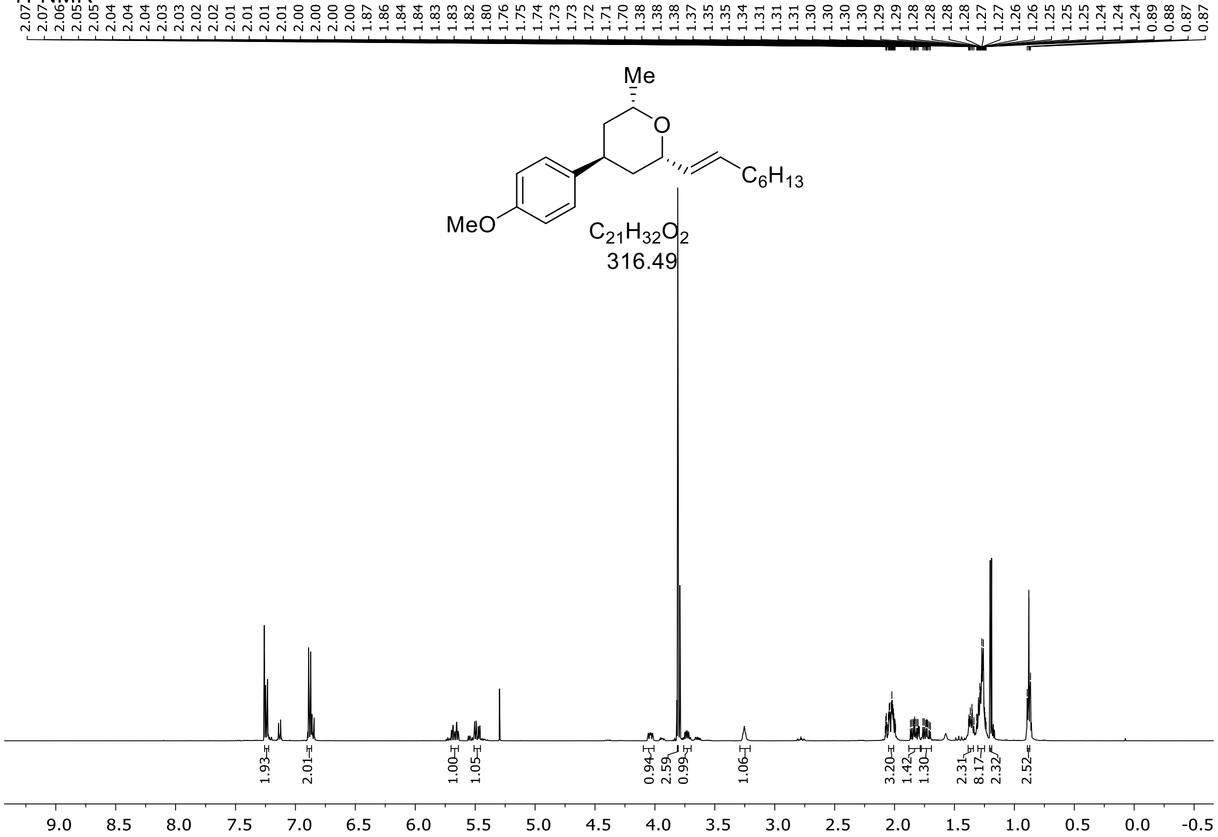

^{13}C-NMR

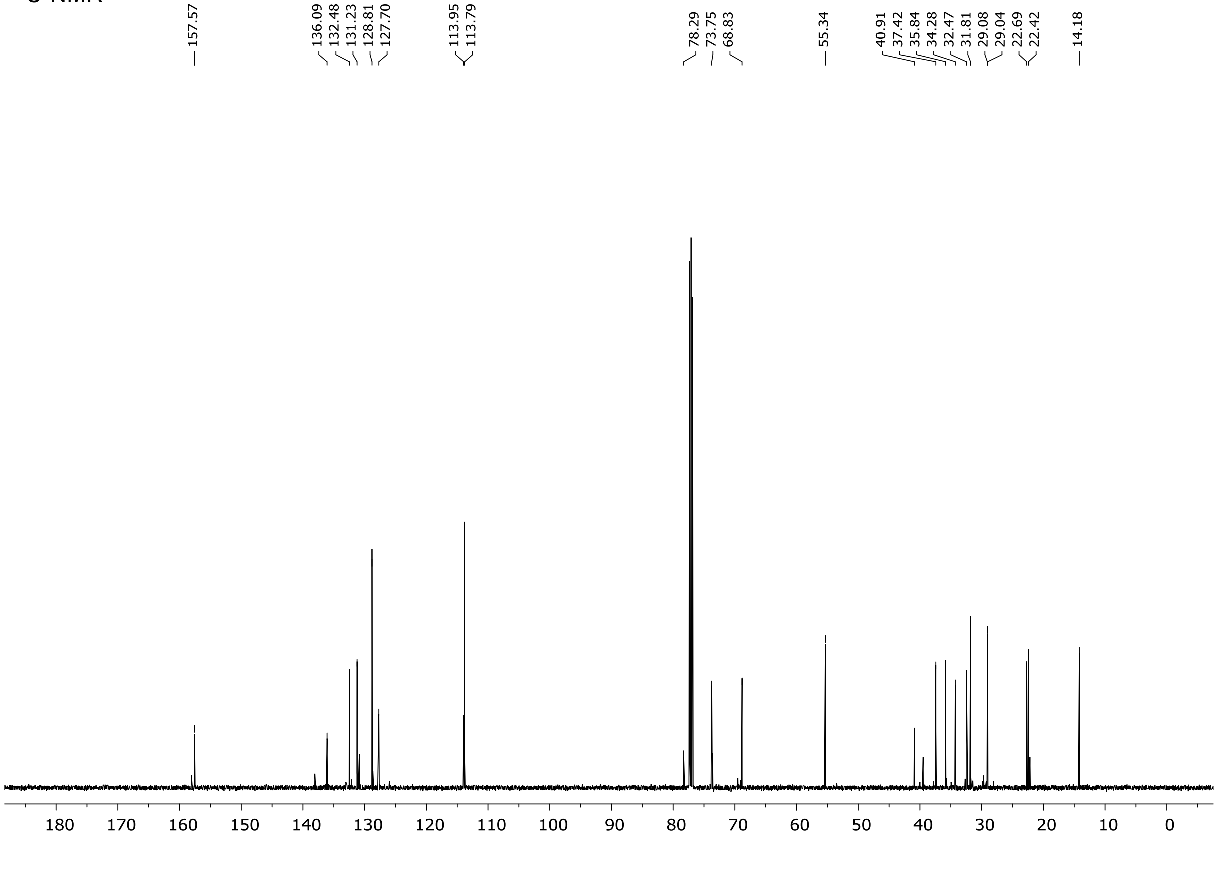

4-(4-methoxyphenyl)-2-methyl-6-((E)-oct-1-en-1-yl)tetrahydro-2H-pyran 48A

^{1}H-NMR

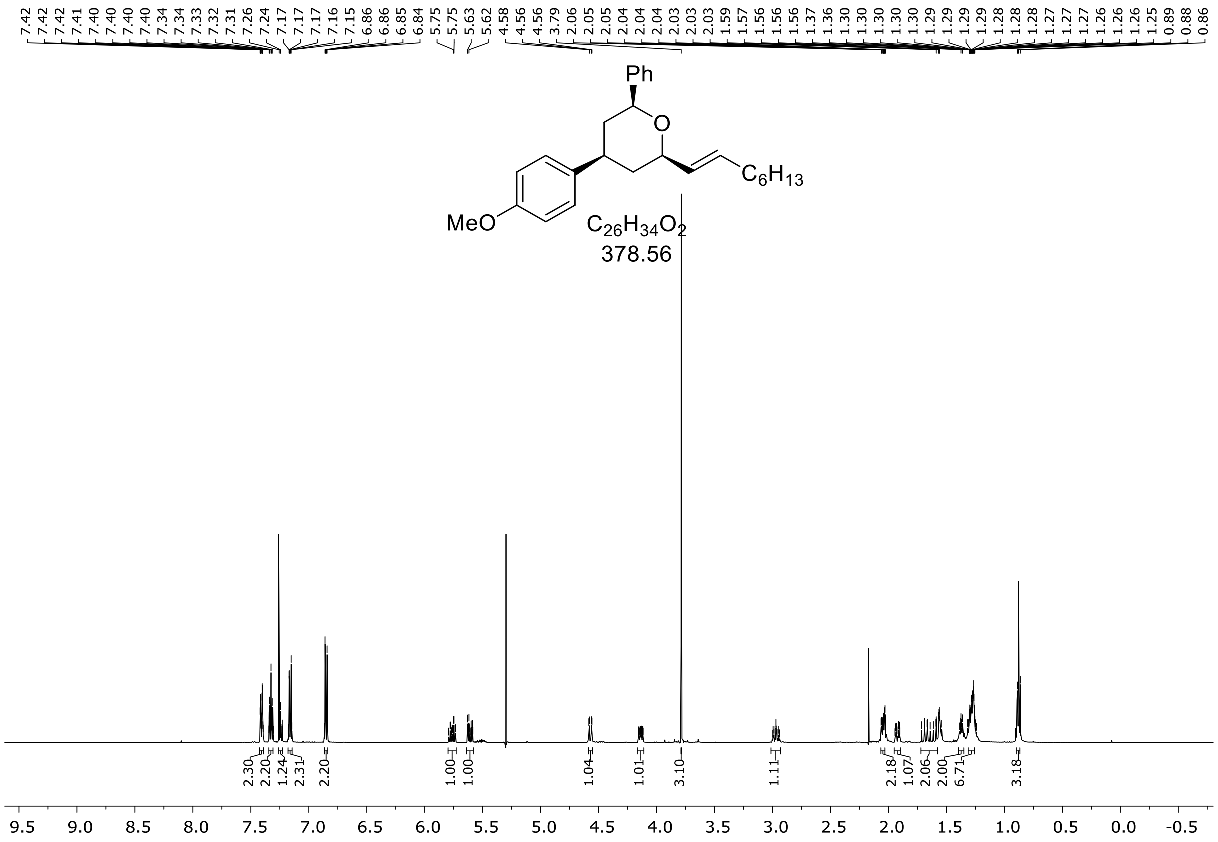

^{13}C-NMR

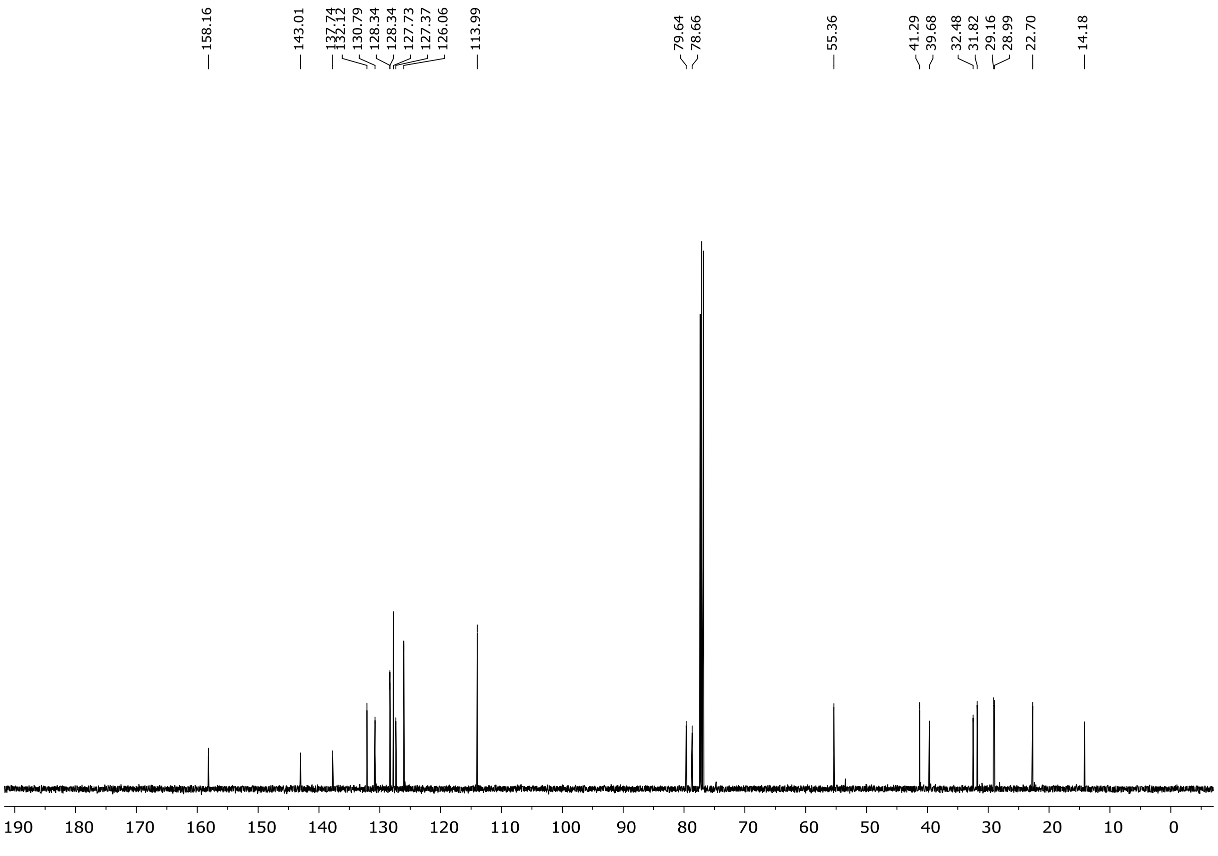

4-(4-methoxyphenyl)-2-methyl-6-((E)-oct-1-en-1-yl)tetrahydro-2H-pyran 52A

^{1}H-NMR

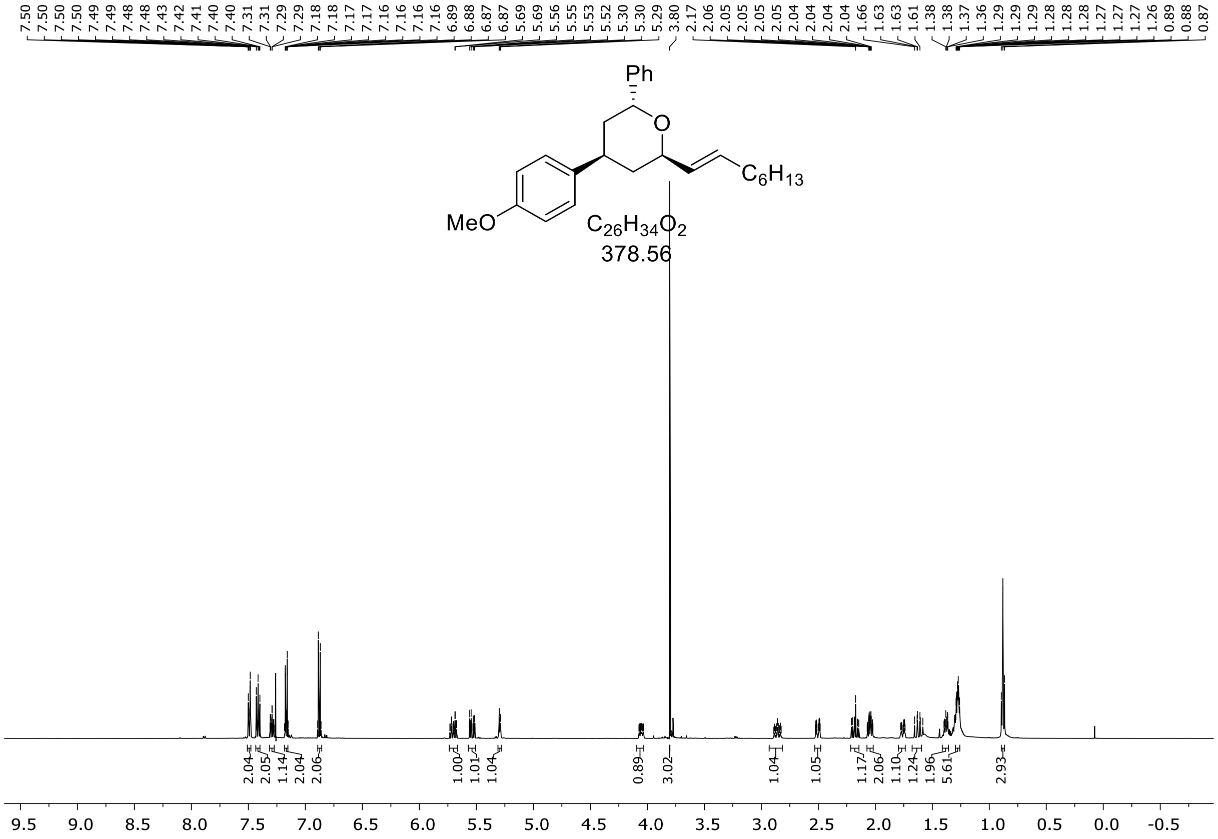

^{13}C-NMR

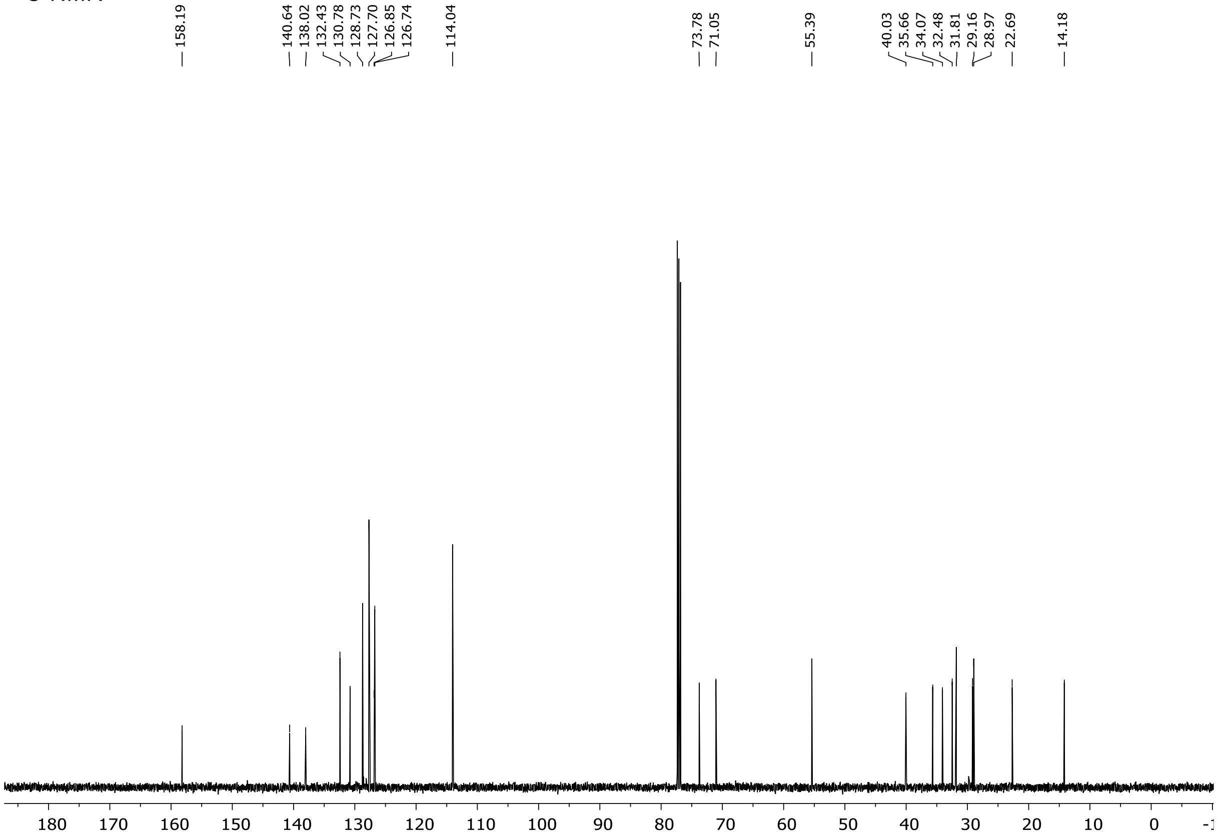

4-(4-methoxyphenyl)-2-methyl-6-((E)-oct-1-en-1-yl)tetrahydro-2H-pyran 52B

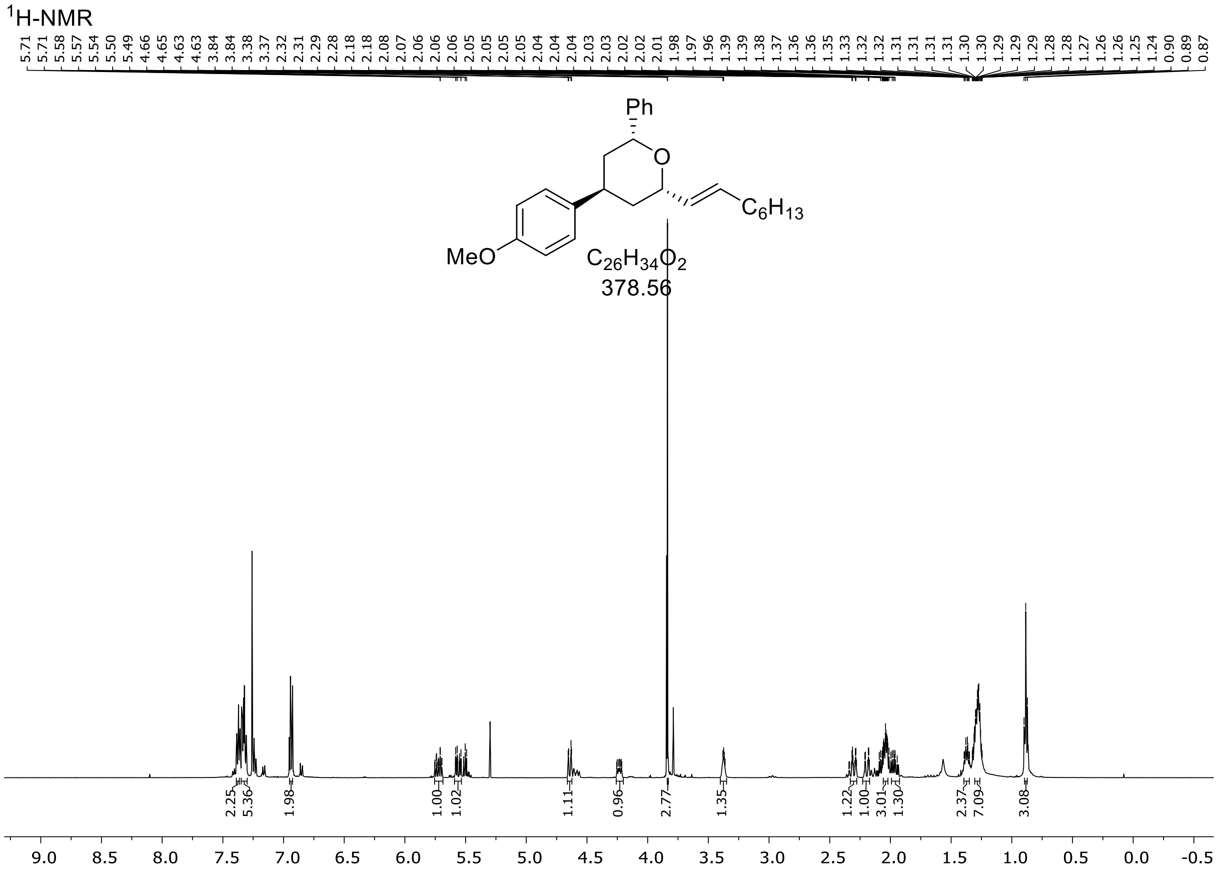

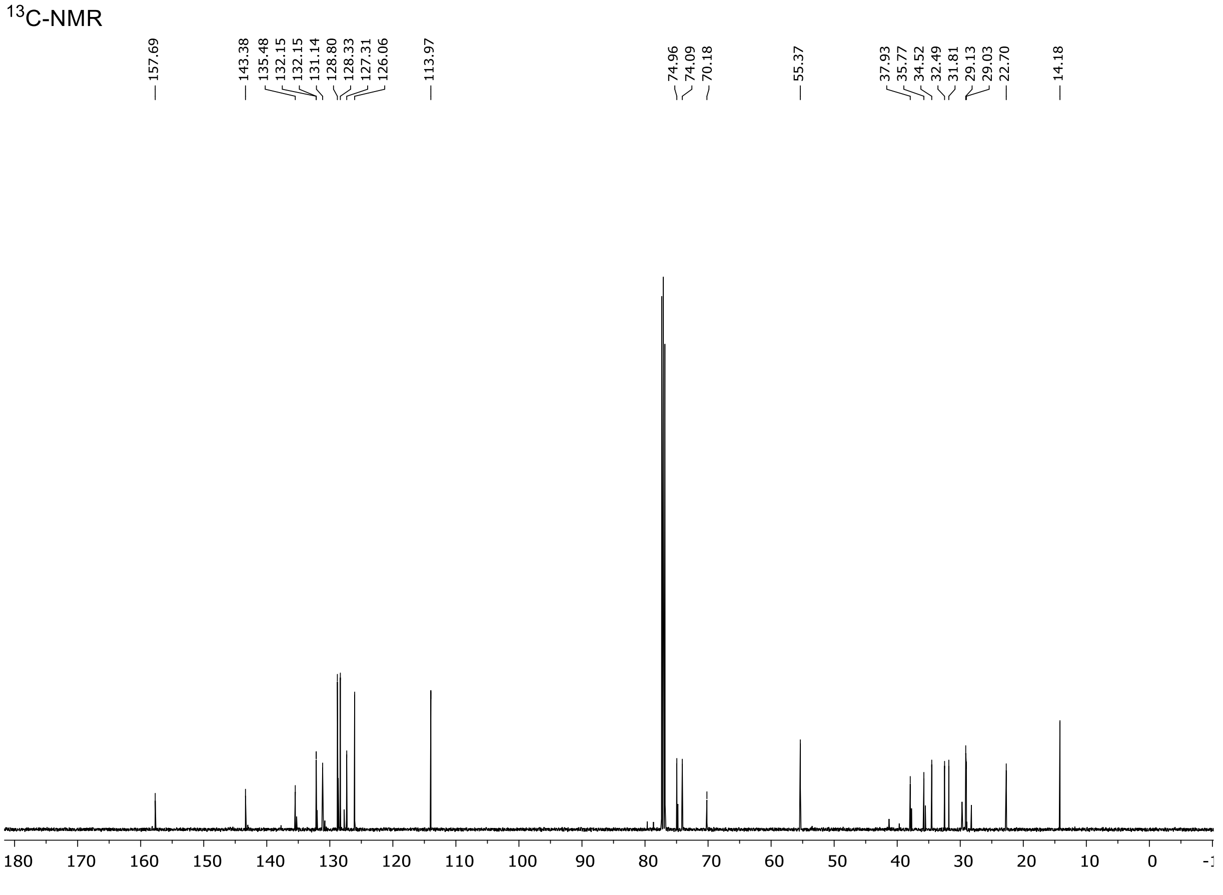

ethyl hepta-3,4-dienoate 131

^{1}H-NMR

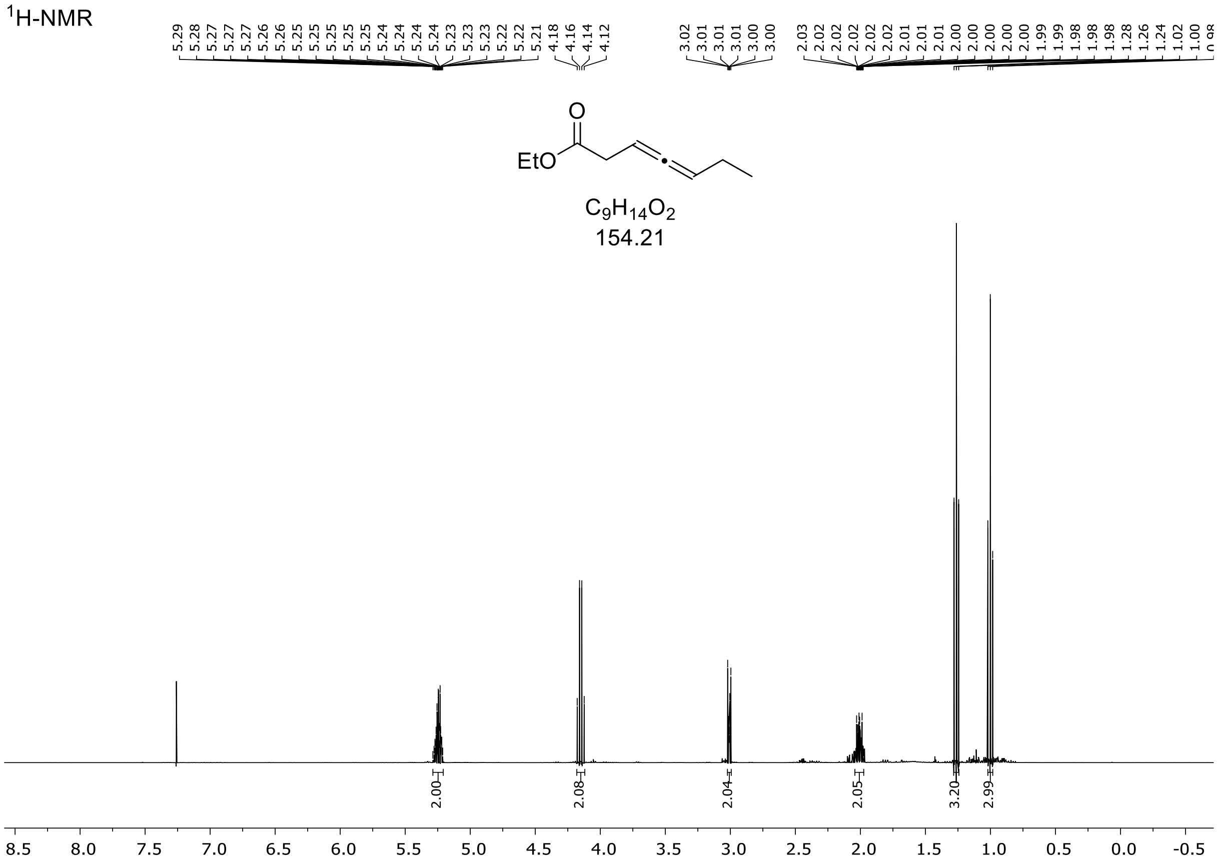

^{13}C-NMR

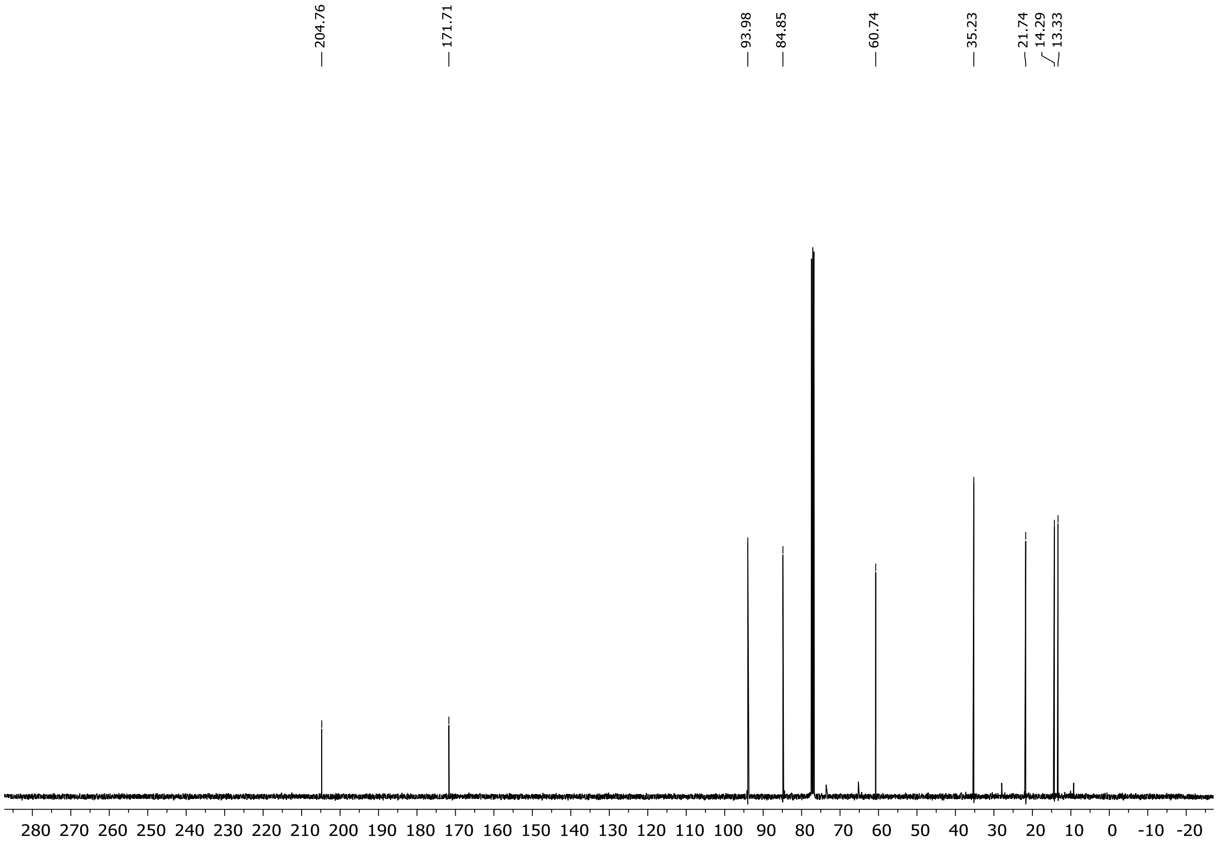

ethyl (E)-nona-2,5,6-trienoate 132

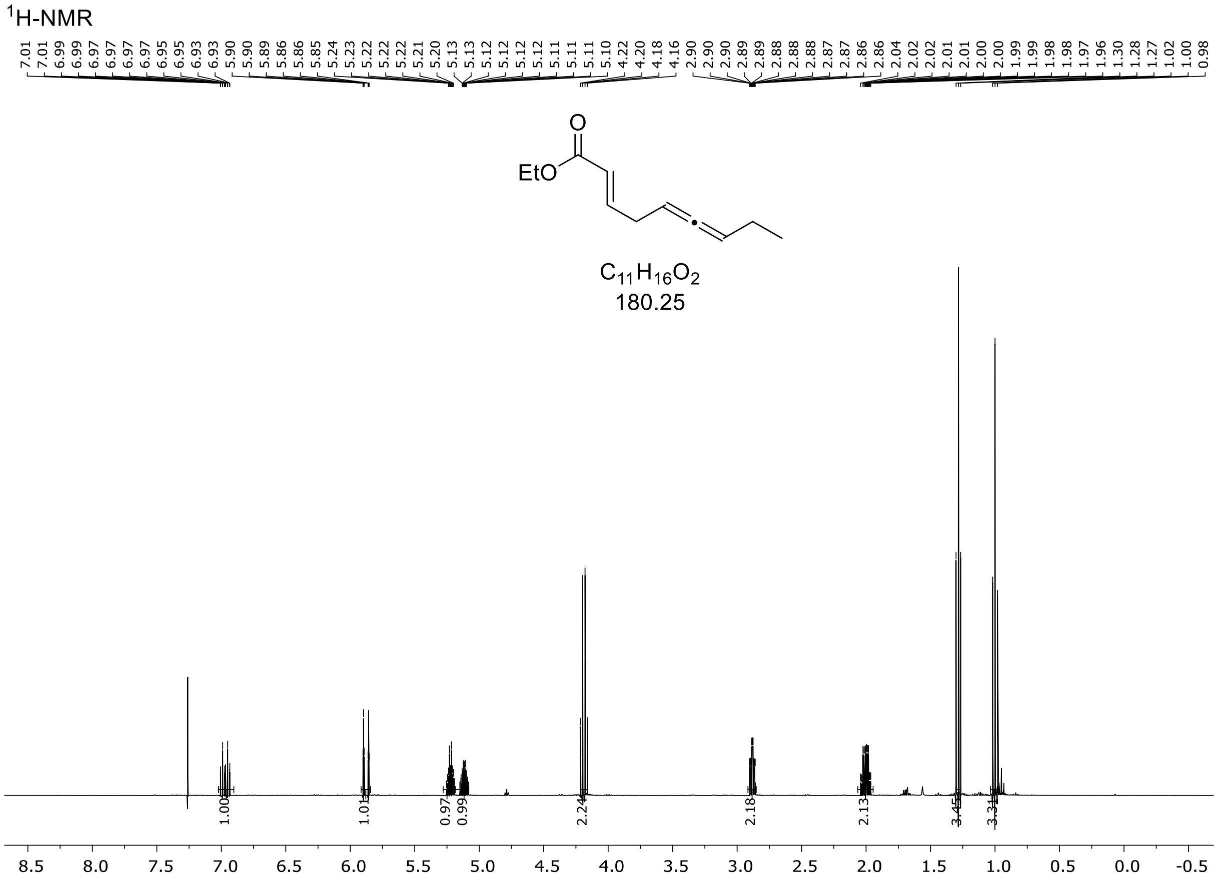

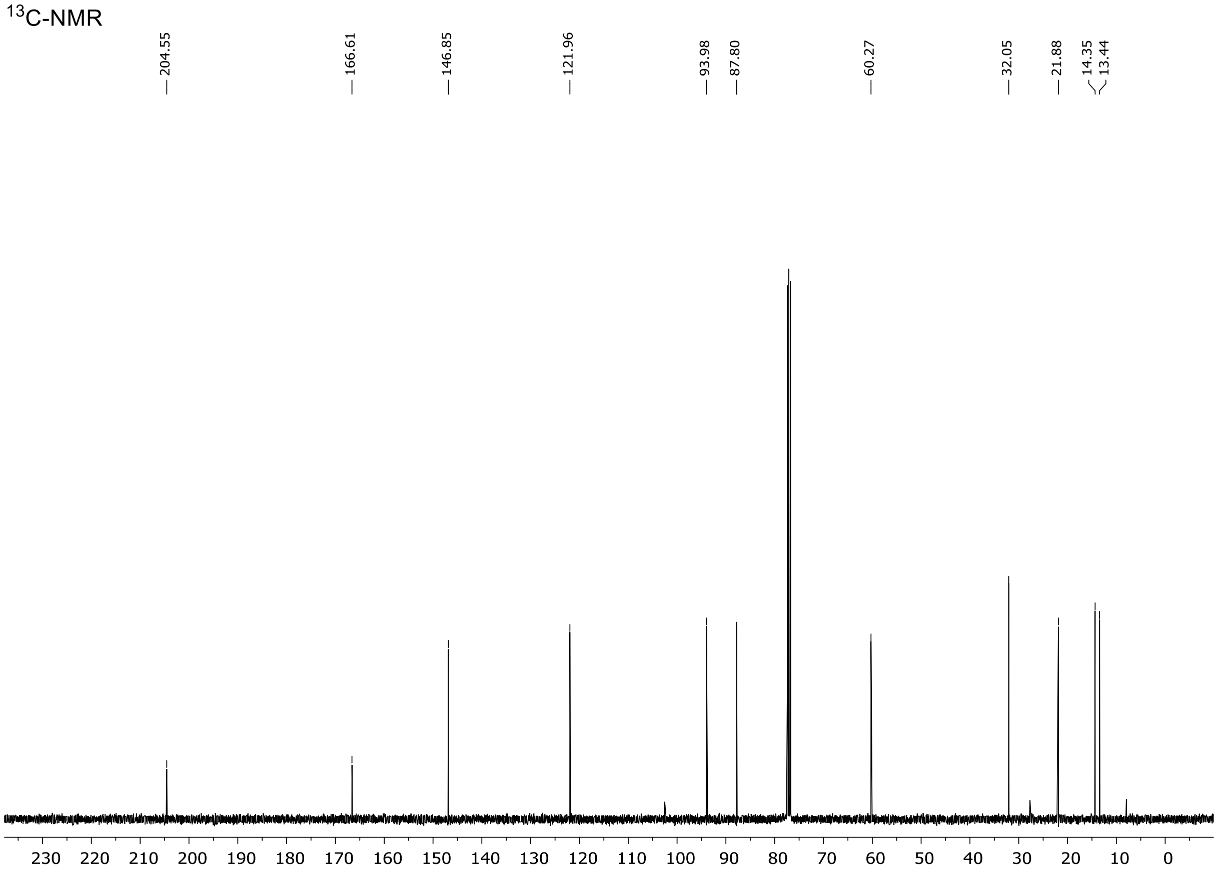

ethyl (3S)-3-methylnona-5,6-dienoate 133

^{1}H-NMR

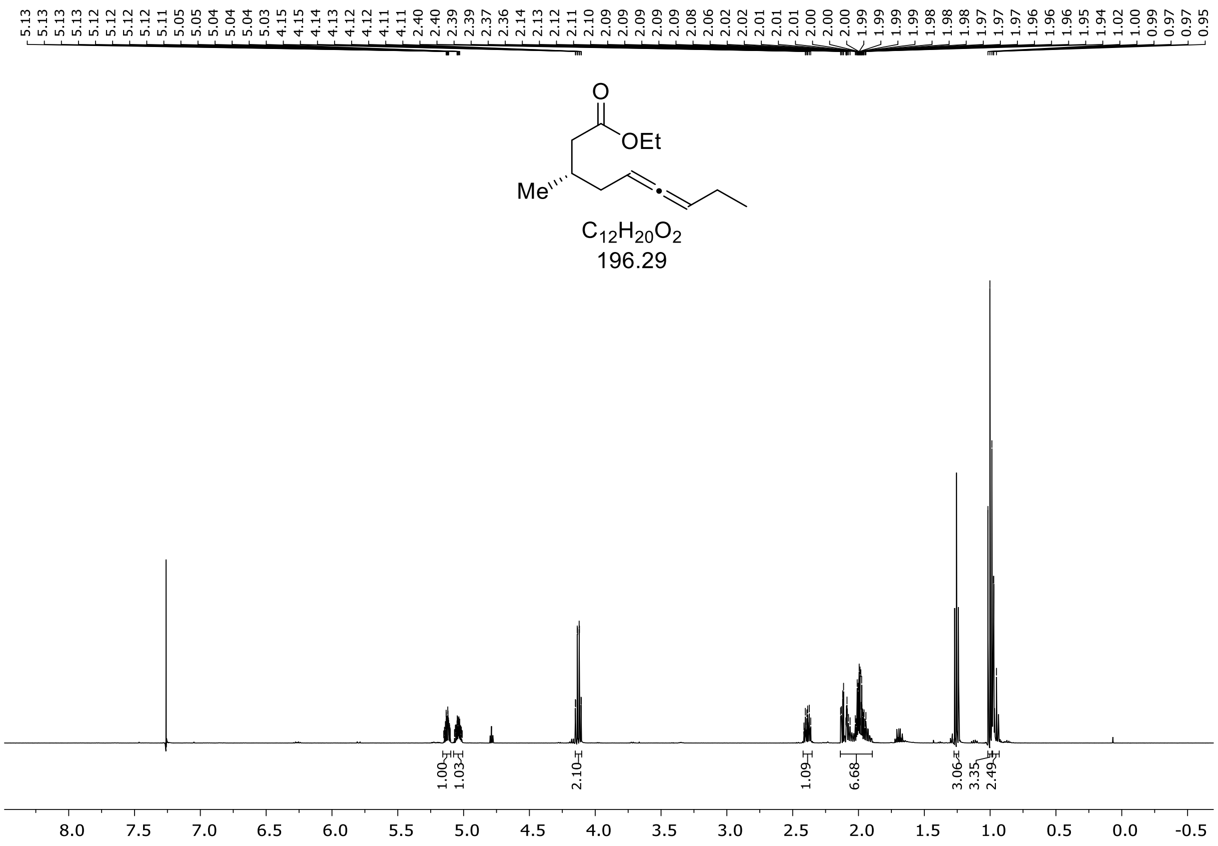

^{13}C-NMR

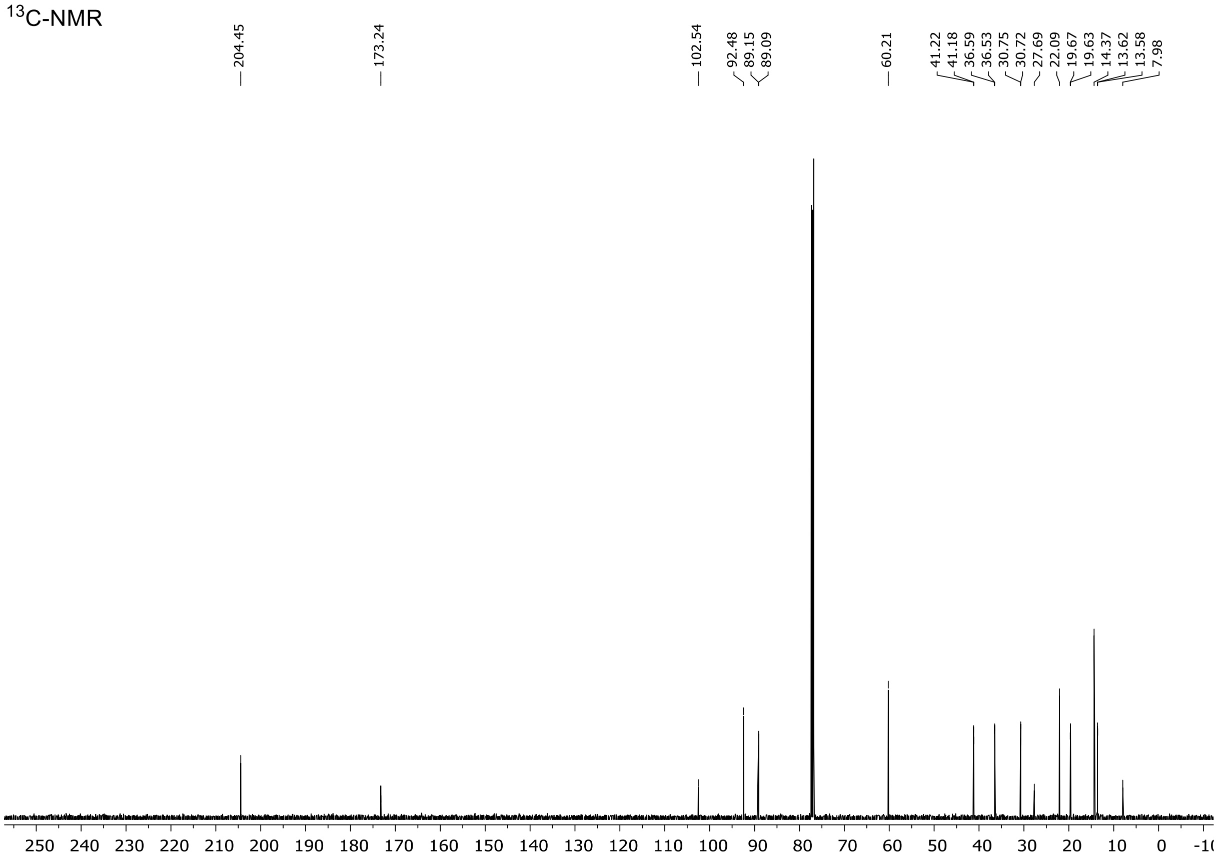

(S)-3-methylhepta-5,6-dien-1-ol 54

^{1}H-NMR

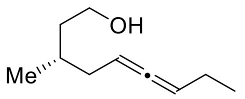

$C_{10}H_{18}O$
154.25

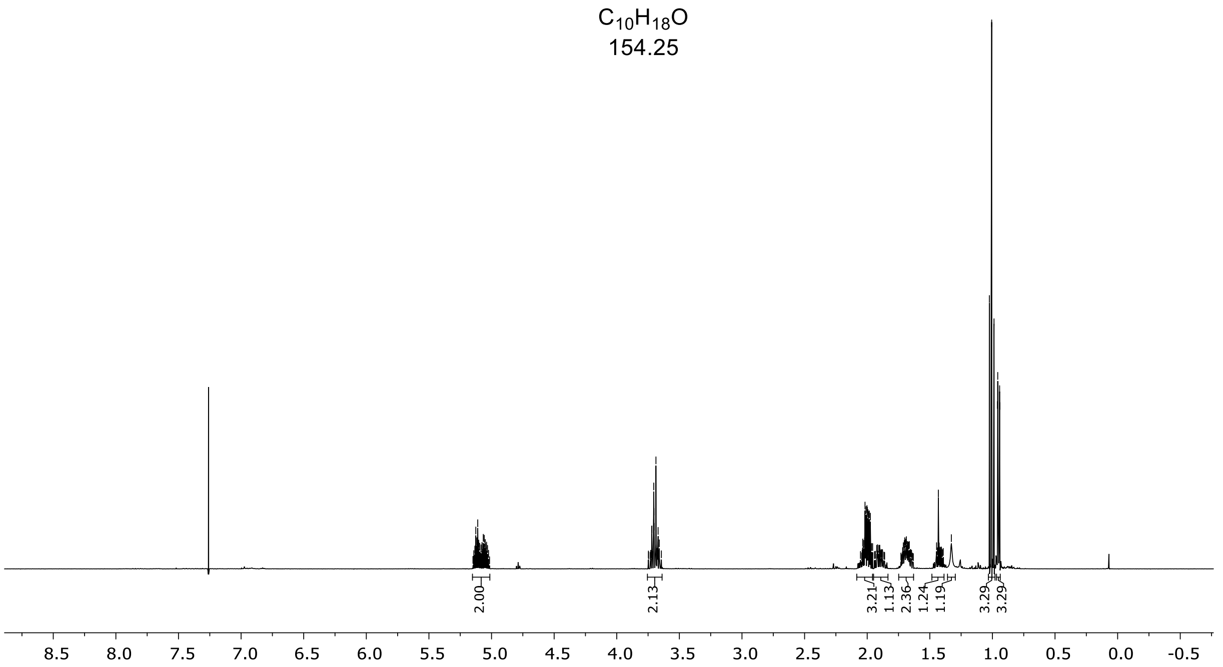

^{13}C-NMR

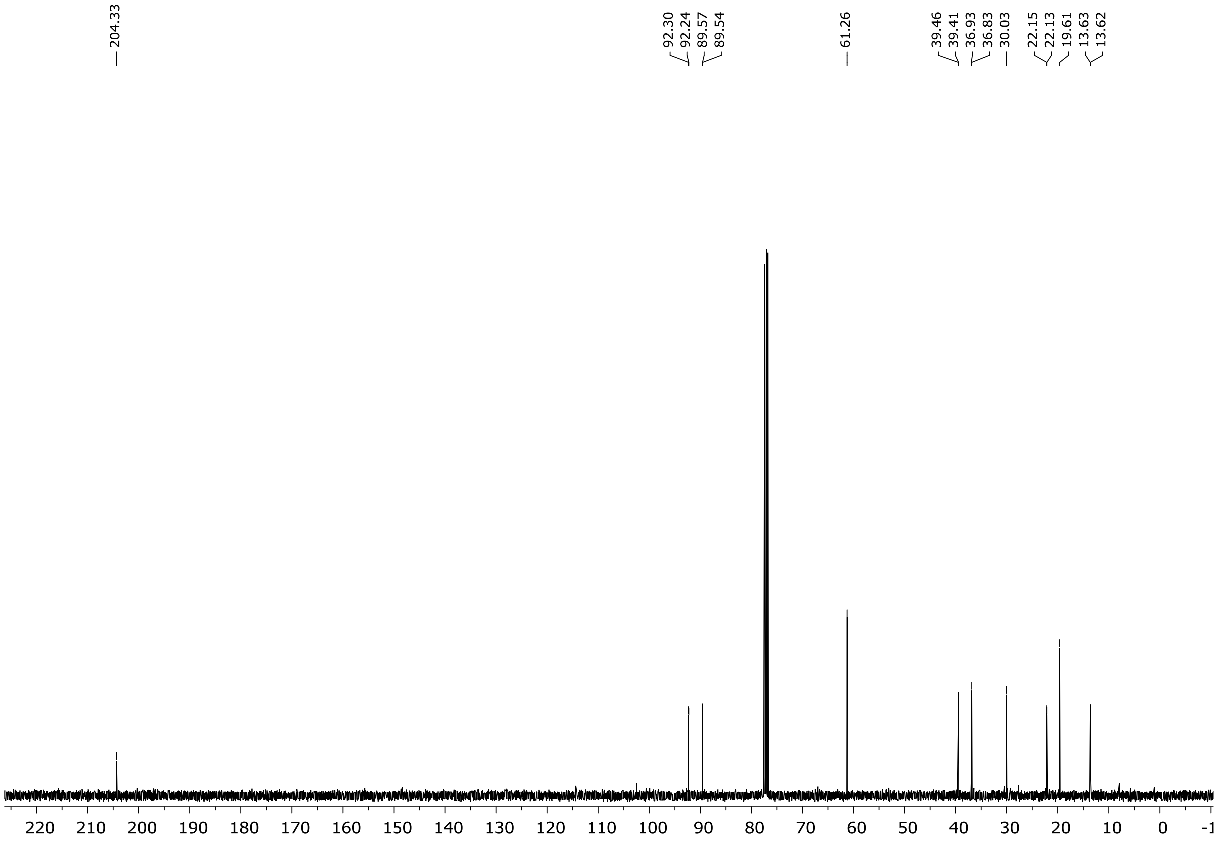

(2*S*,4*R*)-2-((E)-but-1-en-1-yl)-4-methyltetrahydro-2H-pyran 55ee

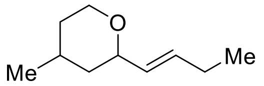

$C_{10}H_{18}O$
154.25

^{1}H-NMR

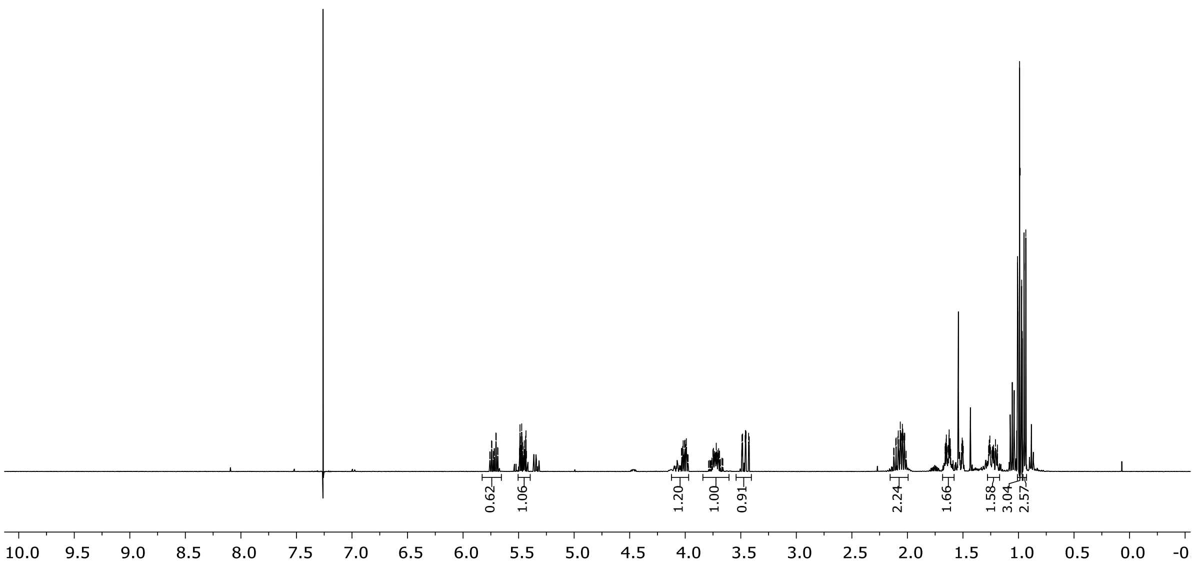

^{13}C-NMR

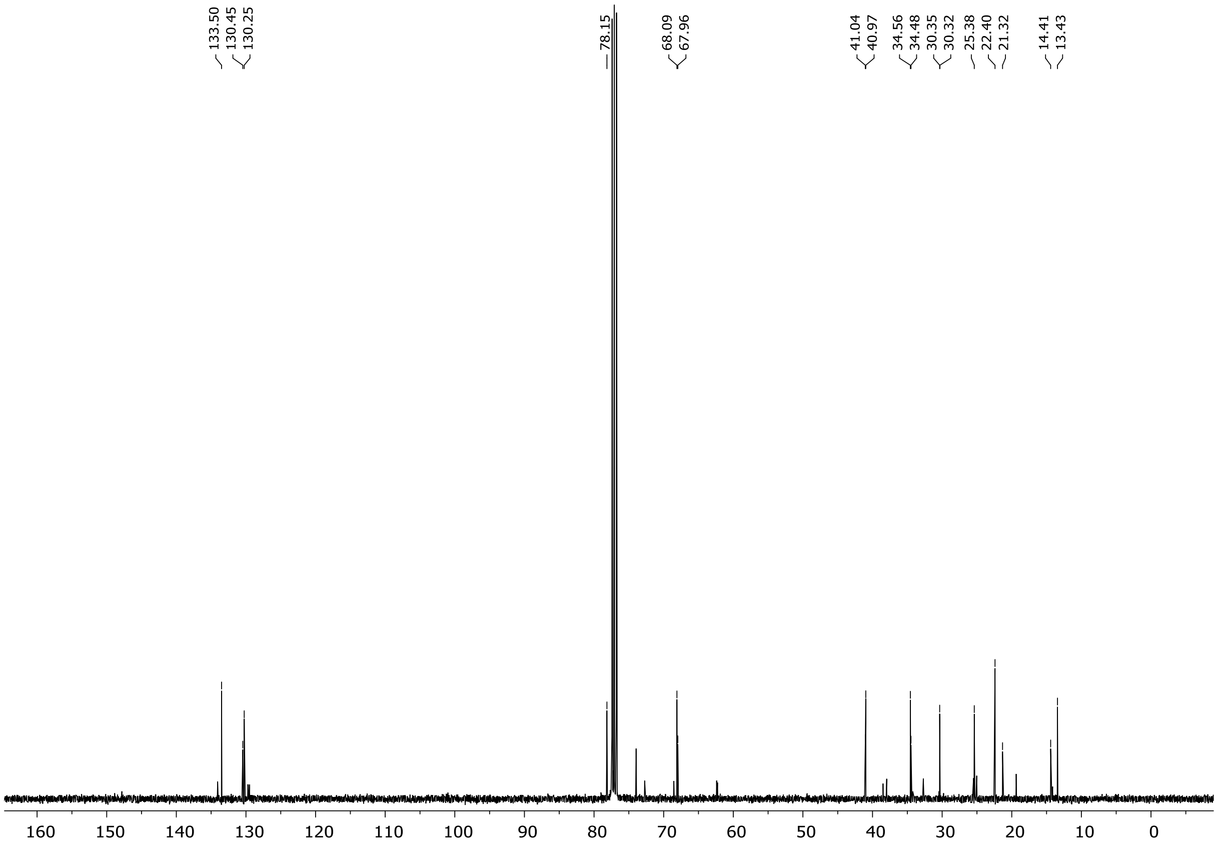

syn-(4-cyclopropyl-2-octyltetrahydro-2H-pyran 56

^{1}H-NMR

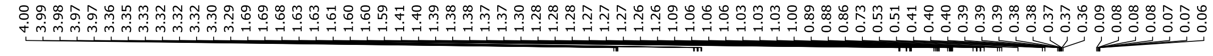

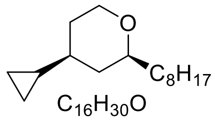

$C_{16}H_{30}O$
238.42

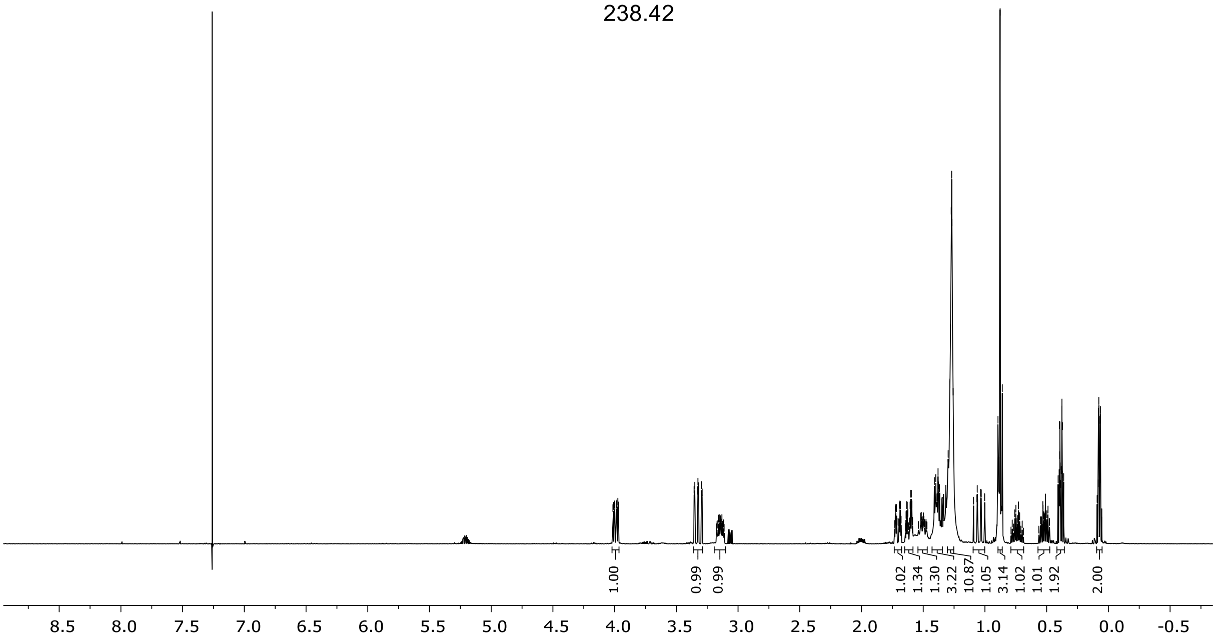

^{13}C-NMR

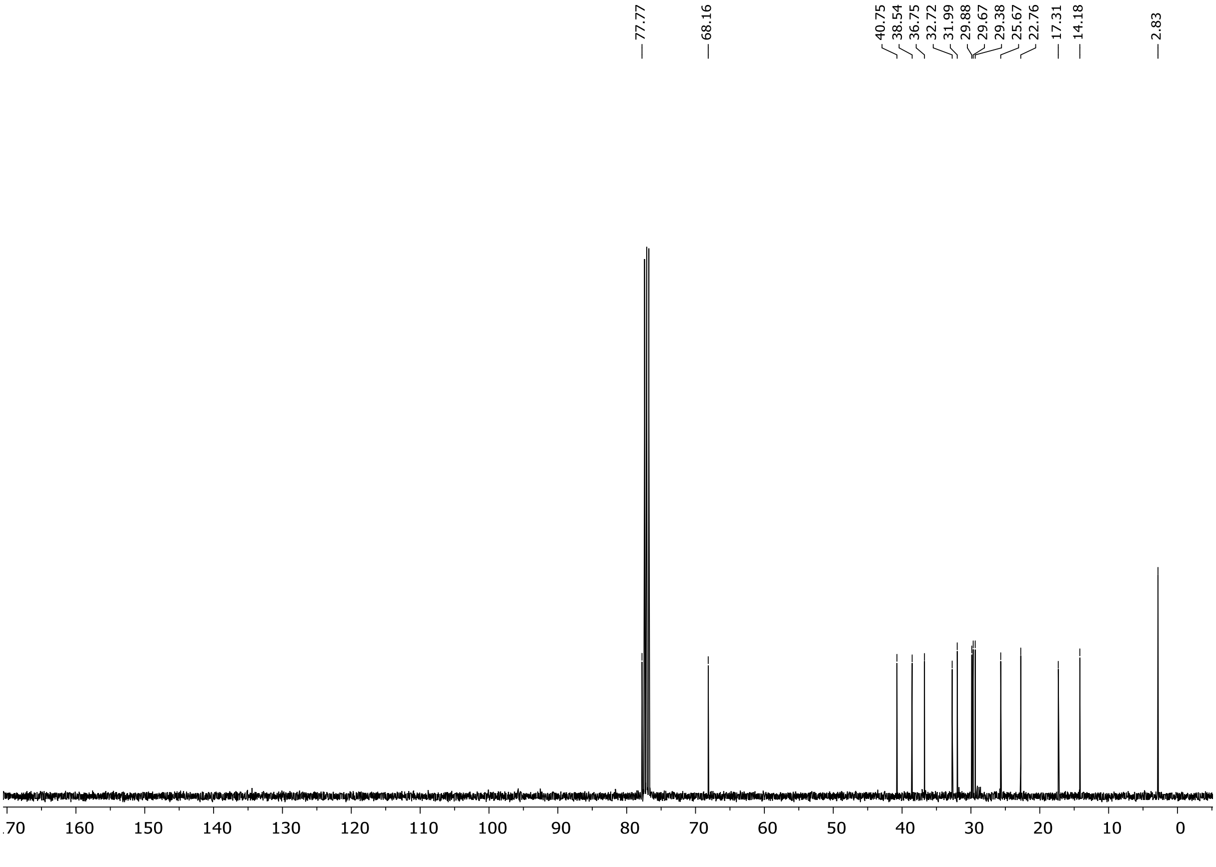

syn 2-octyl-4-(4-phenylbutyl)tetrahydro-2H-pyran 57

^{1}H-NMR

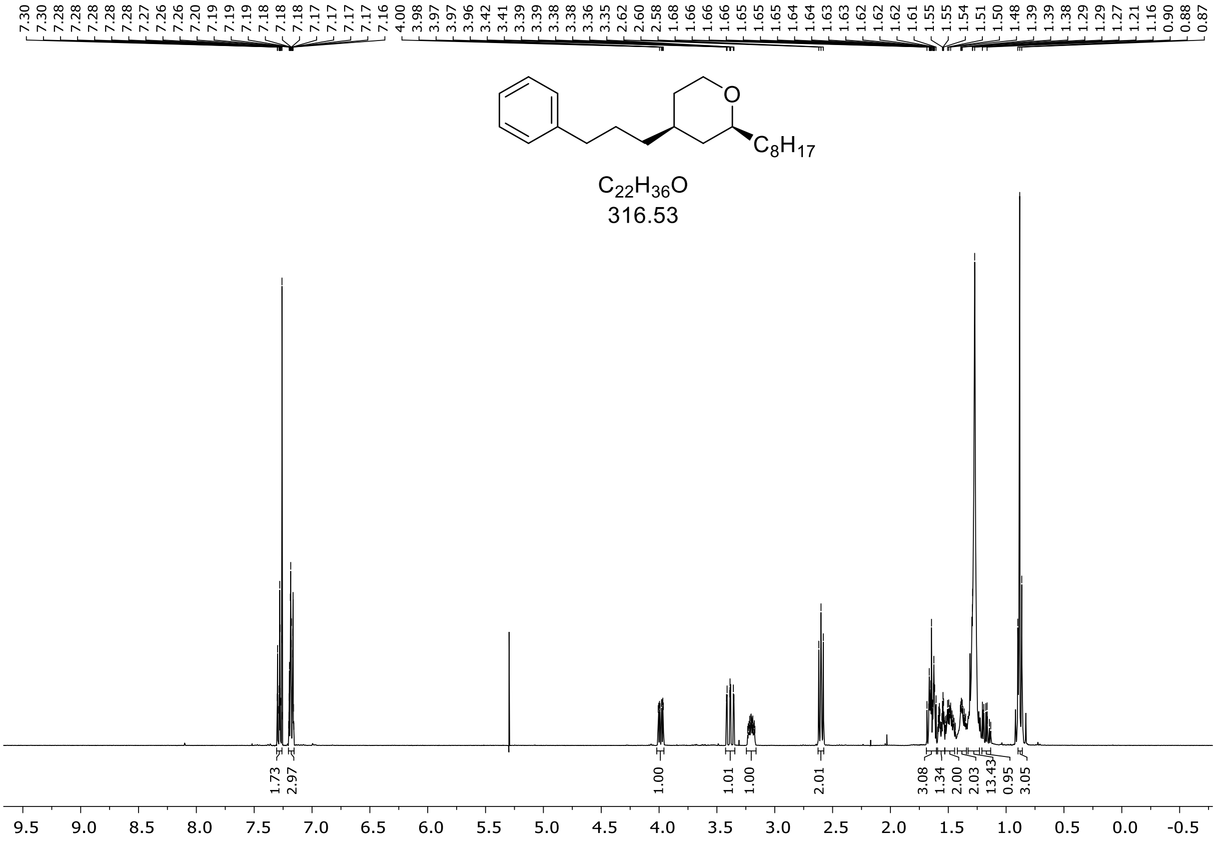

^{13}C-NMR

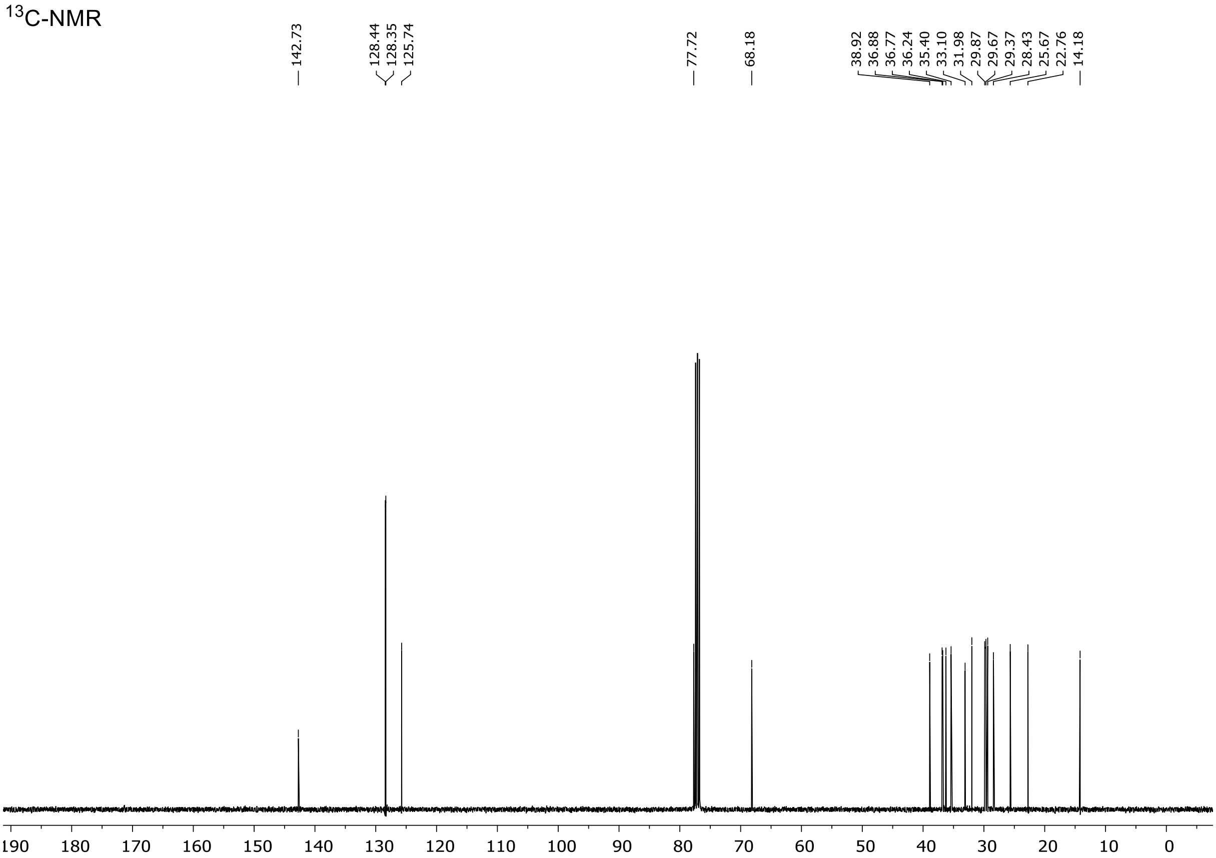

syn-4-([1,1'-biphenyl]-4-yl)-2-octyltetrahydro-2H-pyran 58

^{1}H-NMR

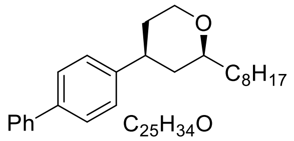

$C_{25}H_{34}O$
350.55

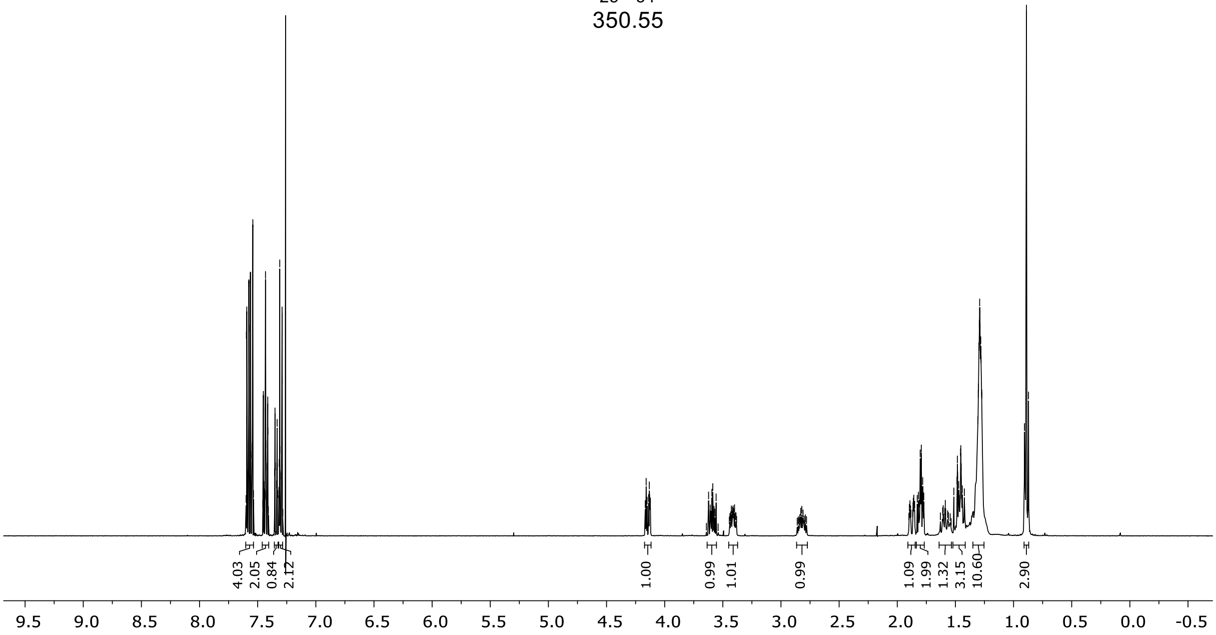

^{13}C-NMR

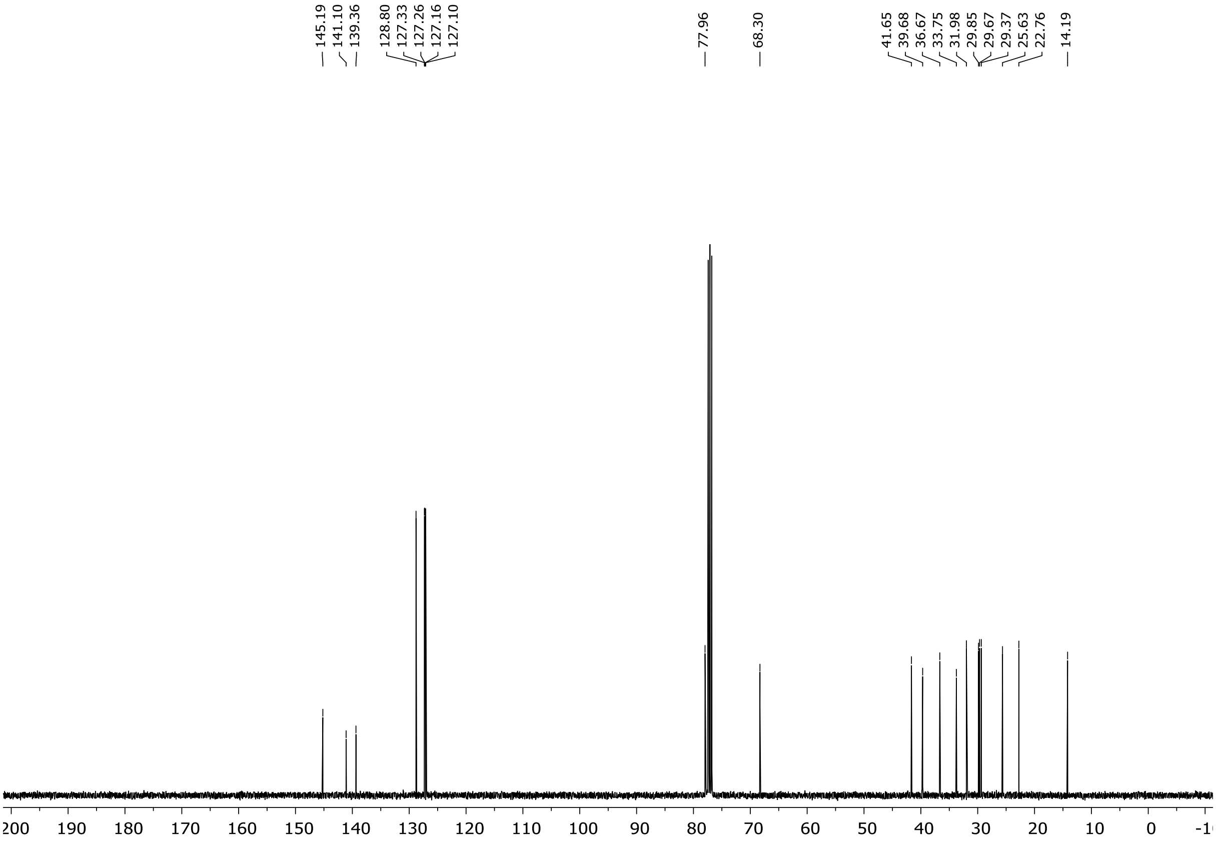

syn-4-(4-methoxyphenyl)-2-(4-phenylbutyl)tetrahydro-2H-pyran 59

^{1}H-NMR

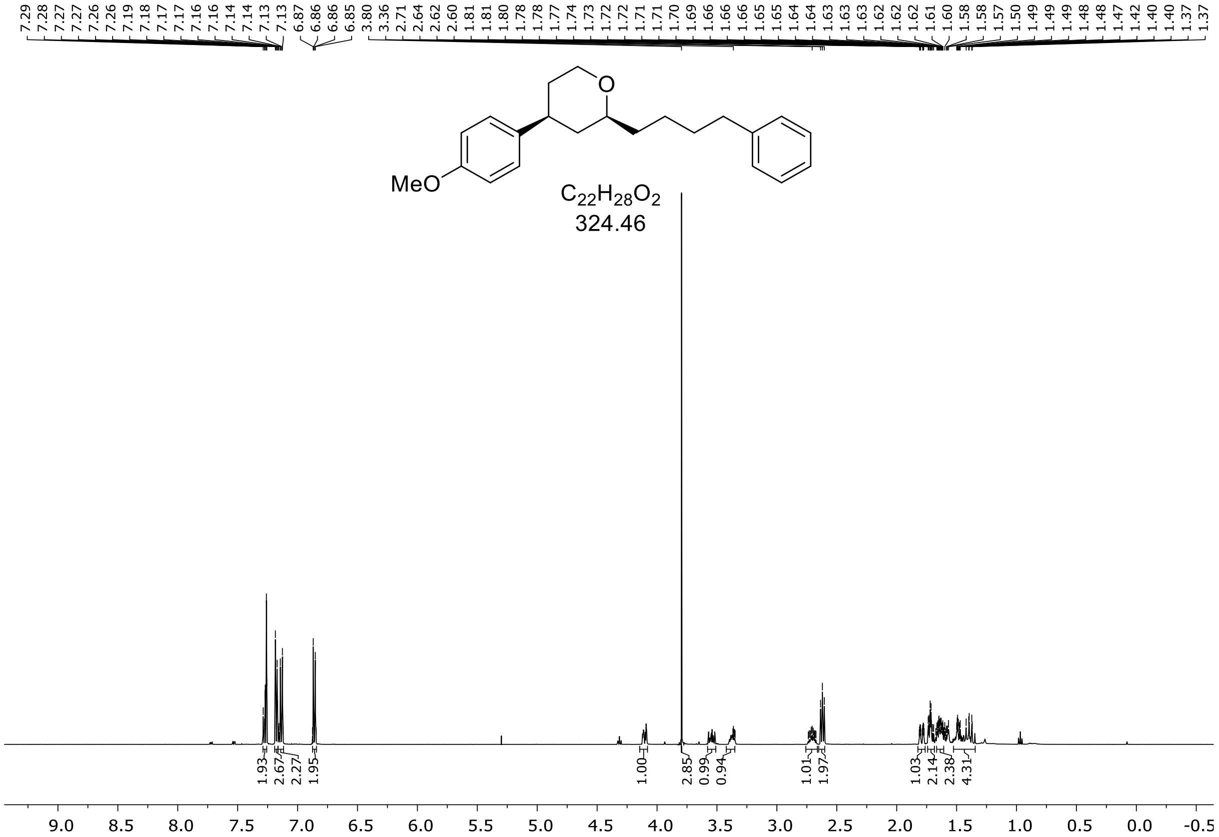

^{13}C-NMR

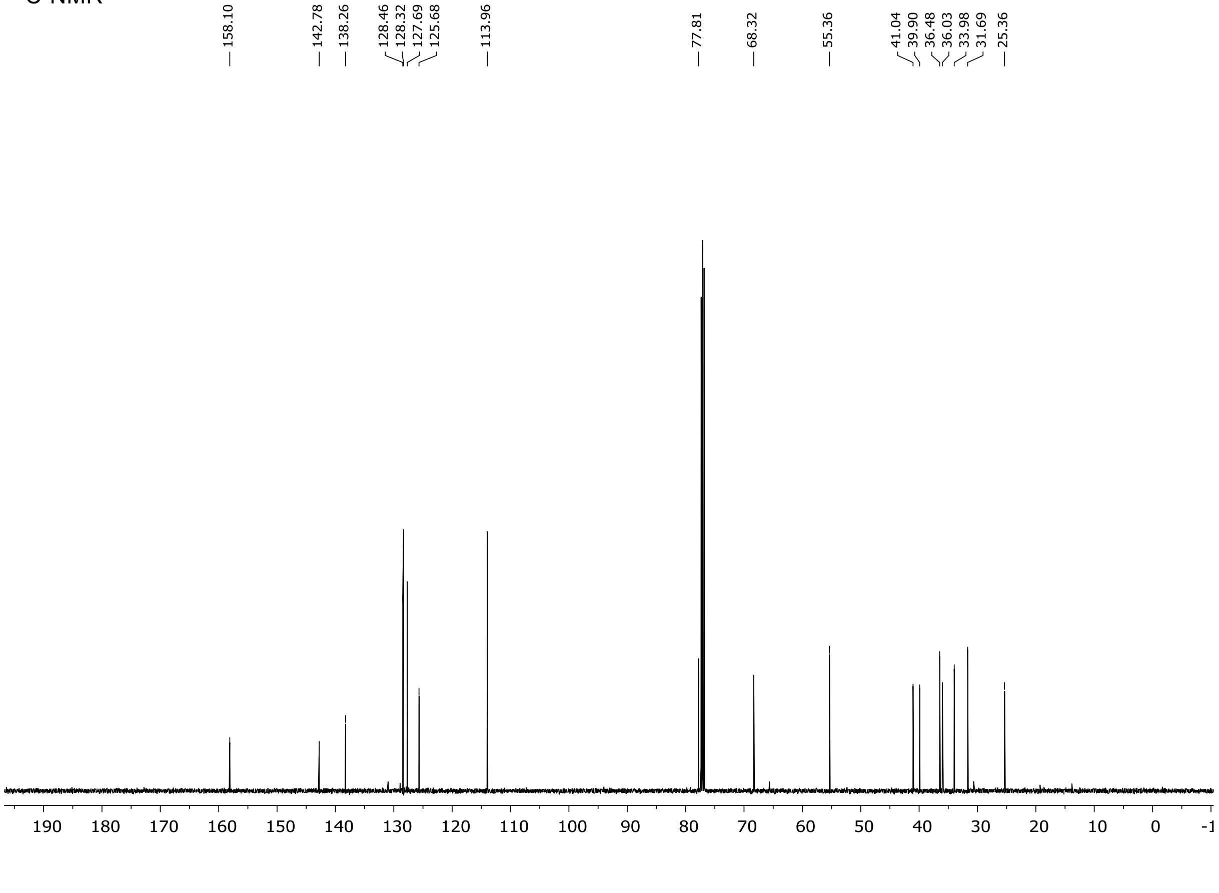

5-(4-methoxyphenyl)-5-oxopentanal 61

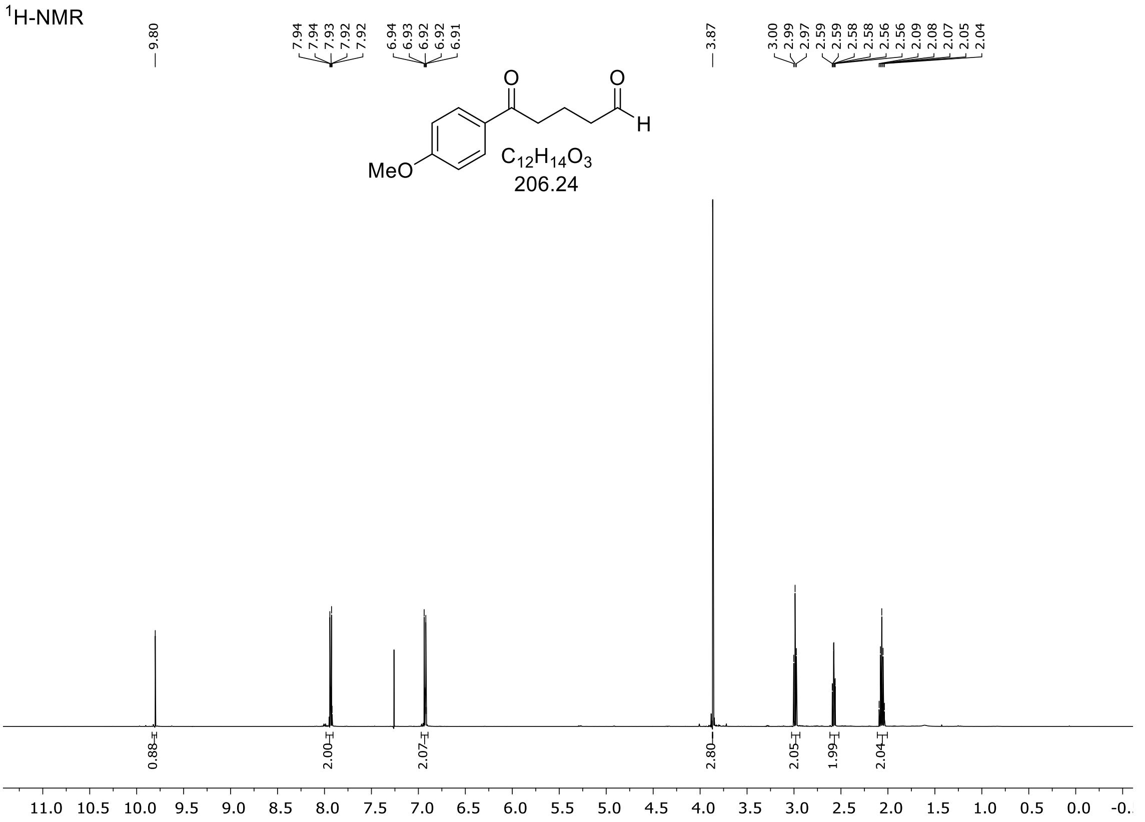

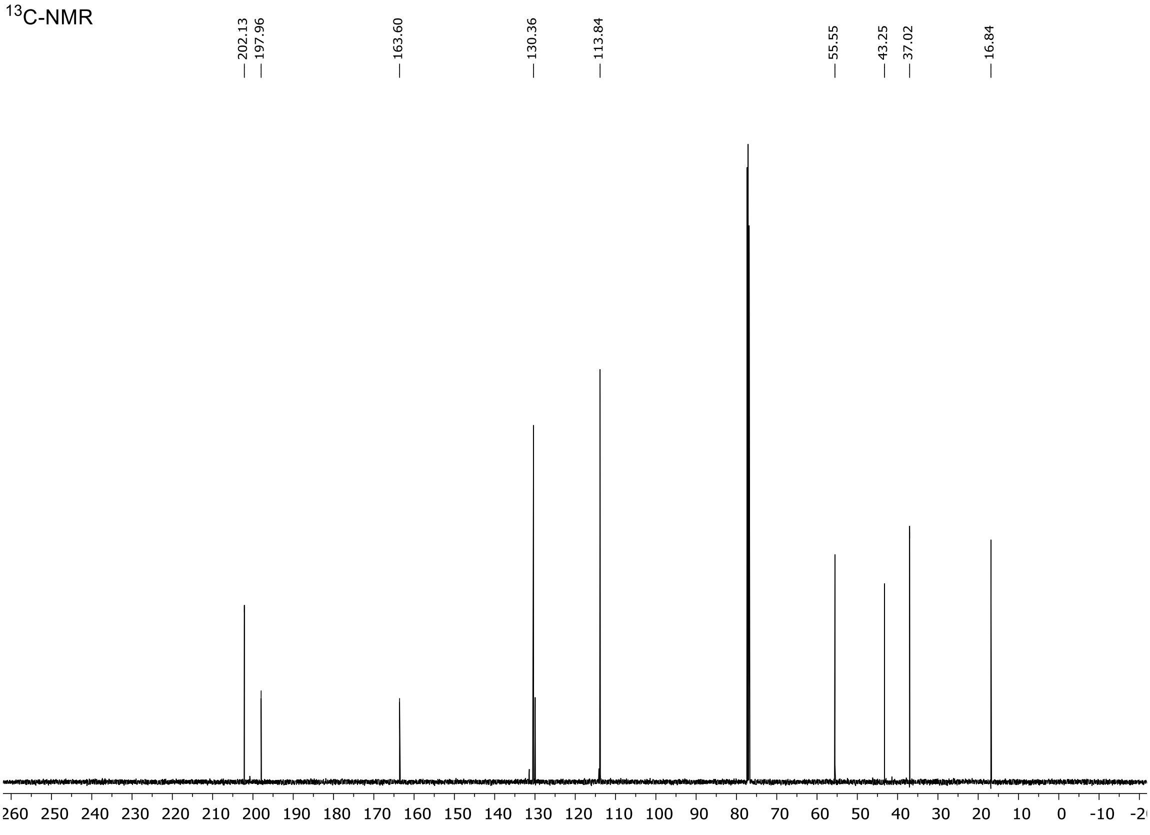

7-(4-methoxyphenyl)-7-oxohept-1-yn-3-yl acetate

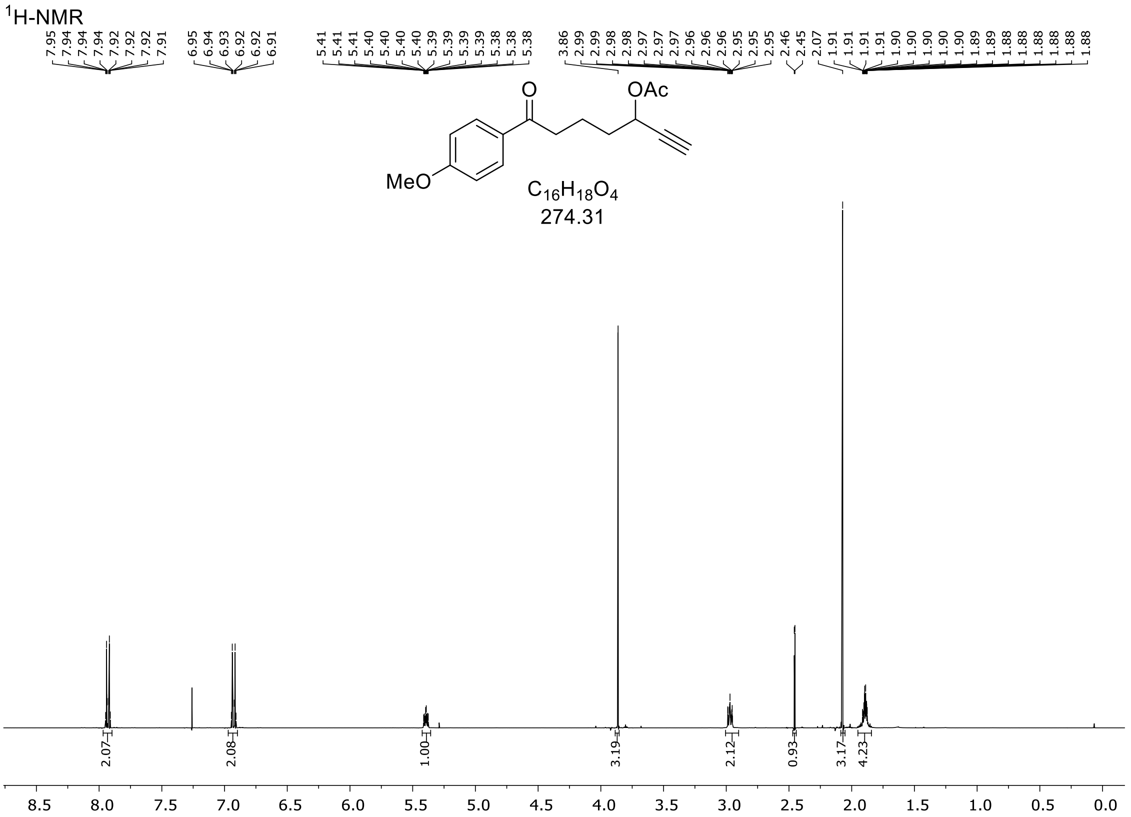

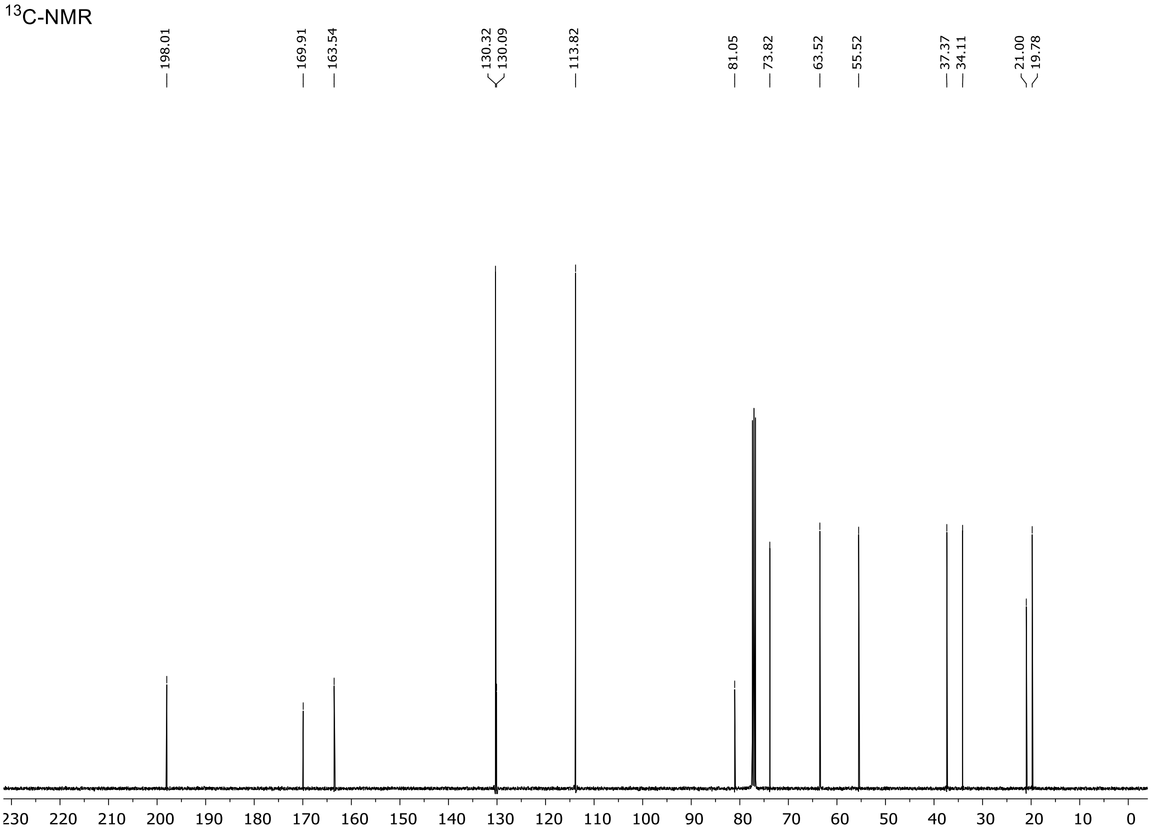

7-(4-(benzyloxy)phenyl)-1-(4-methoxyphenyl)hepta-5,6-dien-1-one 62

^{1}H-NMR

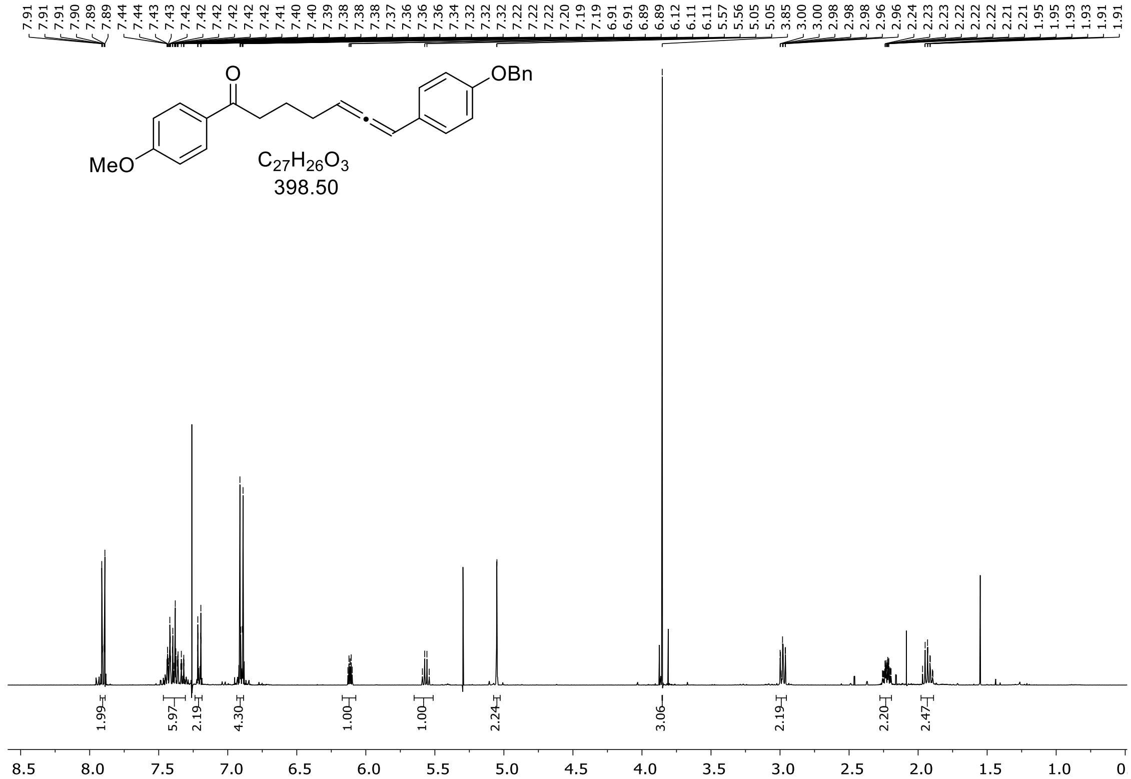

^{13}C-NMR

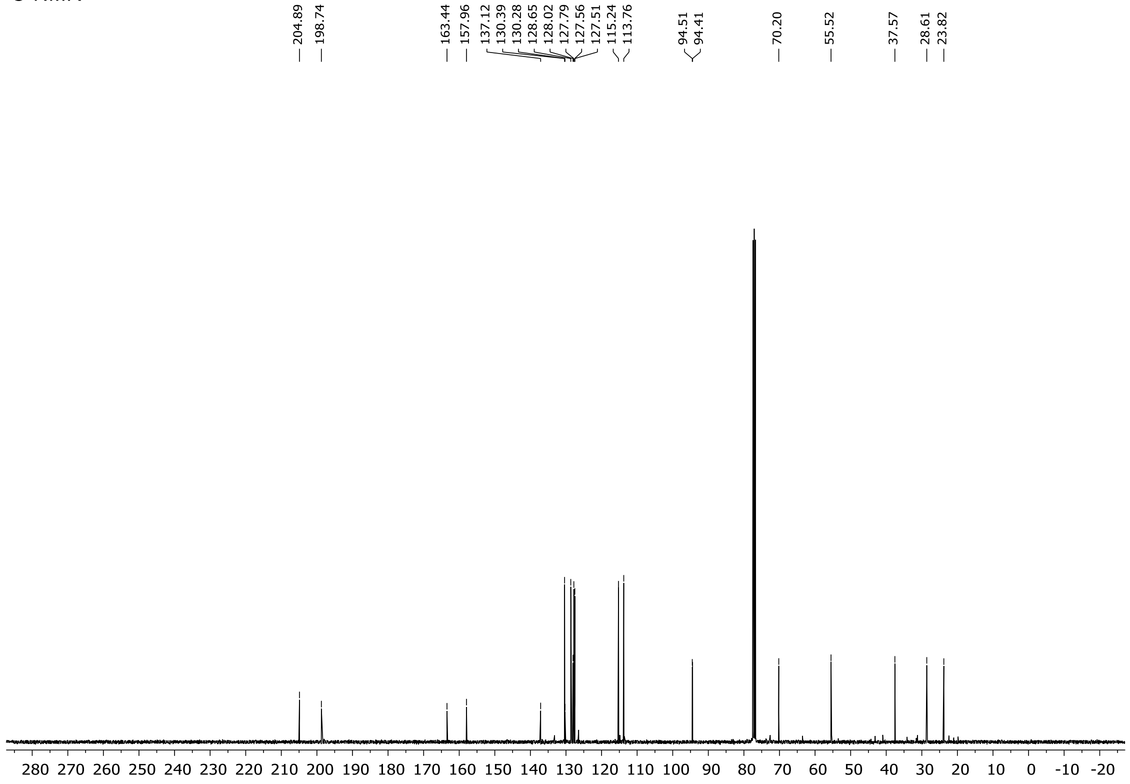

(1S)-7-(4-(benzyloxy)phenyl)-1-(4-methoxyphenyl)hepta-5,6-dien-1-ol 63

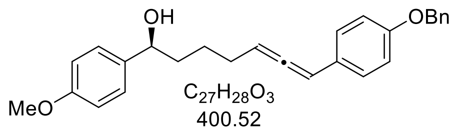

^{1}H-NMR

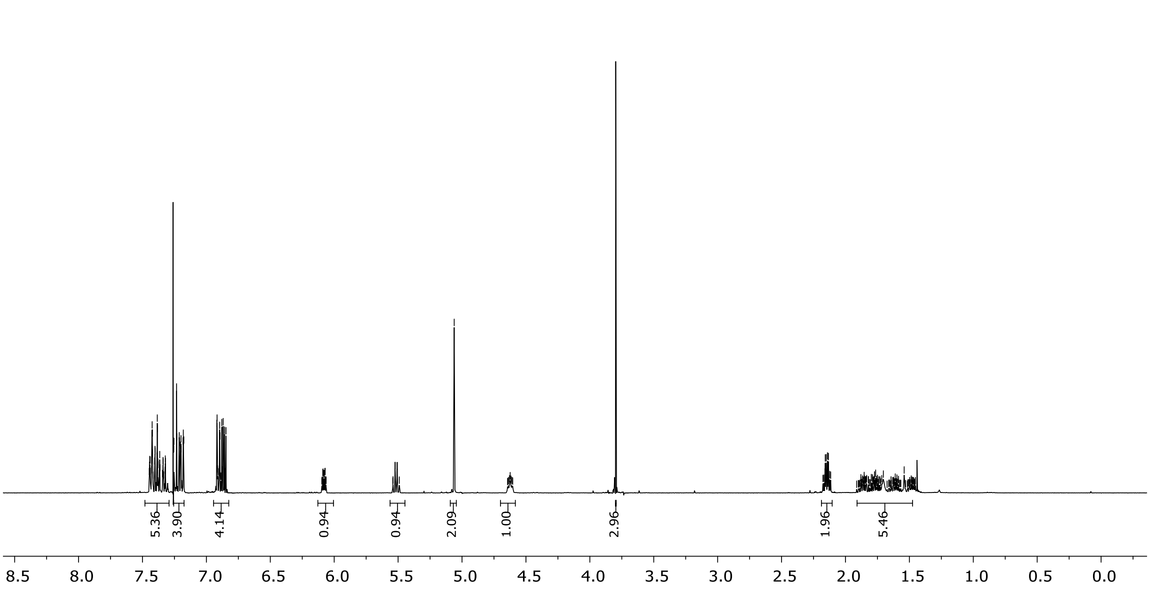

^{13}C-NMR

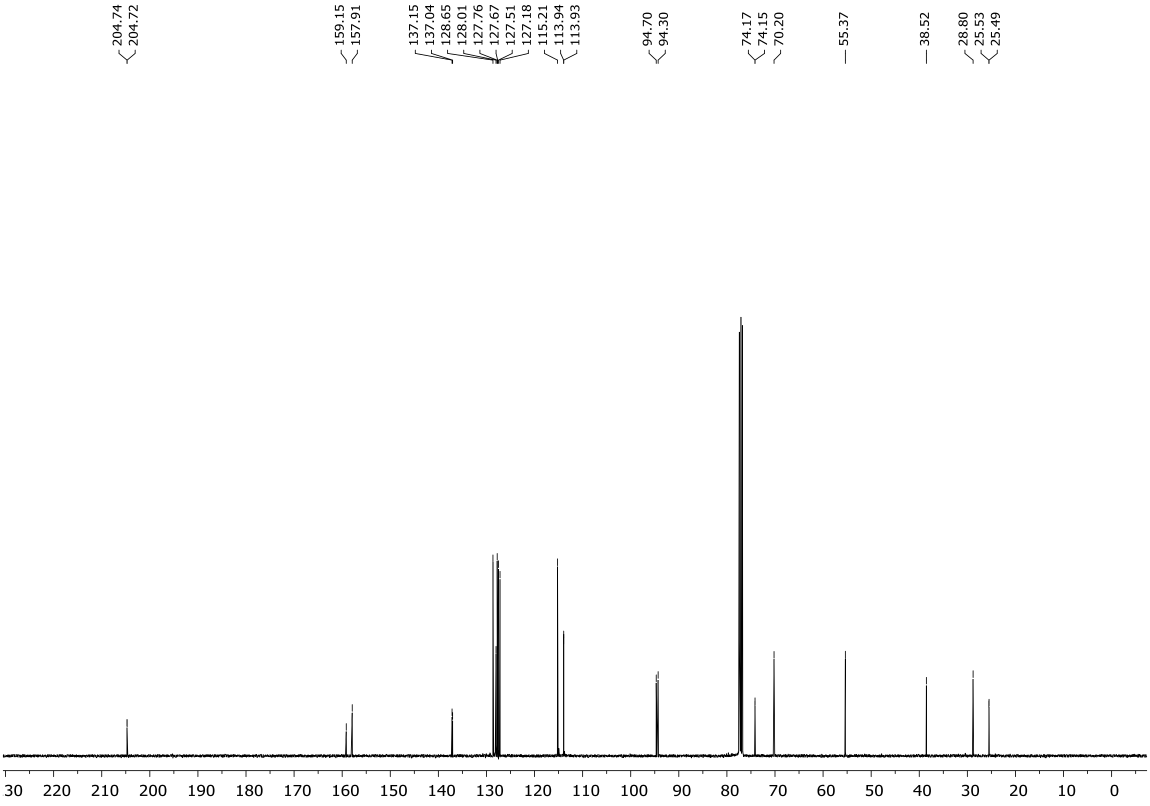

(-)-Centrolobine 64

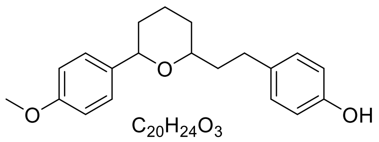

$C_{20}H_{24}O_3$
312.41

^{1}H-NMR

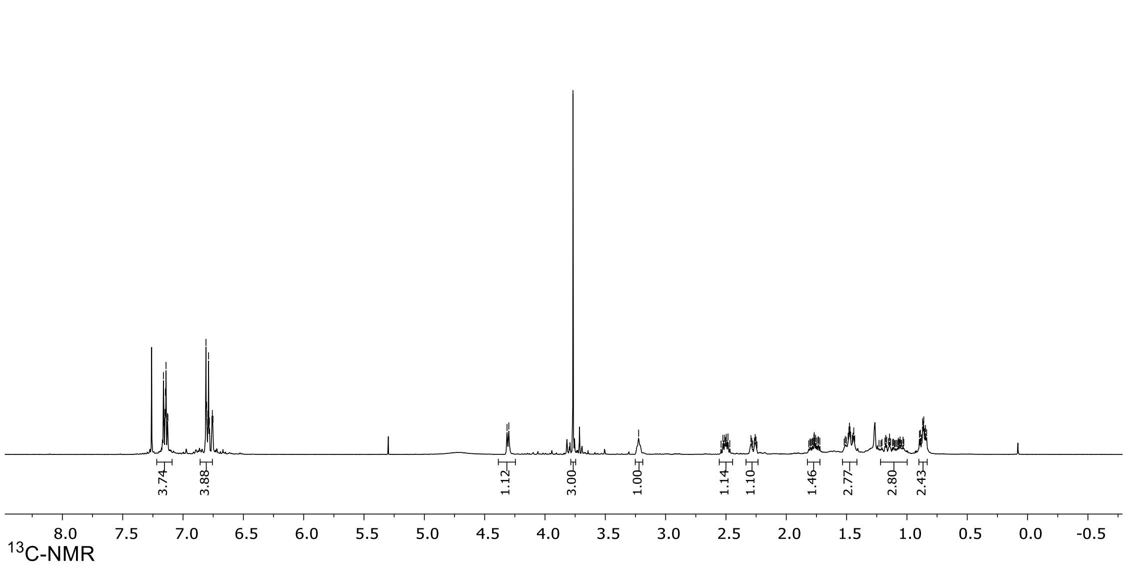

^{13}C-NMR

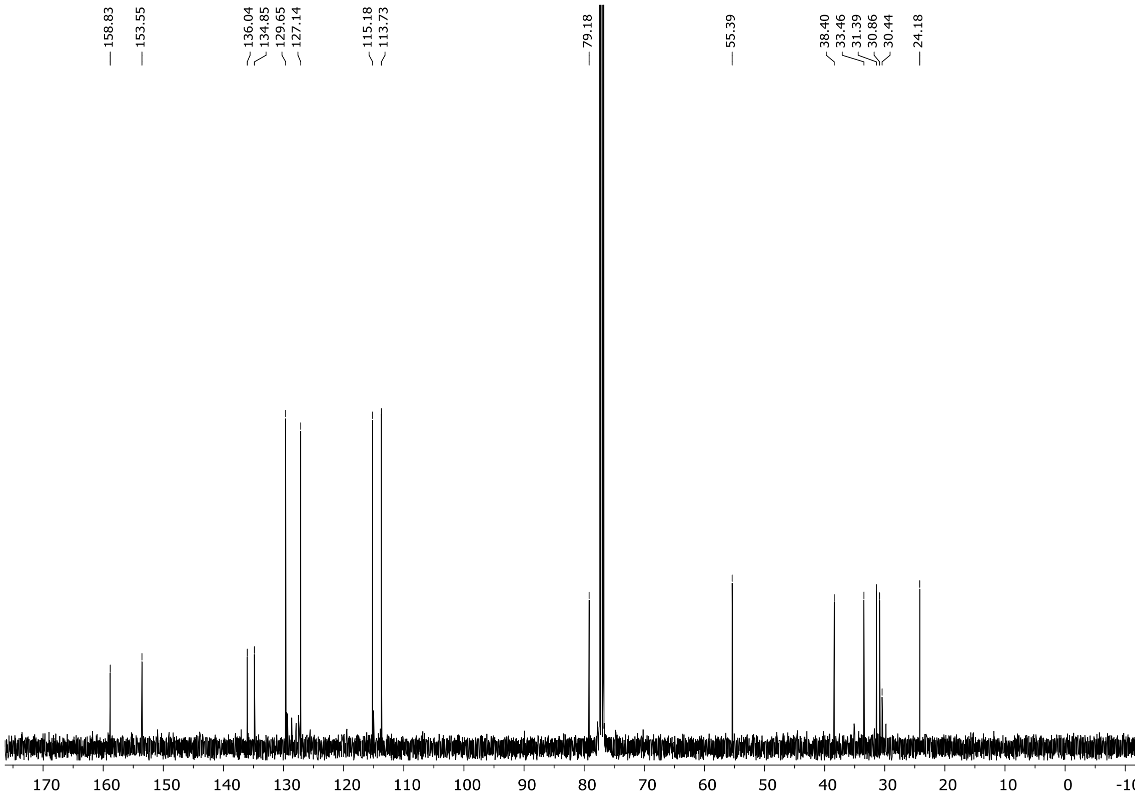

Bis(4-(trifluoromethyl)phenyl)phosphine oxide 134

^{1}H-NMR

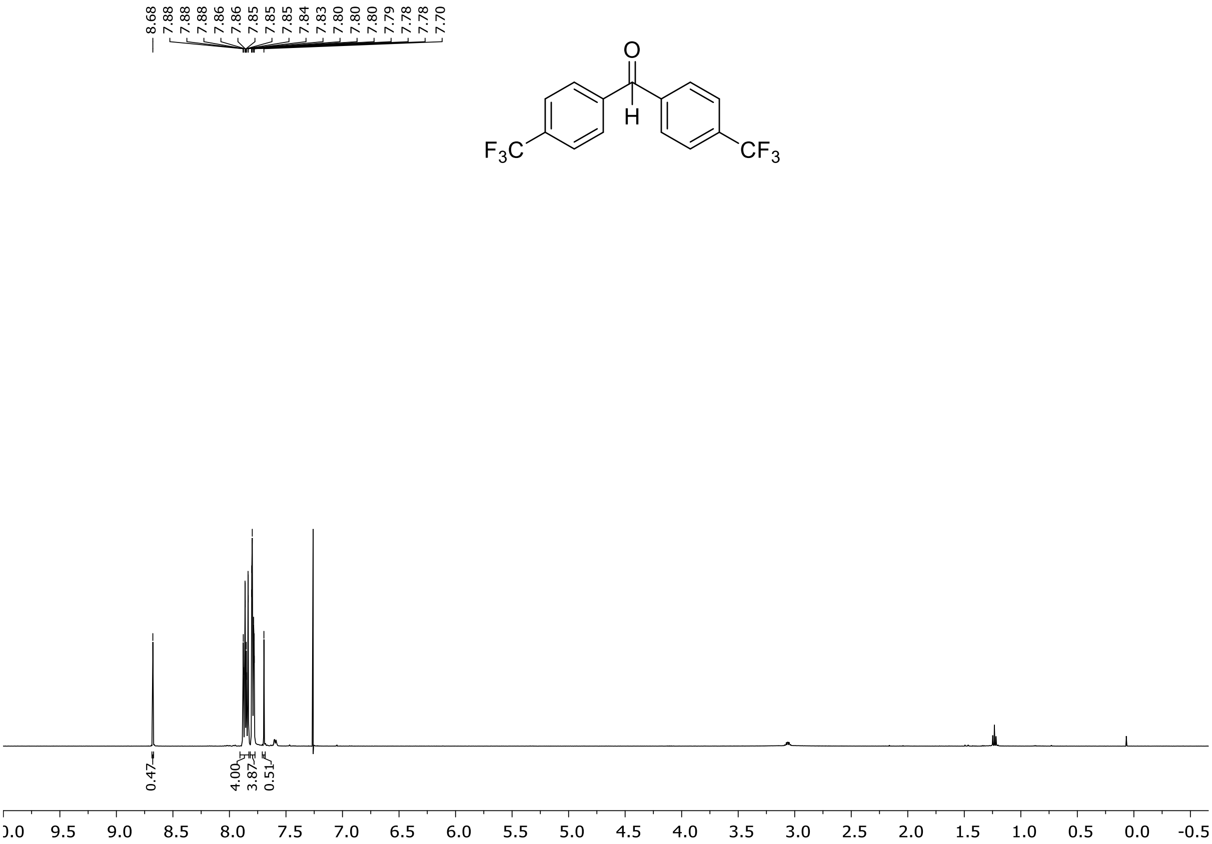

^{13}C-NMR

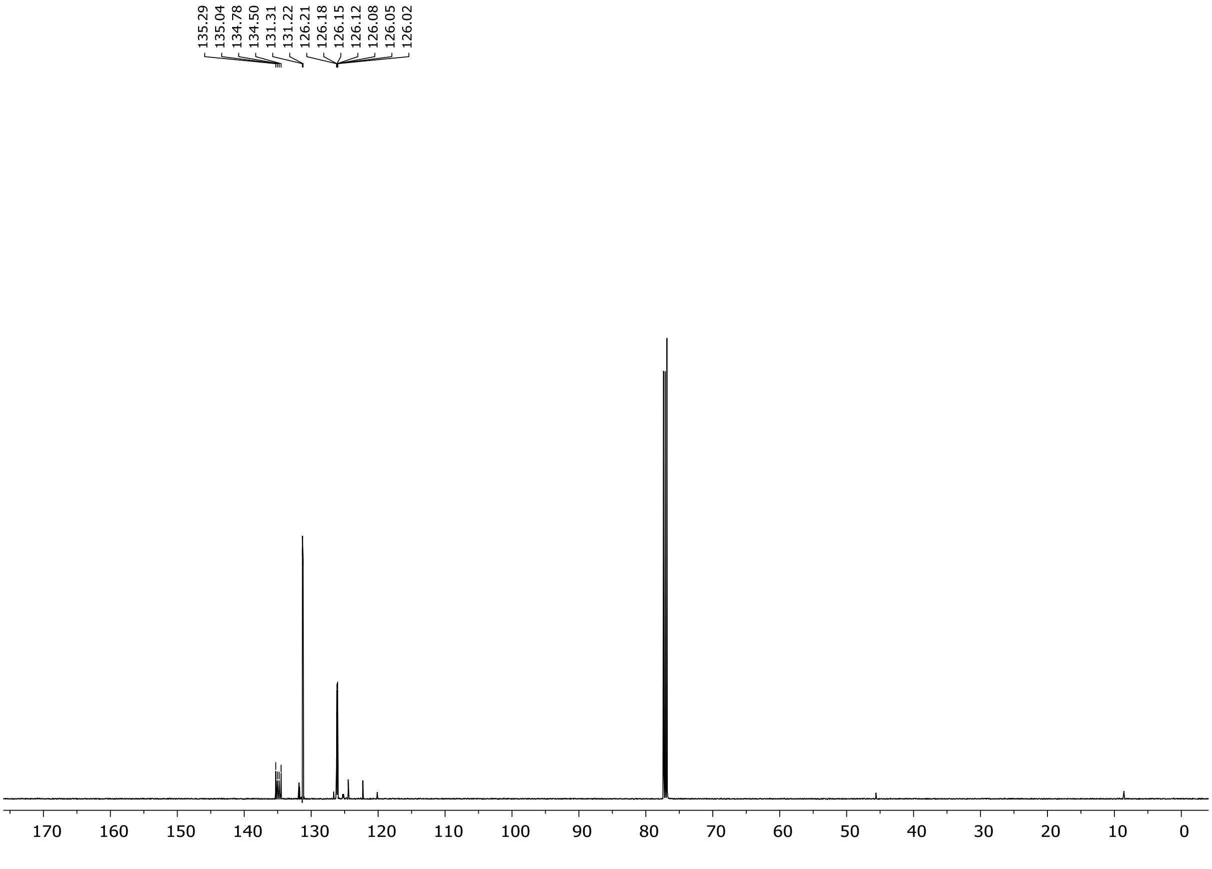

^{19}F-NMR

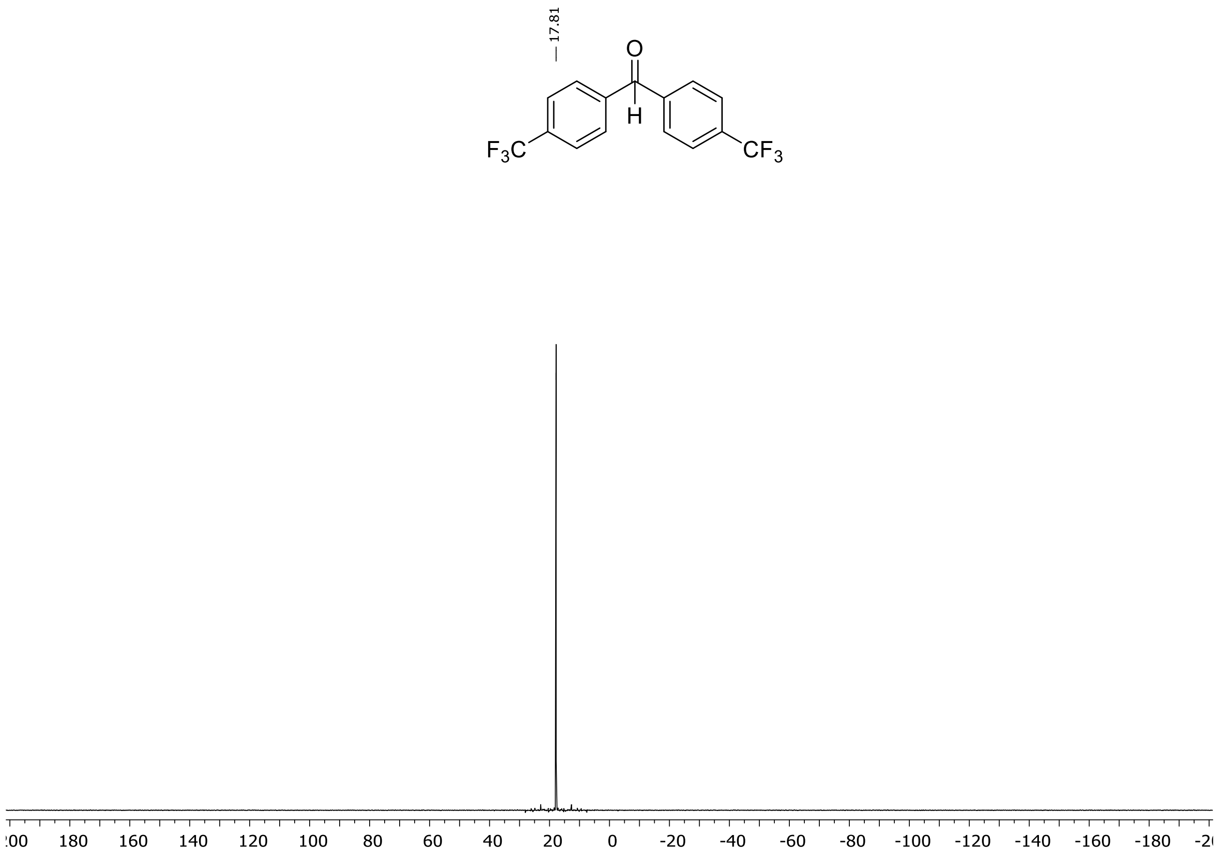

^{31}P-NMR

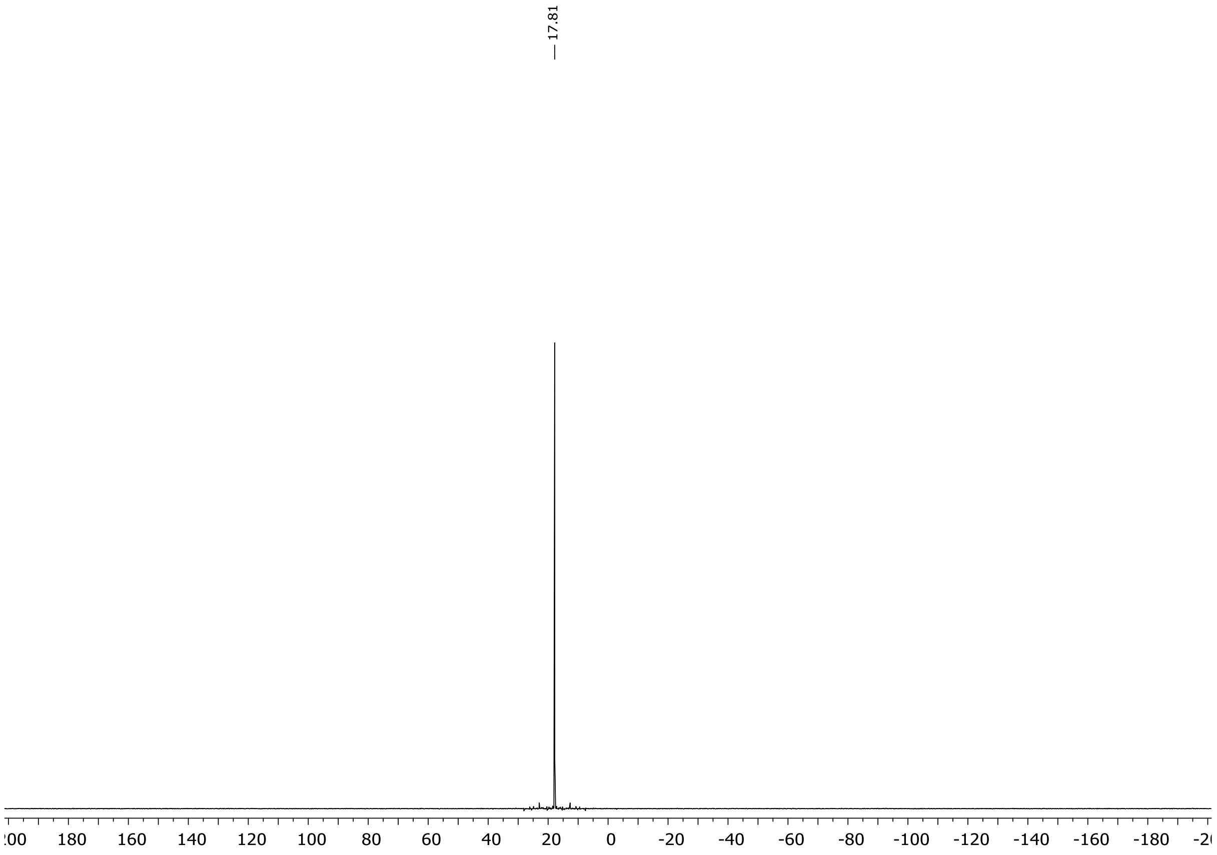

1, 1`-Bis[bis[*4*-(trifluoromethyl)phenyl]phosphino]ferrocene 135

^{1}H-NMR

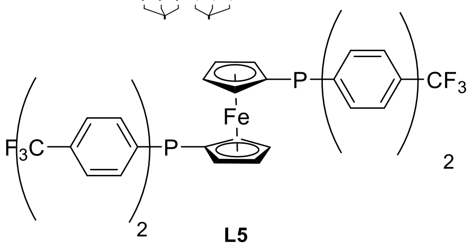

L5
$C_{38}H_{34}F_{12}FeP_2$
836.46 g/mol

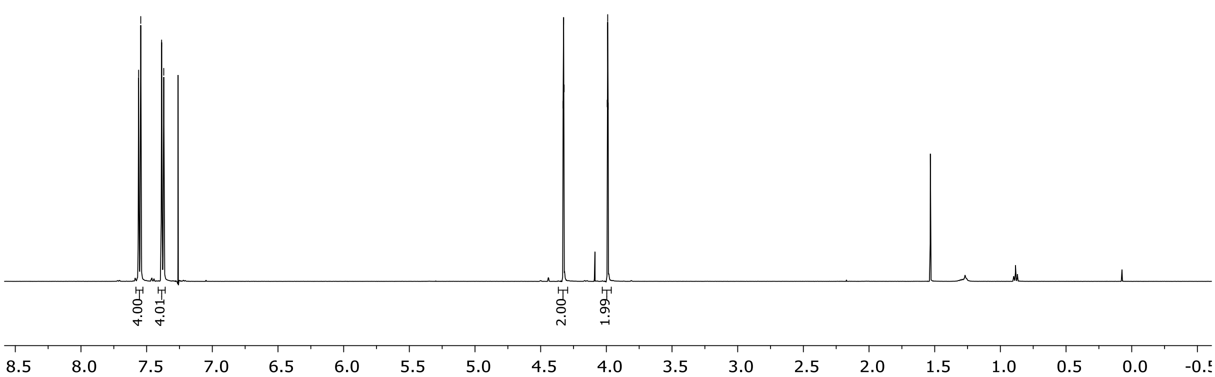

^{13}C-NMR

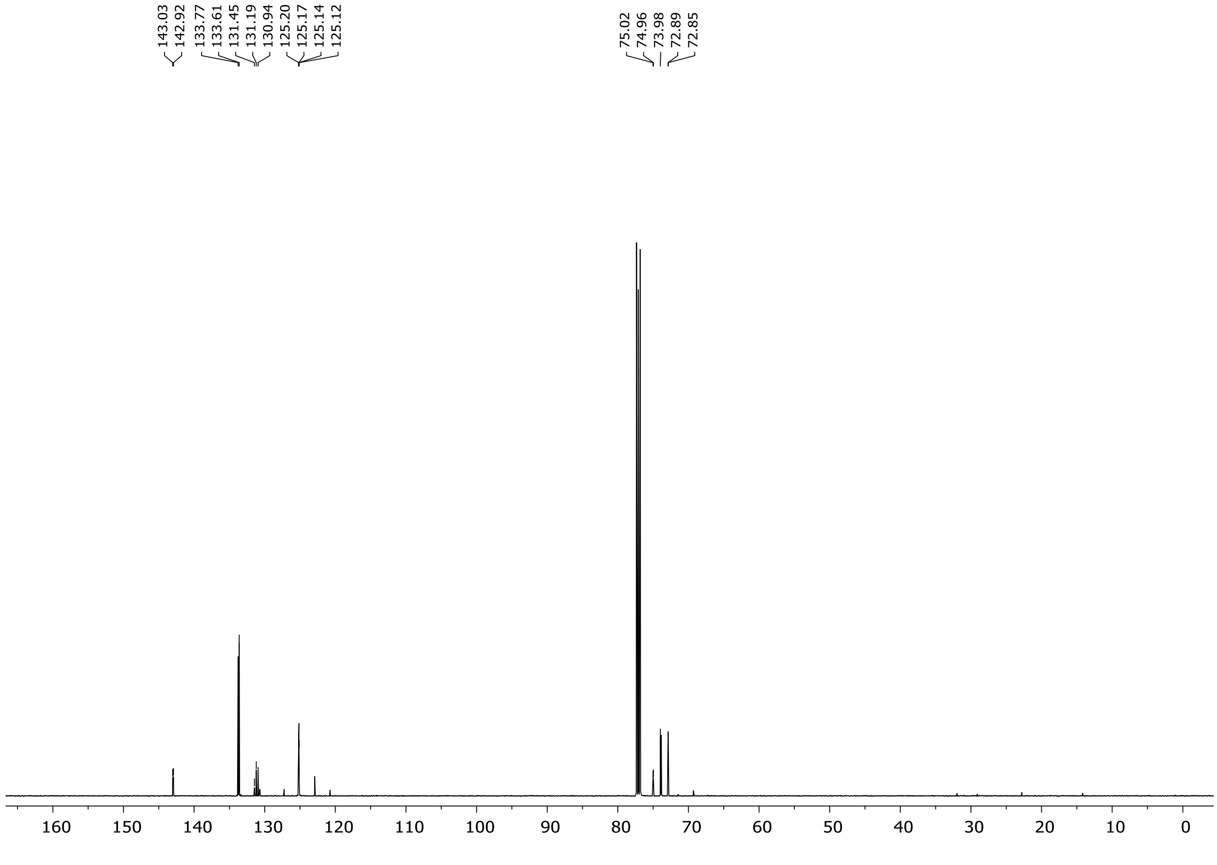

^{19}F-NMR

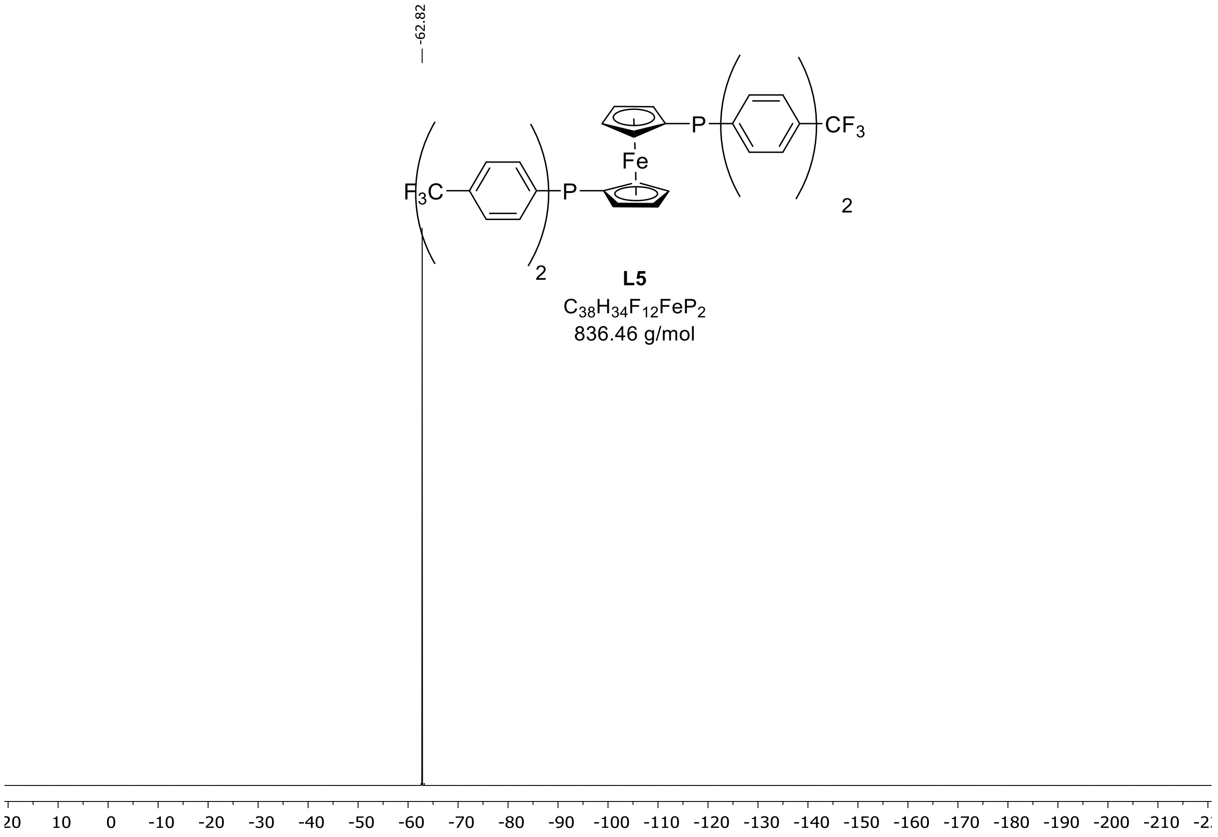

^{31}P-NMR

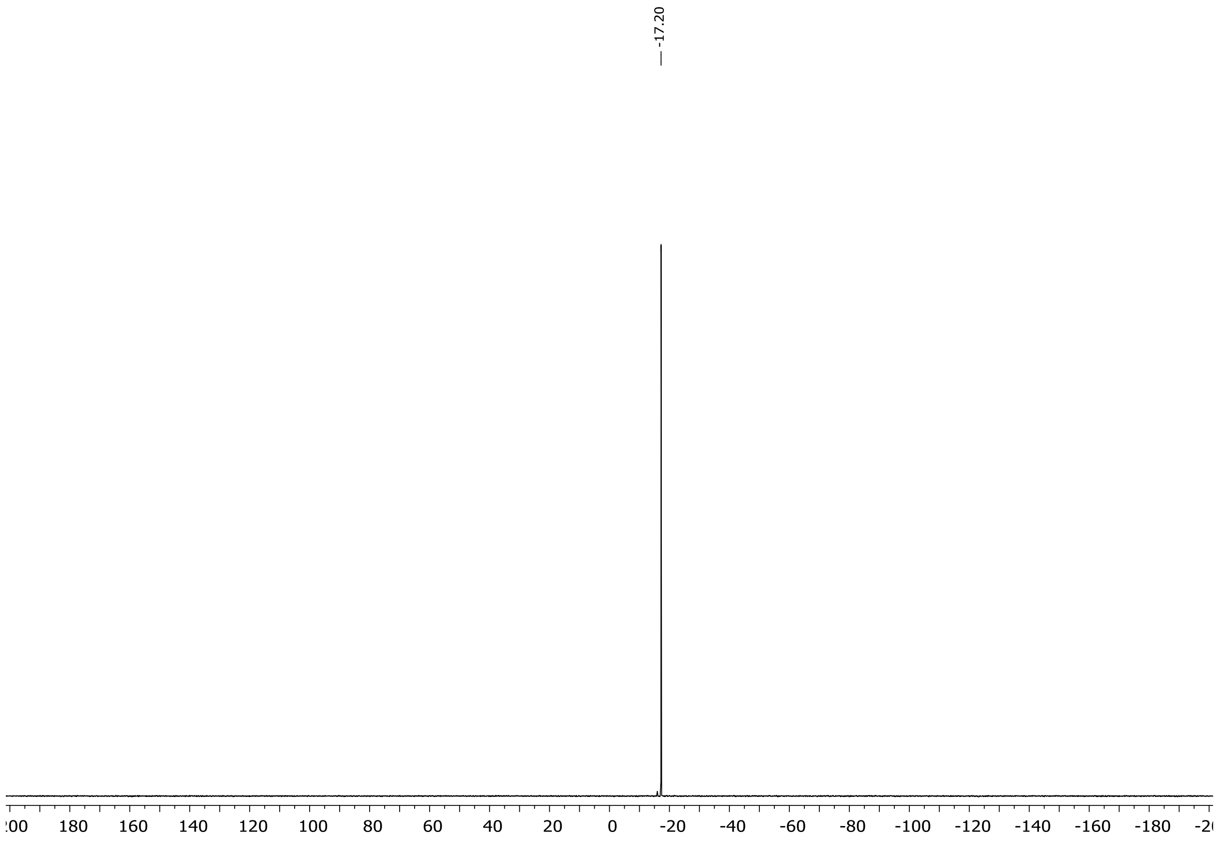

bis(3,5-bis(trifluoromethyl)phenyl)phosphine oxide136

^{1}H-NMR

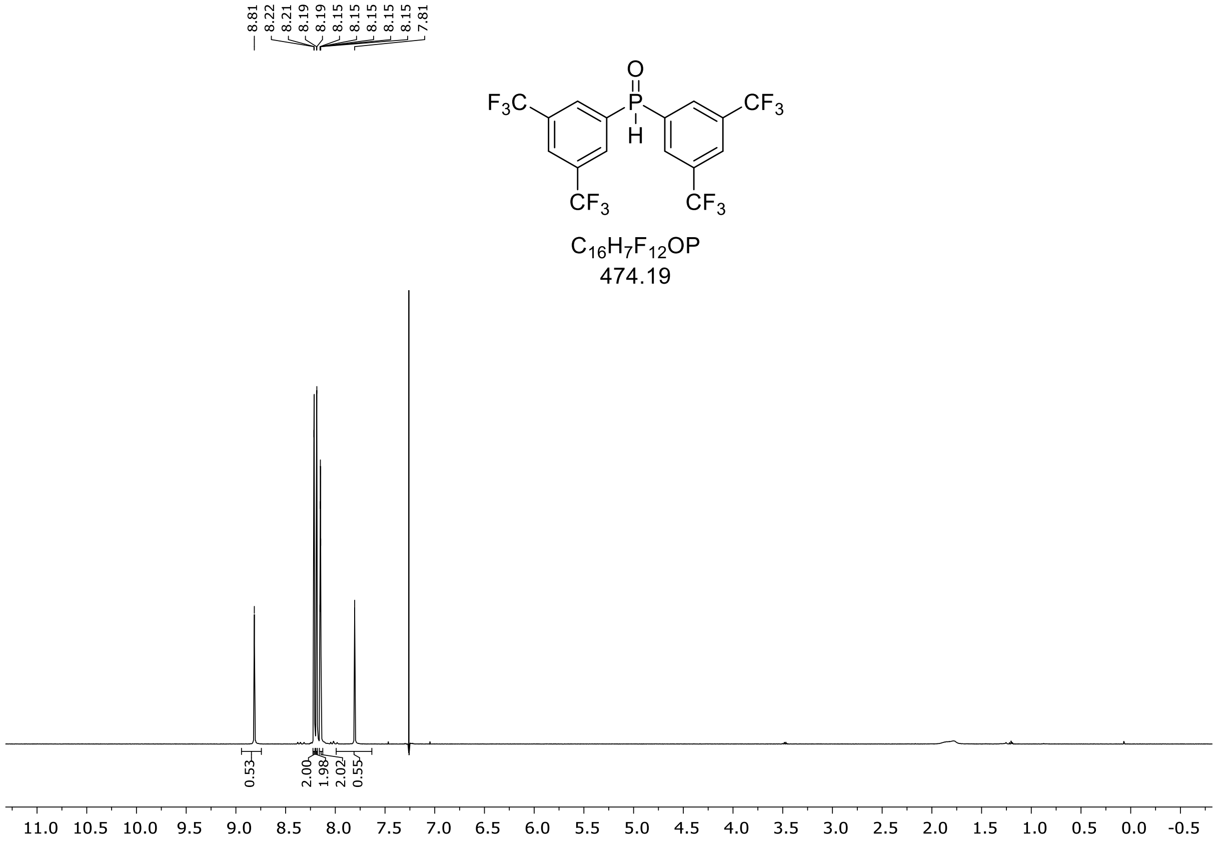

^{13}C-NMR

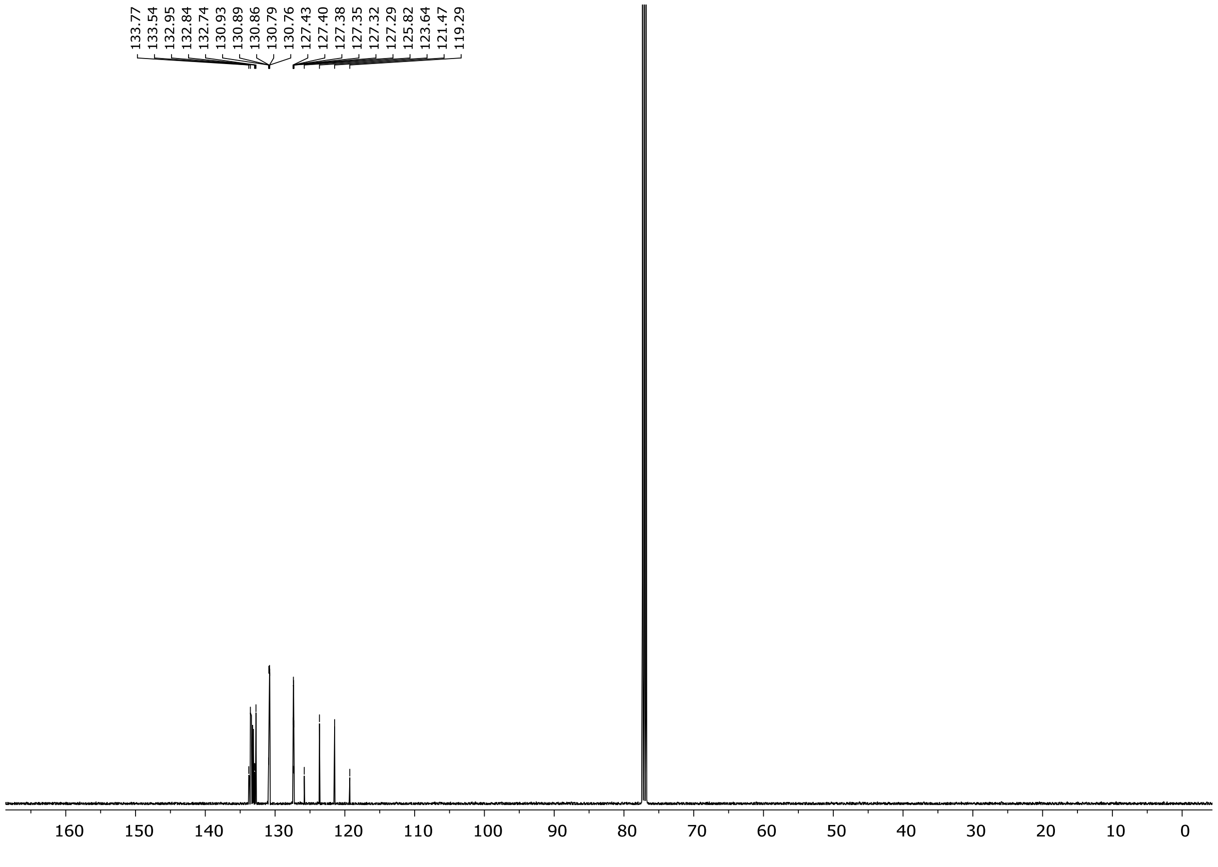

^{19}F-NMR

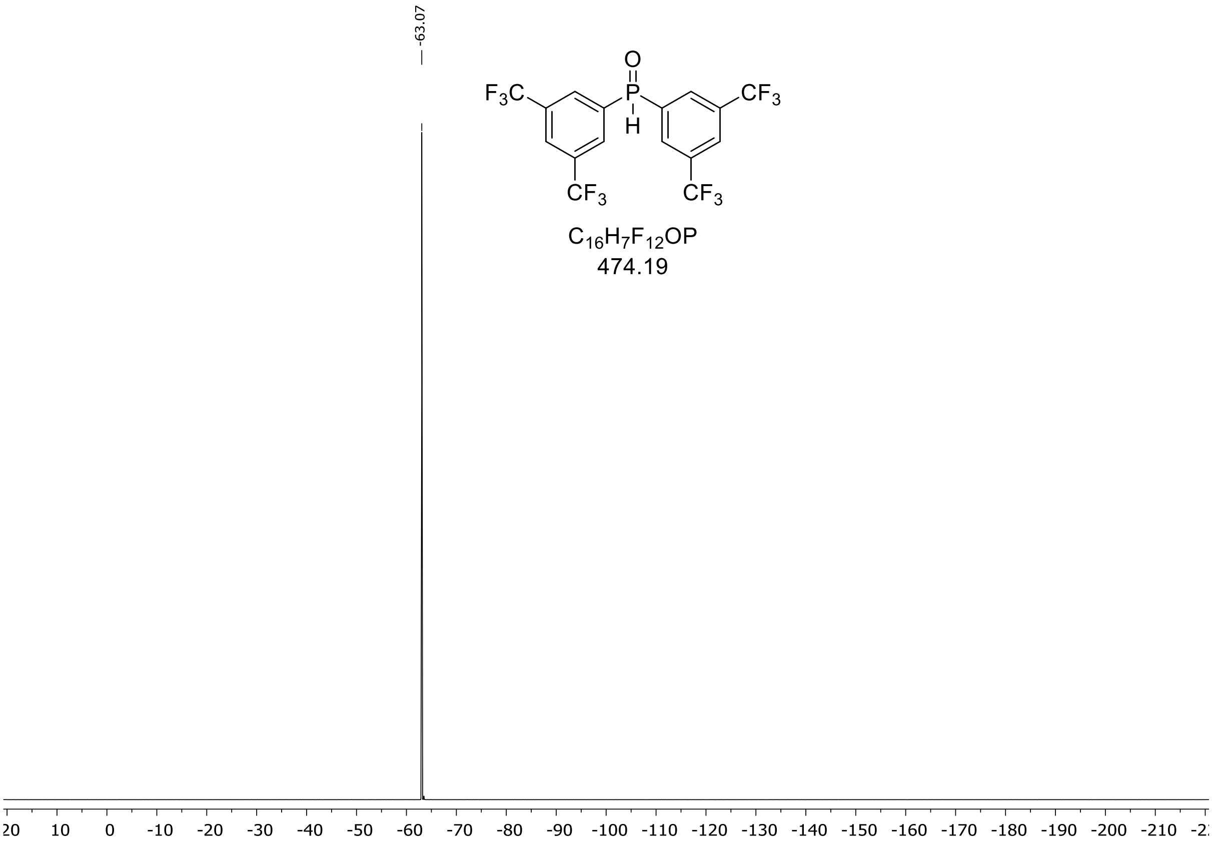

^{31}P-NMR

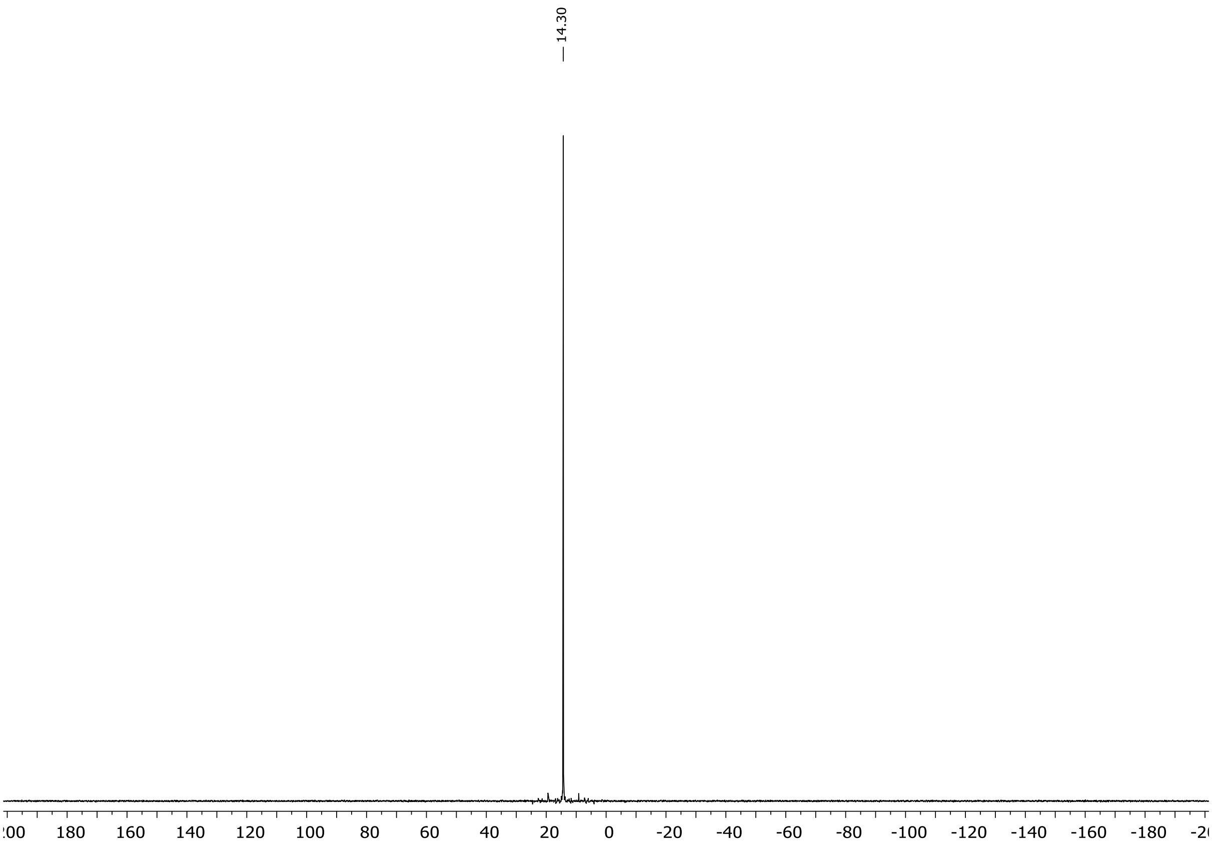

1,1′-Bis[bis[3,5-bis(trifluormethyl)phenyl]phosphino]ferrocen (L5) 137

^{1}H-NMR

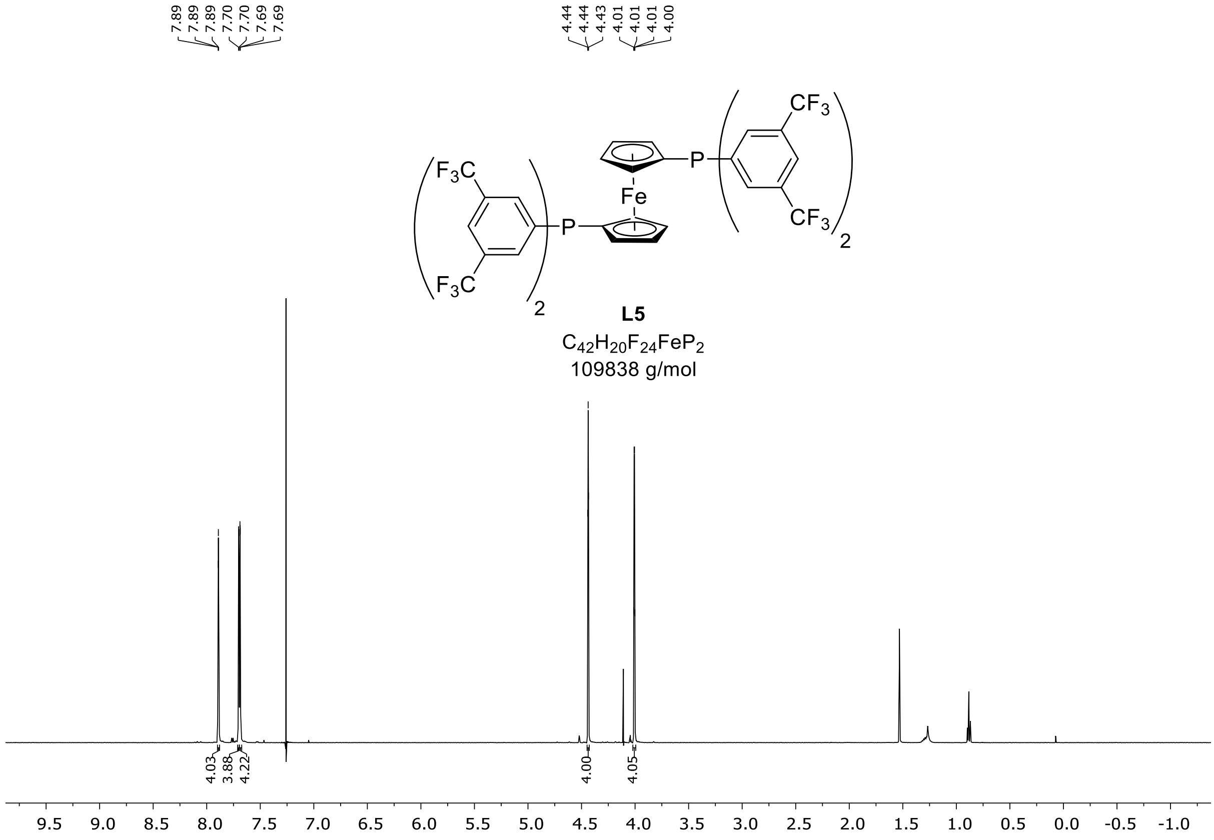

^{13}C-NMR

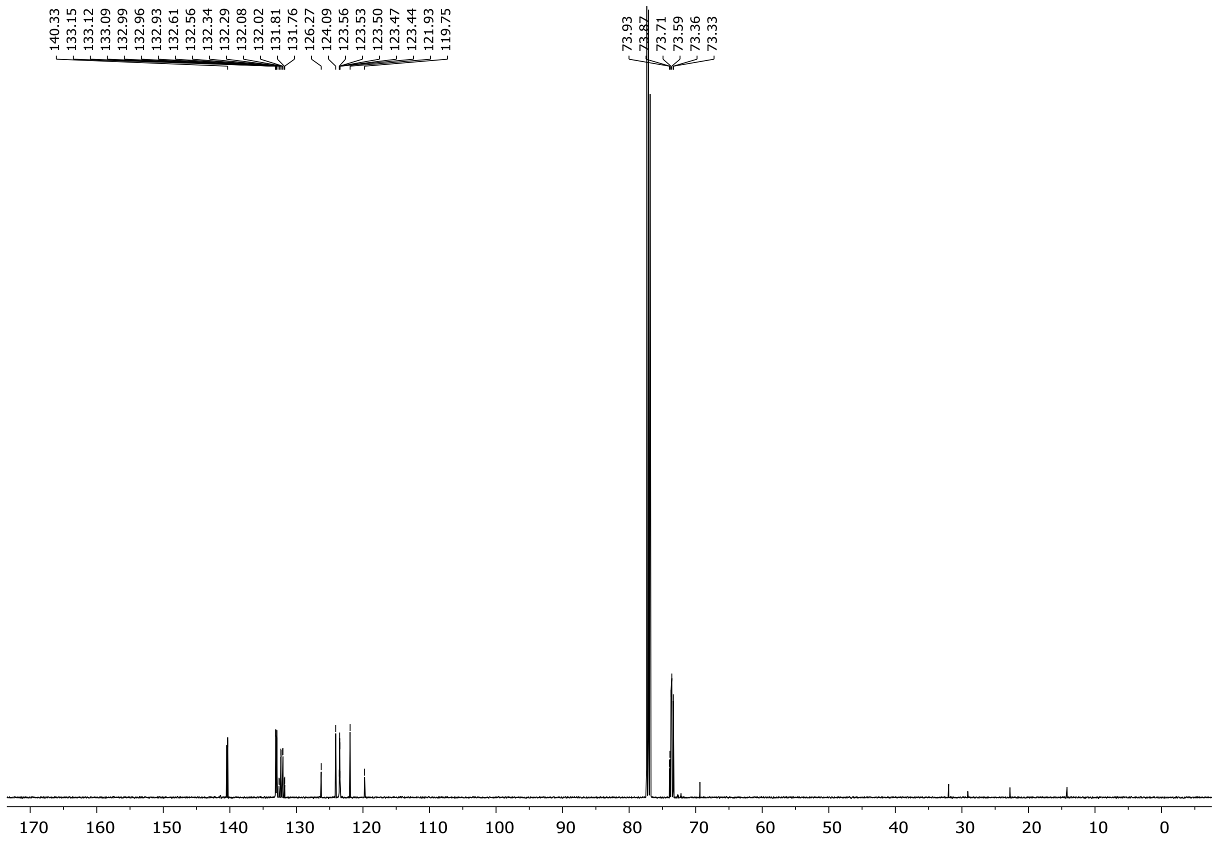

^{19}F-NMR

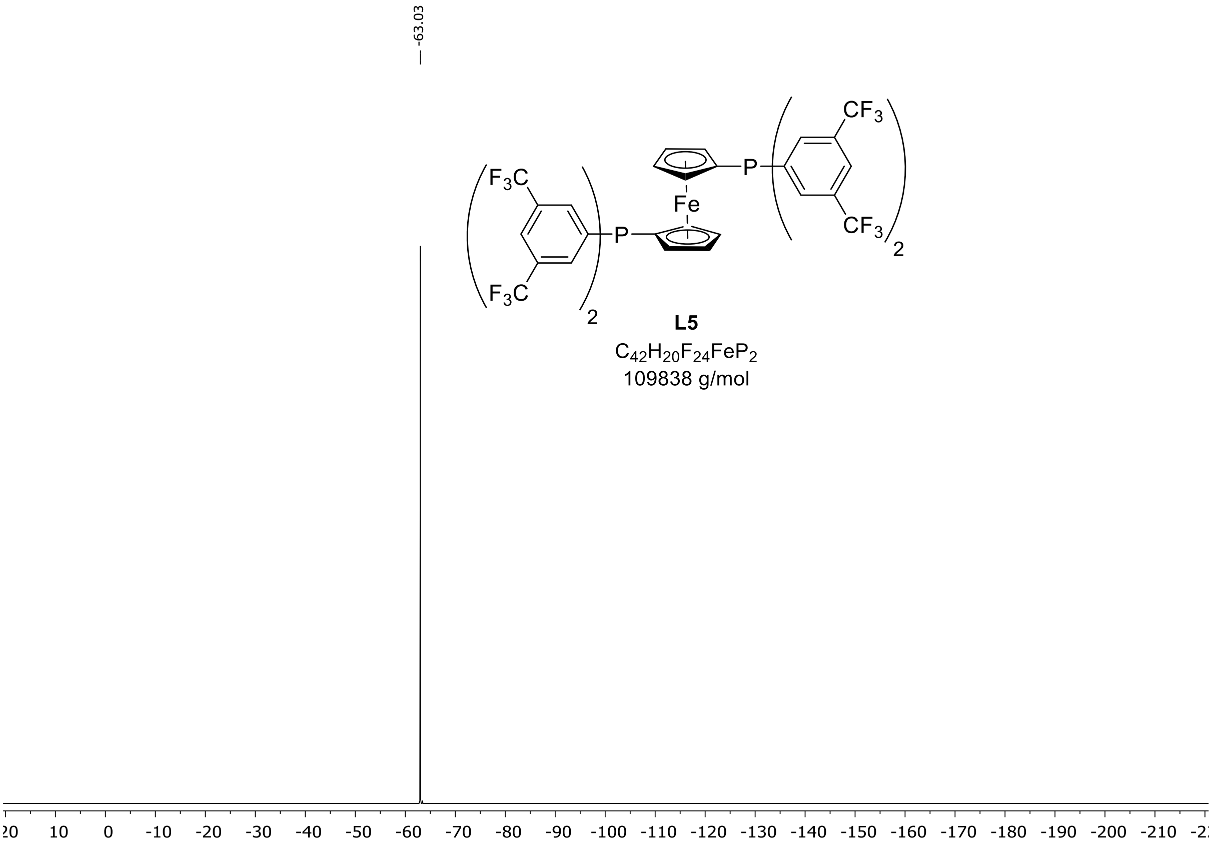

L5
$C_{42}H_{20}F_{24}FeP_2$
109838 g/mol

^{31}P-NMR

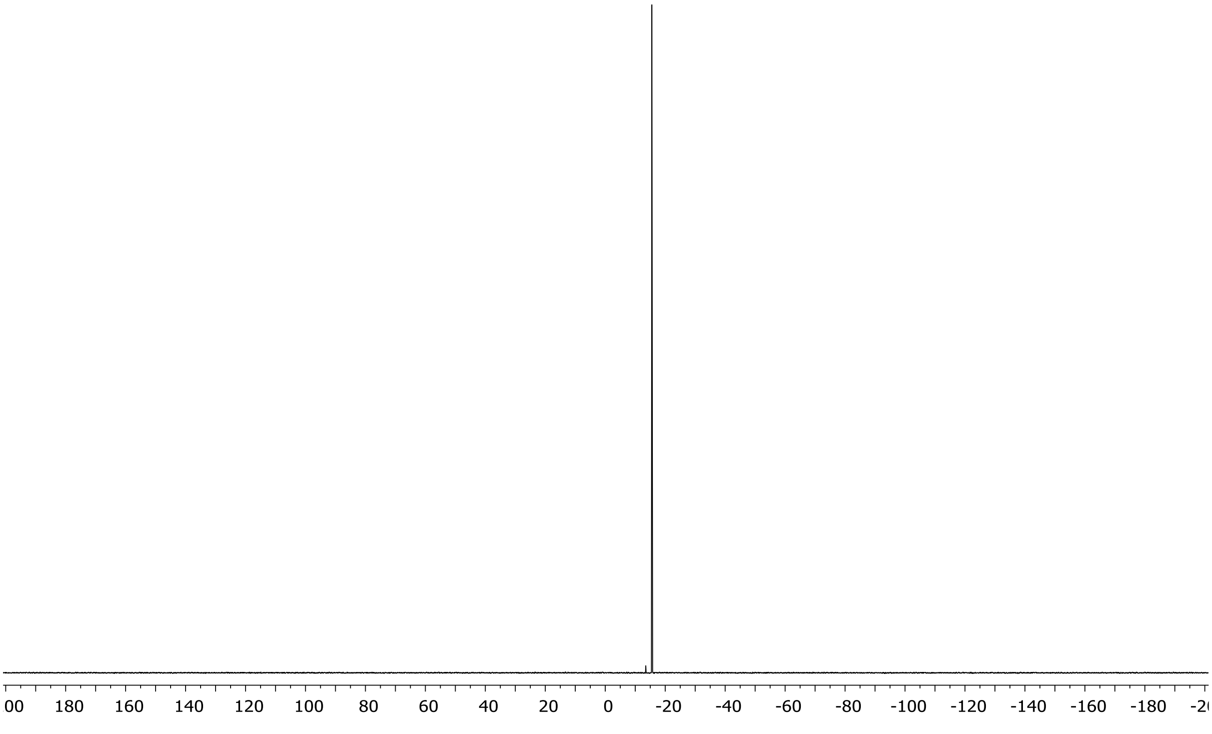

13 GC-analysis

GC – analysis of *rac* and *S*-3-methylhepta-5,6-dienoate

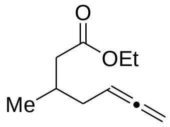

$C_{10}H_{16}O_2$
168.24

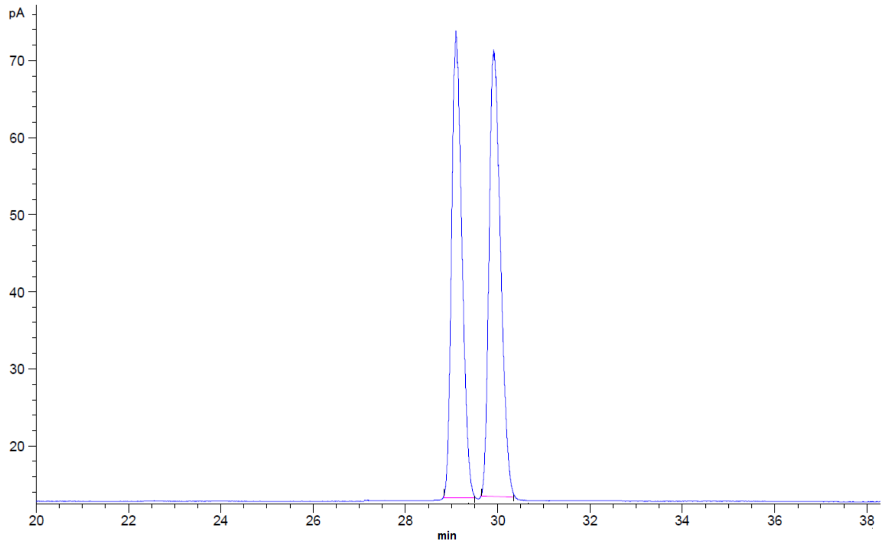

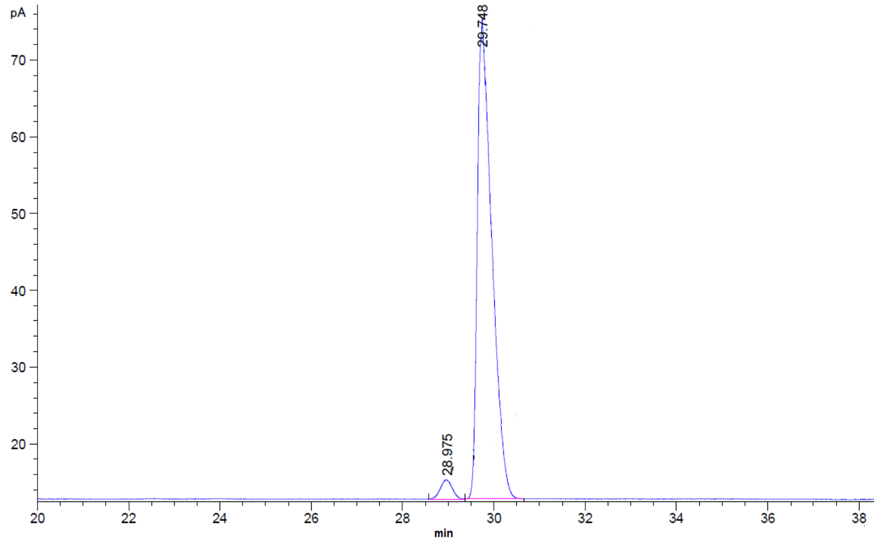

Spectra	Peak Index	t [min]	Area [%]
A	1	29.005	49.17
A	2	30.048	50.83
B	1	28.975	3.17
B	2	29.748	96.83

GC – analysis of rac and *R*, S 4-methyl-2-vinyltetrahydro-2H-pyran

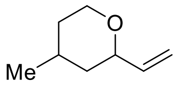

$C_8H_{14}O$
126.19

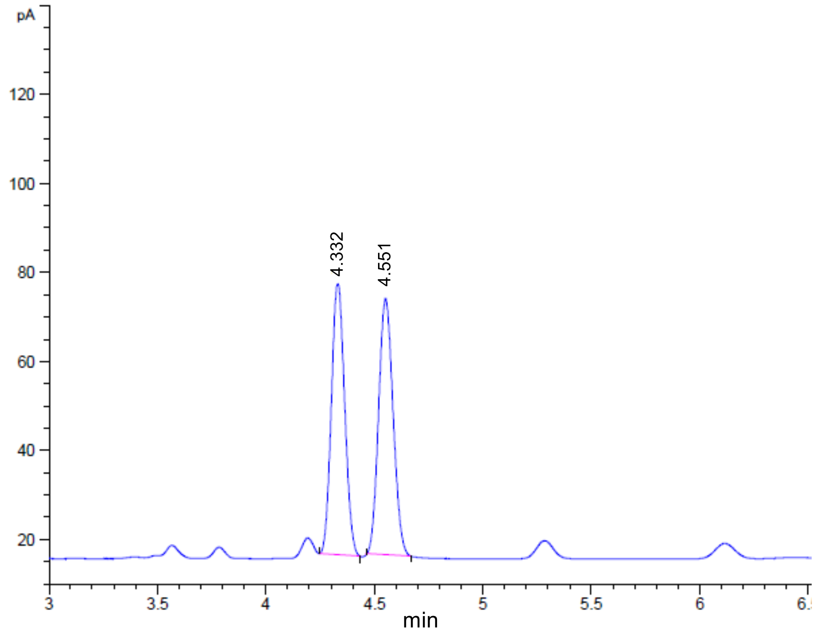

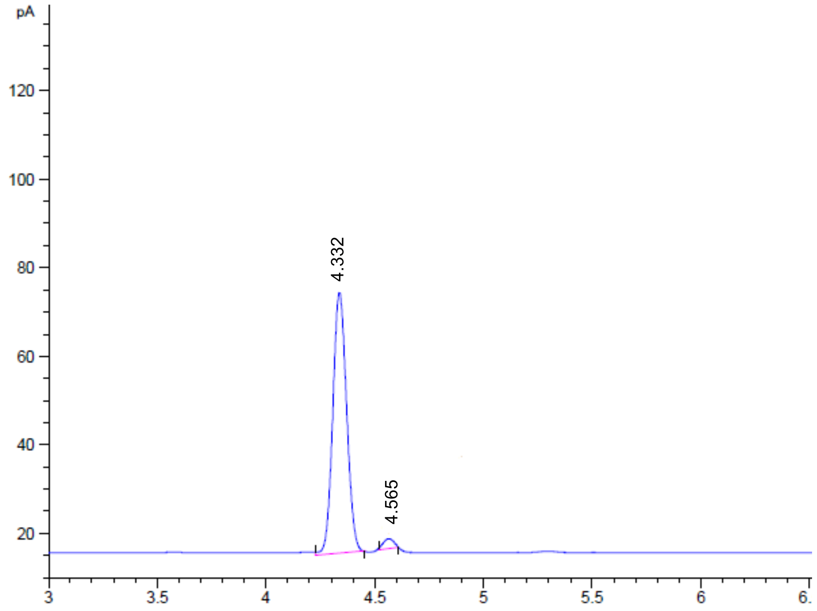

Spectra	Peak Index	t [min]	Area [%]
A	1	4.332	49.28
A	2	4.551	50.71
B	1	4.338	97.23
B	2	4.565	2.77

GC – analysis of *rac* and *S* ethyl 3-methylnona-5,6-dienoate

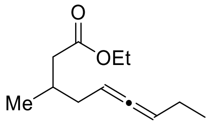

$C_{12}H_{20}O_2$
196.29

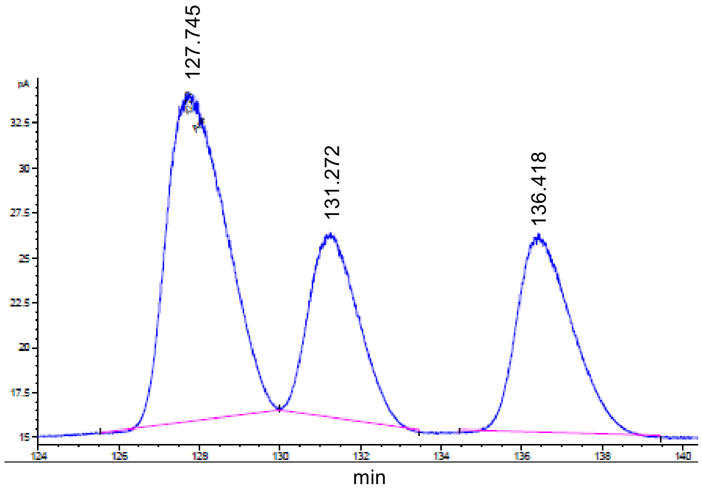

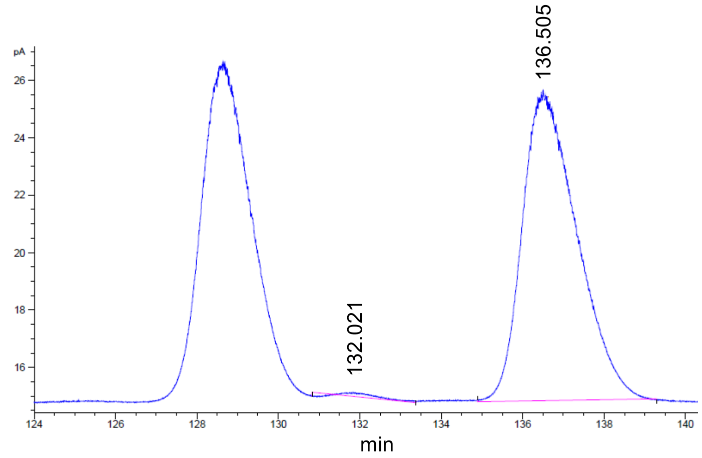

Spectra	Peak Index	t [min]	Area [%]
A	1	127.745	49.37
A	2	131.272	23.10
A	3	136.418	27.53
B	1	132.021	0.78
B	2	136.505	99.22

GC – analysis of rac and (2*S*,4*R*)-2-((E)-but-1-en-1-yl)-4-methyltetrahydro-2H-pyran

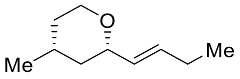

$C_{10}H_{18}O$
154.25

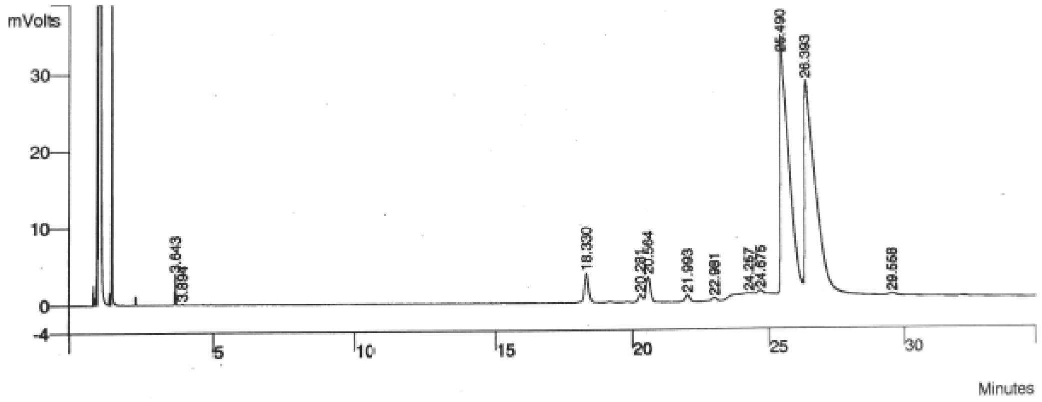

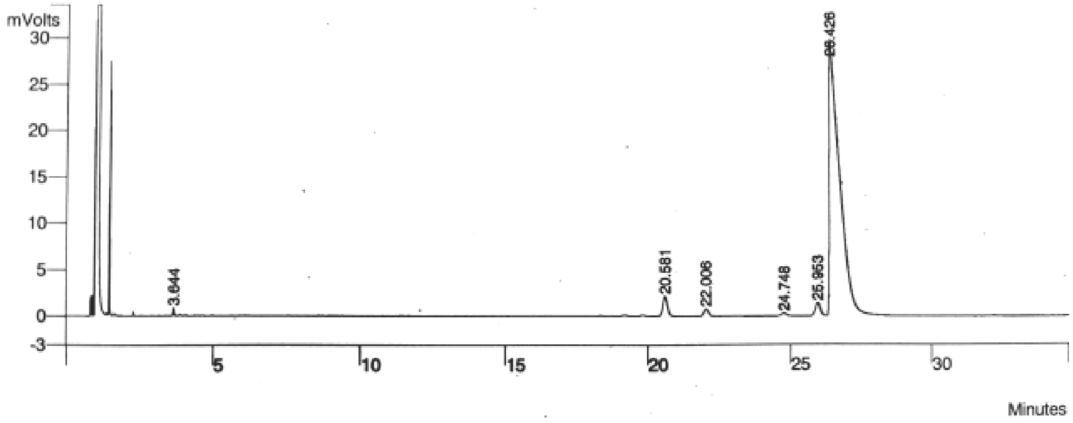

Spectra	Peak Index	t [min]	Area [%]
A	1	25.490	50.52
A	2	26.393	49.82
B	1	132.021	2.09
B	2	136.505	97.91

HPLC – analysis of rac and (-)-centrolobine

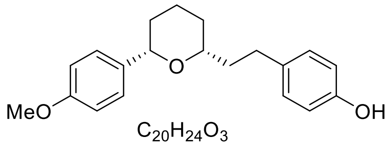

$C_{20}H_{24}O_3$
312.41

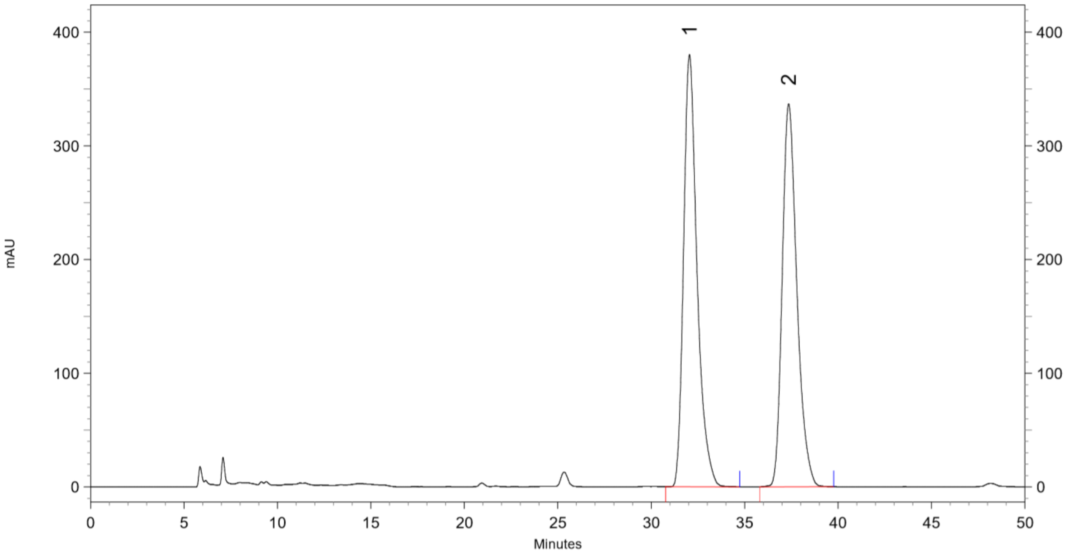

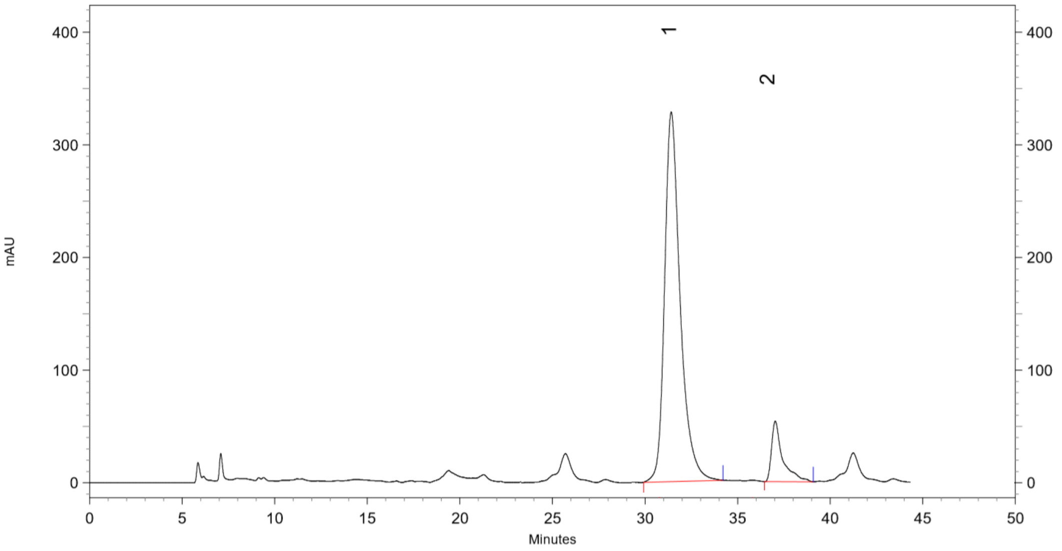

Spectra	Peak Index	t [min]	Area [%]
A	1	32.047	50.65
A	2	37.060	49.35
B	1	31.983	5.21
B	2	37.009	95.89

14 References

[1] P. Spreider, A. Haydl, M. Heinrich, B. Breit Angew. Chem. 2016, 128, 15798-15802; Angew. Chem. Int. Ed. 2015, 55, 15569-15573.
[2] J. P. Schmidt, B. Breit, Chem. Sci. 2019, 10, 3074-3079
[3] NMR-Spectra of diastereomeric mixture. Overlapping signals were discussed as multiplet.
[4] NMR-Spectra of single diastereomer.
[5] NMR-Spectra of *syn*-product. Some spectra are containing traces of anti-product signals from this compound were not analysed separately. Overlapping signals were discussed as multiplet.
[6] NMR-Spectra of *syn*-*E*-product. Some spectra are containing traces of *syn*-*Z*-product, signals from this compound were not analysed separately. Overlapping signals were discussed as multiplet.
